# Evolution and classification of Ser/Thr phosphatase PP2C family in bacteria: Sequence conservation, structures, domain distribution

**DOI:** 10.1371/journal.pone.0322880

**Published:** 2025-05-19

**Authors:** Hang Li, Rui Li, Haoyue Yu, Youhuan Zhang, Hong Feng

**Affiliations:** 1 Sichuan Key Laboratory of Molecular Biology and Biotechnology, Chengdu, China; 2 College of Life Sciences, Sichuan University, Chengdu, China; University of California Riverside, UNITED STATES OF AMERICA

## Abstract

Serine/threonine kinases (STKs) and serine/threonine phosphatases (STPs) are widely present across various organisms and play crucial roles in regulating cellular processes such as growth, proliferation, signal transduction, and other physiological functions. Recent research has increasingly focused on the regulation of STKs and STPs in bacteria. STKs have been well studied, identified and characterized in a variety of bacterial species. However, the role of STPs in bacteria remains less understood, and the number of proteins characterized is limited. It has been found that most of the STPs characterized in bacteria were Mg^2+^/Mn^2+^ dependent 2C protein phosphatases (PP2Cs), but the evolutionary relationship and taxonomic distribution of bacterial PP2C phosphatases were still not fully elucidated. In this study, we utilized bacterial PP2C phosphatase sequences from the InterPro database to perform a phylogenetic analysis, categorizing the family into five groups. Based on this classification, we examined the evolutionary relationships, species distribution, sequence and structural variations, and domain distribution characteristics of bacterial PP2C phosphatases. Our analysis uncovered evidence of a common evolutionary origin for bacterial PP2C phosphatases. These findings advance the understanding of PP2C phosphatases, offering valuable insights for future functional studies of bacterial serine/threonine phosphatases and aiding in the design of targeted therapeutics for pathogenic bacteria.

## 1. Introduction

In order to respond and adapt to constantly changing environments, the protein post-translational modification system (PTMs) of cell provides robust adaptive and regulatory mechanisms [1]. Among them, reversible protein phosphorylation is one of the key PTM mechanisms regulating many cellular processes [2,3]. Protein phosphorylation on serine (Ser), threonine (Thr), and tyrosine (Tyr) residues has been shown to effectively regulate various cell activities in bacteria, archaea, and eukaryotes [4]. Serine/threonine kinases (STKs) and their cognate phosphatases (STPs) are prevalent enzymes in eukaryotes and have been extensively studied [5,6]. In bacteria, the two-component systems (TCSs), comprising histidine kinases (HK) and response regulators (RR), were considered the primary phosphorylation modification system [7]. However, with the development of whole genome sequencing technology in recent years, analysis of bacterial genomes has revealed the widespread presence of STK and STP genes in many bacteria [8]. Several studies have shown that many bacterial species encode STKs that have structural similarities to eukaryotic STKs, thus called as “eukaryotic-like” serine/threonine kinases (eSTKs) [9]. Interestingly, most of them were shown to catalyze the phosphorylation of Ser and Thr residues in bacterial proteins, but not Tyr [10]. In addition, many studies indicated that phosphorylation signaling systems consisting of eSTKs and the cognate phosphatases (eSTPs) also played important roles in bacterial growth control, malignant transformation, DNA damage response, and regulatory networks that regulated bacterial pathogenicity and drug resistance [11–13]. Notably, eSTK/eSTP systems and TCSs can interact to regulate multiple bacterial cell activities [9,14], and TCS histidine kinases can be served as the target protein of eSTKs [15,16].

In some cases, eSTKs and eSTPs are genetically linked. It has been shown in bacteria that the genes of both enzymes were regulated by the same operon, and the two enzymes played an antagonistic role in regulating certain bacterial physiological processes [17–19]. There is also evidence that phosphatases can dephosphorylate their cognate kinases *in vitro* [12]. However, up to now, less bacterial STPs have been discovered and biochemically characterized than STKs, and it was assumed that the number of STPs present in bacterial cells was considerably smaller than that of STKs [4]. A typical example was *Mycobacterium tuberculosis*, in which 11 STKs and only one STP have been found so far [20]. Although previous studies have primarily focused on eSTKs, recent research has revealed that eSTPs also played a regulatory role in various cellular functions of pathogenic bacteria [21]. Some studies have even suggested that the impact of eSTP mutant on bacterial physiology was more pronounced than that of eSTK mutant [22,23]. As a result, there is growing interest and extensive research into the role of eSTPs. Current investigations indicated that eSTPs may be involved in regulating biological processes such as metabolic adaptation, cell division, cell wall biosynthesis, spore formation, antimicrobial resistance, and virulence in bacteria [12,21,24]. For example, serine/threonine phosphatase Stp, the only functional STP in *Staphylococcus aureus*, was involved in the regulation of cell wall synthesis [22], virulence factor production [25] and antibiotic tolerance [26]. Huemer et al. (2023) showed that the deletion of Stp increased the growth delay and phenotypic heterogeneity of *S. aureus* under different stresses, contributing to antibiotic tolerance [26]. Kant et al. (2023) showed that PhpP (an eSTP in *Streptococcus pneumoniae*) was involved in the regulation of cytoplasmic division, polysaccharide capsular homeostasis, and virulence factor production*,* which could be used as an important target for development of novel anti-pneumococcal infection drugs [23]. In addition, Prp C was the most studied serine/threonine phosphatase in *Bacillus anthracis* and regulated the growth and survival of the bacteria [27]. However, in a recent study, Gangwal et al. (2022) characterized a second eSTP in *B. anthracis,* PrpN, and found that PrpN regulated toxin synthesis by dephosphorylation of the global transcription factor CodY [28].

STPs found in both bacteria and archaea are members of two families, including phosphoprotein phosphatases (PPPs) and metal ion dependent phosphatases (PPMs) [12]. The PPP family can be further categorized into three distinct subfamilies: PP1, PP2A, and PP2B, whereas the PPM family primarily consists of the PP2C which is a major subfamily and pyruvate dehydrogenase [29,30]. Both PPPs and PP2Cs form similar structural folds and are dephosphorylated at Ser and Thr residues of the substrate protein [31,32]. Among them, PPPs bound to additional protein regulatory subunits to confer functional specificity; while the PP2C gene evolved additional domains to confer its unique role [33]. In addition, PP2Cs were resistant to classical PPP inhibitors such as Okada acid [34]. Both PPPs and PP2Cs have been identified in bacteria as key regulators of specific life activities through the dephosphorylation of Ser/Thr residues on their substrates [35]. Notably, unlike PP2Cs, bacterial PPPs may even specifically phosphorylate Tyr residues [35]. In eukaryotes, PPP family protein phosphatases represent the most abundant class of serine/threonine phosphatases [30]. However, in bacteria, the majority of serine/threonine phosphatases (STPs) with well-characterized biochemical properties and physiological functions belong to the PP2C family [12]. Eukaryotic PP2C phosphatases comprise numerous subtypes and play critical roles in regulating diverse cellular processes, including cell cycle control, differentiation, immune responses, and metabolism [36]. In contrast, bacterial PP2C phosphatases have been relatively understudied, leaving significant gaps in our understanding of their functional roles and evolutionary significance. It has been found that in the genome of most bacteria, the phosphatases encoded upstream of Ser/Thr kinases were of the PP2C-type Ser/Thr phosphatases [37]. Therefore, this study primarily focused on the PP2C subfamily of phosphatases to further investigate the function of eSTPs. Based on the PP2C family PF13672 in the Interprot database, the evolutionary relationship and species classification of bacterial PP2C phosphatases were analyzed, revealing the common origin of bacterial PP2C phosphatases. This study identified that a family of STPs in eukaryotes originated from bacteria, providing an important basis for revealing the origin of STP in eukaryotes. In addition, bacterial PP2C phosphatases were divided into five groups and their sequences and structure differences were analyzed. Finally, the types of the other domains of PP2C phosphatases were analyzed, perhaps to explain the functional differences of PP2C phosphatases in different bacteria. These results provided a theoretical basis for studying the function of PP2C and designing target drugs.

## 2. Materials and methods

### 2.1. Database search and retrieval of PP2C family sequences

All amino acid sequences belonging to the protein phosphatase 2C entry (PF13672) from the InterPro database (https://wwwebi.ac.uk/interpro/entry/) were retrieved, totaling 57,520 sequences as of 5 March 2024. Of these, 53,574 sequences were from eubacteria, 439 from archaea, 3,063 from eukaryotes, and 444 from unclassified organisms. For further analysis, these sequences were downloaded from the UniProt database (https://www.uniprot.org). This provided additional comprehensive information, including sequence ID, annotation, and species origin. In addition, the sequences with lengths less than 200 and greater than 1,500 were excluded for the following reasons. According to the crystal structure of the PP2C phosphatase that has been studied so far, it is found that the phosphatase with biological function contains at least 200 amino acids [38–40]. Further, the majority of sequences within the PF13672 protein family were less than 1000 amino acids in length. The presence of unusually long sequences may negatively impact the results of multiple sequence alignments. Consequently, 56,494 sequences were maintained and applied to next steps.

### 2.2. Multiple sequence alignment

The easy-cluster module of MMseqs2 [41] was used to process 56,494 sequences to remove the redundancy sequences, hence 3,573 unique sequences were obtained (S1 File). In addition, Muscle [42] was used to generate candidate multiple sequence alignments. To perform multiple sequence alignment on the dereplicated protein sequences, the Super5 algorithm was selected as the alignment strategy. Super5 was known for its efficiency and accuracy in aligning large sets of sequences, making it an appropriate choice for handling the extensive dataset of protein sequences derived from the PF13672 family.

### 2.3. Phylogenetic tree inference

The alignment was trimmed using trimAL [43] with automated selection on “gappyout” mode, which used information based on gaps’ distribution. FastTree [44], which can handle alignments with up to a million of sequences in a reasonable amount of time and memory, infered approximately-maximum-likelihood phylogenetic trees from pruned protein sequences. FastTree has five stages: heuristic neighbor-joining, reducing the length of the tree, maximizing the tree’s likelihood with NNIs, local support values [44]. Finally, the phylogenetic tree was visualized using iTOL version 6 [45]. To further analyze the candidate sequences (3,573 sequences), a Python script was employed to extract the species origin. Subsequently, the family lineage of each species was determined using the TaxonKit [46] software. To illustrate the association between sample abundance and species, a chord diagram was created using the ‘circlize’ package in R.

### 2.4. WebLogo analysis of conservative residues in PP2C family

The sequences corresponding to the five groups inferred from the evolutionary tree were extracted, and sequence alignment and pruning were carried out to obtain the core sequence of each group. Quantitative residue conservation at key sequence positions in these alignments was determined using reporting features of GeneDoc (http://www.psc.edu/biomed/genedoc). Alignments were used at the WebLogo3 [47] to generate graphical representations of sequence conservation. The core sequences of PP2C were divided into 11 motifs according to the results of sequence conservation. Finally, the sequences corresponding to motifs at different positions were input into WebLogo3, and 11 motifs corresponding to 5 groups were obtained, and their similarities and differences were compared.

### 2.5. PP2C structural comparisons

Representative protein structures from five phylogenetic groups were retrieved from the UniProt database, including Q8VQA1 (PDB ID: 2PK0) from *S. agalactiae*, P9WHW5 (PDB ID: 1TXO) from *M. tuberculosis*, A0A1L7GEB2 (AlphaFold ID: AF-A0A1L7GEB2-F1) from *Streptomyces sp.* TN58, A0A7V6HC85 (AlphaFold ID: AF-A0A7V6HC85-F1) from *Clostridiaceae bacterium*, and P76395 (AlphaFold ID: AF-P76395-F1) from *Escherichia coli*. In addition to the PP2C from *S. agalactiae* (PDB ID: 2PK0) and *M. tuberculosis* (PDB ID: 1TXO), which represented the known structure in bacteria, the other three proteins were predictive structures obtained from AlphaFold (https://alphafold.com/). The five representative protein structures were then superimposed using ChimeraX [48] software, and the amino acid residues from the active center were labeled based on the sequence alignment results. Finally, ESPript [49] was used to analyze the primary to quaternary protein structure information of five representative proteins.

### 2.6. Domain architectures of PP2C

HMMSCAN, a program for HMMER [50], was used to compare the input protein sequences to the HMM domain library (Pfam A) and returned the domains each protein contains. Following the processing of the annotation file, the types of domains outside the catalytic domain of PP2C were identified and quantified. The distribution of these domains across different species was visualized using string graphs, generated with the ‘circlize’ package in R. Additionally, domain structure maps predicted by HMMER were constructed using R.

## 3. Results

### 3.1. Phylogenetic analysis and classification of PP2C

**Fig 1:**
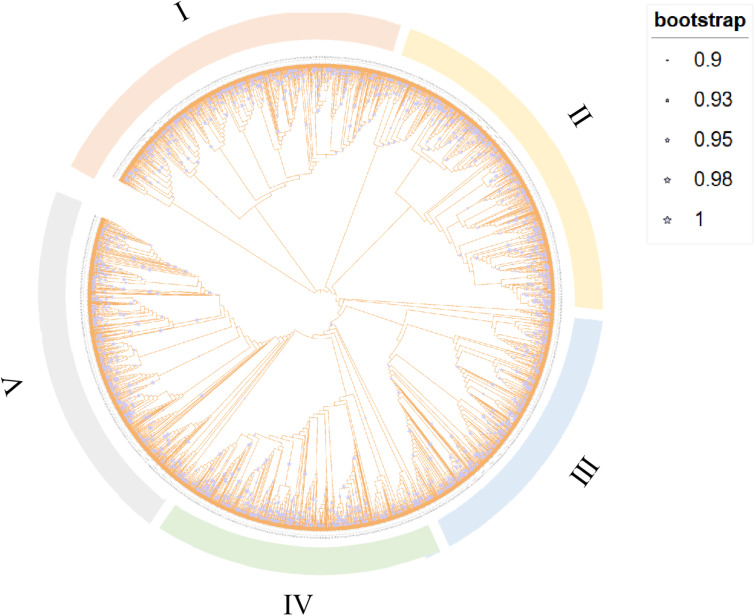
Phylogenetic tree of bacterial PP2C phosphatases. The Bootstrap value, obtained from 1000 repetitions, shows nodes with > 90% support as a light blue five-pointed star. The orange, yellow, blue, green, and gray circles on the outer layers of the evolutionary tree represent groups I, II, III, IV, and V, respectively.

For phylogenetic analysis of PP2C, 57,520 amino acid sequences were retrieved from the PP2C family entry PF13672 at InterPro. Most of these sequences come from bacteria across almost all the taxa. Initially, the redundant sequences were reduced by the clustering program, and those less than 200 or more than 1,500 were also removed. Finally, 3,573 nonredundant sequences (S1 File) were used to construct a comprehensive phylogenetic tree by the maximum likelihood method (Fig 1).

The resulting phylogenetic tree revealed a common origin of bacterial PP2C phosphatases, suggesting that these genes may have come from a common ancestor and have conserved key biological functions throughout evolution. In addition, the phylogenetic tree showed that the PP2C family can be divided into five distinct groups, named as Group I, II, III, IV, and V (Fig 1). The number of sequences within these groups are 801, 764, 612, 647, and 749, separately, indicating a relatively uniform distribution among the groups.

**Fig 2:**
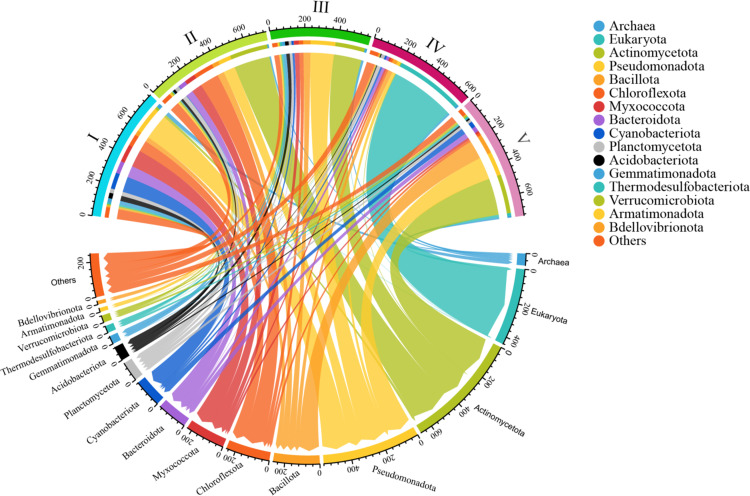
The distribution of phylum levels of PP2C phosphatases in five groups. The distribution of PP2C phosphatases (PF13672) in eukaryotes, archaea, and bacterial phyla was illustrated by a string diagram. Each chord connected different subtypes and phyla, with the color of the chord representing different phyla. The color map on the right represented eukaryotes, archaea, and the total number of the top 17 bacterial phyla. In the upper part of the string diagram, the blue, light green, green, purple, and pink circles represented Groups I, II, III, IV, and V, respectively. The thickness of the chord reflected the relative abundance or number of species.

To further investigate the distribution of PP2C phosphatases in bacteria, the species abundance at the phylum-level genetic divergence of sequences in five groups was analyzed (Fig 2). Our analysis encompassed bacterial PP2C phosphatases from 83 bacterial phyla as classified by NCBI, though only the top 14 phyla with the highest representation were depicted. The results showed that PP2C phosphatases were widely distributed among bacterial taxa, with notable abundance in Actinomycetota, Pseudomonadota, and Bacillota. There was considerable variability in species distribution across different groups. Specifically, Groups I and II exhibited higher species abundance relative to the other groups. The PP2C phosphatases in Actinomycetota mainly belonged to Groups II, III and V, while the PP2C phosphatases in Pseudomonadota were distributed across all five groups. This suggested that PP2C phosphatases from Group I within Pseudomonadota may represent ancestral forms of the PP2C family. Nearly half of the PP2C phosphatases from Bacillota were classified within Group V, while those from Bacteroidota and Cyanobacteriota were predominantly found in Group I. Additionally, PP2C phosphatases from archaea and eukaryotes were also considered. Archaeal PP2C phosphatases were distributed across all five groups, albeit with lower representation, whereas eukaryotic PP2C phosphatases were exclusively found in Group IV.

### 3.2. Conservation of sequence motifs throughout PP2C evolution

**Fig 3:**
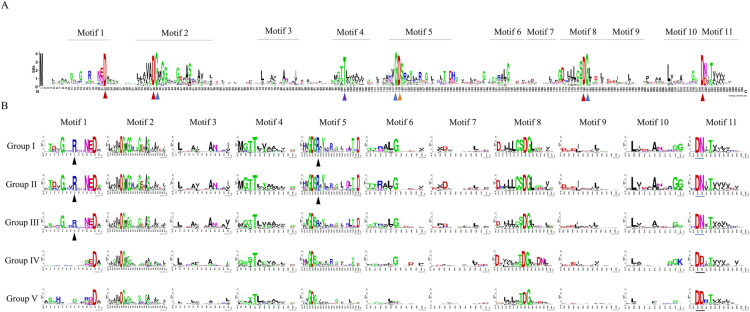
Sequence logos for conserved motifs in bacterial PP2C phosphatases. The height of different amino acids represents repeatability. The scale bar at the bottom indicates the length of the motif protein sequence. **(A)** Sequence conservation and motifs distribution in the core catalytic domain of 3573 candidate sequences. Eleven motifs were labeled on the sequence logo. Conserved Asp in Motifs 1, 2, 8, and 11 were marked with red triangles, while those in Motif 5 were marked with purple triangles. Conserved Gly and Thr residues are indicated by blue and yellow triangles, respectively. **(B)** Sequence logos for conserved motifs in PP2C phosphatases of five groups, respectively. Conserved Arg residues were labeled with black triangles in motifs. The conserved “DN” sequence and “DD” sequence in Motif 11 were marked with blue and black short lines, respectively.

Early analyses of PP2C protein sequences identified that this protein family was characterized by eleven conserved motifs, containing a set of conserved amino acids including four Asp residues (in Motifs 1, 2, 8, and 11) [51]. To investigate the conservation of PP2C phosphatases in bacteria, we aligned candidate sequences and visualized the results using WebLogo [47]. Consistent with the results of previous studies, conserved Asp residues were found in Motifs 1, 2, 8, and 11. Notably, our analysis also revealed that Asp residues were conserved in Motif 5, as illustrated in Fig 3A. In addition, Gly residues were found to be relatively conserved in Motifs 2, 5, and 8, positioned adjacent to the conserved Asp residues. This spatial arrangement suggested a potential combined role in maintaining the enzyme’s structural and functional integrity. It is worth noting that Thr residues in Motif 4 were also conserved.

To analyze the similarities and differences among the motifs in the five groups, sequences from each group were examined using WebLogo (S2 Fig), resulting in the identification of 11 distinct motifs for each group (Fig 3B). In general, Groups III, IV and V exhibited less conservation at many sites compared to Groups I and II, which may reflect the diversity of PP2C phosphatases during the later stages of evolution. In Groups I, II, and III, Gly, Arg and Asp in Motif 1 were conserved to some extent, while in Groups IV and V, these amino acids were no longer conserved. Additionally, Group I and II exhibited an extra conserved Arg in Motif 5, which was not conserved in Groups III, IV and V. In Motif 11, the conserved “DN” sequence in Groups I, II, and III was replaced by the conserved “DD” sequence in Groups IV and V, which involved a change in charge that could potentially impact the active site.

### 3.3. Structural characteristics and differences of bacterial PP2C phosphatases

**Fig 4:**
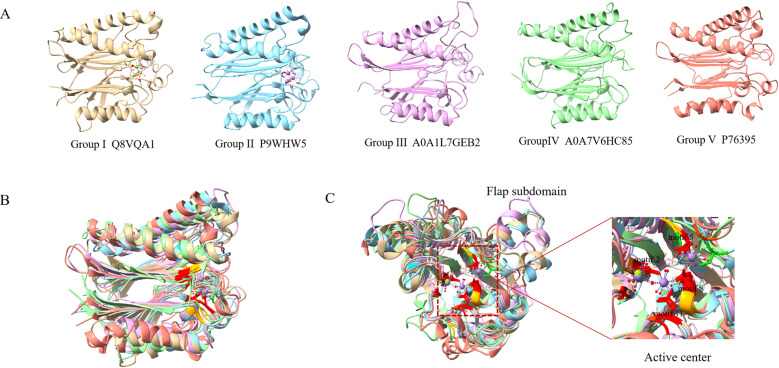
Comparison of bacterial PP2C phosphatase structures. **(A)** Known and predicted structures of representative proteins from five distinct groups: Q8VQA1 (PDB ID: 2PK0, shown in golden) from *Streptococcus agalactiae*, P9WHW5 (PDB ID: 1TXO, shown in blue) from *Mycobacterium tuberculosis*, A0A1L7GEB2 (AlphaFold ID: AF-A0A1L7GEB2-F1, shown in purple) from *Streptomyces sp. TN58*, A0A7V6HC85 (AlphaFold ID: AF-A0A7V6HC85-F1, shown in green) from *Clostridiaceae bacterium*, and P76395 (AlphaFold ID: AF-P76395-F1, shown in coralline) from *Escherichia coli*. Metal ions were shown as colored spheres. **(B)** Structural superposition of five representative proteins. The five representative protein structures were superimposed using ChimeraX software, and the amino acid residues from the active center were labeled based on the sequence alignment results. Asp, Asn, Gly, Ser and Thr residues were labeled with red, orange-red, orange, green and yellow respectively. **(C)** Flap subdomain and active center of PP2C phosphatases. The flap subdomain is a frontal extension of alpha-helices outside the catalytic domain. In amplified active centers, motifs involved in the formation of active centers were labeled.

To further study the structural similarities and differences among PP2C phosphatases in the five identified groups, five PP2C phosphatases Q8VQA1 (PDB ID: 2PK0), P9WHW5 (PDB ID: 1TXO), A0A1L7GEB2, A0A7V6HC85, and P76395 corresponding to five groups of Group I, II, III, IV, and V, separately, were selected as representative proteins for detailed structural characterization (Fig 4A). As depicted in result of structure superposition (Fig 4B), bacterial PP2C phosphatases shared common secondary structural units, including a central β sandwich composed of anti-parallel β slices and a pair of anti-parallel helices on either side of the β slices, in an alpha-β-β-α arrangement. Nevertheless, the secondary structural elements shared by the five groups also show differences. As shown in S3 Fig, the number of β-strands in the central β-sandwich varied: Groups I and II had 10 β-strands, Groups III and V had 9 β-strands, and Group IV had 11 β-strands. These differences may underlie functional divergences among these phosphatases. Importantly, the proteins of Groups I, II and III contained frontal extension α- helices in addition to the pair of antiparallel helices flanking the β-sheet, a feature referred to as the flap subdomain in PP2C phosphatases (Fig 4C).

Furthermore, upon examining the superposition diagram with labeled conserved amino acids, we observed that conserved Asp and Gly residues were predominantly clustered at the active central sites within motifs 1, 2, 5, 8, and 11 (Fig 4B). In the active center, conserved Asp residues located within motifs 2, 8 and 11 combined with two manganese ions to form a binuclear metal center (Fig 4C), which was consistent with the active center of eukaryotic PP2C phosphatase. Notably, analysis of the PDB structures of the representative proteins from Groups I and II indicated that the presence of a third metal ion was present in the active center of bacterial PP2C phosphatase. The superposition of the active centers of five protein groups showed that conserved Asp residues in Motif 5 facilitated the binding of the third metal ion in Groups I, II, III, and V, while conserved Ser residues played this role in Group IV (Fig 4C). In addition, comparison of the flap subdomain across the five representative proteins revealed significant similarities among Groups I, II, and III, whereas substantial differences were observed in the flap subdomains of proteins from Groups IV and V. Recent studies suggested that the flap subdomain was crucial for the binding of the third metal ion [40,52]. Consequently, these observations lead us to hypothesize that the third metal ion may be prevalent in the PP2C phosphatases of Groups I, II, and III.

### 3.4. Domain arrangements in bacterial PP2C phosphatases

**Fig 5:**
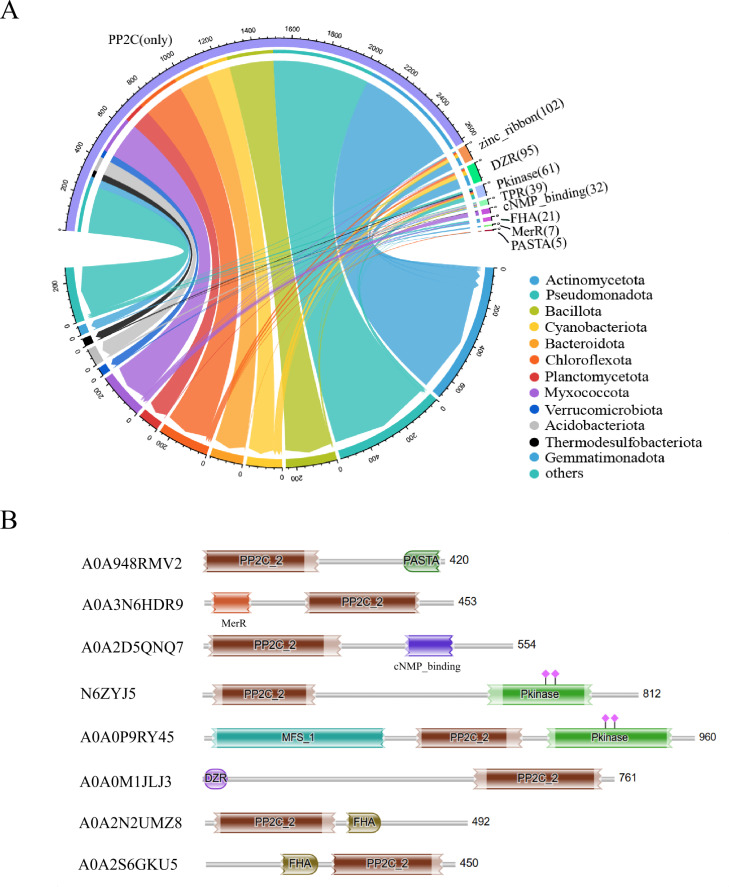
Domain arrangements in bacterial PP2C phosphatases. **(A)** The distribution of domains across different bacterial phyla was visualized using string graphs. The color map on the right represented the total number of the top 12 bacterial phyla. The thickness of the chord reflected the relative abundance or number of species in different domain types. **(B)** Domain arrangements in bacterial PP2C phosphatases. The domain annotation results of the proteins with uniprot IDs: A0A948RMV2, A0A3N6HDR9, A0A2D5QNQ7, N6ZYJ5, A0A0P9RY45, A0A0M1JLJ3, A0A2N2UMZ8, and A0A2S6GKU5. The PP2C, zinc_ribbon, DZR, Pkinase, TPR, cNMP_binding, FHA, MerR, PASTA domains are represented by different colored rectangles.

Interactions of multi-modular signaling proteins with cognate ligands are often mediated by their constituent modules. Using hmmscan, a total of 3,573 sequences were annotated against the PfamA database, yielding 3,016 sequence annotations. Among these annotations, 90% corresponded to proteins containing only the PP2C core catalytic domain, while the remaining 10% were annotated with additional domains at either the C-terminal or N-terminal regions. A total of 119 distinct types of domains, excluding PP2C, were identified. Of these, 8 domain types with the highest number of annotations were selected for a detailed analysis of their distribution at the phylum level (Fig 5A). Sequences with zf-ribbon and DZR were predominantly found in Pseudomonadota, Cyanobacteriota, and Chloroflexota. Sequences featuring the Pkinase domain were primarily derived from Pseudomonadota, whereas sequences with the PASTA domain were mainly associated with Chloroflexota. In addition, the organizational structure of these eight domains in PP2C protein phosphatases was presented (Fig 5B). The results showed that other domains may be distributed in the C-terminal or N-terminal of the core catalytic domain, revealing the structural diversity of PP2C phosphatases.

## 4. Discussion

### 4.1. Evolutionary analysis and conservation of bacterial PP2C phosphatases

Since the characterization of the first eukaryotic serine/threonine kinase (pkn1) in *M. xanthum* [53], the regulatory roles of eukaryotic-type serine/threonine kinases (eSTKs) and phosphatases (eSTPs) in bacteria have been extensively studied [12,54]. So far, most eSTPs that have been characterized biochemically are PP2C-type serine/threonine phosphatases, which require metal ion binding for their dephosphorylation activity [37]. In this study, a comprehensive bioinformatics analysis was conducted to investigate the phylogeny, sequence conservation, and structural diversity of bacterial PP2C phosphatases. Initially, a phylogenetic analysis of 3,573 sequences was performed using the maximum likelihood method. This analysis yielded a phylogenetic tree that highlighted the common origin of bacterial PP2C phosphatases, suggesting that these genes may have descended from a shared ancestor and have conserved a critical biological function throughout evolution [33]. Specifically, the core PP2C domain, responsible for binding divalent metal ions (Mn^2+^ or Mg^2+^) and facilitating dephosphorylation, has been preserved. Furthermore, STKs, which interact with PP2C phosphatases in regulatory roles, also appear to share a common origin across bacteria, archaea, and eukaryotes [55]. In addition, the PP2C family was divided into five groups according to the evolutionary tree, and the number of sequences distributed relatively evenly among the categories, as illustrated in Fig 1. The species abundance distribution of PP2C phosphatases in bacteria was further examined, focusing on the distribution of different classes at the phylum level. The results revealed that PP2C phosphatases were widely distributed in bacteria, with particularly high representation in phyla such as Actinomycetota, Pseudomonadota, and Bacillota (Fig 2). Specifically, Groups I and II showed relatively high species abundance, which may indicate their widespread prevalence and functional importance in bacterial systems.

In Group IV, 62% of the sequences were of eukaryotic origin, and eukaryotic PP2C phosphatases were exclusively found in this group. This observation suggested that eukaryotic PP2C phosphatases may have evolved from a specific branch within the bacterial PP2C family. This supported the hypothesis that the PP2C family shared a common ancestor between bacteria and eukaryotes, with eukaryotic PP2C phosphatases potentially exhibiting characteristics similar to those found in Group IV. Previous studies have suggested that PP2C from the ancient *α, β, and γ-Proteobacteria* may be the ancestor of eukaryotic PP2Cs [33]. Consistent with this hypothesis, a portion of the PP2C sequences in Group IV identified in this study originated from Pseudomonadota, providing additional evidence supporting the evolutionary link between bacterial and eukaryotic PP2C phosphatases. It was observed that sequences from Pseudomonadota were distributed across all five groups, suggesting that the PP2C family may originate in Pseudomonadota. Additionally, the concentrated distribution of eukaryotic sequences in Group IV likely reflected the specialization and conservation of PP2C genes in eukaryotes throughout evolution [36]. Studies have found that eukaryotic PP2C phosphatases differentiated into various subtypes [36]. In mammals, there are at least 20 distinct subtypes of PPM phosphatases, all of which exhibit similar enzymatic activity, with only minor substrate preferences [56]. Studies have shown that PP2C phosphatases played key roles in regulating stress signal transduction and were involved in cell differentiation, growth, survival, apoptosis, and metabolism [57,58]. Due to their involvement in critical cellular processes, dysfunction of human PP2C phosphatases can contribute to the development of several diseases, including diabetes, cancer, infectious diseases, autoimmune disorders, and neurodegenerative diseases [36,59]. This suggests that, in contrast to bacterial PP2C phosphatases, eukaryotic PP2C phosphatases have evolved into more finely specialized subtypes in order to adapt to the complexity of their environment. However, current research indicated that in bacteria, there were few PP2C phosphatases responsible for dephosphorylation, typically only one or two [12,24]. This suggests that bacterial PP2C phosphatases may have relatively poor substrate specificity, allowing them to recognize a broader range of substrates.

Furthermore, a detailed analysis of the conserved motifs within PP2C phosphatase sequences was conducted. Previous studies have identified 11 conserved motifs in PP2C protein sequences, containing a set of conserved amino acids, including four Asp residues located within motifs 1, 2, 8, and 11 [51]. In this study, a sequential conservation analysis was performed on 3,573 PP2C sequences. Consistent with the results of previous studies, conserved Asp residues were found in Motifs 1, 2, 8, and 11. However, Fig 3A showed that conserved Asp residues were also found in Motif 5. Studies have demonstrated that a model of two sets of Asp (in Motifs 1 and 2; 8 and 11) appeared to be an ancestral trait for PP2C proteins, and Asp in Motif 5 were acquired later and appeared to be a more derived character [33,51]. Overall, Asp and Gly were among the most conserved amino acid residues in the PP2C core catalytic domain, and they were often positioned adjacent to each other. Numerous studies have identified Asp and Gly as key residues in the active center of PP2C phosphatases, where the precise repositioning of Gly was crucial for phosphatase activation [37]. In addition to this general conservation, notable differences in the conservation and diversity of sequences across different PP2C groups were identified, as illustrated in Fig 3B. Specifically, the sequence conservation in Groups IV and V was reduced compared to Groups I, II, and III. One significant finding was the shift from a “DN” sequence to a “DD” sequence in Motif 11, which may indicate that PP2C phosphatases in Groups IV and V have evolved distinct biological functions or enzymatic properties, potentially as adaptations to changing environmental conditions. These changes in Motif 11 can serve as markers of evolutionary differentiation and could be an important reason for the emergence of more subtypes of eukaryotic PP2C phosphatases. The conserved “DN” sequence found in Groups I, II, and III likely represented ancestral features of the PP2C family, while the “DD” sequence observed in Groups IV and V suggested significant divergence, reflecting evolutionary processes that have led to the specialization of these phosphatases.

### 4.2. Structural evolution and catalytic mechanisms of bacterial PP2C phosphatases

Research on human PP2C phosphatases has established that all PP2Cs shared a characteristic β-sandwich fold and a binuclear metal center (M1, M2) [60]. Consistent with these findings, structural comparisons revealed that bacterial PP2C phosphatases exhibited significant similarities to human PP2Cs, with the primary difference being that bacterial PP2Cs lacked the three C-terminal alpha-helices present in their human counterparts (Fig 4B). The missing three C-terminal alpha-helices may lead to the poorer substrate specificity of bacterial PP2C phosphatases. Additionally, structural studies on bacterial PP2C phosphatases have uncovered the presence of a third metal ion (M3) in the active sites, with Ser and Asp residues implicated in its coordination [34,61]. Interestingly, the crystal structure of *Pseudomonas aeruginosa* PP2C phosphatase (PppA) did not exhibit the third metal ion, leading to speculation that M3 in PppA may be less tightly bound compared to other PP2C family members, or that the flexible flap subdomain of PppA might contribute to the absence of M3 [62]. Even so, some studies suggested that the trimetallic catalytic center might be a conserved feature of bacterial PP2C phosphatases, with the third metal coordination site typically involving Ser/His/Asn residues [38]. Specifically, in the representative proteins of Groups I and II (SaSTP and PstP, respectively), the third metal ion was found to bind to Asp and Ser residues. Although a crystal structure has not yet been characterized for the representative protein in Group III, predicted structural models suggested a similar flap subdomain to those in Groups I and II. This leads to the hypothesis that bacterial PP2C phosphatases in Groups I, II, and III likely possessed a trimetallic catalytic center, whereas phosphatases in Groups IV and V, which resembled eukaryotic PP2C phosphatases more closely, may typically have a binuclear metallic catalytic center. The precise role of the third metal ion in bacterial PP2C phosphatases remains unclear. Some studies indicated that while the third metal ion may participate in substrate binding, it is not essential for catalysis [38]. However, there is evidence suggesting that the absence of M3 can impact dephosphorylation efficiency [63], implying that M3 may play a supportive, though not indispensable, role in the catalytic activity of bacterial PP2C phosphatases. In addition, a fourth metal ion was unexpectedly found in the recently reported PP2C phosphatase (STP) of *S. aureus* [40], which was different from the previously reported bacterial phosphatases. And they found that Glu18 was a key amino acid that binds the fourth metal ion, and an equivalent amino acid (Glu/Gln) was found in previously reported bacterial PP2C [40]. In this study, a conserved Glu or Gln residue within Motif 1 of the PP2C phosphatase core region was identified across bacterial Groups I, II, and III. However, these residues became progressively less conserved in Groups IV and V (Fig 3A). It is suggested that the presence of the fourth metal ion is relatively common among bacterial PP2C phosphatases in Groups I, II, and III. In course of evolution, in order to adapt to the changing environment, the bound metal ions were gradually lost, and eventually the eukaryotic PP2C phosphatases with typical binuclear metal centers were formed. Therefore, it is speculated that the binuclear metal center represents the fundamental catalytic element of all PP2C phosphatases. This can explain the functional conservation of all PP2C phosphatases, as they participate in the same regulatory pathways across different species, such as cell division, growth, and stress responses. The additional metal ions might be ancestral features that initially played a role in auxiliary catalysis or substrate recognition enhancement. This may be the key structure required for bacterial PP2C phosphatases to regulate life activities unique to bacteria. Although PP2C phosphatases were generally characterized as Mn^2+^- or Mg^2+^-dependent enzymes, studies have demonstrated a pronounced preference for Mn^2+^ over Mg^2+^ in activating their phosphatase activity [60]. This preference was consistent across both eukaryotic and bacterial PP2C phosphatases, with Mg^2+^ often exhibiting negligible stimulatory effects or even inhibitory effects in some cases [30,64]. Furthermore, this study revealed that the catalytic activity of bacterial PP2C phosphatases is mediated by the coordination of three metal ions with conserved Asp residues within motifs 2, 5, 8, and 11. These findings underscore the critical role of metal ion coordination in the functional mechanism of bacterial PP2C phosphatases and highlight the evolutionary conservation of this catalytic feature.

As shown in Fig 5A, proteins in Groups I, II, and III possessed a front epitaxial α-helix in addition to a pair of antiparallel helices flanking the β-sheet, known as the flap subdomain in PP2C phosphatase. The size of the flap subdomain in Groups I, II, and III was relatively consistent; whereas the structure and length of flap subdomain in Groups IV and V were more variable, which was in line with the characteristics observed in human PP2C phosphatases [60,64]. These findings suggested that the flap subdomain was a relatively conserved structure, with its conservation diminishing during later stages of evolution, paralleling the changes in the number of metal ions in the active center. The alterations in the flap subdomain may influence the binding of the third and fourth metal ions. Previous studies have demonstrated that the flap subdomain was a mobile element and its rearrangement was associated with the binding of a third metal ion [38,61]. The flap subdomain was more flexible than other structural elements and played a crucial role in substrate binding [12,64]. Furthermore, in a study of Stp1 inhibitors, it was observed that the flap subdomain shifted from a horizontal to an almost vertical position upon binding of the inhibitor epicatechin gallate (ECG) to Stp1, resulting in the allosteric inactivation of Stp1 [65]. These findings underscored the flap subdomain could be used as a potential target for bacterial PP2C phosphatase regulation. In conclusion, the trimetallic center and the flap domain are key structures for the dephosphorylation activity of bacterial PP2C phosphatases. Recently, Bradshaw and colleagues (2017), through biochemical and genetic analyses of the *B. subtilis* PP2C phosphatase SpoIIE, revealed that bacterial PP2C phosphatases may frequently utilize an α-helix switch mechanism that is activated by protein dimerization, which involved a pair of α-helices at the base of the enzyme. The carbonyl oxygen of glycine was then repositioned into the active site, facilitating the recruitment of metal cofactors that regulated phosphatase activity in response to various physiological processes [37]. Furthermore, in a study of Stp1 in *S. aureus*, Arg161 was identified as a critical bridging residue, connecting the flap subdomain and the catalytic domain to enhance substrate binding and promote dephosphorylation [66]. Our research identified conserved Arg residues within motif 5 of the PP2C core structure in Groups I and II, suggesting that this Arg may represent an evolutionarily ancient feature involved in bridging the two domains. This conserved arginine has gradually lost its conservation alongside the evolutionary modification of the flap subdomain.

### 4.3. Functional diversity and regulatory roles of bacterial PP2C phosphatases

According to the studies mentioned above, PP2C phosphatases are evolutionarily conserved proteins with a core dephosphorylation domain. However, previous research has demonstrated that different PP2C phosphatases perform distinct functions in bacteria [12,21]. The functional diversity of these phosphatases may be attributed to the presence of additional domains that determine substrate specificity [67]. Therefore, we analyzed the domain architecture of bacterial PP2C phosphatases. 3,573 sequences were annotated in the PfamA database. The analysis revealed that proteins containing only PP2C dephosphorylation domains accounted for 90% of the total, and the remaining 10% of the sequences had other domains annotated in their C-terminal or N-terminal regions. Numerous domain types were identified, with some being prevalent in specific bacterial groups, such as the zf-ribbon and Pkinase domains. Notably, nearly half of the candidate sequences contained the SpoIIE domain. SpoIIE, a transmembrane protein belonging to the PP2C family, is composed of three domains: ten N-terminal transmembrane segments, a central domain involved in SpoIIE oligomerization, and a C-terminal phosphatase domain [68]. Several studies have demonstrated that SpoIIE played a critical role in the formation of polar membranes during bacterial sporulation [68–70]. Consequently, a substantial portion of PP2C phosphatases may be implicated in bacterial cell division and spore formation processes. Interestingly, the PASTA domain was detected in some bacterial PP2C phosphatases in this study. Most characterized eSTKs were transmembrane proteins that contain PASTA repeat sequences, which were located in the extracellular signal-binding sensor domain [71]. Zucchini et al. (2018) discovered that the PASTA repeat sequence in the protein kinase StkP was associated with cell contraction and separation in *S. pneumoniae* [72]. For PP2C phosphatases containing the PASTA domain, it is hypothesized that they may also function as transmembrane proteins and play a regulatory role in coordination with eSTKs. A prominent example was PstP, the sole PP2C phosphatase in *M. tuberculosis*, which functions as a transmembrane protein harboring a putative PASTA domain [73,74]. While the precise role of the C-terminal PASTA domain in the catalytic activity of PstP remained unclear, accumulating evidences suggested that PstP collaborates with the PASTA kinases PknA and PknB, encoded within the same operon, to coordinately regulate critical cellular processes, including cell wall metabolism, cell division, and antibiotic tolerance [20,75,76]. This functional interplay highlighted the potential regulatory significance of the PASTA domain in bacterial PP2C phosphatases, particularly in mediating interactions with cognate kinases to modulate essential physiological activities. Additionally, we identified that some bacterial PP2C phosphatases contain the zf-ribbon domain, which was known to bind DNA motifs. Although there were few studies reporting DNA-binding PP2C phosphatases, a serine/threonine protein kinase YabT in *B. subtilis*, which was activated by DNA binding, influenced sporulation and DNA damage responses [77]. This suggests that certain PP2C phosphatases may also be activated by DNA and play a regulatory role; however, the specific regulatory processes and mechanisms require further investigation. Moreover, some PP2C phosphatases harbor kinase domains, indicating the potential existence of enzymes with both phosphorylation and dephosphorylation activities. In summary, while most bacterial PP2C phosphatases are characterized by their core dephosphorylation domains, the presence of additional domains in some phosphatases suggests a broader functional range.

Recent studies have demonstrated that deletion of PP2C phosphatase resulted in significant physiological abnormalities, prompting increased attention to the role of PP2C phosphatases in bacteria, particularly in some pathogenic species. It has been found that PP2C phosphatases were crucial regulators of various virulence factors and played a key role in controlling both virulence and drug resistance in many bacteria [26,28,78]. At present, PP2C phosphatases in pathogenic bacteria such as *S. pneumoniae*, *S. aureus*, *S. agalactiae*, and *M. tuberculosis* have been the most extensively studied. Our findings indicated that PhpP from *S. pneumoniae*, Stp1 from *S. aureus*, and SaStp from *S. agalactiae* belonged to Group I, while PstP from *M. tuberculosis* belonged to Group II. These results suggested that PP2C phosphatases involved in the regulation of bacterial virulence and drug resistance might be predominantly distributed in Groups I and II. Transcriptomic analysis of *S. aureus* revealed that many virulence factors and their regulatory factors (Agr, Sae) were significantly downregulated in *stp* mutants compared to the wild type, indicating that STP played a role in regulating the synthesis of these virulence factors [19]. Additionally, proteomic studies have shown that PP2C phosphatases were involved in the regulation of cell division, with cell division-related proteins such as DivIVA, MapZ, GpsB, MreC, MltG, GlmM, MacP, and Jag identified as potential substrates [79]. The regulation of cell wall synthesis by STPKs has been linked to the development of drug resistance in bacteria. In *S. suis*, Stk and Stp regulated cell division through post-translational modification of DivIVA and modulated the translocation of certain virulence factors, thereby enhancing pathogenicity by disrupting host interactions [18]. Several studies have targeted STP for the development of therapeutic agents. For example, in *S. aureus*, compounds such as aurintricarboxylic acid (ATA), verbascoside (VBS), and epicatechin gallate (ECG) have been found to inhibit Stp1 [25,40,65]. Among these, ATA was an effective anti-virulence compound that did not affect the growth of *S. aureus in vitro* but significantly reduced bacterial virulence in murine models [40]. It is worth noting that in recent years, some studies have found that serine/threonine kinases and phosphatases played important regulatory roles in bacterial DNA damage response. The protein serine/threonine kinase YabT, identified in *B. subtilis*, phosphorylated the DNA damage repair protein RecA, which was also implicated in spore formation under extreme conditions [77]. Although *Deinococcus radiodurans* lacks the classical LexA/RecA SOS response mechanism found in many bacteria, research has shown that its DNA damage response proteins, such as RqkA, could phosphorylate both DNA repair proteins (PprA, DrRecA) and cell division proteins (FtsZ, FtsA). This phosphorylation significantly enhanced the activity of these proteins and contributed to radiation resistance [80–82]. Serine/threonine kinases and phosphatases typically function in tandem to regulated bacterial physiological processes. Although experimental evidence for the role of PP2C phosphatase in DNA damage repair was limited, transcriptomic analyses indicated that pyrimidine synthesis was markedly impaired by the loss of STP, with only a minor impact observed from the loss of STK [19]. This suggests a critical involvement of STP in pyrimidine metabolism, implying that PP2C phosphatases may play an essential role in regulating DNA metabolism, though further experimental validation is necessary.

## 5. Conclusion and future perspectives

This study provided a comprehensive analysis of the evolutionary history, sequence conservation, and structural diversity of bacterial PP2C phosphatases. Our findings suggested that bacterial PP2C phosphatases may evolved from a common ancestor, and are categorized into 5 groups by phylogenetic analysis. Further, the eukaryotic partners may originate from one specific branch. Structural divergence and fusion of the other domains may result in the PP2C phosphatases with functional diversity. These data offer a theoretical foundation for further research into the functions and evolution of the PP2C family in bacteria and eukaryotes, even could inform drug development by targeting PP2C phosphatases in pathogenic bacteria. However, unlike cognate protein kinases, research on bacterial PP2C phosphatases is still very limited. Although the existing literature also suggests that PP2C phosphatases play important role in phosphorylation signaling system in bacteria, further work is still needed to identify the target proteins of PP2C phosphatases and the role of specific domains in PP2C phosphatases, which are crucial for understanding their functions in various cellular processes, such as stress responses, metabolism, and pathogenicity.

## Supporting information

S1 File List of bacterial PP2C phosphatase sequences used to construct the phylogenetic tree. (DOCX)

S2 Fig Sequence logos for conserved motifs in five groups. The height of different amino acids represents repeatability. The scale bar at the bottom indicates the length of the motif protein sequence.
(PNG)

S3 Fig The primary to quaternary protein structure information of five representative proteins. Structural sequence alignment of five representative proteins. The sequences covered by the red short lines form β-strands, while the sequences covered by the blue short lines form α-helices. Additionally, the α-helices corresponding to the flap subdomain are also marked in blue.
(PNG)
