# Supplementary material for: Evolution and classification of Ser/Thr phosphatase PP2C family in bacteria: Sequence conservation, structures, domain distribution
Source: PLoS One. 2025 May 19;20(5):e0322880. doi: 10.1371/journal.pone.0322880 (PMC12088040; doi:10.1371/journal.pone.0322880)
Supplement: S1 File — (DOCX) [file pone.0322880.s001.docx]

**S1 file. List of bacterial PP2C phosphatase sequences used to construct the phylogenetic tree.**

>tr|A0A9E1AQF4|OS=Clostridiales bacterium

MKKQDILSYTLKELEDYMTEHEVPKFRAKQIFRWLHCDQVDSFEKMTNISAKFREELAESFYITSLKIKKRLVSDIDNTVKYLYVLSDGSCMESVLMRYHYGNSLCISTQVGCKMGCQFCASTIAGFVRNLSPSEILQQIYTAQKDTGEKISNVVLMGIGEPLDNFDNVLRFLELVSAPEGYNMSLRHITLSTCGLVDKINLLAQKRLGLTLTVSLHAPNDRIRSASMPVNQRWNMEQLLDSCRNYAKVTGRRVSFEYALIQGVNDKEEHALELAQKLKGMLCHVNLIPINEVSERRDKYRSSKPQNVTKFQNILMQQGINTTVRRTLGADINAACGQLRREAVKKEAGSLVKIAGITDIGLSRDNNQDTFEYGLLDDQTYYALVCDGMGGENGGDIASKMASDIITERLKAGYRSDMDANSIRNLLLTAIGAANTEIYAKAKQQPELAGMGTTAVVIILNEKEAYISHVGDSRVYLFRNHELTQMTTDHSIVQELIAQGKLSESDADDYPHKNLLTRAVGAYKQLSVDYMEQPLNNGDKLLLCTDGLTNFCPADILTRILQDSDIDSACKKLVNAACNAGGLDNITAIIAAV

>tr|Q5AME8|OS=Candida albicans (strain SC5314 / ATCC MYA-2876)

MLSSARKILLQSPKQLTTLIRPTNSFIIKRRNYSGSSIFKGARNYFLGFRNNSEDSQINNNNNNNNNNSSMHYDSVDESIPYVKYPLFTKEQLKKIATDKFKLQVGHASFGYHSDSIVPSINSLSDLTDPTSLNSLLPRRRPQGSPSDTLSIKAGDDTMLVSPSVIAVADGVSGWESDGITSSSGIWSRSMVETFSRLMTEYKLNHFPHYLNQRDIQEILDDSYLHTSHLMDLQKLNGSSTLILGMLNGDLLSIVSIGDSKIFIIRDGEIVLTNEEQTKAGLCPEQIGTHTLDHLPSDIAWIKSFKLMEGDYILMCSDGISDNLYEWEILNYLNEWINTKRNNNNNNNVKNIASKLLIKAKEVAFDDYAYTPYNEKVNSLNNTNGGSNGTNHSQGGKVDDMSVIIAKVELNKKDNTKLVIN

>tr|A0A317Y4H8|OS=Zea mays

MLAGYGGGGRSVHLSSHKDLLLGRCGRSFLFGNTWFLLSTYPARLLHTTDRRAPSAFVAAINRVPCVRSHCTGQGLLQRGSIVMAACGYVLRRTELGAAKRQQPDKDPSGGTRASRIAAMGSLGSAARPEVSFRYRGLEHCKKIGASLKCREPWGNSAFWTNATGPGWKLSFTVEPWTKDFSTACAAPYSAGATEDQLPLNEKMNSSTVGMSPVSEKLKLLSGSCYLPHPAKEATGGEDAHFISIDEHVIGVADGVGGWADLGVDAGLYAKELMRNSMSAIKDEPEGTIDPTRVLEKAYISTKARGSSTACIITLKDQGIHAVNLGDSGFVVVRDGRTVLRSPSQQHDFNFTYQLESGGGSDLPSSAQVFHFPVAPGDVIVAGTDGLFDNLYNNEISGVIVEALRVGLEPQIAAQKIAALARQRATDKNRQSPFASAAQEAGYRYYGGKLDDITVVVVEVSTSKTGKHGHAKCHFVAIDIFNGKKLEDIVPSSHNCDIPHVNRTEYQLIDISEDGFVSLLTSDGNTKDDLRLPTDETLVAQAYLLAMLALVIVLFYFTIFAFIVTHRGYQELGQD

>sp|B4NBL6|OS=Drosophila willistoni

MLSSLAGKLQGKTKFLIDVLKFYDARMLKSSSMSGIGKRGDPYLVKVVQGRSRKHSIASAKDNHRYGEDSWFISSTPKAEVMGVADGVGGWSELGIDSGLFASELMFWCANYAKRESFDGRTPLDLLIESYSEIKGKTDPIVGSSTACLVSLNRRDCTMHSANLGDSGFLVIRNGRMLHRSEEQVHDFNAPYQLTVVPNERFDNVYCDRPELADSTRLPLQEGDLVLLATDGLFDNVPESLIVKTLGKYQGVTREEDLQSAANSLVHMAKDLSISPNFESPFALKAKAFEVDYPGGGKPDDITVILATVAVPQND

>tr|A0A016S819|OS=Ancylostoma ceylanicum

MQKGPSTVLDSGVYGDDACFISRFKNTFVVGVADGVGGWRKYGIDPSQFSRKLMRECEKRVNSGDFDPKRPETLLEKAFKATAESPRPVGSSTACVLVVHQEMLYSANLGDSGFMVVRDGRVLTKSEEQVHYFNAPFQLTLPPEGYRGFIGDTPDLADKSEIRVQKGDIVLLATDGLWDNLTEQHVLEQLRPLIEEKATVQEVCNALALTARRLSFDVTHNSPFAVKARENGLSAAGGKPDDITLVLLLIA

>tr|A0A017T4Z7|OS=Chondromyces apiculatus DSM 436

MPGHAHTTHLKIEFAQASDPGRDPNKQVNEDSCGYQVCRLGHLAVLCDGMGGHQGGREASRTAITTIFEQVEQPAPGTSPAMVLKAAIEEAGRRVYQLGGAPESRGRPGSTVVAMLLHDRGVDVAHVGDSRAFVIRAGQIYPLTRDHSMVQGLLDAGMITEQQAIGHPDANKITRALGMKPEVEVEVRPEPMELYPGDILLMSSDGLTDLALNNDILGCVRQALSSGSLDHACQMLVQMANNRGGHDNITVQMARIVETGSRPMTLPGEPADTVVAAPPSMQGDAVAKTHTGATSHAPPGMTAPEPTAPWGPVLPTEPGALRPTAVDASLAAAHLHAGTPHAAAPHAAAPHAAAPHAAAPHFGASSHASAPAPFLPAQPTITEHERHTSAMGAPPGAPMGHHPAHLDAAPLPYPAPHQNHIAPYPHPTSPHAPAMSRISYGDANAKPSRGGLVFLIISISAVIAVLLILLLWVLLPG

>tr|A0A024TDK6|OS=Aphanomyces invadans

MVQVLQRSTSLMKMLRGGNYPRDRQPLTPGTPQQQLPALGNKVVRTPILVEEKHVHPTTPRPEPSSGSNKLTRMWQNIRRRGRDESAPRHGSASFVLHGEDAMAVGPLYHIVADGISTTTHHSTSAAPSLSPSPSAVLARALVHAVEHVLLHDPPPSSLADFEHMVIRGILTAQALCQHVSSTMGSTLVVALVHAKHLFTFSIGDSKCLVLRKGRIVYETLAVMKEFNVPWTVTHHALRPHMYVVQRIPLKKLDIVLSFSDGFGDNVYKDDLVNLLHVTATPSDKCHRLLQHARALNDVSKETAFPFSAAAAAAYVARAKEDESSEVRKLTFSDI

>tr|A0A024UU57|OS=Aphanomyces invadans

MGLDGYKYMISKMKASRGNQSRLGSFSSIGCASYDFHGDDAVGYGPNYMVVADGVSGTQKASGILARVLVTETLNSLERLRRKSLSDPAVPIKSSDFGAEMIGAIRVARALTRRKGRFDSALTAVYVDAASSQLFVFNIGDCKCVLVRHNVVVFESDAIIYDFNVPAVVSTMNEIHYPTDAVDIQVTGYEPGDVVMVFSDGVHDNLYVDEVLHVVASHPNHGADIAKATVRACRDTFTGHCGYIPFAVAAAGFCLTAVEEMKTNDAITRADFEAFEAKCRDLPRPGTRAIFGNDKRVKKLAFYSASNLLTFANKQIGKKDDISVCAGVLA

>tr|A0A024UUZ2|OS=Aphanomyces invadans

MGLDGYKYMISKMKASRGNQSRLGSFSSIGCASYDFHGDDAVGYGPNYMVVADGVSGTQKASGILARVLVTETLNSLERLRRKSLSDPAVPIKSSDFGAEMIGAIRVARALTRRKGRFDSALTAVYVDAASSQLFVFNIGDCKCVLVRHNVVVFESDAIIYDFNVPAVVSTMNEIHYPTDAVDIQVTGYEPGDVVMVFSDGVHDNLYVDEVLHVVASHPNHGADIAKATVRACRDTFTGHYQRCDHARRFRGV

>tr|A0A060TDL0|OS=Blastobotrys adeninivorans

MQTDSKMISRRGFTRFHQTLSSVKRTFRTTAITDRASKNSQIKITSTTSSSSTDSSTTTTATNSGPTSTLSSSNSIDPIANIQLTTDEPINDSTHSGDRLSFQMAEAYLPKMQDNAISLPEEARKRVSRDVNSKLTDRPDSGQDSFFHVQIDKGTKRGSIALGVADGVGGWATVGVDPSEYSHTLCELMVERFLEQRDLPHNEPLVQPLKLIDHAYEKIKKEDLVQAGSCTACVGVASPLSGILQVANLGDSGYAIFRQGRVYRLSTPLTHRFNTPYQLAAIPNWIKFENVRRLDDKPSDAIISTHTLQHGDVVVFSTDGLTDNLFAHEILNIVNDTMIKTSSWVTGDDGEIKPGKTMSGSADLAFALVKNAALRSVDLDRTTPFAVELRREIGITATGGKPDDITVVCLLVQDAQSEQDDS

>tr|A0A061AW70|OS=Cyberlindnera fabianii

MPCPNWDESVAGPGAVAAGNKKKSCHPFTYIYIYPQRFSLSRKTYLIHTRGYQAVMSAVPKSTARSGMFSNLVGLVTGRSKSSPIVPDPRGPSQSYLQNKTTINGSSNLTPRSVPKQFATPDHYKFLYSFASYVHHGSIRKPVVNSLMDLMDSTSHLSLLPRRRLYGNPYETLSVKNGDDAMIVSPNLLGVADGVSGWSGAHADSGLFARSFLENISRNFAELSHKNKEHLNRISNEQLKESLDLSYNESLQVMERENFKGSSTVIIAMIIDKKLKVLNIGDSRLFILRDGEILATNKEQYISNMCPEQVGTTAEEKLPSSVAQISDYELETGDLLLLCSDGVTDNLYQDEILDFVNTQLNADKTNLGEVCHSLMVKVKSVAFDNYVVTPYVEKVNELNTDFITGGKLDDISVCLARVAENN

>tr|A0A078K375|OS=Brassica napus

AVTPEEPVAVSPDELISTSEATNHSVDETAETPVIDTSEVANDEENVASTIEGEVVVIDSKNDNGSISKTVNDTNDEDLQLPEPETAGLQPIEVASDREELVSKSFYLESGSASLQNPNKALAGREDAYFISDNNNWLGVADGVSQWSFEGISEGMYAQELMSNCEKIISDETAEICDDPVHVLHRSVNETKSSGSSTALIAHLSNNELHIANIGDSGFMVVRNRTVLQKSSPMFHHFCFPLHITRGDDILKLAEVYHVNLEEGDVVITASDGLFDNLYEKEIVSIVCRLLEQGLEPQRIAELVAAKAQEVGRSETERTPFGDAAKEEGHDGYRGGKLDAVTVIVSLVKTVSI

>tr|A0A085LTE4|OS=Trichuris suis

MRNTTSFLLASRVVRFGLFRFNAYRLFVVGLHERYPEETDVLGFISAKSGFAKYPQSEKRKNRFGDDACFIAHHYSADVLGVADGVGGWRDHGVDPSRFSYSMMNACAQLVAEGLFRPYELRELIADSYEVVKNATSFHNENGSSEAARCTDAIIGSSTVCIVALERRVSQIHTANLGDSGVLLFRDGELLWQSAVQQHEANVPFQLALIPEDQYPNSIADQPDAADINSIDVELGDCVVIGTDGLFDNMPVELIEEEVASLRSCMYTDVKRLCDGIAQKAKTLSQKARFPLSFARKFGRQGGKPDDITVIAAVVARVGREGVDAV

>tr|A0A093V061|OS=Talaromyces marneffei PM1

MAQYFFDLLYTFTDCMCCFPSSPQLKINNRSFKLLRLLGEGGFSYVYLVQDKSTSELFALKKIRCPFGQESVSQALKEVEAYSLFTPNPYIIQSYDHCVVNESANKFRGGDDSSSKTVYILLPYYQRGNLQDAINANLVNHTSFPEKELMTLMLGVAKALKAMHQYRVKSGSASTRQARGVRIEGEEADEELSRKVGKPKRRNTHGMDEDVEQEPLMDDEVTRSQEGVGEGEFRPYAHRDIKPGNIMIDDDGRTPILMDLGSLAPSPIAITSRSLALAVQDTAAEHSTMPYRAPELFDVKTGSIIDTKVDIWSLGCTLYACLVGKSPFEARSEETGGSLSMCVLGGDWRFPDENASSAKGKAKSNTPAASGSSQSSGEGGISESVKDIVRKCLQVEPAERPDIDELIQLIQDAIKVIIVYTVVTGWKKERILHIRANSAIGAHFVATSEDVASYKVLGSAGRLHGAANPYHALRRTPRSLRLSRKFSSSTSSQSSPSSRISYRVAGSCSAKGRRFNPEKHTYNFEPTIHEAIGVSLDNIGEGRSRQKRPASGEDAFFASRVGAVDTGAIAFAVADGVGGWAEHKIDPADVSHGLCTYMAQHALTEEASQRKLRPKELLQKGYDSVVADESITAGGTTASVGVALTSGTVELANLGDSGSVLFRLGAVHQYSAPQTHAFNTPYQLNIIPRRMREQAHMFGGVYFEDSPRDAAVSTLSMQHGDVLVLATDGVFDNLNNQDILKIVTGRMLATGAWKESSKNAAICPSEELQALTQPGGLHRQTPSSPSSSPSSTSIPPTPHHKSHHTLQALLAASIVGEAKLASVDMRRDGPFAKEAQRYYPGHWYRGGKVDDICALVIVAIEDNIDPRVASELLYTGSSPAWTCVSDPRFCYFLYIPRNYYTLPATPQSLNLIVLIHGSGRNPYTLRREFSTFAETHSCALLAPLFPVGLIDPQDTANYKYITYKDVRYDQVLLSMIDEAAARFRRIDTSAFCLYGYSGGGQFVHRFAYLHPRRVRALACGAPGTQTFLNFSQRFPDGVQDWEEVFGAPLDIKAVMGIPSMFVAGDQDTDIFYAIARGRIREDQKDDEVAMAKFKDGRYGATNRLCENWVQVGVRSCVMVSVEGVKHEERPMLESVKGFFEMFVTAATTTSSEDRAKV

>tr|A0A094DBC4|OS=Pseudogymnoascus sp. VKM F-4516

MDIAARRISANTLATTASKRLALAECVIPTLCAQQPTGRRSLTNSSLAKSRTKTPSRRQYSLPTPIAKRQESSAAHPPPRFSYGIAASFTAKDKRFKPNENVVHFEPHPIPVKRRTNRKARPASGQDAFFVSQLGDSGDVAMGVADGVGGWADSGVDPADFSHAFCDYMAYEANNFDPLSGEALSAMALMQEGYDSVVEDKTIRAGGSTACVAIARTDGSLDVANLGDSGFLQLRLNAVHYNSEPQTHAFNTPYQLAIIPRAMRMMTQAFGGTQLDDMPKDSAVSKHNLRHGDVLVFATDGVWDNLNSHDILKLVSKLMVGANAWTHSDEGIKVTDRLSEYIVKDTSGGPDKSVKSLQSFLAMGIASAAKAASINRRVDGPFAKEVQKHYPHEMWSGGKVDDICVIVAVVVEDGK

>tr|A0A0A1D5B1|OS=Arthrobacter sp. PAMC 25486

MSGDAGPADGRPSGVQLAVGAGTDRGLRRELNEDDFLATDPLFAVADGMGGHEAGEVASQECIRTLGQQPFLTGGSREATASDLQMALRRADARIRELTNARAGTTVSGVVLVEERAVPYWLVFNVGDSRTYCLSQGTLRQISVDHSEVQELVDGGFITAEQALVHPRRHVVTRALGAGSEIDADFWLVPVKEGDRILVCSDGLTGELSDSQLHGILSTLHKPQDAVDALIQAALRAGGRDNITAIVLDASNIGGPAGTDTLDKTEPRHHDDGDTLPRAPHPAAAAATQPTTQPAAQPAAQPAAVETATTKPPAGDTAVQDHAGTPLHEENHG

>tr|A0A0A1TI93|OS=[Torrubiella] hemipterigena

MVEIVILGREGDSLFVDSRKKKGIRGLIKQPVLSLLYSIDTEHSEDPFLVPIPDTKRSKKKKEPFAPSNCTYMATSSRRLVASTPWFTAITAGGRNCSRRTGALAMRPQQNSYSTTTTTSTSSPKFSYNIAASFIAKDRPYDPSTHVFHFNPYNRIQPPRNRKRSSRPDSGHDAFFVSRVNDSGAVAFGVADGVGGWVDSGVDPADFSHGFCDYMAAAAYEHKGSTALTARSLMQAGYDAVCNDKSLSAGGSTACVAVASPDGVLDVANLGDSGFIQMRLNAVNTYSEPQTHAFNTPYQLSIVPPSVAARMAAFGGTQLSDLPRDADVTHHNLRHGDILMFATDGVFDNLFHHDILRIVSRVMTSAKAWEVAKSGSVNVGSNLETIVKSPLETKLSHSVTLQSLLATELVLAAKRASVNTKMDGPFAKEVQKYYPHENWHGGKVDDICVVVAVVCEEPAAAASAPLKSKL

>tr|A0A0A9FE58|OS=Arundo donax

MQNEVDGRNGLAKGELELCADGDDVEEATEMSGVLCHEGLEGMETSLEECEASDGSTVHVEEGVDRMETSLEDSEASDGLTTQDSDTDVETESSGSSTEEQDAEYGAHIPQMDQAIRKVARENNISEVNSSDRMTSASVSRLVLASGAAMLPHPSKVLTGGEDAYFIACDGWFGVADGVGQWSFEGINSGLYARELMDCCKKFVENQGAPGVRTEEVLAKAADESRSPGSSTVLVAHFDGKVLHASNIGDSGFLVIRNGEIYKKSNPMTYGFNFPLQIEKGDDPLKLVQKYAIELQEGDVIVTATDGLFDNVYEEEVAAIVSKSLEADLKPTEISEFLAARAKEVGRSGFGSSPFSDAALSAGYLGYSGGKLDDVTVVVSIVRKPEV

>tr|A0A0B7FLU3|OS=Thanatephorus cucumeris (strain AG1-IB / isolate 7/3/14)

MMNHHIKTAVAKRARSLYTGSALPQTTLPNVVFDYAHSPRPSPRKTPYQHPSGQSQSEQPSGSPATTLPSPYYTSQTAWIGNPGQTPSYAIPPPLWLCPPLDAAWFPLALAIDVGPGPWDLIRVRKEPEPAPAEPEVEPVPWSFQCGAYGIPKKGKRKAESDDLQMAVQVGEDSYFLRPDALGVADGVGGWAHHHLRADSARFARMLMHNCASEIANPCRPQDAYPSPPLTPRSPSTTDDISHLASVLESVSLEPEISPREVLHLAYERTVATFRATGTAGSSTALVALLRDGELSVAHLGDCMLAVVRDGKFVLRSEDMQHSFNFPYQLGPHSSTTPRADAHLIKSKVVPGDIVILASDGLGDNLWDEEVLSEISRFQLSQIVSGEGDVNPQVLGEALARRAKKAAEGKADVPFGTRARAAGVQFGGGKMDDISVVVAIVRGPTPSTPS

>tr|A0A0C2IS41|OS=Sporothrix brasiliensis 5110

MFDATNHNDIDHHGSPARRRCVGLFAKRIPRPFPPPFLSPPSGSFSDPLSTHDRSRDRRELVDGHLIGGCTNGDDAVYAGEYFVAANDGVGAWSMRPRGHAGLWSRLVVHFWANAVSESVAAGSITAPEPIKYLQRAYEQTIEATRAPNDWQGTTTAAGALLSYRQIEAADDRAAYTDENEPVLYVTNLGDSQIMVVRPAEGKIVFKTTEQWHWFDCPRQLGTNSPDTPRDNAVVDVVPINVGDIVLAMSDGLIDNLWSHEIVEQVSKSVAAWQAKDKTPADLHRGMMTAVAEELVEAARVIAVDPFAESPFMEHAIEEGLASEGGKLDDISVVAALCRRSDAT

>tr|A0A0C3KBV8|OS=Tulasnella calospora MUT 4182

MKPGAHLKLLPRSTTQAARRKLSTATAYTTQSSPLYFDVLPQTSQASGSSSSHSGGPSSSPRILKGPSQPTTALTPYSPPPTHSGTSSSAIAHPRYTTNSSHAYPGGNGLPLVWFVPTSTNSGRSEIERRLPWEGQSEGKGKERRTFSLEMGAYGIPKRRKNQQSEEHNGSELHQQQQQQHVHLSASAPTSSSLSTGAGAPKEDPAAYSLSVQVGEDAYFLRPDALGVADGVGGWKHKGESNSALFAKKLMHFCSVELARQQQQDHYPTPPPSSASTPNTSTANSSPPPASGPDPVQILQTSYERCIQDARQSGMLGSSTATLAVLAESELRIAHVGDCVVCVVRDGEMVFRSEEMQHSFNYPYQLGPRSDTTPATHAQRLTVPVKANDIVILASDGMGDNLWDEDVLDEINRFIRTSPLSSSPSQGGGGSLSFSASSTSSSTPPSSSNLRNPHAPAGPVTNTVLAQRLSEALASRAKRVSERRAMTADASGTMGAFAQRRPTIPPPPKGPPPPPPPRSARPASTSAPMPIPPRAAAAAAAAPAASSHPTQTPTPPPSPLLKPIPVVEGMELPIHKSITQSDGQLDDDVLGDGFGGDGEYGGAGLGGLGWGVEEEEEPEWVRDEIPFGRRAREAGVRFVGGKGDDISVLVAIISSVPEPTAEPPQPAPAYQHMRSSSSSAIDALAKFRLHSTL

>tr|A0A0C9TUP4|OS=Sphaerobolus stellatus (strain SS14)

MSSSTVSKRLCAVIPRQTRALSLYSHHSSGGPTLSPDYAPSPVVRPSPPNPSSTARQSVGPISTSTHTGANGNAGSGNANSGGPTPGPSTPTAQHTPQSSQNPPPAPLQLALTPLAGRGQSANASSTRDTQSLALFPSISFFPSSFPFLNPSGLYQPPEPAKRSRLTYHLSTGAYGIPKRRAHTVAQPPQSLHPDLNLAVQVGEDAYFLRGDAMGVADGVGGWSKVCKSPGEANSALFAKSLMHWCSYELALSSQSAPPPIHIPSIPVRPDPPVSSPQLHPSPHPLDILQRSYDRCLASFSSAGISGGSSTALIATLHAETLRIAHLGDCAICVIRADKLVYRSEEMQHAFNYPLQLGPKSPTLPRDARVVEVPVQEGDILVVCSDGMTDNLWDEDVLDEVAKFTRGGKDGGSGGKRAYENKWLPGMLSQALCSRAKSVSESRGCSTTSKSASTSIPRSQGVSSPILSAAAASASAHPETQAEEDAEQYRDEVPFARKAREEGIKFVGGKCDGVFHLHFPYCVEIEADFCVGCRYLGVGSDYIAVFGAGADGVLRCLSLPSLVLSSSCSSTLIHIAPYHLTFGCLILFRHTTYTPPHSPPVIQYSDWTGRDLDWPAPKSDADADANQSWSWTHVRYPTPDSALPHLHRLIPPSPSHPQLNRIPLILARPLPVHRSPTHLHHNLRHHPIQCRHHHHSSRLRKPNPKTADLANEA

>tr|A0A0C9VJJ2|OS=Sphaerobolus stellatus (strain SS14)

MGVADGVGGWSKVCKSPGEANSALFAKSLMHWCSYELALSSQSAPPPIHIPSIPVRPDPPVSSPQLHPSPHPLDILQRSYDRCLASFSSAGISGGSSTALIATLHAETLRIAHLGDCAICVIRADKLVYRSEEMQHAFNYPLQLGPKSPTLPRDARVVEVPVQEGDILVVCSDGMTDNLWDEDVLDEVAKFTRGGKDAGNGGKRAYENKWLPGMLSQALCSRAKSVSESRGCSTTSKSASTSIPRPQGVSSPILSAAAASASAHPETQAEEDAEQYRDEVPFARKAREEGIKFVGGKCDGVFRFHFPYCVEIEADFCVGCRYLGVGSDYIAVFGAGADGVLRCLSLPSLVLSSSCSSTLIHIAPYHLTFGCLILFRHTTYTPPHSPPVIQYSDWTGRDLDWPAPKSDADADANQSWSWTHVRYPTPDSALPHLHRLIPPSPSHPQLNRIPLILARPLPVHRSPTHLHHNLRHHPIQCRHHHHSSRLRKPNPKTADLANEA

>tr|A0A0D2B8R1|OS=Verruconis gallopava

MQTVRLRLQIGLLQQRQSTCAYRLASTSYLSIFQARHCLRHRSNSTQRRWQSVQAQSQNEIADAEGNVQLESSGTSTPAPDTSQKKYAKTPFYFEAGYALFAKRRSRPFPPPFVSMPSGSFSDPLSTHDRSRDRRGQPTVNGEIIRGITNGDDAVLVSENFICANDGVGAWAQKEKGHAALWSRLIIHFWALEAEKDEYGGDHEPNPVAYLQRAFESTKQATSHPSEWYGTTTASSALLSDDHKNPPHPVIYATQLGDSQIMVLRPRDREIIYKSKEQWHWFDCPRQLGTNSPDTPEKNAVMDRVEIEEDDVVLAMSDGVIDNLWDHEVLQNVVDSMHRWENGEVPLENEGREPAEQSYSEEMMYVAEELVKAAKAIAQDPFAESPYMEKAIEEGLSIEGGKMDDISVVAGQCKRRKT

>tr|A0A0D2BWE5|OS=Exophiala xenobiotica

MTSAAPIRRPAFLTRSLIFTPSYINHPLRPHIRRGLDPYKPHARSLHSSPRRVAATADPTAKTASSAIPHISLHIAASSSGKGRKFRPESSTFDYIPSNTDGLGLQQGSTIEEKRSRRPDSGQDAYFVARVGQDSDTTAFAVADGVGGWTEHGIDPADFSHGLCSHMVETALSWSQEERLGPRQLLEIGYDKVKNDPAIRAGGTTACVAVSEPDGRMRIANLGDSGFLQLRLGTVHHYSNPQTHAFNTPYQLSMTPPEILAQAMIFGGMPLADEPEKADLADHMLRHGDVLVLGTDGVWDNLNSQDILSIVSNQMRTLGAWLRSPDQGYSISPILPELVDRSLGPKKHKLPGTLQSVLAAAIVGEAKAASLSAKRDGPFAKEMQKHFPYDPWHGGKVDDIAVLVVIPVDQARAREDKASLKPKL

>tr|A0A0D2HLM2|OS=Dethiosulfatarculus sandiegensis

MEITFGGSLDVGLVRTENQDAVFLPPEDQGPVPGLFVLADGMGGVAGGRVASELATNTIPRVFATESRENGLERGLTAAISAAAEAVFQKSQETPDFRGMGTTVVAVALEPGRAVVANVGDSRCYLWRKGELTLVTHDHSVVMEMVDQGQITLEQAREHPMRNVLTRVVGNQPVVEVDLFSVELMPDDQLLLCSDGLHGPVSEELISAVIGAENGPRGKTRVLVEEANRAGGPDNISAVLINIQDPGEMPEEARAASFLQLPFSRKTLALGLAAVLALGLLVFAFFFIPPKKQDNSHTPVIHGKVDAKELDSAKPLQTKPPAKAKPSSELVVPGAAVKANEAGQTEDPLASQVKSVEKQEFSEEPTADPPPKKLTEKDSGHKEPQNAPEPKETSPGLSPEEGENNKGLEALSSLAPELQKDPMLVDHIWKDGGGSQNWRQIGAYAKTANLKKDLEIVRKVLAGDKRVKLVWTIAPAKGKSPMLYRLLLGNSKKKGLKKSLSALGRKGVKPLDPPNLLAGGGRYSFVLSRVKDLKQAKPASSPELNLVFLAGGMADQAQIRTVTDIVKSGYPGGLLLMDYGAGREKTLSLALMAADILGHKRVFLYRSKRKTRSKDSQLLVPRKMVRDFCMNVTAALPVISDLPLTRRASLIMIKGVN

>tr|A0A0D2IYK6|OS=Rhinocladiella mackenziei CBS 650.93

MALPNQLYVDHAYEEYLCRPCDRRFSTLNGALNHCQNAAVHRGEWCTRCERLFVSPAARNAHVANSSRHHICDRCDLDFPTFRQHRGHDISVHHLCTECGQEFSNDNNLQQFSLLLTYTTVRSNPLRQGPALTDRVFLELPRSIRRSLDLDKPHARTLHCSPRSAAAAASSTSAIPHISLHIAASSSGKGRKYHPELSTFDYYPSNTDGLGLQHGSTIEEKRSHRPDSGQDAYFVARVGQDSDITAFAIADGVGGWTEHGVDPADFSHGLCSYMAETALSWSRDERLGPKQLLEMGYEKIISDPAIRAGGTTACVAVTQADGRMRVANLGDSGFLQLRLGTVHHYSNPQTHAFNTPYQMSLTPPEILAQAMVFGGMPLNDKPDRADLADHMLRHGDVLVLATDGVWDNLNSQDVLSIVSKRMRMTGAWLRSPDQGYTISPVLSELVDKSIGLQKHKMPGTLQSVLAAAIVGEAKTASLSAKRDSPFAKEMQKQFPFDPWHGGKVDDIAVLVVIPVNKGSTEKEGDGDRIKAKL

>tr|A0A0D3A282|OS=Brassica oleracea var. oleracea

NDVQALTGREDAYFISDNNWLGIADGVSQWSFEGISEGEYAQELMSNCQKIISAETAEICDDPVHVLHRSVNETKSSGSSTALIAHLSNNELQIANIGDSGFMVVRNRTVLQKSSPIFHHFCFPLHITRGDDVLKLAEVYHVNLEVGDVVITATDGLFDNLYEKEIVSIVCRLLEQGLEPQQRIAELVAAKAQEVGRSETERTPFADAAKEEGHDGYRGGKLDAVTVIVSLVKTVSI

>tr|A0A0D3FQH4|OS=Oryza barthii

MPRMEGEGDEEEMLGGGLIEELRREKSAKKRLDMDEGEEGGGEIAMDAEVKGKRSKRRKKDEAPKDSARGKKRSEKERRVQLDSIHAESQRLLRETRSVSFKPSAQPVYKPISSVLEKIRLRKLEILKKSVSLLFLSATPNDDDDEEEDDVSSDPVSGTAGDLGAPQVKEVDAEGKDPKIDDIENEGGMNSGDVNQCDSVPENKDALNCDKDLDNCGSKDLDKELLENSQDNLEDKAQSSDNPNNAADEIQSPPSSSPTESTDDISSEDEEYNDKENIAPSTPKDDVNVHEPLQRALAGDSCPDDAILKDFLDVEAEEEDESDNDMMRFKDNEEDDGSDENEVFNDLIEAGYEEGEIDHEKRNALHQKWLQQQDAAETNKFMQKLKFGHQEQKKVMDQDEDDAEDCEDESENEMSYDLTPTNVVRQNSEKAKQMIARMFTDDNDTYEHSDDEEIEEHLARQRISKREVHNSSFISPLEDDSSREVFSLIKKLNIAPQPKRRGKQVTSNHELLTVGRNNSASSKSSFLGRTASSSLASSHRSAYRTYVFGRDDSNSSNKSCLSTSESNADTDQTNSSQPKKAKFSSSQSKQAATKTNSKGDNSSGVSLIEVLRRSSSTSDKQEYTRQESCAVITESQAAHQFSAFKLSRRFSRSLLQRGIVMAACGYAFRRAELGAAKRQPEKDSSVGTRISRVVAMGSAGSTPRPEVSFRHRGVEYCKKVGVSLKCREPWGPSRAFWTNAIGPSYKLSFSVEPWLRDFSTSCVAPYSAGATEHQLSLDEAVQDKQMDNSTVGPDGKPRAPGPLKLVSGSCYLPHPAKEATGGEDGHFICVDEQAIGVADGVGGWADHGVDAGLYAKELMSNSMSAIKDEPQGTIDPSRVLEKAYTCTKARGSSTACIVALKEQGIHAVNLGDSGFIIVRDGRTVLRSPVQQHDFNFTYQLESGGGSDLPSSAQTFHFPVAPGDVIIAGTDGLFDNLYSNEISAIVVEALRTGLEPEATAKKIAALAQQKAMDRNRQSPFAAAAQEAGYRYFGGKLDDITVIVSYVTSASAT

>tr|A0A0D6R0W2|OS=Araucaria cunninghamii

MERVLTASSQCLKFITTEFTIKCKIQGAAASVAAWSPKPRKLSTPTLTAAFLVPPSTSRVCCLSAREHTTNTGFYQNQAPLDNKVLKLISGSCYLPHPDKEEIGGEDANFVLEQTIGIADGVGGWAEVGINAGDYARELMANSVAAIQQEPESFIDPARVLEKAYLNTKSRGSSTACILTLSDRGLHAVNLGDSGFIVTRGHHTLFRSPIQQHSFNCSFQLESGDGSDVPSSAQAFTIHVAQGDVIIAGTDGLFDNLYDSELSALVVDAVADRLGPQATAEKIASSARQRALDKDKDTPFSDSAQSAGYWYRGGKLDDITVVVSFVTAID

>tr|A0A0D9ZES9|OS=Oryza glumipatula

MLAGGGSRVSGGGGGGGFNLASYKYKDPLLGRGGRSFLFGNTWFLLSTYPARLLHTADRRAPAAFFAAINRTPCVRTHCTGQSLLQRGIVMAACGYAFRRAELGAAKRQPEKDSSVGTRISRVVAMGSAGSTPRPEVSFRHRGVEYCKKVGVSLKCREPWGPSRTFWTNAIGPSYKLSFSVEPWLRDFSTSCVAPYSAGATEHQLSLDEAVQDKQMDNSTVGPDGKPRAPGPLKLVSGSCYLPHPAKEATGGEDGHFICVDEQAIGVADGVGGWADHGVDAGLYAKELMSNSMSAIKDEPQGTIDPSRVLEKAYTCTKARGSSTACIVALKEQGIHAVNLGDSGFIIVRDGRTVLRSPVQQHDFNFTYQLESGGGSDLPSSAQTFHFPVAPGDVIIAGTDGLFDNLYSNEISAIVVEALRTGLEPEATAKKIAALAQQKAMDRNRQSPFAAAAQEAGYRYFGGKLDDITVIDSKTVHYRKKSATIIRLRGSLDSKVNSSTFQI

>tr|A0A0F2LYU9|OS=Sporothrix schenckii 1099-18

MRRLPLRLRTSPSSRFPPLRPAPSGSSRPVLSGQCLSLGGSSSGGHPRVRCMHGSPHMSFPVITERPSASGSTDAPTMFDATNHNDIDHHGSPARRRCDSHTTSSSLHPLPKLPFHFDTGVGLFAKRIPRPFPPPFLSPPSGSFSDPLSTHDRSRDRRELVDGHLIGGCTNGDDAVYAGEYFVAANDGVGAWSMRPRGHAGLWSRLVVHFWANAVSESVAAGSITAPEPIKYLQRAYEQTIEATRAPNDWQGTTTAAGALLSYRQIEAADDRAAYTDENEPVLYVTNLGDSQIMVVRPAEGKIVFKTTEQWHWFDCPRQLGTNSPDTPRDNAVVDVVPINVGDIVLAMSDGLIDNLWSHEIVEQVSKSVAAWQAKDKTPADLHRGMMTAVAEELVEAARVIAVDPFAESPFMEHAIEEGLASEGGKLDDISVVAALCRRSDAT

>tr|A0A0F3GLA3|OS=Candidatus Magnetobacterium bavaricum

MTYTISYAGLTDKGRIRKRNEDNWSVDPEKGVYIVSDGMGGHAAGDLASKLVVEMLPLLLNKRMKDITTLNSPKATEQLNVALTELNEQVRSISKSKPGLSGMGATVVLLLVREMTALLAHMGDSRAYLLRNGKFKQLTMDHSVIQVLINSGEIKPEEASNHPARGKITRCIGMTGEALPETMLLDLKPRDRILLCSDGLTGMLTDKMIMDIISEYRDSKAACQALIDSANNAGGMDNITVVMVDCQGDQTDTDDIIAISETEELGSGTGNIMDMSVPVGFVINLDKQPSDFFLPVSYPSFSIGRGTSNNLALKNDRTVSRSHCVISVDGESLKIKDLGSRNGTYVNGRRIKGIVDLPIPSWLSMGRTRIGVIPAGADFKQEALIDEAYTTEGSILIPPSEFFEERTEALLVVDIVGSTRLVKGGETQLVKVVSALGQMLDRSLQNEKHPFLKCTGDGFFATFGAAEDALKSGTKLSAGLAKYIKFPVQICVAMHWGSVRLTQEGERTGRNAHAVFSLEDLRHKEKNVTALLTTNNKREIILMTEAFWNMLEQQTRARAVAIGKFNLKGLDKEEKIFHWPM

>tr|A0A0F3GYT7|OS=Candidatus Magnetobacterium bavaricum

MSVHTWTLDFGNEQDKGKRKGQEDYFASFSPARGVVNSDSGFLAVVADGMGGHTGGAKASVLAVNTFIEAYKRKTAQQAISDALADALASANQMLVDTNIKTGQDSDMGTTLAAVVIQENRLFWASVGDSRIFLYRGGRLMDVNETHSYGAELDVKHRLEQISTEDLEANKKNRGMLTSYLGLKEIPRVDISHRPIELRCQEKVLMCTDGLVDALDRNEIAALLQSNKSAQEQCEVIVQHALKKNLQTQDNITIIILELKDDAKKQLIQQEREKTLDDVTLPLNTLDYYNSPEDMGQTTKIKTENPDTEVATAKTNSERSKLKVGLAIAIVVIIIAALCLYILL

>tr|A0A0F7SS08|OS=Phaffia rhodozyma

MSLAQNPSSKKLINVSSRSLVQLQRHAARSTRTSPRRPLSTHSRGGLQVVYQSNATVNHAAGQAGPQPSLTHCHPSTAPSPPPSSSPLPSSLPGPSSNSPPSLSFFEFSTFSSTGNPVTSPPSSNSTSASSPSHAGNKHPRSSPPSSGSPSSLLSSFDPTSTYPANASPAYSPSALEIWSLVENPGSIGLGSSSDVSAGLRRRRLVFDAGAFGIPKTTTGGLTRDIHKASTPFSIGPGLPDGSHPHGHSYTGSSSSAGWNSEGESESVRVGEDAYFVKTSAGPVVGGVQALGIADGVGGWSRKGIKGANPGRFSALLMSHIASLLPPSSTLQTILSQSAPDPVGIMQAGYERTLEQAAREGFVGSSTALLAVLKGEELRVANVGDCSLWVIREGRMVFRTEEMQHAFNYPFQLGTNSKETPIKDAKSYTVKVAKDDVVLLCTDGLVDNLYDEEILEEIASFSPPSVSSSSTPSSSSLSSTSTSAHSSTPSSPASSSTTGTGNGASDNTGSPSSSPSTPSSPSPSSLPHQPNQRPPISPQRISEALCSRAKAMSASRGTPYGPKQETSSFVGEGGTGGLGNERFEIPSPGLDVGVDGAGIGNGTWRGRDINSPFGVKASVCGLKFWGGKEDDISVLVAIIGDPTVSPPHA

>tr|A0A0F7SU59|OS=Phaffia rhodozyma

MFRPPLVFPFSVPRSSTLTSLPILFRYFSSPPPSSTSPNPARTLKDSPNFTFEIGLSFAGKPRRRGEQIPRGISSGGEKGEWRERMINWTEGSSEFNGETSSGKVGEVRVKKDKKDAGEDFFMIHERPADQLTTLAVADGVGGWAESGVDPSVFSQALMYHAWKAAQGDVSAQPKDILQKAYDGVKVDAEVKAGSATACVLQLDGSDGKLTSANLGDSGYLVIRKNNEFLDVPGAQQHYFNCPRQLAKVPHRRGYETNITDLPRDADLFEVQLEDGDVVILFTDGLGDNVHNSEVLLLLAQAQIETPPPSPKFGVTASKKLSVAQKLADGLKTYGQVCMGSTEKVSPFTLAAAKEGLNFRGGKTDDITVIAVRVKKNQQTSA

>tr|A0A0F7ZN32|OS=Hirsutella minnesotensis 3608

MRRAQPGRLTPPPSSSSDHVASRSAKSPFSAAAAVPTTSKFSYHVAASFIAKDRPYDPSTHVFHFNPYNRIQPPRSRRRSSRPDSGHDAFFVSRVNETGAVAFGVADGVGGWTDSGVDPADFSHGFCDYMADAAWSHDATARDSLTARKLMQKGYDAVCNDNSLHAGGSTACVAVAAPDGTLDVANLGDSGFLHLRLNAVNAYSEPQTHAFNTPFQLSLIPPRLAARMAAFGGTQLSDLPRDSDVSQHHLRHGDLLILATDGVLDNLFNQDILRIASRVMVSSGAWTMTESGGVRVADSVDSLVLPARSADIKDKKSERTITLQSLLATELVTAAKAASVNTKIDGPFAKDVQKYFPHERWHGGKVDDICVVATVVCQEPPATKSKL

>tr|A0A0G4FYW2|OS=Vitrella brassicaformis (strain CCMP3155)

MCPYRIPDPEKAETGGEDAFFVCETAGGGALGVADGVGSYAAFGLNAKMFADELMGGCQQAATIALTDTATPTSSGADDGGASAGVNAEAAGAYLSEIAQLAPAQVARRLLQYGYSCTRSIGASTALTAHLDRASGRLGVANLGDSGMIVLRRHEKGRMTVLLRTKEQQHEWKMPFQLARSPDESNRDALEKDMKELVRQLREGKRLGVVPDKPEDAQLYTVALREGDLLILGTDGVFDNLFDDELCGIADLTLSPSESLVLNDPSLATPARSVAAAIAEAARHRSMDPHCKTPFAKTARQNNTHHTGGKLDDVTTHRPTPSSGAGGGETVAVKEGTCRPREAYLQEIQRVDLPPEEIALRILQYGYIYVHPCLWLITALVVVYLDKKGETLAVANLGDSKMILLRRQKNQRMTVPHHQRAAALLELPVPTL

>tr|A0A0G4GF58|OS=Vitrella brassicaformis (strain CCMP3155)

MPVLLTAFAVSVGGKLIALATSKAAIGFAAFYLGWQGSGIADKAVEKALDTWKHLNGERSASPPADSPAPSPPGPPPSPSAAAPPTRDPLRRPPKTFASAECLIPLDGKDGGTEDAACHHNEAGVIAIADGVSDIKPCYGFSARLLSRWLMSRIREVAPLYAISRWTENRAVDILLHVWGERALGEGQRGACTVVVVAMVRDSWIDIDNLHVAAWGDSVALLLRRDRFGKLFILYRSGIMVEPSLPDVPVQLSALPRTVGWTTTTRVNPSPAVLRRSLQQLKWSVQEGDLLLAMTDGVNDNLFHEQIESVANQYEPYAEPAVIAQAICEAAHSHSVTPTSVTPFGTLRHRHGGKPDDISVGAAWVRRAAGGDDAAYAAAQQELVPRWLWPQPPRDRRRERGERARDR

>tr|A0A0G4LNB7|OS=Verticillium longisporum

MAASLTAARQPLALCTARQPMSIAIGLVSKPLPRSSRPTSSHLGRPIPARSMSSTPRGGPFSFHVAASFIGKDRRYDPSTHVFHFNPYNRIQQTKHNKRSRPDSGHDAFFVSRVGDTGSVALGVADGVGGWVDSGVDPADFSHAIGLVSKPLPRNSRPTSSHVGRPIPARSMSSTPRGGPFSFHVAASFIGKDRRYDPSTHVFHFNPYNRIQQTKHNKRSRPDSGHDAFFVSRVGDTGSVALGVADGVGGWVDSGVDPADFSHGLCEYVASAAYEYDPSVSSPADPSTPPSARSLLQTGYQSVCEDRSIRAGGSTACVAVADPSGSIDVANLGDSGFVQLRLGAVHAASEPQTHAFNTPFQLSVVPPSVAARMAAFGGAQLSDFPRDADVSRHGVRHGDVLIFASDGVWDNLFNQDILRVASRVMAGAGAWVTAAEGEAENGGTRVIDDLASLTEQQQKTTSKSAVTLQSVLATELVAAAKAASVNRKLDGPFAKEVQKWYPHENWRGGKVDDIVVVVALVSDDSKAAPPKSKL

>tr|A0A0G4LVT4|OS=Verticillium longisporum

MSSTPRGGPFSFHVAASFIGKDRRYDPSTHVFHFNPYNRIQQTKHNKRSRPDSGHDAFFVSRVGDTGSVALGVADGVGGWVDSGVDPADFSHGLCEYVASAAYEYNPSVSSPADPSTPPSARSLLQAGYQSVCEDRSIRAGGSTACIAVADPSGSIDVANLGDSGFVQLRLGAVHAASEPQTHAFNTPFQLSVVPPSVAARMAAFGGAQLSDFPRDADVSRHGLRHGDVLIFASDGVWDNLFNQ

>tr|A0A0J0XVA8|OS=Cutaneotrichosporon oleaginosum

NPPPPPPPPAVAAIVRVPSPPHPAKQARPAEVHSVLAPAPQLNNTTTLAPNPHFTYLGARSDGIDLQSTATLPDPNNLNLYSSSLIFHLGSSGAPKERTHIPPSRKRAVEPPPPRQRSFPLPDVSPPATLQSVGVGEDSYFARVDGVCVADGVGGWSRSGKGPGDPGRWARLLTHFCEEEVERWWAGAEDYMTTGDDVSGWAARAWKRGSEDRKERKHRRPLDPVEIMQKGYEKCLACAAQEGIYGSSTCILALLHHSTLLVANLGDCSLLIIRRGEVVFRTSEMQHAFNFPLQLGTHSRDEPMKDAKRYDIGVEKEDVVIVGSDGLMDNLFDEDILETLSEFAPPNQPGSLPAFSPQLVSEALLDRARAISEQTTATTPFMMRAIEEGIDFVGGKKDDISVVVGVIGDRD

>tr|A0A0J7NHB7|OS=Lasius niger

MAMAVDPSDIRAKTVMKGAVCIDFADIVAFFIFKHAIQGFMPCISIFFRSMAGIAYAIDPMQIDDFVRRADHGDVQAEYVLGISFLKGDGVVQNDFLAKQYLKLAAAQGHQAAQIQLDKMNRKSKPIIALQPNFDRTLPLQPKIGQTIATKPTEQLDDPQKERGGGRMFLIFELALGAVCVLLGFQGLHPKALEAFLKDFPKGSDLHNHLTGAIYAENFLQWAGEDGNCVAVDKQAILPTFCVKPQPQKNLLPAKEILSDKAQEEKMVDAMSMRHFAYRPGGETGHDHFFAAFHRFDGLKTFRMGDMLAEATRRAAEDHVHYVEFMIAPTVVEAINAGKKLPDSKETGDTRLKFWEQSLAPELPNLVAQAEKEIKDAEKRSRKLLGCDEKNPQPACSVHIRYLYQALRTYSPKEVFAELSLGYALSAKDPRVVGVNFAEPEDNEVAVKDYAEHMQMFAFLNKAYPSVSLSLHAGELSRDLVPESALKDHIRQAVEVAGAKRIGHGVDIAGEKDHQQLLKEMADKGIMVEVNLTSNDEILNVKGKAHPLSLYKSFNVPVALSTDDEGVSRAIEFAENPENRCPCILLLDVSGSMSAGMFSPKGPIDDLNEGVRLFKNELEKDSLAAKRVEISIVTFGGDVKIAQEFSTVDEFHPYDFKATGLTPMGEAIETAIDLLKNRKSQYQANGIKYYRPWIFMITDGGPNDDVSRAMELIKKGHRDQQMSAHIGDGAIVTCNEFQTWECFSEPHHGEYASTTYFLTDEVPQIRISVQFKNIKGICVFSDGLEGLALNLSNNVPHQPFFQTLARPLKKSLSRKKTGCNKVLSKGLGELLLSEKVCNRTDDDKTLILALVR

>tr|A0A0J9XAV7|OS=Geotrichum candidum

MLKHSKNIFQLCRHTLRSSYTAFAASNSTASMAATTTAPNNFYRPPRAPCQQPSLSPFEDDLSIPGLSTAPNSPIHKNPKITDLKDYSDSLAPPSHDFNEIHSATPLGRIITEPPHPTLFPSESIVNHNISMPTGTSIVVDESPAVATSIPVPAPTSEELGSAEETFEKSNNLDDFPVTLEFACSGKPYHYPKPSSTPESSHKYFENIINSLPKRVEYDLKLTYLDKQDHQAFNSLSVNDTLVSMGCGEDSAIGTHQLLGLADGVSGWTDMSGGHASLWSRLILHHILDQYVSDKSLALPPSEALDEGGPNLKRVLDKAFESAKANISELHEIGSSTLVLARLRPDLKLDILSIGDSSIYIIRDGTEIVFTNNKTESAQTPDVAESEIATDNNTTTDTKSTASTITEAGSKHDLEDEKTMKEFVNCPQQVGTNTTVVPSSYAQLYQVTVEPNDVIMLCSDGISDNLWQNEITEVIAHDNLDLQKKADMLVQNANDKAVDNFAVCPYQLRSPRSTGGKSDDMTVLLATVKV

>tr|A0A0K1EG88|OS=Chondromyces crocatus

MAELAAALLLGSSLIGLVFWYFYMKDRSRRNAAAGAQPTPPPSEAKDSGTRSDSLPKESAAPLSSGPASTSRGVTAPRRRSGPDYEDEEEIDHKDEDDDLTLITLTPPEVINSPISRPSSPSYGDEEEEAREEREIEQPSAVPIIYDDDAAIDEPTSSSPLVLISAVGQTHLGQKRRKNEDCYLCMDDYHLFAVADGMGGHKGGDVASRLAVETIQKAFEEANWTEEAPYPDVPRRGSELATAIQRANKTVYDASQVDRSLMGMGTTLISARFSPNKQRLYVGHVGDSRCYRLRDGELRQLTTDHTMGAAGITGPLANHLSRALGVASAVKIDLVIARPRPGDVYLLCSDGLSKMATDDAIQDVLVAQPDPEKAAAALIDRANEGGGRDNITVILVRVSDPKDLAQIVKRAS

>tr|A0A0K1Q3N5|OS=Labilithrix luteola

MRGRTAREEVSFSMSDVIWVLSCLVSLVLAIAIGRLWSGGGEESEDATPAPTPKARSLRPSSEADRSERITVPVPQANTDSHEADTPQAPVAEALPRIDFEDDEEVEPTLVGKKPEQRLVIQPPAFKILYDVDAADDEPTQAKALIFVSARAQTDRGLKRKRNEDSLLVRDDEGLYVVADGMGGYNGGEIASGLAVKTIDDAFTESRFEGVPHATIPPRASELARAIQMANVAIQSRAESERELTGMGTTICAARFLARKQRLYVGHVGDSRLYRLRNNELSQMTSDHTMRDYGVTGADAAHLSRAVGVWPTVPIDVIVGKPEPGDLYLVCSDGLTKMLDDDRIRETLLGSTDPKRMVDALIKAANDSGGKDNVTVIVVRVDPPAEAPERAA

>tr|A0A0K1QDG0|OS=Labilithrix luteola

MSVRKAAPARPSDDEADDADLTRMSPSMKSPATLPQLANSEAPDETMTTSGSLTIFEPDAEGDETTGSSDLFLLTAAAQSDRGMRRRNNEDAYLLDEAQSLFVVADGMGGSAGGAVASRIAVDSIEHVFSTGPIETVDHPRRPRRARELVTAIEIANEMIHEQAAKQPEYHGMGTTIVAARFVRRKRRAFIAHVGDSRCYRLRGGQLKLLTTDHTLGAQGISGGFSGYVRRALGVRSRVKVDVVVDRPQSDDLYLLCSDGLNKMVPDEEAKALLESQQSDLDAAARSLVEAANHRGGKDNVTVVLVGVRESKRRSHRMRARLSEDRRSS

>tr|A0A0K8Q1V9|OS=Arthrobacter sp. Hiyo6

MSELTITALRFGFLILLWVLIFSIVSAMRRDLMIGRKAATGAPTARQVRKHPELAEAAPAPVRQQAHQLVVTEGPLKGHSIPLADSPILLGRAQEATIVLEDDYASGRHARLFPQGSRWFIEDLGSTNGTYLPISSSPGTAGGARCPRENRQDGHRIEAVAVAVPEIPAEKAEASKRPLIMRYAARSDVGRIRAKNDDSAYVGRHLAVVADGMGGHAGGDVASAATVLDMLHLDHGDYDGDAGTVLADEIQTANSLLSELVHINPKLAGMGTTVTALLLAEGKLHFAHIGDSRAYRLRNGEFKQISVDHTFVQRLIDEGRLRPEEAETHPHKNVLMRVLGDVDASPELDLATLDVEPGERWLLCSDGLNYVAGHVVERTVRETKDLRECVEKLVDLTLEAGAPDNVTVVMLEIAEQTPDDVRTAAVGIVEPDASAHGDKATAADESPHPPHPR

>tr|A0A0K8VNR5|OS=Bactrocera latifrons

MHSLSWTTRAISRALRSGFSTLLETASNATANVNSASKQRPHFVSVVCGFPKDFLTSKYKPGKYGEDSWFKTSTTNADVLGVADGVGGWRNYGIDPGEFSSFLMKTCERLVHCVNFNPQRPVNLLAYSYCELLEQKKPILGSSTACVLVLNRENSTVYTANIGDSGFMVVRQGEIIHKSEEQQHYFNTPFQLSLPPPGHGHNVLSDSPDSADTLSFSVKEGDVILVATDGVFDNVPERLLLDVLKEVG

>tr|A0A0K9NIZ2|OS=Zostera marina

MFSGYIIRRKGEQAMSSFASILSGIEASSRQHRSFRSPSMFRNAQLAISHSKGSRSRSKGSFEQTVVPCIITFVNISSGGKSIGRRLASRALNGCDNVVNRRCFWADMAWIRTMCFRFSCSVAHALPLFDGSSVTDERHQIFETSSDLKGLGERTIKLLSGSCYLPHPDKDETGGEDAHFICVEEQVIGVADGVGGWAELGINSGKYSRELMSNSVAAIFDEPKGAIDPSRVLEKAHSCTKSKGSSTACIIALNEKGIHAVNLGDSGFIVVRDGCTTFKSPIQQHDFNFTYQLQSDNSSDLPSSAQVFKFPVIVGDVIVAGTDGLFDNLYNNEITAVVVHAIRAGLGPQVTAQKIAALARQRALDDNRQTPFSSAAQDAGYRYYGGKLDDITVVVSYITASTV

>tr|A0A0K9PUC5|OS=Zostera marina

MLHTNLSNTKGLLLLSEANNLVCGMSILSSSSPILNGSSSASLVFRQFHHHYSKNLLISSPRISMPLFKFGFVNFAVLNKQKCVPSLNVSCSIKKLSCGRGVSGNLNYWRRPNGDNMLYKYFWSDTVEPITDSFISSCLSPPHSANETANVSLYFEGSSKNLNSEDSTANSDKNKPGGKKLNLLSGSYYLPHPAKEKTGGEDAHFICVDEQAIGVADGVGGWIEAGVDAGIYARELMANSVAAIEHEPKDLIDPSRVLEKAYAHTKAKGSSTACIVALTDQGIQAINLGDSGFIIVRNGAIIFRSPVQQHSFNFTYQLEYGRDQDLPSSGQVFTLPVVPGDTVVVGTDGLFDNLYNNEISAVVFEAVRTGSSPYQTARKIAAFARQRALDKNRQTPFSTSAQEAGYCYYGGKLDDITVVVSYITASDT

>tr|A0A0L0D392|OS=Thecamonas trahens ATCC 50062

MPAPSKERSEDAMVVEPEHGVLAVLDGVGGWASVPGANPAEFAARLADALRAAAAAGAAADADADADAAAADAHRVVDLADAAVASLDKSTIMLGSSTMNVARIGRGASPELHIYNLGDSQARVLARTGDDGYAIVASTENGPSSQIVFNMPYQVGRNGNAVDDGSLFSVPVARGDVVLLTSDGIHDNLFNNQIVAVVDSLLGAASGPLQDLPHDELSTLLDAAAARLIIDARFHAERDAPTPFSPHGGKPDDSTAVVAVVV

>tr|A0A0L0GAH9|OS=Sphaeroforma arctica JP610

MLACSVRPHCMRQLTGKLPHTIHTNLVLRTIALGANRLQRSNIHSLSSDSINHTYGSGLTHTNSVCTHSQKQLYIPRQYISYTPHPPVRISCAYQGKPRRPAARPSPINTYNTPTQPHLYTHLDHNDTNMARLPDSGEDAFFVHETDDAHYIGVADGVGGWQEMGVDPALFAWALMNHTRDAIREGMHLDIRDALKYAHEKIVEDGLVTHGSSTACVVKVEKKTRIMTVCNLGDSGLQIYDRDGKLAARTPSQQHGFNFPYQLAVMPPDMREGAMMHTMADGDLFQYQMKDEQVMLLATDGLFDNMFPSDIEATFKEVGVGNMDKLATSLVRKAESLSKDQRRRSPFTVEAEKHGYTDMLGGKPDDITLIVLAV

>tr|A0A0L0SZP6|OS=Allomyces macrogynus (strain ATCC 38327)

MIRSARPLAATRRTLASAATAAVAAPSTRAAAGLASARRSHATAVPSTTLCANAACSRPTARTSSSSALRLSRSAHRALVPAGIAATALVPIPSTCPRCTHARPARRHASYSTASLVPAAPTSLVFNHFGTGFAKHAQALVVPDRRSPLKSMQCGEDAFFSRYNCVGVGDGVGGWQGQANANAGLFARKLMHYCSAELERAHEVPPNVAARDEIDIDPVGVLQHAYHRTVQDAQSEGFVGSTTACVVLLVHDELRIANLGDGAVMVIRQGEFVFRTEEQVHSFNYPYQLGTAASDLPTHAQQFRVKAHHGDIVLVVSDGILDNLFEEDILAAVREHVPKPTVDPPVVPLARDPGLPAGAAGTAETASSHPRGFASWLTSPASSLAKSVRAAVAGPPPPPPPPATEPVAPIPDPSAHPLGHDVPTLPPVSSAGATDPAAAAVPDFGPPHAIPAAYTGLLALADQCRAASVADQVALARAAAVAAAAAADPRRIAEALARRAKDVAEDVRCASPFQTRALHEGLYFQGGKTDDISVVVAVVRDPAQARAEGLAAAAAAAAAEVEAGVGRGADAGASMGGA

>tr|A0A0L9UQU2|OS=Phaseolus angularis

MTTASHCNAVLGGVCVNGLISGCGSVLDFTKPAVVYLKDKTFKGCVRGSGNLRRPQPSFGSLSFGSSTFDGNWRIRDSSLLHGSWLKSFSTSTSACPSAGVARAVSFDGSPPDEQLANSSFSPDETTVGGKTLKMLSGSCYLPHPDKEETGGEDAHFICADEHAIGVADGVGGWADVGVNSGLFSRELISNSVRAIQEEPQGSFNPTRVLEKAHSNTKAKGSSTACIVALTDKGLHAINLGDSGFIVVRDGCTIFQSPVQQHDFNFTYQLESGNGGDLPSSAEVFTIPVASGDVVIAGTDGLFDNLYNSEITAVVVHAIRAGLEPQVTAQKIAALARQRALDPSRPTPFSTAAQEAGFRYYGGKLDDITVVVSYISGSLTE

>tr|A0A0M1JLJ3|OS=Planktothricoides sp. SR001

MLVCPKCQYTNPINHKFCQKCGISLTQKACSECGTQISLSALTCDHCGAKTGKTLWGIILPKTGAITPREEVSLEDQSTQVETEEIFAINPPILNQTDISSETNKIDVLSSFEEAENTQASQVVTEIGQPDEGTKPIEAEPSTNIEETEDTMAAAVVVEAAETVVETVVEDVLTPQTPATETPATETPALETPAPETPATSKPDFSMAPGDYLDDQQRYQLISQISEKTKSGDWQGCVLDCQPLQVSPLLTSISASSTASRSGSTTVMQPSINLIPAIAQPYLRLRNQWAPIIPLIHDAWENQDHSILLIEDRSNFPRLIDQCHQLTQVEPLDLQPFIQWFQQMTQLWQVLTPLKCRQSLLNIENLLVDQISGGLRLQRLYGDATTAPTLAELGKIWQQLLPIDSNDSASNHPEIIQDVKCVVNDVVNENIFTADKLLATIQAVSGRNATAMLHPQRYAIAHPRHDNAELSHGDDLSTMLHPQRYANVMTEGLRNLEAIGATDVGQQRNHNEDDFAIKTDIFKSQTSRDRSLQFRGIYIVCDGMGGHEGGEVASAQAVKTLQEYFATHWQDPLTLPDEATIRAAIIEANQAIYDLNQAEVRSGSGRMGTTLVMALVNQNQIAVAHVGDSRLYRLTKTQGLQQLTVDHEVGQREIQRGIDPEIAYSRPDAYQLTQALGPRPENFVQPDIQFFPIEENSLFILASDGLTDNDLLENHISTHLEPLLNSDIDLNQGVKDLIALANDYNGHDNITAIAIRAMVQQ

>tr|A0A0M3I7F9|OS=Ascaris lumbricoides

MLVLDVMNSLPALAVANSVLQLDERSAPLGPSSRPGIFEVLHPLSGCGGADCEATEALAEVISVNLHIVVLEYSTSRRYIILTNMIRGKMLVSCGRVVVRAVISAGFIEQSLPLPSDCRPSVSKCAHVSTTSAHQPDATPTRNVHASCCGFPKDMVNGPSVVRDHGIFGDDACFIARFRNTHVVGVADGVGGWRRYGIDPSEFSSRLMKICSDLVQLGEFEPCRPDRLLAHAYEAMSAPPRPIGSSTACILVVDQDTLYSANLGDSGFLLLRRGQVVYRSREQTHYFNAPFQLSLLPDNAGAAGFLGDPPEKAELNSIDLQSGDVVVLATDGLWDNVSENLIVEQLSNIQPGDIQAACNTLALTARRLAFDSRHLSPFAMKASQHGISAVGGKPDDITLVLLLIA

>tr|A0A0M9ZMW6|OS=Streptomyces sp. MMG1121

MSQMPQQAALSKCPSCAEPVESGDLFCGACGYDLSVVPAPPQDHPTLTMTTPEPAGLGDTPAHGHEHGSGVRFDRPEDPEEYPLQAPDPRAAADAADATGAVPAAPDAPEADPSQVCVACRAGRVDSDGYCENCGHAQPRERDHVERESGPVAAVSDRGLRHHRNEDAFTVGHTALSDGTPATLAIVCDGVSSATRPDDASLAAARTAGDALLAALPRGTHPQAAMHEAIVAAAQAVDALAGEPATAREHTPHQNAPACTIVGAIIASGLLVVGWVGDSRAYWVPDDRTAPAARLTEDDSWAAQMVAAGLMSEAEAYADERAHAITGWLGADAYELEPHTASFKPDRPGVVVVCTDGLWNYAETAEEMSEAVPADAALRPLHSARVLVGHALDGGGHDNVTVAVLPFPAPPQGAGSA

>tr|A0A0N0D7I6|OS=Candidatus Magnetomorum sp. HK-1

MNNWTIDYGNDQHQGNRKEQQDFFASFEPSMQVVKSKSGFLAVVADGMGGHKGGAQASLIAINTFVKEYQQKKDNETIPEALNRSLIKANVAVFKANMDAGPNADMGTTLVAIVLKFNELYWSSVGDSSLFLLRGNKLDKLNEEHNYGADLDRKVLSGQLSKPQADAESNKRNMLTSYLGIEEIPKIDLQKIPQELVINDKIVVCSDGLVDALIPDEMISCLQSQGSSQDKSELLTKTALNKQRKNQDNITTIVIEIKPEKSNIKISKEPNDDFKKKEKADKKKKMNLYLNSAIILLLIAIVTLSYFIYKKITKQDISQPESEYTQSLTGIKNNIDKPTPEPEKKSKTIKNETLQKGNQQDENQKNKSPEDSSSINNNDPYLYLKTQVVLREIDQYMGLIDNKPGQKTKEALKTFQKIHKLNPTGDLDPKTIKKLEFQFGEYKAKIQKEIKRIESKKNDRNYILKVQCILKHFGKNMYTGDIDNVFGSKTKEGIKALQKARGLEVTGQLNNKTKKLLKEYFTIHKSALKDCINHYNTSIKSISSQKTTENYQNHSLQKNRENSEVLEDLDSEPPVSDEVPVSDEIGKGSVSSAAEDPEDQNLHNTFTEHSPNPDKISPSVKQ

>tr|A0A0P6VFF4|OS=Prosthecomicrobium hirschii

MQFEFASGQIQGKRAAQEDTVDVLLPSDSHEKPGELCRAGAAAAVVVVVADGMGGHVGGRIASNLSSKYFLDAVNKTPGSWGERLDQALIQANDILRTATEKRPELDGMGSTLVGAVVNQHGIGWVSIGDSGLYLFRDHALVRLNEDHSFGAYLDEQVRQGLLAPEHAAMDRRRNQLFHALLGDPIDHYERFAGFRDLRPGDAVLLASDGLKTLSDETVGRLIAEHEDESAEQLVVRLLEAVDAENRERQDNTSVILVKVRDGVAPGRTPATESSTTVPLNMIAADVLDPLHPGEAEGASSMVDPTGHRSAATRETAPPAAGRAAPAPVMAAAAAGPAAETGPEAVTETGSGTAAGRAGGGIVAALLGLAIGLGIALWLYRGRLPPWPF

>tr|A0A0P9RY45|OS=Pseudomonas ficuserectae

MHMDTSFWKAGHKPTLFAAFLYFDLSFMVWYLLGPLAVQIATDLHLTTQQRGLMVATPILAGAVLRFFMGLLADQLSPKTAGIIGQVIVIGALLAAWQLGIHTYGQVLLLGLFLGMAGASFAVALPLASQWYPPQHQGKAMGIAGAGNSGTVLAALIAPVLAASFGWGNVFGLALIPLVLTLIAFTLMARNAPERSKPKSMADYLKALGDRDSWWFMFFYSVTFGGFIGLASALPGYFNDQYGLSPITAGYYTAACVFGGSLMRPLGGALADRFGGIRTLTVMYAVAAIGIAAVGFNLPSSWAALALFVAAMLGLGAGNGAVFQLVPQRFRKEIGVMTGLIGMAGGIGGFLLAAGLGTIKQNTGDYQLGLWLFAGLAVLAWFGLLNVKRRWRTTWGSAAVTAARXPPMTLQLQFAQFSASGPRAENQDALRLVTPAPTLAASKGYLFALADGVSQCADGALAAQSTLQALALDYYATPETWGVAQSLDRLLLAQNRWLLANGLLTTLSALVLRGRRFTLAHVGDCRAYRWQAGTLKRISEDHVWEQADMQHVLKRALGLDQYVVMDYLDGELCEGERLLLVSDGVWATLGDASIRSILTEQDNLDSAVKTLVSAAHLAGSQDNASALLIQVDSLGEDDLGDTLLQLQQWPLPPALKAGQAFEGWTVGSIVAQSRQSILYRVTDTHGQPWLLKTLPASRHDESGAGQGLLLEEWFLRRVAGRFFPEVHPLADRQHLYYVMREYCGNTLAEVFTRNGPLPLAQWQDLATRLLRAAGLLHRRNIIHRDIKPENLLLADDGELRLLDFGLAYCPGLSTGNADDLPGTPSYIAPEAFNGAEPHPQQDLYAAGVTLYYLLTGHYPYGEIEAFQHRRFGTPIAASRYRPDLPQWLSQSLDKALQADPHQRYETAEQWLLEMEQAEHRPVVAKPRPLLEREPLKVWQTLALISMLLNLLLVIWLMSHH

>tr|A0A0Q6PUT8|OS=Leifsonia sp. Root112D2

MNVNVEASARSDVGSVRKVNEDSFVARTPAFLVADGMGGHARGDKASQAAVRVLGEFLPAGYDPSPDEVVAAVHAANDAVRALSTVDDSGVAVAGTTLTGVVQVHADDEPRWMVVNVGDSRVYSWDGRTLGQLTVDHSAVQELLDAGAISVGESLIHPERNVITRALGADDTIEVDVWLLPVTGTQTFLICSDGLCKELDDEAIAMILADTGAGHTDAASVADALVDAALEVGGRDNVTVIVVESQAGDVADDPDSTRDRPATAHEFLEDTRPRG

>tr|A0A0S3U6U4|OS=Leptolyngbya sp. NIES-3755

MKSPNYYARTETNQRENNEDFFNGFLIEDQGGQVPVLVVADGMGGHEHGEDVSRQAVLKVEDFLKKTIQQIADHSDPVEYLKHCLLDAIESANELVQRMVSVNGWDRAGSTIVIALVWQNKVIAANLGDSPLFHWSQKTGELVRVTQDHSVPGILAEAKLITEEMARYHERRGQLEFFLGNKTLPQPDPVYERTLEPSDLLLLCSDGISGSLSREQVRAILADSTIDLPDKAEQLIQAARDEGETDNQTVMLWRHKVPSQTTSTRKIAHSTAVQARSRSMVQTSQPTLIQSSIAPQVHSRDRRKRSISRKGLFILGAISLTITITLVWLLFQVIGVLQAHLGGSNLQPAANPVQQPIQPKPAPKVQSFQGSVITTIDENNQIIEWAAPKLKPGEKECRSNIPDVTDIQIIVSQSDEQLTTQSERTRCIRVWKPKLSQGVSPRPTTPSVEVPIVSIQGERA

>tr|A0A0V0Y1Z6|OS=Trichinella pseudospiralis

LMASCARLVREGRFIAHRPAQLLAASYQEVTRGAWAPGGGGGSPERPLNGSSTACIVILDRRSSEVHTANLGDSGFLVVRQGRVVHRSQEQQHYFNAPFQLTVSDDAVGQFFGDSPDSAETSTFRVELGDCIVVATDGLFDNLPASLIENELSKLESFEPMEVQRACNSLAFQARLLSFDRHSMSPFAKKAQEHGIQAIGGKPDDITIILAVVASAESEEEDEEEVEASVALLLLFLP

>tr|A0A0V1EIX8|OS=Trichinella pseudospiralis

MYNLYLCISFKVLANVLKLAGGAFEVDIFALVVKAWLVMCVQPAASYRPRLWSRLITLTISRFLTQFGIRYLSAPKCQRFVAARCGFPKNLAFQVSRVLEQDVYGDDACFIANHRTADVIGVADGVGGWRNYGVDPSRFSRRLMASCARLVREGRFIAHRPAQLLAASYQEVTRGAWAPGGGGGSPERPLNGSSTACIVILDRRSSEVHTANLGDSGFLVVRQGRVVHRSQEQQHYFNAPFQLTVSDDAVGQFFGDSPDSAETSTFRVELGDCIVVATDGLFDNLPASLIENELSKLESFEPMEVQRACNSLAFQARLLSFDRHSMSPFAKKAQEHGIQAIGGKPDDITIILAVVASAESEEEDEEERCPPPPLSPVAMFVTNCFGLPALVLTFLLASFSMTKSM

>tr|A0A0W7WB70|OS=Leucobacter sp. G161

MNSVELDFATLSDVGRKRQVNEDSVLAATPCFLVADGMGGHEAGDLASRAALAAFADGIVAGRPTSVPAAGETLNAARIAVAEVAAGRERGAGCTLSGAMLVEHEGELHWLVLNIGDSRVYMHRGSELQQITVDHSLAEETRRSDAGAPLPPRNIITRALGSSDSTADSWLLPVETGTRLLVCSDGLTTELEDEEIRAVLTMGGRADAVAAELVQRANDAGGRDNITVIVVDIIGWRPYLARRLSDRSTRRHRFGQDDHGDQAAAKPACTARVGGERVSTGDEPMGDVEDTATAPGPGRATQPGAAVPEPLDELSPELRAQLFKRPIDSRLRAPASPFPVSEDARPREGVRSSIPVMYGRRTENTGELRRGSVSAQSIGDPPAGYELPVAARTELPSIASMNRRFGLIAFVGGVGVSAVAVAGVWWIVGQLLP

>tr|A0A100YWD9|OS=Tractidigestivibacter scatoligenes

MAAEDQQTPLKDTPLEEVVPDVPPAGAVGKHAAAAEQTVHAAVDQSAPADEEATPAPQPKHAAPVEQPASSAPEGTHEEEAPEDTTQPQEPVNAPGRATIPVEGRAGADATCGKNAFISWGARSDVGLVRSHNEDSFLIRTPCFAVCDGMGGHAAGEVASSIAVETIGEKAPATADDTLLGAAIEAANQAVIKGAEEGRGKPGMGCTASAALIDGNRMAIAHVGDSRIYLLHAGTLVRVTRDHSYVEELVDAGQITADEARVHPSRSIITRALGSDPDMYADHFSIEVSNGDRVILCSDGLSSMVPDSEIESLAVSSATPQQAANNLVSAALTAGGADNITVIVVDVLNDGVAAAARKHSVGFALRVVVAMVLVAVVVFGGIFAFAKNEWYLGVDGDTVGIYQGIDGEVLGISLSNLVQTTAVEVSDLPANVATQLEHGGVRVDSEQEALSTVESYRTQIDAEKTKAAEKAAEVTAEGNPTGEAAPADGTQTTESGTDSGADSANGDTATTADTTGDTTQTNGGE

>tr|A0A134BH30|OS=Varibaculum cambriense

MSIGFNYAALSDIGKIRQSNQDSGYAGPNLLVLADGMGGPAGGDIASSIAIDHLRSLDNEADNPEEALESLREAISQAHQELCERSTQDPALTGLGTTCIAIRRSKDALTMTHIGDSRAYRLRGGELRQMTNDHSFVQYLVDTGQLTPEQAETHPQRSVLLRVLGDSQEDVTLDESHPEFTVGDRWLLCSDGLCGFVSGQTIGKMLYRSETPQQACESLVELALKAGGPDNITCVVADIVEDPRETEPQVVGAAATDRLAQLRSQQKEDSRDAATKADRDASEIASEDKSDPEDENEDSSFSQARAAIPTESLVEEDPAPKNRWGRIIGVIVGLLILIALFAGALWYGISERPDPTPSVPSTSVSEAQSPAATPEKTKQEVDPSTPAETSSSTSANNS

>tr|A0A136L6M3|OS=Chloroflexi bacterium OLB15

MENVPPTAPTLRANWLSITGSVRDHNEDAIGIYESSPASFFVLCDGVGGAEAGEFVSEFAVKRMLKAFHEAPKLPSTNWSAVMEAALRDVNSEVRRVADEATARSGKPVMMGSTMVAIVIHGWNAFVAHVGDSRLYHWRAGGIVQATEDHSTVSTMMIPAVNPDGSIKKNVLMRGIGKGATIEPDLLLLNLQPGDKLLMCSDGMSDKITADEIATAFNTMPLISIPEYLARTADTRMSKDNISVITIEIDSMAKPKEYQVPEQERAFYGYNSRWATTNFQAAGVNVGSGGAGSKRGWLLLLLAIIAVIVVGAVVVVASGGGGAVAAPATATVEPKGEVMQTQEVTPTATPAPTDTPTPTETPVPTDTPTPTETPVPPTATLRS

>tr|A0A136LLL9|OS=Chloroflexi bacterium OLB15

MNWFRKLLQPTKSQSAQEKISAPSTADEPEATVAELKSESPALKVTEPAAESSAPLNGTHAQKSEPESGKDLSEVVADLDNLITEPEPEDGLEATSPQETVVLRMELDEEPQPPTHATAPLPSTGDLMISVVDGATRPLPQDAILEFSPLTHTAFGQATDVGRMRTNNQDSAFSFFASGRSVDDIPDFGLFIVADGMGGHQDGEKASAITTRTVASQVLNTMYLPMISRQNKNNDVPIAESLANATQKANIEIMSHVPEGGTTLSSVVIIGDRAYIAHVGDSRIYMFHHGILEKLTRDHSLVQRLIELDQITIEEAADHPQKNVLYRALGQNDVVEVDTLTRRLPPGAMLLLCSDGLWNQVSDSAISDVISHVHSPQEACQKLVAMANAAGGIDNVTVILLQIPG

>tr|A0A137P1M4|OS=Conidiobolus coronatus (strain ATCC 28846 / CBS 209.66 / NRRL 28638)

MHRLYKVATTLKPHLTHSVRYRVSFRPSPSINRNFTQIPFTINLAQEVSIPNYQPPDSSTTAGRSSNSFYVPKPKLTLLSSPLNDGETGGNGAEDALSGSGIFSRYDISFLNQPSSSYEGTIPIVDEWKLPEHDFTSFDQKYHFNFGVTAFPKIRPNPVTQPLFGELMSFGCGEDSYFTRYDSLGIADGVGGWAKVKGSSSAFYSRLLLHHACQELSKYDEILNGLQDSSHSTPFASTSFSDPIQLSSTQVRYQTMDPVQILSKSYIKTQEEAKYRSIQGSTTACLVMLRHDVLSIANLGDCGVMVFRDGKVFFRTEEQQHSFNHPYQLGTNSRDMPEDAQSLHCKVRPYDVILVASDGLFDNLFEEEILEEVNSWMPVSTLSECKSFDSDIPTTVGKFSFQSTTLPNSNTILDPQRLSMTLAQRAKKVSYNRNTSAKSPFSQKCSEMGKVHYGGKMDDITVLVAIVSPHGSAL

>tr|A0A150H4Q9|OS=Gonium pectorale

MHSQDQAYPTTGSTGLLQPRRCLADDGLAGGAAVESAAARGIVTATLPNGGGANPESSVRFALSAGAYVLPHPDKMEKGGEDWYFVAKHRRAVGVADGVGGWSEVGVDAGAYARQLMGNAEAAAEEFAAASDQSSADQPELSPQDILESAYQMTTARGSATACVAVVNGNSLSVSNLGDSGLLVLRGGAVAFHTPQQQHGFNFPYQIGSADSMSDSPSSAQRFVVALQQGDLIVLGTDGLWDNCFDEEVASVLKYCGDRSMDVAKMAQVLAHYARHRASDSKFASPFAYAAFQAGYAYMGGKMDDITVIVCQAEALP

>tr|A0A150P7P2|OS=Sorangium cellulosum

MGQVREHNEDNFIVADLTKASRGLMEMDRYQVVGERGALLGVCDGMGGAAAGEVASQLAVDIIYQRMSAGGPPQHHDELAARLVQAIEAAGLRIFSEAKLDRTRRGMGTTSTIAALMDDHLFLGQVGDSRAYVLRGDRLVQVTRDQSLVNQLIEAGQLTEEEAETFEHNNIILQALGTADSVQVDLTYVELKRGDTLMLCSDGLSGMVRNEEIREVLRTVDDPIEACKVLTDRANQAGGHDNITVVVAKFDGDGLAEPDLVDIEELRYQKYSLPEHLLAQNAAASEPARKVKELDEKKISQRPPSPKSWLSAADGDLDEDGDDYDPVISPLPADXPCRRTTRWRSARPAPPSRATSRS

>tr|A0A150Q9X3|OS=Sorangium cellulosum

MMPPTESSVHLKIEFAQASDAGRDPNKQVNEDACGYAETKFGHLCVLCDGMGGHYGGSEASRTAIKTIFEMIEQTPPTVDPRAALKAAIEEAGRRVYQLGGPADNRVRPGSTVVAMVLHDGGVDIAHVGDSRAYVIRSNQIYPLTRDHSMVQGMVDAGMITEAQAIGHPDANKITRALGMKPEAEVEVRPEPMELYPGDVLIQSSDGLTDLVLSGDILGCTRQALASGSVEHACRMLVQLANHRGGHDNITVQMVRVIETGGRSLTIAQGPPGAGGAGGDEPRPSGPQETLAMTQPEGAAGGEPGPVEAPAAPAAAASTPVPAFVIAGRAAAAGPAAAASTPVPGLAATAASTPVPGLAATAASTPVPGLAATAASTPVPGLAATAVSAPAPRPASTSNAATVVPTGNSSTPAPNSDAITVVPTGSDRKPAVVPQAASSAPQLHSTTIDSTTAAPAIRPTVTDDLPRAAPKAASSMPQLHPTTIDSEASVVSTVTDSLPPVAPQAASGAPRLRPTLDSTPAVHLTATDSGPAVRPTAMDAALALPSTVPGAPPPNAPDAPAVLSPLPLSAPAVPAPSPGIQGAAPHPGPFGAPPHPGASYGAAPHPGHFGVAPHPGPFGAPPHPGAAPYGAAPHPGPFGAPPPPGAAPYGAAPHPGAPAPPPGAPYGAAPHPGSFGAPPPPGAPFGAAPHPGPFGAAPHPGSFGAAPHALAAQDAPPVSVGFSGHHAPAPGAPPAAAPHHHLMPSPPNAHGYGAPAPSFSGPVSSSAAPSVPTTVRAPRGATIAAIAAISAVIVVLLAVLIWQLTAG

>tr|A0A158AZD0|OS=Caballeronia calidae

MNTWSVGQRSETGFVRAENQDRMSWIRARAADIFVVSDGMGGHAGGALAAQLTVETLQRKLADFASLDGAAATLESAFQAANDAVFARGQSSNPATARMGATAVALLASGNQVMLAHVGDSRAYLLDRRGTLQRLTKDHSLVQRMVDANVLSEAEAANHPDASVLERAMGQAPRVEVEVSGWLRVHPGEACMLCSDGLCGYVEDDAIASVMRSGHAPQQTADALVRLALERGGEDNVTVQVLRYGARQARWRRWVLPGVIAGALAALALALAPGLGLGLGWLGMQSKRFSADDDARRVNDAASKAASTTPASAAAPDARLVTLEARVQENAANQRAADADLDRKLDELRGRIDRMEKRLDAGSHAGAHVAPRAESARKAARTTGARAAAPSSQHQAVSAAPASATDAAEGSP

>tr|A0A160T5S0|OS=Candidatus Promineofilum breve

MEATVPLEAAWRTDPGLVRGHNEDFVIVYEPETADDRRRHGSLYIVADGVGGADAGEVASQYASERALHHYLAGDGENWGARLIEAMQAANTDLRRLAADRDDSRRMATTMVATVVQAGRAYIGNVGDSRAYLRHDGTLAQVTRDQSLVARLVEEGALTPEEAVRYPYKNVILYSLGSEKRPPIDLFEVELGEGDLLLLCSDGLIRHVDDGEISDLLGRELPEAAAERLVALARERGGEDNITVAVVQHGARPVAMPVIAAPLAEDETLTRTAAHPRPVPVASPAPAPRRPAPVAAVAPHLWPLVFLLTAVMVVLIFLLWFFIQQTLSL

>tr|A0A161KBB8|OS=Candidatus Promineofilum breve

MTMAISHKGKTDTGKVRQHNEDAFYPDPRDGTHDPSGVAAAGQLFIVADGVGGNRGGARASHLAVTRLPAFFYGATNGDPVRGLKVAFNSVAHEIVSEAAANPDRANMSCTVVAAVIKDGAATIAHLGDARAYLLRGGLLQPLTTDHTWVQMQVEKGTLSAAEAENHPDRNVITKSMGNPSFPEPTVRQIALQAGDRLLLCSDGLCGLATDAEMAAVLNRAANPAAAVEPLIELANRKGGSDNITAVVIQAGPGGAVAAPPRRSNAGLIVGALVGLVLLLAAFVVLRPQGSGVGNGPPAAEATGTGGPGTVVAVVAGPTLEPGAPTSTLAAGVVATEMVTVTETVAVAVVATLPATAAAPTVAVVATPIKPVLIRPGASCQPGNVGQNYTVPAEGVRFVWTDNGNPPPAGAWIVRYGTSSGLKMVETPPQQDGNTWSVLVNGSEFAAAGDYMWQVSYTANGQPVASDRWCFRIEAAAPTDTPEPPQTRPSDTPEPPTPTNTPMPPTPTNTPLPPTPTSTPVARPTDPTATATAPAVPTASPTP

>tr|A0A167FCR3|OS=Sugiyamaella lignohabitans

MYTIKPNSLIAKATFENGIKKTNVGAVGGYCALRWFITATSKSRQRHRTLDFENNGRDHLQVSGNSSDISNKVSGPDSCNLADRDRRNKNNRKFAPSSIRYLHNVTKPRFRFTVAEAYCPKKRYEYDHQERTPRDSRNDILGGRLGRAHTGEDSFFIKEWTSDGPLKGSLAFAVADGVGGWAAIGVNPSKFSHGLCERMASEFKRTEDALSLQDPNSETYHDDRTTTASPQSLLTSAYKSLKDEGDSYAGGSTACVGVASAFTGMLSIANIGDSGFFIFRQRRVYRRSTPQTHQFNTPFQLSIVPKSMAKRRGRSPISDDPQDADLSQHQLKHGDVVVLATDGLTDNVFAQEILKVVTNGMWHSRSWIHEDEAAIDDLEPVDLQAGAQEIAQNLVRAGINASLDPNSESPFTLRLRQEMGFVAPGGKPDDITVLVMLVEELNHDGDGL

>tr|A0A167H382|OS=Beauveria brongniartii RCEF 3172

MPRRPYSASSSSSSFSAFSSTPSSAASSAASAPSSSVTSSKKPPRFSYNIAASFIAKDRPFDASTHVFHFNPYNRIQPPRHRRRSSRPESGHDAFFASRVHETGGAVAFGVADGVGGWVDSGVDPADFSHGFCDYMASAAWEHQPADTTSAATPANPLSARKLMQLGYDAICADKSVLAGGSTACVAVASPDGRLDIANLGDSGFLQLRLNAVHSYSEPQTHAFNTPFQLSIVPPSVAARMAAFGGTQLSDLPRDADVTSHRLRHGDVLVLATDGVLDNLFNQDVLRVASRVMGATGAWTVGDAGQIQVAENLDAIVRSPVKLAQSRGGNTRQQQHHQQSERAVTLQSLLATELVLAAKRASVNTKQDGPFAKEVQKYYPQENWHGGKIDDICVVAVVVVEDPVSTPSKL

>tr|A0A168BC98|OS=Ascosphaera apis ARSEF 7405

MDFLLLRPAHLAAATAAKTHSRRAAAAASAASASASVSLATSVTSRPSSLVSAQCATHVHLPLIHHQHQQRRSFRFTPSVRRAGNRNEDTDPRAAGAAAAAANGIGIETETETDAAGRQQHQQQKLQQSTPKTKQKKRMRYRIAAAASAKTSGVSLDKNVYPFLPHYHDAIGVQDISSMDPARRRRSRPDSGEDAFFVSKVGIGRGIDSALRHDRDGALLEGTFSSAVPRRSTDGDGNGDEDEGGAIAFGVADGVGGWANSGVDPADFSHALCTYMADSAMLWKDEPGRLTGRKLIQLGYEKSLRDRAIFAGGSTASLGVAWDNGELELTNLGDSGSMLFRNGAIHHYSPPQTHDFNTPYQLTIMPPRARSQSAIFGGRPFDDLPNAADVTTWDMQHGDVLLLATDGVFDNLFRNDLLRVARRS

>tr|A0A168I0D9|OS=Frankia sp. EI5c

MSLRLHFAVRSDVGHVREGNEDSAYAGVRLLAVADGMGGHAAGEVASSTVISRLAELDEYIEPPDLLGELSXAVRDANRRLRDMAIENNALDGMGTTVTAMLSSGVHLGLLHVGDSRAYLLRDGVLSQITHDHTLVQDLVDQGRITPEQANTHPQRSLLMRALDGREVEPDLSIRQAVAGDRYLLCTDGLSGVVSEETILEALLLEAPQDAVDRLVELALKGGGPDNITVVVADVLDDDQDDDAPLVAGAAAEKRVVPGAAAARSGPNPRNADPESAAAKAARIGRTGFHRRGSGDGGDDPDEDPERAAPRGGGGRRRLFVSTVVLVLVVASAAAGWTYVRGQYYVGVDDDRVVVFQGMKGSIVGLSLSTVRSRHQRLSEVAEPYRDRVRDGIPADDLADAQELAAQLPRPSETDDSDSEPEVSPSPSIGPELGKSGGSESPSSPALACPSPVANALPSCTSLAAPTSSP

>tr|A0A168JWG9|OS=Mucor lusitanicus CBS 277.49

MSPQEKPRTNLNVIDFFHPSLSSTLFHYTLNLGVSGYPKAGKPVEQTDDLNIYSSVQVGDDAYFKRQDALGVADGVGGWRSHKGANPALYSRKLMHYAQVELDRIKTNVRPQQLQAKPDPVQVLESAYHMTTLDAQNEGIVGSTTACIVILCQDELRIANLGDCGVSVIRRNDYIFRSEEQQHSFNFPYQLGTASFDSPNDAQRTPSQKQFTVKIEEGDIVILGSDGLFDNLYDDEILEEVQNCIDQQTTTHNNNNNTDGEKEDPSSILHQIAPQTISDALAWRAKIVSEDPDNPSSPFQVRAMHEGLYYQGGKADDISVIVAIVKKDEQPPEPSPPPLP

>tr|A0A176W658|OS=Marchantia polymorpha subsp. ruderalis

MAARRMHLRLSRLGRGGKADPRVARAVSRTARQWSKEGREVDPDMVEARCRGGHWATLARALGKSLPRPDAGAGRGLVRACRENMWRSGICGAMAGGRRDREPRPREADVALMCKLEFFTSRFPMCSCCNSEASSVMGFLDSFNARVPMSSCCSAGYPEEKKSNMALAPQMQGNVSPIGGARVNLGIATPSPARFPSAQFPTRPMSPGPAILSRPQSPGPSIKAILSPPGQTVVHQQRKELALAPGVKVIPHPEKVAKGGEDAYFTSNYKGGVLGVADGVSGWAAENVDPALFSKEFMAHAAAAVGTEDVNDDPRLLLAKAHRATCSIGAATAIVAILDERGTLHVANLGDCGLRLIRNGKVVYATAPQQHYFDCPYQFSSENAQTADDAAIYDLDLLEGDTIVMGSDGLFDNVYDSDIESTVKVFSGSDEDSANRLAMALATLASKHARDRNYNSPYAVEAVSQGHDVPWYSKIFGKKMTGGKLDDITVVVGHVVSMPVADKAEVKAEEVPSAQDEQGEKVEKVAETETFTGFQQPEAKGEQVGDLLTNGINAKTEEPLP

>tr|A0A177CPK8|OS=Paraphaeosphaeria sporulosa

MSSAITGAAGLPLRASRQAASARLFLLCVSTSSLLAPAHAHRSFSSQNTRSSTLYAPQTCPLNRDSSPATIGLRSFHSSSHNWQSTPQFTFHVASSYSAKQDRFSANRNIYTSDVYDPSKIEVTDLRQCKTAKARRSRAKSGQDAFFFSQVGTSKSTAFGVADGVGGWVESGIDPADFSHGLCEYMACAARTWTPRNKSSSTMHPQSLLQVGYDHVIDDDSIVGGGSTACLAVAEPDGHVEVANLGDSGFMHLGLNAVRHFTEPQTHGFNTPYQLSKTPQRMLVQMAVFGGPSTLSDLPKVASVTNHRVRHGDVLVFATDGVWDNLSPQDVLGIVSRNMVDIGGWVENDGAIEVGSELRALVQAGKESKADATSLQARLAIAIAKEAKETGLNQRRDGPFAKEVQKHFPGEAWHGGKPDDIAALIALVVEEPQE

>tr|A0A177R5U5|OS=Planctomyces sp. SCGC AG-212-M04

MMVGDIKCWAQEARLQKGVLESELGARFANAGGEAGRRYTGVDSYCYSSRRAPVLHDWKAILGLVLFYGLTLVLIRWVILTRRRQPASTVAWILAIVLIPYVGGLLFLFFGINRVERRKMRRRAAKKSMQGKMPMLKVLSLEDEPLKSFPQQVHRMVRLAARLEDTNVTGDNEIEVFNDTNIILRRIEEAILAAKHSIHLDCFGQTDRGLKRPTNEDHFLIADLNKALRIHQTSLNFDDEETIFGASQGMMLLVADGMGGHAAGEHASRLTVESLTSSILNHMPWFFSRDAEGDEDLRTALERALHRCQERLQTEAKAHPSVQGMGTTLTAAYVHWPRTTVIHAGDSRCYLLRAGKLRQITRDHTLAQQIADEGEFRSSNILHTRWANVLWNAIGSGTDDVKPEVHQFDLQLGDVLLLCTDGLPKHVEPQEIRDALERNLDARATCQL

>tr|A0A182FQM5|OS=Anopheles albimanus

MRSWMSRWLTRALHQSVQSTSSTASAESGPRTYSRFISVVSGFPKNLGQSKYKPGKMGDDAWFIANTKTADVLGVADGVGGWRSYGIDPGQFAEVLMRNCERLVKFARFDPIKPVNLIASGYQELRAHRESILGSSTACIVVFNREDSSIYTANIGDSGFIIVRKGEIVHRSEEQQHYFNTPFQLSLPPTGHTDVLCDRPESANTTTFPVCNGDVILVATDGVFDNVPIKLLVDTLHRVEGEHDQVKLQMCANSIALMARSLSFDSKFLSPFSINARRNNINAMGGKPDDITVVLATVAL

>tr|A0A182GSJ3|OS=Aedes albopictus

MNWAARLWSRTLRSSFSTLIEANIEKPYHLVSVACGFPKNILRSKYKPGKFGDDAWFITSTKKADIIGVADGVGGWRSYGIDPGEFASFLMKTCERLVQCAHFNPTSPVSLLSHSYCELLENKRSISGSSTACVLILNRENSTLYTANIGDSGFIVVRGGQIVHRSEEQQHYFNTPFQLSLPPPGLDNVLSDRPESADTMNFPVEKGDIILVATDGVFDNVPMKLLVDTLSEVRTE

>tr|A0A194SCR7|OS=Rhodotorula graminis (strain WP1)

MHHCSYELARYENVDDDMFLRYYEVDPVDVMQRAYEKTLGECKEEGTIGSSTALLAVLRNDELRLANLGDCSCCVIRGDEYIFRSEEQQHRFNYPFQAGTNAKDVPARDAQKFTVKVQRDDIVILASDGLVDNVFSDDLLEEVLRFVGSSHATSSSSSSAPLPPPPSSSSSSSSSSSSSSTAPRYTLRRFSPQAVSEALCFRAKSVYEDQRAVASPFQQRAMDEGIHFAGGKPDDVSCLVGVVGELEAAPNRRDVQ

>tr|A0A199UQB2|OS=Ananas comosus

CGNTISFFSCNCRSELSFSVGTHLIPHPKKAETGGEDAFFVSSFNGGVLAIADGVSGWAEQNVNPALFPRELMANASDLVMDEEVSYNPQILLKKAHAATSSIGSATVIIAMLEKSGTLKIANVGDCGLRILRKGQVVFSTAPQEHFFDCPYQLSSELIGQTYRDATLCSVELTEGDTIVMGSDGLFDNVFDHEIVSVISKIQNAVEAAKALAELASNHSVDTTFDSPYALEARNMEQAMQVGRRK

>tr|A0A1A0H257|OS=Metschnikowia bicuspidata var. bicuspidata NRRL YB-4993

MAAKNGGLRPLSATATNTLGASKHTPAAKPAGAPEPQTAANGGLLRTLKSVFGLAKLPEQQRLEAREQRENANSPFIQYPLVTREQMRRMASHAYRLDVGHASFAHHSFSADPTVHSLSDLTDPAQLNSLLPRRRPQGSPVDTLLIKAGDDAMLVLPTVMAIADGVSGWESKGEQASLGIWARSMVETLSRLLTEYRLSHMPHVLNARDVQQVLDDSFLHTSHLMDLQGLKGSSTLLLGMLSGATLKYISIGDSRLWVVRGGRVIRSNTEQMAAPLCPQQIGTQTLGRMPSEMAEVDEVALQENDLIVMCSDGISDNLFDHELVDALHQYLAPPNSSVRVACSKILAKCKLVAYDDNAYTPYNEKVNAPPGQAAKKASLGGKMDDMSICIARVVANESAAK

>tr|A0A1B8DEU7|OS=Pseudogymnoascus sp. 24MN13

MDIAARRISANTLVTAASKRIALAEYAIPTLCAQRSTGRRNLTAPSLRTKAPSQRQHSLLTHITKRQEYSSSSATSPPPRFSYGIAASFTAKDKRFKPKDNVLHFEPRPIPVKRRTNRKDRPASGQDAFFVSQLGDSGDVAMGVADGVGGWADSGVDPADFSHAFCDYMAYEANNYDTESGDALSAMGLMQEGYDSVVNDKTIRAGGSTACVAIARTDGSLDVANLGDSGFLQLRLNAVHYNSEPQTHAFNTPYQLAIIPRSMRMMTQAFGGTQLDDMPKDSAVSKHSLRHGDVLVFATDGVWDNLNSYDILRLVSKLMVGSNAWTHSDDGINVTDRLSDYILKDDSPGSDHVKSLQSFLAMGIASAAKAASINRRVDGPFAKEVQKHYPHEMWTGGKQDFEILERPEPPTFIDQLLQLASQATFKTENVQQSIKTSGAGARHDTSTRQNSTSSPAASKAPLASHSPISTEAIASATEIKAAQPAAPSTPYAVTESVKELLPLLNAQPSKFITALIHGRPYLLTVGDTVRLPFHMPGVVPGDVLRLNRASAIGSRDYTLKGQPHVDERVFECRAIVMGTEGEPARIKIKKKQRNRRTKTVISKHKFTVLKLSELRVKDLAEIEQ

>tr|A0A1B8W0E4|OS=Bacillus sp. FJAT-26390

MVKKWINRGLAIVLLLLVIVFIGMKAIPVEQLDQLWAKDQIEFIERMIIGGMIGFGALLAVFVFDLPAGRRRTSDQRDAALQAQSRTKPKVELAIAPTITPPSEPPPIPPIPPSIKQTIEPLMTAAPNPPTPPAASNREEAAPFTVITGYAQHIGEREEQQDAFGFSPLAQPFGSKQNGVFAVLADGMGGYAMGKEAGELAVQTMLSEHMGTAASACSIPQALEHSLHMANKAVYELALEHGLEWSVGTTLIAVDVREQQLHWISAGDSRIYLYRGGVLIPLTRDHVYANRLNEQVKAGKLTQEEADSHPERHLLTSYLGIPRITEIDANQAPLQLMAGDFILLVSDGLYDDLSEPLLQEAVRHDPQQAAAFILEHVLAQQRPYQDNATIMVIACS

>tr|A0A1B9IC14|OS=Kwoniella pini CBS 10737

MSKPKPGLGKGLVRTFATSVSQYPSHHLQGIITLAPVIGQQHYHSISASSSSHNTLGSPSKNGVNGWPTSSSSTITLLPTWDQPSEIVLPYHSSTSSSSKSRIRLKSSSASSSSSIIGNNQITSRRYSSISISPSAFLTSPIPPPHSLFFDKSLSYQSNVTPPSPAQISLETLLSALEPTSLINEFGFNSRNNSFSDLPPPEPPSSPSPLPSASRSSSPNNPPIKGLRSVESHTVLSPNPLTPDNDSSTNQSTLFSSNSLLSLSDTSNHGTASSLKEDSEDISSSTYSSSLIFHLGCSGLPKERIPLPPSKNIRRTPQPPRARSFPLPIVESPSHLKSIGVGEDAYFARTDGLCIADGVGGWSRSASASKGSADAGRWSRLLTHFVEEEVADWWNGKEYYLISKEKGASKDEKSQYDSSLKKKNQSQGWARESWENKLNRNMSSSALTSSSEKFNRESERRPIDPVEIMQRGFEKCLSCINAEGIHGSSTCLLALLHDSTLHIANLGDCCLLLIRKGEVVFRTQEMQHAFNFPLQVGTHSRDEPMKDAQRYDVSIKKGDVVILGSDGLMDNLFDEEILEIVLQFTSDSITSDQSPTTPSLWNKEPSLPFSPQQVSEALCKKARSISELVTATTPFMCKAIEEGIDFVGGKKDDISVLVGVIGDKEEVGQEGEEKTQGGLQLHL

>tr|A0A1C3PGU7|OS=Candidatus Protofrankia californiensis

MPPLVGPPPPIIPTPDSIRSRTAGGRAGAGGHGRGADLPRGAPARSAPGSPGGAGADEPTGTQVGGYSDGRAGTVGSHHGHDALRDDHTVPAEGGRTAPPVPPAPPGPTGEQLVADEPDVSTSAALRLAAAGRTRRGKRGGPNEDAFVVVDGLLAVADGVGGEAAGQIASTLAVTTVASFRPQYAADPRDGLRGALERANRTVREKPREEPSWHGMACTLDVVVLGRQRDTGKTLFVAHVGDSSVWLQPGRGRPRRLTTPHAIKNGPLLNAIGLNEEVELDLLEEPVRAGDRVVLASDGITKVMTPEQLDGLMMELGSLSPERTADALVDAAMMAGARDDTTIVVADLVADASPR

>tr|A0A1C9W9F9|OS=Microbulbifer aggregans

MSEANVRERSRHSGTIQRVSPPSRSAGATHPGYKREQNEDALWSDEDRGVWVVADGLGGHQAGEIASMTVIEEVQRSAATDRHYERALQRAHALLLGEEQNTANMGSTAVVMAEDGAYFHIYWVGDSRAYVFTPSAGGGELKQLTIDHSYVQMLVDSGAINAEEAANHPNRHVITRCIGGSTNPQLEIDRVSALWQPGQRLLLCSDGLSAEVPAEEICRILAENPDNRRAVDLLIAAALDAGGKDNITVQVVEAPTSPADGREWPFGAAHDGEGTRSSLLPGGTMTLAFSAVILLALLATLTAWLLDSL

>tr|A0A1D1YFP3|OS=Anthurium amnicola

MAPPRPLRPHWSSSPPLVLSLRFPLYPLPMGGRIPVLTPASSGAALVLLLLTTGWVVVSAACSDGSVVFRFGVATNKNEAGAGGGMGAEAVEVIPEPFDGGNGASWGSAGGLRSGSDVERLQITVEEKGLGMAMCEAPVVEAEEGVDGEAGSSVLAKEGARSAGVLSVDHDQIMRSVNAVESSCRLQEKEDSLGKMEKLESVVDGESEMDGTESGNGWYMLEEKRDSDSVLGKLELEGGDGAAGDVVVNEAEEPLVGGGGSFLNKVNLEQEVESNHGSDMIAAGVGEIVQEGGSPASLREPLAPQDDDHGEETVTARPDVAGVQDGDQEAHSVSQDDKELLQDEEMSNFDMERLKKLELQDVKMNPVTVETTDHTDQLLVAENNELKGQGFDVHNSGSALGTEDNRSTAVPIEDMTVSTPIYVLSSGAAMLPHPSKALTGGEDAYFVASKNWLGVADGVGQWSLEGINAGLFARELMENCAKLVSNCQGVSAAKPAQILSQSAMDSQSPGSSTVLVALFDGQVLHVANIGDSGFIIIRNGMVLQRSTPMVYGFNFPLQIERGDDPSKLIEVYSVDLEEGDIVIMATDGLFDNLYEQEIVAIVSKSFQAHLKPTEVAELLAMRAQEIGRSPSTRSPFADAAHAAGYPGFTGGKLDDVTVIVSIVQSL

>tr|A0A1D2JPQ8|OS=Paracoccidioides brasiliensis

MDRVIVARASRLRGLAQMRLWNVVGGTNFSRAPCTSAVTRSRGVGADRSFHSSPSLMAEYRVSYRIAASASGKGHKLSPTKNVINFNPGKTDAIGLHKGVTAAAWKRSRFDSGEDAFFVSKIDNETNSVAFGVADGVGGWAEYGVDPADFSHALCSNMAQVALDWDRKFDKLRARTLMQAGYERCKADPTIFAGGSTACVGVAHQDGKVELANLGDSGSIVCRLAAIHHYSVPQTHNFNTPYQLTLVPPLMRLQSSIFGGRVFEDFPYHANVTNLKMQHGDVLILATDGVLDNLFNQDILNIVTNQMISTGAWNGSSDSGISVSAELDKLTHVGGLVPSLEISLSPNNHIPNRQMPYAQEQLHTLQSLLAFSIVRQAKVASVDHHRDGPFAKQAQRYRTLDKFRGGKVDDICVVIVVAVEEGRAGP

>tr|A0A1D2NKP3|OS=Orchesella cincta

MSCATWLGRVVTRIFVAGLHNSLGEQIFASEIARGHRHLSSSSSSSETSTNNNNEEQLGELMKKATEKEVARISLASQMCRALHSTWHDFHLGAGFVSVVSGLPKDFASSHFRRGQFGDDAWFIARNTSADVIGVADGVGGWRNHGVDPGEFSFSLMRACEKLVENDLFSPTKPERLLANGFSRIREANRVTGSSTACVLVLSRSDAMLYSANLGDSGFVVVRDGIVVHRSKEQTHCFNTPFQLSCLPPGQQGLSDSPESADISQIPVKDGDVILLATDGVFDNLPDYLIVSELSKIQGQKDPLQLQSAANAIALMARTLAFDSKYMSPFARNARLYGYHTVGGKPDDITVLLATVSIER

>tr|A0A1D6HE41|OS=Zea mays

MTIALETLQLQQIQQTLAEIKERTPDVNISRFVASVLGDWMETEYATSERKRGDAGHGRGAGPSDARPCAPTLEMDWAACVLPLHGEDAHFGHAEAGVVGVADGVGGYRDNGVDAGAFARALMANALASAERVAKASRRLRRLCPEKVLERAHKKAAADETPGASTAVILALHGTALTWAYIGDSAFAVLRGGKIICRSVQQQRRFNYPYQLSSEGGGLDDAKVGSMPAARDGDVVVVGTDGLFDNVHDWQLERAVRMGTNLGFSPKNMADIIAGIAYGISKDKWACTPFGMGYMKITAPSVPSYVELFGSAPALPKAMPSSSLNNIFDSFKQPASTLSLPRSKHSSMPVFDNPVYDDNIFIEVSRELDLSTFIRIWI

>tr|A0A1E3NZH6|OS=Wickerhamomyces anomalus (strain ATCC 58044 / CBS 1984 / NCYC 433 / NRRL Y-366-8)

QSPSSINSSKKKDSPDHFKFKYSFASYVHHGGSSKPLISSLMDLTDSSNNLSLLPRRRLYGNPIETLSIKNGDDAMIVSPNLVGVADGVSGWSGAHANSGLFARSFLENISRNFSELSFKNSDNLNNIKEEDLSKNLDDAYKDSIQIMKSEDFKGSSTLLIGMIIDKTLKIMNIGDSKLFVIRDGEIVQSNSEQYIANLCPEQVGTTKTDKLPSSVVQFYDFELQQDDLILVCSDGVTDNLYNDEILEIINKKLNKEKSNLQEVSNSLLYKTKSTAFDNYCVCPYVEKVNELSNQFITGGKIDDISICISKVLLN

>tr|M6ALQ2|OS=Leptospira sp. P2653

MIINYFGITEKGNFRSHNEDSMYASGEIVAGNVSGSFSSSGIRDSAATPLILALADGMGGHISGEVASRMTLEKLAWTERAVQPLEELPRAGWQSLFSIINHEINDHAKATGKLGMGATLVGILFGKRKVLVFNMGDSRAYHFSSKGIHKITVDHSFAGTIKGNQISRSYITSCIGGGTTDLQMDLFDITSSLNTGDRILLCTDGLTDVIKIDDLEEILKNSSNVKEACYHLLEEANLRMTKDNTSIIVIEVQGMMFARSEPRKLTPSEKKL

>tr|M9YQ02|OS=Azotobacter vinelandii CA6

MSISTSFDSALGYAALTVAGQVRKCNEDALLCCPQLCLWAVADGMGGHGRGELASALALETLHRLVACGQDLRSAVQGADAAVLAAAETRVGGYGMGASLVAVRFAGADFQLAWAGDSRAYRIGGDSIRQLTRDHTWVQTMVDAGRLTQEEASRHPLRNLVTRCLGLNHDMLAVELVQGRLDQGERLLLCSDGLTRELSDARIQHLCAHTLSLDTLVGALVGAAERMGGSDNISCIALGLAAPPARTEERPRGFLRRLLKSAGKMSGARLRRKIDARSAPGALARMAAYRPVA

>tr|N0CQJ8|OS=Streptomyces microflavus DSM 40593

MKMSESHQQPALARCPGCEEPPASGDLFCGACGYDLSAVPARPDDRPTMAITVPPAAPAAPAVSPTGPPAPAVPPPAAPPVPDAPAPPVAAVQWPAASETDSSDVPAPVHRATDLPGTDSGGKPLPTTPPVRHDDRAAAVPAATEPAAEPAEPPYSPDSSNSPDSSGDFALAAPDPRTAEPTPTPAAGTKVCVACRSGRVDPDGYCENCGHAQPRERDHMEQELGSVAAVSDRGLRHHRNEDSFAVSSTALPDGSPAVVAIVCDGVSSASRPDEASAAAAVAANEALLESLPRGTHPQQAMHEAIVAASEAVNVLAQDPPGAAEPEAHRHQNAPACTLVGAIMAGGLLVVGWVGDSRVYWVPEDRTGPPARLTEDDSWAAQMVAAGLMNEAEAYADERAHAITGWLGADSYELEPHTASFKPDRPGLVVVCTDGLWNYAESAEEMAAAVPPEAHLRPLHGAQVLVGHALDGGGHDNVTVALLPFTVEQQGAGSACTTV

>tr|N2AJA5|OS=Eubacterium plexicaudatum ASF492

MLGKKKTNKNTGYTQNSKKAQRFAKKEKLDVWNGPLDETYAQLGELCEQPQLPDSPQSTEPEVRTAELTESKVATKQLAGQKHVAQQRAQNRSQIEIGTASIIGTRKSQQDSVFGYESGGRAIGIVCDGMGGLSGGEVASRVALQSIADAWFAQTDVLNIPDFFRREAVCADEKVYLQEAADGKRLQAGTTVVAAIVQRNELYWLSVGDSKLYFIRGQEILSLNVEHNYRLELNKMLRQGKMTAQQYAAEEYRAEALTSYIGIGNLSLMDINKQPYLLHDGDIILLASDGLYRSLNEEEIISIINKNRQEMQKAAQALTAAVEGRKKQDNTSVVILRYRTVSSAYRK

>tr|N6VR70|OS=Methanocaldococcus villosus KIN24-T80

MKVFGITHRGGRDKNEDHILIKKIGDIYLLAVADGLGGHNAGDIASKIAIEELEKFFEKNCYKSLLYEEIETILKEAFKKVHNAILNQSYGDREEMGTTLTSVVIKDNKAIIANCGDSRAYIIRDGKIIFKTKDHTYVQELIDKGYLSEEEAMYHPYKHVLKHALGIDFAVDIYKKELKSGDILLLSSDGLHDYVREKEILEVINKYEEPKDIVENLLKIALKKTMDNVSIIVFKVK

>tr|N6ZYJ5|OS=Thauera phenylacetica B4P

MKDLTPLEIHLGHATRPGPRPANEDFVAAAMPEGAERAAKGVLLAIADGVGGHAHGREAAEYTVRSLLADYFSTAHTWSVEKSLDTVLGAANRWLLAQSARSAETAGMATTLTALVLRGRRWHLAHVGDSRAYLWRDGTLLRLSEDHTWPHPELNNVLRRAVGLESRLLVDHDDGELAAGDVFVLLTDGVWNALGEVEIAALLARHPGAEKARGGGGRAAVGLVTPEPVTGGAAARGSAVRGAVAQENRVGLSGEGRTEAPGTSEQGMATQGTATQGTATRENGVGLSEEGGTAARDAEAGLDPQAAADALVDAALRAGGNDNASALVARVRSLPADNLHDRLAAGRRLPLPPRLAEGDTLDGLRVEAVLHVSRLTVLYRVCRIEDDTAAFAPAGAPTLPRTASTSPQPASTSPQPASTSPQPASTSPQPASTSSRAPSTSPQPAPTSPRALSTPPQPAPVHVGAPAGANAASISPDHVAERTASFAAAGAPTSGQPASTSSGAASTSPRPASTSPRPAPTPPQPAPAPVGAPAGANTAARWVLKTLLPAHEHDEDARRALVREEWLARRVPAQGFPQVADWPARAHLYYLMSWHEGESLKALLARGERLRAHEVAELGARMLRLVGVLHRLGIVHRDIKPDNLHLGRDGVLRLLDLGVAASEAEDLREINNPGTPSYMAPELFAGEPASEASDLYACGVTLYELLTRKFPYGEVEPFQHPRFGEPVPPTRHRPDTPAWLEAVLLKACARTPDARFETAEEFRLALERGAWQPLAVPRRTPLLERRPTLALKLLALGSLLLNLVLLFLLTRS

>tr|N9FI05|OS=Acinetobacter beijerinckii CIP 110307

MSQVLHPEIQSAFTDLLTQLLQDKTQSPDLSENAEWISQLLKNKRIYNAYYDLAEEIYDASAFQCEQLPYFHQTQLYHILQQGRQAIYLPCPNNLSEFEQPVDSEIEIEKNTVHTDVETRQSDFNQKTELLSLVLDYQLESAVSHEQSLDPQMLATNLQVVLNSAPNKDAPNVSTDIIETVKKMPYFQIPNARVGQIYQAKIQMQHPMQQPVYICADRIEISDDLGLYFNQDMQQLQGTPLQAGEFKLNFQYKINQEQVDWQQGEVTFIVTPDPRSLWQINEPDANEPYQKSHSDSEYIQAENFKIAASSQRGRSHEHAGSFRDDDFLIRQIDNSDWAILIVADGAGSADFSRRGSQLAVQSMGQILLDDLKQRQAHLDQLIAGWQVAEVDQQLQALGQQLQDLFYQAALTAVEAIEHEAHEQQVDVKKFSTTLLAAIVKQQPERTLISSFWIGDGAIAVYSPDKIRLMGKPDGGEFAGQTRFLSRQVVNQFSAHVNIGYFQDCQAILLMTDGISDPRFETDAGLNNLQKWQQLWQEIQPKLQHEKPDQALLEWSKFFSAGHHDDRTLAVLWQDSLQKKVDENDDQKSSELNVHDKPLRG

>tr|Q0ABA8|OS=Alkalilimnicola ehrlichii (strain ATCC BAA-1101 / DSM 17681 / MLHE-1)

MQAGGFTEPGLGRPHNEDHFGYDLPAGVAVLADGAGNRPHGEVAASLAVETVLAIARNHHGADHTWLESGGEPRKLAQLANQALLAHTESNRRHRGMGSTLALLCVAPAAVSLALVGDSPAYRLRDGQMTALGPGDAADQALGEQPRVQPDLHDLDRAAGDLFLLCSDGVSSALDDEALCALLMATQNDLAASARQIVLEARQAGGEGDASALLVRLD

>tr|Q0RIX6|OS=Frankia alni (strain DSM 45986 / CECT 9034 / ACN14a)

MSDRGLVHRTNEDGFALRVLAGPGDDDRPAATLAAVCDGVSTAPGSGPAAVRAARDAVDLLAQLAGTLTVPPATPPHPGDATDTSDPRLSGNAPSDAPSDALGDALGDAPGGAGGVGAGGDTRPLGPRPGSASPGGDTARWQASALRAAAATAQRSALASISGPDDAPACTFVAAVVTPDRLSVGWLGDSRAYLIDRSGARLLTADDTLAAEAVRAGLLPPERAETGPGAHTITHWLGVGSASAVPRVAVTALNGPGRVVLCSDGLWNYLSAAGAVAERIAELPAEAPALAVARHLTTVALARGGGDNITVIVIDIPGSSDDHLHL

>tr|Q1GXA3|OS=Methylobacillus flagellatus (strain KT / ATCC 51484 / DSM 6875)

MKFTIHQSSRSGGRLVNQDRVAYSYSKNALLLVLADGMGGHQRGEVAAQLAVTMLTSAFQRAAQPELPDVVAFLETEIMKLHEAIHQLAKNRELPETPKTTLVVGILQHQTLYCAHVGDSRLYHFRNGKRLFRTEDHSVVQLMLQQGKLDAKSMLQHPDRNKIYNCLGSELLPTIDIAPPSPLKAGDQILLCSDGLWAQLSDERMQDILNNGLGVAHSVPSLLDAAERLGGKDMDNLSAIGVQWETKAEQPLTISTLDMSLAQSHTILGLAAPAASSSAARAADLDEEDIERAIAEINAAIKRSKY

>tr|Q1IB08|OS=Pseudomonas entomophila (strain L48)

MHFDLIQTLSLAGKPDVPNDDRIGCADRHAWVIDGATDLGAPGLLGERGGAAWLANAAQRAFSAASGPLQTLCETVFDTLAGDYRQDRRRDPVAHWELPRAAFAAVALEGDELVCAHLADCVVLHRSARGVAFLTPEPDREAERGEAAALGPGTGAHNVRTPAVLADRRQARERPRAVLGVDAELSREGTCYSRAPVARGDDIVLMSDGFAALFDTYQAYAAATFVERLLSHGLIDLARTLREIEQEDAACLSHPRFKMSDDASAIWLRVS

>tr|Q28RY9|OS=Jannaschia sp. (strain CCS1)

MSPAAPIGYDVATALWQGARPYQEDTLLADFHGGMDRGFAVLADGMGGHAAGDLASRLVVIDAVSHLKFLMHDGAALETSLVSELTSAIETANNVLKDRAAEDRRLRGMGATFLATVVFEDRLYWASVGDSPLYLWREGTLRQLNEDHSMAPVIDQMARAGEITPEQAASHPDRNALTSVLMGEPLKAMDVPKTATVLDPGDVLIQASDGLQYLDDKKISEVVARGGNSREIADALLAALRDLDDPVQDNTAILVLQLMDRVGEGAVSATAGSATAKSTVGSPSAPADVPSTATATGDTLSVGARRWALPAGLLAGAALVAGLVFGLPGGPPEPTSVAELETAGGTADAAPEADQAPAPALADAPVPATTAVSSATPTPDAVPEADVTQVHETIGETTALAEEGPEESVPAVVGLKPSQPAATEAARAANGATILFETTTPGTPAAPGANAVGLDVLRPQDAPPLPNQDAVEAQPESQRTRIPESPLPKSRPSATAKPTTSEDARNG

>tr|Q2FNC7|OS=Methanospirillum hungatei JF-1 (strain ATCC 27890 / DSM 864 / NBRC 100397 / JF-1)

MSAGHARPGSYFACGASVAGTRHIRDQIPCEDAWAGVILPGALIIAVADGLSSAEHGGKGADIAVSSSVKNAADGYKAGEPDIAALIRSAMSAGREDIDDHALSEGLDISSFATTLLLAFLTPDGAFCGHIGDGACVTLSEGEPSLLSVPGTAEYANETAVLTAQNWESQCRISHRAADAIICATDGCQGALIRREDGAYIPYSPFVVPLVRSLGQYIHEGRDLNTEVADLLSSSRMRALSSDDMTLAVGFSLSGEPF

>tr|Q2J548|OS=Frankia casuarinae (strain DSM 45818 / CECT 9043 / HFP020203 / CcI3)

MCDDQLDECDHFGRSSGARRRCPETATASIDDPIVRGRRHPAHGSAHTARMRSPGCFRPRPISPGGMTVDLNPHDDAPTVAYQITGSGPFELDDDLGRCPVCAAPVYADDRYCEVCGHGLEGPHPGHGDADHSEVDLGQLAGVCDRGVRHTTNEDAMGLAVVYGTLIAVVCDGVSTTPGSGQASAAAAAAAMAVLADAVRAHGPGRPVHHGTRPSRGVSRAEEMLDILEPSYADYKKPRTSPRAVVGGFSPEDAEAALHAAVDAAQATIAQLSAAEGRMAPSCTFAAAIVTPPTPDGPGMVTVGWVGDSRVYLLGPRWCERLTADDTWAAEAARAGLIPANEAETHRRAHTLTRWLGGDVEDVAPHTEMFPIEAPATVLVCSDGLWNYASRPDVMAALVNQLPPHCEAIDVARHLVDFAINSGGHDNITVVAARVED

>tr|Q2LEX9|OS=Streptomyces sp. 44030

MFFSSHTSPGTGKAGSENEDWIAATSDLVVVLDGATIRTDTGCSHGVSWYTRKLGANILDGAASRSRSLKEVLADAIEAVANLHSETCDLSHPGTPSAAVGIARLDGDSLQYLVLGDVSLVLEMADLEIRRISDDRVSETGKKEREIADKFAIGTQEKHDAMLEMKRAELAARNVKGGYWIAATDPSAVDEAIVGSVSLRNVVRFATMTDGAARIVDLFKSRSWLRVLNTLEQDGPSFLLADVRHLEDSDPVGENFPRNKTSDDASVVFVDPHRKPTRWKGSRPGRTINSEVDFDSSEVQAAREKTLAEFRAMTQNDPAAIMGENPEIWKRNNPELAEQYRGKRLDAARRLVAEVDTPTR

>tr|Q479A3|OS=Dechloromonas aromatica (strain RCB)

MRFTIYQESRQGGRSNNEDRTTYCYSRDALLMVVADGMGGHHYGEIAAQIAVQTLADAFQREARPLLNDPFRFLQKGMTNAHHAILDYTARHRLKDTPRTTCVACVIQDNVAYWAHAGDSRLFMMRDGRVIAQTKDHSRIRLLVEEGMITEAQAAFHPDRNKIYSCLGSPTPPEIEFSRKTPLDHGDILLLCSDGLWGEMSGDMMAVALKGTNLLQAVPMVLNQAELKGGAHGDNLSVVAVRWEDTYVEEASSAISTQTMSQDEVTTRLEEFGRNPAYKSELSDDEIEDAIEEIRSAIEKYSPPKK

>tr|Q5H422|OS=Xanthomonas oryzae pv. oryzae (strain KACC10331 / KXO85)

MRGFLRHLAERGGFEPPRRYKRLPDFESGTFNRSATSPDGPQCPGDVHHTVTRPQPQAARAGAAKAAIRPGVNRHCDAHAWCVAAGRSGRDDGGASSPWRPGPMLEFGHLTHVGLRRDLNEDTYYGDSELGLWLVADGMGGHACGEVASALARETIVREIRAGTPLAQSVRIADEEIIKTSRRCNDTLPMGTTVVAARVLGQRFEVAWVGDSRAYLWRDGRLAQLSQDHSYVQELIAQGTLTSEQARAHPHRNVVTQALGVTDPAHLNVATMQGELKSGMQLLLCSDGLTEEVDDAAIAATLSQADCSAQEYVECLVAAALDGGGSDNITAILVRSY

>tr|Q6A844|OS=Cutibacterium acnes (strain DSM 16379 / KPA171202)

MNKSDQSYTVTPDTAATLRVDLTDAAAARGDVACASHIGLRHETNQDAAALGIDGSGHHIVLVVADGVSSTEGAEECARVASHTARDYLTATMDQGLPINDDDTVTLFERTFQKTHEAVVSGSGPIGACTLAVAVATHDRIVVGNIGDTRTYWFPDDGDPVRLSIDDSMAQAQMDLGLSREEAERGMGAHAITKWIGASATDVAPRVMAYQPQQSGWLLVCSDGLWNTYRMPAIWPGLWPTSSAKRTLMITATLLRQA

>tr|Q6LUE0|OS=Photobacterium profundum (strain SS9)

MQSRYTRVTFSQSIFSIIRRSLEKVGMVMKHANWSFFSFSQTHPGKVRAYNEDACLALQKEGVWVVADGMGGHEGGDIASRILVDTVEQAVVRLGKEYINPDRLREALLDANERIFQYGQHNLSESTIGTTAIVLLIENGNFHCLWVGDSRFYLYRDQVLIQKSKDHSQVMEMVEQGLIGARDAEDHPMANVITRAVGVDRYLMIDQLSGSILPNDQFLLCSDGLSRELTLQDMNACFQAQSVNDVGLALMHSALVRGASDNVTCVVVKASQQQAIAAQTRHQYLDATVPVFTHQRAIRGSE

>tr|Q733J1|OS=Bacillus cereus (strain ATCC 10987 / NRS 248)

MKIKTYQQKSPLKQECEDSYFCNEENKIYGVCDGATPLVPFRDEQGHNGAYIASHLFASYFASLRENHSLPVAVAKANEALQRKMLEYKVDTRKKEHLWCTCIAAVQIGGEKIEYAQLGDCMIVAILRNGTIQVLTKDTVEGISKRAKKKREEDRKQGLSVLEEHVFQDVREQLKYNRYLANMQGGYSVANGMKEAIHYLQHGELHIDEVSGIFICSDGLFHPDWSLEQAVAYIRKNSISEYVAIIEKLEGEKRIRPDDKTMMMIDF

>sp|O34779|OS=Bacillus subtilis (strain 168)

MLTALKTDTGKIRQHNEDDAGIFKGKDEFILAVVADGMGGHLAGDVASKMAVKAMGEKWNEAETIPTAPSECEKWLIEQILSVNSKIYDHAQAHEECQGMGTTIVCALFTGKTVSVAHIGDSRCYLLQDDDFVQVTEDHSLVNELVRTGEISREDAEHHPRKNVLTKALGTDQLVSIDTRSFDIEPGDKLLLCSDGLTNKVEGTELKDILQSDSAPQEKVNLLVDKANQNGGEDNITAVLLELALQVEEGEDQC

>tr|R4LKK1|OS=Actinoplanes sp. (strain N902-109)

MFKLLKESWDWFIGAAPQDVPARAPEPERYPRHQPAHREPPGPGPEPVRTGGGTVYIPGRRQPEMAEMTAPDGGGAGRASTAVAAAATPVPVPVIAPEPVPRPAPEPAEPDWGPPVLERPGPVFEPRPSTATSYRPDVIADGWATDHFVVRLASVRGYDHRYSGAPRQDDVAVAHHARTGAVMFAVADGVSAAPLSHLGATAACRAAISAIGAGLDSPHRRVDWQELVQLAAWQICEQARLALGLPEVDRQAADEQMATTLVAGLVLPTQEGPEVHLVQVGDSSAWRLRGDDYYCLLDAKYNPDAAVFSSAVSALPRVPRVQPSVGPLSPRDVLLVGTDGFGDPLGSGRGAVGAHFARSLAEVPAVLKFAHDLDFSRETFDDDRTLLAIWPRGEG

>tr|R5AL49|OS=Prevotella sp. CAG:1031

MEEITTSYQAVGYAESRIGGRSENQDTCAFTDTPVGLLVLVCDGMGGGPGGKTASMIAAAVITERMKKVKRVEDAEVKLTAAIAAANAAIMEATGQESPLAAKFPDKKFLPEGMKIRPELKGMGSTVAALLLTKDYALIAHVGDSRVYQLRGRKVVHRTTDHSRVMLRVLRGDISEEEARTSSDSNIITQALGHSGANMHSDVTKVPYLKGDRFVLCSDGVWGAFPQPELVAMLTSNRNVGGTVDNTVISVDDAGRQAGGTHDNLTLALIETTTNSTLPVKMNKTTKLMVIALAVIAAASIILNVILWCARPKSETTEPVNPTVENVKPVVPERPETQTNPKAEPQTEVGQSEKDDATSELNARVDDLTRQLDQLIEEVEGLKKVTDKDAKTSMINSIKAQMKGILPMVDDNPAVKELLNKAFNELDAKIMFIPSRCDGQINAISKMLRTANDQLKSKN

>tr|R5B534|OS=Clostridium sp. CAG:226

MRLAMKGATAKNTRYADKPNEDLFFFDEATGFAMILDGVSRDRENGIYPNPSPACRADNAFADAAQAVLLKKNGAAPADRLKAAVYAGNMAVAAANEGFPSPFLPGTVGVLALFAEHKLHYAYIGDSNGILISNGTLSYFTTPQTAEVHRRRKEFTSDEIRAVICNNPSHPCGYGVWNGMPSAAEFLRVGELPLSAGDRVLLCTDGIDPFLASLSDAALAAMDADTLISQAMAYMMPEGYMDDRTAIVINILDV

>tr|R5EBR4|OS=Firmicutes bacterium CAG:110

MIKRRQSLLAVMAVALILLALVTGFLGFAHKSDDLIAAPSLPQETLPQEILPWDTAPQETLPQETPSQETLPQETQPLETLPQETPSQQTLPQNTPPQETLPQNTPPHETQPQETLPTKVPILPPDAAAPSQAGVIIPIPGETENSAAEQTPLTAFRLAMRIACFTFTAGACVGILLLAMLRKNVREHERANEMPLNPPKPVAVHPVPITKETVEETVCPIPGISLGKIHDIGRRDYQQDSFGQTAVLRNTGILAVLADGMGGLSGGERVSQKIVMEALTFGSTLQANQVPTALPGMVAGINRAVNQMLGPKGLYTSGSTVVSALITGNALRWISVGDSRVYLYRDGQLSQLSRDHDLLQDWMPDILEGKRSMAEALRDPNGRKLTSFIGMGELRHVDYNRTPILLLPGDRVLLMSDGVYGTVSDAEMAAILRDCGSVQLAASHIGQRIMGAALPYQDNYTLIVLGYDPPDQPRNNR

>tr|R5XWM4|OS=Ruminococcus sp. CAG:488

MENDMILIASCVSYIGAGRANNEDNFYFNKKRLAEKNNGMTAPISQQTGLKNPVGFAVFDGIGGASYGEKASYLAAEIFAENMDRLNDLIIPEREFLVEICNQANEKIVSLAHNKQISQTGTTLVSFLFSENGVFTCNVGDSKAFLIRKNKIMQVSKDHTDKDFLDSIGINKKPSLLQYIGISDKEGELDPFISKGQVMKGDVFILCSDGVTDVISANDLYNTIKENSDVSSGVNAILDMIKQRCGQDNATIIVIKVS

>tr|R6AAA8|OS=Prevotella sp. CAG:5226

MKYQIKAYNLQELGQRANQEDSLFPALGQSTTDCRLFVLCDGMGGHEKGEVASATVCEQISRTILSQWHAGEPLSDDLFRQALAAAYDALDAKDDGAERKMGTTMTFLCLHANGATVAHIGDSRVYQLRPATKHSPARIVFHTRDHSLVNDLVKIGEITEEEALHHPQKNVITRAMQPCQEQRARADIAHLTDIKPGDYFYMCSDGMLEQSTDENILNIITKPNSTDEEKLEMLRSVTEENKDNHTAHLIHIEKIIGANPLQAPVPPRQQEPKGTFVIPDFPATTPRPKRRNLLPWLLVVLLLVIIAVGASYFILGNKENESAKPDSTKMDSAKGRSAEKKNSDALIEDTSLQSVSPQRETSLPTSAERQSQGVGSKPVKPTKQEKPQRVADDKQEAKEDEHIVSEIFKSRKGSPKEEKQTPKFNVKNKYVREKE

>tr|R6CFV3|OS=Bacteroides sp. CAG:530

MKFTINTPLAIHELGKRQNQEDAIYPVIGNATADDRLFLVCDGMGGHESGEVASNTVCQAMSKFINENANGDTFSDTLLKQAIDYAYNALDEANIGHDSVKTMGTTMTLLKLHEGGCTIAHMGDSRIYHIRPAEHYIWHTRDHSLVNDMYEIGEISLEDMKNYPQKNVITRAMQPGQERRSKAAVKLITDVKAGDFFFLCSDGMLEETEDENLLNIFSDPDTTDEEKREILVKVTEDNRDNHSAYIIHVTGVTEDTKENDTPCTTQEESTEEEPNERFVAPQPVDNTAEVIRPAKQPAEKKVEATATQAQQPAKHSLICHIDKKMRYVLFLALVALAVFCISMIIIYFVRLK

>tr|R6CG47|OS=Firmicutes bacterium CAG:56

MERNEDNFCFNGIGMPEVHDDLEENLTAEVGGQWVAVFDGIGGLPRGEEASYLAAKTLLDEERPWTEQDEQNYETILLQMLKKINDNITSWRKDRKITKMGTTVSALKFGQEAIYGLSIGDSRIYRMHDGKLEQLSTDHTFRRPGHMKSALTEYLGKEHQAEFPENSFFCVPYEKGDRYLICSDGLTDMLEESKIKSRMEQPVEEAVTSLIEETIRMGADDNTTIILAEVESETDEVQNGSDAMQSESDAVMGKQIEYIQDGAEITSEEWIMQLQQEKYRSFGFEYKNWKNENLIFYRTDQDTNEKHYYSNFVGIVRWADKMLLSLPKSVKLDQSEGYLKKLEMLKVYAKLLDLYLADVWEAAERNQKCKPKKAPWNMVSQSVEQWCAQTEKEQADTAEVQIIAAVKFEKVYEWLLGWLYGNQISLFGEKVFFESKILDQKEININECQNNVYSWKVYGKSGNGIQDRTPVFKNQEKKNIPDIVIEMDKEDSELKNICCILDAKYCGWDGESYILPGNADIYKQFFYQEQFVKLYSKQAPEKEVKIYNALIFPDYMGDTVRTNKNKEGILRLCAVAAFDLHKDRTIGIWQVNLQKLIEGRISSDEEVQKRMQKNCRGITESLIKTGKDFLPMRTK

>tr|R7FCZ0|OS=Ruminococcus sp. CAG:330

MRPEFMIDYRSKTGIEHQKRNLENQDACFSEIQEGKNACLCIADGAGSKKHAALGAQQLVQNLCTTLMQNASELFQKAEQEISDFLSEKIHQLLREVAKAKNVEFRELSSTLLFTLTNGETYITGHLGDGVILGEQGSKWSVLSFPQNGQTARTTYLTTMPLLKNHLRITKKDCKELNRIWMMTDGAMYATFQPYFQLPKGHSLEKSILQAAHKMTEDDATYGYLAWKGEDSYAIPI

>tr|R7FGG0|OS=Ruminococcus sp. CAG:330

MKLTHSTSAVTGSHIPTIHVTAQSMAGQYHAVRSQDNEDSIIKFENDSIMVVAVSDGCSESAGAKAASQASCEASVKFAKTRDVWSTDDNTLKSAFLSVIDDVYMDTKLPYNLLKATLLLLVVSKITGDYVGISIGDCSALVLDENLNKPEVLLKPYNPFMQATRTVFANNKDADALMAIVRGRVQDKAGFVLFTDGAKALLEDTYEEEVKKLVGLTAINAGEAAQTEADVLVNTFQQGGHDDVSIIAVGLENPNTVRIASTLYQPKQEEAPESEPAPAPIPMEIKTEKTEEMPAPDKPEKPAMPAAEPAQEFTAEELLKNGIIQRPSEFFVQISPFLQNSLMTYSAATGAFQFCDLSGQS

>tr|R7J223|OS=Prevotella sp. CAG:873

MKIEIRQPYSFRQLGRSPRQEDARYPDLDVPGRDTRVFVVCDGVGGHGSGDIASALVAAAVGEYMCGLNLSRPLSAETVGKAVGQAMQKLREASDGKPSDMSTTLAMVCLHGGGVMAAHIGDSRIYHIRPDAGILYRSEDHSLVNVLVHTGNITPQEAMNHPQSNVITRSIGCSATGERRSPASVMQISDVERGDYLFVCSDGLTKVVDDDMLVQILSGDATDFEKTAALARLCRDSVDNSTAFLIPVAGICGVGSEHVRATGCDRGEITELLDRASREVRDVSPDTRTKDCRLCRRLAKWIARWRRRSPWPCLRVQTCNNANTTDTNHG

>tr|R7MMG0|OS=Ruminococcus sp. CAG:624

MQSVISYLQNHLPTEWNVEVICDVLKEAYDSAKKSIEAISQSSEISIREYDTTLTTAIYNGNQVVYAHVGDGGIVQLLTNGQFLQLTAAQKGDEFNSVEPLRNEKAWVFGASDENVCAFAMFTDGVYDVVCPWLLASEKQKIYVNYVRLYMDMNVIKATSEEDFVMLKENAESFLLSEYNSNITDDKTVAVVVNTDIVPPLQSDEYYIEPNWEALKRRNDEKLYS

>tr|R7TLU4|OS=Capitella teleta

IADAPTWNCKSRKAYGICMSLYDQHPINGKISGDPIADSFAICARTNNALMIVADGVNWGEKSKMAARCAVYGCMHFMNQKLFHSTKPIKTTHVCLDNIYISESAFDLLLASLDTAHQKILDHDGGLTTLCAALVLPLKESKQFAVCIVNVGDSLAFVFSKNHGAREITTGSHDIQAERDIRDAGGALGPVNGTDPELHNLTCSMTVVDPGDIVYLTTDGISDNFDPVVTKLAVA

>tr|R8MU82|OS=Bacillus cereus (strain VD146)

MKITTYQQKSPLKQECEDSFFCNEDKMIYGVCDGATPLVPFCDEEGHNGAYIASHLFASHFTSLREINSLQGEVAKANELLQNKMLEYKVDTRKKDHLWCTCIAAVQIEGDKLEYAQLGDCMIVAILQDGAIRVLTKDTVKGISKRAKKKREEDRKKGLPVPEEHVFQDVREQLKYNRYLANMPNGYSVANGMKEAMDYLQYGELQVAEVSGIFICSDGLFHPEWSLEQTVAYIRKNSIKEYVAIIERLEGENRIRPDDKTVIMIDL

>tr|R9IXU0|OS=Lachnospiraceae bacterium 3-1

MMHYTKMHGKIHPQKLFPMIEQVLFAVEQTHECGFIHRDISPANMICTKDGDLYLIDFGAATSCDRNSELWNEQVFSHKGFEAPEHLLYNNHGTWTDIYSLCASVVYLLTGEGLPSAKEREKADCIPQILMRSGLSGRQQNILMKGLAIDSRRRCASAKELRMALCGETVKFEDVWDVAYTARTDIGSRKMNQDNLMVDGLFCYEGEDFRKAGQIICMHEELHMAAVCDGVGGACLGELASRAAAQALMHFMEQYRYSHKLPERLIDELLDQMNEKVASLGKKIGTVATTLSLLLWKGNHYWAVNIGDSPIYLLRKRKISRLSVPHTRAYAHFMSGRPIGRSDWHVLMNYLGKEKTAGSQMAAIRHGHLQKGDVFLICSDGVTDHLDEAGLKRCLLKGGEKGMESIWKVLNCKDSNDNCSAVIVCF

>tr|R9LWY5|OS=Anaerotruncus sp. G3(2012)

MNLPVHAIAILGLTALLVVLIVLRARPKRYPAKPAADIGKCMTIGHREVQEDNCAAEVTDAGILAVLADGMGKRYGGRVASRIAVQIFSDLFLDYNAFDNPQYYFRKAFHAANRQILETVDEQRGSASVAAAMIRSGKLYYALAGDVRIAVFHRGDLVPVSEGHTVDVLAQREFRRGRLTRQDAVAMLERHRLYNYVGQDGFRDIEFFDTPLTLDAQDTVVLMSDGVYDCLAWREIEEVLEGKGSCQDKALEIIQRINRCPVEDKDNASILLVRP

>tr|R9MVG0|OS=Lachnospiraceae bacterium 10-1

MILFLYLFILLLTVIRFTDKEKGKVMRRFKVGKAMTIGTRQVQEDNYGICQSSEGFLAVLADGMGKNYGGKVSSRIAVETMKGMFAGYQAVENPSYFFQKSFSRVNNEILNQLEEGRGGASLGAVLIKDNFLYYAVAGNVKLAVYRNDDLIPLSTGHTIDMLVEDRFQEGTITREDALKFLENRRIYNYLGQDDFQEIEFFDTPVRLKEKDLVVLMSDGLYEGTEWRTIEELLAGKKKCQQKALEIIEVINADSREDKDNASIVLVEACT

>tr|R9MZ84|OS=Lachnospiraceae bacterium 10-1

MVLKMISGVYWDQGAGRINQDSVALQQVMTYKGRVMMAVVSDGIGGLKEGEVASGYITERLIENFYCQMVPLTAKGKGRKAVKRSVLRCLYDINENLKYYGKGKDILLGATISLLFVWGRNYMIIHLGDSRIYRYQTSKYHKCRVRQLTKDHSDGGSRLKKCMGSFPFQFPDITFGHLLGKSGFLLCTDGFYRKLNNEIIKVLAPEDVESDEQVYIRLREIGEEILKKDEKDNLSAVYAVIG

>tr|S0EVU6|OS=Chthonomonas calidirosea (strain DSM 23976 / ICMP 18418 / T49)

MTETYSTSQGGQEPTAKFEREVLVRGWRSYAPRAPRCLPLIKFAARTDMGQVRENNEDKFDFYEPEEPAILAQRGCLYAVADGMGGALAGQIASELTLKRLLSAYYDHPADDPHVAMREAILEANNYIYALAQAIPERNGMGSTLVAAVFIEDRVFIVQVGDSRAYLWRRGQFRQVTVDHSWVEEQVRAGVLTREEAELSPFRNVITRSIGATPNVLPDFYDESVQEGDIWLLCSDGLTGHVEDAEIADIIGTHAPSEATRQLVELANARGGRDNITVFVLSIRAVVAYEEAQAFFTAPQMMPKEAAVDDGSLSSTPKPAAPFWRRFLLGS

>tr|S0FRI1|OS=Desulfotignum phosphitoxidans DSM 13687

MTRKINCWEYMGCGREPGGRHAADKGICPAAVDRSHDGTNEGTCAGRFCWAVAGTLCHNRVQGTYAGKQEDCLDCEFYLQVRAEQGSTNIRTKFLKFIHPFAASPILNHLEPVQIPGGARFITQGSRTSTGYIIQQGACLELVENEHGLHPVGHRSEGDVVGMISLLTGEPMGFHVEAETDLEAWAIHKPDFDRIPEQDPDLYAFLTELVADRFDRNGPIAERRIGPYLITDIIGKGGYSIVYKAVHLDLETPVAVKMLRHHLSMNKEFKDNFKNEARIIAGLDHPHILKVYDITSRFKTVFIACEYLTGLSLEEMITRQHRIPSDLARSFLDQLLSAMAYAGNKGLVHRDINPANIMVSKDHPIKLIDFGLACPVGTDDFMMGGNLHYLAPEVFDGEPADFRSDLFSLGITAFHTITGRLPWDAVDSGEIMKQIRHQPLPDPGKQVKHLPGSLRQFILKACEKNPDKRFQTPEEARKWLMNATDFGPDPVRRSGPVLLPGRYCCMSSGQHETRDALPGRHMDIAAATHIGHQRKTNQDRYLTCLENHSDPENHDFALLALADGMGGAIGGEIAADHVIKHLLGLSLQDDESPLNSLKRFYRKMDRDICDMADKDPYLNGMGTTLVCAVVSNNTVFWAHSGDSRLYLLHGDHLTRITRDQTLADFLIREKQITPDQAQTHYSRQVLEQYIGCGELAVQSGRFELAEDDMILLMSDGCYRHISLDTIITTCRQTTDPATAADALIKAALTEDGSDNITGVILTLRTDV

>tr|S3B3H3|OS=Streptomyces sp. HPH0547

MSEKGEPDAEHRDWWARLYDSDAPDTGRARRPDTLDDRYDSVTRTLAGPGRAAPGAAVSRPAAAAEPAGRDLGRDLGRDLGRDLEGGAVGRVPVVGLDAAVEAVPDTVLEEGRCGAATLRAASVRGAGARTAGVPRGDALLVVRFDAGESGLVLAAVASGAYRATREATRSVAAALGRGSARLADDVREGRAEALRAGLHRVSDRVFGRLRAQAAAQEAPPGAHTADLRCLLLPADPHCRTRVFFGHGAGGLFRLREGAWCDLDPPHTEEDAAPAGPRPLVEPAREGRDSPERGGPARADPFRFRLWTAEAGDALLLCTEGLARAVRGAADSGARLAAAWRKPGGEVPGAAAYLTALVDGTAGQEGDRTAVTVWEE

>tr|S3X206|OS=Propionibacterium sp. oral taxon 192 str. F0372

MNEINWSQPPSANTKPKLRLSASHVPDEAPGVPGGVSQPVRTFLPQPITCAKCSGEIDFDGYCLICGEKAADPRMHYEMTSCDWVAGVCDRGVRHPGNEDALALDASAFEQGRAVLVVCDGVSMAADSAEASLSAAHAAVAAVNAAEFDWDLSPQAVQAATGEVLGGVALAANQAVLDNSDLQVQNPASCTLAIGLLNGRSMLSATVGDSRVYWLPDSGAAMQLSIDDSMAQQQMDAGMDRLTAETGEYGHVITRWLGRDAPDVRPRVSVATAEGDGWFLVCSDGLWNYASDPSEMSELVAAIVRELGEPTPLELASRLVGWANTQGGADNVTVAVARVTVEPGSETQEAAENELDSATVRLRG

>tr|S4MAC3|OS=Streptomyces afghaniensis 772

MLDGARYGACTVRAVSVRGDSARYRGEPRRDSLLTARFGTGEQALLLVAMATGARATPGAHRAAAEACHWIGRAVGRSHARLVEDIRAGRRGDLKSGLHRLTDRSLGKLRASAAEQGVDPEEYAAGLRCLLLPADPECRTRVFFGVGPGGLFRLRDGEWQDIEPQVAEIKGEPVVGFGSPPSETPDGDRLTMDLGIPTPPSPYEPAPEPPREPFRFRTSVARPGDTLLVCSAGLAEPLRGEPELGEYLAERWSRPEPPGLAAFLADSQVRVKGYADDRTAAAVWEA

>tr|S5VCV0|OS=Streptomyces collinus (strain DSM 40733 / Tue 365)

MLKWLRGRGPGPGGARTATGPSLAGDGPAVGGSGPTAPGDGRTALTRNRGYGSRPDGDGGDGTAGGRTAAGAHAGGGPAAGRPTGEPAPGPAHSAAPGSEASVSEASGSDASVSEAPGSAASGSGISRSDPPGSRPHGCDDSGSGAPGFGESGSGAVRSTEPDPAAPGSGRAVPHLAEPTAADTARPPRTLPTDAPRIGHLQSLPLPRLSAVETSLPGVRADAGLLRNRWLGAASLAGQSHLNGGTTAQDAYQFTVSDDGSLLVAVVCDGLGSRPLTSQLGAVLLSSLLCQAARPVTAEHLAADPYAVLGGVLGGACARLADVRAAVLPALADRDLACTAVLVLVPAEGTGWAARVGDCAVLTLTDGAWDTVFPREEGPLNKVSAALPHPAPADAAEYARLPEGPGGTLVLGSDGFAEDVYGSPGVRDWLAACWSRPCDATAMADSLRYRRGGSHDDRTALVLWPPSTGGRA

>tr|T2GDS1|OS=Megalodesulfovibrio gigas (strain ATCC 19364 / DSM 1382 / NCIMB 9332 / VKM B-1759)

MHACGVSRAGPRHTSNQDAWSITEPLACGMSLCIVSDGVGGRAAGEVASRFVAKALPALLRRELTNTPQTGPHACADIVARCVRRASRHLRQHAQRLPALTGMSATLAMLLLRDDEALVAHLGDSRVYQLRDMTITPLTEDHSFTANLVSMGDLRSSQAFRHPARHQLMRCLGMQRDPQPDVTMVHCLPGDRFLLCTDGVSKPLGDDTLRLLLTRDGMPETLAHGLLAEVDRRKGKDDATAVLVFV

>tr|U1I987|OS=Gallibacterium anatis 12656/12

MSGLEQIQKIKSWLEEMDEESIDAFATQNATLVKLVSETFINFRRGIENVKVIMPTESTVVQKIMPAIQLVNAKQDETYLCKPNTDRLIKDVHFSQECGLCWNAEQKVIEGIPTVSGEIQVSFLLEDGSTSLGTLWINPDPRKLWDNIPSNKNERFWKDDSASDEISTNFGKLLAARMRGRSHAHKGICCDDDFTIAFHEKSGVHFIAVADGAGSAEFSRLGSKLAVEAAKEKVLEQLTNNDKYQAISSSFETDKLKGIANGLLFQAVQSAFQAQQTEAEKEHIPLKSLSCTLLIALTLRLENGQWFTACYWVGDGAAVIVDLGSPKVKLLGEVDSGNYSGETVFLTHSEIEAEKLVSRIHTDLQYYPPLLMLMTDGVSDPKFKTDAKLQTIEAWQALWDELKIPLQAENPAKALEQWLDFWSKGEHDDRTLAMFISQDEWNGVVNQRQNDISSEAHQVALLPNKQAQISTEEVAQKSGESEIEDEAERTINITSNGVTTTITLTKVSDQKMDTQGANQ

>tr|U1P3Z4|OS=halophilic archaeon J07HX5

MIAARLRQALERLKGIFGSSEPTNKLEPANNQPRPPAQVAGCSVGGKKNERNEDAWSARTPTEETLVIAVGDGLGSKTHAHEGSAAATTAAAAELAEAEALTDDGDATSDRLAAPMEAAFAAARTAVEQRASTLGAPVDECATTLLAVASDRSETVAAAVGDGGIVGDDDGVYFPVIDREAGEYANVTTPLTAAGWRDAYRFGYTDRAEAVAVFTDGLSNFTWERAGDATPETAFFEQVFPPVHSARRAAEVEPALCAFLGDEHFRQHSRDDKTLVVGVPVPRPSTPTATVSAGTTDPAPRSTSRSTGETDR

>tr|U1RCU9|OS=Actinomyces graevenitzii F0530

MRTRIAVKYAAGTDVGLVRQHNEDAYLASGPVFLVADGMGGHKAGDVAAATALAAFSELTQDHLVEPSQLQKAVKRAARAVNGLPGVPAPGSTLTGLVLSENGAMPCVRVLNIGDSRTYYQSKHGFEQITKDHSQVQEQIDAGLISAEQAQHLPGRNVITRALGAGCGPRVRADYFVLPARVGDRYIICSDGLSSMVTDTLIEATAAVVSEPEGVVDALVKAARNAGGRDNISVIVVDIAAAYPPWEDDDLTHQNLPGTQGWDYDPDAPTLDRSLPWRGGNEVASEQWSWMFKDNE

>tr|U1RI57|OS=Actinomyces johnsonii F0510

MLEPFAFGVLQAPVWWNLRRGAVVQDSVLGFTRGVQANAGERETMTEHVRADAEALAIRGADGVGASGVVVGAATDVGRLRTINEDGYLAIAPAFVVVDGMGGHAAGRMATQVALDSLYSLAGTTVTDVETVVRAVMAAQEAIVAIPSHAAYLPGATIAGVVLAWMQDEHGATRPTWVIFNIGDARVYLLRDDMLSQVTRDHSRVQILVETGEITPEQARRDPRRNVVTRALGGGIADSAVPDLYSVPVAAGDRLLVCSDGLSDELDDEAIATVLAAGYTAQRTAELLVAASLEGGGHDNTTAVVVDSIEVPTPAHAAVPGIAAVQEAQ

>tr|U4UAZ6|OS=Dendroctonus ponderosae

MYPEEIQNEKMPSLRKRVSTYLRQLSFHNEPREKKNNVNENAFVTKYLQGHISLQEGPPIIYGKTPTDLPNYELAKYESGTNTVVGCYSGPNGGLTTVKRTEKHLSVPDDDIDFIDNPEEEEHLPPPMPKISPKRVSGQPIDNTTMVIKVAGNEYCVTNKPKRHSKSFSLGNDSTENQQFAIDDASLPAEWTRKSDCFYGVSDSLYDRNQVTKQKNGDPIADCFGLITRNDSAILAVADGVNWGEKASIAAKSAIHASLHYLNKTIFNDTKPFEKLTTKTDDVALSSDALSTDETTNLIANTREVFVCLLRAFNCAHDLILENQGMLTTLTVAVILPLNETRSQRHENGIHSNNRKSHYVCCVCNVGDTLAYVYSQKSGIRELTKGSHDVNSNRDMRDALGALGPVDGINPELSNLTLSITTLNEGDIVLVASDGLTDNFDPNVCRFTVNTVNSKADVTKNRVQNTSSSGVAPSHLPNSQLQGNARLLEGRTQPSSVNSQRAEGVISQKPPIKPPRRSKNRGSISSASQSERSASSVEKDNVPISEAPNQGYVNKNLAAVKTKDLTEMENPLVAHFVKENSPGDHRTVKSEVKKQRAVIDNKASSLRKQVSTQNRTELDRKVPPAVKANPNTLDSSFAKQKTQTHKFLKSKTSLDLRSSHQNKIQIPRNVDGIPFVTPYQRYELQLLLMEDILKNGISGNDSPITTAKRLCENLVSFTMSITSAKRRTLEDQDMYFDHRNGVLVEVSNQEKKLRRKKGLEKVQSLPGKLDHVTVVAYNVGNVAI

>tr|U5BZR9|OS=Rhodonellum psychrophilum GCM71

MFAFNESVYNNLETLNCIIILVFLVGLVIFLYLVWDLKMERKERGKQKTKMIKKEPILNLIIKIRAIFTTSFNHFERKIIDSKPEKTKESKNYYIKIEPDTPKSKPIDPQSTPPESQSKSDLTEEPKNIDKELKEAALTPPLIITNDFLKSNSKVVITGDWLIVGGSSIGKSHVESKMPCQDNFFLSAISENLGIAVVCDGAGSAKNSQLGSKFVSERVALIFSQMVESEIGLESIKSLSEEQWSNFAKRGLFQARKDLEEYSLKEDLKFESLACTVIVVIYHPHGILITHIGDGRAGYLSKEKGWLPLMIPFKGEEANETVFITSKIWSEKDIDTYLEAKVFIEEYSGFTLMSDGCESHSYLCSIFDEDSQTWTDPNMPFSRFFDPMMDGLKIMRDSGLSPAEIESKWIDFLRSGTKGISNEPDDKTLVLAVLKK

>tr|U5E601|OS=Nocardia asteroides NBRC 15531

MTEAEGAEPRRRPSLVEAIKGLGAQMMPRGTGPEHANSDNTPPDTQNVANSQPAGATRRGGQLPLRRCPSPVNSASAALAADAGVVGNSWIYAASAVGLSHALEGVRREDAYAADAVNDTNCVIAVGDGLGSTANASVASTAAVLTFTASVCRANSAAGSWENTARTAVREVNQQLYAVQERSIPTAPGAGPATASQRGKSMAPPKSTLTGFVLRHDGERSKLYWAAYGDSPLLLLSLSTNTWQWVSGQPTNPPTAATPALPGDERRLQFGSLALQPDQVVVAASDGVGDAIAMAPKDFAKALAEAWHTQVSAAKFATLLDFEIGGLNDDRTIVMAKGITPGMFESQ

>tr|U6RGV7|OS=Phocaeicola massiliensis B84634

MIPINTQLPIAVGFVDSRQGGRAENQDTCGYADTPLGLLVVVCDGMGGGPSGKAASSTATDVIIQTVRSGKLSDSPLTVLQQAIKMANQALISEIQKKPALRGMGTTVTALLINEYSAIAAYVGDSRIYQFRRGHKKFRTFDHSMVFEMVRNGTINEEQARLSEQSNMITKALGANSDIEADIVELPYEKGDRFMLCTDGIWGMFPERKLIDIVAGTSSLGGAVESIVIRVDEEGIANGGKHDNLTVALIETNSNSILKEKMSTKIRNILFALIFICCVSIAGNIIQGFYLPGQAVASSKSEELDIEALQKVWSEKLQAEFDEKLRKSEQEQKRTIDSLSQIITNNPGKAKEYMEVIINQYDIIERLDDIINNLQELRDVSEGKEKEQKLKETVAMVQKLEPELKKKYGISENEFNGKEKNGREYGILLLLKQNIAKENSSRAQSHYDSIINRVKEIRDKIK

>tr|V4J9X2|OS=Thiohalocapsa sp. PB-PSB1

MSTSNEDQLVQEILLEIYKRLPDAQACSDAGIQTRLRSQTQDLIALARSAASCHARQNDIAEGECTEDNTEQPAQSAHGQTDQSDQAVTEARAGIQAEPGVADSSEHRQEQIRPSTVANASEEPAESLIPDRAAHPDNHQLKQAASSTPQDSGEHPPELSEPTIAPSDRNAAPERSATIDPHRKPGAIPTASLPSAEPALPRLIVTIAANANAGKPYQAAIEARTDSGHPVRILHCDIPTQSGVRYEDGALVGSTPEAGEYRIQVTGACEDIEYPLTASADLIVNHDPKTLWKDIPSDPCAPGWKLDERAFATSGAGGRRLIGASIRGRSHAHTGGFREDDFLLQASTDGRWNIIAVADGAGSASRSRIGSRIATEVAVQQAQAQLLSLGPAAIDEWRVSGDKEPTRALRVMFYQALGTAALEALKAIEEQARYHDADIREYATTLLLATHTHTEIGDLVGTFWVGDGAIALCDQAGGARLLGQPDSGAFSGQTRFLDRSAVDSGQEIMSRIHCAITPALDSLLLMTDGVSDPLFESDNELHSPARWRELQDQLMPLIQTDTATEKLVSWLKFWRKGHHDDRTIVVLY

>tr|V5C2X8|OS=Methyloglobulus morosus KoM1

MINYAAFTHTGKHHRQNQDAIFLPGIVQQKAGFWEGQFNLDAPLRFAVADGVGGLPSAATASRILLQELMVLDTAQPDLLPRQRLFPLHHRLVKSCQSQRALQNAGSTLVTAEIAADGQIGLWHVGDSRGYHYKPQGLRRLTDDHTLAYCLSRSGSHSQIQLDAVAKTRMGQALDNLFIYSPEAEEPFIGLQRLQLLPGETLLLVSDGVTSHLSDDALAVCLSGNGLAANVQRIFDAVMAQGAEDNLSAVAIAIM

>tr|V6JU11|OS=Streptomyces niveus NCIMB 11891

MNGAPGAAPSGGVAWPAAAETDSTATPAPVQVPGELPGLDSTGVPLSGLTAGGHRAAPPVPSAAPVTPTGDFELPAPAPAVSTGAPPSVPHVTDHPSAEPPDLADPRTVAPAVPAGTKLCVACRAGHVDTDGYCENCGHAQPRERDHVEQELDAVAAVSDRGLRHHRNEDAFAVSSAALPDGSPAVIAIVCDGVSSATRPDEASAAASAAANEVLLAALPRGTHPQQAMHEAIVAAAEAVNVLADPPGQSGDQEPAHRAQNAPACTIVGAVVVSGLLVIGWVGDSRAYWVPDDRTGPPARLTEDDSWAAQMVAAGLMNEAEAYADERAHAITGWLGADSYELEPHTASFKPERPGVVVVCTDGLWNYAESPQEMARVLPPDAAGRPLRAAQVLVGHALDGGGHDNVTVALLPFAVPRHGAGSDRA

>tr|V9HB68|OS=Simonsiella muelleri ATCC 29453

MKKLMNTHDSWRYWANDLSEQDWLDFVQENDAVVQAFISAWQTFYDKNYPVTTVSIQVATPNAPKNHLNPATELIAPTEKNESSSELMPIRQPENPKPVYELQQKTDLPLAEVANSPTELIALNPEIRQPENPKTVLLTKEPVMPIAPKITPLPNARCGENYLVTLPKSAQDVVFKPDCGLIWDAATRSIHGKPTYSGDVEVRYSLVYGDNVIPTRQTLYINPNPRDLWKNIPSNEKARFAKPDVFCDEIDTIEGKLLAARVRGRSHAHVGTHCDDDFTIRHHLRTNLHLIAVSDGAGSAEFSRLGSQVVVDAVADTVWELLDSQEDNFTILSQFDLDNCKRVLINLTTRAVYCAYTSLHNAAKDEQIPLKQLSCTLLFALSLPMENGQWLTATYSVGDGAVAIWRPEKQQLDFVSKSDGGSYSGETKFLTVEETTKDSLTKRVRCIVSDDSPVLLLMTDGVSDPKFETDAQLSNPQRWADLWAELQTPLRNTQPEKALEAWLDFWSAGNHDDRTLALFVPSVFFRQPERLTELSSSSIKTENAS

>tr|V9Z5Y1|OS=Streptomyces sp. FR1

MITYATAQALGDRPVQCDATAVATESGARAWVLLDGIGSTAQVQTWTREKARVLARVAAVTRSPRAAITAARIVSDSDGYEDEHRPPDAVAVIAVRTADGRLLIGWAGDSRAYWMPTGGALEKLTVDHNEAEVRRARGETHIPPDYRHFVTSSLRRYDGEEGEIGTARPLRAQGGRLLLVSDGTYSPFEDNGIDMRAPLSQETPRGVASALVRTAVSFTDVRRDNATAMVVQFD

>tr|W0A9S3|OS=Sphingomonas sanxanigenens DSM 19645

MSTPPVSVSGLFGTSATIGKREVQEDAVGWSASHNETGGLPALTVIADGMGGHAAGEVASDIAVNGFLDAWRRGEHGGKTTLIEALDQANALISAHVAEHPETDGMGCTLIAVELDGQTAAFRWISVGDSLLLSVTEAGVERLNADHSYREERERLIAAGESLDGAPAPNVLRSALMGFDVPLIDDRQDWRAFAPGETLILATDGIETLSDAQILAIVAANPGANAVAAALVMAVEAAGKPRQDNTTVAVLRPDADEAASPNTAPMPPPTAGDQPTLSVTADAAAAARAGAGIGAPEAPTLPARPARAAATAAKRAPDVRIILIGVAVAVLALLAILAFSGLLSRKPARSGDAPAAAAPAAPVTDAATEPAPADPDEAAATQGAQQPAERAEAGTGGNAAANNSASNTGSGKPAATSRGAKTPPPPARTSRGQPAVTEPTVAGPN

>tr|W0EA51|OS=Desulfitobacterium metallireducens DSM 15288

MKVVSFSETGCVRKNNEDSLLVLSQYGLYAVADGMGGHLAGEVASRTALEVLSEASPELEGLGEDAVLDWVKQALIQANRRVFEASTVNPENEGMGTTLTVLVFKADQVVIAHVGDSRGYLWRKGNLIPLTVDHSLVEELVRMGQISPEEAEKHPQRHVLVRAVGTSQEVKVDSGCFDCQPGDFFLLCTDGFSNVITEQELIQELSQTGSWEEHFERMRQLILDRGAPDNFTALGCIME

>tr|W1WLW0|OS=human gut metagenome

MFNVTVSGGCQIGDKRKYNQDNFYLNRQFRKNTRENNNLFLDINTNDEIQIYAVCEGMGEDKLGEIASSVAVQTLAKYHEEAFKLCSKSTIQKCIEEYIEDVNERLCDIRNNLNEKYIGTTIALVAIYGDYLYAYNLGNTKIYFISSNEIIQMSKDNIDIIPSKVLFRINKGDKFLICSDNLSTSISNTEIRKIIKNSKSDNDAVQKLVDKSLLEENIDNITAILINIESREYNRVEILLLSIFFIFIIVGINFLS

>tr|W4MCL2|OS=Candidatus Entotheonella gemina

MSDLETKIIFFGISDVGRHRENNEDRFIVADLTRKVIGVYDNTVTPDLICHKIGAFGTVFIVADGLGGHEKGEVASQIAVEGTVQFLFDMENESGCPAEWLNAAIQSAHSNIRKASNYSDSPKGMGSTITAIHVGQDVMTVAQVGDSRAYSFRDGKLKLLTEDQTLVGMLQKRGLLTDDQAQSHPSKHVILQALGQDKEVYAEIDSHQLRDNDYLLLCTDGLS

>tr|W4V2K1|OS=Acetivibrio straminisolvens JCM 21531

MYSEKNIILRASAATNVGFVRSINEDNFYLNGIYFSPSTTEKTVLFDINEADSFFLFAVSDGMGGEAFGDKASLTAMLELKKAHNFIKNNNNGNIEGCVNRINSYIKNTNNLIYNLGVEYGARTGATFAALLIYGGQAKALNLGDSRIYHVRDNEIIRLTKDHTEAERLIRLGILSLKKHRTAQVNTCFTSFSAYLRKKELLKRTFRKAFLLKRGMYFFFALTALQIWLKMSS

>tr|W6JV50|OS=Tetrasphaera australiensis Ben110

MPGAASSYDATPVAVRCGTCGEGVYTDGYCDQCGAKEPNPRDHLEENVAVWVGGVSDIGRRHSRNEDALALAAQADPVSWAVLVVCDGVSNTTDSDIASLAAARAARTVLEQPLSSGMGVAAAAQAAATKRLGEAVAAANGAVIGKTKQGDEHSPSCTFTGAIVDGRTAYVANVGDSRIYWLPDSGGGEQLSIDDSVAAEQIASGIERKVAETGPMAHSITRWLGIDAPDDLTPHTRTIQLSEPGWLMLCTDGLWNYCSEASALHDKIVEFQANGTPAQPLPLARALVGFANNAGGADNITVALARIPADGEDIAAAGAVLQAPAPEATAEVPPVPGKTSDATAPDPAGVTDASTQAPGIPAEESGTPAGPLEAPAEAPAETPTQALGIPGEQPSIPAEQTAAGAAAPPTGETQDTESAGDLAGSAHTEKGQ

>tr|W6JWJ1|OS=Tetrasphaera australiensis Ben110

MEKPTTPDDPIVDSEPPAQERTPAPVQEPQTTSPEQAPQPRSPVLPDASPSTPPFDAFAEIQELFNPAEVDAEPSAHAGVETDAAEPPQLVADDSEAALLPTDQREELRWTRPAAPDDVSVQEGVYRGRTQVGEAGAMLRILPSVRSKLAGAFVPDSIVDSGIVSDSVSIGVISIKGASHHLSGIPRQDAYAIGSDDSWVVIGIADGVSEGKLSHVAATEAARVAVTESIRALTASDPADVSWPEVGRKAREAIRALGKRRAQQQVGPTDTAPEISDRTIARIMSTTCDLLIAPTARKDGSLRVWRVRISGDGSLYVLDPTQGWGLLGNGKDAASSTVDNSVGDPLPIGGEHPDIESWELRPGQAIILCTDGFGDVIGEGALPVGRYLFDAWQQPLDTTQLLFTSSFVNTNADDDRTAAIVWATA

>tr|W9G4H1|OS=Intrasporangium oryzae NRRL B-24470

MTPDSSGPSGEPSAEPSAAPSAESPADDSAGRSGEPPWSVGVATDVGRVRSHNEDAARAEGGVFVVADGMGGHAAGEVASRIAVDAVAELAGRPDLSVDDLVAQVGEANRRILDSVAEHPARSGMGTTVTGLAVVRGEGGREWAVFNVGDSRTYRFADGRLAQVTVDHSHVQELVDSGIITPEQARWHPGRNVVTRVLGRPALRSVDVWLLPPQPGQVFVLCSDGLSNELTDAEIEGILTEAGGEVLVDAQAVADELVRRAVEAGGRDNVTVVVVATGGAPADAVAPDSEAPREAPTGHA

>tr|W9UWH3|OS=Nitrincola nitratireducens

MSNRLILSYGSSTHQGARPYQEDSCYWLETNKSGCIGFVLSDGMGGHAAGDVASKTLVKAYRQVFSKEVTLDTLVSQLKASMDLGNQQICEIITKKPELTGMGATYLAGFVYDKILYWISVGDSPLYLYSNGKLRQVNEDHSMAPILLERVIKGEITQEQASSHPQKNSLISVVMGDQIEKVDQPTTGIALSPGDVVIIASDGIQTLSEKEIERLVEKWQQHTPHDDLTDMLLQAVLNKKNPKQDNTTIMVAALMPTDGSLSDLHGTPSNASTANQVSWSALMRDDDPVMEHSFSKENITVVKWVILLLVMIVLGLVAYFYIDRLKDEALSPEATKDHLIEKSADFDQSIDQIFDSTLIDDSIQEPIPVLEEPVNDRVSDNADAVSEMTNQDKKVSEG

>tr|W9UYU0|OS=Nitrincola nitratireducens

MKTKTYDIASAIHIGGREEQQDSVKVYTLNNNHLLVVADGMGGHRGGQLASKIAVDVCVKCFQQEKGSPSNPKDFLKQMVHHASEEIKTQGELQNISPRTTLVVAIILGNHAYWAHVGDSRLYYFKQSVFSQRTRDHSVVQMLLDTDQITEEEMGTHPDQNRLLQSVGGENPPKVSLGETALEAGDALLLCSDGLWERFDIPEITKAINLEGKVGIETWPQKMVDEAAKRGGPKGDNISLAVYKHYGNNSSKVQNTRVLISGLVFLTLMLAVAAFAFFKPAEKPIPNTEPTQIEELTRTEESISDDLNSVAPYSEAVPASKVEPPYTEASNDDNQRVIQVE

>tr|W9VUT2|OS=Imhoffiella purpurea

MAVLDGMGGHAHGREIAEAAVDALRLLPPCSSAIAQRHAILDLHHDLLRRFGTGQANSPGTTLIWAEVDRLRRRCHLLHLGDSRAWLGLDDDWQLLTRDHTLAEFGYRDGRIDASAYAAERKRPDRRLAQALGHGAWGLKMDAEGHESFGFSPDIRLDSVRDLPSLEGHADLRTLHLPRGVPLMLATDGLWSGGTCRLPPPTDLMAPGALGGLAQAAIDAGSTDNTSLLIGFFDPESTS

>tr|X1CJF5|OS=marine sediment metagenome

MGAHAAGELASKLAADSIPHLFSKDLDNDPINSLHQAVTGANTTIYQRGQSEPEFTGMGTTCSTLLLLPAGAVIAHVGDSRVYRLRGGQLDQLTFDHSLVWEVQAAGKNSGTAADLHLPKNIITRSLGPAPNVQIDLEGPFPVHKGDIYFLCSDGLSGQVSDHELGAILGCLPPAESAQILVDLANLRGGPDNITVIVVQVDHAPVA

>tr|X1F7A7|OS=marine sediment metagenome

MAATPLHVTDDKPNRPASVTQQVAARPYLSVAGHTDVGKVREKNEDTFVVADLTGGTLLEGAPHARFDVGERGVLLAVSDGMGGAAAGEVASALVVETLTRAMKDAAPETPRDALMNDAIQRAHRAVWDEAKRESKKMGATLTAVFVHAGQAFIAEVGDSRAYLIRAGKMCQLTHDQSMVQMLVDTGIIEPDQAEHSPIRNVILQAMGN

>tr|X8CD76|OS=Mycobacterium intracellulare 1956

MTLVLRYAARSDRGLVRANNEDSVYAGARLLALADGMGGHAAGEVASQLVIAALAHLDDDEPGGDLLAKLDNAVRSGNAAIAAQVEAEPELEGMGTTLTAILFAGDRIGLVHIGDSRGYLLRDGELSQITKDDTFVQTLVDEGRITREEAHSHPQRSLIMRALTGHEVEPTLTMREARAGDRYLLCSDGLSDPVSDETILEALQIPDVAEAAYRLIELALRGGGPDNVTVVVADVVDYDYGQTQPILAGAVSGDEDQMTLPNTSAGRASAIRPRDESAKRVAPQPETPHRPRWSRRRMVVVAALAVLLVVAGLSVGWWVIQRNYYVAEYNGRVSIVRGIQGTLLGVPLQRPYLVGCLNARNELALVSYGQSGHPNCQLMTLQDLRRPGQVQVQTGLPGGSLDQAESQLRQLLAEYLLPICPPPRATSPPGSPSTGGTPQSSTTATPSPQTTSSTSASPHPAQAPPTARVPSTGRSDDHLANGDRSPRAPTPTGHRLPDGGMTTQLQPPVAVTPPLPTRRNAELLLLGFASLITVAALLIVEANQTRDLHWDAINYGLVFLVVFGSAHMAIRRFAPYTDPLLLPIVALLNGLGLVMIHRLDLVTNQLSGRHHPSATQQMLWTLVGVVTFALVVTFLKDHRQLARYGYICGLVGLVLLVIPALLPASLSEQNGAKIWIRLPGFSIQPAEFSKILLLIFFSAVLIAKRGLFTSVGKHFMGLTLPRPRDLAPLLAAWVISVGVMAFEKDLGTSLLLYTSFLVVVYLATQRFSWVGIGLVLFVAGSVVAYFIFSHVRVRVQMWWDPFSDPDGSGYQIVQSLFSFATGGIFGTGLGNGQPDTVPAASTDFIIAAFGEELGLVGLASILMLYTIVIVRGMRTAIATRDSFGKLLAAGLASTLALQLFIVVGGVTQLIPLTGLTTPWMSYGGSSLLANYVLLAILARISHSARRPLRAPARHEPPIAAAGTEVIEKV

>tr|A0A1E3PMZ6|OS=Nadsonia fulvescens var. elongata DSM 6958

MRTDFKDCSVSPVPPINSSISPLRFCCATSRIPFHQYKNVSDTLIQKYYPPSLKVSLGSSRQTNTTFVKNADDSLLCSNQFLGIADGVSEWNEKSNGFTSLWSQLILLRTLNWLEKTMVNPAEFSILGDGSDSSLLTRAMDQSFDETNYVMENFGYMGSSTLLLAYLRLRKLQIMNIGDSRLWVIRDGQFIHTNEVQHTSAPGQIGTNSSIYPSDKAEISIIDIQPGDIVILMTDGISDNLWPEDILSITLEGLKSGGIQMAAKQLTANAFSTANDNYAVCPYMLHNESSMAYRGGRNDDATVCVAIVEENSA

>tr|A0A1E3Q4Y4|OS=Lipomyces starkeyi NRRL Y-11557

MPAPTRFSLLVAAYPTITAALLFAFRRLQYQYPILDRIITHQTQARLFQHNTATGLAITTPTTNTAAQYTAQISQSATGASGASMASNNFQYTISPSYHAKDRNLVARKYSGSVAANSGKRATRPASGEDSFFFSKIRESESVALGVIDGVGGWNEIGVDATDFSHSLAEAMAELTAKLSTDTSAKRDESNSIVSKFSFPPLVLLDAGYNAIRNSGKVKAGGSTACVGVAHGNGTLQAANLGDSGFFIFRDGKIHYMSIPQTHYFNAPYQLAIIPQKIIEQNARYGGKNFDDKPSDANLSTHKLKHGDVVLFCTDGVLDNLLPSDCLRIVNEEMISQGNWIVDRRSGDIQASGERQYSGVDSLAKRLVSAAFKASIDPKTDGPFAIEAQRQMKVLYKGGKPDDITALVMFVHQHVGGKTQEKL

>tr|A0A1E3QMB9|OS=Babjeviella inositovora NRRL Y-12698

MCPRNIITSRLCRALHNNSHRYSTLSQPQTPTTSAVKRFWTSLYAPMESYRPKPRTPKQDAETAARKLAAMHKRKTAAIAAAAKASTHIIDKFRFDFSFASHVHHGSKHTPLISSLTDLTDFEFSAVPRVPRRRVAGNVDTISVRSGDDALIASPYLLGIADGVSSWGANSDAGVWSRLILENMSRNVMQFKLNITDKTKTTHVPSYHFQEQEVLRCLDHSFVQTLETMDTDAIEGSSTVLISLILGNKLKIISIGDSKVYVIRKGALIETNEEQMQSGLCPQQIGTNHTDVLPSLVTWMDSVELQADDIVIVCSDGLSDNMWQDELVECVNKKLAEMERDGEEDMQKLANYLLYRSKDVAFDNFAVCPYTEKVNNLSLTQRCLDGLAVGGKVDDISICVARVVENY

>tr|A0A1E4B7V5|OS=Gemmatimonadetes bacterium SCN 70-22

MKTPVEPMAPRTPTPPGGDEAPVMVEVYALTDVGRTREHNEDAFVVADLATGKAMEFGHVLRQGAGTVGTLFMVADGMGGAAAGELASETAVDVVLRYLRDEWAPRRSLDAGAFAESLESATSMANSAIYRHAAEHPELRGMGTTATIAGLLGDTLYLAQVGDSRAYLVRDGVARQITKDQSLMQKLIEAGEISEEAAEQSERRNIILQALGPEAMIRIDLTHQRVRRGDVLVLCSDGLSSVVRAEEIARAVGEERDLQMACRRLVALANGLGGPDNITVVAARFEGAGLAAPNADDHVGHRTFAIPGRQVVQGETLDDLLDARTAQMEAVYEKTPLELPAAVRAARREKSLVVRGVLAASAVVLGGYLVWRLLQLLGR

>tr|A0A1E4CR31|OS=Microbacterium sp. SCN 70-27

MSELPIVVRAGAASHTGLRRRVNEDSFLAESPLFLVADGMGGHDAGDRASAAVVSEFGRLTGAPSLGIDEVRATLRRARGRVEAIRSEGRAAGTTLTGVVIAEVGGVGYWLTVNVGDSRTYRFANGMLEQISVDHSVVQELIDAGVLSGADASSDSRRNEITRAIGAGSDGQADYWMVPAEPGDRILVCSDGLSGELSAVQLRDILVGEDDPQEAATRLIHEALLHGGRDNITAIVVDALAVSGVDDGVFDTAPAGARGIEPDSDTRPRSGSRGAR

>tr|A0A1F7XYI0|OS=Candidatus Woesebacteria bacterium RIFCSPHIGHO2_01_FULL_38_9

MENTGASEEVSLQPPETTSKRRDLRVEMKQGSEASKSHLDRNEDALFVLPDKNAFGLFDGVGGHTSGKDASNIANDSIKDRFSNEIHEGMSLDEARNLVGSAMIDASKKVYDAGTEEIRAGKTTEDKRMGSTGIAGFVWKGSGEERKLVFGSVGDSRVYLIREGKLEQKSIDDNEKSLAKAKTALGFSDNEIKILQDKLKNISSLDELSPKEKGFYGQFGQMIDKFLGDETLQPRVYTVDLRPGDRILVTSDGVPDNLTQDRAGDSDSIQEILSHNSDDTLAVKELIEKAKGKGKKPDDITVIVFTVPPIEVPSIVKTGPTGIEAAVVAAKGQTEAEVTTLGAFGKEKKIKLSARELIEVKATQLAKLMSTEFKQKWDKESRDGMGRLERVWQGAKTLSERAWKGTLGEMPHFVKEKRHAIKLMAATGVEGDFPYEAFSEIDRRAREAIAQERSTGLKRFVGGVKDLGAELFARRRDLHKKRIEIAGQLRRE

>tr|A0A1G2VJ95|OS=Parcubacteria group bacterium RIFCSPLOWO2_01_FULL_48_18

MVSSEELKRKDEQPLASHEGAERYFESAGESVKHLGPERGESNEDAWFSDEAKGLFAVFDGMGGHSSGEVASRMAKEYVENAVRALREDVSVEELKTEIERILNEASNRILNDSLDKPEYRGMGTTASVVKLWKGPDNQRKAVIGNVGDSRVYVLRRNESLEQITLDDSRIREVSGNEEQARQLQASLNDVEDPATLAEPARSLFKTRNVITQALGTFRAEPRMYVVDIHGGDTLLITSDGVHDNLTSKEILDELMKVGGVAEEVDVLLQKAKARSQEKHPRGEIDPETGEVKKHPRAKDDDMTAVVIKLGGPKESSSQNIVS

>tr|A0A1H0FRM9|OS=Prevotella communis

MIQIASPTYQLFGFANSMQGGRPENQDDFGFAETPLGFALVVCDGMGGGPGGKTASYIAKNEFLSTLMQCNVQTPPATALRMAVSRANDAMEEKMNLVPELRGMGSTLVAVLVSRQSAFIAHLGDSRCYQLRGRRVRYRTDDHSLVGDLVRGKVLTEEQARVSPQSNVITRGLGNTSNHVADVVEVPYRKGDCFVLCTDGVWGSMRHEDLLQRLTSVQDIASKVGNLSAEVDRIGFAAGGHHDNHTLAIIETKTDSILKEKMSKQVKILLATLSCMLLVSIILNILLLVKMSNHAVAVNDSGAGGVPVAESGSLTSSEEPIATIETSSVTEKAEGKVTTITEENDSLGRQIKKLKETQAEQQDKAGQTVKEMLEQVVAQLNGLKAINEKDRKDASKKSEVFTKTVKQLLTTLKEKVPAPNKQKVADIESHVSGEAFDCTKNIWPSKKMFVTSPNTRKVIDEKILPKVEELKKNL

>tr|A0A1H0KPV1|OS=Nakamurella panacisegetis

MSHVIRYAARTDRGLLRANNQDSVFAGDRLLVIADGMGGHAAGDVASRLVVAAFVDLDELPLGSDMVRPLTEATREGNAAIAEMVEENPEFDGMGTTLTALLFDGPVVALAHVGDSRAYLYRNGVLHQISHDDTFVQSLVDDGRITADEAAHHPQRSLLLRALNGMEMDPSITLRETSPGDRFLICSDGLSDVVSPESIADTLAGPDVDQVADTLIQLALVGGGPDNVTVIVADVLETGATGSPGVAAGAVDPEATGPMQPIHLTQRMPRVPLPPIPEDIPAKPEYAPAEEGPELHNGDDEEYDDEDDFSAASDIDAHDDPDDHGMVSPPRRHWRRRWAFGIALLVLIGAGLTGSVLWANSKYYVGQDGREVAVFRGVNGSLLGWKFASVQENSCGTTPSGCRPLMVTDLVQAARDQVSAGIPVSTLADARAVITRLTSEQLPPCPTTGTGSTLSSLTPASSGAASSSVAASSAPTTPTSSVVVPPTTAAKTTAAKTTAKVTPPRTTAKSTAKATARTAARTTSKASGAAKPTTTPRAVKATTTALKAIAPVTTTVVVTVNPLAPSAAAVTDGQHLRSRGVLPTPASALGTAPITRSNPAGVTPTITVFTTTTLLPTAATPSAQPGTPLVSPAAEPGVTCRPVS

>tr|A0A1H0ZG20|OS=Leucobacter chromiiresistens

MTARGENGVRRRLNYRAGGELELELSWFAVTDVGRRRENNQDSYVTTPPVFAIADGMGGHSAGEIASAAVVRRLAELGGRELVSEQDIDETLSDAVDDIELDAGDTELGAGTTVTGVVLGTEAEPTWKVFNIGDSRVYQYFKGALSQITVDHSVVQHLIDTGAITEEEAEVHPHANVITRAVGFNEAPIPDYTSLALIPGQRILICSDGLTKELTDVGIQHFLATEPTAESAARSLVRHALENAGRDNVTVVVIDVHAVGDVVDTGSLAGSGIELVEAGGSAPLESGASRAAAAVTASPRSGREDADRASTDREQVPGADDASADGFADDRSAEYDDLEPVILPDAPPTPRAERA

>tr|A0A1H5L7Z6|OS=Ruania alba

MAVRFRYALSTHLGTVRTNNEDSAFGSDRLLVLADGMGGHAAGEVASAVAMRVFAGLGTGDAPAAETILAAGRRTRRALHTMSEADPMLESMGTTLLVVACDGDQVTVGHIGDSRVYALRDGSLYQVTTDHTHVQRLIDTGQLTPERARTHPYRSMLLKSLDDQPAGADLDIIDVELQAGDRLLLCSDGLSDYLSAEHIGELLSVPDREEAAHALVEAALAVGTRDNVTVLVADLEADARATDSSTPDGGVRLPGQENQVVGAAGEAIGLSAEAAAALRATLPDMPLDATAPMAGIAGIGATDKTDHTSDPDETATDEPAASESDHRDSTEAAPHDDQATEAAPSGNSDTSSGEGTRTGEPAPSGLSASTGSRLPGALAALTVLAIVIALWIILG

>tr|A0A1H8G5R1|OS=Prevotella sp. ne3005

MKPINTSLPITGFWDSRQGGRDENQDQCAVVDTPLGFLALVCDGMGGGPSGSFASDTAVRKIYEYLNAPHEDMNRKKVLKEAIEYAHQSILSLGDENPRLRGMGTTVVAMLINDYSAIIAHVGDSRAYQFRYGHKIFRTSDHSMVAEMVRNGVLTEEQARLSSQSNIITKALGGNLKDLAEVSEHAYEAGDKFLLCSDGIWGMVPEKELISLTAKPSVLSKAVANTVDTIEGIGRESGNTHDNMTIVMLEMKKDSKLKVKMSKKTIRLLAIMAVVCLLSIVANIILAKKLSAPNMAEQKVEVLTKELSAKKKQIEDLQTEVNKLNTEVAKSKRETADAQLEVAVEKNKAAERAQAEAEEKVKEATKASEKAKAAAQQAQKTANEIAALRKKILDDLNTAKNTKKKDARKVIIKRMVSNLSLLSAKDTKNKTVYEDIKDKLSAPISTSNSPNAIGHYDILIKRLNAIK

>tr|A0A1I0YV15|OS=Cellulomonas marina

MRTQWGSATDRGAVREVNEDALLAHPPVFLVADGMGGHDAGDVASRIAVEEFADLAGRGATPDDVHACFLRAAHRIRTEFTGGRQGGTTVAGVVLTHDGGRPLWLVLNVGDSRVYRVTADGLVQVSVDHSVVGELVAAGGLDAAGARTHPDRHVLTRVLGSTEVVEPDYWLLPAAEGDRLLVCSDGLTRELHDEDVAALLAHPDPQAAAAALVDAAVGRGARDNVTTVVVDAVPAGRTATGVLVGSVGAARAGTGPVVGPAGPGVPPADGWDDARDGVTVPRTPRPRPSLEPGAVARPDVVPSSPPVPTVPPVPPPGGAA

>tr|A0A1I2FA60|OS=Nannocystis exedens

MSRRSRRLTLTPASSAGLLASDDAALPALVPLDRVSGRPQRWSPVPAPYFRGERGLRTRCAAATDIGRQRVQNEDALAIDPKLGLFVVCDGVGGRVSGEVASALAASTIREWVQREATRLAAVARSPGDAEAVASVGVLMHDAIQGASRAIRGLARSEPQHEGMCTTATVLLIVNDFAVVGQVGDSRAYLGRGVGVYQLTEDHTLHNLQIPQGLLTPDSARGCKSPITRALGREDAAEADIGALPLMAGDRLLLCSDGLHEYLADDELRELLRLDIRDAAPAAIQHANARGGRDNITALFGRTRWSSLAVDRGVRDGRRGESVAGLAGGAARGRTADPAGRRRRGRRSHDARRAARGARGARGRRGARSGRRSSAARRRGDADDDADARRSRELRARERVARCPRSGGRRGRGDLRRARGGVARVTRPVGHFGRTTRIARPRHPPLPRPDERRLDRRTWIAADDFTIADVLLATLPTVPRARRDRRRRARLFGKAAPIGERRVEHELGLARGRHP

>tr|A0A1J1IGH5|OS=Clunio marinus

MSRTLNETHKLALPEKSEYCTLKTKEEILLCSGVSEENNSETLIDPRQNATRILKTPNNLVKDHRYTSELKVHEHKIMFGMLKSNIKHNPRINNISHQQFRKISQTPTAALIEKHKIEVMQFNKCAEIFFYRHMNELNDLLSEDMKVFLIDHWQRIEDLSIFQVVTKLLLNKTLNTIEIKVEHLNSESEINSSPYTIIEEYFPIINRLSSEDLKFYCQLNEIYKSEMIFYPTSDINLSVDLLSKLFSDETFSVRFDNCINRDGFKFCNFDKPLPLKSVEIQQALEEIVKLKIYMMIDWSIMDGFIQISEHKEEVFRAETISNQLKKLFLMFRKRAGNNKKNHLWKINNDGQNYILNVRQPNVYFMMDKEILQPVNISVKLEYQTNFGAEKMTSNELIKEWCTLKFTSSSNIIRYRIDAKTLEVLSMTTVNIEEIEKELKKNYNINPSDLIVNLINVFGCIKRLPEGEYLIQAINEDTWKNMYIYRKSTSGKNFSDKPWEIKKVFTSKWTPIDEETPTFIHIPNHNAPCCFQPSCDSKYRVRYFSKPEKRAQTKNKLDKVQKTLNAIRNLQMPNKRVGSKNKVDKAQKSFTKPLLIPENRKAKTNSMPSLGQKVFGYFRQLSFIAEPREEIKPINQCFITKYLETSGAEPTQVNGPSIKICSGKQPTDLPELTLSSYEYGQNSAKIALTGPNEGHTYVHRSRLHLSAGGDIDFIDDKDEEVRHNPKKFPRKLSTVSRKSTSSHETASLMPSDFKKATDEENNESNHLIADVSHWNMTSTDQGHGIAISLYEKNPITNEHAGNPIADCYGLVARKNSCMMAMADGVNWGEKARLASCAAIQASLEYLTRALFSPGNTAKNTREVFVSLLRSFWEAQDFILEVNGHLTTLTVCVILPLSDEEVNKNKYVICACNVGDSLGYVYSRTHGVREFTQEELIDSHDITNNRDMRDALGALGPVDGNKPELSNLTLSLTIVEKGDIVYLTSDGISDNFDPVVGKFAEAEPEVTETTTPVTEVTKAELAPKRQNKSASSLYPKRPQASSSSSSSSTKASVSFPNSDIPVRPPRSKKAALTSAVISNPMPVTDQLPLRPKYTRSKTLIEPRHSSKSSPQIRIRKSPAGLPIVSAYQRHALTLLRMSDLFCYGINGTLRPCTNAKKLCSLLIDFTRMITSAKRKLLEQRELFWKISYDANGQRKEVEMTRVQQRVARKRMVDSHFSSLPGKLDHASVVAWTVGIEDGNITNNNNDILLDPKAKTLSILMARVRNDFRNGNYNERRVITRVNYNESPLYNRAGKTRLAKWYMNFDDDEKQKLIEEVHAVVTVRDAKHTNFVEFRNFKIVYRRYAGLYFCICVDVNDNNLCYLEAIHNFVEVLNEYFHNVCELDLVFNFYKVYTVVDEMFLAGEIRETSQTKVLKQLLTLNSLD

>tr|A0A1J5DIF7|OS=Deltaproteobacteria bacterium CG2_30_63_29

MSRDVEHPADDSKETERGEKDREVELVAPIVSPFNSPALADTAPVAVVRRFDGPPPSFSDPTPEHPFFGDPDESSETYDDEPLELLPASTGDTTSPGVFVLAKSDSNEHTRELPVVRAEELWLVHDRPPQVLKAGTEIVINLHNVNILSCIAVQTFERRYRVAVDGFEHPVMLKQGAKGAQFVRYELEAEILKKVQASEENLRFPKFYDYWEDDECKYLLTELPGGRPYFNLLESGELELRDHVRILQEVAAILRTLHHLGYVLTSLRPMSFDVTVNRGYEVRLTDFLNVCETVERPPYALASPFTAPEITELGPADEGADIFSIGALLHRIVTGEDVPGERVHGLFIPQLVDAQVPMVFQLLARSLGSPSERFFDIEELEKAFQQLERELIPRLRVSTTMRTSTGINPLRIVNQDSGGFIERKSLHRSIQTHTGFYCVADGMGGHEDGDRASELAVQGALRCYEQLSLELNHDQWRDGCALLCRRIAAAGSRNLVETITRSGGGRRMGTTFTGVLVVDNQLALAHIGDSRAILFRNNQLEFLSEDHSLVGMMVKAGQMTEEQAERADEKNILMRSVGAEHIIQAEMFDGFESTLGAKVFTAEPGDRLVLVSDGVWGMIPRIDMVEVFRRTKACDDLCDALCSEAIKRGGGDNVIVVALDFREEPSFTR

>tr|A0A1J7BCW1|OS=Mangrovactinospora gilvigrisea

MTQPDGRCPHCGAQVFPDERFCVECGYDQAQPDAAAAAPAAPAAAPAAAPAPPTGAAPPIPPPPAEPPPHAPTLVLETTPAESASPRTCVHCGSSEIVPEGYCGVCGQAQPRPRDHMENETGHAAAVSNRGHRHHRNEDSFALGGTELPDGTPVTVAVVCDGVSSASRPDEASEAASRAAVDHLHDALAEGENPERAMYDAVLRAAEAVRDIERPPGSRNAPACTFVAGAAFDGVAVLGWVGDSRIYFVPDDRELPAQRLTEDDSWAAGMIRQGLMTEAEAFADHRAHAITGWLGADNDYLEPHTRIYRAEHNGVLIVCTDGLWNYADPAAELAAVVPPHARLNPLTAARRLVNFALDGGGHDNITVAVVPLAASPDPRRVPGELPAEGGIADLATADASGPATADAGAGRLDPGGMDAVDATADPQAPAPAAADPESWPLQAPGGPVPGPVPRAEPVPDTVPDPNTRQEQRPWHD

>tr|A0A1L6MXJ3|OS=Pajaroellobacter abortibovis

MTYIPESVVKGEVHSAVREKFTSGNVYIRFHTKTDVGQVRKHNEDNFLLADITRKIRQDVNRTYNYLVGEQGALFAVCDGMGGAAAGEIASQLAVDVLYQRMTENLSTNAPLLRDELAYQLIQAIQHASFQIFQEAAIDKTRRGMGTTITAAALVDDCLFLAQVGDSRGYIFRQGRLVQVTRDQSLVNQLIEAGQLTEAEAKNFELNNIILQALGTSESVQVDLTYVPLYQQDTLLLCSDGLSGMLESEEIETILAIYKDPEEICKVLVEQANQAGGQDNITVIIVQFNGDGLSPPNALQENPQYCKYAFPETYKPSSQQDEHLQNSAHNHQDHQQFQLYPTTKPFSHHSPSSSQESSPENPNHTFIYPNQLELIPSDSFSDHPPLKQNPLLPPFVVESWTPSSRKRLFSQNPYVNGSVFLLLLGVIAILILGSLAFSFC

>tr|A0A1M8A2S7|OS=Malassezia sympodialis (strain ATCC 42132)

MFNAAVYGIPKPRANRPPQRKAESRTRTMFAPLADFLMGTPADPPARPADAEDDVEEPQLPSSLSYLHGKGLQSTIRYHGRIVKTRLQSGQVGEDAYFLKDDALGIADGVGGWASNAHADPALFSRLLMHFCHDELSKLDERQLHAWAGDTSEALTAWFNCDPVQIMQVAWERCVRASKREGILGSATALLAVLRGDELRVANMGDCVLVLIRDKELIFRSAEQQHSFNYPVQLGMMDATVESVTLASALCMHRDGTIPDGAEDLDLPDVNEKMSRYIHSYDASPENPDFDSPRHDAGIWGLKVQPGDVVLMASDGLFDNLFDDDILETVLEVLDSRPGADLEHDPDLPHAVARALCERARGVMEDPRGIVSPFQYHANEEGIYYVGGKNDDVTVLTGVVTEHLEPHHAGPPGVEW

>tr|A0A1Q3GTS9|OS=marine bacterium AO1-C

MSATNYISELGEENTATETLVNLFGRTDVGKAREHNEDNFVICQNVQGNDWEFDEHALKLGKLGCVMVVADGMGGANAGEVASEIMVTTAKSMFQQITTLPESSKGVKDFLIKVLNTAHQNILEHAKKDSQTEGMGTTGVLAWVIGNKAYVAWAGDSRVYLHRAGKQLRPATDDHSMVWQLVLNGHLTPEEARVHPQSNIITQSLGDPNNGPKPDTKTLALQAGDRLMLCSDGLNGMLDDIELEGILNQHQDTATTCQELVNQANLAGGGDNITVLLMDVLETSSNTESTYRDTADQTGGKTTMKKGILWGVSLVILFFSIFLLTQSISNSSSKPNAADSALVGGGKALNSEKLRQIWQNFQKQKEKDQDTTSKKTDSTRSQGDGRQRNNITDESAPPTERVDQSLIKALEAQLSQLLNEKAGVKKKIQQLKIKYKDVPGDLAKLKALEQRLTDEIASPLLSNKVIEGANQLRVPKTMREYEKAKNTIERVEANLVIIQEKMRYWALNPSNG

>tr|A0A1Q3HMY6|OS=Archangium sp. Cb G35

MSLPLYIHGSSDVGRQRSQNEDSYRIGVQPDGSRLLVVCDGMGGHEAGEVASQVASDRLVEVLSTSAPDNPPRALYEAFVAANQAVLEAAGTRGAPGMGTTGVIAWVMSSRCYVGWVGDSRLYQFRAGGLIDRTRDHTRVAQMVAHGILTADEARNHPDSHVLVQALGGSPGVQKSFKPEVWTEPLELRSGDVVLLCSDGLYDFIEDHELYPLIEGRDYQDAVTRLIQTANERGGADNITVILLVAGQPEVPRRVTVPQEARRETLPDGMPMLAPVPTPEPTVHAPPVEPASPVPAPRRREPTVPVMPAAPAEAVPSGNAGRRVPLWWLLATGILTMGVGIALGLGAGNTPSTPSAPPPPAPVQSTSPVVEVDGGTEQDAGVAQSGPPEGMGTPHTTPGAAPPGSSQVTESGGK

>tr|A0A1Q3TSA0|OS=Chloroflexi bacterium 54-19

MLKLEPVALTDVGRKRSNNQDYLGDLIFKSGRKYGPEKLNDRGHLFAVADGMGGYAGGEVASELAITTLFERYYNGTSSGDIGTDLSDAIKAANLKVHTEASGSGRPQMGTTLTTVLVKGNKAIFGNVGDSRTYLIRQGLAERVTHDHSLVQDQIDAGVLTPEQAERSAIKNFITRAIGHRDDVESDLFEREIQPGDVLLLCSDGLHGLVKEQEMGTIVATAPSMQEAAQDLVNMANERGGPDNISVMLIKVEDIGEPLPNILKGREPVYNSNRHLFNQTTEPLGAVSASAATTVSQADRATVPSPVPVPAAIPSAYSQPTAQMATVSETEMKKGGGRGIILGIIGLLVVAAIAVIVFVLISGQPNPTPVPATTLPPQTSAAAATPTVTATPATTATRTTVAALPTATPPAGTAAPGTGSGSASGAVANPTPTGDRNPRGTVGPTEDQSNSASFTNCPTTTPKDGLRCWQIQNMGQIRTIRVTLANPTSGLTLRLQPPATSSAVTIPFNLETGSATTFEATGTLLPGRYSLVITNPNSTASPESYDVLLENDPAQIWGTPEVQIRQDNDNLVILINNK

>tr|A0A1Q7CMZ7|OS=Actinobacteria bacterium 13_2_20CM_2_66_6

MRENNEDRYLVREGRNTMLLAVADGVGGEAGGEMASAAAIDALAAAIRGANDAVLGAAGESGQKGAASTLVAAAIDGTSAFIGNLGDSRAYLLRDGDIRMVTADHAGDYQSSITRFVGDPRGVQPDVFVETLRPGDRLLLCSDGLTRHVPDADIASTVRSRDLGSAVSALVDLAKSRGGEDNITVVLYAARRTFGLADVRRNFVNLILALLVALVVGGTIAALIFASGAYPIAR

>tr|A0A1Q7YKW3|OS=Acidobacteria bacterium 13_1_20CM_3_53_8

MSEQPATVEIQAAAVSDRGLSEKRPLNEDSFLSDAQRGIFAVADGVGGAQAGEVASRTAMEVLDEAFRHQLEDADVEDLMEIAIQRANASIFQMSREHPKFLMMATTVVALHLDGHTATIGHVGDSRLYRVTPDGKIHRETDDHSMVEEEVRAGRMTPQQAANHPSRNVISRALGAEDTVEVDLKIVEIEDATTFLLCSDGITRHIQDRELEAILNRPESLEQICEEMKRRCYDRGAEDNLTAVVVHVGQRRAVQPTEEVPFDEERTVQFEKVSTAAAPASASAIEAAAPITNEQAAPALQTPRGVDRLMDAQPAQPAPMPPQTEQAAIPASTARGGAGRTFGLLLALLILLVAIGAAAFYGGLQYHLRLSRRVRAILR

>tr|A0A1R0H0F2|OS=Smittium mucronatum

MSNLFQVGVPLVRGLTTRNSKSSFRCIYSQANIKGGKQTKYSFLVGAYGIPKNKKDSSGQYEPFNGYPKPLKFDNSESSWNVGEDAFFFRKDAIGLGDGIGGWSTKKNSSSALFSKRLMYNTCHEIGHFKDGEDDAEDSEIEISSSSVLKDAFISTLRDMKKAKLKGSSTACVALLRGDELQVTNIGDSGLTIIRDGELIFRTEEQQHSFNYPYQLGTEENSDDVSNAQTFRIKVKKNDLIILASDGLYDNLFDEDILEEIENVLDPDHEGKILNPGQSLDKKKSSVKLGVQDDEAKPLFLLKSIASVLARRAYKVSLDPDCTQSPFQFHAIYEGLYYHGGKHDDITVVVALITDN

>tr|A0A1R3RPB0|OS=Aspergillus carbonarius (strain ITEM 5010)

MIALASSGRACGVACSSLHTIARDGFLASLAPKPSGLRYHPIPSPLSTPRSPRFFPRRAFHSTSPLDSATPRISYRVAASSSGKGRRFHPAKNAYNFTPELHEAIGVATDTQNKALRRKRRPDSGEDAFFVSRVGQKDSGAVAFAVADGVGGWAESRVDPADFSHALCGYMAQSAISWDSPAEQLRAKNLLQAGYDQVVADETIRAGGSTASVGVGLEDGRVELANLGDSGSVLLRLAAVHHYSIPQTHGFNTPYQLSIIPPRMRAQASIFGGAFLEDYPRDAAVTNLHMQHGDVLILATDGVFDNLNNQDILKLVTSRMVLTGAWAANPDSGIRPSEGLKQLTSPEGLKSLLPSPSADPDSPKSSSSSTREQTYTLQSLIAATIAGEAKLASMDMRRDGPFAKEAQRYYPGDWYRGGKVDDICVVAVVAVDEGYSG

>tr|A0A1S1QYV6|OS=Pseudofrankia sp. EUN1h

MKSSLLPIMLIVVLLVGATVFVVLSSRSGRTQYGPGGQRPPADEPGRDDGLPGRRAGRRRAGRGNAGGGAGGARRPAPDDRYSSDAANLIPGFGPTVPAGGRAGTQASAPGGGPAAAAGGAAGPGPDPDLDARGADHAWSDDVTTQVPTGGDGGWPDDDRDPESTGWAPPPSGPPHPRAAPPEPATVSSTAPTDGVDPAWVADDAAWTVQSPSNRTPLPEVPAAPPAPAAAAAPFPSAPTEPAPAAPPAASAGITSTGPGPYVAEEPEHFSAPPLRLAAAGRTRRGKRGGPNEDAFVVVDGLLAVADGVGGEAAGQIASTLAVTTVAGFRPQYAADPREGLRAAVERANRVVRQRPKSEPSWRGMACTLDVVVLGRQETTGRTITVAHVGDSTVWLQPGKGEPRQLTTPHAITGGPLLNAVGLADEIEMDLFQVEVRAGDRVVLSSDGMTKVMKPEQLYGLLHQLASDPPERAADALVEAALLAGARDDTTVVVADLVPEPTPR

>tr|A0A1T3NW11|OS=Embleya scabrispora

MPARGGFPERTLRQDELRAERPAAVDDDIFGDLYLDFEDAPTRTPVEPSAAPEPVAEPAVEARAPRGFDDTFAAEPADPAAPADRPEFDHDAAPAERPDTDHAPTAVGEPDGARAASPRDRAPRPAETFAAEAADAEPAPDPVPHTVTPPAVRSESRAEAVPPPPTIAAAGSTDTTLASAPAVPPPPTVPPMPTEPPRVPEPPYTPEAAGVPQTPYTPDAARAPETPHAPEAAPTPEARTELLPVAADAVEEPDELDVPTTSRSDSLRRAEATEEEIRARSGPPLGVQASAWSTSSHPGRRRAARRERRGLGPELTARDNDDRYAATGEVFIVADGLGGHPAGWYAADVAVRTLVSELAAADLSAGWSDRLGAAIAVADLAVRRHGVAQYEGMRTTVVAAVLQHGRLHLAGCGDSVCWLVRGGRAFRLTEQGNAAEFGRPWLLTSTPLGGSQAPEPELQRIELHPGDRVVLATDGVGHLPDETVADLVHGDPFHAATTLTRTAVESGDDDATAVVVECDALPVPEGHRSGADPREQSEDASGTPGALRSDGGTAW

>tr|A0A1V2L112|OS=Cyberlindnera fabianii

MNFGGQTPTIVVLKEGTDTSQGKGQIISNINACLAIQDTLKPTLGPLGSDILIVGGNGKTTISNDGATILKLLDVVHPAAQILVDVSRSQDAEVGDGTTSVTILSGELLKESKAFIEEGISSHVITKGYRKAVALAIEKVKELSKRIDKESSTEEEYRDLLERCARTAMSSKLINNNSQFFVKMVVDAVLSLDQDDLNEKLIGIKKIPGGGMEDSRFIDGVAFKKTFSYAGFEQQPKKFSNPKILSLNVELELKAEKDNAEVRVEKVGDYQAIVDAEWQIILNKLKLIEDTGAQIVLSKLPIGDLATQYFADRNIFCAGRVASEDMDRVISAVGGSIQSTPTDIKPEHLGTCEFFEEVQIGSERYNIFKGCPQAKTCTLLLRGGAEQVIAEVERSLHDAIMIVKRAVQNNEVVAGGGAVEMEISKYLRDYSKKIAGKEQLIISAFAKALEVIPRQLCENAGFDGTDLLNKLRMAHAKGETWAGVNFRTESIGDNFEEFIWEPALVKINALASATEAAILVLSVDETIKNKESAAPNAGMAPPPGRGRGVPAINAPRLCHVRIGMKGVAGPGAVAAGNKKKSCHPFTYIYIYPQRFSLSRKTYLIHTRGYQAVMSAVPKSTARSGMFSNLVGLVTGRSKSSPIVPDPRGPSQSYLQNKTTINGSSNLTPRSVPKQFATPDHYKFLYSFASYVHHGSIRKPVVNSLMDLMDSTSHLSLLPRRRLYGNPYETLSVKNGDDAMIVSPNLLGVADGVSGWSGAHADSGLFARSFLENISRNFAELSHKNKEHLNRISNEQLKEFLDLSYNESLQVMERENFKGSSTVIIAMIIDKKLKVLNIGDSRLFILRDGEILATNKEQYISNMCPEQVGTTAEEKLPSSVAQISDYELETGDLLLLCSDGVTDNLYQDEILDFVNTQLNADKTNLGEVCHSLMVKVKSVAFDNYVVTPYVEKVNELNTDFITGGKLDDISVCLARVAENN

>tr|A0A1V5FQF3|OS=candidate division BRC1 bacterium ADurb.BinA292

MQFEHFALTHTGLVREMNQDSILALPRCGVFMVADGMGGEKAGDEASAQVVKTTQAAVEAFFKSKPTGPSQIENMLRDTLLEANHEVFQISVREPAKRGLGSTASLLCLHRGVYFVAQVGDSRVYRLRDGAAVQLTHDHTLVWLLYEQGNITRDQLETHPERHLLTQCIGSQKPVKVDVFEGDLQVGDTFLICSDGLTGYAREEKVLEILGETEISLEQRAGMMVDAALEAGGGDNVSVILVKITQLDAEDNWEPEATAPPTEFTEDTVDMLIDDEMRPQARPRRRRAGLLWGALVAVLAAGALFFLLPREAVGPNAIAVYLEPAEGVALPAGAVTATIVSTAADTTASQTLQAEDDAAPSFTVPGPGEYQVTLQSAGYAPLTLPLTVPAAEPAVERVSLPIAEWTRYGTYTLDYGDPSQLSRVRLFRADANGRVPFDRAAELTVTPDQLAGQSRLSFDLLPGEIYLVQVDVAGRRPITHEKISVDSGETVVYELNLPPPLRADNPTTGGSQP

>tr|A0A1V5X0V7|OS=Deltaproteobacteria bacterium ADurb.Bin207

MSTTRLSVAARTDAGRVRDNNEDSFTVADLNTGERIDDPSAMDGILVKDRGVLLVVSDGMGGHAAGEVASALVVDSLRGTLGEMDDHSESAHQRLEEAVKRVNANVYEAARSSAREGMGATLTAVVVHQREAYIAEVGDSRAYVLRRGRLRQMTKDQSFVQMLIDSGAITTDQAKNFFQKNWIVQAMGLEANVHVALGRLQLRTGDRLLICSDGLYSLVEEHELAELLAGDDLTQACHQMIDLANERGGTDNVTAIVALIESQDLPFAGHDESVTQTFEVLKEYAGEKIVDPSRSDSRAASTLPPSDSQDSKPQSQIQTVRPPAPDNGKSSFFIIILGILALIVVALLGYWFFSS

>tr|A0A1V5XDJ5|OS=Deltaproteobacteria bacterium ADurb.Bin207

MTWLLAVGVALVVFAFVVHRLASKQTAPAIRGAEPKRHSLEDSNRTHRSSKDKSKGKADSVPSSSPNGKKAKPSKGAKSSPALKVSPAVLESIRPLSVPPPANDFDDEWEDVDVTVVAELPDEIKAMQKGYRSVDPKQAVAQIAEEYDDTAEIEIIEERLVDGLLVEELLNDEDTGPNALMLPIGEVLTDVGRRRPNNEDRPLSLPEHFVFGVADGMGGHAAGEVASKIAMETIEKAFEDNVFEGEPNSLWPRRGDELARGIEMANRNIFRVAMEDDALSGMGTTVTAIRFCPERQRAYIAHVGDSRCYRFREGQMRQLTKDHTLGAELGAKGKMATHLSRSVGIAETVEVDLMVDAPRVGDRYLLCTDGLTKMLSEERILEISTDGIVADCVRHLIDEANDNGGKDNITVALVEIREPVGTGMT

>tr|A0A1W9V185|OS=Anaerolineaceae bacterium 4572_5.1

MTVIPTEQAHLHVAALNDPGRKGKNNEDRYAVSSYHFSAEDAMPSLFAIVADGIGGHLAGEVAAEMVVDYVSQAVAESNAQTPLKTITRAVESANQAIVSKAKEERTKQGMGATCVCAWVIGDRLYTGSVGDSRIYLMRGAAIQQLSTDHTWIQEAMDKGILTPAQARGHPNVHVIRRYIGSAEPPEVDFRLRLNSEEDDAQLKANQGVRLQAGDTLLLCSDGLTDLVWNDEILEIVRAAQNLEKAAQKLIDLANERGGHDNITVVLLSVPNGEGLKRKQDNNSLLWIIGGMAVFLAVASVLIGLIWNLIQPDATPTPTLAQIATASPVIETTVAPPTATMLSTLPPPTSTLAPTSGPTYTPWPTNEP

>tr|A0A1X2H903|OS=Syncephalastrum racemosum

MKAVAAAAAFKRVATTTFTTTAALARTQRSFTSTAAVASSSHRRPSHPNPNSNKSNNFAVDKQPLYDYAVHRNVSIDPLPLFDFFAVPKAKPSYSLSHGAAGFAKRRKALPSTVDMNMDLYNSNQVGEDAYFCRSDALGVADGVGGWVNTAGANAAMYSRQLMHYAYQELERFDNVEDPCFYHYDQANPLSILQNSYEHMQRHNNVVGSATACLALLRNDELRIANLGDCGISVIRRNHYVFRSEEQQHAFNFPYQLGTGSMDRPTDAQTFDLSVEKGDVIIVGSDGLFDNLYDREILALVQQYLAQYTLQLGGQMRMLNFQPQKLAHALALRAKTVSEDRRHIDSPFQTRAMHEGIYYQGGKPDDISVLVAVVGDSEDSPDRRL

>tr|A0A1X6X6E5|OS=Brachybacterium nesterenkovii

MSYALRFAARSDVGLVRSNNQDSGYAGSHLIAVADGMGGHAGGDVASSIVIGRLSQLDSETPANDIVAALEETILDANQAILRRAREEPQLAGLGTTVTALLRVEGRFALAHIGDSRAYLLRDGETTQITKDHTFVQRLMDEGRLTAEEAERHPQRSVLMRVLGDVDSEPELDLSLRPARAGDRWMLCSDGLSGLVSHQTIAETLRDVADPDECADALVQMALRAGGPDNITCVIADVVDLDAMAPGEEAPSTSPEVVGAAARTRNRPTSASGPAARAAALTRPERPAAFEDDDYAEDEEPPRRSRWPALVAVFLLIAVIAGGLWAGYAWTQSQYYVTAEDGRVVVYQGVSQDLGPIDLSHRVETTDVPSADLSAVTLQQVEATIPASSRPAAEEIVDSLRDEASRTQLARQQQEEAARQAALQQQQATPAPTPAPSPAPAPAPSPTTGG

>tr|A0A1Y1IL17|OS=Klebsormidium nitens

MSSRMMHGPSASLHKTVAAWGARVSSRRARDLARPIGDAVGLKLFAMPEAANSERHVVSTASAGFRSQFEDLIQKQRQGAASAVDLAVRDKTLALLQKPDIQAAVEASFWRTSRACGESPDESPDMRAAKPVPPFGVGCQAVLEQAAAFHFVHPMTASAIKLPVAADFYWKELNNAGFPRPLPGQVEDDDDEDVAESAARKPHVHTGSLCLRSAAASVPHPEKEFKGGEDAHFVYGEAQAIGVADGVGGWANVGVDAGIYAKELMMRTRDAIRDSAATVRDPLDALARAHAETHSQGSATACVLVLNDDALSAVNVGDSAFVVIRDDRIVFKSPVQQHRFNFPFQLARTRGDPITSAETFRLKAVPGDIVVMGTDGLFDNVFDFELLNIVNVAKRGKLLPHEIANHLASCARARGEDTQRHSPFAKAAMDAGHLYTGGKLDDITVVVSFVCSKHELDVNNPSATG

>tr|A0A1Y1RDX9|OS=Candidatus Cloacimonetes bacterium 4572_55

MSIQTIKSDLGNAVALSDVGMVRSENQDAFAMWEASARGGLPTRLLCVAVADGMGGHQGGSTASRETLLVVKENLSSIDERSVAAHVQASLENANRHVHKLGHQNPELQGMGTTCTLAVITPGELYLGHIGDSRCYEIAEDHISQLSKDHLYVLELLERGALTKEQAANHPDQNVLSRALGTRPSVEVDVSKRIIDPNSTLLFCSDGLYNYLSDSEIRSIVIQNDHQTAIQKLIDLAKERGGADNITVILLALDKDALRSTAEDQKDRYETRQVAAPHSATRKMNIDPEKLNPTNAIDRRDVLKKRVIVILAVLALITALLLGMTFLYYKDKQSSSASNKSVPEDAIAPDKSEHDIDPPTPEPTLQSIKKENQGNECPMPDLQRSNKEANATAILHFGPDDVNFDQYDVYIDGARIHSKKIEKFPGSYDIKVKLDDETICEYKIELAAGDKREIQLPRFSPKKVTQQLTTKLPDVKPDTGSVADPVKAKKGKLNITINGCACKILFKGNRIDDTFPRKMDPGTHKFKFIVNDNFGYTFDKDIEIIAGQTNDVEIKLHKVTIASPGNQNSKNFEIHINERLVEYAPIVGICLPTGEYNISAIGTKSYTISKRIIEQDCTINEWDSGSP

>tr|A0A1Y2ED97|OS=Leucosporidium creatinivorum

MSKSPKSLSTLASSLSLSSISRARSSGTGHVAKYTIRHTPGGAVIVARSRPASASPPTVGKAGRTGSSVASGSGTRDGTGLLRSGSSAGSNGPRALERQLHTTSRSAHQQPIPSSSSSINPPIVPPSSHASSEASFHPRPSSLPASENSLLLNSHQPSVGGPSVSSTSAEASTSASSTSTNSTTTNSTNSSDRATLFTANTPTSFPSHSLPAFFGTSLNIARRSNLVFRNGAYGIPKAGNKDKGKEKERLMIQAMELNDLEYPLSVSVGEDAYFLRTDSLGVADGVGGWSGHAGANPARWSRKLMHHCSAELARYDNVEDELFLRYYDIDPVEVLQRAFEKSLAECKSEGLIGSSTALLAVLRNDELRLANMGDCCCSIIRGNDYIFRSEEQQHSFNYPVQVGTNSKDTPLKDAQRFNIKVQKDDIVILSSDGLLDNIFDNDLLEEVNRFVAASRPSPSSTPPPGASPSSRPPRYTLNQFSPQAVSEALSRRAQSVYEDPHAVASPFQQRAVDEGIYFTGGKRDDITTLVGVVGELEASPDRR

>tr|A0A1Y3SHM9|OS=Flavonifractor sp. An82

MWRTAMSKLTEVIESIQEWALQVLEENRVGQADGETQGNEEHAVQESAGPAAEQTLPAQLASPLPTVLLPEEGELPGEQTIETVPSPAAETVAQTLVPSPSPAADEMTSGGGMWLWMGLGVIVVLAAVAVGWALFRRKSRRSGPTVPAGETGGGLSVGKLHQQGARSSQQDCFSVSDPSVMAQKGLLAVVADGMGGLEQGDKVSQTAVEAAMNGFYLADAQGEQLLLYLLEQANQAVNTFLGPDRQKKSGSTLVMGLIQQGRFYSLSVGDSRICLYRDGQLYQLNREHIFRHELALRAVNQEGSFQDAWTNSQASGLTSFLGMGKLKYVDLPAQPVTVLPGDRFILMSDGVYNAVTEPELTAALDLSTGEEAAQAIGQCIQGKRFAKQDNYTAVILAC

>tr|A0A1Y4EYA9|OS=Blautia sp. An249

MNYKIRYACVCDRGKVRTNNEDNFWCQGLYLDQEHQGLDKTLTGEALQETMPGFAVFDGMGGESCGEIASYLAANAFQKCYQKEIGNGSSDMEGFIRRACREMNREVYQYSLDHKIRSMGSTASVIMFGKEQIYIGNLGDTRIYHFYDDTLKQISKEHSLKRFPGDKKGPLTHYVGIPESELQVSPHIARGSYHHKDKYLICSDGLTDLVSDQEIGQVLKNGKSVKECVEILLEMALNQGGRDNITILLCELEQTGGTEPAPRINRKKKWGIIGAALLLGIVALGVLAWFQFGGTAEETSGEETRQEAEPSKAPDKDREGKNVQLQEQENNTQLQENSIQMQEQEENTVGLVTKPKNQQQEPVVETAEAEYTYDEMEEDLKLLEEQYPDYMSLNSLGKTADQRDIWEAVLGDPQASRHILIQASIHAREYMNTLVAMKQIEEYLTGFESGAYENIPMENLYEDICIHVIPMVNPDGVTISQKGPAGIRDPQLRAAAEGIYQADLQNQKTTLPEEEYWRNWKANARGVDLNRNFDVGWQEYQGTSYPSSDCYKGEAPASEPETQAILQVQQENPLVCCVAYHSSGNLIYWNYGSQGQVLAQDQALAQKVQEVTEYPLHSTIDDGTDSAGCSDYFVLKLNIPAITIENGTGACPLGIEEWETLWTRNKNLFPALAVLYR

>tr|A0A1Y4GLQ7|OS=Barnesiella sp. An22

MKSKIIFKMVAYTDAAGKFSADAPRNGNEDNFFFAYDLSKDTPYKGEPDSDTVLSDCGLLMVVADGMGGMNAGEVASQIAVDTVSDFFAPGKISVELAMDHQKRKKYMERVIGEADIRIKKDAKFNAEHRGMGSTIIMAWIVGDELTLSWCGDSRAYRYNPAGGIELLSKDHSYVQELADKGIIKYEDTFDHPQGNIITRSLGDVTQKAIPETVLFKVYNNDIILLCSDGLSGVLRDKKTYDADGELIEGENLEDIISANRDSLKKCREALWIAAEKADWYDNVTAVLCEIKSGAGTYLPIQKNEIHSKIHNSSSNDIGKVFKGINIHLSAKSILLCFIGLLTIVGMSFFTGWKFFSNKKFQNKTAIDSTFVGNQNSVQYVNISVNFENDRTELKTKLANIQKELDLQSNDSNITNLAGLITEAKDTIELSNAKHKIELLEKKVTFLKQIKEMIQKAPDSKIKGLLEQLYKKIYLSEEIKKDNWARQISEILKTISTPKEVGGMAESGNEEITEIVESKDTIIHDAKIQQDDTYESLIKFLEEKKFPGYSVSIVKEKITGRTLFNIKSSQATIDINTLRGKDVEFTLYRKK

>tr|A0A1Y4W4D6|OS=Flavonifractor sp. An100

MKYEILQLTGGGDPGVSGEDPTVTRPAPDTSAPGGEESGSAPPGTQAGPAGREDGAEAETEQPSEGDTSTQTGGAESQPSDREGDDTQPQQPARQEGDTQPQQPDREGDDTQPQQPAREGEDTQPQQPARQEAEAAGSGAAPSGEGREEERTHSGEEPLPEVDAVLSNGERAGEEGQSLVPTTLTQPSLPGGSGLGPALPFSLGVLAALGVTGLLLWLRRRRGSGALRLPAPPCRGFRCAQVHEVGARDSQQDAFCLAGLESPEGGVLAAVADGMGGLVDSGQVSRALTDALGGSFAPGGTDAPARQLQLLLQQALDQVEDLQKGRTAQSGSTLVMCLIREGALSWLSVGDSRIYLWRGGGLIQLNRDHDFHHDLTLLAMQKDMTFAEADQDPRRENLTSYIGGGFPRKVEWNPEPIPLRAGDRVVLMSDGVYRALSQEEMAWCLGGDAQAAADALRAAVTEKSLPQQDNFTAVILEATGP

>tr|A0A1Y5P284|OS=uncultured Microbacterium sp

MVFEGSSAAISHTGKVRSNNQDSGYCGANLFIVADGMGGHAGGDVASSIAIHRLKDLEHGYTVTADAERDLRDAIADTAGQLIETVAERPELAGMGTTVSALMMVEDHAVIAHIGDSRIYLYRDATLTQITTDHTFVQRLVDSGRITPEEARYHPRRSVLMRVLGDMDPDPEVDTFIMPTQPGDRWLLCSDGLCGVVDDAHTSKTLDHGFAPGRTADLLLKQALDAGAPDNVTIVIVDVGGQHPLVSGTPTIVGSASNPQGIEIPAARSGRGGGWFHPGRVAANEPTHYEPSSEFLEELIEEDRRRARRRRVWWIVGLVAILAAFAIALFGAYSWTQTRYYVGADEDTVVIFRGIQQDIGPIRLSEVHDDTQILLADLPAYQRIAVEQTISARSLADAVAIVDNLRPVTEDTP

>tr|A0A221VXC6|OS=Actinoalloteichus hoggarensis

MGHGDRRWLAYAGGTDTGRGRSVNQDSAYASPRLLAVADGMGGHAHGEIACSVVVAALHELDASLSDPRPGRDLLRMLADGVTDAFARLNALGEARPEAAGMGSTLTALAWDGSRLGVAHIGDSRAYRYSGGVLRQITRDHTLVQSLIDAGRLTQAEAEDHPRRSIVVRALQSGGPEPEPDLFLLDITVGDRLLVCSDGLTAVVSDHAIADVLSGVPEPREAVARLIDLANRGGGPDNITCLVADVVSAPVGAPGGAGRGVSAGRGGGSGAGVGGAGRGAAAGVGTGMTGEERAVVGSAVGAAGGVTSPDTGATGGVVGSAGVGGLTRAGVAAQHSDPDRAAVTVPGSTAGGVGGGTGPMAAGVGTGSGLGAACGPPAGWASTTGVAPAWGDVWVSGDARVLGDVRVVEATRPDRPPGMALRCGDVGPATAPSLVFGAAASRPEAAGRLPAPSAGDRRRSGAVGAGSGIGAGPGIRAGTAVTSVPGSEPRVGGPGIGQATRPGPEAGAGSGAGTDPEARNGPVTETGPVTEAGSGSVTGSGSSTDPRTGFGPVLGDGPGRSTAVRVGSRPPQPPDPGATESHTEHSAACENTEVPRRRSLRLPRWFRRLLGD

>tr|A0A225AN09|OS=Talaromyces atroroseus

MSGLGTIVLVRDVIGVALSGDEKQQKATSTRHKRPGSGEDAFFASTVGAAADAGTVAFAVADGVGGWAEHKIDPADVSHGLCTYMAQHALGDSSSRRKLRPKELLQKGYDSVVADESITAGGTTASVGVAQPDGSVELANLGDSGSVLLRLAAVHQYSTPQTHAFNTPYQLNIIPRRLREQAHIFGGVYFEDTPRDAAVSTLTMQHGDVLVLATDGVFDNLNNSEILKIVTGRMLLTGAWSSSSSPSPPEEKRNNGAIYASEKLRELTEPGALDLASPSSSSSPIKSSTTTNRHHTLQALLAASIVGEAKLASVDVRRDGPFAKEAQRYYPGHWYRGGKVDDITVLVVIAIEE

>tr|A0A225DCL4|OS=Fimbriiglobus ruber

MNIRYASLTDVGVRRSHNQDACATQPASDASVFRTQGHVFIVADGMGGHAVGEKASAKAVRDIPLTYLKHVVHEGPAAAIRRAFREANEAIYGIGQNNPEFKGLGTTGTALFLRSEGAWLGHVGDSRAYRIRGNRIQQLTFDHSWVWEVARRQGIDPDELGDFKKNVIIRSLGPDAEVEADLEGPHPLEPGDRFLLCSDGLSNVVPADELAAVASTFPPDEACRYLVALANLRGGPDNITCLIVQAPGGPGDGSESTRRRGVFGLIRRAAVGWNRFVPWAFTTLGAGVLAAGVSVWLRAEGIPGAVGAFGAAAILILTGLVGLYLHLRLRAEQTSDDPGYVPQELRLYRDYAFEIGKPLVDRFVDMDSSLIEVLKDQDIGEVRETHVKYSAEAAEATGRGDWAAAFRAQFQALQVLAAVYHQHRHKDEVFQPNWVTKTRAAQ

>tr|A0A251XLW8|OS=Clavibacter michiganensis subsp. michiganensis

MATVTQAAAVSHVGKVRSNNQDSGYAGRDLFVVADGMGGHAGGDVASAVALTRIIEADKPYASAHDAEFALQAGLVAANQLLAETVFEHSELTGMGTTVSALARVGRHVAIAHIGDSRIYLFRRGELSQISADHTFVQRLVDSGRITPEEALVHPRRSVLMRVLGDVDAAPEVDTQVLDTHTGDRWLLCSDGLSSYVSEERITEILATAGTPDTVADALVKESLDHGAPDNVTVVVVDVLDEDDETAASRPAPEPVVVGSASQPLAFGTSRRSAPCASRRSCCTAARHHGRARRAVRAGVGPVPRGPHRGGQAPRPPPPRDLAGGRHADRRGTRPRVRPRLPLDPVPLLRGRVRRGRRGLQRRAADHRADRALARLRAHRGAGRRPPALLPPAGGADHQRRLARGAEEIVNRLQEAAGG

>tr|A0A255UEV3|OS=Prevotella sp. P4-51

MDKQTNPTASIRLGEIACYSEIGQKANQEDSIFPAIGEATASQRVFLVCDGMGGHEHGEVASACVAKTVGEKTAVLPLCTTAEMRTVFETALEAAYDELDAIDTPPSTGRTMGTTLTFAACCSDGVLVAHIGDSRVYQLRPGAGVVFRTRDHSLVFDLIAAGELTEEEARDFPQKNVITRAVQPHQERRDRASFNVITDIRPDDVFVLCSDGVVEQLTDDDLCRLMLNHDTLDNRIRSVAEACRLQQTRDNNTAYAFSIEGGSVTAQPEVKVEAVKPKGKKKGHTWVWLLLLAIIAALALFVIKTQGGDDKGDKQPAKTEQHDNNAPLRTIKHK

>tr|A0A257AZS5|OS=Chloracidobacterium sp. CP2_5A

MTTATTANGVRRIEVYALTDVGVVRPHNEDNFLIVNLSDGQSWTAENQLPEHPLLVELRPPDRGVLLAVSDGMGGALAGEVASHLAVTQVCDSMMRLQKDAEFKRFGFHEHLRFAIERANLFINSQSQRSPEYAGMGATFTGAGLDGTMLYLAQVGDSRAYLFRQDLVVQVTTDQSLVEQLIQSGHITREEAETHPYKNVILQALGATPNVTVTVDGLPVCRGDILLLCSDGLSGKINGEDMRAILDATNGNLRLACQRMVDLANERGGEDNITVMILRFTGDGFPERGEVDGYDRITPIDRDDDLPYPTEDKLGLGNEPTQDEADSSPTMELASESGSVQEPTPAELAITGGLGLTRQFSVVAASPAPKADAAASNGAAPAAVVLPPAPAPVAENAADDGLRMYRLVAASALVILSVCALVWTLINWGKDGGRPDGSDPPRNQTRQVAPRSS

>tr|A0A257B536|OS=Chloracidobacterium sp. CP2_5A

MRRAGNEDNLQVVDLTRQRLGLAHREATLSPDVAQHPLGPLGTLLIVSDGMGGAAAGEVASEMAVEIVSREMGRQMSAGLASRDAMRLAADRANAAIWDRSQNESAIRGLGATLTAIHLSGHEAVVSQVGDSRAYLIRGGAIRQLTEDQSWANAAKKAGMQVANVPNNVILQALGTQRAVNTDITVEGVQPNDIFLLCSDGLSNKVEEHELLAQVSAAESLDAAAEALVKLANERGGEDNITVLIAQLLPSAAGEASASSRVTQVFASAPSSGDATIPFERGRVTAELHGGQTLSSLSVVTRQANASSDGLAPVNESAAGGGLSSGQLSPLPEGSPALVTEPVTPTAAPRSPTSGKLVVLLALVAVAGIVLAGAGAMLWILQSRAAAVRDLGKNPSPEASPSAPPPVAAPAPSPASPPSNPSSGIGRADGPILDEVEKKLELAERQADDLARRMEGLSVHEAERKACEEKCDQLKKLKEALGRHRRREAEPGDLSVADIEREVVAISKWLSELPEPLRKRKAPIGKKRPGTESLDEAIERGIQEMLPNALQWPGARP

>tr|A0A257RJ44|OS=Actinobacteria bacterium 21-73-9

MTRWRCAAATDIGLVRQTNQDAVYVDGALAVVADGMGGHAAGEVAAQIAVETVRAGYYADESVEGLVAAVERANAAIVRDAKENPERFGMGTTLLAVAITYDYAGASSPTLVHVGDSRAYQLRDGALRQLTQDHSVAEEWVRLGRLTPEEAAVHPRRHQLTRGVGVEEEVAVDVLSIDALTGDRILLCSDGLSNELVPDELARLAGDPADLEDAVEGLVAAARAAGGHGPSARTLAPASLHPAGPARSRRVPRDRGRGGRSHPLVRVLLVLPGRLPGSRRHLPGPALGHALVPADPPGGDALLHERASPARPARRAGDDPRAHPRRRPGLREEPVPRVAARGHLDDDHDDDPDVDLDDDHDEGPGLAVGRRLSLLAAAILVLFAVVVGQSLNLQFFRARALDASPLNPRNNTVTANQARGEIVAADGTILAQSVPAANGGYKRIYPLGSLMAGLVGFVSPYYGTWALEAQYNRQLTPHPQPPQSLAQVLAPTQASDNVVLTINVGLERVIQHALAGRDGAGVVIDPRNGDVLAMYSNPTYNPVPFTSSNYAVAAAAWKKDTTNNAHGFPPLGELATQQTFPPGSTFKVVTTSAVLLSKPQLLKKVYPAKTFIHLPNSNKTLSNYAYELCGGTIAEMLPPSCDTGFAMVGLDLGGNALSAAADAYGFNEVPPLDLPGVAASNFPPASAFTYDLPQLAYSSLGQQNVRESALMDAMIAGTVANGGVEMVPHLMKEITGPNGAVVTRFKKSVWKTPLSAAQAAQIVPLMVAVATSGTAAGIFPAADDVAAKTGTSQTGTVAQNTDDWLIAFAPASDPTVAAAVVVPYQVTSATGASVAGPIIACLIEGGLAVQAGHPAASSACPG

>tr|A0A257TPU5|OS=Planctomycetia bacterium 21-64-5

MTWESYVEHAALSDIGLRRSNNQDSITVVMAKSGELWLQRGHLFMVADGMGAHAAGELASKMACDTVPLTYHKLLDDPAPLAIRKAVEDANGRIHSRGQANLDFRGMGTTASVLLLLPQGAIVAHVGDSRVYRLRGNRIEQLSFDHSLVWEMMATGKLREDEVPGYIPKNIITRSLGPSPHVEVDLEGPWPTAPGDTFLLCSDGLSGQVSDDEIGAILSTFSPAEAVRVLVDLANLRGGPDNISVIVARVVGAPPSASIAWPAPGRSTRRPVHPVAWVVLGVFALATLLLLLARHPGGAAVMGLATLIGGATVLVRALGAQGRRLEGQHFGRGPYNPHTVLINNEFVGKLAKF

>tr|A0A259U0S4|OS=Rubricoccus marinus

MKVRSSSPGALPPTDVVSVTGRRGHNEDVGRVLQGEAAGMPVTLLLVADGMGGHAHGEVASRLAADALEGLWRRLLAALDAPEADSEAVARGFLRAAYAEAERKIGVEGEGNGMGTTLVAALVYGECAVLANIGDSRAYLVRDTSAELLTDDHSVVADAVRQGALTPEEAEKSPFQHALTRALDGSGDADPDLYPASGCIRLGPAAVLLLCSDGLSGVMEPADLHTHLTRTPDLTSAARALTASALDRGSPDNVTVALIEVGTLARAGGPPLATERVDALLAEAEPARPAEPLAPEAAAPAHEVRARETLGRSPWMLGALALVLLIIAAFLWLRRAEAPPPPARVPAVRGPARAVPSAFELSRDRTALTWRISGVATRSDSVRVTVSFPADTLTRTVEVVGASLPLAEVASAWPGGVLARGDYVWKVEARSLSGSRLRSGPAPLILDEPVYASGG

>tr|A0A259WYU8|OS=Rhodococcus sp. 06-621-2

MNKHLPLREARAMVPNSSPVPPAKIGIREALRTRIACAALWIVFVAVLVFGVGRIDFVRDNAALAVFLVGSVVPLMVWRFAGIGISDLKYRRERGQGLGSQAGASWTDREEQTEHDPFDPVAQSQAASPRRDDRNDMSRNLFDFHDERRPERTPAPQPFGPDRVLRQASPDDFSAPRATVNPDSLTVTRHVPLGGETDSVQSEMTDIGVPPASRVIDRGEPSTSTSTRGGALDRLFGRSTDRIDTEESTVGRILSFPGGGGGALFFDADSATDAGPRKENQDWAILSGSLLGIADGVGGRSAGGQASRAALRAVRDAIEEDGMSLASAVTRANTDVRARQDDDPADRGRATTLDIVHLDETGYLYGAHVGDSRVYILPARGRRLRRLTADHSTGNTLTRSIGGARSVTPDVWAHEAEPGDIVLVATDGLWKGSLHENDIESTLVEARQRSTIHIAERLVEIAKTGATDNITVIVGRIQRA

>tr|A0A292PPK6|OS=Tuber aestivum

MIPLCGSGCSLRLGMGLMAQGPSSAMRGLHSTASCTLRASGHFFRPPTRSPPPLRPKQPGSSGEPALHHHHHHTANHCQQQRIYIMAPPHPKTSTTFCPSITYSLPPPLSPMSPALTSVRTFSTKAVDHSSSASGSPPPNMTANAATSKKFTYQISAAYSAKQKRFSPTNNVFHYNPYNRVHDTAKRKSRPDAGQDAFFVSRINDTGAVAAGVADGVGGYIESGIDSADFSHTLCERIATAAHQSPTDNINAKYLMSIGYQKILEEDAIAGGASTACVGVAKADGQLSVANLGDSGFLIIRQGKVHYVSSPQTHDFNTPYQLAMIPKKLLVQSKQYGGGPLSDQPSDASVSSHSLRNGDVVVFATDGVWDNLSSQEILRIVSDEMVTGKGWIVGGEAGTVPSPQLENLTSFTEGQGLQAAVAKAVAARAKSASINMKVDGPFAREVQKRFPSENFHGGKKDDICVVCMVVVEVSWTFRSITLSNFP

>tr|A0A292PRL3|OS=Tuber aestivum

MRVTSTSFSTLSRTRLLAAPLPPRSISKRCFLTLASSPSSSTRDIPNPSTPSTAPTLSPRPTTTGITSSVSASTSPPSLHNHSHLRTPPKPPRVVPATVAATRPLVKPIQYGFKTSFSLFAKRAPRPFPPPFNSPPASSLSDALSTNDPSRIPTPEGATFLRGITNGDDAILHRHNHLGVADGVGAWNTKVAGHAALWSRLILHYWSLALDAQRKSPEAAGEGKIDVVSALQHSYNSTVSATTREGKTVWQGTTTACVSSLEGNILTIANIGDSRAYVYRPSSDSFVYKSTEQWHWFDCPYQLGTNSLDTPAANAVVDRVELEEGDIIILATDGLPDNLWDVEIADICSAAGGEEAGGLADKLVNAAWKIAINPFGESPYMERGIDEGLSMEGGKYDDISVVTAVFKKK

>tr|A0A2A2TMK9|OS=Calothrix elsteri CCALA 953

MEKDAATLYCPNQACQAANSLNHHFCHQCRTPLPKRYLWAVTEGKSLGVDGDILADRYLIIRQFILLDTKPGLVPSVPEMENLHKLKAYLRLFPYRLHVPQVYGVMYLGDSESQKQVLLLEKPPLQINNELVTEAQLYNSIDSAWGEATSMRQLNWLWQIANLWQPLASEGVASSLLDPLLMRPEGSIIKLLELHADTEKTSTLGDLGEFWRANLLHKTKTAIAEFLTQVCDSLISREIHSGEQLVAVLDQGLIELGRAENSSGGIFNSSVKIVTKTDTGPSRQRNEDSCYPPSGSKVSKPPQPTALAIVCDGIGGHEGGNVASNTAIEVIQQKVNSLTREPRDRIDPASLIADLEQAAAAANDKISQRNDIEGRQGRQRMGTTLVMALPIAHEMYVTHVGDSRAYLITRQGCYQVTLDDDVASREVRLGYAVYRDAINQGASGSLVQALGMGNSNSLHPTAQRFVLDEDCVFLLCSDGLSDFDRVEQYWESEILPILNTDSSEDVDISGVVDRLTDIANTQNGHDNVTIALVYYQIKYTEPESILIPNISDFYGSNIHDLPTNETPSRQFTSPNQRTQVIPDTAPATSKKIPLQLVILPILLAATGFIAYLFSSQRQFPFTTVPIVSPTKIVNSTASPTVVNDTKQANFPAGSLIQIKEKDAIAFSKTSKPFNNNGGFIPSGTILKVLETSPETSAPENQSNVLQVLVCQAVPIPAGPPAVDQASFVLKPNDKIWIQASQLKGSGFSKTQPASGTKNPCLQNKVDGTAPVDKSPNTSTPTANPSIIDTPKIDTNRPGNN

>tr|A0A2A5BG44|OS=Gammaproteobacteria bacterium

VSRGLASTFCGLVIKSTTAHLFHVGDTRIYLYRNHTLEQLTTDHRIHMPGEKEYLGRAMGIDYRLDVDYKAVPVEEGDVFFIATDGVHDYIDDEQISKVFEEEVDDLNKLCKRLVEVSLEADSLDNVSCQALKITELPTQNIDEAYSQLTQLPFPPELYEGVVLEGYRITRELHASSTSQLYLAVDVDTDEKVVIKTPSVNFEDDPAYLERFQMEEWVGRRIDSPHVIRTVEATRPRQFLYYVMEYIEGRPLDQWISDQKEPNLTVIRNIISQAVSGVRAFHRLDMLHQDLKPGNIMITDAGLVKIVDFGSTKIAGIADVSTPIERKELLGTKHYTAPEYLINGVGTSQSDLFSLGCIAYEMITGKLPYGHDIVKATDKASIKRLKYRPVSRHVSNIPDWIDRALQKAVNVNPVDRYEKMSELESDLRKPNPEFLIEEQRPLLERNPEAFWKGLAILSLLGNCALIYLLTQ

>tr|A0A2A9D348|OS=Serinibacter salmoneus

MSLTFSSAARSDVGLVRENNEDTGCAGPTLLAVADGMGGHAAGEVASSVVMYALREHSEDHTPEAITQAVERAQGALQAMSLVDPDLDGMGTTLVALAVGPQGVSLAHIGDSRIYRLRDGCLDQITTDHTHVQRLVEAGRLTSAQARHHPYRSVILRSIDDTHADLPDVNLTEQLADGDRVLLCSDGLSDYLTDAQLAGILSHGSPAVCAEALVTAALQAGTRDNVTVVVADARDADSPAEPAAPRAEGTEGSDVVTVGARVLDAPLSAAARAVLAETFPALASALTSGTDPETHGATPEAAAGQEPAAPQEATDRGGDEGSEVPAAARQAVRSGAAPRGREESRAAGFGAEALSAGNNGVRRADLGWIAFVALSFVATTATVWFTAGG

>tr|A0A2C9U008|OS=Manihot esculenta

MAACTSRAIIGECFWENLISRGGHLSFSTPNSSIYCANRNSHSYRNVTMSLNNRGQSTNSSIYGFVVYNVLKKCCDFSPYMEIGARYFHGSSPSCLSAGIAPDVTFENSARDEQLENSAGSSEQKRSAGKTLKLISGSCYLPHPDKEETGGEDAHFICSDEQAIGVADGVGGWADLGVDAGQYSRELMSNSVTAIQEEPKGLIDPARVLEKAYSSTKARGSSTACIIALTDEGLHAINLGDSGFIVVRDGCTVFRSPVQQHDFNFTYQLESGNNGDLPSSGQVFTIAVAPGDVIVAGTDGLFDNLYNNEITAVVVHATRAGLGPQVTAQKIAALARQRAQDKDRQTPFSTAAQDAGFRYYGGKLDDITVVVSYITSSEDEHQSS

>tr|A0A2D5EUJ7|OS=Myxococcales bacterium

MSNEGRTETEAPDEEEAPPEKSAEAGEGRDPSEEKEDVTATVPDMPAAKVAAEMRKRAEEEDEAADGDGDADDGEEEEEQEELPPEDLPPEDTGPEGPITFRYFGLTDVGLVREHNEDNFTVVDLATEEAWWGGEGEDANSPKEILEGELGPQGLVFAVCDGMGGAAAGEVASKMAVETIREVMLAGDPPQERDGFAHRLVYAIEEAGARIFSAAKMDRSRRGMGTTATVAGLVDRVLFIGQVGDSRCYVLRDGELSLVTKDQSLVNQLIEAGQLTEEEAEAFEHSNIILQALGTTEEVAVDLTFLELRQGDRLMLCSDGLSGLVHGEMIQEMMAEGDDLQAIAMKLVEMANAGGGHDNITCVVAEVGGEGLAAAEGAPAAIYQQYPLPPADGYDSEIPPREPTMKTATRKPGADVKRDPMQLEAGRADVPTKGFPFGMMLVALVLLGLVLAVVFGLDDEEPERPAPPPPTMEPEPEPEPVEVCVRTDVPESELVVDGQTISHFPESGAAICLPVQPGVHRFEAHSGGNSVVRASITVREGHPADVNLELPEGTFETPTEEDAGPGAEPEVEPEPEVEPEPEPTMETVTRMRPRMREQPTMTATETTETTTAMMTSTPMTSTPMETAPTMEATETADTEMAPPDNPFGP

>tr|A0A2D5F6K4|OS=Haliea sp

MSGPEQPWHAAGDTHVGRREHNEDALLIEPASGLVMVADGVGGHQAGEVASQITCEVLARELAAGQDLESAIRQANREVRDAVATGRGKSGMATTVVAAQFSGSDYHLCWVGDSRAYLWDGQLKLLTRDHSYVEALLEQGQITFEEARNHPRKNVIVQAIGLQDEDKLRVGSNRGNLPAGALLVLCSDGLSDILDCDRLAAILGSDGSLQARSETLVRAAVDAGGRDNVTVVLAERVTGAKDAGDAPETAAEPEVVWRYDPASGEYHGLPELVQPGLPEGAGGAAPVRAPQRQRVRPKSVESTQMMSADAMDGLRRETEKSRGPTRGRRRLWLLLTVLLGALLGYWYSTGLGG

>tr|A0A2D6MML7|OS=Deltaproteobacteria bacterium

MTRQTTDPVAPSIRSASDSDVGQARSANEDSCELLSRADGTHLLVVADGMGGHRGGATASSTAVTTIATIVEESAPGNPGDTLRHAIEVANARIYEMAQNDRQLEGMGTTVVAFLLDAPQYASVAHVGDSRAYRLRQGRLETLTTDHSVVAEMHRRGLISADEAAVHPRRNEILRSVGAERYVEIEVADVDVAPGDRFVLCSDGLSGVVGDDDIAAVVRSEPPDGAVETLIRLANEAGGPDNITVQVLSIPAATTPLELSGSSGRVTDESRPEQPKMPRLGMALGAIALAIGAWLLWQQM

>tr|A0A2D8IRJ3|OS=Haliea sp

MSGPEQPWHAAGDTHVGRREHNEDALLIEPASGLVMVADGVGGHQAGEVASQITCEVLARELAAGQDLESAIRQANREVRDAVATGRGKSGMATTVVAAQFSGSDYHLCWVGDSRAYLWDGQLKLLTRDHSYVEALLEQGQITFEEARNHPRKNVIVQAIGLQDEDKLRVGSNRGNLPAGALLVLCSDGLSDILDCDRLAAILGSDGSLQARSEALVRAAVDAGGRDNVTVVLAERATGANDAGDAPETAAEPEVVWRYDPASGEYHGLPELVQPGLPEGAGGAAPVRAPQRQRVRPKSVESTQMMSADAMDGLRRETEKSRGPTRGRRRLWLLLTVLLGALLGYWYSTGLGG

>tr|A0A2D9TEZ3|OS=Sandaracinus sp

MGGGSRFPGGGPRRAPRGKGADERLARSRASVTSFRRVRPTYVGTIPRHCSKVERRHRESPKNRRGTSVASVRGHDPPDPRPIVLVTDPIPAPRPYPPLHAHGRSVTGYHAVNEDSFLVDEVAGCFAVADGVGGAPAGELASQAVIDGLDANSGPGTLIERGHRMLRAARAEVMDRATGRSRGMASTLACLVVEGTRALVLHAGDSRVYRLRHGTLALLTEDHTVRNELLREGENADWLDDRAGGSLTRCIGSFPVPHHRGAVMAVVDARPGDRFLVCTDGLTKVVPHQVIANTLANNRVEGGVLELLAEASRRVARDDVTVVVVEVR

>tr|A0A2E0L148|OS=Anaerolineaceae bacterium

MNSTHYTEVHLIHQQTQQHTVIQLGGQFEHGNNEWVLDQTGVDFARFRFVAAADGVQLQYLEGDSVVEVDGTAVTQSAVIRDGATLRIDDETFRCELHQQRYEATQPELDAGWLTITGSVREHNEDSIGIYQQPPYHLFVVADGVGGAEAGEVISEFAVKYLLYEFDRYRDTQTDWASVFHTAVKDINDEARSYARTLSEQSGRQVQAGCTLTAIALNGWDAQIVHVGDSRLYLQHDGTLQQVTVDHSTFSTANAGAALQTTAKFATKRNVLIKGIGKSDQIEPDLKSLRLTPGDKLLLCSDGMSDRINDAEIAGLLDGMPPQKLVAHLAKTADERRSADNISVIVVRVNAPGQVVGGAQALPQPRAYIGAQPRPRLSAGVDLTTDYSTETGESHLPLNLIIPVAIVLLVVIIVGLVLVRAAG

>tr|A0A2E3GNH1|OS=Saprospirales bacterium

MSKFIHSTSETDIGLKRQANEDNYGTQSTVNGEVFIVCDGMGGHVGGATASKIAVDSIIQYLTSEYYENIVIALDKAVEFANAQVYGEAQSNPDLKGMGTTCTVLVIRDYKIYIAHVGDSRIYIQNDGKLKRLTKDHSFVQGLVDKGIIKDSEAEDHPRKNELLQALGVRPEVEVTVAQEAIVPKKGDKFMLCSDGLCGLVNDTDMNQVIQRSAYLETAAKDLIDLAKAAGGHDNITVQLIEVLESPSLESTFVDLSPSEAAPSVINANRTTAIEDRRGQSSQNKKSSRSKPVLIGVVSLLITGLSIGGFFFFMNSGVDDKAKQEEVTTGGLEGGEDTTSNIKGDSYVDIDYSYRVVIDLKKDSTDKGKCKLYDSNGKLIGPITPEDFTFSWSKLARVLGNTIKEAKKGNSLCSEACYKLSAHGSYQPLCEYFRNNGNKKSLEGIDEIFVTEAAWADENQKSCCIKERSTVVIERRTSGDGTSTSGSTKTTSGSTTTTTSDGTTTTSSSTTTNNDDGTTTTSSSTTTNNDDGTTTTSSSTTTNNDDGTTTTSSST

>tr|A0A2E5A021|OS=Opitutae bacterium

MAGKFSYQVGNHSDKGMVRKNNQDSFGSATNAWGEIYIVADGMGGHKGGEVASQITVSHITNAFKKADANEKPIDFLERTIQEANQLVLEKGKKDNELEGMGTTIVCILMVDDIAHIAHVGDSRLYIFRYNNPYFVTKDHSVVQDLLDKGLISEQEAEDHPNKNRILQAIGIGKISTSITIEKLYKGDYVLLCSDGLTGEVSVPDIFSIIKSNKPMDATEKLIDKANNNGGSDNTTAIVLKVDKGPNPPKQKVLSASHRPTTDKKNVSSYMVLSFIIGSIITLLAIITYSKVSDLLIKDEPIIDIDPVIIEEEQADQSEEVEEPKEEEKADQTEESAPSKEEKVGDIGNKSSNPIVNTNQDTSKNDTVKKISKDQTSKKKK

>tr|A0A2E5HM07|OS=Myxococcales bacterium

MSSRKPKATRMSVIELSDEGSRDHNEDRGLALSGRSQHLLAVCDGMGGHAAGEVASGLAVATFAKLFANDYSPESPAAGDFLEQAINAANQAIRMDAKENPSREGMGSTCVVGLVRGRKLSIGHVGDSRAYLVRGGGIRQLTRDHSFVEEMVRAGMITPHETHTNPQRNVILQSLGCNDNINIDVNENTVNLKVGDYVLLCSDGLTAVVADQEIAETVERLAEPDLIAKDLVDLTNRRGAPDNVTVVIGRFDGDLIEGPPIAIMCEDDTSIDLYRLILQREGYRIQVHDCNTVDFDFQSSEIPALVIIEHEDPKVTLSLCRDLSNKADFKKTPIIVVTDGQIPAPQAKGSGAADVLHSKALLAELLPKIHELINSNSPKVYILEEEPDAAKAVGQSLSQANLTVRTFNTEHDLHDAIERNAPHLLIVHMGASPGLERICRRLKTTASIQNAPIVLTAEGAVANPREAYLLGADYYLPNVSDLLNLTQMLLAKAR

>tr|A0A2E5HMU1|OS=Myxococcales bacterium

MSFEDRLTFRCETDVGVERSENQDHYGTFSPDQVGTGWLFVVADGMGGVAGGQEASRIAVAAIEETYRALMADDGTIAPFDAISGGVKAANQAIKDRIAQEPGLRGMGTTAVVMAIIGDLVYTVHVGDSRIYRHRDGRLEQLTRDHTRVQFLVDQGIITPSEARKHPDGHIIVRNLGGNREVDPDIPHDGPFRIRDGDIFLLCSDGLYGLVSDEAIRQILSYAPPDACAPALIELANRRGGHDNITVSVVCAGTHPESWAEFDPRKLARMVDALALDDTSDTALFEAYDPSALAPVDFETARLEAVSAEMVTLDRGASEPGSAPTPAPAASDPDPTPVEAAAPAPPAAAPEAAPPAPAPASSSSSGSSGRGLLIAGVVGFVLLLAVVGALAGAFFYQDQLMEMFGVEDEESDKKDKGKKKRKKSRDKSDKSKKSGQLPGRDVGLAAVTFGARADLLGATAPREGGRALPTLRL

>tr|A0A2E5HPX4|OS=Myxococcales bacterium

MIGGLTYVPLSDIGVQREENQDYQGHDLTPQGFLFIVADGMGGHAGGATASQMAVNHTRRAFSVMEPDKPVGALKDSIQIANRAVWSMAAQRPELHGMGSTIVALLVQERCAYIAHVGDSRIYLLRDHKLHQLTKDHTMVQRLVDEGVIAPEQAEHHQKSHILNRSLGGKNWVEVDAREEPIELQEGDIFLLCSDGLTGLVDDDEIGRILVHTELHQAARILVDEANARGGFDNTTIALVRIDSLPLDVRVRSSYPPPEMFEPPVKPTPKAEEPEALDGSEPEPEPDEATSEVEEAEEGAAEPSDDAADVSEEDASEEDAADDSEASKEDPRGWFQGDEPVAGEGDEEASSDAAPDEASEEAEDAKQSDEGGSVPDEGVEAPEEGAEALEGDEAAAADEADVSEDPSDSGDGEEELDESPLVGRLPPQVSSEPIDDDGVGSYRVTKLREGMPALLYTRPSFPRLTEPEPPPAMSTGTMLLILVMGLSAGLLLGFVLAQTLNKAPMP

>tr|A0A2E6EJI9|OS=Rickettsiales bacterium

MGTANAHQQSRYPAPPRTVKRVNQQSRFQSAPRQQIQKPKWQRAESRDRSGHFWLCFAFPQHPSEMKQMSGSHDPQHNVDTARMPSTGRINTSMKPADCSQVAGLSDVGMIRELNEDNWYWGALNETLSLYIVADGMGGHDSGEVASELAVETLFSNSQERVAELNEVNEESLRDLLRSSFEEANRVVVTTGMEQESNMGTTLCAILVNENKDVVVGNVGDSRIYLLRNGVLSQISQDHSLVAFLVQLGELSESEARDHPSGNILVRSIGSMMDVEVDLFHLKAQDGDRILLCSDGLWGEVPDDELTSLLLEHNNPSKACTALINAANEHGGKDNSTLIVVDV

>tr|A0A2E7C4K7|OS=Haliea sp

MTTLDTAGFSHVGARRHNEDALLLCDDLGLYAVADGVGGLHGGEVASRIVCDTLEAGLRAGLSLSAAMDRAHAEVLSAAARGEGVPGSASTAVAVTLDKARLQLAWVGDSRAYVWDGELQLLSKDHSLVQTLVDTAQLDFAEIEHHPQKNVISQALGSRSGTPVTAYKEGSLKTPCVLMLCSDGVSGELSERRLIDTLASGESAEACARSLVEAAVGGGGQDNASCIIIRVPKMGLPLATESTLAFMRYGADNRWHLAETVAGGDTQMNPKVADHTQTLALPAKAEQPPAVKKKLSLLRIVTGTTFSVMLVVGLIYALERTTALQSILALIAHD

>tr|A0A2E7M731|OS=Deltaproteobacteria bacterium

MEADEQPQKPRPTDSEHPGSTRTRQTGGPIHEVRPDRRRARDSWTATAARTTPRGPETPVGRPAPHLESRLRRAMGSLLPRPNSRGPADPQRPKRTEKPVSWNLSGASDSSKSPVRTPSGRSHDCQRSGSPGMVLPSFEQRQMTMATKKTNTSTKGTSPQGEAHTNPPITTSGSVAGVGSVHSLDDTTLIVQPPESNEPVSAAGQSDVGRVRELNEDNWHLGALNDDLMLYAVADGMGGHDRGEVASKLAVQTLFEAAREGLEDLSDTEVTTLRSLLRKCVQTANERVVITGIEEESNMGTTLCAALMHEESDAIIANVGDSRVYLLREGKLIQVSDDHSLVAYLVRLGELSAEEARNHPSGNILVRSIGSVPHVEVDLFHLPVQQGDRLMLCSDGLWGEITDEELEAIMLDHDDPRAACRALVNVANQNGGRDNTTLIVVNV

>tr|A0A2E7PK51|OS=Rhodospirillaceae bacterium

MHRFAFGLNWKSYTVGSPRSIPCGNRPGAVAAILLAPSCGETYLYGPHQQLLGGHRVAHEPRLAVESIAGRREYQEDSLVAQQLSDGRTLVAVADGIGGHAAGEVASALALETLVASLEEGDALNEAFVKANSGVWQKAREPEKKGMGTTMGAALVDDGEYTIANVGDSRGYVLSADGIRQISLDHSFAAEAARRGQPIAEEVALRYRDALTRSIGTEEHVEVDVFGPFPVEDHTAFLICSDGLYKVLDDDALYRIYVQSSGPREAAQALVNTAFGDGSDDNISVAIVEYGELPRAALEDTMPTGFVASAAEDDAWVVATSAYAGAVTTPREVRIGPVIVVLVMVGVLLAIFVF

>tr|A0A2E8K8E8|OS=Gammaproteobacteria bacterium

MAILSIAVGMALCSRIMDNYFLSEMSIVSIEEQSLHQYGAGTHVGKIRDNNEDSYVCDGERGLWVVADGMGGLGFGEVASAISTYTVTKMIAEGHGINQAIELAHSEIKAYAESDGLGTNMGTTIVLMLSHGSLYNIFWVGDSRAYLLDGDDFRQITTDHSLVQSLIDQGELTQEEAITDPRKNAVTRALGVQELETVRADSLSEKWQPRQKILLCSDGLTDCVSNSGIQAILSEEGTDQELTDRLIQAALEGGGKDNVTVIVVSSPSSATVGDSDTHVPEGIKDDTDRPVDDVTEIPNSDDWEDPRRKHERDQHSSAAQLELVLPEASEPGPPLAGLPEPIRKQTTEAKSTFSNRARGIAAIAACLVLMIFISLSKTDSGERPGKPEAFSQQEEVSQYAGRSFPAIDLPENGPVIQVGVFTRLEGAEKKQIALSQLGLESYVQKRSTENGLQYAVLMGPLSAEIHQSTVATLTANNLSYFHRPTRGS

>tr|A0A2E9QQG0|OS=Deltaproteobacteria bacterium

MSKATPSPSIEVYQSTDIGRRRHENQDSMGYIKLSQIPYQDAHLLVVADGMGGASGGQVASSIAVREIVDYFQDDPNMEPDVALREAIELAASRILERAREEPSLTGMGTTCVCVLSLNGFVFAAHVGDSRIYLYRDGVLHRCTRDHSAVQRLVEAGILTEEEARNHPRQNVLSRVLGSEHPLYVELMGPPKKLLPGDRYILCSDGLHGEITDEKIIELVEDGPLDKITDNLVQAANDAGGNDNVTVQMLAFGESAPLEMAEEWQEFDDQVGGYVYYATMVVLLFAMGYLAGWYSHEESPARRQLAHNTTKRHVVHHPPAREHQSKPDERRQTTIAGRTPARRVPTQSRVRQSTKSRTKAPTRSMVQTRSKAPVARCEANAKAGSTRCVAPTKRSAPSTKPAANTRRTPPPARRTQPPVKRSTTAPAQRVAPAKRRTAPPVRRVDARKAPSSKPASVPASQPTKPPSR

>tr|A0A2H0UL64|OS=Candidatus Harrisonbacteria bacterium CG10_big_fil_rev_8_21_14_0_10_49_15

MNRFEDYDTPKPAPEEEPSAEIKSPQALEQGPRPRFEFAMKTEAADYRNGDPNEDAVLADPTRGVFGVFDGIGGHDGGEIASQIACEKIAEALAKDPKPGQGVEEVVKNALIKANTAVLEAPRQGKGKYGMGTTASILFLLPSRPGKKQEAVIGHVGDSRIYQITVKGEIRQVTTDHGYAGYLGSATEEEIKKELVTRPKQVGARGLSQDAQNYAEKRNILIQAIGSRAIFPDVNRVSVQPDDIFVLLTDGGHDPLLINDVLPEIAAAARTQSPQAATALLMQKVRESRQAETMISKDDDTSVLVVRVQVVEAEPEPETLTMDANPEEIKNTAQTMDRYRALEKEIEKAQSFHELYIALESARSAKEKEILNPDEAADLVDSLRQADTLDAGRVPETAGLRAKVVALLESGAGIREANSLQQLKTGLRWAKDVASEEDKTTARGLSEVVINRKQPALARQLGGLHKDLEEVVIRLTLHESTDFEELYRMLRDLKTIRSKSREYAAEAVIAKIQAYRDRTARDDMPQRDTITNAYNLRELAQTLLLKERRSS

>tr|A0A2H4SGP2|OS=Cordyceps militaris

MAMQLLLIPILFASTASIISASNILGLGSMGAALNRALSYNIITMSCSLFQNLYEFGVCQARHCDARQWYVTLCSGSPRPVCCNPICATRSETTIAKKKAQMKGLLTRRTAARLSCARLLRPLSAESRRTLTTPTTTMAPPSNMPRRCYSSAAGPVSSSTPASPSTAATHPPKKPRFSYHIAASFIAKDRPFDPSTHVFHFNPRRRSARPDSGHDAFFASRVHETGGAVAFGVADGVGGWVDSGVDPADFSHGFCDYMASAAWEHQPPSSSPAGSSSTLTARKLMQLGYDAICADGSVRAGGSTACVAVASPDGHLDVANLGDSGFLQLRLNAVHSYSDPQTHAFNTPFQLSIVPPSVAARMAAFGGTQLCDLPRDADVTQHRLRHGDVLILATDGVLDNLFNQDVLRIASRVMGATKAWTRGDAGQVQVAPDLDAIVRGPIQAARPAPGRQQNPDRVVTLQSLLATELVLAAKRASVNTKQDGPFAKEVQKYYPLENWRGGKIDDICVVAAVVVEDPVSTPSKL

>tr|A0A2H6EI24|OS=bacterium BMS3Abin03

MNYSLEIGNFSDIGKAREINEDYFGTFSGSYGRLLIVCDGMGGHEGGEIASRLAVETIRNYFENLGTDFNPDEGIKNAIQKANEVIIAAAKTKIELSEMGSTVVLVLVKDGYAYAANLGDSRIYRIRNGEIQQITTDHSLVQQMVDSNVISPEEAKTHPKKNIITKALGIDEIAKPELPEPFQLMVNDCLILCTDGLTNHVNNNEIREICASNNSQDAAEQLVDLANERGGTDNITVQVMKVLEKAAPAEHTNKKSNIVSYTILLISIAALVFILFSLDVINLSGNPVTKQFDSGNTPSPAKFDSSGKTATGVINEVTNNDKTDSNLVKEDSTKINNEEEQQNENNSE

>tr|A0A2H9TID1|OS=Paramicrosporidium saccamoebae

MQLPFLLTLLAAVNCSSESISALHNQQLQTVLDDLIERNDRPTETDWGYFREICSNRKKQRHCAEIFDYFHTVLVPLEFPQVPTPPVMWSDWYGAAMPKHGVVPHYFRDTRTTQTFGDDSQTIEGDFMIVSDGITRDPDSDFFSFQIVEFLRVTLPRLNRTVSVEEELYRAAKAVEELLVEVRLPGAATLSAAYRRDNTLYVLTLGDSEARVVRDGQVVYRSPRQRTGRKHEQLAAHRPDSVTRMIIDAIPIQADDYVLLATDGLWDNLSEADVVAEVVGTVKVAARSVMERALQAKEGKRQCIRSKDGDKIDVEECVGGRRDDITVLIAKV

>tr|A0A2H9ZQN9|OS=Apostasia shenzhenica

MLVFRYPEDLVDRIWHVDREEADFGRRNNLESFNISLRGCNTSVPLKVLQSATDDNKQLVFLHEGLANDQEEFLIILHFLELDGNVHTGERVFDVYVNGDKMHEKFDILEDEKSSNYRVFSVRVKSGGYLNVSLVNALDAAKYGPICNAYEIYRVLQKGVETIERDVDAVMKLRDELMVENSKLEIFRNWCGDPCSPTSWAGLTCEDHNDSGFLDRSIHHPIKSPDVKTYTLEYIKKATSCYQTLIGEGGFGAVYRGTLPHGQEVAVKVRSATSVQGTREFDNEVIGQVNLLSKLHHDNLVPLLGYCCENDQQILVYPFMSNGSLQDRIYEVHIMGTRNVLILELNMMFICCQDIVLKRELSSSRTQFVAKPFIREQRIDEMVDPQIKGSYHAEAMWRVVEAALSCIEPFSAYRPSMADIVRELEDALIIENNASEYMRSIESFGGSNRFHSIDRKIPGLALTPSEQSSGFCQIITGPQPSTYTLLLTEVETSELQSPPPYKSKIIIPTKRLGAPERSLYVVHVFLGSQRLCHPFNCQYFRLSRFSASFEFSVFLFPSAPRRCPSQTLTILRLLSRQPPGFGLAFAESWSLVDQSLKLLSGSCYLPHPDKEDTGGEDSHFVCDNERIIGVADGVGGWANVGINAGEYARELMLNSVSAIKDEPRGLVDPAKVLEKAYLNTKAKGSSTACIIALTDQVLLRTQTCDLLVKGGIRAVNLGDSGFIAVRCGSTIIRSPVQQHDFNFAYQLESGNSRDLPCSAQVFSFPVESGDVIVAGTDGLFDNLYNDEIKRILVRASRVRLGPEVAAHQIAALARQRAQDKDRQTPFSAAAQHAGLLYHGGKLDDITVVLAISSFTSLQTLQKRRRPDSETLEIPTVMENGLIAVFLLLSSPLLCASFSADNPTSRRLLVLVDDLALRSSHSIFFNSLQSQGYDLEFRMADDPKLSLQRYGRYLYDGLVLFSPTVQRFGGSLDQAAVLEFVDAGHDLILAADSSASDLIKGIATECGVDFDEAS

>tr|A0A2I0A2R3|OS=Apostasia shenzhenica

MLSTFSCLPSRVELGMGELVNSSLMTLRCKTLPCHQVRLLMANTTYKPPSRCCRIPGNCHEFVNGLSFKQIKLDFGENSILVFCQRSRPTCIIRRPPLSCCSRSLHYQRIITGCLKNGEHWGKNMLYKCFWSDVSGSSWKSYNPTEPWFKNFSLASTPCSAGAAPHDVSLDGIPHGEQLENPGASSDRKYLGDRTLKLLSASCYLPHPEKEETGGEDAHFISNEQAIGVADGVGGWADLGVDAGQYARELMSHSVSVIAEEPKGSIDPARVLEKAYTCTKTRGSSTACIIALTTQGVHAVNLGDSGFIIVRDGCTVFRSPVQQHDFNFTYQLECGNASDPPSSAQVFTFPVMAGDVIVAGTDGLFDNLYDNEVTAEVVHAIRAGLGPQVTAQKIAALARQRAQDRNKQTPFSAAAQKAGYRYYGGKLDDITVVVSFVATSSS

>tr|A0A2K1L6E2|OS=Physcomitrium patens

MDHLQLSLIPVAMATPAIAVRPHARLHHHHQQLVGPSRLCAPPGNGWNHLSRIRIAPVFPCVQFHSYSCSHQLVAYATSASQDQTALESTSVELAFAVGATMTPHPDKVQKGGEDAYFVSNYGGGVLGIADGVGGWAEQNVDPALYSKELMAHAEAAVSSEEMEFNAQMLLAKAHAATNSIGAATAIVALLERNGVLHVASVGDCGIRILRQGRVVFASQPQQHYFDCPYQFSSEQSGQSAADAMVFKAELKEGDSIVMGSDGLFDNVYDRDVETTLSVFGGSDEESAIRSAKALAALASKNSRDPAYESPYSKEAIQQGLDVPWYKKILGQKLTGGKMDDITVIVAHVVKEQKSPDEAIVEMRSEPVPQMQDVDGETSVQRRDDEEQLIPDGLSEGWA

>tr|A0A2K2H5X1|OS=Geothermobacter hydrogeniphilus

MRSDQEVFVAQLTSGHATDVGRARDHNEDSYVARPELGLWAVADGMGGHAAGEVASAIAVEVVAAQVEAGRDIATAIQAAHEAILQAGQEGRGESGMGCTVVALLSRGLDYQVAWVGDSRAYLWDGGTLIQLTRDHSYVQQLIDSGILTEDEAREHPQRSVISQALGIGAVDGIRVDTVAGRWGRGQQILLCSDGLTGEVEDTEIAARLGEREDLRETVAGLIRAANDNGGSDNITVALVAAPDRAPQIVPKGGTVPFDAAALNRVVAPKKKLFRRPLLLLILALAVLVAGYFAFRPASAPTPATGGSASGVLQVPADLSSDSGTRAIGPTDSLQQKPLRQSPQTRSVSPVGENSFVTQNVAPREGEAAALPTVVAPDASNDENPSVVAESTAEPALTDADLLREVKQVGAKPVSAPLAPVADNVPQLGRQRTPPKGDLSQGLNEK

>tr|A0A2M7ACW1|OS=Armatimonadetes bacterium CG07_land_8_20_14_0_80_59_28

MKFGKCTDIGKVRSANEDSVLIDEKLQLFVVADGMGGHASGEVASRLAVESLQRFLLERMGSGAQATTAEVARNVREAFECAGDAVVQHARRTASDMATTMTVAVVHGRELVLGHVGDSRAYKISDTDIRQVSEDHSLVAQMVRDGLLSPEDAEQHPKKHIVVRAIGPQVENSSPDVTIVPLDESDSILLCSDGLSNVLPPDEMFNIVQRHDAPDKAAQALVDAANAKGGPDNISAVLIQGPWDTPGAARSRSSHLPLRGLLIAVALFALAGLGRAYVIHHFFYLGVSDGKVAIYPGLARFPGILATREPHRVTKTPVEECLSPYQDRLVRGIQFSSIEEATRGIAGLQSKPPLGVSPFPSSDSAAASDLPKTTIPDPLTPPR

>tr|A0A2M8NW69|OS=Phototrophicales bacterium

MADFSKNLEAFGLSDVGRKRKRNEDFHRFVIPPANTPEERLGAFFVVADGIGGMGFGDEASAVATNTLLAAYYDPKNPEIDPMKRLEVAIQVANNAVIARAGELGRTLIGTTAAGIIVHPREVVLFNVGDARVYRIRNNSIERISKDQSVFEEQLSLGLVTEEEAAQGRNMNVTAFIGQPGGVEPVYARVQNLSADDIFFICSDGLWDLVKEHELLHIISKNPPEIAMQQLVAIILDRGAPDNLTGIIVRLSPFKKKASPLVPVLVALALMIGIAIGVVVVGNMNSATTNDIIQTTDSTDEPPLSIVLITSEVVEITPDETDVPIDIITDVTEMPVNIVTADSTSEITEAPLSIVATDNTSEATTDSIPTVVSVANLPTSTPIVIIITATPMPTDTPSNTPTSTNTPAPTKTPTASPTKTATATSTVTPSSTATFTTTPSATATPTATATSSPTQTATRTSIPTETYTPAPPTATLDTESLTLLAPFTATARANLPPETQTAMSVQETQAAIDMANTETRVAFEATLTVVYTQTVAAQTLQAVQTSVIGTATARAEYTPDANITPPAIENLMVVVTDASRLYQLPDARSQLLVPLLFGDELTVLGVDRSGTWFYVEIQNQIRGWVPRFPDKQLDAYVSGTPIETLPILNSETPSAPSDETLGVHLIVTNDVAINVREYASFDERGNIFAGLDPFTVARIDAISRDRQWLRVSFTRNNRLIIGWVSALLAESNEIVIVGDLTTVRAITPLPLPTMTPTPTPTEEPTPDVTPTE

>tr|A0A2M8PQX4|OS=Phototrophicales bacterium

MIDTSHTTAFAAADIGPREILEDYSVTEEVFTMGGLHLRLAIVCDGVGGAASGERAAFITAQTVIEFIRNSTDTIVPRLLTDAIKEANRKVYNDSTAGKSTIAMIAIHYDGSPYGRAYIASVGDSGIFLIRDGKVRRLNTEHNVANDMIWEGRATPEQAFALPNAWHLTRAIGVNPDVEPDIGFYYNATNRQEARQRGKAGILLEEGDTIWACSDGLTDSGENGIPYVTDEDFLRHALDDNVERTARMYLTYALRSGTHDNVSIALVFVDSKKRHSTDPNARGRISMPLIVAFLVLLLIAIGGSAFFFSTQSNEEKGALEEQIVAAEQTQVALQRAVEEATQTAIAIQSFTPTPSATFTPSPTFTLTPSPLPTLEAGNAGYSFAGRNTLPEVVAIGQVVEAQEAPIYVSAFVGEQRSDRFSYYYLFPQSKVRFFDVTDTKVEFYVRLDSDVFIQNQGYVEGTEVELEQLPAARFSARNACMSIQQTEANIAITCFSGTCEYTFERGGDYNLLEPGQTMLVEIEENNELSVREVELDIDEARRYDELLVEFSAPGAVIGKSCAGVWIPEPTATPTPTATNTRRPPTPTRTPRPATGTDDDPDNDGVPTGFDNCPNNHNSDQKDTDGDGKGDVCDGDDDNDGIADGLDACSKVAGVASAQGCPDADGDGVRDSQDNCPNTPNPSQANNDGDSQGDACDSDDDNDGVPDSSDQCPTQAGQASAQGCPDADGDGVRDSLDNCPSVSNSSQTDTDGDGQGDACDSDDDNDGVPDSSDQCPTQAGQASAQGCPDADGDGVRDSLDNCPSVSNSSQTDTDGDGQGDAC

>tr|A0A2N0VE51|OS=Rhodohalobacter barkolensis

MIKKISIFGITDVGRSRTHNEDDFAICKDLSNHKWGFKRGEVHSLSELGALLIVADGMGGENAGEVASHLAQETVKEDFDALDELPDSVSEREGLLARSILKAHQAVVNHQHKNLDTAGMGTTLIIAWVVEDSVHVAWSGDSRCYIYNGYKPLYPFTEDHSLVWQMVEENHMTPEEARVHPESNIVTQSLGDEKNPPQPSVKTTKLYKGNQLILCSDGLNGMLSDEQIESVLEDGGPIDETCGKLIKAANEAGGGDNITCLMLEVHEGAEPSEADLAAQSQIKSKTTTTQILRKKNSSKSLMIGVLIVVIAVLAAWIYFDGRSPEANADPVIQDRDTGQTEEPVDSAETNQDPPIEDEPGDVDPPEEEQPPVADPDPEPAEEPSETPDEQSPLPPGNAERPSEEEKDSVRQQIPPVQKDTTLTEPGKKIQEPKDTMSTQPALPDSTNADSTADTTSTGSIEKQVNN

>tr|A0A2N1PTJ2|OS=Candidatus Wallbacteria bacterium HGW-Wallbacteria-1

MKISASAITDVGRKREHNEDNYYSCPEHFLFAVADGMGGAAAGEVASALVAETIGITLPAALMSAEKPDELTDENLERLLAASIEKANFRIQEDIRRSPEKAGMGSTCVLMALREGKVHMAHVGDSRIYRLRNNVLTQLTEDHSWVNESLRNGLITAEEAVNHRFKNVITRAVGTKETVQVETQTKFSLPGDVYMMCSDGLMAGKVADSTIEEVLRKNFNDVNASARELINLANQGGGPDNITIIVIRVDETVTDPAFSPETPQVTRGSGKKAVLLALTGLLLLTAVGLVLFKILTFSPPPTPSMDEFADLTSEKNLMVSGKSIPSSSVEILVNGASATSIQTSSDGSWKAQIPLTADGQFSISARTVDASGNTSPESPVRIISRDTIPPAIESMKIHSPANNIVTGTDSLELSGSTEPNTTVQVMLDDISLKPLTVDEKGFFKGLVEIRGLGDGEHNIRIRPMDKAGNIPADFMVIPFEYDTEAPPLPDFITPSGDTVNTTSLTVRVKAREAGKVEIQLDSNPFKDAELEREDVFKMELFGLQEGREHTLRARSHDKAGNKSPGERVVTFTVYTTRPPAPKVDSPTQGGITRSPLKIMGTASPGSRITLLVDGKQAAMIPTTGEKITWEAEIQGIADGNHVLKAQSEYHGAISLFSDPITFTLDSIAPPIPVPNGGRSLLVSESPFKLSGTCEANARVFITNAAPGSKTFETQCSSSSEFQFGPVSLEEGKNKFYLRSEDMAGNSSAESIIMIVTLDTVPPAPPVLEKMKDFYLKTPVLLSGHADTDSTIIFLVKLDTGKDVRFETTVSHNGQLDPVSQNFPMGKHTLIYWAADEAGNESTRKTLPLKIIERLPATPYFPDMGKTVFLDSSGKARISGRSGPGDLISLSCGNTGSTSCQADDGGRFQLDMTLPIGQIKIEAFASLIDDPAIRSEKSTLELTVRMAESGRTSGNSADPAENSMLNGSSSWHVPRPSGEVDINSASAAEIATALDIPPAVAASIVAIRTKNGSFTGTSQTLDVPEMTNFYQSVRSYMKVSSTQKSTPSAENGQSLQNRIVRAPVINPGDSISSTIKATAGKTISSLYRFRPAGRGIYKFIFNGKGLKFEILTTNPITPEHITTVGKTGSSQMGTWDTSASSTLMPEAILIKITASTDNSGEVPFEIKTTYTSIGTGDGF

>tr|A0A2N2IJF4|OS=Deltaproteobacteria bacterium HGW-Deltaproteobacteria-17

MRSAGRRARSATPTDSYHIGSGFFHGIRLLSPHVAQAADVHGGILTHGIHSHTDIGRMREENQDSMGTRVLADDFTFLVVADGMGGYAGGSVASSLVTSTMLSELANVTMDAFRENPQLFLRTAILSANAAVNKEARANHELRDMGSTVVAGTVIGDHAYIAHVGDSRAYLLRDGSIRQITEDHTVVQDLVRAGYVNPEDAVYHPSSGILTRCLGQMDMPDPTFAEVIQLLPGDRILCCSDGLSGMLEDEEIAEVVFHEPPGDAVNLLVNLANEAGGFDNITVILFAYGDLPEAARNWPIRVGTPLYRESLTPRTLDEPTVPFKFATRDKAKLHDTMAMGTASGVPGHSPERMAWLWPILIGLAIISIGLFIWYFIGTGS

>tr|A0A2N2IWW9|OS=Deltaproteobacteria bacterium HGW-Deltaproteobacteria-20

MPTGSIVILAVMVAALVAIYLLWRPRQPNPPVESGASPPPPEPSPVPVPARPETPAPTQSIPDLSGTLPKERESDPEITMMGKVPAEVLALMQGKLSADVPVEQEESYEIDVEELVSETAEEETTGKHALILVVGVARSDRGRKRQCNEDAFLILEDQHLYVVADGMGGHNAGEVASQNAVDAMGDCFKRASFPGEPRPDWPRRGDELVRSIHHANQTVFELSQTQEDYNGMGTTVVAVRFSPNKQRAYIAHVGDSRCYRIRQGELHQLTEDHTLGRLMGATGKAARHLAQAVGVRPDVAVDLTVDEPQPGDHYLVCSDGLTKMVPDARLLEVATEDENLEERCQQLIAEANERGGRDNITVIMVRVESPTKEARRVQDGES

>tr|A0A2N2IXG7|OS=Deltaproteobacteria bacterium HGW-Deltaproteobacteria-20

MVLPVPQPPRVSLAVASMTDPGLDPEKQVNEDACVARDLPIGHLLVVCDGMGGHVGGREASEAAIRTILEHMGAVAADVHPGASLKAAIGHAGKVVYDLGGSSANLLRPGSTVVAILSHAGGSEVAHVGDSRGYLIRASQIHPITRDHSMVQQMVDAGVLKPEEAIGHPDANKITRALGMSATVEVEVRATPLAHQKGDLFVLATDGLSDLVMPPEILSVTLQARQARGLDFACQQLVALANSRGGHDNITVLLAEVLDCPSCSEARSRTVVTDDPVPADAAAMTVPLSPGGLHPTMVDDGAEVAAGQPAVEPTPTWVGDEPSERDSEAEYALEALRRRKLTWLIVGTALVLFGLGLVVTSIWWAWAAAAMREAEATERLAIAQVSDPAPGIPAHRDVTVRGLPARSRPGAEQTLLKPVPWEAAPTRRRPGVRKWFVS

>tr|A0A2N2T638|OS=Betaproteobacteria bacterium HGW-Betaproteobacteria-18

MRFSVFQLSRQGGRKVNEDRMGYCYTRDSAVLMLADGLGGHPEGEVAAYLAVETVANLFQTMAQPKLTDVAGFLESAMMAAHHRILQYAMEKAMRDAPRTTLVIAVIQSGHVCWTHCGDSRFYLVRQQQLLARTQDHSFAERSRHNRHQGTTAPEPTEPNRNVLFTCLGSPVKPIFSRVEPLALQQGDTLMLCSDGLWSSLPEQDIVSELSQKTVATAVPDLVDRALLQAGDNSDNVTCLALSWQTPGPLEHGQCDIATESSHDVDFASTQRPNWLKHTHGEPNNLSP

>tr|A0A2N3IIG6|OS=Raineya orbicola

MKSSTQELKNFEFGNATDVGKVRSNNEDYLGYFQTPNGHLFVLCDGMGGHQAGEKASQLAVESLKDFFNKEFYTSPQEALRKAIVEANWIIFSTSQKFSEYAGMGTTLVAVLIRDNQVFYAHVGDSRLYYFEQATQTLHRITKDHSFVQELVDRGLIKDEDAEKHPRKNEILRALGIHQEVVPEVGTLPILPADNDLLLLCSDGLNGMISDFQIAEIITDKTLNILQRTQKLIEKANEAGGTDNITVQLVRFYAQQRSQATLPAGIYKSVVLTQLPSGKKISSKHYILGGVIAVVALLVGVWAFQHSGTAEKKSTDTLQAKPTPDYSQRKYPVENKQENEEYTQPKDSTEKKKDTKLKPKPTEKDEKKQEKTDKKTTENPKKDTKTTDAKSNTQKNNPKNTQQNKSKTEQK

>tr|A0A2N5JZM2|OS=Chloroflexota bacterium

MTPLTSTLDVGALTHVGRRRADNQDSVLHVEAWSGALEPAALGKYGRLYLVADGVGGHDDGDVASRMVVDNVMAYFYTADPVLSNPINRLDDAIQRATADVYVEACRRRNNMASTLVAALITNEQLIVANVGDSPAFLIRRGEPAQKLTVDHLRREADGSQSLAQALGDPEVSVAFRTLPWRPGDTVVLGSDGLSDLVKPDEIKRVVGGSSARRATTELIQRANRYGGHDNISALVVRYGKLPLAAQTWFRQVVGAGLGVVLLCALLAWTLPALLADAAFNAAGKSTSGVSFTGMSIAVPRLNPTTGLTEMVQATITPTALPPAPTAPVPAVPSSSPLSPGVAQPAMAAKPPRAAQAPAPTPRVATTPLPGRTAVSVSAQATPTAQRVQAQPTARSTEHVPPPTARPTDAPPPTEQPRPAQVQVATPRPTDIPQAARPTEVPQAPAPQPTDVPQVPVVRPTDSPPPTTV

>tr|A0A2N5TGR1|OS=Puccinia coronata f. sp. avenae

MAVPNSRAPLLSLVSLKRTPNKEAIQSIKSKLLRPASAITPASAAATASQSISPPNPQLHHHRPQGKKIPPSSRSTSSHLSPHNQFSPLNLYSHSHTTQNHRQLAPDESELLHKKNVFVFRNGASGIPKNRFHGGVGAPPDNSSSSSSTPKIDDTTRSSEAKHLNLMSSVQVGEDSYFLRSDSLGVADGVGGWSGKPGANPGLFSSKLMHYCSTEVSRYEDMDDVRFLSYHAIDPVEILQHAFERSIHESKLEGLLGSTTALIAILRDDELRIANLGDCCCSVIRGNDFIFRSEEQQHSFNYPVQIGTNSKSTPVKDAQRYNIKVQKDDIVILGSDGLADNLFDEDILEEVLKFTTISNEDHDGTSSGDGGAVNKTRPFFTPQMISESLCLKARTVVEDQQAVTSPFSQRANEEGIHYVGGKNDDISVLVAIVGDRSDS

>tr|A0A2N5X2B9|OS=Pseudohalioglobus lutimaris

MNEAVVLNAEAATDVGQRDHNEDNWLVDRELGLALVADGVGGHNAGEVASALTCSTISAGVAEGLTLREAVEEANRSVREAIEQGKGRQGMASTVVGLQCDESGYRIAWVGDSRAYLWDGMLHLLTQDHSFVQTLVDQGQITPEQARVHPRKNIIVQAIGLQQEGDLAVGENQGQLPQGAELLLCSDGLSDVLDSGELAAILGRDEDPASRCKAMVQLAVDKGGQDNITALLVDCAGAAASEGEGAPRTYWHFDPQSGEYHGMATAAVEYSGDTGQTVVRRIAPRGAEDTVQNFTPGSEPGVSGDERKDDTGAQRSRRLRIFLGAALIVAVILGLISQGVV

>tr|A0A2N9JDS0|OS=Micropruina glycogenica

MTNDHPDTSSRRTPGPPNPTPAPAPSLDPSSRRTPGSPDRNPTAQPDPSSRRAPGSPDPNPAWPLWATDQRAGELAQQQPQPDAQEFSTLALAAPCPRCLKPVRPDERYCENCGWDVHPEVGALPGPSDSSEATVKLNRPQQSRPACVECGGQVDSDGYCQTCGTKAPSPRDRFEASPADWVGGVCDRGVVHARNEDAMALWAAPGDERNAVLVVCDGVTSSQDSDVASLAAAERARDVLAASQPAGLAVTASHDAALDAALVDAAAQANAAVLATTAADSVNAASCTFAAAVVHGDRVHWASLGDSRIYVLGAESVQLSTDDSVAEELIRAGSSRHDAETGPQAHAITRWLGRDADDIVPRTGTHQVTGDSWLVVCSDGLWNYASEPSALAAQVATAAEPGGSPTQIAARLVKWANAQGGKDNVTVALARLTATLNEAAGADEAPATAVQPAPPIVEEKI

>tr|A0A2R6WNT0|OS=Marchantia polymorpha

MAHLIDFRCRCFGGSGYPEEKKSNMALAPQMQGNVSPIGGARVNLGIATPSPARFPSAQFPTRPMSPGPAILSRPQSPGPSIKAILSPPGQTVVHQQRKELALAPGVKVIPHPEKVAKGGEDAYFTSNYKGGVLGVADGVSGWAAENVDPALFSKEFMAHAAAAVGTEDVNDDPRLLLAKAHRATCSIGAATAIVAILDERGTLHVANLGDCGLRLIRNGKVVYATAPQQHYFDCPYQFSSENAQTADDAAIYDLDLLEGDTIVMGSDGLFDNVYDSDIESTVKVFSGSDEDSANRLAMALATLASKHARDRNYNSPYAVEAVSQGHDVPWYSKIFGKKMTGGKLDDITVVVGHVVSMPVADKAEVKAEEVPSAQDEQGEKVEKVAETETFTGFQQPEAKGEQVGDLLTNGINAKTEEPLP

>tr|A0A838RDG5|OS=Ardenticatenales bacterium

MKWIKSWVATVPSPTHPDRNEDNLWVAASGRAAAVIDGMGGYRRRTAHGDVGGEHASSLASKILAERLDAWDGLLSMKEAKVVLRQVIEEINARLWQDLNWSGKVPPEENPDGKSVDETSVGAAMTLVAMCDAGTRAIAAQHGDTHGYALKDDMGLIQITEDQDLLTWERMNGVISEDDAVRIAQAIDHFDGVNLTEMMDQKVMRYFFDKNIFGALGVDGTCPETGWSAIKLVVGDRIALLSDGAYSNMSLDELSNMLSYPDDPAQVVIELSMQRCVLPRFPDANELSKPYNMRATQDDMTAVVLEVGVEDTMEGTTESQDDLALSAEQTTEAMPRPRFITEPLAAPNTLVESDEPTEVPYLDLPEDTEETAPPPPAEDIGEGMENMSGEL

>tr|A0A838TVU5|OS=Candidatus Levybacteria bacterium

MIENPLPEEGGRPPTRKEHREREAQTTGLISAGESNRGGRATRSVNQDAFFSGTRGFHLGEVGGSIDRPATPEQNVAGMITALNGLDVYTIHGENKETIAILPHDIRQTEQDVAALVKSGKMRGIDVVADGVGGGAHGELASSIVTYIFVREITSAVKEGAQVSDDLLREAVISANDVLRSYNRKHKTASQTSLLAQVTDKSGETRIASVLDSRAYKETADGQVTRLTVDQNRREEAVAAGSHDLLNVEDAVQNKPTRVLGFTEDTPENPLLSRETVQINRTILAPGEKLYLVSDGVSEGYDPDDPSTAGILQRVNGTYLQDLAGGMSQIEARHKAEGTLFKEIHLQNAPDNIQDLARYLTRNEVGHHSKDNVTAVV

>tr|A0A838UXB2|OS=Ktedonobacterales bacterium

MTSADQPVALAPLPDGTIVGSYVVLGHLGTQDDHNIYITRVASDDEAEFAQPKPTEPHLLVIEAAAGTIENMRMLEDLRLRHPRLLALRDFFTQEDRDYLAIDLPGNTWPMPARLPLTTEEALAVGVIIGEVIAFLHAHGIAHGHLTPQTVTIAANGVFLAGVEGATIATATDPAPLFREDARQLGLLVGTLSEGYEGNPKLMAAVHEIAANAAAGRYERIEEIISDCLRVLPDGLPQLSEENALAPFTVLVGRATSVGMIRQQNQDAVGILSMEILDDQPEASPGGIFLVADGMGGEASGEVASRIAARVIVAEVARRFLSPAARAAASDAPRDEENAGEAATRMSLDTISSLTEAFRAANSRIRNLARRIEKPTGTTATALMFFAHEAVVGHVGDSRAYLLRGEELVQLTHDHSLIQRLIDIGQYNPDGPNEVAVPRNYLYRSLGQLDDLEVDTRVVKTGIGDSYMICSDGLWDLVPNEGIREVLTNAPTPQEAAEELVRRANAAGGYDNSTALV

>tr|A0A838UZK8|OS=Ktedonobacterales bacterium

MMMPLPETPQPRAALQFALDVTGGRDIVYRVAVYLQELLRKLQDAPGFSRRARIDILLFDGQTAQSVPLERLLESASAAAQGQNGGARFLPLYQQIRALPYEGGAFHTFFLLTRNPAEGWANEVTPLLRLVASVTSLCCGPDCAPDVAIALSKDPGGTRIVNPLDQISRQFDSIALWLTQHFAERFPPPPAPPPAEEAPLPITRPLQPAQWRVQEPQDHTDPVPHTAVARSSGAGGWQALAASRRGKLHAHEGSYREDAFALSTHEDWLLIAVADGAGSCRLSRVGARVASEAAVAGMAGGLRLSWPPDA

>tr|A0A838UZT3|OS=Ktedonobacterales bacterium

MAKQLRIEAAELTDVGRKRSENQDNLAQRVPDDPQELEQDGALFVVADGMGGHAAGEVASTVAVQTITSTYFEAAHGDVLQGLAQAIKQANEAILTIARENVGRAGMGTTLVAAVLCQGILYVANIGDSRAYIVRNGKLRQLTEDHSWVAEQVRAGVLTEEQARNHVHRNVITRSLGTQINVTADVFVEPAREGDTLLLCSDGLHGYVNDVTISDVVSNHSPEEAAKLLVNLANEAGGPDNITVSIFHISEMPEASPEVLAKLQLLKEQPRPTRPVPIVAKATERASVAAPAPPVPLTIHTNGADDDVAVAAPARPRRRVGAWIVRVAAVILIVAISLAAWDFTLGPFAQSRVVATRVTNDLNKSKSDLAALSTHVLPDQLAILSTDQQLLTSDLGLDLTAAQRSSLQSLLDSQIVPAVRSAIVAYDAQAQIVPLSAALPNAAAIPPGCASNIYGSLVVVPAPKGNGDPNGNYFFAGTVDGKVQLISQQLGQISCDVPFAANVDLLTNAPGGAALLINDAANPANSSIATISSDNKNPVPILKLPALAAGTTIPFFAYSPKTIVVVQRNIATQVDTLVIYSGGPKFVASGTPIALSQSIRSLGFGDNNVLYLLLNNGAMATFVPGANTEIHLVGDLQVLPALVTG

>tr|A0A839IT76|OS=Oceanospirillum sediminis

MEFIPATNYRETPEISVFQDVGFRDEQQDRYIACRLDSGLLLAVADGHGGCQASSLVASHLALIFSEETERVSSDARLSDACVRQVIRKVFRRLDQLTQDMACGSTLTLAFIESGSRRSDFSPAIRITTGQLGDSVFAVSSAPGRLSSAPIHSVRYRKKDVDIIRQQFRDQYNEDCRVSCGYIYSSPARYAALALTRALGDTSFTLIRKPEVKSYIVNTSETILLLATDGILKPEVRPGTTIRQIMRRIRQGQYATEIIQELKPLEDNTTLLIARNFG

>tr|A0A839Q1F3|OS=Mycolicibacterium iranicum

MSEAQQSHLGSAIAVRSAAEWLHKSLARETAATDWQLCFASVAWALNEHAQRLLNLSDPDPVATEQHFATTLVCAVVEEIALDALRVHVAAVGDSTAWLLSDGIFHDLLADGNSPLSPLTSSEVTALPRVPSNIFATVVDVYPGDVLLVGTDGFGDPLGGGEGGVGNLFRDVLNGRTVPSLVEFAHALDFSREAFDDDRTLVAAWPADKRGAIGGNR

>tr|A0A839QQH7|OS=Helcobacillus massiliensis

MRDDSQYGTLLTEPIRVRSAAATHVGRVRSMNEDSYLAAHPIYLVADGMGGHNAGEIASAIVVDQFSDLAGQHDITPETLAENLIVAYERITRLDDSTAARSAGTTVALVGTSFDHDQATWVVMNLGDSRIYRLSNDEFEQISIDHSVVQELVDRGEITEEEARVHPYRNMVTRALGPGPNSSPDFWRLPAMLGDRFVICSDGINGELDDAVIEYCLREADDIRDVAPSLVTKAVEAGGRDNATVVALEAVADDAAEEINRYSLFDAGSV

>tr|A0A839YHC7|OS=Sphingomonas sp. BK580

MHDPVAHTGPNRRTGGDRDVRRWNSLRLRSSRPRQASMLSRLGESFGSGAPTRLSGRFVARSLAALRCCEPTNRWSWGSSADQAGPSKRRSEGSREPFCIALLAEGCDVATLIVCEGDEEAPLGGLGATIAARTLADCAGGWTAEIGRFPERGTVEGWLLLARDRIRSVAAERGADPSDFATTAIVLSSDGAKTDVCHVGRGAVLARDASAEEWLVVSRPTSDTPASSGAFLGAGGPSAMRWQRHDALVDALVVVTKDLEPSAMDPRSEASRASFLEPIIAPGFAKPSFGYSGFLSSALTSHLARETVNDRRIAGGSILLARRERPSSGVRSRWMAKVASRRPSAAHDFVARRVSSSLSRVDAGDEPSVTAALK

>tr|A0A840FWY1|OS=Variovorax guangxiensis

MPPSTERNDFNAALPEIITWLMNQVLKLPTDARSSLRVESFPLVGFTDVRHAAHGNQDRIALAYSVESNVTSNWLLAVVCDGVGGSSHGERAAAMAVASMALDMAGLRRMGAINMLREALQNAHVRTSSAFHSKSSTTAVALLVTGESAAIGWIGDSRAYQISEGKVRLLTTDDTLASAVARADSTLEFELNEEYADRLSQAIGAEGSVTPNVIAWQPSSSGAHCMLCTDGIWKPTEGALDAMANVCRDGQELMRRLLLVSDWMGGLDNASAILVPPLDVVREFICDPANMTPKGGVVVCLPGPQQTLLPALSLLDSAGLTRIRNQDRRLDESNESTYNSKKAPTKRRGGGSSKIRSTSGAGTAGQLVIAEEPLDDTSPHPPGKIPGDGTST

>tr|A0A840REQ7|OS=Silvimonas terrae

MKFSIYQDSRTGGRKYNQDRVGYSYSRDALLLVVADGMGGHLHGEVAAQIAVQLLTDQFQKKAQPSLDSPSQFLAEAFQRAHEAIFHYAANHQLVEVPRTTCVACVIQDGIAYWAHVGDSRLYLVRKGRIVGRTRDHSKVQRMVDEGKITAEEALIHPEKNKIYSCLGGSYPPEIEVGGKVALSDGDSILLCTDGFWASVDHDELTQFLSAFPVLFSIPQLMDRADLRGGKFGDNLTALGINWHEEEDDNVSSSAFVSTQKLDQVTIATHVDPIDIKKTGEITEDDIEKAISEIQSAIARYSK

>tr|A0A841DB24|OS=Planomonospora venezuelensis

MIFLSAAVGRFLAGRPGTVIGVCTALVLASVFTFLLHRQVASVGPAGRAGATGRRPPEEGGPSDAPAQPAPPAAAVPARPAGEPLPERHFPRPVGRPGQANLQPWRLPGQPGPAGICADSALVGDLQVRAASIVGPGHRCEEPATPRQDAYRIGVDGTGRYLIVAVADGMSDSSHSDLGANVAVDTVVRRLRAVLTGRTVNEQDMRECFGSAAERMTATARQRGLAPEDVRAAVLAAVVELNPDVHGGRAVFFAAIADVSAWLRRGRSWCRIAGDGKHGLDAGRLSDFLPHFPSAVRLMTRRLDPGDVLALTTDGIGDALLASGELAAWFADEWKSPPFIGRFIDTVGFEARGQLDDRTAIVIWCPERQP

>tr|A0A841GNZ6|OS=Longimicrobium terrae

MTELMGDVREASPLCELTLAISAWSHLGHVRQRNEDMAVVGDALVRDGAHERAVKLATLEHALVVAVADGLGGHLGGAEASRRVAERVREAAPGWPAAWGYERLSGALQAVLSAAHLELLAEGERDATLAGMGTTWTGLVFTREAALLAHVGDSRCYRVRDGVLSLQTRDHTALASVGPGATRTVLTNSVGAGDTVSVDLANLTRRMYSGDQYLVCSDGAADAVVPDAEVLRALESAPGVEGVVHHALERGGSDNITAVRIVVT

>tr|A0A842L2W8|OS=Thermococcus sp

MKKAVLLAFLFLLFSNFVGAGVIEVITSPENASVYVNDKYYGQSPLNIPINPTGRHYELIIEVKKEGYLPENRTLPISPDIFDALIPVEFNLTPLNGTLYVCSNPSHAPVKIIGVNGYEFVGKTPLNVSLKPGNYTIEIILKDYLPHSTRVEILPNETESLSIDLIPLNGTLVIMSEPTNASVYINGSFKGNTPLNLSVEPGKYIINVTKDNSWNATSIIINPNKTYSIHLIIPKKGLPLGLILVILLVIVGGGVGVYFTKFRKDRGERVNHKEQERALKNILSNTVKPYMTMKTPDERTFGMSHVGGRENNEDNLLVLKLQDACLLAVADGLGGHNAGEVASKIAVDTLKEVFEGEYSPGMSDKEVKELLRKAYEEAHKRIKEDAVGEREGMGTTLVTAFMRDGKAIIANTGDSRAYLIRNNKIVERTKDHSLVQELLDKGEITEEEARKHPMRNVITKALGIDFGVDLYEWLLKESDVLLLSSDGLHDYVEEKRIVEIASKGKSAEDIVRGLINEALPVTKDNITVVCLEL

>tr|A0A842NMC5|OS=Candidatus Woesearchaeota archaeon

MEEEQPLLDSEKQSQEDQNLEKGLEKLMKEFEVDKNSLISSTGITDDTIEKRNGTYTIDKKGSLPGGNCFVKENGFVTNPIKSIYAVTDGMGGANHKSGDVASELVCNGLNYKLGLLKDDLSDSKEIDILQNMEDTIYALNGTVSLASGYSRFKKIDEELKTIIDRQKIDENGKKLINLARKKIDDSGILSMDILDKMGCTLDVCFLHKNMVYGGHVGNGRVYKIGANGTIDKMTNEHVAWNPMIDRSKLSRLEEAVEEKIYNAGLTSYMGIGKDIQIDMYEFPLEKGDSFLICSDSLSHTVSEEEIVHAFGDISKTKQRLWNLVVEPEWFAEEYAIRKGIGKEEAVNYLRGNDPSFIILKRR

>tr|A0A842QD34|OS=Candidatus Lokiarchaeota archaeon

MLSRWVAAGGVQQGKHHTAKGTPCQDSIAILQKNGVMAAALADGAGSASNSHIGSSIAVETVTWELVTDFEFLHDSPEQAVADYLSEMVSRALITYAEQQSISASSLSSTLLFVGIKEYDMIIGHLGDGVILSVQKQKPGVLSTPERGEFANQTYLTMSKEWKKHLRISVKTDTSLDALVLMSDGTADSFYLRKEGEVAPAATKLIDWYRDISSSEMDQIVRHNLREKIRKKTPDDTSLLIVQQKKHTSLELKSKPMDYVKEFLGVRRRDALTNSLKILNYMGSEMSVEDVAQKTELGVSTVYRHLSRLNDLGLFTAE

>tr|A0A842QDS9|OS=Candidatus Lokiarchaeota archaeon

MTYWDVSKSLHSDAHYDDHLAVRFFDDEHSTGVAVIADGVSNSAGGRIAALVACLVIPEFIQNNRNDVIYSDPFALIEAALEEAGAELLDVGKRVFDAGLDGIRNILKEIDPPEIREKFVEKIRPKVEEKRKKGNIPDFESTAIIAYFVGSKIYVALVGDGSMLEMRDGTMYPYPQSRGSLNTFLSSKDGYQGPARKLHIRLPKGGTFVLGSDGCQIRYSGEHGAPYALFQNTFRRSLEEGGFDSFAERYYEQLKKQEKKRNVKILDDDFSLIALHLVETPPSSDSIGEGGLEMENVIDKSVEEIESIYRNMGKAIAERVYKEISEQAETAFNEKQEEIESQLPRRIQESIERETKKAAQIAADQMTEEIQKRIKVLSYQLPRSLLKEEITESKPFLESIEKEIDPILTEFVERIEEHSRKSVREAFQEEIPKIGADLKDPVENIMVRMIHSKVDEFTAEIPKILEEELSKEEISKQFDKQLQPLANDFRSNLEKAAQDAIEKASDQNKTKLDNQVQRLRRDMKKQAKLAFDDFAEKSEKSITEIFNKNKANLKEYTESVTENLEKQAEQAISEAYDKQKTRISQEVVSIRTGIEQEATEFFKDLIEPLQDEIRETAKKQLREAGGQVKEDILLDVEKTTSKIIAEKLKETSDALQE

>tr|A0A842WC34|OS=Candidatus Woesearchaeota archaeon

MQYFSFSKKGNRPINADYLITDAKKGIFIIADAVGSNDSAKKTSQTACTIAHEECLKKEIKEPMIKIISALDEAHNSVVRNLPGSATTLDLLLIENKQAYIGHLGDSRVYLFRNHELRQLTEDHSIRHKIYQAIGLPDSVPDQINPPKLKRGDLIAMTTDGVHNSVSREDLTYLFNTGIALPDLAEVLIWEIERKGEYDNYTGILIRI

>tr|A0A843E7C2|OS=Candidatus Methanomethylophilaceae archaeon

MEYDIRAYGGSKRGRSHEVTGKPCQDSSHFGLHPLFPNVALLIVADGVGSAEHSEEGSRIAVDVVREYLESCSMPVTPDMMREAYQKAWDAIDKHTEEGTGWPCDYDTTLSAAVFDAESGKVVYGHSGDGAILAIGLDGIVRSLTTPQKGAEANSVKPLRDLFSWDFGEYEGPFVSVMAMTDGVLDLLMPGRLKLTDEPIYIRLVSWLADFKFFEKKGTPIEECFAKRLSYIQSDIHKGITNDDLTFVAAMNIHVSSEYRGDEYYKEPDWAALNEMWKRKAYPSLYDGKSSESESVEDDKGSIPEEEQVQETKVIEPPKGQWDYEKALLMASRGNIAFTRSNMVRAAEAGHPDAIAYLDPKTLGPLEDPWEMVRCRVMIAAGIETPYRNISEYYKSKGEYAKAKEYEERDQKSSFPLSHTVSSFRSKLGRFR

>tr|A0A843T1W4|OS=Gemmatimonas sp

MSRANLRGIRLEVSGFSEQGPRAENQDAYTVSGFEDVGLLAVADGMGGEKSGRVAADAALHVLLDASPLRSVDDARRAIREANRAIAALSDADPDAHGGMGCALGVLGLTDGGDGPGWIAAHVGDVRILSRSPDGLLRLETRDHTPAFARWEAGEISLDEIPDSAGANRLRRAVGRGGEADVTWLPAAPGWSWLIISDGIYKAMRFDELAAAMDLPTTTETCEAIRLKVQERGSDDNYTAVYVRAIGGPAAAFDHSNDTDPMAPTPATPHGPRSGRGGLALALGILALLLAGLALWTAYSGVLGASQQVEIESLRSEVDSLRIIVDELRDPFGPAAPTDTSGLSTQPPPAR

>tr|A0A843T484|OS=Gemmatimonas sp

MSQAEVRSVKLTLSGRRRNNEDSVVDLRFPDGRHLVAVADGMGGHQSGEVASSMAVDVLSREVAGGRDLREAVRAANLAIYNAARRDPRRSGMGTTLVALLRQGSTYEIANVGDSRAYRIARTGIFPITRDHSFAAEARQSRLMSPEEIARSPWRNALTRSLGAEESVEVDLFGPFGWTESPHIALLCSDGVYRSVSDEAIQKLVLSCGDLASAAEVVATRAFQGGSDDNMSVAGVEFGRVVKMPNGPQANSLPPQAARPVAFEQLSAPPPTVRRTALAAADAGGHSETQTAISPALWGSVTEPGVFRWIRRFFGVIAGDNTLFGVSVAIPVLWLLAHPSTLG

>tr|A0A844B081|OS=Tritonibacter aquimaris

MGFVVLADGMGGHSGGDLASRVVVTEVFSALKRFGSDIERMENRITQILERALVAANAAVARCAKEDPARRGMGATLLAPVVFGQRLYWASVGDSPLYLFRNKRLQRLNANHSVAARLEQLVRSGRMLPETARRHPDRACVTSVLAGGKIHQVDIPAKPVALLPGDIVLAASDGIQALEAKQIGDLIGQDIGSSAAGLSQKLLQAVLDAGDPQQDNLSFSIIRIPPAAANHGVEVAPKPRARARRKVALVASAHVQSGQLVFQSLSKSSA

>tr|A0A844IB27|OS=Aphanizomenon sp. UHCC 0183

MGWKALVRFDSGVSHHKQGIPCQDSGGYICFKRILIGAVSDGAGSAKFSDEGSQLAVEVSLKFLKKQIEELLEDNSYNIRRFYSEEEVKKLFSITLEKVIKELEKKSQERNCDLNDFACTLLLFVSFPDFSAAIQIGDGFIVVKLHGKEEYELLFKPDKGEFANQTSFITSTNPFQDMKFVYKSTYYPEFVCMSTDGLERLALNSSDWKPYSRFFQPLEYYLQNEDPNLDDPEEYVLKFLKSEKLNAQTDDDKTLLLCLFNHDFPEVDRSYIPKVTISPVSDPGEDIKISSPPSNYENNHPVGSKTPIPIITRRKRKNNHIPILNKLVFISFLLLLMITIFSLFSKILSPDSADKNSYILSTPLTIDRRTQNKNIDSDFKTNKSSLVKIFKENIIDNEFYKDKIQSDARIQDDGIKVFKNTSDHFIVIIPVKGKDFQKEYFIELKNIGVKKGEKVWRIVWLASSD

>tr|A0A844MLB8|OS=Scytonema sp. UIC 10036

MENRIDTPEWRCIGASVLGASHERTNLPNQDAIQWYPNLGKGPPLILAVSDGHGSPRNFRSDIGAKVAVDTATRVFRELFVEGDLGKERDKAIAIKDIAQNFWLPQKLVRDWKEAIKKHWQENPASDTDQAWQRVVEKDGQPAVAAIQKHPEIAYGATLLVVLVTDSFILYLQLGDGDILCVDSKSKVTRPIQRDPRLIANETTSLCMPDAWKEFQIVLQQYPQGEADKMPALILVSTDGYANSYSTEEEFLKIGQDYLRMIRGEGTERVARKLEEFLKETSSGGSGDDITLGIISRVEQGYKDEELKRMNSLEKNVSDFDTKISHLGSKHNSLSKVVSRHGQLLIVAFFLAIANIAFSSLLFFRINHLEKQQKAFEQHLTDVQQSLQNTQEQLQKLTQNQNKQPVAKPQTTSQQNRQR

>tr|A0A845F243|OS=Pseudalkalibacillus hwajinpoensis

MDRNRVGVIIIQDDQIALIERVRNEESYYVIPGGGIEGSETSEQAAKREASEELGVEVRIGALYTSFQFNGEHFYFMAEIISGEFGNGTGSEYRSSSQKRGTYKAVWVPVAELNGLRIYPEEVISVLVTDARRKIAESDPETEKSHQLSWVGSMYPYLDEPTCTEVGNVSVGRFGGKTSAGQNKNEDGCLVWTGVDWEFAVLLDAHKTASSAALILDEVERYHSMLTNILKKKTQEAVSELREAVVGLFQNATFKQKCREVEGETACLIVVRKDKYLWWFSVGDCLLLLIHPELEALGESVQNHRSFYEWIGEVNTFDTIVPCYSTGTKELRKGDNLIFLTTDGLLECPHTNLHFLDQIVARMKGKSVKEDVQQLLKEVEGKGVRDSTTIVAWKINIVEDGVMPSDL

>tr|A0A845FV74|OS=Pseudomaricurvus sp. HS19

MPSTPYSLTFELNDIGGRKSNQDRVAHFANAAGDQWLLVVADGVGGSDRGELAAQAIVDVAAEVWAEGAPTANTEAWLQAFAQRCNDRVGEVQSSSGHRSQSTLAALYLSDGQAWSVHAGDSRIYQLRSDTAEHSRDHSWVYAQFLLGALKQEELATHPARNQLLNCIDGRAESMFDVHHWDADSDAGYLVCSDGFWDIFTDAELPALVRERNCEAQILNRVDDYIAAHPGHDNTSALLLMPAVAEPVATEPVTTEPAAISATDTGTRNQVVVVRKTPPKHRALLAGVLIMTALLLGYSAWRSLSPDNSGNAATAPSTASTAAGEENGGDDNQGNSEQAGEGSPATQPGNDSNSEDSPATAPGFEGEEGLNQGLLYVEGIRIPVPAGEPDVVDAIKDYLVSHGYMRQQDKLWLGKGSPSELTGEWIATVKFLYKLAPVLGGELKVRLLVDGTTVTEVEVLSGQIPQLQQLPEAPAHSFADCLQQHNAVPAGQRQTPEPTLLVSAAHQDFLWVDELVINNEPARYYLLAADCSVELTEPLTISGGETP

>tr|A0A845XC00|OS=Spirulina sp. SIO3F2

MARSPLSVHPYLWAIGPIAAKLTPPNVIVNRYQIRAPQIWQDLYPYDLPDFPESLPPEVLPYLRLYPQRYHVPEVYGICTVDDSDVVLLTNAPLTAQGELLPSLESQWLDASPLRQAYWLWQILELWSHLQREGVATSVLVADNLRVEGGRLRLIELLSDRQPLTNLSPLVKLWLRLARHAHYSIAATLENIVAMLQQPEPSPTMIRRSLNHLLLEQAAQLPLSVQVASATDPGSEQLRNEDAIYPPAGNTAVDPRVLVVCDGIGGHAEGAIASQSAVQSLQLQGQALISELQADPLRLEPEMVADQLTALIRISNNLIANRNDQQNRSARQRMATTTVMALQLPQIIETPENRGTSHELYLLHIGDSRAYWITENYCHCLTVDHDIIRREVCKGEAMPLQARQRPDAGALTQALGTRGADRLHPTIQRFVIEENGILLLCSDGLSDRYLLERCWTDFAPEVLRGTMTLSVAVEYLVRLARQHNGHDNISVVAALYNVQNVVLPTASVVDSPPAELMPTLAELELQQLANEPSEDGQLEVSTIADSNGTHQTPSFLESEPTVPPTFKNLEDVENLLLELTQAEPSSEFKPSESQPEISPPSSSVPSAKSETDAPSAAQMPEPIAPPESSTPAPTPAPTEASDMAAPLPPLPKPNAPNNSDDSGQASDAYTPNNALPDMVPTWDNPNPWAAASKPPYGPGSEPDGSDEDAPELPSSLFENMDMDDLELTSAPGSRWNWLWVVGILGVVAIVSAIVLVMRSQFATEREPEPERTEQQERSQDEE

>tr|A0A845Z5C4|OS=Okeania sp. SIO3B3

MPWKAIACSEIGTSHQKSGLPCQDYTDFIRLNNAGKISDNGEIVIGAVSDGAGGYKHSRIGSELAVKTALNSLKLWPKSLKKEQELSAERLKELANKAFGKTFREVTKAFEKKAKKISCSPKDFSCTLLVFVTTPNWLAAMQIGDGFIVIRQPESEYQLLFHPSKGEYANETTFVTASNALGEMQVKTLFGKQQFICAASDGLERIAINMKYLQPYPPFFEMFEKALEVRHEHEEKRSTEEWLKSKDVNNRTDDDKTILVCWYKDFEPGENPSYPRIGEKYNTDLTVFAVNVLSGIFWNSLYHDFFVQIEFNNSIFRWIISINIAVVLTAIIILVNLYIYKPLKSSKSQYQKVKALIISSAGLGLGGLFYYSIYLYILYLSKL

>tr|A0A846CIU1|OS=Moorena sp. SIO2C4

MNKSKSKSKSIPFNDEEFSSLLHKHSSNLIAAQAITSSNTARTRKSFGHYYYISLDDLIEFQEQLEHKDALNELIDDIEIHRDFIKEQKHDEIKSPLNKYFEINIKEIKSEGYQGYMAVEDLANEDNNEESDDKNTSDDELELYARETENVENSNEDENLEETVASESSSMALSIQEETEELSEEDQSDELDSKAAAKEISKVSDSSETSTDAELVNWKKVYGKAVGRSHLKDDPPTPCQDAAIAVLDPRPAILVADGAGSAPNSHLGSQEVTKHLRAAIKSKNIEAIQRKLLDQEEFTESDDTVNYAHGFIKEAISSLKKLSQEEGYPIDSLKCTLLVAVLGKQRLFWLKVGDGFIVIEKNQSLQLVGPIGKGEFENRTNFVTENIRSKNIHYGFVTSRNITGVAAFTDGTAEKLVSTDGKKIAGAISDFFNEIREDKLTNDKLTQFLEDPQVWATPWGNDDRSLALLSK

>tr|A0A846D753|OS=Moorena sp. SIO2B7

MAHSAAKIQCSNPHCQTHNSKDLELCKKCGTPIVKRYLWVIGEGIAAYQVGKLIDERYFLKHQRLVLDTKPARMPQIPDEIPQSIRPYLQLFPYRLHIPQVFGQLTPPPNQPESEIWLLEYATVSHNSSGEFEQGQLLPPLTKVWQEANPLRQLNWLWQIAGLWQPLKTKGVASSLLNPSLLRVNGSIIQLLELQTDDDETPSLKHLGQLWSEWVVNSSPSITEFLQKLCLRLEQEQITNPDQLIAILDQGLKHCGHSDLRTYQIFSSTDTGMSREHNEDACYPPSGQLVTPQPEEKALAIVCDGIGGQDCGEIASQIAIDSLQKGVENLSLDLEQWNPIQTSLELERVIDVANDLISKRNDSEQRYERQRMGTTVVMTLNHAHEMYFGYVGDSRIYWVTPTGCHQVTVDDDLASREVRLGYALYRDAIQYPTAGALIQALGMSASVNLRPTVQRLILDEDCVFLLCSDGLSDFDRVEQYWESEILPILQGKTDVAKVGKKLIKIANQKNGHDNVTVALVHCKVQRRELTDKVVLSWDEIESSVPVLPNQAIAEEFEEKSSQITMQYLEEEKPRSPLTLLLALLIFLVLGVGGTLLYFFFPEEISHQWHELKARFLSYPSAPTSVNSSPDLVLDKGDVIKIQEDITIRVKIKDSLNIEEDHKFPEGSIIKILQKESVSSEETLKVLICQYSTPEVITEELEGEITVKDIESKKIEKITAPNNGNCFLAQPNDSKP

>tr|A0A846TCE6|OS=Synechococcaceae cyanobacterium SM1_2_3

MSDWRTIKASVRGAAHVRAGLPNQDAVRVTRCNDDLWLLAALADGHGSAKSFRSQRGARLAVVVAQQVCGHLFKLDGPTQIKRWAEERLPQELVRRWRERVDKSLKIKPFTPEELSPLDAASRRQIETNPYLAYGSTLLTVIVAPAFILYLQLGDGDILTVSAQGDIKRPIPVDTRLIGNETTSLCSAKAWNDLRVRFQTLAGSPPALILAATDGYANAYRDEAGFQQVARDLWALLRDEGDAAVKPHLKNWLNEASQQGSGDDISVGIIWRPVHSPATISIFMPTLIN

>tr|A0A846U495|OS=Spiroplasma platyhelix PALS-1

MNVVASYKTDIGTFRRENQDSFVFLKNKTGQALAVVCDGLGGHNCGDIASSMAVAMLKNFFNQTEWKKIKTDDQIYDWLRTSVETIQQEMNEYSVSHEEANDMGTTMVITLIANHKAYLVNIGDSRAYMFNDKGLKQLTSDHNILNLYLKNNPEKLAEMDVNKTYWKALTSALGPIKNLTIDIFDLSITTPSYFLLTSDGVHDFLEEWEISHILEKNSSLKSKVKMLIKQALSNVSTDNLTAMLLLVKD

>tr|A0A847AZJ2|OS=Elusimicrobiota bacterium

MKISYVTQTDVGIKRDLNEDYYGAIPEKSIFFICDGMGGHAAGDFASQTSAETIEQLFKDSSQEDWEKITIAGPAEISLQSRRIASAIITANRRLFKLQVMYPKIRGMGTTFCGISFQNGLCNIFNVGDSRTYRFRGNNLEQLTVDHSWVEEMLQDGDISAKDLDTFGGRNVITRALGTNPLVQVDWKTVSPMIGDIFLICTDGLYEEIDDSQIQHILETFGSNLEVAAEKLIQAAKEAGGSDNITVTLARVDETTAATPAGAIQPTIKALDADESTIQMIDRHIDKYMPPAKTKVPAGVVREKRKLYQIPLVQVIGILAIMTAGALLISRPWNKVVPEEAAATTTGDILLSTMPGAADVSLYLDDQLITTTRAPASFLSLEEGTYSIRVLLDGYQPESFQINVIKGEQIVREVQLRAQAELRLTLGMSPGFNNEEKIYLNGETWDYFGSPLTVRRVGVVGKSIHITRDKDYALRVGNIERTFRLNQNEESITLTLDQGKMLIER

>tr|A0A847GNE1|OS=Candidatus Anammoximicrobium sp

MLFEHRAFWLPKDVQNPNAYEDAFDVDAVRGMAAVCDGVSSTIFAGRWAAILAKAVVAEPPDVSSHELLDVWLKRTREIWSHSVDETALAWHQKPKMLDGAGTTLLWIELATEESSDGIARPYQLRAYSIGDCCLFHVRHGQVLQTFPIQDSARFETNPQVIRSVFKRGDVVELEAMETQCRPGDLLALCTDAVAGWTMRQLEAGATLDWNAYWDMSLEQWQQWVIGLRQQNQIRYDDSTAVLLRLGGEAPAIRVVDREREESLLDTAETKWKDAIGSLKGSLRKGLRELSESKWLKDRGKK

>tr|A0A847JZJ7|OS=Chthonomonadales bacterium

MTDCSGDSGSNGMDEDLEITARFSRQELVRGWRERTCAPPRVVPVTRLGLKTDLGAVRENNEDKAEFYEPNDAGVLASRGALYVVADGMGGHAAGQIAAELAIKKVLSEYYDGTADSPGQALLDAFEVANEHVRSVARAIPGRSGMGTTLTALALVEDQAVLCHVGDSRAYLIRGDQIRQVSEDHSWVAEQVRAGALTEAEAESSPYRNVITQCIGPLAEIAPDISAVETQPGDRWLLCTDGLTGHVADHELLAVASGQSPSEACRRLVELANSRGGRDNITVMIVDITGLAPWPESS

>tr|A0A847MUE3|OS=Intrasporangiaceae bacterium

MPPSNHTDAGPHGTGVALGISWATASHLGRRSENQDRVFADGRLVVVLDGVGGVEFGGQAASTALAEAIRVTALQQAQGAYDLDVILRSMNDAVLALSQATGWQTATTAVLAIPEFTDQGPLLHVGWTGDSGAILVRGSQLSDITGRGSGGGRRLEEWLGNPDGYGRNQVTLPLQRGDRFLAFTDGLTSACSLDEIGAQIASAGSADAAVRAVLGAAHKRGISDNTTLGCVFFSEQHSADAEILMAPEVDSEGLDEHNQMVLQEHR

>tr|A0A847WDQ6|OS=Epulopiscium sp

MWKTVNYEVCGRGHKKDSIPCQDKTYSSYIKNVNIIALADGAGSARLSHYGAEVVIKAASEYISNNFDAVINNEDGKEVKINILNYLLDALKNKSTELSCDLKDLASTFLMVAVKDDVFLIIHIGDGVIGYLKGHELKIASRPENGEFANTTTFVTSKDALSSMKLFRGKTDNIHGFVLMSDGTSDSLYNKKDATLAPVITKMMHRNAILDHDKIFEKIKQSFDSVIVNNTLDDCSIAIMSRKSEILCDYQDMNDLGKCDLLGIVYGTNSYKQQISKYDYIVNYLHKPRTLEQVSRQLYIKKKYARKHLDKLLATGLITRVGHKYAQYIK

>tr|A0A847YDG3|OS=Thermogutta sp

MGGTVMVQSLCWKVVGVSVIGTQHMAAGGRCEDGWSTVRRRYPDGRTILGVCVCDGAGSTSHGWLGAQIASRVVAAWLADNFDGALADPVEDNRWSISSVAKRAIRRVAEKAGLSIKDYACTMVALAVSSDGRWIAAHLGDGAIVGMFDGCLRLISSPRKGEFANETYFITDQDSVENIEFQSSAGFDIGEQGTAFALFTDGVEVTLVNRHTREVAPALRYMLGWLADHDEGEVAQDLEEQVKSVFQQHTSDDCTLALVAKANHQSDVDIISDSTR

>tr|A0A847YU41|OS=Veillonellaceae bacterium

MGRIESEQSREIKITQLDRGPFLLRYASYRKSEDAKLNLRSEDYLASDIERDRAIFVLCDGVGSSFYGDIGSQILGEAVLFWLQNIRFEDVLSSIHSMEGSNWIKNIHKNLQDFLDSQVKFASAMVEQKDITTGKDSWTQLAEKNQRDQFGTQSNFVGGIIWPKSTICPNGIVLLFWLGNARVRIFSREGELTYLLRWGEDPNQLREVWSSREGVIGCVHSYVTDLSDIEYIIGYSDGLEEVENLIYPGISSDQFETIVNRAQSIKDDDISFFEISPSVSQVVGEGDDIASSVREYFRTSSLKSNGASIQEIEILKRQIRSLIKRIDEYKLLLIKANQRTKVAVGIASLIFLCLGFFLSYLFNPNREIIVDRPVYVTSEPVIIYKYWPTPTITATPTSTPTLDVTPTPTVTETQTTTPTISATPTISATPTVSATPTFTPTLTATPSSTLTETPTSTTALNMTQTVVSQVLSSPILVRTGLPTLNIINPALKCQTIRLFQPSTSPPSTL

>tr|A0A847YVX8|OS=Veillonellaceae bacterium

MIYAYGITTQGTRHIKTNTPCQDVHKIGKISNDIVIAAVADGLGSEKYSDIASKIAAETSVNYCHSNISKKDSGEAILNVIRSSFEMALNSIKDEATKEENDFYQYGTTLTLAVMVSNDLYYGHAGDSGIIVLKLNGLYNKITEQQRDDYGFVFPFTYNKEKWEFGTVENVASVLLATDGMLETFFPIYLRDEKVNIYVALARYLMDNNSLHIDEVGEGATQDRILTFICNIPDAQVNDDKTIAVLVNTAVKAGVQPPEYYLEVDWAELKRKRNEAWKREAYPHLFTDETNNDQATSDNTKIKNPTTTQEILVEETDN

>tr|A0A848ERK9|OS=Megasphaera elsdenii

MTKRIYGGFSVKGIHHSKNDDSFDAGKFSDGYYIGLSDGLGSCPLSHIGSQLLVRILAQVVAGINPQKETAASFAVQINQLWCQKINESGYEVRDCLATALFAVVFESAQTVWLSRLGDGVIVALTDKGSVVLCDSKEMHFCNETNSLGEINAPWEVKEIKGDWVKAIFLWSDGVSTEGDISEIESIANGLFEEYCGKSRLMVEKDLSQWIPELPGNDDKTLVFLIAGEQTL

>tr|A0A848HFJ9|OS=Massilia polaris

MAERAPIALTWASINAPGMRESNQDAIGDARKGGVACFVVADGAGGHAAGEVAARIAVESVIGNFVAEPVFGAAALQAGVDHANAAVARDKQQSAERQDMSTTLAVLLVGEQAARAVWAHLGDTRIYLFRDGRLLCVSKDHSLTQQLIEAGYASTAQLRTHPQRNILYAALGAGGDTPPVISEEIEVRAGDAFLVCTDGLWEWVVEDDMERSLRAANGPEEWLAALCATAHTHASATPKVRDNYSAYAVMLGAAE

>tr|A0A848U0D7|OS=Granulosicoccus sp

MPDSPSSAPPAPPLLSPAGRSGGPVSDPGTTAGATDTSSEKPVADKRTPGTQRWFSCAASHLGHVRKINEDAFMDAREQSLWVVADGMGGHSRGDRASQSIIEALHTFEPLANPLDSVDDLLSRLNQANDTCRQAAQGQVMGSTVAALYLHEDRGYVLWAGDSRIYRHRHGDFAQLTDDHSLVQELHRLGELTADEAENHPSSNVITRAIGVADDIEVQVRQVDLEPGDRFLLCSDGL

>tr|A0A848WJS6|OS=Silicimonas sp

MRETADISYDVSSVLNRGCRDYQEDAIATDFSLGAEFGYAVLSDGMGGHAAGDVASKIVVTEVFSELKFQSSNVQKLAENMGHVLKNAAVSANECMAAHASTNPQTAGMGATLVAPVIVRDSLYWISIGDSPLYLLRNGTLRQLNEDHSLGPHIDYMVRSGMMSEDVGRNHPDRNALTSVLIGETIERIDCPDRPFQLKDNDILIVASDGLQFLSNGQITDTLLENAEEGSAKIAECLLDELRTLNDPEQDNVCFSVIKVEISSAAGASSNIFTPEFDRKENRPVRVTTRPIKPEDVPEQADVTARNGAEPPHGKEAPIFLRRRWLAADGGT

>tr|A0A849AC31|OS=Flexivirga aerilata

MDHGESAHDGAARNHLLLTPQPWVAGLTDIGRRHHRNEDALALTASAEPAARAVLVACDGVSTATDSHIASAAASQAVAERLAQPMPRDEAGEDDWLATVSRAFTEAAQAGSRAAAATAADGDPSPPSCTLAAAIVEQGMIVGGNVGDSRVYWLPDVGPQRAVQLGTDDSMANELIRRGMGRAEAEATPTAHAITRWLGRDAPEDLSPHLAHLRVEEPGWLLVCTDGLWNYCSDPADVWDLMQRAVAQRDGTPGSVVEALVDFANGQGGADNITAIVARVVPWDVAGQSVPAR

>tr|A0A849DJ53|OS=Dactylosporangium sp

MPDADPSRRPLGASRRHGGDLWPQVVSGDRGSDRVGPVPDVRTRRRRSVDDPRTRRQPRVLLGDGRTRGRVDRHRVRGATHLARSGVARRRRCPRARLRRRGRLPQQGTGQPVRRRRHPAVLDGRSQRPRRRSCHPKRAAVPAQNPRVARIRTAGRPGSDDRPSQDRVFTTPNAVIVLDGASQPNPDAHDGGWLADTLGSQLRDRLIAEPLTADLGAVLAQAIDGVARRYALVPGRGPSSTISIVRWNDTDMIDVLVLGDSPVVALARTGRLRQVRDDRLAQVAPAERQALRDTRPGQFGFDRPHQWQALVEAQRRQRNTPGGYWIAEADPEAAAHAVRARWNTRDIVAVLAMTDGVANGVDRYQTPDNWHTALTLARRSPTDLVNLVHDTELTDPDGTRWPRSKRHDDKALALIEFVRRETA

>tr|A0A849W9C8|OS=Candidatus Brocadiia bacterium

MGNNFYKNFTFLRAQKTDLPFTMGGISQTCEQLESKNNQDAMILDVDNKAILAIIADGCSSPSCSDNHVSINEIGANLSCLIARNCIRNILRKYDLKDTNAFLYALAKSFLGGLKNVYNGIFSYKEDFQYIYSNLLSSTLLCFIVTKEHYLLFTVGDGIIAINGRIENLEEEEGIYPVQKIGETFARKDALDTLFKVRSFGETCSLESLMIATDGFQDFLSLPGEPLATFFSNSNRMFTPGYDPAMFLEFRKRIFIPLQKREALRKIHDDRTAIFLRRIESQNLKGEMYA

>tr|A0A849WL05|OS=Candidatus Brocadiia bacterium

MPLTLKGKSKARLEIQWDGNTPVELSIVPSSNTNTLLYREQILKAGGFQLELTHLEFNNLSEIAELELVALVKSNGVLVEVSEPLSIQKCPHPGCGQWSHAREDGYCKHCGRRIRDQASNYGFAKLEISHLNVLLWNGVEGQLWKKREAKEYSNGIYSIGKYEDKTHPRAYVEILEEKYDPKAQKRFIQLLGVLKEANLLDKSWKPPLACFQEEQQRTIWIYYPWLPKQSKWESLSALSYIVSENIEPLTIKEIAQIGIKLCDIAKKIRSLGYSWGGLKLTDLILCRDNPSISIYLRSKEIAWEEVPPKILLDSCLIPWELFWESNGNENALSEATEVYVIAAVLYLLKAKSPNLLSYNAISYHHGLPSLKLFKPVFMEKSQKKDFASDHFESVINQALLLDPQERGYQTLQELKTILENLLLYGNSPMINRNYFLDVGYTLDVGDEKHEDDLTQNQDAVFSTCFTLRKKKWGIFVLCDGISTSTIGTGDLASRVIINTFRRWWKSTTEEEKKGVCEYASTDIAKAQEFFSNIIHEANQKIYKEAEKIAGKDGLENALIMGSTITAGMIHDGVMFFCWLGDSPIYRISPFGWERLNFEDNEKNSRILKGTPLEECFVEGGNALTRCIGAHFYLEQKLDIHFGYAHLYPGEQILICSDGIPDYIEQEASYARYENYQMLRIASVIQQYEKEELFNAKALSSILLSSVNRIGGGYDNLSAILINTIPESSLSFEKSYQKLRSLSPGMKKILKEANLDTKEAKTMKLPRL

>tr|A0A849YR26|OS=Polyangiaceae bacterium

MALSEHIQFFPLTDQGRVRDLNEDNFLVDKKLGLGIVADGMGGHAAGEVASALAVRIIHQEIRRRYDLVKAYGSGSASRDAAREIMHSMEGAVLRACSRIHEEAKSDASKRGMGTTLSSLLITGSHGFVAHVGDSRIYLYRDGKIQQITEDHTVYNELIRRGRLTKEQIEKVAQKNAVTRAVGVYERVEVDTLMLELLPGDQFLLASDGLHGYINHVAELEPYFEDEDGEKASRELIELANKKGGKDNITSVLIRVGHTAQKDTERARRLAVRREALAGMPLFSKLHEREMLRIMQVAEVLEFEAGQDVVREGDRGDELFIVVDGQLQILRGEQVLSEVGPGEHFGEMALIRSLPRSATVRAIDKSELISIRRSDFFGILRKEYELAGKLLWQFLGVLADRLDQTSRDLSSAKEELAAEDISDAIAEDEELGLADTGAVPNGAPSDGQADDGEAEEPPPQSLRDGPTSVQLNKTMPSAAAVAAARAQVRGALGTIPLVEVEGSTEGQAAEQAGRTSGS

>tr|A0A850APG1|OS=Calditrichaceae bacterium

MKNYIFAILIALLLAAAGSLPAQSPSPPDTSGGGAASADTARVDTSSETLPDTSAAAREDTVQTAPAQPQQPDTSAGAAARPDTAAVTPSARAGSDTLRRTAPDTAAPISRPAPVEEPPATSPALPEPGRDLPASPQIPAETAASGGNDTPLEVQDTSQASPGVSSPAEDFLPEKTGEGGSAGWIFWLAGLLLAVGLLIAILRKRFRGYPSPRDRESAGFEIPAHEPRRRAEAPAPAPAAAPAMEIALPGGKLLFSHAQHPGSQWEQQDAFGFSETETAQGGKGFLAVIADGMGGHAHAREASRAAVDGFKNAYRAKSSDEKIPAALHRSLRAANDAVLALAKELKAENNLGATLTAALIFEKNLYWIAAGDSRLYLLRNGQLTQLTTDHIYLNKLREEVAKGRMKKEEAENHPDREALYSFLGLRTLREIDAAKTPFSLNPGDRLLLCSEGVYRSLSASEIVQILSAGENANLADQVVEAVLAKRRFQQENVTALCVGLG

>tr|A0A850ARJ6|OS=Calditrichaceae bacterium

MPGDSIEAKTRYQVDYAGVSQGENRQPENNGFPAKPLLHTPKLSDAKGQLFITAATRSSRSGGQDAAQMAAQIIRENYYSYPSNDIQFCLKRAFDIANRQIYLHAKANGSYRKFKASASALVVTDSRAYFAHIGDCRIYRIQGDAIELLTREQTRSPELPVAEPPPAPEKKTPAIRLLTRSLGLKLGVKVEVSHSLAVGHDDHFVLCSSDFKNITEGELKEVVLSAPAPQACEQLLSLARQRGGRDDAAIQIVTISNPPPAPPVEIIAEIQEPAARFRFRAEHLLVFLLLAIVAMLLYQPVRDNLLGMATKRLQQIAATGNEMLSGEETENLLLQRGEQYLDDEQWDQALEQFQAILRINEESPAALNGIALVREAYEYRGERAFRQKDWKNAALYYSKLVNLNPDEPHLRQRLAESRRNLEVRANLAGTPAAASRNSDAQPQRPPDSDDFSLTIVDGIQRAQWEMPGLYETEDYRLGVGYITFFDNLRIKKAFHQGSYLGLEAEASAKVLSASGGGRYGIIFGHDAEAPSSAKNFYIFLIDQEGQFILQHIAADKVKTLVSDPIKPGILSASRAVHLKVKSVDNNILLYANGELLKMVSLPQPAIGGVGMYVDPRMSVEFSRFKISPAQFR

>tr|A0A850AWJ2|OS=Chthonomonadales bacterium

MIEELDETTARFDIQELVLGWRRLNGEPPRLRLTTRVGLKSDLGCVRDNNEDKAEFYEPIDPPTLAARGSVYCVADGMGGHAAGQIASELAVKSAIAAYYSDMDNTPDVAMVQAVRQANDTVREAAIEAPERAGMGTTFTALSLVQGHALVTHLGDSRAYLLRDGELRQITTDHTWVEEQVQNGVMTRAQAESSPYRNIITRCIGTEPTIDVDSFAVDTRTGDIWLLCSDGLTGHVEDDEIQSIVDGAGPSEACRQLITLACARGGSDNVTALIVKIEDMETVEVAA

>tr|A0A850BS71|OS=Polyangiaceae bacterium

MSEEMSSSPTRLVAAGVTDVGKTREINEDNILVEPELDLFVVADGMGGHSAGDVASSLATKSIATFFKNLHDSPPASDGSSINSQALPPEAQSLVTAIRKANQELYAISSTDARHKGMGSTVVALLFSREHGQVHVAHVGDSRCYRIRDGSIEQLTRDHSLVNEALALKPDLTKAQIARLPKNVITRALGMAPVVQVDIRTEQIQPGDAFLLCSDGLSGLVPDEQMLEIVGMAEDTNEACELLIAFANEAGGTDNISALVVRVEGEERESAAPSSAAVEIEQAASSKEFEPLSAESLAAALAAEAEAEEAMTPAEAMEAAPPPAMGHARVGEADFERPVTSAASLLSTAAMEVPAEEALPDIVAVSDESPEVAPSEEEAREIEAAPEIEAAPEIEAESEEIPPELLAEVEEVAAIDVEAPVSEMPPEEPGPPVFIAPIIDINAPPGTTPAPPPPEPKYKPPMPPARVAASADEGVEVSEGAEILSEFADTLVGDEDLDALLDEREAPPTSFVPVARCKSCHHELYIGNLFCTECGAPVED

>tr|A0A850GE47|OS=Pseudenhygromyxa sp. WMMC2535

MVSALAAVLARWLRWRGRGSRRSARPRDPLAVYDSFGALDRQGQVPAGDDVPYTACFGDARVGLSAATVRGRRHRAAGVRCEDAHRCVVLRDGQVLIAVSDGVGSSRCADIGARLSTMAAIDYLMNACELDVEPPLEGALSAARRRLESYAQGRCILLEDLACTLLLAVFRGGRLYTARIGDGGIVIARGGEWSVATAPARASRYVTPLTTADWRARWDHGRFEEVEGVCVFTDGLERAYLYARDGEHEVVEHVHAAAADSDTPEHGMQALCDELLGPTFEGLSDDDKTLVVLWRSAG

>tr|A0A852SE68|OS=Agromyces atrinae

MIVERVMSVPGGSVRFRSSARSDIGLKRAVNEDSLSADAPVFLVADGMGGHARGDIASRTTVDVFREHIADDAPSTPERVLDAVHSSNDAVRDLSDEGDVGEMIAGTTLAGVAFVDAGAGEGCRWMVFNVGDSRVYSWQDGVLAQVSIDHSAVQELVNAGLIDAERAETHPDRNVITRAIGTDEYVDTDVWLLPADGVRSFLICSDGLTKELDDRAISEILAGCDAARDESGTLDSDSPADLLVAAALDRGGRDNISVVVVESLFTPDGTSAGQSPLA

>tr|A0A852ZHE9|OS=Actinopolymorpha rutila

MDWLEVGEPNGGIAVRAVSARGHMHRYLGEIRQDSFAIGVTPEAVVVAVADGVGSSSGSHLGSALAARGIANDWELIEEIIGGVKTGETSFGKIAVRLTHEAKNSSFTPRDVSTTLIVAIVRRAPEGDGTREVVLAQIGDSDAWRKTDNGWTSLGHGDDPNSGSAVLSTVVDPLPEHPTARVWRESFAPGETLALVSDGIGNILRLQPSYAHDLAELWRRGAPSPASLLKVVDATVKAFDDDRTFVGVRFP

>tr|A0A853CDI1|OS=Pleomorpha daqingensis

MADGMGGHVGGAQASELAVQRLAEIAQGPTVTMEAIRTALEQADKDIMAMGADVDPLSRPGTTVAGLALTEHDGELCWLAFHVGDSRIYGCSEKGLERISTDHSVVQALVDAGAITEERALTHPQRHMITKALGFGDRGGADFAFLPVVPGQRFLMCSDGLTGEVLDARIGELLRGDADDQSLADRLVAEADPAVAQDNVTVVVVSVSSTAG

>tr|A0A853UEP9|OS=Dermabacter sp. HMSC06F07

MRSQRFGLHAVLDQGDVNQLVNADLSLRAERHGGDFGVASTGATDVGLVRSINEDSFISYDPIFLVADGMGGHNAGEVASGIVADEFASLAGGDYVELEDVADAMVRSYERIASLDDDSGRSAGTTVAMVALTLHESEPHWLVMNLGDSRVYMLRDGEFSQISVDHSVVQELVDRGEITLDQARVHPYRNMITRALGAGPDTRPDFWLIPVRLGDRFLVCSDGVTGEVSDGELHTMLQETSEADFVAQRIVETAVAHGGRDNATAVVVSAVEPDDAGVYGEHIFTTVDRLELPVITDEDALDDAATAQLEGEPDTGDQSDRGDGAEAVGSFEAEQEHPEGA

>tr|A0A857MI85|OS=Candidatus Mycosynbacter amalyticus

MTFMYNIIIMTRQNEGFNNFDDISPNNPEWNHARSIRIPDFILRQRPHPNKHVPTQGHITPPTSHVQNQEIVSSPRFDQPVESLDLSNLREHYAIHTKLFGTTEQSLRKHSSDDYIFMDAENNAAGVFDGVGSAVKADEAAYAAGAAAKKALARDHVLTEKRAANLMRDALFAAHEVVCDMDATVPAGREAPATTGTIAQVFRNFDGDQYVAVASVGDSRAYIHSPSHDYFSCITTDQTPSRLPQDALRARQSRLDHVERRDDYDTLSPEDKRAWQTRNIIGSALGSERVIPQIILHKVSPGDRIVLTTDGVHDNLSAQQMRQYIGGSDAAAIPKSLIMSAQDMEDSFRGKPDDMSCVVIEVQ

>tr|A0A8A2WGK9|OS=Glaesserella parasuis

MIELITSSFFSLPKEEQRKNQDSILPVKKVGDNYLFAVADGVGSYAGAKEASTIAINYLNQLQEDQLHNIDNIFNNIRNQIIELSDSISEFDKAATTLTFGIFNHNGLLIGHIGDCRLYVAENGKLRQKTKDHTSHQRLLDKKIYTKKELKEISGKNVINEAITKRFEMNYDTLFIPMSDIRRTDDNVVSFYIMSDGAHQYWEHRPRFSLNTISHTDSFSSSLRKRIEKNPTDDYSLVAVQFKIY

>tr|A0A8B4GMH4|OS=Serratia sp. JKS000199

MNITIASTSNQGGRASNQDQTGEVLGNRAACFVVCDGIAGFPGGDIAAKLARDTILQNFDGEKHLNAQSIRQHITRANAAIHQQQRQSDEYSKMGTTLVSLFIDRDYQLAYWAHAGDSRLYLFRRGYLHAVTTDHSLIQQMQDAGYQTNGINSNLLYFALGLNEERDATYSDVLQLEDGDVFLLCTDGFWHSFSQAELEQSLHMVNSPSEWIALMQQAWKKNNNSDNYSAIAVWIGSPQETTLLHSLADAERFLIRD

>tr|A0A8B5WTZ3|OS=Candidatus Competibacteraceae bacterium

MNDRPGLPNLARHLFAPTAATHPPCPLEEQLTALSSSQALDWLERMALGLADLHAAGWLAVNLEPWVWGLGDSGPVLRETGALTPAAAPVAPMDSGYSAPEAARGGVLTPAADVYGLGCLAYRLLTGRPYARFESSPADPPLPLRGLFDRVLAEDPARRARDMPAFVAALRRAREELAPVVAVEVAARTSAGRARECNEDAYFHGVAGDFGRGETGLTGLFAVIDGLGGEQHGDRAALVAKQTFARGYAALLRGEAVDPLALMRAANTAVMAMAEEAGTGRAGAAASLAVLEAGRIRIAHLGDTRIYRYRAGRVEQLTEDHSLAAVLRRAGVLSEARARRSKDRSRLEHCLGQPNFPTDRAAADVGAGFGLEDGDILLCCSDGLHGWWGDELSAEQERAYLAEVLGDARLSLDRRAARLLADAMRRDGGDNITLLLAAYRLLPRYPEEAPP

>tr|A0A8B5WUT7|OS=Candidatus Competibacteraceae bacterium

MAHGLSKSRLQAFRQCPKRLWLAVHRSDLQEISLETEQRLQIGFQVGDITRTLHPDGVSIDTPDPREALALTRRALAAHPDRPLFEAAFGPAVARSGGTRDYRRAAHSSRLSPDIQARPGGAGDPVGRGRTRSGGGCGTMLTLAYTMHQGKLCRQQQDCILIDGAIHQDRVLPIAAWSSMADEVLLAVADGVASSGGNRRIAPQQASRIVLEELVKAVREHPEWLQDGFVANRHVRHVQAQLSDKLADNPKTYGAASTLAVAHVRSGRAAVLNVGDSRVYQANREGQWRRLSKDHTVLQGMIDRGEASPAIEFKHTLDVRQVASDRSLVTYGAASILAVAHVWDQCSAILNSNDLRVYVDG

>tr|A0A8B7NMC2|OS=Hyalella azteca

MSRNGAYGSQGGPSFKKKFVDLFRHISHPGSNGKDGREHLAPSRPDTFINKYLLGESRGAQPQILSGRSCDDLPPVRIGRMPCQVYSAFTGPGGGLASVNCIAPTLNISDQDVDFIDEDAGLPDDQPNAYVTCQRPPPRKSPFSDMAMYFRRPSMATKPDIRSIQEDLQSKNNNIIVRDDSGDDKSRTGLKSSDFSGKAYGNSLECIVPSWRGEAPSPLSPNLTRAPPNAVIQFGSNPLTYELSIGGTAYAGVDCSGTRSCENICSAASPLSNSCSSFCGGSLGSLEELGVARAQTGNDPVLFNGNDSKNCAAASLSHLDGSEQSTSVPIPSVCGALNVTHEPPFSSASLPTSSYLNYISNMDQNFNPVNDPCIKKSPPSVTRSNPIGRHIMSPTNVDHSETCSAPTTPRSDPGDSQVCHPQQSSISLAPTELPTKKHQENRAVSQTPLQQKLLQQSIASKSQSRISNSNLNNNIRNSHGDSNLNSSHIVNSNDINFSPACKANNNVVGSDLTNEGGLINHRAKFLTLDLVEGCTLSAPHPALKRSEQEEVAGVLNWNRPSERAFGLSTTLYERHPITRERAGNPIADSFGVVARYNSALLALADGVNWGEKACLAARCAVQGCLAHLNAAIFSPTLAHPPRTTTDLFVALLRSFHAAHNLILQEGGQLTTLTAAIVAPVSKSGRYVVCVCNVGDSLAYVYSQQHGVREITQASHDIHSMRDMRDALGALGPVDGLNPELNNLTCSMTFVEEGDLVFLTSDGISDNFDPVVGKFVLPKKEESGEKREKTESECMQEEKKKRQKQKMNLQAQEQQQKPNFSLTSKSKSKQSRRQMQLLQKYEQICQQNPRKQSRGQRIRHQQLLSVTNGFKQVVSRSKSQPSVLGASAAPPCGSAAPKQASTLGSAGLSGNLSLTENKNSSSGDTNKNLAGNRSYLQDSEDLVPPEVEAHQRHELTLLRMEDLLRRGITTNKPVVNAQTLCLEMVHFATKLTLAKRRVLEDPDLYPPPKITPSTDGVSAAGTEQKPGTSQLSRGEQRNRRRK

>tr|A0A8D8RX07|OS=Cacopsylla melanoneura

MMPSFRQKFVGFIRQLSNTSDSPRDSDVPDNVSECFIQNYLKGSDKVNKNEPVILHGKKPYEIPPLTIGEYATDEIKAAHTGPDGGLGSVNKKEEENGNNIIVGTDNWNNAHERSYGKSCSLYEVHPITELRAGDPIADCYGILVRENSAILALADGVNWGVKASIAAKAAVHGSIDYVNQHVFGMASPKPTTTTEVFISLLRSFHTAHSLILQEKGMLTTLTTAVVLPLADSQGKYVVCTCNVGDSLAYVYSPKHGVREITQGSHDVHQMRDMRDALGALGPVDGQNPELSNLTLSMTELEPGDIVFLTSDGVSDNLDPVVGKFTGLPTVEAHQRHKLVLLRTEDLLRYGVSGKGPSISSARDVVTLLLDFVSRLTAAKRHVLEDRDLYTGRDGKVLSPSEQVARRREVCKRLHAIPGKLDHATVVAYSVGQWGEGEVKGNYAWVEEDGEEGEDQGIEAEVDEKNLENLKKDEKEDGNNRILDGEENEQGENERVEDSKEEENGEIGTRLMEMKGNERNVKERQDKKELREDSREKERREREEKRMRNEGRDKEVKDKRSKENVNASNKTQGHSKETASKVDLKEELGNRKQRKEDNIRKGQEKRSESTKGGKNVDSQSGKGEKKESIKTESGESRNRSSSVSKRREERKTGGLKGKDERALMKGKEEKGKSEKGEEKEKGKREPG

>tr|A0A8E0KJA0|OS=Brevundimonas abyssalis TAR-001

MIAAIADGVGGARGGRVAAELAVRSFIDGYLDQKPLSGVAASATAALRGFNRWLHATGRSDPKMEGAAATFTAMILRGREATVVHVGDSRAWHWRDGVLTRLTDDHVLNRPGQDHMLFRAVGIESDVKLDVRVQALEPHDRLLLTTDGAHRALNEAEIGRILGRRASPDADAQAVVQAALAADGQDNATAIVIDVIRTGALDWASLGPAARPCPCRRRRRPARSWTTGGWSGNWPTGGTRGCFWRGIGRGATPWC

>tr|A0A8E0R4U6|OS=Faecalibacterium sp

MRPRARPRRQSLPPRSRPPASRRPRRRTACSRPTSPRRTQRPPRKARPRPQIREKGSCMKLSKKPARKTADPGLELSALFFVNGEKKQDLCEDSYALGAHDGEVFLGVFDGCGGSGARVYDVYGGHTGAYLASRIASGAAYDWFQNEEPPAALERDIARALQCYKENASGSSGIRSSMAKDFPATAAAAKCSCSPSRVDADFLWCGDSRGFLMTADGLKQITVDDVQHTVDGPDMRSDGIMTSVCSASKPFHLHEKQVSCSQPCIVLTATDGCFGYVESPIQFEALLLDTLLQSSSIEDWREKLFGILKKISADDYTMCLACFGYASFADLKKAFEPRHKALLETYLAVWNDSTQEQQQQLWLEYAADYLSEQDDVK

>tr|A0A8F4FW77|OS=Aquihabitans sp. G128

MGPAAAIDHGWATACGPRSENQDRGAAAPAWLVVSDGIGGHAGGATAAALTVDAVAAVLAPVAAPDGVGPEDLLAAAVARANDAVRAGRAADPAVADMGATVVLAVADPAAVDGSRWLVASVGDSPAWVARREGVQQLTHDDNVAGELARTGAITAEAAEHHPGRHLLLRAMGLEATVAVTPIEVVLGPGDALVLASDGLSGVLGPADIHAAIATAPTMAGAAATLVERSLQAGTRDNVTVAAVRRPG

>tr|A0A8F6Y9V0|OS=Gymnodinialimonas ceratoperidinii

MSPTGYDVATALWQGARPYQEDTLLADFHGGMDRGFAVLADGMGGHAAGDLASRLAVIDAASHLKFLIHDGPALEKTLHAELTSAIETANEVLRDRAADDPRLKGMGTTFLATVIFEDRLYWASVGDSPLYLWRENGLRRLNADHSMAPVIDQMARAGEITEAEATAHPDRNALTSVLMGRPLKAMDVPETATVLDPGDVLVQASDGVQFLDDKKISAIIGKAVAAGESSAGIARALIAALQERDDPTQDNTAIMVLQLTHAAGAGGGAHAGAAASGVVASERAVGTAAATTPRAATPTDIPRPAGARRWPVPVALLSGAAAMAAVAFVAPGVFTPQDPARAPETLEIAAAPEPAPEPEPSPEAAPAPVPAPPAASLAPGTASSGGEGSAGSVGTGTFSAVEALTAAPAPVFDDSATAPTTAVPTARPAPPSAPVAEVADAVAAPVAVLPVKFAARAGSPSLVPQTDAPAVAPLREGISAPVAPLVPEAQPISEREQRLPSASDASRRSGG

>tr|A0A8G2C4B6|OS=Desulfomicrobium norvegicum (strain DSM 1741 / NCIMB 8310)

MKSASPAAALTVESWGMTRRGHACHENQDAFLNWPEHLLWGVADGVGGSGNGASASRLLVRYLMRTPAPPSLDGHVENVTRLLARSNRELRQGGLGDAASTVVVLLIHGGGAACVWSGDSRCYLLRERTLYQCTRDHTARQRTIERDELTHHEAERMVRGNVVTNAVGVRDTLRLETTRLSLRRGDRFLLCSDGLSDSVSPEALATHLGRPRARDAALGIAETLDGKEQPDDATFVTVFLSG

>tr|A0A8H3CEI3|OS=Rhizoctonia solani

MSNTINLYPLSNFTFSTKEAQPEEDPSVSARLQRLQNNYEDFGMRRTVEGILVVHDHGHPHILMLQIANAFFKLPGDYLKPGEDENEGLKARLDERLAPLPGSTQHLGQDGDWEIGDCLAQWWRPNFETFMYPFIPAHVTKPKECKKLFLVQMPERKVLAVPKNMKLLAIPLFELYDNAARYGPQLSAIPHLLSRQVVDVTESIGELMSCPASTLPSTLPPYVLDYSPRPSARKVPQSGQSQEQPSGSPATTLPAPFVGAQAPWVSNSPSLPSYSVPPPLWLCPPLDAAWFPLALGIDVGPGPWDLIRHRKQPIAEPEPEPEPVPWSFECGAYGIPKKAKRKTTDEDLTMAVQVGEDSYFVRPDALGVADGVGGWAHHHLRADSARFAKMLMHNCATEIANPTRPQDAYPSPPLTPTSDISHLASVLESVSLEPEVSPREVLHLAYERTVATFRASGTAGSSTALVALLRDGELSVAHLGDCMLAVVRDGKFVIRSEDMQHSFNFPYQLGPHSSTTPRADAQLIKSKVVPGDIVILASDGLGDNLWDEEVLCEVERFQASSAESANLLSPQALGEALAQRAKKAAQGSKDVPFGVRGRAAGVQFVGGKTDDISVVVAIVRGPTSSAQAPKSTPVPTQQKPTTS

>tr|A0A8H7LHR7|OS=Rhizoctonia solani

MSNTINLYPLSNFTFSTKEAQPEEDPSVSARLQRLQNNYEDFGMRRTVEGILVVHDHGHPHILMLQIANAFFKLPGDYLKPGEDETEGLKARLDERLAPLPGSAQHLGQDGDWEIGDCLAQWWRPNFETFMVPGRKNIPPRLIYDDRLVERVPLGSTSDQGEISPSAAPIPPCWVKRGPLYLFLLPFPFMSPNLVHVCDPISLPRAWPGAEQTSHHGWTSGSSGSSWSCAGLEPEMQMQMELEMELEMSRRRDSPRMCCAVLRGMCFELVRFDKLYREIGSHRGREAQLGSIGWLGPGYPLRLNCIAQDEIARILYPSLDLSLVLFPCYLPPHPNLSYRTTLSVQHGLFLDLKYGPQLSAIPHLLSRELGTGWMMMNQHIKTAVVKRARSLYTASALPQTYLPNIMFDYSHSPRPSPRKVPYQQPTGQSQQSEQPSGSPATTLPAPYYTSQTAWIGNASQTPTYSIPPPLWLCPPLDAACFPLALGIDVGPGPWDLIRVGREPVVRKEPEVDVVPWTFECGAYGIPKKPLGKRKSESEDLHMAVQVGEDSYFVRPDALGVADGVGGWAHHHLRADSARFARMLMHNCANEIANPRRPQDAYPSPPLTPRSPSTDNDISHLASVLESVSLEPEISPRDVLHLAYERTVATFRATGIAGSSTALVAILRDGELSVAHLGDCMLAVVRDGKFVLRSEDMQHSFNFPYQLGPHSSTTPRADAQLIKSKVVPGDIVILASDGLGDNLWDEEVLSEVSRFQLSQILSGDQDVNPQVLGEALARRAKKAAQGKADVPFGARARAAGVSFGGGKMDDISVVVAIVRGPTLAAPSTPPIPTPAHS

>tr|A0A8H9CM80|OS=Pseudomonas sp. OF001

MRVEWTSQQGSQTRTNNDAAAVGRKGNCLLAMLVDGAERGEGQALARHWAISIITDALAADEPPEASALVRLMQARQRELRQHYLHEIASYCCVRLDLNSRRLDVLHVGDCLAGVCQPDGQIDWLTIPHTLSRQPFYHSTPQASDPASRHLLTRSLNARRFCAPEHRVTMLATGTELLLCTDGYWYEHRQQGVALSRVHDDASELRLSPGAMRVIQAPDSDNLFIIAD

>tr|A0A8H9LKC2|OS=Streptomyces gougerotii

MSHQGDRRTGTSAQEDAWWAELYDDVAEDAGPAPGAGDSLDDRFASAAGTLSSVPPQAPAPGQPGAWWERPGAAAPTGARPGPVRTEPGGAASLPPQAPEAREGSGRHPRGDAAREPAGGGDRAAADRPDRADAGSATPDGTGVPRAPDPDTDTDTDTAGSPDGRPSAGAPWSGTGDGAERDGPRRPEAGARGAGRSPSKGAARPAAADGPRTGAKGADRGGRGGGLRGTGSPGRDADAPPQAGTPARREDGPLRGEGAPAGPGREQAPPGEGEAEESPPNRHAEAPRSGPGVFDQFADAAGPGGRPTGPPERPSAPRAVAETEAAPRGTDTGPDAPGSASARPPHTGPEPADGPPRPPQAQAPTDGRGTGEGPSAQGRPPAAEPPRDPGQDAPRHPGPEPDPAAGRPRTSPAQPPPGTGPPWPLGRWAHRSSPTTPGGADRSGPVAGTAADGTTTNGVPEASAGHLPPAGPATAPGPKEAPVPAGDPRAGTARPRRTPPFDTWSASGGDSGETLWPAAMRPPHAPAPFTDGCPDTPAPPAPGPAPDEDASAPVTELPPAPERLPHRRDRRGERAVAPAPVPPAGGPRVPGGSGAPPGPRAGLVPEPPYVGDGPPTYDAEPAALPAADPDHLDHLVPDTVLDGGRHGSLTLRAVSLRGDSARFRGEPRRDALLTARFGTGQDAVVLVAAATGARSATEAHRAAAEACGRIARAVGRSQPRLAGDLRDGRRAELASGLRRLTDHVLGRLRARAAELGVGPEAYTASLRCLLLPADPACATRVFFGVGDGGLHRLRDGTWQDLEPRPGEHGDAVADAGGRAPVDLGIAVPPGPWEPAVEPVRAPFRFRASVGRPGDVLLLSTAGLAAPLREEPAFAARLGARWGEGPAPGLDGFLADARLRVEGYADDRTAAAVWEA

>tr|A0A8I1NQZ9|OS=Hyphomicrobiales bacterium

MDNPAWMICGASARGAAHHRSGAPNQDAIGWVAPNWGETRATLVVSDGHGGRRHTHSHVGSRLAIESALAECARLSETADETGDFDFLERCHRRWLAAVADYATHGPDRPDDPVAYGATLVGVHFSGSELFLFQIGDGDIVVARASGKLEKPLPDDTGLIGEQTHSLCEQDASKVGRVRRMDAGGDDPITFVMASTDGVAKSFRDDDAFIDLAGQFRDRIRSGGMASIGERLGQWLSDLSGHGSGDDATLGFLCRMTTADETATPPPISVDRPAAIETTSTRPVVIAPLAFLAGVALATGAVAFNPWHPWKPWVEPIVAAAPTKPVSPQATEAPAKPAEEPKPQPEAKPVDAAPEKPAGVPQAAPPTATEPVKQVAPTQDAPPAPESKPLPEAAPAPAATPIPAPKPARPEDKPRSDPAKPETKP

>tr|A0A8I1TTM4|OS=Nitrosospira sp

MGKESRGALTREWSVGARSETGYVRNENQDRMSCVGTPAGYAYIVSDGMGGHRAGALAAQMTIETLTRALPGIQSIPCAPAQIKQALEEANRRVYEQGHSGNAETQGMGATVVVLFVAGPQALIAHVGDSRAYIHAGGTLRQLTRDHSCVQKMVDAGLLAAADAASHPDAGVLDRAIGVLPDVKVELSGWIPLHGGDRMLLCSDGLHGYVTDSEIMTVLEYKASPQELTNRLVDLALQKGGEDNITVQLLEYGRSRKSAWSKIIRRIACKKTRRAAKASGIQHSAPFPLPASSRPIVPMIRNSANGLQIKI

>tr|A0A8I1V3L1|OS=Acidovorax sp

MIAHIAGESHRFQLSVSAITDKGGRASNEDYLGMIDLGSRGFCCTLADGAGGHGNGALAARLTVDAVLGGYRENPMFAPASLASLIFRAEHTVSGEQPTSISRMHMSATVVLLCIDPVTGRALWAHWGDSRLYWFREGQVHRMTEDHSVVQQLLHAGLYANGDPRSLPNRSVLAGAVGAESQVPPTVLHESMELTGGDAFLLCSDGLWENLHESAMEQALQAAERPDVWLQDMAEAVRSKGRPHQDNLSALAVWVSTNQTVN

>tr|A0A8I1YZX0|OS=Chromobacterium violaceum

MLRVEFRASVPKELEQPELNEDALAIDEAAGRFAVSDGASTAYDSRVWAHMLTARFLQSPAVNHEWVTALIREYRASYDFDQLSWIQQSGIEQGAFATLLAAEFQPERVELELLCVGDSLAVLVENGTALKTFPYSHSEQFDVDPTLLSTRSDANEFITASQFYSQHSTTWSLHNNSVVLLMTDALGKWLLEASVKDHSTLQQLLTIGSEEEFEQLVMTLRETKQIKVDDTTLVRLAFEVKV

>tr|A0A8I2FV13|OS=Candidatus Aminicenantes bacterium

MELFYSGLSDVGNLRENNEDYYFAGKLGEDEYLFIVADGMGGHKAGEVASRKAVSFFVRELEKGIGDNISEDLRRIILAANEFLMHEGSRSAVKNGMGTTLSVFYVQGNLGYIVHVGDSRIYRFTNPDEKKGNQFVLEQLTEDHSFVGKLLKEGFITEEEARRHPRRNVLYQSIGLKKDINVQVLKPIPIQKGQKYLLCTDGLYGVVPESEIAECLKEKSTAYIVRVLVQKAKANGGPDNISTIVVSTEKDKIFGEDTVLEDTIRIVLRSKTKKRKKRKRSFFILLGLLVLLLVVIIYWLLTTISTSQHPLPGSGSGGESVTETMEK

>tr|A0A8J2A4R0|OS=Amoebophrya sp. A25

MDRSNMRNMTVAVAGRQFNPLEQDESVLRKGERSNTLPTSFRRGDVEIKAEDLKELEQRQQALKGRQRRGSITLSKVEEKDMGQFWEDCGQIELHMHGKLGSENIPGAEREAVLYKLKEPKLLQEKFKFIYSCVKGSKGYNDNSPNQDNFSYTVFNGWDIIIVMDGHGPCGHRVSARCVETIAYYICKSAKWGKDMKGCIREAFAKSSEDLLGYAIDQDIDVQASGSTCVMYMQNGNSFFLVRLTGHFSSAMISQIPMHESGDILSSRFPYGLVIVANFLAFTNNHSNLGKTPFAPGDTYYTANVGDSRAIIGYENEKDVVFETMDHKPSSPEEKKRIEASGGEIRTLRYDDFSVDRIFVKGYDYPGLCMSRSFGDECVKPCGVTSEPEISGPTKIDLSRKPFLVIASDGVWEFIESAWCIKAIVKKLSQEPAERIMQKLSKEARRRWKQEDYEHYLSSITILDPSPPSSPFPVAFSDVPLGWVYYYYFYSYYYHYCCCWAVIDFGRSLHVMALLGD

>tr|A0A8J2BJV4|OS=Candidatus Methylacidithermus pantelleriae

MKEDSLPRRPVPVLVRDAAEEQRGSFFHLGIVSEKGNRPAPNQDVSGWVWAGELSIGVGIADGMGGHKAGERASWLALGAALQTLAGQSETVSPRERLAQAFLGAWRALNEEAKHDPAVRGMGTTLTLVWLENRQAYFGHVGDCRLYVVKGKECKLLTEDHTLASELEKTGTPLFSREELSGCRHVLTRCLSPMHEAAPDIDSLSIEPPFRLILVTDGIYPWLAVKEATFLLTDDSPLAAAEKLVRIARERGGTDDRTACVMDVFEPTDEKLS

>tr|A0A8J3FY25|OS=Longimycelium tulufanense

MTTATNAPVFGCASEQGPKRPRNADAHAHHVHHGRLAVVVVDGTGSAPDVAAFAAQAADTTARVAARRGPVLGVIHAAETCMDTDNPIPQSDGTIVAATAEEGGYWLVGWAGDCVAYAVDHDGAVTRITTPETEGQRLRDAGEPENVARKHDHQTLNSVGRIHICGVRGKASPADTPRILLASDGLTLKTDEIAAILRDHPNDQQTAAALLVKAARKAGSRDDITVLVAVHPDTQQQEEQVEGGDR

>tr|A0A8J3HQC5|OS=Catellatospora sp. TT07R-123

MLELSTDAPVIGRVQPPGSGQVSAGGVLRTPSVALDGLVAGRYQVAGASVVGRSHLVAGSCRQDAYAFGLGEDGRLHVAVADGLGSRPTSQVGAALFCQAVMRQALHGPQTTDPRRLIEVAAARTAEQGLAAYGLAERDLRCTVVVAIFADDGVTVARVGDTSAFTLHGGEFGEVFDYDDTEAVNSVSASVPGHRPDAVELVRLGRPQVVVLATDGVAMDLRNSAGLRGWLAERWAVPVGPYAMGDSLRYRRQGSFDDLTAVVVWCPTPEPQPEQAAEQTAEQTAAVPAAGPGPDLGGDVADPGWNDSIWDVGRPGPVEDTGPAEADGEPGPGESQPSGGAGAEQR

>tr|A0A8J3IGS9|OS=Reticulibacter mediterranei

MLENCDVLHLCTILEELIRDADEQVRTIGAQRAGTDDLATTVALAAFSHHPETRNYTMVFAHVGDSRIYLLRNDEPLTRLTNDDGLLAKLIENQIINETDAFRIDQAMTAEQLSDTEISYFRLRGGITQALGGPLPPTIHIDQVSIRPGDRILLCTDGIHDNLTDAEIEEILRKGPRTATARLLVERSIERSHDRTNTVRAKPDDMSAIVLSCRF

>tr|A0A8J3INB3|OS=Reticulibacter mediterranei

MDSRPSKHMLVTSGRLYSLLLYVYPKQFRQKYSWEMIQTFRDCCRDSFERCGGWGLVKWWLFILYDLIMTAILEHLKAVITLCKRLLGLEREFTMLDNLLRLDIALQTDVGLKRPHNEDSMVSVVPDDPEILSKKGALFVVADGMGGHAKGEVASDMAVKIVNASYYEDDDDDIASSLSRAVKQANRMIHHLAMVEESGMGTTCIASVLLNDTLFIANVGDSRAYIIRNGLPRQVSQDHSWVAEQVRAGILTREQARTHERRNVIYRSLGCCAEVEVDIFTEQVQDGDVLVLCTDGLSEVIDEDELCSIVEQYESQESVTRLIARANECGGPDNITAIVARVSLPQSA

>tr|A0A8J3IXD4|OS=Reticulibacter mediterranei

MRSQFRIVPRSDEEVEEEALPETAYSTQPYLELENGEVEEPPTLPTTEKLPEDEDDELLVALRLVVGIGLDPGIVRKDSPNEDNLLAIQGLHDGESGPVPLGLFVVADGMGGHADGQEASRLAIQSISDVVSPAILRNAEPDETFSDLLKDGAHRANLSIYRRNREQEHMMGTTLTAALVVGTQAYVVNVGDSRTYRYRPGEGLVQISRDHSVVARLVEAGIISREDIYTHPKRNQIYRCLGEKASLELDYFHIDVQAGDILILCSDGLWEMVRDPDIENIVASSSPHASQVSSMLVQAALTRGGADNISVVAVCVLPAE

>tr|A0A8J3J1T8|OS=Reticulibacter mediterranei

MSTSEGMVLAYLRNMRAAQQNEQPEHARHLFNAAMSWLEQCDKEDEQTNDSLQLNVGVGLDAGTNPRKSVNEDYVFAVSMLNRVQERVGLFLVADGVGGAIDGQEASRRAVHAFVNEVLPQLLEEPLRREALRDLLVAGIQAANQAVYQRNQDPMLLGSTMGTTFTAVVSVGCDAYVSSIGDSRAYVYREGTGLMRLTQDHSKVAELVKLQVITPREAYYHPERNVILRALGGRGLEIDEPIQFQLQKGDVLLLCSDGLWEMVRDPRGDDMAAILKRNLSAEEMAENLVRLALSGGGPHNTGGGHDNIGIVVVKMEVDVAECETMMWPPTPSCTETMVVSM

>tr|A0A8J3MDS5|OS=Pseudodonghicola xiamenensis

MDIVAGMQSLGARDAQEDAFRIVPPPPVDRATDLLILLADGMGGHVGGEIASNLVLETFEHHCIAVSSTPHPRQRMTEAMEAANAALARRVRQEPGLAGMGSTLLSVIKLGDRLSWLSVGDSLLYLWRDGGLRRLNADHSVYGELTELVRAGQMTAQEAQSHPKRNALRSALIGDAISLVDCNAIQLRRGDVVVLASDGIETLSERQMVQVLSQQDRTGATQLCADFLNAVEAAGQPRQDNTTVVVYRYDPGRGNGASSNSLFALPEGAPTGLTRRWGLAIGVAVLAVLVMLWGVFSGPASVPAPPVVATDPGPDSRVIAGGHEADAAQETIIKGEDSARPPTGTEVSPDAVKDGAVPVAPAGRPQPGATDLDRSAPHDSPDAATGSDTTERPDNPGDPEAVEGAEGRDVEAGLRTSPRPMLRPGREGREPVFAAGAAGDLGRVVD

>tr|A0A8J3QTW1|OS=Rugosimonospora africana

MNAADLAGSMQPAGTADRAGSAPSVILSAAVLTDRGLRRDGNEDAAFAGRRLIVLADGVGGLPGGEVASQIAVDTLTPLDDELPAGRSPLDALQAAVEDARVRIGEAAAADPSLAGMSTTLTAMLLVDDRITMVHIGDSRAYLSRKGVLGQLTRDDSYVQMLIDEGAITPAQARVHPQRSLVSQVLQGQPAHPAYTVLRPDPGDRFLLCSDGLSDVVDDDRIALVLRERPDRDECARELVRLALAGGGPDNVTVVVADMSAAVADVTAVADVTAVPDVTAVADVSAAPPS

>tr|A0A8J3RC47|OS=Sphaerimonospora thailandensis

MTIALRYAARSDVGLLREGNEDSAYASGRLLAVADGMGGHAHGEVASSVAIAAMSSLDRGVDHGDAFGGDLLGDIEAAVRDANRQLHAMVARDPSLKGMGTTLTAMLWHGSRFALVHVGDSRAYMLRGDELFQITHDHTLVQSLVDDGRITQEEAANHPQRSILLRALDGSGEVDPDLQGREAQVGDRYLLCSDGLSTVVSAETLHHTLTTVEDPEDVVRQLIDLANRGGGPDNITCVVADVIDVGDAPPPVTAAAIVGAAGSGRAFGPVSPSGGPAPGAMAATAPQPVILDEDLDDEPVRGARSSARRSRGSGSEKRRSWLRPVTAVVVIGAVLGGGGYFGSQWVQSQYYVGASGDKLVVFRGVDVQLGPVSLKDVAYAEEHARVSALADVQQDQVRSGIPVDDLRAGIAMIKSFNASDGAKQGTDQQDTAKQETAKQDTNQQETAKVGTSGQNASNPDANPDAPTSTSTPTSARSGQ

>tr|A0A8J3WXM0|OS=Planobispora takensis

MTAPDDAVATPEAGVGEPDDAVTLPVAARRPAATPEAGESTPDTGDVPDAGDTAGTPEAGDTADAADAADASGASDASGVEAGAGEACPRCGSSVHPGEFFCEGCGQPLDGAGCVRCGPAPVDPEGYCELCGLRQPSESDHAETEAGGAAGVSDRGLRHSRNEDAMALVAADGVTIGVVCDGVSSSPRPETASSAAARTGATVLLEALRSGADPVTATRTALAGAATAVAALGSSVDDAPACTYVSALVGTDRVTIGWAGDSRAYWLGAAPTPPPVPGTDSDTGPMPVPDSDSDTGPMLSPVSGSDPGPVPLRGPALLTEDDVVSPGLLSAWLGVDAGRITAHVGEFAPEGPGVVLVCSDGLWGYLEEAADLAPFTARDTPLEAARALVRHALAAGGRDNVTVLVIPFHPPPV

>tr|A0A8J3ZGE3|OS=Virgisporangium aurantiacum

MIWETHGTVLAALITAALVLVLVAVVAGAAWLHRRPDFSDLPPVADGRHTTEMFGHTRAFSLPSAVAAVVPCQIEADAPSGAPPTGHVPDPSRPSPEPPEDVRADHPRPAFTSPLRPETAVPPRFGVGSTAARLPWLLPEEHAAPSGVAADQAVLGALTVRAASVIGPGNRCARPARPRQDAYRIAQDRAAEHLIVAVADGMSDSARAEFGAMVAVGKAVGLVRNRLDRGERVDDLRVRGLFADVAAGILAAADERGLGAEDVRTTLTVAVIPTGPAAAPRTAWVGHVADTTVWLCDARSWQRVTRNVKDSFNGSALRTFMPHHPDTALSHTFTVTAGATVAVFSDGVADSFDEVPGAAEWLADRWQEPPPLASFVLDVDFDAKAQHDDRTAVVVWCDREPYGGDRGVDE

>tr|A0A8J4A2B3|OS=Virgisporangium ochraceum

MNTQRTGWSWQTTHVSVVGASHLRNTVECQDHSRGESDDAGAWASLTVADGHGSEAHFRSRRGAGFAVDAMTEVFATFRSYLDELSISPEVANANTRWSEAPRLLVANWRARVLKDLIADPPRVSDSGDREPRMKRFFDHIAARQGPAERDRALQQFREFESYADTVRSSGSDDPLASGPGSADWDAERLGGWQLRAYGSTVLGVLIGPSAIHWLQLGDGAMMRILGGQPGYLCPPPSDAIANVTPSLCDDNAAYRISVGTEPLLPGHVPSAVILTTDGVPNSFEDPEGFFKFCVDIADRAVGAADLSADLARWLPEISKRGSGDDMSVSMAWATERVVPPGPADDLTPLDASEAALEREPRAAGAGITASARTNADDDRERDPTW

>tr|A0A8J4B2A9|OS=Volvox africanus

MFLNFFRHLGARLKEQSAAKKLECGTLCGKQGAEGEPPEANLHLSKPNTTIIASHSGPDSGVAVYKQFAADLPVTWDNPVDWAYGISVSMYDLGPTGRRNGEPVADCFGILAYPEGAILAVADGVNWGEPPRRAARCAVLGCLNHMHHALKQHEGDSSLDCHTVFRHLVDSISASQKLILQQFGTLTTLVVSVVLPLKWVRHPGRWAAITMTVGDSAAYVYRSSKRTVEEVTAAAHAEGARDPRWCPGALGYAMGEEPDLANMLLCLTFLDEGDFIFLTSDGVADNFDPVIRKVARHQILESADNDLPALSPAECQAKAMDMLRDVIVEADVRLSSGDGSNSNHGLASAGGAVSGLFGHHGHSMTARGLAEQLLQHVYDVTEEQRQHVEARNRERERSLLSREPGTGAGGPNLNTDMKHGGLMGLAPMARPPGKMDHATVAAYQVGRRKPGAVAKSQALMKALDEEQSNGENKLPFLAAQAATMPPPRTLGPAPLAATAAITAATGSNPANASSAAAPTRQQGQQQQPAASPQPAAAVVQARHNDATAVATGPLGEGAAVGAAAATAAATTATTAATTTAEAEVAGAGEKGPAGGEDEGARPPGIRSL

>tr|A0A8J5QKU9|OS=Cotesia typhae

MPSLRKKVVGFMRQLSISNLTAAVENIGHNDNTLTPRSPPADFPGGCFVTRYLNGLEVKIEGPVILHGRNPEELPIKVLEKLDDNEEVFAAYTGPDRGLTTVNLKQKHLSISDPDVDYIDILEQNDQEATVNTSMDCIFSVADNRWLLIRTNESCPIDNSTIKIRAAGKLYEPDKKYSGENNEYTFQEVDQKNSNVNFNGKKITGVKFLCDDDKKSEDFVDMATIDEGIQASSDSGDNVDNSDDDNKIDLIRVSDDTLGNITPTDIDVDEIIPPPKDFANDMIPLRKSKSAPAPFKNAQKASLSSNEMIAGIENWFLPHELAYGIATTLYEKNPTNNVTNGEPIADCFGIAARPNAAILILADGVNWGVKASLAARSAVHGSMEYLNKALFTPSNNSGVTTTKDVFVALLRSFHAAHSMILQEQGMLTTLTVCVVLPITSTDPSKHKKYVACTCNVGDSLAYVYSK

>tr|A0A8J6BZI1|OS=Zizania palustris

MSLLIPLTPSTLYPFSRTLVLLPRPISARFSPSPGRMSPALALRRAASWLSRGGVANAGAPRAACAGVLLGLGGARSTERRWQETAVEDRDVVWRDASGFLGTWGGTDDGAKPSWFRCAASSVFRPAFFVEQRFVRNARSFANGAAPEETSFGAAAASACEAGDHHSDKSAYASDKNKLGDRSLKLVSGSCYLPHPDKEETGGEDAHFIWDEQAIGIADGVGGWAGYGVDAGQYARDLMSHAVTAIQEESKDSIDLSRVLEKAHRNTTVKGSSTACIIALADQGIQAINLGDSGFILIRDGSTLFRSPIQQHDFNFTYQLESGNCSDLPSAAQVFRVPVASGDVIVAGTDGLFDNLYSNDITAVVVHGTRAGLEPQVTAQKIAALARQRAQDKNRQTPFSSAAQDAGYRYYGGKSDQIKEGPDRSELIKIHEAPSERKHTSRGSQLPSLLPPRDRQGEGEREAALLLQSKCDAMDPIMRLLEDDEDETLHSGADVEAFTAALNREVEGSGSGGGSASASSSAAASSSQPLDHGVGHVPQQSNSIFNHSHEQWQGSVKNEIGKQESQQQEQKHLHHTNEQPSRSELVFEGADNKHLQSNTQRECDQPKVKQDSGNNSQQNIAGQQQPLQQMRSQQTPSTNQTNSGPTVGKPPVVAFHMLIPILSHHLDKDKDMQVQSIFTKLRKNEITKDHFLKVIRSIVGDKLLKLAASQYQTQAAQAQRNPQTEPSNYSLLSQVSNPQNVPSSSMSGVEHKTYTLTHSMPVNQAIDSPRPPLFRPSSGQMQCNMGYPASESNMQKPNETINMSDVKRGHMIQSRPPNVHSVSVQATQHSVPHPQTSLPVLGTNSIHARPFPRPVGGLNVPLRPQMADSKQRGQLVQGAITTVARNMATRSTLQTNQSPWQQANKEQKTNSFTPTEHMDKGGGVHERQPSALSTSKSLTATSSSQHHRSHGTQAEANMQIQPAAQTPLPVAASKTPQKKTSAGQKKPLEALGSSPPPSNKKQKTSGGYHDQSIDQLNDVTAVSGVNLREEEEQLFSAPKEESRVSEIARKVVQLEEEKLILQKGPLTKKLAEIMRKCNLKSIGSDVERCLSMCVEERLRGFISNTIRLSKQRVDLEKSRHHTYPLSSDVRSHILRVNREAKEQWDKKLAEDAERIRKQNDGYDNTIVDSEKDKNESRSTSKHAKTYKEEDDKMRTTAANAALRVAAGGYDMLSKWQLLAERNKQRNEGGDSSGSMPGNMLPHKSSPRSGKGSREQQEIKKSGGVRRSTHIKVARSITVKDVIAALEREPQMLKSSLLFQLYGRSPTESSAK

>tr|A0A8J6L3E4|OS=Tenebrio molitor

MHSLWLTGRLISRAIFNGITNLSTAADPNIQRKTDPQLVSAVCGFSKERGIQRLIKGQFGDDAWFTAKHKSADVLGVADGVGGWRAYGIDPGEFSLHLMKTCERLVKLGRFTPTNPSDLLARSYCELLHHKKAILGSSTACVVILNRDNNTLYTANIGDSGFIVVRKGRIIRKSEEQQHYFNTPFQLSLPPPGYQADVLSDQPDSAITDNFPVEDGDVILVATDGVFDNLPQNLLVDELKKVQGERCASRLQMVANSIAWMARNLSFDETFISPFAESAFANGINTIEEYRVGQLYMIARHSLEQSGDGEGVEVVENKECEDPEHGKGQFTEKRIHLSSRLPYWVQAIIPKIFYVTEKAWNYYPFTITDYTCSFIPRFHIEIKTKYENNNGCTENCLNLTPEQLAERLVDHIDIAYDDLSPKHYKEEEDPRFFQSKKTLRGPLIEGWRANYTPIMCSYKLVNASFEVFGLQTKVEDFIQSCIREVLLLGHRQAFAWIDEWIEMSLEDVRKYEAKLQSQTNSILANQVGGGDSPGPNNVVKSGNSTPESPKTPKSPAKKGYFSWF

>tr|A0A8J6XQJ5|OS=Iningainema tapete BLCC-T55

MLPTPFVNILSLEPMNCEFGLCVFLEAINERGEDGEPVVVPYQKTVAMGVFDGLGGRSAGYDGMTGGKIASSLASRITKQFLQQRYGHFNEQDITQLQKDICNLLKTNADTKIKQSRLQGTMAGKRLCTTLALVSISYITPANNSFRLSLGWIGDSRIYFLSPTKGLQQLTKDDLTIENDAFKLIREDPPMSQYLTADMNANWRINFKTEEFSENGCVLACTDGCFQYLESPWAFEKLLLETLIQCQSFTDWQNLLLDAYTKIKQDDVSLVLRSVGFESFHALQDSYKQRFAIIRDRFSYQIDDYQKLETHWSIYKQNYEEKIKSEYLTTKYNQEPLVSHHKQSQKVVKTSEYEDEQQVKVNDTFQQKSFGEDREQLNKKEVVKLLEQGDKYWKMNSYQLAIYQYSNALRLDPDNCDTEWKLGCAYTELGFFEQKVYVNIFKSRHHKTAVNFFEKALSNPYITKTSEDKYIYLKALLGDRNYDKVIQVCHEIIQNVPNCAYAFHVIGYSQKQQDLLEIALENLLKAKELYQVEQKYQQVQEIDNLCNEIEKKLKAT

>tr|A0A8J6ZBN4|OS=Quinella sp. 1Q7

MQWKSVCCAVQGRGHMRRDLPCQDCVARLEVGGVHVIALSDGAGSAAMSHYGAQRVVDCAAEFVAEKFFDIVASEDGRSVTQELLTVVRRALQAESNLRGCELKDLAATLLLAAVSDEKFFLAHLGDGVIGYLSDAGLKVATTPDNGEFSNETVFVTSADAALHMRIFRGDLKKICAFVLMSDGTEQSLYNKHKRTLAPAIKRLMHRTCLVDGDIFTAQLQHALRTVVAENTHDDCSIALLARNSTQLPPLERLILFERRELERITGSPTFRRVREKISRCDRISDLLKRPLTLRQFIRRRDIS

>tr|A0A8J7CXU8|OS=Fortiea sp. LEGE XX443

MLICPQCEFENPNANKFCQNCGTSLTHHVCHECGTDVPLNAQRCYNCGAECGKVWWAIITKEGSGNWELGSWQEDQDDLPTLPLSTEFAPPLSARPVFKVGSYIDQEQRYQLLEPLPITEENTTQTEVCVRVLDCHPYQISPIEAILENQNQGLLPLSGKEVGITQLAKAYLELQSQGQPGIPVIHDAWQESNTQVVLIEYRSDWPRLLDQWKEETTSSLQILHWFYQMTQLWSVLEPVKCRQSLLELSNLRLDEDQTLALERLYVEPLNSETAIELDADAETEEETSAIVKQPLTVKVLGDVWQELFRQSQRTQFGAVLQILDDLELGKIQTIAQLRSRLEAIATELEASHIPSFSPTANQNPTAPTTIQLENELEEDSSAKTDDLPTIVLSMQLSSLEDIGRTDVGRQRQHNEDYFGIETKVDKLELPKNRVLQARGLYILCDGMGGHAGGEIASELAVNTVRQYFQEHWTKDQRPTEESMREAVYLANQAIYQLNQQDARSGIGRMGTTLVILLIQDTQAAVAHVGDSRLYRLTRKRGLEQITVDHEVGQREITRGVEASIAYARPDAYQLTQALGPRDETAIDPDVEFFDINEDCLLILASDGLSDNDLLETHWKTHLFPLLSSGANLERGVTELIDLANQENGHDNITAILVRAKVRPNMEN

>tr|C7N2F9|OS=Slackia heliotrinireducens (strain ATCC 29202 / DSM 20476 / NCTC 11029 / RHS 1)

MAFDLICVRTQGASHIDDGLPCEDFGAVRKGAFYQVFALGDGHGDSNCPRSSLGSQWVCESALDALCSFAEDVRQDGSVPGAAADATDEAVEQEPAPAEEAPEPRRMRSKKMLLQDAVSIIGDEQGNADLTGREALLFDPQQAKMLMRQVAVSIVGDWTSRVNSHYAENPLTDEEWAGCSDRYKPRYQAGERIEHIYGTTLIAGLVTDTYALLLQQGDGRLCVFDAQGDVSQPIPWDDRCFANVTTSMCDEDALASMRFCIWDLRSHPVVAVLAGSDGVEDAYFSPEQMHSFYREQLVYAARSSVAELQTKLQAELPAFSAGGSRDDVTICGIIDAEAVRPFVERYDMENAQVPIQLELSRIEERLPKMQGKMGYLQGNVAKAEKDYRAAVAVRDPLAVRVQELRSDVSNVNSGAAEGFFSLRTLSALRGSLGQQQAKLEEDLKNAEADVVRTKDAYERACKELEDYVALRNGLIAERDGYKEQLKALGASDDSFDNVFDGGARSGETPETEPSVAPDPEPMPGFGPDVEAQPWTDVSVEPEFDDIGSRIHRLSRRFFNRG

>tr|C7N649|OS=Slackia heliotrinireducens (strain ATCC 29202 / DSM 20476 / NCTC 11029 / RHS 1)

MLYYKMSETGLSHRAEGLPNQDRVKMVCSNDVVVLAVADGMGGEALGEQAAEIACSAAYAAACSTIVCDREAFLYTAMPAVQAGFGAAFNALQKAKLDHGWNLGDLGTTLMVAAYNVKTGDLFYGYVGDGGFAVNQAGECFMVEQPQKGDNANQTFMVLDCDHWRFGHIELVQSFYVATDGIGDCIASYNQEHGTQEVSFEGENLLSYPVGWTQSQVDARMDDIFKAPALVKPSDPVALQAYDPAKLQAGSMQLPAAPGETHAANRPYAKRYTETYAARSKARSIPIEGAEPSMPEPGPDPLPGLFDLVDDDRTVVLVWSDSVDVRNEWPFYEQAVYAKVNPAVARRICRNRKSPLTRIRNLFQSI

>tr|C7N9I8|OS=Leptotrichia buccalis (strain ATCC 14201 / DSM 1135 / JCM 12969 / NCTC 10249 / C-1013-b)

MRKEEAKFETRFFSEAGTKGKNNDYFGYTQLDNYAIWVAADGYDEEAGADVAAKLAVSSAIEYFMLRPRFNPDVIKEIMEYANLKVKEKQEETEKYSLMHTSLLVVISNYNSFLYGNVGNTRLYHLRGGYVVSQSRDDTIAQLLVDENALDMNDMKYHRQRNDLLQAIGDFGKIKPNIIKNPVTLQENDMLCLTTIGFWENIDEREMEVEISRYPNKDSLLRSLEHKVMATTRESVENYTFALVNVEKVASPEPVEKDKKKFWIKVGLISLAVLVIILSLTFWNISKRNNIIKRAAVYEEQANDDIVKKDFNNAIESFKLEKAELEKLKPKSRGIIGFFTGANGKRADVEKRISAVNTKISQTSKLQKAFQDINEANQLFNSGNYDEASRKYQEAKYVLEENTYKKDELNTDEVLTTLNARIDSSSKLKEALAIETAGNQAFSAGNYNLAKENYKTASELYLVNGRADYVANIERKIAEIDDKAKTEYNGAMLTENQADLLSPTDTNKSRQSYYQARQMYQSLGDTAKA

>tr|C7NG50|OS=Kytococcus sedentarius (strain ATCC 14392 / DSM 20547 / CCM 314 / 541)

MAIDPRMLGAPSPEVGASSGAPLEPGELIRWAAHTQIPCQDAVAQRGGVLALADGAGVGPAARPGCEHEVDWFSQRLVDAVVDHLSPADALGPVVGEPVGLREAVAHGIEAVRAAHPQCDADTGPWSTLTMARVVGDELEFLALCDSSLIVEFVDGQVLQVIDDRLDGVNAPGASMNAPGGWWSARGDVRAAEEARTGSFPLADVRRAWLASDGATRPLETWHQYDAAEWARRAAEDTLALAHSIRTHETETAAEMTAAGRKAHDDLTILRVV

>tr|C7QHQ6|OS=Catenulispora acidiphila (strain DSM 44928 / JCM 14897 / NBRC 102108 / NRRL B-24433 / ID139908)

MTIETAAEAAAGSPPTAPAAPGEAEPGMPLQLTCPNCGATDLVMEAGARFCEQCGYGVDEPVTGDEEPSEYQGPKPCVSCGGTEIDTEGYCTDCGDLQPRRRDRMEVDLTVVAGVSDRGLRHHRNEDSMALRPVLGLDGENLVVAVVCDGVSTSERPDEASAAAVSAAAKLLLTAIAAGTPEPFAESVNVLAPVETAVMAALPAAAIGEAGAVETAGSAGYASTAETTEPDESAEDSADAGVEADLGAATGAPGTVGPADLRLISRYAVADADRAVSDLARDADHGNPPACTYVSAIVGQEVTVAWLGDSRAYWLDERGTSRALTRDDAQEGSHAIEAWLGADSGAPEPHLARFTPDGPGVVLVCSDGLWNYVEKAADLAAIALPGALAEPFDAAARLVKKALDSGGHDNITAVLVPYPVQAHAEPSA

>tr|C8W3F8|OS=Desulfofarcimen acetoxidans (strain ATCC 49208 / DSM 771 / KCTC 5769 / VKM B-1644 / 5575)

MNLVVNIGRYYTGKADMDEAKTKLIDMGFTIEETRELVMVKAADAGRTTLLEKLNSGSMSIYEFFSEAEKLYEKPNHKIIKTKNDRIIKSSDYDIVGDVLLPTRDIKLNLNYTKDKKVIQTDKIILSFRQSENKQSVIKQTEPPVKDSREKPAPKEEEVVYPRWRDLPPADQEDWVEINDSAFKEWNGWGICAASRRGKTHAHEGSHRDDSFGFDFENGWSILAAADGAGSCRLSRVGSKIGCEMAVKTLKQFLKDYKVKQIEGVNIPEETDLIKIRTFLVDTMHEVLISLKEEAAARKIEFDLLSTTMLIAVHREWMGKSLIASIQVGDGAIALWHGGSSVSILGVADSGEYASETKFITTKDIEKEFEHKVFFAIKPEVDAVAVMTDGISDDFFPVDTMMPKMFEYVYQIVLGENPADKLVDWLGYEKRGSFDDRTIVFLHRR

>tr|D0BND1|OS=Granulicatella elegans ATCC 700633

MKIKNICSIQGTAMENEDALGNIENYFWIIDGATDLYDSKKSIGYSVSEITHLLSEKLKVHCNEKESLKTIFQNAIKEVRNIIGLNDSNHEEYYKLPTFAFIIARLSQRKLEYLILGDCVMLVNDEQLTDHRVDRLFDLGKNEIENSKKGLNVLNKKEILQKIRGLANKEKGYWIGSLDDASIEHALYGNLEVTGDKIVLMSDGFYDYYSQNTTKSFEELIEMRKQSTELDPIYGKKDDASILVVEL

>tr|D0LRB5|OS=Haliangium ochraceum (strain DSM 14365 / JCM 11303 / SMP-2)

MKLIAWAKSDTGMQRDHNEDSFLVDEEVGLFAVADGMGGHRGGATASRLVLEVLQEQLASVNGDIDVVAKKWTSKAESTKIIARAEMLSEAITLDSSWRDQFERAPTAPLGIPAVDPAATTVIRAAAAEAGAAVYRASRDDHDLRGMGTTLTAMFYYDGRMHLVHAGDSRAYLFRDDTLEQITDDHSWIAEQVRRGKMSEEEALASNLRHVITRSVGFESKVELDAEMISVQPGDCFLLCSDGMSNYITNPEIERLMRTTWHSQVPERFIALANQRGGDDNITVVVVQVANDTDDEPSLDESLDDEPTEAAHVVAKAQAIEDADTDGVDEAAVAAAAADSDDEAEAEAEAAGDDGKDRGGE

>tr|D0W6S7|OS=Neisseria lactamica ATCC 23970

MNQIYDETGIPKQLGELLGRGGEAEVYPLEDRPDILFKKYHDSVLQKRREILINKIDVMKRLGQESELGKSKNLSWPLIHVYDNQKNWIGYAMYRADGVTMFHLAHAMAYKKHFPDLDRIKIVAYLINLLKEVKKLHNHGIMLGDYNLQNILLDPRSDKVTLIDCDSYQIRYKDKFYACEVGTPDMTPKEHQNTPYKDLVRTLESEYFSIAIILFKALMLGRHPYDVVGGTDPVQNLCNGKFPYGKDGRGIPKGAWFNIWSHMPYKLKSHFIQTFTEGANNPQKRTDIDTWLRELNIYLQEMRKGWHALEIRPDRPKVSDYKGNKTIEGKQMIAPLLDVPETNDEACKKFALLTVKHAQGILKDLADEHRRPIKDFRCALLITLAGTVHTLWLKIGDGALVYETIKKIDGQRICELKTLGDVGKGEYANTTTFIDEYLTQNDVQMGTMPSEHITALFCMSDGAAEKLVSTDGKRVSTRLSDWAELLRQRKLARSKLSEAFYDKDFQNRHSGDDCSIAIISAEI

>tr|D1B7C2|OS=Thermanaerovibrio acidaminovorans (strain ATCC 49978 / DSM 6589 / Su883)

MGTGGDKAYCVVVDGMGGMIRGDEAAQRALSASVGVLDAGGSPLDAVLAAQAAVHRWASQGGILGRTGATMAVAAVNLRDGTLEWASVGDCRVYLFKGGRLSRLSLDHNVSSEMVLLGRGPVPGPAGEMITSFIGIENLTEISTSEAPLPLEAGEGVLVVSDGVYRSLHEDRIAMALSRGSDARGILQEVEAQGRPYQDNATLALVIL

>tr|D1PS08|OS=Subdoligranulum variabile DSM 15176

MTENFAAYVSQKDGVAVKQKILGCVRDCLHQLSGRLSCKMEDLASTLLVAAVNEEHFILAHIGDGVIGYLKDDELHVASHPDNGEFANTTVFTTSPSALSSMRLFKGKVASIDGIVLMSDGTEMSLYDKAHRTLAPILKRIMQMSIYLPADKLHELLLRSFEELITQNTTDDCSIAILTNDRDCFHGFCSLPTTRQKEILCLSSRASTVRLHRYCEILQLLQDKHTLRQVSLHIHLKPRHTLRHLRRLLSLHLIERQGAYYKSILILKDT

>tr|D2Q296|OS=Kribbella flavida (strain DSM 17836 / JCM 10339 / NBRC 14399)

MSRAEGAAASLPGPTFSFGFNLGKVPDQGEDSDPILRDGPDLGLVAVFDGMGGAGGTVYETPEGRRTGAYLASRIARDVVERRMLELLEPDWNLNGEAAAEDLRGSVQEALRERLTELNAPPSGLRSRLLRALPTTMAVAALQRTQPGGATWACHLLWAGDSRAYVFEPEGARQLTTDDLRDPGDALANLRHDSVVSNAMSADTDFHVNYRRIELRAPFLVACATDGCFGYVNTPMHFEHLVLGHLQQARTSKAWSAALQTEISSVTGDDAAMSLMGVGASLQEFQELFAPRVAELEQQFIGPLDQLEDEVSRAERELEDLRERRLATTTAIWSRYKVGYERYLQPAGDPDDGATGSAEATAAPRASSVAVPDGESDPATTDPAAEDSEETP

>tr|D3D015|OS=Parafrankia sp. EUN1f

MVGGTLRQNADIPAHPPGRSRDDRLEFVLLVLVLTFFFVVCLLVTAGITSLWYRHLRIGAIFFAGGGVVLILGIPLFHLWRRLRARSAGSPIVSQAGQSGGSSKSNGTAREKSIGKSIGKAGGKISGKSGREKPGDQASSSPPPSSLPHATDDGGSGGSGGPPRARPETGGQGPQAPPVTGPVEPSSWLPSDGQPDTGSYSTPRPDHGRQPAEVPLLGQPSDSTRSPWYLPVVSTQPAVAADQARLGTLEVRAASIIGPGHRSSDPATPRQDAYRLGRDTSGRHLIVAIADGMSDSARSDHGATVAVSTAVAVLRRDLDAGATPEDLSAVEVFKEASGTILGSLEGRAMTERDVRTGLIAAVISVHPTRDGHRNAWFGHLADLSAWLQTAPPGTGWAQVAGDRKDGGMDANTLSRFLPYSHGEAVDTYKLLPAGAVVALMSDGVSDALTGIPGAHHWFASRWATPPALPSFIQDISFEAKTFIDDRTAVVVWCDQPAQGADLAAAPRRVRR

>tr|D4T3D0|OS=Xanthomonas citri pv. aurantifolii str. ICPB 10535

MVRRGGCVPGPQLARAHRCSRIRCGAACARRAGRAFQHVRTGARRIVVVATRCGRTGNAWAAHRRDVSGLVAGRAGHHRGDPVNAPYQSAGHTETGKVRRLNEDALLLREDAGLWVVADGLGGHSAGDYASQLLVQRLRALVRPADLCDFLDAIDDILAQINRELLQVARERRVDMIATTVVVLVHDPDFMLCGWVGDSRIYAQHGGPLRQLTRDHVHGVRDDVTQFGSAHASAAAAGVLTRAVGAQEPLFVDWVLTPSLPGTQFLLCSDGINKEIPDPELDAECHRFGDPRALLARLFELAMGRAARDNVTAVVVRLQE

>tr|D5X0Q1|OS=Thiomonas intermedia (strain K12)

MDPTDHYPPSRLPPLPTPVQHRAGTRWRLSAASGLHKGDRPYQQDQLRVIAHPRVQGCVLAVIADGMGGKSGGRKAADQVMLTAQQLFERYHPDSDDPHALLQQIVQESHSMIRLTALSTEQEPHSTIVCALFHADEGRCYWSHVGDSRLYIFRNGRLLLRTTDDSYVQTLVDKGEITEAKARNHPMSNVLTCSLGMSELPSKPVEESSLMPGDVLMLCSDGVWHYFTDQDLEIATSKLQPRECCQYLIDKARTRAGGRGDNLSLIVIELARLPAPGETSSASSGA

>tr|D6K0W1|OS=Streptomyces sp. e14

DAFGLGRTALPDGTAASVAIVCDGVSSATRPDEASLAASRTASEFLLAALPRGTHPQQAMHEAIVAAAQAVDALAGEPAGAREHSPHQNAPACTLVGAVVTGELLVVGWVGDSPRLLGARGPRRPGRPAHRGRLVGRADGRGGPDERGGGVRRRARPRDHRLARRGRLRTGAAHRLVQAGPARRGGGLHGRAVELRRVGGGDGPGAAPGRRDASAAQCQGAGRPRPGRRGPRQRNSGARAVPAPLAGGRIGL

>tr|D6S7U5|OS=Finegoldia magna ATCC 53516

MGYLKAMAGLYSYSYTTLGGSHKKRNKPCQDFSSDYTCDDFSIIAVSDGHGSSKHFRSDVGSKLAAQGSIEEVRKVIENDFDLVNFKENPRKIIENLIENVYKRWKNDISNHMTENPFTEEEIPEDLDEATKEKFFGTLYPYYYISAYGATLMVGVMAEDYYFAFHIGDGKAVFLYEDGKIEQSIPWDEACYINVTTSLSDTNAVDNFRYCYGYKTDDERFVEVGVEKNTKITKKQCESDNLLSNEIQSKEAIEDTLEDKSRKETEYIKAKVMAIFMGSDGVDDSYRVGDNEESLMNLYRNTYLILMKKEHIDEAKKSIELQADKFAELGSQDDVSMAGLVRVEKNDELIDLFHNQYLEAEKAKELEKKQEQIEEKSYHLSQLQVIYDEKNTAFIKNKVASERIINDNTRKIREIKEDYRNFDKENQKAKAQDWEEYNNFLLKISDKIKEKNQEIEKYELEKNLEADDYQKDLEIKYFDGEDNPILKVLAVLSKFKSSNLLYISQDEKRYNKNRKAISDYYEKIRKNQEAYNARLKEMDEKENTFLKKSEEKILTLKEEKDRAKEKLSLLMENEAKEIEALGKKINMLKEEIASLKSDTEN

>tr|D6U8K5|OS=Ktedonobacter racemifer DSM 44963

MQDTHEPKWGIIEASVAGATHKQNQDAKKTHQDPGYSLLAVADGHGSKAYTRSAQGAQLAVESAIQGIEYLLNQLPPLTDISHAMIEHILLTSSPVKIVEQWRKQVEENIKTQPFVEGADQHSYPFKPYGTTLLTALVTSSFIFYSQIGDGDILVVTEDGHVYEPVPGDVRLFANETTSLSSKHAEHDFRKTFKWLSEQDSGSTPALIMLATDGYRNSFTSEADFFKVATDILNLLRQQGPDYVQQHLEKWLQTATDLGSGDDITVVLFWNNRLLQTHRKTITTQGSLQVPADQEPIAPGSEAENIYMRAQEESANISSIQMQPPAPSDRLLFSNTSFHHTPKQIGSGIETLFQGDFKAHKADQRAILVVSQEPGLGDYTSITEALQFASDGSTIIAYPGEYQEQLNIERNICIKAKEPRKVFITSPVPCIKCYAPYAQIKGLSISGQSHQDIRDQPAISLGAGHTEFIDCDLTSKSQLIVGSSTQNTSSLFLQCTIHEGDKTGFLLEGLARCKVKQCTFSNNGETHIVLDAESELESYDSTFSASGNHGIFIGKNCSLLLQHCTIEKYAHTGICLRQNRDALLEDSILQEGQKIGLSIIKGHCMVQGGEIKNQQKHAVHVEQDSTLSLSNVKIQHSRSETPALIVLQQSVARLEACLPVDPNDNWHFCDTGSHVYIQNDALPAPTIEQD

>tr|D7BCX3|OS=Allomeiothermus silvanus (strain ATCC 700542 / DSM 9946 / NBRC 106475 / NCIMB 13440 / VI-R2)

MPEERPAPRYGHQPGPDGATTDPHDEVEAIAAAENEGMVAEKPPPDPAPQESFLEETPLPLTVENSDLAPGTLLTWGDLTFAVGEPYLAGWYRATQDSPRYGRQPDPDGATTRYGHQPGPDGATTRYGHQPGPDGATTALLNPGLDGTLLAEVGGHRLLPRLLYAGPEGVAVAAPEGEPVGRGLSLQEALEVLRPLAQFVYFLELKGLTLLDLEPRSLLRSEGGLRLVPPPRLARIGSRAEPLWREGYTPPEVLAEATLSAKAGVYLLGALLFELLSGTALPAEGPSDLLLMGISLAGVPQALNQLLAPVDERPTPQQALMLFKSLSAPPLPVLEVGAATSIGLNPDRPYNEDAFAYRLERVQAHANHTLLLRACVADGMGGMAAGERASQAAVETFVAPAPPYPLDDPQAQADWAVRLVWEANAAVLQALGGRDGGCTISAVLLVGARYALAHVGDTRAYLWSGLGLRPISRDHSLVGALLASGMITPEKAAAHPDRNKVLRSLGSLRQPQEGYVDGLPDAPTATLLPGEALLLVSDGVWGEVSDHRMAEILSHNLTPQAAAEALVATALEAGAPDNATALIVRRMG

>tr|D7C4Q4|OS=Streptomyces bingchenggensis (strain BCW-1)

MDDLRTGNRSALKSGLHRLTDRGLGRLRARAAELGVDPAEYTAALRCLLLPADPACRTRVFFGAGDGGVFRLRAGAWQDLEPPGPEQDTVGGPVVGFGGTARPATQATDAPATMSFGIATPGTGPAPGPAPVPAAAPFRFRASVARPGDVLLMCGGGLAEPLRGRPELAGHLAERWAQGEPPGLAAFLADTRTRVNGYADDRTAAAVWEA

>tr|D8THX1|OS=Volvox carteri f. nagariensis

MVGRGGVGPVRGPDLGLVSGLGAGPGADDGGSAPSAEELRRRCAEAALARFRSNGPTHDPQQQQQQQIQQQQHSQLLFLKDGPGQALQGTRSLVPLLPVSPGAPAPEARHACADSRDVVAGHHRSSDGGGGGKDGVCKRRGEEGRERGEQSAANKLECGTLSGKQGAEAEPPEANLHLSKPNTTIVASHSGPDTGVAVYKQFAADLPVTWDNPVDWAYGISVSMYDLGPTGRRNGEPVADCFGILAYPEGAILAVADGVNWGEPPRRAARCAVLGCLNHMHHALKQHETDSSLDCHTVFRHLLDSISASQKLILQQFGTLTTLPGGVVSVVLPLKWVRHPGRWAAITMTVGDSAAYVYRSSKRTVEEVTAAAHAEGARDPRWCPGALGYAVGEEPDLANMLLCLTFLEEGDFIFLTSDGVADNFDPVIRKMARHQVLESADNNDLPALTPAECQAKAMDLLRDVIVEADRSMSAGGGGSGSGGGGGGSDSSSGVTHGSVFGNHLNHHGGQGMTARGLAEHLLQHVYDVTEEQRQHVEARNRERERSLLSREGGAGGGGSGPSLNTDLKHGGLMGLAPMARPPGKMDHATVAAYQVGRRKPGAAARSQERMRRLDQERRDGQQHGLPFLAALAATAHPPARALGQPEAVEDDGGGGWGRRGWGRALFPPTPLYCRLC

>tr|D9UHI9|OS=Streptomyces sp. (strain SPB78)

MPDTVLEGGRFGGSTLRAVSQRGDSARYRGEARRDALLVKRFGAGEDGIVLVATATGARACPEAHRAATEAVRSIAAAVGRSHRRLADDIRAARRGDLKSGLHRLTDRTLGRLRAAAAERGLAPEEYTASLRCLLLPTDAQCRTRVFFGAGEGGLFRLRGGELRDIEPEVPTDGQPRSGPEAGALTLDPGIGRPPSPYAPVEEPRREPFRFHASIARSGDVLLLCGTGLAEPLRGSPPLASRLAEEWSAPEPPGLAAFLATSQTRVKGYADDRTLAAVWER

>tr|E0DEM2|OS=Corynebacterium matruchotii ATCC 14266

MNLFNFSRPSSSTSYPDIGTIAIPIIVIILLIIGVLLSLLGIIIKKNFSQKESNQAMDTDDRHDEWQSNNDDSVNKEECQSIRTDSEGPYGYTDEQEFTQSPTDTSSRSRKNLQYNSETNEVPSPSPLHRPEDRLSSITHSPRLNQNQDNSHHPTRKTVKKNRYVIIAEGLKRGIASFGDVKNAPNRDPLIVLSNKDDSYFPGCVADVIVHENLTIGIVAVRGLSHEENKKVRQDSVAFGVSANGRYLIGSIADGVSEAEFSHKGSYYATQKIVKSVRKKLDEGFHPEDLPWEDITNHVRNELRARGKRTFKIAEDAQDQELDSHLAKVIGTTAEVLIIDSKKINDQVSCIRASLAGDGYSFIISNDSMTLLGSGKETPSGDFVLNEKVCALPKDPGPKYPLINQVTISKNEAIFITSDGIGDDISKPDLGIDVYLCDKLAKPVPAYELIKITSYLAFQSHDDRSMIIVWA

>tr|E0DHK9|OS=Corynebacterium matruchotii ATCC 14266

MKLNYAIGSDQGLVRGNNEDSAYAGPHLLILADGMGGHAAGEVASQLMVEHVSQLDIDPGNDDMRSMLATAADEANRSIARRIKKSPETDGMGTTLTTLLFNGTEFGLCHVGDSRGYRMRDGNLERITTDDTYVQSLVDRGELDPEDVSTHPQRSMILKAYNGRVVEPTLKTLDARPGDRIMLCSDGLSDPVTDSTIETTLSSEGTPEGAVKQLIGLALRSGGPDNVTVIVADVVADDYADTPLPTEPLIAGAPNTGQELDPRPDTSSTRAAAVIAGSRAAKQAQQARQAAASLDDDTPTDPKIPEVPDPDDMDDLDDPGDLSNANGGKGAKAKGAAAAAGAASTHGDGDDDDEDAEDADDEYDAADEPATKKAKKKKKTPKKQTRKSRRGWIVGLVFIIIVAVLVTCFFFVDKYMKDRYFITVNANDKIVINQGMSSDILGGLWNTPYQEACLNANDNLTTIQVGAIESCHRFGLNDLKETARGSISSLPGGDYSEVLQQLHRLAAEALPACVIRKSETPTPAPPTPPTSSEAPRSNNTPGEEPPTSTEPVPTPTTSQQVVDDGNLNTPGVTCREVN

>tr|E0MUH5|OS=Corynebacterium accolens ATCC 49726

MTLKLNFFAKSDRGLIRGNNEDSGYAGAHLLILADGMGGHAAGEVASQLMVNHLEILDQDPGKEDTEALLAAAADDANEAISEHVTAHPETEGMGTTLSTMLFNGTTFGVCHVGDSRAYLLRDGKLEQITKDDTYVQSLVDKGELAAEDVSSHPQKSLILKAYTGRAVEPTLFTFEAKAGDRILLCSDGLSDPVTASTIET

>tr|E1QJN0|OS=Desulfarculus baarsii (strain ATCC 33931 / DSM 2075 / LMG 7858 / VKM B-1802 / 2st14)

MGSSPTKLTGAYYAGVGRGPFYEDDLFFAQVLSGGNTLAVAAVADGMGDGLGGLHAAQTAIGVVKSLLLARLYSLRGRRLSELELGELLKESLQKANAQVLKQSIQAPMGATLTVAVFTDEFIILGHVGDCRAYRLSDGQLERLTQDHAVGIALTRRLGKDPTMQIDLRVEAIAPGQIYILCSNGLHGQLGEDEITRALETTPTLADGCVRLACRALAKGGAEADSASIVGVEVGQYPRRPEIGSLAVDELALSLAEAEPVAEAARQEPPPNIRAPEPPPRFEAPPRDKPPARRRDRTQMAALLGLGLLATLALALLVLGVLSGLQETQGPSQPEAPASFAWPKLLVGLIALGLPVASLLWWRMGGGSKALRQAFERFRLK

>tr|E1VQA0|OS=gamma proteobacterium HdN1

MHQKESSAVLDRSTSQHTAVTHPGHVRSHNEDSLFGDDRSGLYLVADGMGGMDAGEVASALARDEIIRQTAHGASLANAVAGAHRVICGAAARGVGAVGMGTTVVALQLHGQAYEIAWVGDSRAYLWDAPAGRLSRLTKDHSYVEDLLASGAISVEEAHRHPQKNLITQCLGMSDDQVLDVGIQKGTLARGQTVLLCSDGLNDELTDVEIAQVLADSHLQNRAAELSSCADALLKKVLGGVAKDNISIVLVQVRSHFWPVWLRKRLSMARRGDLNRSGDLNRSGDSQQNQERNSANRSDSANRSALASVFFAMSLLVSAVFSVSSAQAEVLRKDLPSDEAFIVDGKVFSSKVFQAFSAAVRAKTPDMSDVAILKGVIENHLIARTGSTESSHEATAESTVWQEYTMLLDQLFPVKIDEAIAHRCIQLLPVKGEELIRWVGAEPTEKKRLVSGVISEEALKAAEKVEIAAIRCQESAPQSIRLDQVLRSADEAAVLKLRRGDTETFGRLTVIYARTRLHEGLLLKQQRLDDVDLSTLNQIVIDRERRVEMEEAAGVRIEMHHSPEQIRRLIKTITDTEIDAYYEKHQRDFQQIGEVSARHITVSSQKDADRVVDEIRKGLSFEEAVRKYSLASDKTENPPGSMGRISRTEKNLSFLQKLTLILPAGEVSNSFRMPDGKSWEILWVDSRTAVSLPKSDESVRSEIRRTLAQEKAQTQFADQVADLWQRAEVQLNQSLFPEPWLRSLPGAQKR

>tr|E1YHE1|OS=uncultured Desulfobacterium sp

MTSPTFYYIKEKIDLGKFKDRSLITFCISSYRNIAYMAVVESTGISDVGRKRKKNEDALYIDNSINLFVVADGMGGHNAGEVASAIVVDTIRDYMGRFIDNKIDAEELEDSDDSVSQEANRLVSGIHLANKGIYSLSKSNESYEAMGSTVSAVLFTKENLIAANVGDSPIYLIHNDSIELLSVPHTVLAEQMAIDPQNAKMFEHHFKHMLTQAVGIGETIKPDVCETPFFKGDILVLSSDGLSDNVTPDEILEIVRTEHPQKACRALVDLANERGGNDNITVIVLKVKDKKNQNGLLKGLISKIRSIFKVFFIFTNLY

>tr|E2MUR6|OS=Corynebacterium amycolatum SK46

MPERDYSDYHAGPASSSRLPQSAAENSGAPSNASNPAPSPKPKPTSATNRAFSTEITATSHDDGLAPLVVGDAGAEVDPIIVDTKFGTGRQVPDTVIDGWRSKQFAMRSVSLRGRMHRYNGAPRQDSVHTLLSDDGNTLFVAVADGVSAAPQSHRGSAIAARYAIGWLKKMYKDNLSQQEWISLAKGASYSIQTAAKEAGEDPMDFASTLVCAAISKCDNGLRGHVLSIGDSGSWTVTANGVELIEGGKEESDSAISSSAVNPLPLVPENLDAVYFETTGDETVLFGTDGIGDPLGGGGGPLSDLFVKRLYKRVPSITEFCHLVDFSKAGFDDDRTLVAIWPAHIGVPDS

>tr|E2NLM1|OS=Bacteroides cellulosilyticus DSM 14838

MQRSILKFQQNDYPMKRLKILIGLLFICLGTFSQTRVDSIRDRLFNPNDRSILVASHRGDWRNACENSLEAIENAIKMGVDIVEVDLARTKDGQLILMHDSKLDRTTTGKGLISEHTLAEIKNLRLRNGCHIKTIYKVPTLEEALLVAKGRVMLNLDKAFDYFDQVYELLEKTGTANVVIMKSNSPAEEVKRTYGKYLNKVIFMPKVNLDEDEALQKLNDYLRILNPVAIEFKFASDSNKLPYKVKDIMSGKSRIWYNTLWDTHAGGHDDDCSLVNPDNGYGYLIDHLGTTILQTDRPAYLIDYLKKRTVKKNMDCDRDWSYLTEENEYHLAESPNFVVEEYFLKGKKNPDSNEDGILVTPDFAAVIDGATSKSDFELDGKKTGRLAMELVLEAIRDFPKDIDAEEAMNRITNRIHSFYVKHNLLADLEEQPGKRFTANGVIYSYVRNEVWQVGDCQCIVGHLYSSNEKPIDAIMANTRSVVNEVALLNGMTMEDLEKKDPGRAFIYPFLQQQAVLQNNPKKGQLYSFPVFDGFPIQMEQVKIFPVGDVREIILSSDGYPHLFSTLRDSECYLMNILDNDPLCMRLYKSTKGIKKGNFSFDDRSYLKIRINR

>tr|E2PW10|OS=Streptomyces clavuligerus

MRFDGWIPSDERTTGSGPLFGAICLRRRTAVDNTRMSQQAEDDWWKRLYEESGPGAVPARTAGSTLDTHFDSAVHALAPPAPPPPRAPAPPEPSSTRAVPAAWDLPLLREDDDSGPPRPSGGRAVPRKAGASPGPVPVVPEADRGVPVAVSPPGRGECGPPPFDGPGPGPVPTVPEAGPVAQVAVPAVPLSVSVPGREVVGAGASGGRGPGPVSSGPEVDPGVPVAVSPPGRGECGPPPFDGPGPGPVPTVPEAGPVPPVAARAVSPAVPVPDAVPGPGAALAEVPTALADGPVPAPRGPGGGQRDGGGAPPLMPLARAPWEPPDDPPLRHAPERPAVPLGPAAPRTGPDSPAPARAAAHPGHVGDGPPTYEPEPTALPAATAENLARITPDTALDGARCGSGVLRAASIRGDSARYRGEPRRDALLTVRFGRGPTALVLVATASGARAAEGSHLAAADLCRWIGGAVGRSHARLAEDIRADRRGDLRAGLHRLTDRGFGRLRSTAAESGAESGEHPAGLRCLLIPADPECRTRVFFGAGPGGLFRLRDGAWQDLEPPHPAAGEPAGEQPEPPGPPFRFLAPVARSGDTLLLCGPGFADPLRGEPALAAELAARWATPAGPPGLAAFLTDVGLRVKGYADDRTAAALWES

>tr|E3J3D9|OS=Pseudofrankia inefficax (strain DSM 45817 / CECT 9037 / DDB 130130 / EuI1c)

MTARSTARRPADSAPKGPAVRVAGDAVRTALLLGPDHPGLGEVALGGLSVEGDGAPTMLGAALSAGWRPKAVPAVEPNEDGALLAVGPAGALLAVADGHNGSAASTAALRALAARAPVLLAHRTGDEAELAAAFAAAAHGAVARPPAPGGSEAGAEGEPRPARTALSLVVVTAAAFAAVGYGDTTAAVVRRGRLHLLDEPSEFLGPTSPADPLAGHPVTRRRRGPGDLLVVVSDGVTDYLGRAFGAAIEAASRAGADGGQPDAARATARSLIARAGAAGAGDNLTAAVLLAARPRRTFGWF

>tr|E5B8V4|OS=Erwinia amylovora ATCC BAA-2158

MNITLASMSNQGARTSNQDQVGEIVGDRSACFVVCDGVAGLPGGDIAASVARDTLLQRFNGQQHLNAQLIRQYVNDANSAIRQRQKADPPHHRMGTTLVSLFIDRDYQLAYWAHAGDSRLYLFRRGYLYHVTTDHSLVQQMKDAGHQTDGINGNLLYFALGMGDEDRDASYSDVVPIEDGDAFLLCTDGFWHGVSQHHMQQSLHMVNTPQEWLTLMQQMIKKNEQQSDDKQDNYSAVAVWVGEPQDTTLLHSLSEAAQFISLRD

>tr|E6QU81|OS=mine drainage metagenome

MRFSIYQSSRQGGRLNNEDRMAYSYSRDALIMIIADGMGGHAHGEIAAQVAVKSIAEAFQRHAHPKLRNPSGFLYGALQQAHKAINECVDKQNLPSPPHTTCVACVIQDDTAWWAHVGDSRLYLFSDNTLVTRTRDHSAIQLMLDEHLISESEINTHPDRNKIYSSLGGSIAPEITISRPMPLMPQDHILMCTDGLWTQVTDEQIATTLFRLPPDNAVEFLLNHAERSAGPTGDNLSAIAMVWESQDETDENAITTVAMSNTTFTTQLGNYMPDDADLDEDEVERVISEIQHTIKKLSETKP

>tr|E6WEU7|OS=Pantoea sp. (strain At-9b)

MNITIASTSNQGDRASNQDQTGEIIGERSACFVVCDGVAGFPGGDVAATIARSSLMETFNGETHLNAQSIRDYVNHANHAIRQQQKASSEHSRMGTTLVSLFIDRDYALAYWAHAGDSRLYLFRRGYLYHVTTDHSLVQQMKDAGHQTEGINTNLLYFALGMGDEQRDASYSDVVAIEDGDAFLLCTDGFWHGVTLEQMQQSLHMVNTPQEWLTLMQQIIKNDQPEQGQQDNFSALAVWVGSPQDTTLLHSLSDAAQFFPLRD

>tr|E8R6P3|OS=Isosphaera pallida (strain ATCC 43644 / DSM 9630 / IS1B)

MNPGNLRYTEEWIHWVGTEQFSSPFQLFDNERSHWSRFLQPAPFNPPRSPGDGLAWIERASAWDPIRRERFLKSDPTALVEGRCRVVIPSTSRKAIEVDLARRIRRMFAWLFPNRNDSDSPVATKAPPGWKPVVRAGVRSDVGPVREHNEDSYYVPGHAPVSRYNSLGSNANVDPTADMTPIKGPENLFVVADGMGGQLAGEMASQLAVETIPSVVKSGLTEDLSPKETRELIRRAMSRANEEILERASKGPETTNMGTTCVVALIHGDRAYVAGIGDSRVYRLRQGKLEQLTKDHSLAQALVDAGTLKPEEVATHKFNHVLYLYLGSREARDGPDTVKELELMPGDRFLLASDGLTGVVPDEDLARVMAQNQDPEVSARELVDMALRNQSRDNITCAVIHID

>tr|E9SH05|OS=Ruminococcus albus 8

MKYYVYGYTNTGNRDKNEDAVLVGNEVVNDGFREAVLDAPFITGICDGVGGEKAGEVASRTCLQMLSEVEYTASSDLKSVVLDIHNKIKKVGSRSRSAENMQTTLCALAVDEEGRGLCLNVGDSRLYRFVNGTIRQISTDQSLGQLMYNKGAIDTAEELVPEYRAAIVSAIGSPHSEPNVEQVPLVSDFGAEPDDMVIITSDGISDYVSDNEFEVGLSMDLPISEKLAALAKLALLNGSNDNLSIIGIKPFLDFEELAALTSHDAVSETVNVMEMLEEQSAPRTAEEKDELSDILTIDLGDIIGKSKPEPKPEPHHEIVSREDIELETHDLFMQAQASLSRLSGMIPKKKKDD

>tr|F0RK60|OS=Deinococcus proteolyticus (strain ATCC 35074 / DSM 20540 / JCM 6276 / NBRC 101906 / NCIMB 13154 / VKM Ac-1939 / CCM 2703 / MRP)

MTDIGVQRRGSVNQDAVMAAEVPGAQLFAVADGMGGHAAGELAAQLALEAFARVLGRQRGPLPQRMIYAAESANQAVYRRAVGELSGMGTTLLAAVVSRGALYLAHVGDSRAYLMRGDALFRLTDDHSWVADQLRAGELTPEQARTHRWRNVVSNALGGEDQVRLELLNIPLEAGDRLLLCTDGLYGPVSEQKLLTVLSLPRTPQQLTRTLIDLANQAGGPDNISVAVVDVLNPGGPPPRPLVRRREGPVYAEQLLREAQQGSPLTYLLLGAVYLVLLTMALLPMYRLHTALVGAGLLGLLLLYRRHWQSPHEAVLRPEARVTLAQPGHDPWTETGRLNT

>tr|F1YG58|OS=Gordonia neofelifaecis NRRL B-59395

MDEPKILRDEALTGRDEVGGVVVDWSVVCHVGRVREANEDAALAVPGRYIVADGMGGHDSGELASEAALLTLAEAPVGESFADTRRDIGELLEQAQDRIGEFGTETGRRAGTTATGVVLALDDGVPNWVAFNIGDSRTYLYSAGELRQVSVDHSQVQELVDAGYLTPEQARIDPRRNVITRALGAGMAVPVADYFAFEAADGDVVLLCSDGLDGELNDDEIAVILEASPDNEVAAAALVEAALESGAHDNVTVAVLSVSLPAEPGAVDETASQ

>tr|F2L5X1|OS=Thermoproteus uzoniensis (strain 768-20)

MRLMISLAGKTVYGELSDIPEDAGGCDSLNLAKLGRKRDYRLAGYLRSIRGPRELNEDSAAVAVKEEEGRGLVAVADGVGGLSYGDAASSTAICAVIANFAKADHYDESWLADLFSKAHEAVSRLGKGATTLSVALVDVNSQTVVAGNVGDSPMYLVDFEKGTVTDLTPNRDERARYIVQAVGHKSYVGPHILKIPIGARGALLAVTDGIDDFLPDKRLYAAYLRGPDRHVNELIKAVENKTKDNATAVALYFKTSRSSLWRLFRI

>tr|F5RK09|OS=Centipeda periodontii DSM 2778

MDTQETQKEPVAAQTKPWTDLSDAVKTETAEPLADIPAVQETAEEDAAAEMSAAETAAAPPPQEVATPPETEEHVAEVPTDGSTAAESAVAETAAEPPGAEEAAPLSDEEVLAHTAALWEYHPVPADEPDAAPEYRSAAMQIGAAEMIAARVRGKKHKHDGSNCDDWFAAVHAEDIAILAVSDGAGSKRLSRVGARAASEAAVGAVYAGLQRLLTAHAELRTACALPMEEAAFGDACAQLAGILHEGVCAARVAVESAFYERCGRAAYRDLLGRDPVLADFAATLLVTLAVPVPERAELLVLSCQIGDGMTAAVNTHAAYGSAVKLLGRPDSGDYVGETDFLTSDGMTDSAALQNRTLVSRGAYDLLLTMTDGVADDYFNEQELHRLYLDLALNGILPAASEQTMFTRAEACLLRSLPKPTAFPWVNDKTEQVALQSAQRCCTSAGCTVETLWQSAHVLPLAAADTTLARVSAPAQRLEIWLDNYMARGSFDDRTLVLLSMGAAHV

>tr|F6BBS2|OS=Methanotorris igneus (strain DSM 5666 / JCM 11834 / Kol 5)

MEIIFFLINYFLNGENMDFKKIINKGLKKIFEFVESKEEGNHKIPTKLDIEEKNNIDNKYNIEIPEKPQNIEIPKIEPKLSEIKSTENINRNEIIFEENNAYGVSHKGNRENNEDYILIKKIKDIYILAVADGIGGHNAGDVASKMAVDILEEVITKNYNENLSIEEIKKLLKDAYNTAHNKIKENAIGDKEGMGTTLTTAIVKGDKCIIANCGDSRAYLIRDGEIVFRTKDHSLVQALIDEGHISEKEAMHHPMKNIITSALGLDEFKVDDYEWDLIDGDVLLMSSDGLHDYVEKETILKTVSSNNNSKDIVNELLKIALEKTKDNVSIIVYKKLTLKN

>tr|F8KTM2|OS=Helicobacter bizzozeronii (strain CIII-1)

MPGEIAVIALADGADLARFSHLGAQKTIEVVARDLRENFTHYFNMARSEEASSAILERVIQALQELSVETANTLQHNKSDVESILQAILEEVRAVQDWQETWHLPLLDTTQTLQKRLQTDQEQRHNAQEPILNALTGIKTEIGALQEAFQGKDYQLEFSALENTLKDLKSKVAEMHCTLQSPQESPILESKYQAIVLGIGIEVTKIKNGGWFKRLWRWLFGSKAQKQAQHKLQALKDALASSASLQFKPWTHTLPKNLKDFHLEDVRTEMKAHQDILEWNVSQCFKDFRLLLKEIGDVSFENWDEKHLKSLFQVMATTRTKHQENREQLKAQIKQANTHLHIYQQQLLDEIRIKEQECVSLMTRFVEIKHGTLHLEENLQCALNKLAEKVQTLNAPYTLQNLRDVLLALQKENLQQYSKLYEDYATQSAQVKNAFENLKASLPHANASQQPCSQDILNAPKHTALLDYINTLETQTRDFQSMQERLEQCQTIHQEAVALEKVLRQDLAALVMGYATLQPSIYTLQAQSLYHIQDLDSLDVAFVQSCLQRLEQHLRTEQELLERLQRELQESSSPAPHFNFTLPTLLQRLQQAIQNKACDLHDFASTLLVTAIDGDQFLLLHLGDGVCGVLKDRQLVVANYPENGKFDNEAIFTTSKNAPLSAKVFKGKLSDKNFTGFVLMSKGASEFFYHDKEDALVTALQDYMNVARAPGMYHGVQDSLKALLETRVREKTSDDCSVVMLVKQSMEPLSESEQRLKTQMETISNDG

>tr|F9ZGD7|OS=Nitrosomonas sp. AL212

MNYEKFQFAAFSQRGRGKSHNEDALLLDGHVYQGSVREQGVVDAAQTCYFAIADGVAIGTRPRIASRCLLEILHKHLAVSTATESLSPLLERVQQDYGAMSGNPRFYGMAATLVGVRLLENAVTIFNVGDSRAYLLVDGQAQLLSRDHTLLNDLIDDGEMVAGQAADSASIVHGLTCQFIADPECDDFRVILPRINCSLENAFCCVATD

>tr|G0ABM5|OS=Collimonas fungivorans (strain Ter331)

MQFNLPRRMSQYKIEAGTGQHIGDRQEQQDRAALFTAPKAPGYMMAVLADGMGGRSGGALAAEQVICTAKQIFEGFSPLIDNVETMLHNIALETHTVIKLSGFSNDKDPHTTMVILVLSPDGTATWGHVGDSRLYRFDGPNFAEHTIDHSYVEKLVSEGKLSRSEAKSHNLSNILLNALGSSTDTPQVSISRHTGLKAGDAFLLCSDGLWHYFTEDELGAAIAMNKPRDAAEMLITKTRKRSEGHAADNCTFAIVKLVALPKEAQNYTVKKMRRAV

>tr|G2DWQ1|OS=Thiorhodococcus drewsii AZ1

MGVIGRCISALLPDFKTEAVSLTGLRHENQDNYLIIAQGSDGQPKARWLRDASAAEETRASWPSRWIRLAVLDGMGGHTHGREIAEAAVDALRRLPPCQSTFKQRRAVVDLHRNLLRQFGTGQANSPGTTLVWAEIDRLRRRCHLLHLGDSRAWLGMNDRWHPLTHDHTLAEFGYRDGRIDPSAYAAESERRNQPLAQALGHGTWGVIMDAEGHESFGFAPEIRLDAMHDLSPSLQGHADLRTIDLPRGTPLILATDGLWSGIGCDLPPPTELMVPGAIGDLAQTAIDAGSTDNTTLIIGHFDPEPAS

>tr|G2E7Y1|OS=Thiorhodococcus drewsii AZ1

MGAKDRPLTAEQRRQRDASLEDLIWGLFGQNNVPFFDKSSFSPTLVEEFVRSEEALEAAEQFATQLLQLWRLRYPGKSLRLRPRGNLSDTVERHVLPDFSDKSAPERPNKGQDAKPVPNADAEGKSEMPTTDQGHSPVPATADSSASSQSDSAPGPSPDTKPESRPGDIPSTGTPDPRPPDPRASFSLPNAKVGVAYAETPQGRTPEGIQVRLVDVRLPEGLGLGFDPDRGEVSGTPAHEGDYRIPLRWTLDGKETYSGDCLLIVNPDPQSLWKVLEPPADAPYRKDHIDSRLIQGDDYRILAASRRGRSHEHAGSFRDDDFHIARDPDSGWDLILVADGAGSAPFSREGSRLAVSAAGDHLSASLAGEMGTRVDEALAGWAEDPQETARAIGTEFHYLFHKAAQLAVQSIEHEAGTRGAAARDYATTLLAAAIRRQGSETFLATFWMGDGAIAAYGPRGRVRLMGTPDGGEFAGQTRFLDRASISDQGFAKRIGIGRFADLNAVLLMTDGVSDPRFETDNGLSDPTRWDALWDDLVPLLELPDPEQRIADWLGFFSPGHHDDRTIALLW

>tr|G2GKY9|OS=Streptomyces zinciresistens K42

PGGPWSAHDAPADPPASPATGVRFDRSPQPEEYPLQAPDPRVASEPPTPPEGTRMPEAARMCVACRAGRVDHDGYCENCGHAQPRERDHMEQEAGPAAAVSDRGLRHHRNEDAFAVACATLPDGRPAVTAVVCDGVSSATRPDDASAAASRAASQALLAALPRGTHPQQAMHEAIVAASEAVNALAAEPATARGQGPHQNAPACTFVGSVVTAELLVVGWVGDSRVYWVPADRGTPAARLTEDDSWAAQMVAAGLMNEAEAYADERAHAITGWLGADAYELEPHTASFKPDRPGVVIVCTDGLWNYAEAAGHMAEVLPLDAGARPLHAARVLLGHALDGGGHDNVTVAVLPFPAPPAGAGSA

>tr|G2GPV2|OS=Streptomyces zinciresistens K42

DSARYRGEPRRDCLLTARFGSGEHTLVLVAMATGARATPGAHRAAAEACRWIGRAVGRSHRRLAEDIRAARRGDLKSGLHRLTDRSLGRLRASAAEQGVEPGEYAASLRCLLLPADPQCRTRVFFGVGAGGLFRLRDGEWQDIEPLGTDAASEPVVGFGSLPAETPEGDRLTMDLGIPTPPSPYEPEPGPPREPFRFRASVARPGDTLLMCTGGLADPLRGEPALAAHLTGRWSGSGAPGLAEFLADAQVRVKGYADDRTAAAVWEA

>tr|G2NYP2|OS=Streptomyces violaceusniger (strain Tu 4113)

MSQQGDRRRHEDDWWGELYDPQRADAGPAAAPDSVDDRFDSASRTLAGAEDAGNQRPAAGAPWGPRTGEPGDAAPGPDADTGGGPQGWRSPTDEEPGAASPFRGEAGPGPARGDAARSGATPPSRGGAGSGAATQGAGSGAAPGRTGSGAAWGGAGSGSAQGDEGPGAVSPSQGGAGSGAGRGGDVGSGAAPPSQSGTGSGATQGGDAGSGAAPGRTGPGAAWGGAGSGAALPSQGRAGAGDPQGDAGSGGAPGRTGSGDAPGRSGPGGAPGRTGSGDAPGRSGPGSAPGRPGAPVPPPPPRGARASVPRRPDLHPDPSRPWRAAAASYVGDEPPTYEAEPTTLPVVDSEELRDLVPDTVLEGARYGTLTLRAASLRGDSARYRGESRRDALLAVRFGIGDSALVLVAMASGQPAVPGAHRVARELCEWIAAAVGRNQARLTEDIHTANRGALSSGLHRLTDRAYGRLRAGATVRGLAPADHTASVRCLLLPAHPACRTRVFFGVGDGGLFRLRDGVWQDLEPAGGERDTVGGPVLGYGGGRPPAQQPPQPPLPPQPPYAQADPSSAEPSPSPDPGPDQDPAPAHGPFRFRASVARPGDTLLLCSAGLAEPLRGEAALADRLAERWDTAEAPGLAAFLADTQTGVKGYADDRTAAAVWEA

>tr|G4T462|OS=Methylotuvimicrobium alcaliphilum (strain DSM 19304 / NCIMB 14124 / VKM B-2133 / 20Z)

MNRDSIGNGQTLGERSEQQDYFATFDSQDAKVFIVADGMGGYSGGAIASEQVTKAFVRSFKDRTNASMPSVLTAALQSAHAHLKQLCEQSSAGEADMGTTLVALALVEGRAFWLSVGDSPLYRLSQGHWQRLNANHAFAEVLLEDVECGRMSREEALQHPEYHTITSAVTSEEITFIDCPNEGLPVHPGDRFLLASDGIHTLTDPEIHALMKEGHDAQQTADCLLSAVAQARLPGQDNTTVLTVFCEGEHSKSVKQRRCRPVFLIALAVAGLIAEAYGWLDKTLWPTQPAGSAMPFDNEPDHRIAAAKEDESTESNVLRTDDVVIPSSSKALDPSLQHEPDQND

>tr|G5H4X2|OS=Selenomonas noxia F0398

MIEVSSASDIGHVRTSNEDSYGVFSPSVYVVADGLGGHAAGEVASRMVVAAVRDMANAAEAIDAATLQSAVLRANQQVLEASEENPSYEGMGSTATVLHIDEQARTAHYAHVGDSRLYLLRRGVFRQVSRDHSYVEELVSRGELSEAEAQHHPRKNLLLRAVGIEERLHVDGDSFSLENGDRLLLATDGLTNMVSDEDLSVLLGDNRFAGVADRMVERALAEGGKDNITAIALAYEAS

>tr|G6FTP0|OS=Fischerella thermalis JSC-11

MISTQRIINCPNPICTHPTNPVGNRVCANCQTPLIHRYLWVIGSSAGTILQGEKVADRYEVIAPRIWLDTQPGKLPDIPGTIPKEIIPYLRLHQQRLHLPQVYGFVRSQTEAADDILLLENVPIDEAGNLYSALTKAWQQATAVRQVYWLWQILQLWQPLSELGVATSLLIPNNLRVQGWCVRLLQLQQSGQPSIKHLGECWQPLVVTAKSQVARDLQKIVQQMCSGEAELKDIAAQLNGLLLASAAELPLSIKVAGATDKGPEALIQNEDTCYPHNNNAIADSLLPRVAIVCDGIGGHEGGEVASQLAVQSVKLQIRALLQEVTEQAEIVPPDLLQQQLEASLRVINNIICNCNDEQKRTGTQRMATTIVMAAQIPQRIQTTAGWQSDNAHELYLVNVGDSRAYWITRNYCQLLTVDDDVATREVCHARSLYRQALQRPDATALTQALGTKHGELLLKQALFNNRIAVLATKHQKERVIAPILEAELRMKVVVPEDFDTDVFGTFTREVKRPGNQVEAARLKAKKALELTGESLAIASEGSFGPHPEIPFISSNREVVLLLDQIHNLEIVGEELSANTNHNHLVVESVEQAFQFAQKVGFPEHGLVVMFDELPNDKTEVIKGITSEEKLIEAVNFVLKNSPTGKAHLETDMRAMHNPTRMKNIEKATRDLLRKINSCCPECSMPGFTITSRIRGLPCALCYMPTSLTRAVIYQCQKCGFTQEELFPDGSEYAEPVNCNYCNP

>tr|G7EK54|OS=Pseudoalteromonas sp. BSi20652

MSKVKLEQALLDILLQASLTNTKQSFVQKRANQHLDQTVVQHHINALLNELTPLVKNDKSMVRLTTEGAKLHKKNNNLSVNKQIPPLVINVRLPNLKANTLFFESISLASETDAKISFFQTKSIDEFEGVEFDADKLTLSGTTTQVGDHQFDLYGSTPLANGQVQGVVVRVKVTIVPDPKSLWKNIPSDDTARFHKPDTHHAYCENSSMLLMGSSVRGRSHAHKGIHRDDDFKLYCDDSSEWIISCVADGAGSCKYSRQGAFVAVNNATDSLKESLNGHYGTALEKAYNDFSNDSNEENQKSLLEAYQHTIVKAVFSAAKAINNSVDANQGDSIKDFSTTLILAAHKKVAGGYLVISFWIGDGGIAIYDKNKK

>tr|G7GL67|OS=Gordonia amarae NBRC 15530

MDLIIRDDSRCGTDTVGPLTLSWAANSDVGRVRESNEDAASVRPGKYFVADGMGGHESGEVASETALRAFDGVVCGGDRTETQTSLIDLLTTAQQQIDEIDPGAHRRAGTTATGVVLVTHDDRPHWLVLNIGDSRTYRYAQGVLEQVTVDHSQVQEFIDAGFITAEQARTDPRRNVITRALGAGMIAPEADFYSFPAVAGDVLLMCTDGLTGELPDGEIEEILAEMQSPKDAVAALVAGAHALGAHDNVTVVMVAVSDAVAEDADGDATVPAESDGTGPAESAADTTDPAAPDTAAPDTAEPVAAAPDAAVPDAAVPDTAVPTTGDPADAAPETAAPDAAAEDASV

>tr|G7GYA1|OS=Gordonia araii NBRC 100433

MGRRAQPTPAPGAEAGPAEADPTGDPEAPPPVAPPASGIDPLSPVDAGVIDVPPVVKPPDDVGPYPAIAPEPPVAEREWVLRELAEEHVVATLEGGFYAGRPIASSGPDLEGLDQTRPTAVGHARGGHRSDVAADMGWINDSLAVGAVSLRGIGHHAKARLAVRQDAYSLGHTDDWVIIAVADGVSAAPWSHLAAQHAVIRASTLVRALLDDVGDLPTPGQWATVAKEVRASVEAFARRSVPAPAPGRDAPSVETLALALSTTLDIALVATAPDADGRYRYVHASMAGDGTSYLLDPVLGWSTIRDGQPDQKDAPADFGVVPLPRDPGRPPVINAGQLEPGQAFMLVTDGFSGMIAGGAGVAGVYLHDEWGHGPVDVARLIQAASLMNPSGDDDRTAVIVWTC

>tr|G7ZGE2|OS=Azospirillum lipoferum (strain 4B)

MMLHSAKCSHQGSRSIQQDAHAIFHDQQASALLAVLADGMGGHEGGQLASRIIVEAAQTQWSEGVPEDARSFFLSICAKAQKDILRLGQESGISPRSTVVLLLIRGTQADWCHIGDSRLYHFHDQQLLHRTRDHSVVQMLFDLEEINEEDMLHHLDRNRLNRSLGGDEAPNPTFGSAMMGPGDGFILCSDGFWEWVTPKEMAGVLWEKGLDRSLQRWVDEAVVRGGTAADNVTAIVVRWPSQREDQLTGPLRVVLPGLPHATKIAVQKHTLHVTLAIIVALLIGFIGGFLLGKLIITNEPPANQADHGAPAARQGNNNNTAVSQSGVPPTSGAQTTGTGQREVPSVPGANTTPEAQGTR

>tr|G9ZHU3|OS=Cardiobacterium valvarum F0432

MTPKTLKEWLHHADDEILARFIANHQNLIAQLMQAWQRQLNKEQAAHNLPDDAAIANNPSPLADNPDDMADDATVPRPHEEEDDKQTASETTATACTAQETPQTDDEFAPLEPSIPDTAMPLHPATPTPTAAETPAIHLPPLPNANQGQPYAHTLPADLPITGLTIETADSGLTWDDDTHRIYGTPTVSGDLTIRLTLLDNGKEIGTHLNLHINPDPKSLWQNLPSDQDAPFAKPDSAHEEQTTPHGRLIAARQRGRSHAHIGSYCDDDYRLHYHEASGLHLLTVADGAGSAAHSRYGSQLAVRP

>tr|H1LZ14|OS=Lachnospiraceae bacterium oral taxon 082 str. F0431

MSYKTFDSYSKGYSHIKNGTDCEDFASSYADPEGRYYISVICDGHSDNNCFRSSKGARFGCESAVETLTRFFDLYYEDNATIDLITPEVELRLKRSLKQCWDDKVLADIRSNPITEAEKEPLSERVRSIYEAGRGLLNIYGATLLAVAVCGDAFIALHIGDGVMLCVDQDGTYYEPLPDDEKSETGSPASLCDSDLFSRNNAFRSLVSPTIPIAVVVSSDGIGDCLDQLDFMEFTYNLITKLKSMESETVFISELSNNQKEYLDSCTAYYSAKGNGVEDDCSISGIYSNEDNVPEVKVPLSDAEKMWSAMTAERTGFIEDYEKRKSDVISNINAELNDSLRLGLNITDSLRLGLNIIESRKLPMLDSWIEKKKKVGELKKVLYNIDHNENNKILYYDQKLEQYAKYIRRAMVL

>tr|H1LZE7|OS=Lachnospiraceae bacterium oral taxon 082 str. F0431

MSNNAKLDYNKRQSEQKVFDGIKEEHLKIIQEQIEDAKDPSSYETYDSRVSSADGRIKLFTARRKGISHITSNTPCQDYCLATSVNGCTVLADADGVSSCERSDIGSKLACEAVVLAVKAAAKSSNGEDQLVNRLLSVSFRDRLVSIWIKCVMEEIAKESILLPEDQLKEFSKYGSTIMYAVITENWIVVGNLGDGQIMVFNDCYGVKLRVHAPKDSSRVRCLANERCVREDFSVAKYPRSCFNGVLLCSDGIYESLDKSNHFYDYCIQMKKRFLERSPNEPYQAFCYKEEGEPYKDFSRMRTQDDCSIALAIDAQDITSDYEEIMTSLLQHSQVAILKRWSCECMSFYTKLKTSYADVVVSAKGSDIELTKLNSAILDMPSQTWNGGDFCFNEYPDAAVKTIEFMHCSGMLRRDKTNPEESEQMILSVYLNLVKLRRELHELGLRFNSSALFNVLYDGKNLHLRKEALSRTANGTQNSNFDDIEVCFSHLLGIFESGDSRIPVFDIGYIDRGIKQYRIGHSSDELAQLQRINKKLHIKNVGLYSWKFDDDIVLQPGEFRELGKNMKFTLLDSNGQKLETYKYVSKELL

>tr|H5UVV6|OS=Mobilicoccus pelagius NBRC 104925

MTSPSSPPPADDRDPVTSEAPTEELRPCPSCGRPTGAGRACENCAAPAGASDVERAPLAEAGETPVPVGVSSDTTRPDVTRSGPMATDPATSDPATTDPATTGPAGAVTSDVAAPASSNTGSTAVTAETFAARTTPDPVPEAGAPPAGGSDDEGETAPPTGSETPPAPLPPVGSPTPAAAGTSGPGPSEGAATADATSVDVAPGSAPRSAGMLVLGGILPEEPVAAPAPAPEEGSRCACGGTFADGYCEQCGSPVPAPRAHIEDAPAPWVSGVCDIGVRHRSNQDAMALFADGARAALVVCDGVSSAMRSEDASQAAADAAVAVLSRATSTGVGVPSSVVPALLTRLDTAAEAAADAVADVTGQIHCELGIDSTREGDTFHANPSCTFVAAIVEAGHVVVGSIGDSRAYWFPDAGEAQRLTVDDSWAEEQIALGATREQAESGPGSHTITRWLGIDCDDHTPRTATLRIDAPGWLVLCSDGLWNYASEPTALAEVLGEIAHGIAGTEAVTSPVVAPLADPHTGAPVDPVPPAATAPDPTVPTAPAATGTDASATAPEAADQDGTTVPLALARGLVQWANARGGHDNITVIAARLTPDAPTQEA

>tr|I0GP29|OS=Selenomonas ruminantium subsp. lactilytica (strain NBRC 103574 / TAM6421)

MGIYSLIGLSVIIVLLLVVRFWPEKQTVVETVPEKAAALPSNPIGTAATIGQRQLQQDLTGSALGDEGGLLLLADGRGQAGKIAAKLAIDTCLDLYQECQGQDKPQYYFRKAFQAANHKILSVLEDGRGSTCLAAAIIQRGLLYYALVGNSRIALFRDGDLVPVTEGQTIDILAQHRYQQGRITKEQALKLLHERRLYNFVGQDGFHDIEFFSEPIELQPGDIVVILSDGVTETAPWRQIEDCLGQAASPQEKAQQIIALVEGSSREDKDNASVILYDTYDPAQMPVEKKQGRKKKRSNHLEKIKQRLSDSLCL

>tr|I3Y946|OS=Thiocystis violascens (strain ATCC 17096 / DSM 198 / 6111)

MRTHQGSVRSDNQDRVLMARWQDGCQTAWVLAVADGIGGGKEGGAAAATALSAFTADVLELSEVSLPKQLERASLRANQTVYERWRGKEGSTLSAVGVRGEQRAWVNVGDSRIYGLNAHGDVSQLTVDDSLPLGRGLVQFIGIGQGLVPHVGLISPTHHLALITTDGVHQYVDPLLSALVRTAQRNSSALVDRMVHLALWCGGEDNATTAVGLLDSSQEGSQAACDVWTPGQHHRLVLGCLDAPNATASVRGCRQK

>tr|I3YCV6|OS=Thiocystis violascens (strain ATCC 17096 / DSM 198 / 6111)

MLDAAGADGTMPSAARKPTPEQRQKLAPEIENLIWGLFGPNDRPFFDRSEIAPVALDEFVQCDEVFEETFRFLDALTGIWNQRRPGRPLKPFTQDRQPKEIAMPMVLPVLSETPVSRREESKAGDESAPTEANIEETSAMPFPHPPDTPTPGSAQGSPGQVPAASTAPAKPPEPRASFQLPNGKVGVDYAARIEGRDAEGRPVRIRDVRIPDGLGLSFDPDSGELRGTPALDGDHRLPLSWYLDEKTRYSGECLLIVNPDPKSLWKVIEPPDGSPYLKPHADRRLLTGRGFSIAAASRRGRSHEHAGTFRDDDFFIGHDAQTQWSLILVADGAGSARYSREGARLAVTSAGAHLSEALAGEIGARMTAALDGWEADPEGVAQAMGAEFHYLFHKAGTLAVQSIEQEARSAGTEVRDYATTLLAAAVKRRDSETFLATFWIGDGAIAAYGPRGRVRLMGTPDGGEFAGQTRFLDHVALADQAFAKRIGIGRYADLSAVMLMTDGVSDPRFETDNGLNDPAKWDGLWGDIAPLMASPEPDRNLVDWLHFFTPGHHDDRTLALLW

>tr|J1GYS6|OS=Actinomyces massiliensis F0489

MSEILATTRPQGDALVVPVVGAATDVGRLRTVNEDGYLAMAPAFVVVDGMGGHAAGRAATRAALSALAPLAGADITGEQDIVEAVLAAGEAVAAIPSHAAHRPGATVAGAVLARRPESLTWIIFNIGDSRVYLLRDAVLTQMSRDHSQVQALIDAGQLTPAQARRDPRKNVVTRALGAGLDQEAVPDIRTVAAQCGDRLLICSDGLSDELDDEALATVLDAGMRPQHTAEAAVAAALEAGGHDNTTALVVDLVPESAL

>tr|J3F2F0|OS=Actinomyces naeslundii (strain ATCC 12104 / DSM 43013 / CCUG 2238 / JCM 8349 / NCTC 10301 / Howell 279)

MTISLRFAARSDVGLVRQSNQDSGYAGPHLCLLCDGMGGPAGGDIASAVAIEHLMPLDADSHQAGELLGLMRDAVQAAHTELVTLSSQDPDLAGLGTTCIGVMRSGNKLAMVHVGDSRAYMLRDGTLTQVTTDHTFVEYLVETGRLTRDQARQHPQRSVLLRVLGDTEGEVQLDESIREAVPGDRWLLCSDGLSGPVTAETIGEVLAGVADPGQAADQLVDLALRAGGPDNVTAVVFDVVKDDPEPQTVPQVVGSAATERLAQERAAAANRKADGEAGTKDSEEASPAAKAAALMATLEERPDSTSKKDSSDVDEAIAVEAATQQKARRRHRRRVLVGSIVLLATLVGASALFYRWTQTRYYVSTYDGQVAIYQGIPQSIGPLKLSHSVKTYADLPVEKLDNNIRERLEATVTQKSMSEAQTYVDKTVRSYLKPARSPQGSSDSTATPSPSASPKPTSTPSSKTPAQPAQPDQAEQRAEMTAAPGPMGAPAGVATIQPAGTARHSGRWAEAALLGVALVLGLGGFVLTALNRTGSSPAQTVMLGGAFLGLTVLMHLWVRYTAPWADPVLLPAAVALNGIGLAMIQRLDLAYEVNEQWQFYVGAKQLIWTLLGVILFCAVLFLLRDYRRLRRWDRWAMWSGLVFLVLPFLPFIGQSINGARIWIRIGPMSFQPAELSKVLLAVFFASYLVANRDNLALAGRKVLWMSLPRARHLGPLFIVWGVSICVLVLQKDLGSSVLLFGLFVVVLYVATDRPSWLLIGAALFLPAAWFAATHLHHVQQRINGWLHATDNAVYNAAGGSWQLLTGMFGMSTGGLMGAGWGKGSPTLVTFANSDFIFASLGEELGLTGTLAILMLYLVLIQRGLRTAVSLRDGFGKLLAVGLSFAIALQIFVVIGGVTRLIPLTGLTLPFLAYGGSSLLANWVILALLLRLSDAARRPATHAPRIIDTAELPVSLRRRVQDAGADEPAEETVGSSPSQAASPASASYVDDQRAGQPEAGGYGPPPRRPATRRTTRPPRSSSARRHRLRRTPHRTERHCAHRGPRVSVTTATGGSSHEPTDPAGRLPGPGHVLRSGPVRHQRPGPGSPRHLGTTVRQRSPHLRRTQLAHRQPGVRDRPRPDPAGRWHHHRQHPEVRRRPGLPELPARLRQWPALRPGDRLLLARLRLDDRPGAGGELRAQRGRPLLVLLADQDPGDRRIAEGRGR

>tr|J4WLL7|OS=Selenomonas sp. CM52

MTENETSAEVELGKVRLEKHAAAAPPEPREELPEASVTPLEPMKEPTTPEPMEKPTTPEPTGEPPASSDALPEPMNEPPASSDVLPEPMGTTLEPIAAQAELPKQPSEWIAIPFAPDSEPPASGAAPSKPTSEPPASNAAAAPADAVAEADDADDASLALSDEAVLAHTASLWQYHPVPQDEPDAAPEYKTEEKRIGMAEFVAARVRGKKHKHEATNCDDWFAVAAVGDIAVLAVSDGAGSKRFSRVGARAASEAAVGSIYEQLRTLDAANAKNRAAFALPMSDAAFGEACAKLGAALHAGVLDARRAVLAAFHERCGQEAYTKHLGRDLALSDFATTLLVTVAVPVPELGEVLVLSCQVGDGITCAINTHAPYGKAVKLLGKPDSGDFSGETDFLTSEAMADIASLQRRTLVTRGAYDLILTMTDGVADDYFNEQEMHRLYLDLVANGIVPGAQGTATFTRAEVEFLRSLPQPTTFPWVNDKEQHIALQYASHMCREIPTTEECLWQNAHVLALAAEDTSLKGVASHAERLKIWLDNYVTRASFDDRTLALLTWKEPPHV

>tr|K0EKR9|OS=Nocardia brasiliensis (strain ATCC 700358 / HUJEG-1)

MRIRVAAVTHRGLARAHNEDALGWGGWALQGATPATITTEIRVERPTTLVVCDGLGGHAGGATASRLACEFLTAPETVAVSVGAEQTRAIIGALLQRISETLDDLGAQRAELRGMGCTVAGVTVHPDGSALVFNVGDSRAYRLEGRYLAQLTLDHRAAHSNRLQQALGGGRRTLLDPAFFDCRLPSDPGMLLCTDGLDDYAKPEAIERLFAAGAADLPAQLRDLALAGGGGDNVTVLQVMACDGEGDDDG

>tr|K0YK38|OS=Slackia piriformis YIT 12062

MRCYRFTQTGAAHALRGGGNEDACLLAHRGDTVLAFMADGMGSAAFGGDAARLCVDVGSRVACAAFAPHELMSDESGAVAVKAAFLAAFNELQKDSVLANHPLAELQTTFMAVAFDARTGVLRFGYCGDGGLFVCLKDGSFAVAAQPQKGENVNLTNGVLDCDSWTFGRLENVAGFMMATDGLFDRMSKVDGSGRVIATLEALRAFAAAFSGAQSGAYCNADALFASLDAAESTPFSTVSDDRTVCVAASEDVRLSALRLASFRPDCERPFCKDDAAEPLCDESVAGGDAWSEYVPLPLCRSSRGKRTVMRRAAKKGALPWS

>tr|K2AMK1|OS=uncultured bacterium

MRERLNKLFGSNKEPESVKQAEGVAVAFFDALPSVGPAVANEVEAKRISLAVGQRTNIGQVRDHNEDNVDTKTIEDWLLLQAADGMGGYLAGEIASELAIKSVPEKLAQLVAESGQLGQKSMQQAIRYADKQMTGDMGTTYSGVLINRKTGEAILGNVGDSRTYLLRAGKLFVTFDQTFVAHKVFGESGHIDDIKDEDGKNVLANALGGTYGMQSDAETHQIETTKGDVFLLCSDGLWEITDTQDILDAMQAVNLGEDPQETCDSLVDLANKNGGADNISVIIARVDAPEDKPEEEPPAAVAENKAEDNPTIETERLEPHKPKRYDLEDGEIKIILGNPDKPWKTVLRVVNLKKHPDLKKDHPDYIEFMLIQERVFQKGDDKPGFIELSPKDKGIFIGRNWWSNEVLSLNNLVSRDHLFVQLIKDKDDRFYLQLTDLKSKNGTYVQIQKPVELNRPV

>tr|K2BT50|OS=uncultured bacterium

MATTARPTARRPREAAQRPRKGSERQPKQPTAKSERGVLGRMMDVGVQRLANRRDRALADVTEESIRPEEQLRLNAIDRRVGAAGRVSKDRMDAIVEEEKRYNISSGSVIEASPNHATCEDNAVVDDDLELYAVIDGMSQPRGNDVVARDIARLVGEKWKSVSISAITSPQEAETAMKNIMDEINGEINVQHNATAVRLQDRFGAVGTVVKLITLNGKRWAVVGHTGDTRLYVRHADGQMEQKTQDQGRTEYFLGLLQPKKAAELRKKFANVTDLAQLNRTKTLKKADGTDLIANDETPPAPVTEYWLYEQGNYVSQGFGVEGFKPQILCFELKENDQLLLTSDGIHDLVPDSQIGVRMNQVGVSPQARANALLADSEAGIRVGAGNKTQDDRTAIVVNIGVDRLNGEVAYAKKEYRGWANGERWGYLSVPVTTAQIEGLNRLLREEPGLRAKLEELAQEQYERLMASGIDTKGNWRNVGLAGRLDDIDVVQRLAPQDVSLVDRQGIYAAKVEDMITRAANDPTSLPAIADVLRASIKYMETTEIAQDDVLLNAVMDVLEQVALRSSDTKIEVSTLVGNSGVIALAAKLGFKPETVAELLIKQIGAIGDAARQQEKSQRSLKLNLAKAAMYAGTGIALTASMVATPLAALVGVGAVRMVDAFVTTRIHDSNRRKIEATIRTGLRDAVVEFKGTAEPERTNGQVKPELRGFVDSFVAMLALAKRDQITEYTASFETREAEIEQCASDVAIDVPVTRKKYDILVTEQENKLITRRRNELELVLAATEIAAYWETKRAYDDYIRQTDPQSFEVKRLETAHNVANERWLVRSREIDRIALAEAALYRMDRTTRLIETQAHLSADSRKNFWDRHPSLRALVGG

>tr|K2BXV6|OS=uncultured bacterium

MAEPERRHSPEPRKAEPLVVVEQYEGHEAIQDLDTAQLFLQQWRGVLEKAKVHFETERGKKTSLFARLQRWLNQANVAEQQMITLSMDIDKLLSQLRSDKPDVALPAILEKIRVIEEAYQQAMGNAVTIEHDQMIERNIEEEQFEIATGGVMKADWEKSQRRRDGKLGKYGQDCLRIDVNKGIGVVADGNSSSGHSYEVARRASRAAKEILQNVDEELYRSVDEIRWFVDGELNAILHAVNEMDPFLTGGTTLLAARYIKKFDAVLMIAVGDTEACVVAGQDTLPLRKNERSSGALEHTSGIRKIQNDAPLIQKNADEQVVTVASLAEFRRTHPGLELHLALATDGCINNTGKSLDQVAVELVQHGPRALTEGMLRKDDLVLIDMALPTPTVDYQVQKVA

>tr|K2CIA6|OS=uncultured bacterium

MENKPHDLGLVERRYFQTKALELAEELGMGMKEGKLTRRDGWLNVIQHELKEAVGVGVLGELLGLSTEEIEQLERAAALHDWDKRRDIKPKDFENEMPTETIVEQIDPAVRHIMQGTKERYILTELAQGRGDFLQNLLFYVDNMTAETAILPWPQRLDREEEKRPDATMAKVLGSLLPPGVSYWQTERRIMEQLDRELEQRLRKNGYIKPNGVETATFIAHLINQKIEEVIRRDVELLNLKTIMIEVRDQRRGDDPELSEDRSSVIEDENWLSATVVDGASTFGQKINGKTTGQIAAETATKTHNTFGASKDPLSALLEMNTALSEKARLLTEALTGEGGETFLKTLDLFGAAAVAIRLNKKTGVLRYAAIGDCRLVVQRPDGRIHHYVLDKVTPYEAQEISLVQRLCHEKGLTPQEAFSDPESQALITHNHHEVENRPDGTGYGMLKGITSNEVLEKYIETGMIQLEPGCKVYLMTDGMLPPAEENEQNPGQVKELQRRMLMNLALKKGGIAELLRATRELEEADHDLKNPRLKTHDDACGLEIEVVLS

>tr|K2ELY4|OS=uncultured bacterium

MVLERIRVDKDKRYVWGRDKHDFGLEKLGPGEIEKKSGSSDTVSFELRRRSSPERKDGLVGDNYCLLPDLDVYAVADGVSESSSDSRSAAGVSKYLEKVWQKNLDKSGKKIDDLSLSEMHDLMLKVVSDLQEIFLRHNKKNPEIFLATTFSMVKMYEEYCLVVSVGDSPVFLQNKEGLEQVTIAQDIIRQGTKDRDGNYLPKSIDELYSAELNFDRVALQTKREEPGIIRNNPTSLLGLEKGFSVDFYIRKLKDGDRVLLATDGAVKTNDNLSLDSKKTLSQVADTLTDNFDGNDDATILILE

>tr|K2GUJ4|OS=Entamoeba nuttalli (strain P19)

MHQTYSINFKKIFQKEIKTIPKKEIKRINNKQYSKHLITDTLSIKSRDQHFAMASISTYPIINGVKQGEPNCDKAGIVRFSNATFIALADGCGWGDKPSFAAKLAVTSSLEFVISNITTVSTLREIASLLVETIANAHQIICSSYPNNNAQTTLLVALSCTLIDEQQVTVVLSVGDCRLFHYSLRECKVKKIIGTGHHSIDETKECIAALGNNQLGIDIINGSQLAVCECEEGDLIFALTDGMYDNFDPTIIKEKKPYDQLMNEILGIRIKRSANLNSLVEELCSYTIGITQTVREFHQNNPTKRRRDGLPGKLDHSTIIVYSINTQNLDIGLIDKYSPELFKTLKPLETKKPLVHSISFDEQNSPLMPFHSDSVRTIKNSSKISIHSVRLSTFSPPRTALNDHYTPCLTPRIYRDVV

>tr|K2KTX5|OS=Helicobacter pylori R038b

MRDFKAFGASIRGVYHAYARLPNQDAFLIRKNQKGLLLVLCDGLGSKKDSQIGAKKLCESVEKALNAIDIQGCNLALLNKVIFSIWECLLYPLEVRNALSTLQLVFIGKEKAFIGKVGDGLLAVLGKEHRLLREDKQDLTHFTTPFDRNTLLTWEVLESKKIDALFLCTDGLDESLIDARRLDFVKDIISEFKHKKKASKKCYALLKNHKFYDDTSLIIAYRKDSLWS

>tr|K4QWL4|OS=Streptomyces davaonensis (strain DSM 101723 / JCM 4913 / KCC S-0913 / 768)

MNTAAAPGRSAAVLIGVSRYDHLAPLPGVRGNVVDLAEQLRDPAVWGLSEERCRVVLEPTDATAAFEPLRETADGGLDTLVVYYAGHGLIDPNRGDLGLGLPGSVVGRPYTSLPYYWLSEELKGLRIERRIVILDCCYSGRALGMMSDAQSAVANGAEIEGTYLIASADESAQAVAPPGARHTAFTGELISLLAGGVPDGPELLPLDTVFQHLSAACRSRSFPLPQKRVRNSAGQLPIFRNRAYAPMRAGRLLAERYELGQLIEADSATETYAARDTELDRPVLIKMMRPEAAADAALAAGFRRRAKARAALRHPFVAVLHDIGTTRRDHVPCPYLVTESVAGETLGTFVRRRQNHPDWVVAVVCELLGVLEHAHGLGVFGWRLDPESVVFTAENHVKVVDLGDAPDGHDDLLEVGRLLRTLLAGAAPPTSYEVDAVVRRALATDPAQRYRSAGDLWRELYDLRGRATRPEPVSAPESLWMRFAAGSHKGMIREQNEDSGYAGPRLLALADGLGAAPAGAVASSEVIASLVELDDDTGDPPDLLTPLHAAAQRAQRQLAAMAEEDPQLRGMATTLTALLWVGSRLGLVHIGDSRAYLLRDGTLTQITQDNRPMVSTAEEVDPEFWVRKALAGDRYLLCTDGLSDVVSDESIEECLASHLHPQETVGALVTLALRGGGPGNISCIVADFLATREDDGPLSDTPVVIGAVAENQTDLYAN

>tr|K6VTH1|OS=Austwickia chelonae NBRC 105200

MTSTPGRPEHSHPFEAFMPPESGVIPPARAASSGPFPDREPHFLDDEIPTMPVPPPRHAAPRTPATEPLPMSSWVPPEEPEGLSWSPQVETPAEEERAVPEERAVPEEQPAEVSAEGDMLVLGGIASSAEQARGGTSGLCRECHGSYSPDGYCENCGAKAVDPRHHFEIDVTPWLAGVCDRGIRHRGNEDALALHGTSSPYGPRASLVVCDGVSTAPHSAEASLDAAEAAVQVLTDGWEYCAEEMPGGPVGELGRRVEEAGAAAAKAVARMSEPFLSEGEGGPSCTLVAASVEGETVVVGSVGDSRAYWIPDRDPAEVLTTDDSWAAEQIRSGVPRAQAEAGPYAHTITRWLGEDSPDQVPALTPLQVRVPGWLLLCSDGLWNYASEPAELQAVVHREAMRAAEAGRVIEGTAGSRGADDPGTPTALALAHGLVAWANGCGGSDNITVALARLVPAPGQSESSSWEEDLPEPSASPEVEDMGLAPTIVMPVSRPGRHSLEGRGILSQSRTNTERG

>tr|K6WS89|OS=Kineosphaera limosa NBRC 100340

MSDPTQPLPTIRESDADSSGQDDAAGGAPVAAGTPAAAGAGSTDAAADQGAGAAPTQELPVPEPRPLTPPPPTMPESADGMLVLGRPAPAAQPKQSPPPAAPAARCLECGGHYGPDGYCDRCGGRRPDPRHHFELCAPSLPETPTSPQVGQTIAPDEVPPQSRAVGTDSAESTDTTVQFAPPAGNAPPVLDDPLRSTAKPNPLTWVAGVCDRGIRHVTNEDALALYADPPAPGENRAPRRAAIVACDGVSTATRSARASLAAAQAALSVLTTSRSRGLAGVASSQAGAMAGRLDAAVDAAMDAVIDVADGPAQEEDFTETGIPHMSDPACTLVAAVIEDDLAVVGSVGDSRAYWLPDDGREPLLLTRDDSWVEEQVDQGVPREVAERGPGAHTITRWIGPEAPDPTPRKTTLQLREPGWLMLCTDGLWNYAPTPRALASVTRQLARRLAQPTPPLALARLLVEWANAQGGRDNITVALARIDPPTGADPADGAGAAEAESGAGEAVGAAAGEDGSQSAARDGLDDGGTDGTRPSDPREAH

>tr|K9PBA9|OS=Cyanobium gracile (strain ATCC 27147 / PCC 6307)

MNRWVAPIGASRTGAAHRRAGRPCQDAVLCRELRGLEGQPVMVMAVADGHGGRRYRRSEVGSRLACETALAAVQGGLAARSLDGGEEGWSRWLERDLPEAIQRGWLEAVAAHWQSDPGGGGFEALLYGSTLGLVVMTPRWWGHTGLGDWDLVRVEADGQARLLEEEDEPTALGEATCSLCQPRAASLFARRAGLHPLDGGAADFALVLCTDGIRKSCATDGDFLTLAAWLARGDGEADPEAAGTLAAALDRISREGSGDDVTVAIGRAWSPGGGPPPPLPPPPVAPASLAPPAPPATEPAQEPLPRRRRPAIGRAVAIGLALAGAVGSWLAWNWPRTPTALPAAPGSPGLRAALSEVERLCGQPAAIPLELRRRRELFVALSQGRRDPAPLKAAAAADPLAALIAADLPAVPPGTMRGPVPAPNPEPRRPLRALGACPALLEALDRQWAVTPAAPSP

>tr|K9PXI9|OS=[Leptolyngbya] sp. PCC 7376

MQKQHEQPDNLLVIDIETKPNKGQDAHLYLSYLDSFVLAVFDGLGGRSGMMGSEHGARVASRSAKTSTYSFFQDLINKHQKLDKTNAFDLQRFIQQDLRKSKNDYERTSNKPSSRIKSNLKPSKDIASTTLALANISKSRSSQKALDLDVAWIGDSRVYFLSSRKGLQQLTQDDVKEYKDDFDSVSNSGAPMTQYLTPDMDSDVGINFNHFSIEEPGLVIVSTDGAFDGLHPWYFEYLLLFFLQSSGSMREYEQKLSEYLRERRVGDDITLILQPLAFGNNFSSIQEKYQERYRTVEDLANSCRELKNYAEVWNKYRVNYEERIEQTASQVSNDSIALLCDSEVINELIDIDLDFNDPEQSISKKSIQPPRGSQQFNEERAPLEQPISYGANQKNPQERINELGIKESKFSNQPNYQNPSLDIDNSPSQNTLPPIQPLSYNTNQDHPSDISKTTNRVTAPSEEPNTNVYVPDQKKQAQPSNPPYPSHVKNKKIDLLAKASGNKISTHCIDYIYIAKGLLYENKDYNSAIQTIIQILKSNCEEIPALSFLGLAYFMESNSGVLAPLPKTDKWANVFRQTLNIIEKKKYLEKDSQHESIRISLIILVECLTQVEISSKVFSSSELKEIEAICRKIKTYREQNPHPWYVTGKIYAAQSKTIQKKEEKNYIEQAKKDFRYAQNIYEQRGDTRRVNECNKEIKKLDSVWNKRIF

>tr|K9RS27|OS=Synechococcus sp. (strain ATCC 27167 / PCC 6312)

MLVCPHCHQFNAETNKFCLYCGTPLVPPAPVENELSSPTSESAAKSSSQQLGAKGTAELAWSELGEMTWTPDLLTAANSRPGDQPPSPANLPPNPTEADSPQTETPVSLPVAEAYPGDDELTLIPDAIDIQEISRDLLTNFGSQSEPISAVVVYPCDSDSPWLACERRPDLPPVLEQDTERQEGAIFLDEENRYRCLTSFSLDTTSGRVEDTQPSRPTRVLEFSQTLIQQADQFDLTQPQEMLTQLQARPDAPTPIVISYLSLRLLFSEFVPRLQDAWVGYGTGQPGIHEPRQFVVLQDRSQCPSLWQAWQDPELDNGQLLAWMEEMIDLWPNLEAWGCAASLLDEDNLCIYGEGILAVRYLDFSPLPDPSPQGLIALWQSLLSQAPASRQQAFQPLFVMLQTQPQQTLADIQALIEQLWETLQSDLINLGNSQDFDLKNVVASLDAHWQQDLNSTPAQAITEDSPTAVLPKQLVQLDTVGLTDTGRQRHHNEDFFLIDHRQHYCGTSIGQKLQARGLYVLCDGMGGHAQGEVASSLAASTVFNFFQEHWLEGEPLPEDEVIRQAIFAANQAIFNHNEDQRTIGSGRMGTTLVLALVHNQAVCLAHVGDSRAYRFTRRHGLEQLSVDHEVGQRDIKRGVEPEIAYARPDAYQLTQALGPRGNDYLNPDILTLEVTDDTVFLLCSDGLTDNDCLESHIESHVAPLLSFDSSLSKGVRTLINLGNEHNGHDNLTVIAIRMKLRSELDQIF

>tr|K9S3W1|OS=Geitlerinema sp. PCC 7407

MLVCAQCQFENPLDHKFCQKCGASLTEKPCFQCEQPVALGALRCPNCGAMTGTLWWAIAEATGTEPPAEWAQWQAALTAPEAPPLYVDEQQRYHILAGPLSKDPTMLEFRVHDQRPLELSPLDMVAAEVMFKLAQGLESAETLDPRLPAIAYPYLGLQEEGNLAVPMLQDAWQSPDQTVLILEDRSQWPRVAEQWQEEQASPLQMLSWLHEMTHLWSALEPWQAQQSLLDSENLCLDEDQLLCLRRLILTEGSEALPLAILGEAWRSLLSLSQRTQPGELGLLIEDMVAGEVLTVEGVQERLAAIAQSLQVLPETAPCAEDLGLPPELQPAIAADWTVDDDDDDEATRPLLTPVASFPDDSELDFDAGDDSFDEMPTVVLPMRLVGLEESGRTDIGQHRQHNEDYFGMQSQIYKIESPSGQTVRAQGLYILCDGMGGHAGGEVASALVVEVLRQYFQREWLSLPTQASEASNRGGGLGEDWRRPLPDEDSIRQAIHLANQAVYDINQANNCSGSGRMGTTLVLVLVQDNRVAVAHVGDSRLYRLSRKRGLEQVTLDHEVGQQAILQGVDPEAAYGRPDAYQLTQALGPRDSSYVKPDVQFFDLAEDTILLLCSDGLCDNSLLENHWVSKLEPFLSSRTNLDLGISQLVDFANGYNGHDNITAVAIRLKVRPNLEAMQRT

>tr|K9S467|OS=Geitlerinema sp. PCC 7407

MEDQFEMAAGSVIGTEHLRTGKNNQDAFYSLRQPMVTVAVVCDGCGSSAHSEVGAKLGARWIAEAIAQRLDAGQDLSEPFWWGVQRSVLAQLRALVERFGGDRPQVVQDYLLFTVVGAVVTPTETQVFGLGDGAIAVNGHLQHLGPFPNNAPPYLAYGLLVPDTARSPILDLQIHHSLATQDVQSILIGTDGLEDFCRAADQPGPDPSTPLGPLSQFWQDDRYFRNPDAVRRQLTRLNHQRTIANWQAQQLTKFGGLLRDDTTLIVLRKRSPST

>tr|K9SFY1|OS=Pseudanabaena sp. PCC 7367

MKAKPEPESESAQSASIEQEKPQSSNGQPANLASLFEVASGSVMGRHHRLNGKNNQDASYYATNDAAMAAVVCDGCGSCQHSEVGAKLGAKLVVNTLLQMLVCPPGGKVADPTNNFFWQLLKQNLVEQLGRVAIALAGFCPQPDTRFANHPQAYLPEVKQVITDYLLFTVVGALITPQITVLFSIGDGLVCLNEQTIRIGPFPGNAPPYIAYALLKPKHGMVLPSDLSIHAQRSTSVVNSILLGTDGVDDLIAMAGQKLPDKHELVGPVSQFWQSDRYFYNPDLVRRKLWLINQEGVKPNWSSRRLHKQPGLLPDDTTLIAIRRIGTMQSDRNLAATEEELTTIQSVAPRKIPQKTSSKTPKKMK

>tr|K9SG17|OS=Pseudanabaena sp. PCC 7367

MNKDDKPTSSHYLWAVGLDIEEFSAGTMFGDRYRVVDAQIVEDTQPYQFPEAPEELPIDAITYLKLSRLLLNLPRPYAVLQWEEAIGTAQVILLENVPIDAQGELLPNLRSSWASATPLRQVNLLWQVLQLWQPLAQQNMCATLLEQDFLRVDGPWLRVLELKPDATATELTQLGDRWVEWLDDLDKSKVDEDYYELLAEFFYDLGRGDLEISDAIDRIDAIANRIFEDVPLTVRIASATDVGLRRDHNEDACYPYPEQQNREAQPDILRDRLAIICDGLGGHEGGEVASAMAIATIERLLKTLLQQVEDESEPFSADSFMEQLVKIVQLVNDQIVALNDQQQRLAQQRMGTTLVMAVIPRPYGKFGSEVYIVGVGDSRVYWLDRHGCKQVTLDDDVATRDSVMGYNLYAYSSQRADGGALIQALGTRSSDLLIPRVQRLRIDDDFLLLLCSDGLSDFDRVEQLWKIHIQPALTENLSLSGTCKDLIEQSNKLNGHDNVTIALMRGRLAEPDPNEEAPTVETLIPAAALASDDDDTAGDAPSDKPTKAGKSKSSALVAAGKDPDSEQNQNEAGMDFEDEPDAAAVAETLVTKRQGGAGFFVVLVGLILIGGAAAVLIPQSNRWLGEQFPWLEQYLTLVRGDRIKPSVSPAEENGSEPTEGANETEPNPERSPDPETNPEQNPETEPSSNNPGGDETSTNDSADSPSETDRRSNPGGDGKETDVINTENIADPVES

>tr|K9SNN7|OS=Pseudanabaena sp. PCC 7367

MISCSSCGYANPAGHNFCQNCGSRLAEVAAKIDPPPTVSPNETVGNKTIGKNIKGVSDDDSNDSSEGISTSQAELNQQAEANLPALSQSIELDHDAGSDQNQNRDVDLEQKAQAESEDQPDSELKPNAIATTVQILRQPKTDEEGDRSEVSGNVDAQPTQQSSPAPIAPDLDSELELTGDSGDVVEQAEPVQAKNINLEQADADLDIDPDIEIPNIEIPTEPDQEARSELAPSSDQTPDQMLDPDQVPTLEIENLSPAAAIEGDRPEPENLNLENQDQPSNQELSLDPEADLMAQAELENPIDPLPPTHPDRQIEPSIHEQALDQSEEADQIDNLDENQVPTITGLVSTEPDTSDQPDKLTPDYCLSELQSAGLSDVGAQREHNEDAFWAKSQAIATESDHKGLIQAIQGVFILCDGMGGHESGEVASRTAIDSIAEGFTPFWQEDSIPGEQTLADIITAANQAIFSLNELEQRRSAARMGTTVVVLAVYDTTVAIAHVGDSRIYKITASELNQITQDHEVANQLIAQGVAEDMAKARPDAHQLTQALGPRANAQINPTVQFLELEQDTLFLLCSDGLSDNEVVEQYWRSHLLPLLEPGADLKNGVTNLINLGNKLNGHDNLSAIVVRCRVGSITN

>tr|K9SQP3|OS=Synechococcus sp. PCC 7502

MIKCSACGFDNPDVNKFCQNCGIEIITSENSEDTDPLELANSGLADSGDITSGDVGEDVTDPELCNSHLISLKYAALSDVGKEREHNEDGFRCFSQFMTTVSHSQPEFQTHRGLFILCDGMGGHDGGEVASAIALESIAESFKPFWTSGLPSQEKLTEIIGIANQEIYDRNEAEMRRDAGRMGTTLVILVIYGTEVAIAHIGDSRIYKITADGLTQLTRDHEVANRLIDQGVSETIAYSRGDAHQLTQALGPNISASLEPAIAFFNLDSTSLFLLCSDGLSDNNVVEDHWQQLQSIDKNSDLSIAAQDLIQLGNDLNGYDNITAVLVHCQIEQN

>tr|K9SVT5|OS=Synechococcus sp. PCC 7502

MLEKLEIAGGSITGRNHTVVGKNNQDAYFSISNDQFAIAVVCDGCGSGKDSEVGAKIGVRLVVEEVADQLQIQPQLNQEFWEQVKLNVLEQLQGIAKLLSGNCSEFISQIINDYLLFTIVGVVITPIETVTFSIGDGIVFVNGEVSYAGKFPDNAPPYLAYGLYHADLIDFHIHDRLPTGEVKSILIGTDGVADLIAAEFSHLPKKAECVGSISQFWQDDRYFSNPDQIRRRLSQVNYEFIKPNWSNRTLTKETGLLPDDTTLIVIRKKSS

>tr|K9TLH1|OS=Oscillatoria acuminata PCC 6304

MLKQQSPIDILAGLILLGCLVWLILENLHRDKETSTENQNQDNQEPNSKDNPVVSKPNEINPESTLPIEVDSSTQGTEEQTDEQSSRSDKVKVEIKDSVELRALDDYPVEIRRFQIPKQGLTEAECEDKSALSSTEGRLRVAVADGATESLFSDIWAELIVNSYVDKGAEIFNIGSLQSLSQAFLHTASKLILQMPETRHWFMYEKLERGSHVTFVAAEFYNPETMEVLAVGDSCIFWRNEENGNVEMLPELSAEDFGVFPASICSLQKTWQNLESKIVKKEVHLHNGFQVILCTDALACWLVKALQQDPFVWEKLFQLSDSISFTNFIDSLRSQKEIRNDDVTLVLIHAIPINVRLP

>tr|K9USL3|OS=Chamaesiphon minutus (strain ATCC 27169 / PCC 6605)

MNNFNIVKSINIGSRHQESNQSCQDHADYKFICENQILIAAVSDGLGSAEYSGDGSKLAVETAIIELEYQLKQHVNNLNNISGNEEQAKTIFTHVLKSVQERLRKRSRELIGNTRSNDRSYYRGKELDCTLLAVAIAKDFIIAIQIGDGMIVSHNFEANDYCILLDSTQDIVCGRTLTVMHPECIVKEVKLYYNKILSEQILENIHIIGANNMNSESSKRISKEIQIYTSNNVPSFICMATDGIEDFAFEGMCNSKYILYQPLFQSIDHLMKDNKQSDCQDFIDREIKSEEWQKCGTDDKTMLIISRPQSN

>tr|L0H415|OS=Thioflavicoccus mobilis 8321

MDEDQQLSIARALAEEAVRRLAETGEEAGEPIDPAALGPESALIRSALNLLAEAQAAAAIATPVDDDEPDDPPSTPVHDEEPEAEHLPHQPPATETDPSPEIEDQGAPAHNTAQDRGPSPEESIVAPAATNANDDPQATEGESLARDTDESSTSPERPTDAVPEPSAAEPPHPRIAFAVSANARVNESFQANIAVRGGEPEAVVVLDCEVPDGLGLAFDPAGAILQGIPTSAGDFALTVHYRFADADPDRPTMAATAMLTVNPDPRSLWQDRPSDREAAGWKSDAVHTLVAAGEDRRVVAASKRGRSHAHIGGFRDDDYCILVEEARPWTLIAVADGAGSAERARIGSHLAATTAARRAAAYLAGGLGETLVEHTLAGEAAQQRGAREVAYEVLGGAALAAVKAIEQAAERDGATVKDYATTLLLVGHRPLGERHLLIAFWVGDGAIAALEPFQAHLLGTPDGGAFAGQTRFLDRSIVADANAIMSRIRVTTVADLQALLVMSDGVSDAHFASDQDLADPVCWQTFWHLIEPLLAGEHPGEELLAWLDFWSPGNHDDRTIAVLW

>tr|L1KTM6|OS=Streptomyces ipomoeae 91-03

MVRPTDIQGTESGGGELPGPGRGVRFDRRREPDDYPLAAPLPPDPRTAEPATPPPGMKLCVACRAGYVDRDGYCENCGHAQPRERDHMEQELGAVAAVSDRGLRHHRNEDSFAISSTALPDGSPAVVAIVCDGVSSATRPDDASLAAARAANETLLESLPRGTHPQQAMHDAIVAASNAVNALAEDPQTAAEHAPHQNAPACTIVGSVITPSLLVVGWVGDSRAYWVPVDRSAPAARLTEDDSWAAQMVAAGLMNEAEAYADERAHAITGWLGADAYELEPHTASFKPDRPGVVVVCTDGLWNYAEAAEEMAEAVPLDASARPLHCAQVLVGRALDGGGHDNVTVAVLPFPALPQGAGSA

>tr|L7FA51|OS=Streptomyces turgidiscabies Car8

MALFGKRQNETERRPDRSEPGRRPDSVQRPDSGQQPDPARQSDPAQQPYAVRQSGAQHGNGTGVPGPGMAPGSAPATAPGKAPGTEKAPESASEPGAAVPTEAPAPAPVGMNIAGHWRPIVVGKPVPPFDTVPPRGLSYRPDTVCDGWQTGKMALRLASVRGYQHRYEGRPREDDAAAAWDEGTGTVVFAVADGVSSARQPHIGSQLACRSAVDEMLSQVRGEGAGYVADWDKLLSTVHWQLVEQARRILHRPDAGPEETAGLLATTLVAGTATPTDQGVFVHLISIGDSGAWQIKHDTFYRLTGGKAAATDGLYSSTVDPLPFLPGVIRPLSFALEQQTVLLVGTDGFGDPLGDGKGAVARHFYYGLLRPVPPLGFAHLLDFSRETYDDDRTLIALWPWNDAPGGPSC

>tr|L7FBS6|OS=Streptomyces turgidiscabies Car8

MIATTPALLVSPNGEVEELLLDSRHADQLHGIGFYVQAPHAHRLGRRAVVHAGHGVGSVNDVAQQAWMSITGGSRPPLLRGPVVITGATNHAGDLTPLPELSAEAIQRAAGLLSGATAIDFKVSAVQHRGGRKYQCDAYTVKRNKNTGRWAFAVLDGIGDRPQVHRFARRFASLIAREAARHGSPTRALAAARVQARNELRWDFDPRTDPSAVAVIAVADHRSPLIHVAWCGDARAYRLAPIGISQLVTHDHNYAQALRSRGRTPRPYDRNFVTSCLMNGDIGTTTIERDHTRRLLLCTDGAYAPLEERGQDVGGILDLVDDAKDAAALVVDDAIAASADEFADNATALVVDIAQA

>tr|L7LLT6|OS=Gordonia sihwensis NBRC 108236

MDEPRILRDDALTGRDEVGDVVVDWAVVCHVGRVREANEDAALAVPGRYIVADGMGGHDSGELASEAALLTLAQAPVGESSADTRRDIAELLEKAQERIGEFGTETGRRAGTTATGVVLAIEDGVPNWVAFNIGDSRTYLYSAGELRQVSVDHSQVQELVAAGYLTSEQARVDPRRNVITRALGAGMTVPVADYFAFAAADGDVVLLCSDGLDGELSDDEIAVILEASPDNEVAAAALVEAALESGAHDNVTVVVLSATLPSAAAEPDPNATASQ

>tr|L8GL94|OS=Acanthamoeba castellanii (strain ATCC 30010 / Neff)

MSGAKEKKQRSKEKAKPEEKEKEKKSNKSRSEGEGEEPTNKIKRRRSFRLSRGATNKEVNKAFAKEEEKAKEKESKQKRLSKSTSAIASAALTDRHVKSDGDEPEKGGDKSGGSRIHHKKKAGQEEGEVSKKKSTQVVDKEPGSSSFVTAADSSDNKDIMEKDRGKRKSKSDDEWERVKRLIDEAEAQKGRAVKEAEVVDEAVAYYAQKAETPRKDGTAASSFAPASSSSSSSRKRLTVSLGPGASPRSNAMGSNRMNTNARRVSNELNIKVQQFNIEKEGVFKSFKYQQLPPLRVKPLSSQSNVIELFGPSDEPGAANRAEMLPSGDVYCRAISTYPWDMHASAQAEKAAAAAVAADPAGGLNTNWSQVFEGVNIVSSADGKTKAGARPKVFANRTLVALADGCNWGEKPKKAAQTACQAFLEHMKSKFGQVNDIKTAGHYMLRALAHAHTQIMNTCARDAQASGKTMKVTDGGTTTLLGGILLELEESEAEQLGSQWVFVCVSVGDCKAYLYSPSSRQVIDVTRAGNRSAKADMSDPGGRLGPTVGRGGDADLRNLGLYMAPCEEDSLLLVVSDGVHDNLDPEHLGLSPNDVGVDQDKWDSNFEALEKAKHLFRSSCLMQKIRGSNEEVRPTPEDAVNRLLDHCKHVTQRSRQWMMANPTRRLPNDHINFPGKMDHTSCIAVTVGRRQQPKEGGSSIIMPAPVQANTPITVTVAQNEQQVAVVCRTLLINNLSCSVQGRKIEFTLRNPDATATKSASIVPSGFRVVGDNEVEAKKWKRVVDLPCRVALDSQQTKQLDDHTILFTFTKAL

>tr|L8GMX5|OS=Acanthamoeba castellanii (strain ATCC 30010 / Neff)

MKRRGGLAPCELVPSASACDETIATTGAGVVTGRSRAYIPYEADSNCDFSGASEQLPLALAIDGTGHDKGSDAAKHSENGRAILKEVEACIRECLDDVQLPQQLADKVLALLKALEASDDSRIHHISCTLSVAIKVEARMKAYALTFHWGDNVIFIANPKRGTLALINPPLNSDYGIGATHSVSSRKQGESLLPHSLCVRELEADDHIVCLTDGVWDHFVLSSADAAGAKKKQGPPELDETALLAVFEADEGEERNSNDDAVESIVGRLFEEARNRARAKMEAKEMREAELDDMSAFIIPPSTPALVLPNAAASHHNNKSDKKSCTLS

>tr|M1WUT8|OS=Pseudodesulfovibrio piezophilus (strain DSM 21447 / JCM 15486 / C1TLV30)

MTTSSVQYAYESLKGTNRTSNKDGVGAFDLDIDGYSLFAIFDGVSSLANAKNGVNLALRELGRIFYENKHNNDIDLVKIVESLNAKIRESKYKKPYTTCAILAIPKDKTRPVKIASLGDSRIYGVDKQFIEQLTEDHHIDGHPSVLTRYLGMEQLEHSDIFYKEFISSFSDYMLCTDGFCSLFEKDKSLFHDIFLRHTPTGAKKEVNKLIQGKNQDDATYIYVRTNDV

>tr|M2RXT9|OS=Entamoeba histolytica KU27

MSKDTPYHLLFRKAFKKDLGEIPKLPIDMVDTKQLAIHSAERKKPATIVSRDQSFGLTSFSLYPIVEGKKQGEPNADSLKFIQFANCSIGIIADGCGWGFRSKKASSNAVSGCWESIRSSIGKCKTTREIATLLVDSMASAHLSILLNSDSSLLETGTTTLLISIIVTLRTQQPYALVISIGDCQAFLYSHEIEHTDSIIGDNRKGTATYDCGGVIGPAEGKNPSLRHVELKGRYVKENDILLMMTDGFHDNFDPDICRITSDKENYVNSIISPIIKQSVSIGDCVQQLSEHIINLTDKLAELHSTLKRRSCIVSPGKLDHSTLLVYNISLHNIDISNEDCYVPNYYFKLYENPSRISISVNKHLLPTTTHLKPKLNESLSQPITPSLTPHFQFSTPVLTLSKPTSPRRLRALSRESLRALSCKSISECNPNGQCFSSSPSYSPNLSPKHTEESKQVTTSSNSPTEPYHSFVQFFMQHQQN

>tr|A0A4P5VZ80|OS=Deltaproteobacteria bacterium

MALDGAARTHPGKQRTTNEDALVCRPAAGLFAVVDGMGGEEAGEVAAAIAVAAIAEVPDLADLAGETVLSQAFHTARERILAEADSDPTKEGMGAVGTALRFDDRGGSVSVAHVGDSRAYLVHAGGVRQLTHDHVAEASPGKKAQVARDLGRRDLRGEWVETSRAKVARGDLIVLCSDGLHDVVGAEELAREFIKLRGEAKSADAIATRLVGMALAAGGPDNVTVVVVRVGRYRRGAGVGGVSRPIWLVLSIFAALAVGGWMWLQGGLRPEALPEIVSGVTELGATAGLSVPAASRTAISGRATFRVSGERISGGDWTLAVAPEGAVQLERVVIALERELVLEVAAGGEVLLRDLRVESGRVRIVAAPGSRVLLEHVRVSEPGALVIEGEGLVSRSDVGLIDAPRVPVEGAP

>tr|A0A4P5W8V2|OS=Deltaproteobacteria bacterium

MPLYVKSATDVGRQRSVNEDTVADVRLGDEEWLLVVCDGMGGQEGGQLASTTATAHIVRWIEERRDEPEPVPVIRDALLGASEAVAAVAAARGVPSAGSTAVVAWTRGNQVWMGWVGDSRYYRFQGRDINGRSIDHTRVAEMIAHGILNEAEAKAHPDAHILTRALGGGRGLAEAGGPTVWKEPQLLQPGDTLLLCSDGLFDLVTDQEIPEIIADCDLATACSRLISTANDRGGHDNISVIVACWDRVAVPALPPPPPPPPAPLPSLEPRRTLGDDATAVLWTPDGREARAAAPVLPFSRPSESFAAPPTASDSPRAPLKPPGSGRLPAGSRPPRTILPRLIDIGLGAAAGLLFGLVAGFLLADVVKPWIYAATAPPIATAPLPTTPAAAAPGVVPAVESNPAGAAPSANGAAAPEPAPEAGTTAKPARSESPSGPATGNPGSPKGGGSHPPGPVKGPKNGAGG

>tr|A0A4P6JT30|OS=Ktedonosporobacter rubrisoli

MSKQLRLDVAQLTDVGRKRPHNEDNMAYVIPKDAQIMAKKGALFIVADGMGGHAAGEVASEIAVDTVSNVYYEDDSEDIAVSLLHAIKRANSLIHQRAAENMLRSGMGTTCVSAVLRGNMAYFANVGDSRAYLIRGGQVKQVSQDHSWVEEQVRAGLLTRDQARSHAQRNVITRSLGTQPDVEIDIFAERLEEGDSMILCSDGLSGLVDDEDLRSIVDQYVPQESVYHLVERANENGGPDNITAIVIKVLEVGMEEPPSSLPGVLFPVPVGGREADGAGSMLKNLPHSAIGLPTRPEDNRLNGSSLRISSGPLGAPGGMSSYGATPQQSQSTRVQAKRNRLFYTILAVFILLVLAGAGAGVWYFMLRPQHTVNVDGLLKDANVQVEQANKILANDPATALKQLSQAQSDLKQLQEVSLSDAQHNQASGLHSKLVSTTKTAITNYNQLAAIVPLAAAGPCVNATNSPLDSGTTGTQANILAVIQNGKNKQFFYAVGADQKLYRLNDQRSFVNKISAVQDVLVQAVVSDGQRLVALASQPKDNSYALHLLLPNDDGTLKDSGTATVDQKYMQNGLVPQFVAAWGQDVYVILTSKTAPNTADMLHYKISGDNQWEKATPVNATLSISNTLVSVAAFPDGRLFLLYSNGDVQTLQFANGGNLTAVSVVMQSAIPVPLSATAADFSPTSAVPTPGTQGVQAKSFLSVPGASMLAAGLIDSDNAPHLFILDKDHFRVIDLKVAQEAAMTATVTPSASPTSSTGNGNGGTGGGTVSTSSGTQLSMTLVRQYTSANLLAVMKSAVADPHGASLYLLTQSAQSNATPGLISIDMHQQNDQQSACASS

>tr|A0A4P6K599|OS=Ktedonosporobacter rubrisoli

MRLPDTGLYDVFLALLNRIGSIVEECELIYEIKPWLRYAIWAALVSTTLFLIWVAGGFPPQAWLLLIQLLLHLSHLWSFYGSLILLPLLALSVLSMIWLVVWSLLVWGSVRLACYQWRLYQCREGQGISRWLPAQKLQLVVANAGDNATMEATLSTTVPRLRLPQKVQLKPDRLEAAHTSKASRALSDIPTRPSESALSSESLAEATTENRSMLAQSLSVGVGWHTGITRKRSPNEDSLLVLQGTCTYQGRLVPFGLFVVADGMGGHAYGQEASRLAIQNMMHTVLQNIVMSNDLDDEYLTNMLIGGVEWANRAIYQRGQEWGQEMGTTLTGALIVGLKVYIVNVGDSRTYLYREGMHLAQITRDHSVVAALVESGTIAADDVYTHPERNKVYRSLGCKESVEVDWFVVDLCAHDKLLLCSDGLWEMVRDPMIERIAASGYDPTRICSMLVQAALRGGGQDNVSAIMVELP

>tr|A0A4P6K5K3|OS=Ktedonosporobacter rubrisoli

MGAVIFMAIKVLWDWLRNLYEQRRFAAELQEAEFLAEQMAMSEVEKRMGLENVRELEMEWQPQTALVPAVATPRRASVPPQRPSSLRQEEEIYSDNAIPAYMFSSGKQRVMPRARLAPMEKKSSPVAVAPSPVGPATQRQERALPQRRQWPREVKPPTSRGRSDLELREVKPPTSRARSDQAVQVTRQHEEVSRAQPAYRASSAQVSPVQKPIQTSAFTDDLVNSEDDDATLPYLKLINQGDTLPTTENLAPEKELEEALRLVVGIGLDPGIVRVNAPNEDNLFAIQGMRAKQDGPEPVGLFVVADGMGGHAHGQEASRMAVQTISDVVAPVILRSSEDENVFAELLKEGAHRANLAIYQRNRQQEHMMGTTMTAALVVGSIAYIINVGDSRTYLYRAHEGLRQVTRDHSIVARLVEDGVIAPDDIYTHPKRNQIYRCLGERASVEMDTFVEALQPGDVLVLCSDGLWEMVRDSDIHKIIAASAPHPSQISTMLIQAALARGGADNVSVVVICVLGAEK

>tr|A0A4P8ILN1|OS=Burkholderia sp. DHOD12

MRTKTLRRPVATATDTGCRSTARTRSTRSGDGMTATRGTNRWSVGQRSETGYVRTENQDRMSWVRTPVADVFIVLDGMGGHAGGGIAAELAVQVLQRHFETLTSLASVEAVLRSAFADANAAVYARSHSGDPATRGMGTTAVVMLIAGWRLMLAHVGDSRAYLLTRSGALQRLTKDHSRIQRLVDTGLLTDAQAATHPDAGILERAIGHAPQVEVEVGQWLRVRAGETCLLCSDGLCGYIGDADIAAIMRSDRTPQELADELVRLALERGGADNVTVQVVRRDRVHVLAPWRRLRARFTPGAAAIAAPAALVGVLGAGWLASLPTQLESERETAANTVALRSEVQQDRKVADERYESTKQELAAIRTRLDQLGGSGSRANASSPPVTPHAQKPPSATGKKEGAAPLAGRRHVGGPSHTARAQREAAVADAASAAGTQPASQPAPQPGPESAPHPPVADGPDPAGS

>tr|A0A4Q1KK02|OS=Sphingobium fluviale

MSQFNAFGSAKTIGQRKAQEDSIAWTEEFVDGAMGRPRLVVVADGMGGHAAGDVASELAVKSFITAYQRDTSGEATILREALDWANERLADFVARNSSADGMGCTVVALELNEEGSAFRWLSVGDSLLLSIRKEGISRINEDHSFREERRRTEETGGDVSAVPSANMLRSAVMGSHIPLVDDHTVWRRFSEDEILVLATDGIETIEMDRIAAIVRGSADAPSAAEALIAAVEQAQRPRQDNTTVAVIFGGSASIGDVAAGSANKKAERKSSPSGRRTSRQWQVGLLTGLLAGLALGTILGALGTWLILSPDKEKIEEAADNASVTGDTAKARVPIAGVPPVPQPAVTKADATTRLVDTPAVVEKPAPVVKRTERSNAGDKGEPLKPTLTGNGPATKSAQPAEPTPAPNPSKAGQGSSQDETS

>tr|A0A4Q2JX51|OS=Agromyces fucosus

MTRGAALIAHSARTDVGRVRSVNEDSFLAEAPVYLVADGMGGHARGDAASQAVIETFSRHLEFGVPSSPEQVLDAIHSSNEAVRSLSEAGEEGTAVAGTTLSGIALVDAGDGAGFHWMVFNIGDSRVYAWDGRTLEQLSVDHSAVQELVDAGLIQPEEAERHPERNVITRALGADEFVDPDVWLIPAAGRQVFLVCSDGLTKEVDDRTIAHILAGDTGHAAGLAGELVDLALAHGGRDNVTAIVVESVLGSDDDDDETTRDRRGRLSRAAEDTRPRTGGGGDGDVPA

>tr|A0A4Q2ZGD1|OS=Sphingobacteriales bacterium

MQLSFGNHTSIGHVRTANEDAFGNWLTPNGHLFVVCDGMGGHIGGAIASQSAVATIYQTMAAQPYPSAPVAIRHCLEQANKKLHTIVQGNPSLQGMGTTAVVVLVQGDTAFYGHIGDSRLYLYREGSLRRLTKDHSFVQSLVDQGHITAVEAEKHPRKNEIYRALGISETIDPTISQAAINLQPGDSLILCTDGVNGMIGDAAIASVLGETRLSTQQQAEKLVQLADKAGGHDNATVQIIQPQQKPVPAAVPPIATSAPVTTAASFPDEPVIDAPASKRPILPIVGGVVLFIALLLVVFSYFRETNAPKLGMEPTDTSTAVVPPPVIDTPMTQAPVPDPVPTPVATPSTKPPTKPAATDTAATSATPPATRVVTPATTTSAKPKTDTAARKTSAPDKAVAPKATTPSKPAVSKPAASPTPAAASPKPKTDTAKTRRAKAVMPESNPAP

>tr|A0A4Q4SB91|OS=Alternaria arborescens

MPSNFAQPTCPLNRDTSPATVGLRSFHSTSHNWRSTPQYTYHVAASYSAKQDRFNAEQNLFTQPVHDPSISNEDLRDCKESIDKRRRARSGQDAFFFSQVGNTKTTTFGVADGVGGWVESGLDPADFSHGLCEYMACAARSWPHGFNTTALHPKDLLQVAYDEVTDDDSIEGGGSTACLAVAEPDGNVEVANLGDSGFMHLGLNAVRHFTQPQTHAFNTPYQLSKTPQRMLVQMAVFGGPSTLSDLPKESSITHHKVRHGDVLVFATDGVWDNLSPQDALGIVSRHMVDLGAWVEKDGTIEVGQDLAKLVQADSARKADSSSLQAKVAVAIAKEAKVTGLNTRRDGPFAKEVQRYYPGENWHGGKPDDIAAVVAVVLEDAPQRAKL

>tr|A0A4Q5NS33|OS=bacterium

MGFFDRLKSKKDAPSEASTDPNAPPAEGTPSANTPPSTPLNMDEANRLMDSAAQSINQGARSLAESAKPLGESLKPLGHSLITELSSAGRDLSNTLQNLTTPSTPASGAPSAPVQPSAPVQPSAPVQPSAPEATVPPTAPPSQPPVPPTEPPVSSAATQETPAWGTTKEVEIPQPPVATPSPYTPQSDDALPLPDSYDPSGTFDPFAPSSDSTPTITPNASTSESNLYAPPTSAPIDETPIVISPAISPASASSFEQTAPSDFLDDEGHLEEYIEPLSSLAPSTLESGALVSLLANDGTPIDLTVTEVLVPRATVNCYRVQNAAGATLLLREAAHSTEAAARLERENTARRTINSPQIPPPAAFGLSGDRVYLADTTGDTQKTFASQLQEDVSLADLISTLTQTSAALSGLHRAGFVHGAMRPEAIVLGKPVRLSGFESLSPIGHKSAQTASFAGYGAPEIVAGEPLDARTDIYSVGALLHRIITGNGVSETGWDDTTFAPKILLPGAPQILAKTLGAPATRYSDMSALHRDLVRLRARLRPLARHQSVGETTIGLEWGRTTNQDAWGELRGHKQGEEGAIEWTAWVVSDGMGGMASGEVASQVAVGAVLKEAAAWGAAFNGETVAEADQAQIVKTWTREANRQAVEAMNNIGARGGCTLVCGLIVNKRLTLAHVGDCRAYLWRDGELTQLTRDHSYVMSLVLQGEITRDQMRDHPDRSKITRSLGDRHPMPDYFVDGLEVERKTPSIELQNHDVLLAFSDGVWEPVVEEEMKTILTSAPSLEEAAHQLVDAAMREGGPDNATVYLFKMGEAAPVVVHQES

>tr|A0A4Q5Z192|OS=Chitinophagaceae bacterium

MADNFFGITNTGKIRDNNEDAFIAEKLSNGWIIACVIDGVGGYEGGEVAAEIARTTIFQKLQQPSADVIQTMRESMVATNQNIYNEKLSGKGNNQMACVVTLAMVDVENNQFYYAHVGDTRLYLLRDQSLVKVTKDHSFVGFLEDSGRLSEKEAMSHPKRNEINKALGFDAQMDVKEDYIETGSSPFLPGDMLLLCSDGLTDLVNNETMSSILAASKTLADKGEALINAANNAGGKDNITVVLVKNDKKPLKQKATKPIAPKKNENNKEVTATSGNEAPAATVSNTPRKKNNTALWLLLLLTFLLAGVCYWLWSKNKELE

>tr|A0A4R2IMT6|OS=Kribbella antiqua

MTLSLDYAALSDVGRVRRNNEDSAYAGPHLLLLADGMGGAAAGEVASAAAVQVVRRLDKPGISGEDMIEALAGAVHRANERLSELVEEDQEREGMGTTVTALMFDGQQLGLAHLGDSRAYRMRDGQLYQLSHDHTFVQSLVDEGRISKEEAFTHPHRNLILRVLDGRPDSDPDLEILDVQAGDRLMLCSDGLPDYVSDDVIAASMAGGTPDSVVVELITHALEAGSNDNVTCVVADVVETPPTSGTTPQLVGAAAELAQGSTGRGDTTIAVPGGRGHGGGGGGAVLDPEELRYAPRAPKRFPWLRRIAVLLVILGLIGGGGWFAYSWTQKQYYVGTDGDYVAIFKGVDADIPGIALSKVYEQQTLQVDKLPTYSRQQVEGNIQADDLAGARAIVSELQRAADECAKPTPSPTPTTKPSTTPKPTAPATSRPATAKPPTTAPTTARPSTTPSSTPGSVDPNDCDGVR

>tr|A0A4R2KHC5|OS=Chromatocurvus halotolerans

MSAPSWRIEAAGATHPGQRSHNEDSWRLDNDSAFYMVADGVGGRDAGEVASRIACDVTVDQVRAGTGLAPALAVANAAIREAIAASGEGASSMASTAVALHLQPQARRFSLAWAGDSRLYLWDGRLKTLTRDHSLVESLLKRGEISRAEADDHPRRNVILTALGDTEAELETGENAGALTGSALFLLCSDGLSDVVPASVLCDIVCRDSDSLQTRADALVQAAVDAGGRDNITVVLVSWRDTAAAQASSSGVDGPVVYEAFDPVSGERIRNTEEDRAAPTIRRVPARSEQTGSGHTGSGHTGSGHTGHGRAAPSSDLSQSGEASRTDRWRWRWWLAAVALLAAGGIVADFVTRQGS

>tr|A0A4S3Q6H6|OS=Kocuria rosea

MAIAFRYAARSDVGRVRSKNDDSAYAGRHLAVVADGMGGHVGGDVASASAVMDLTSLDRPDHHGDGATVLTDEIQNANQNLAKLVLGNARLGGMGTTVTALLADQDELVVAHIGDSRAYRLRGDEFRQVTKDHTFVQRLIDEGRLRPDEAESHPHKNVLMRVLGDVDASPEIDVETLRPERGERWLLCSDGLNAVVRPETIHSVLGSTDDLNQIVDTLVELTLDRGAPDNVTVVVVQVFETDDTAPVPQAGEARGAVPDPEFARTSDEITGDAGTPGTAEAESSAAALRRDLAARPHLLVGAAANATQTGRIPTVSDSVLERRATLLQTSAPDTLADPRDVESALHRADATVPSPRRRRHRRAVAVLALATAGLLLVAGGWLTSGWISSQ

>tr|A0A4S4CYR2|OS=Camellia sinensis var. sinensis

MPSNYFSSLSTSVKREIQRATISGPEGGLQDLVGVFVGQGKLLVGSTRFFHSTPFSTLSDLHVLVQPGTVVAAQSNLHLVNRRRNLSFVGALSRTFSIPSVSGPSFQVCGYHVDRLLSESSHFPLGVESQKTPMALYGSKAVFGDCSLNNLTSKLGHLTVSTNNATTFYSNRSFDSCRKASMSLRNKEQPHNLFLYGYFIYNVTKRKGNSIPDLGFGLKVFHSSSSSCSSAGTAPDVSFDNSGREEQLPSAAVSSEQKDLVDRTLKLISGSCYLPHPDKEETGGEDAHFICVDEQAVGVADGVGGWADLGVDSGKFSRELMSNSVAAIQEEPKGSIDPARVLEKAHSSIKAKGSSTACIIALTNQGLHAINLGDSGFIVVRDGSTIFRSPVQQHDFNFTYQLESGNGGDMPSSGQVFTIPVAPGDVIIAGTDGLFDNLYNNEVTAVVVHAMRAGLGPQVTAQKIAALARQRAQDKNRQTPFSTAAQDAGFRYYGGKLDDITVIESSSPSPSDSGGGFLLPPAYWTGTVRNSDREDRAQSLVLSKIIYETYSIWVEESELQRFVVAQDCSTGFRKSLMPCLDGNNEQNRANLSWYAMLRSGSSRILYLQING

>tr|A0A4S4D1K2|OS=Camellia sinensis var. sinensis

MSWHEFQTCVNLDRMADDVLQLECSDGSTVFQVGDMSEVTINFGMEEPNDTDISTEVADTVMNIDIGVPSEDDGDNESNTSVAIDVTDCGMECNEVLLEDNRDFELSDIEELQIEVIDCSSSGFDFGQEKSVHALEGCMENERHEISSPVSVSQLGALNVIETMTVSNPIEAEPIPDEETSHDTLEELIDTNKSESTMKLESSDRFRSGGDVKEGLALQRGVIVRGEVHDGVPNVELEAQSSDLDGSEHRAVDLESETAVKREEISTAGYILSSGVYLLPHPSKASTGGDDAYFIVGRNWLGVADGVSAWSEKGINPGIYSRELMENCRKIVSECDSMPLINPEEVISEGALEAESPGSSTILVAYFDGQVLHVANIGDSGFIVIRNGVVYKKSTPRSFEFNCPFQIASGVDPSEYVDTYIIDLDEGDVIVTSTDGLFDNLYDPEIVSIVSKSMQSNFKPEEIAEALARRAHEIGNSTTCMTPFADAVATFEFDEGFTGGKLDDVVVVVSLVQKISSPHME

>tr|A0A4S8IMY5|OS=Musa balbisiana

MAEILLAGHLDLRLGPASFKLSPHPPTPLPLGRLLSRPPSLPLPRSRTVTKPLSRSFSEIRSVTECSDGSTIFRFGDASEVKRDEVAAFGNCVLEKDVENSGQANAKDPTENDSCEMSQETPKRSDLEMKGLEPVVIGDLTAEGVNSLGRTYREPVEMVPKIEAGICLEETAVGSEEKITVTEVEKPEATRESCGNGELKGLAAQLGQESCISAIDDVGSLIEDEEEDVNSVDVGNKVDEAKCCVEWTSILIGDASGNDDKVVRDIDSDIHVSPEDQVVQSLDEDSDSALRASVRSVSLAKCMDMEVNSVSETSQLSSSSCMDESEGESSYETQVEKAPEYSEGRTSDMDIDFGDNSLDEMTFEATKEHKESRSPDMDDKVKDVHGDGMLQRSGSFENLEIPVHETIQIMEAQFEDIGASQVSRAAQDITDVDVELISSYADDHGDSHTVESLVKAETIDGMRQDENAEVAGYNRVDQVPAHLLSLSSGAAILPHPSKALTGGEDAYFVALNNWFGVADGVGQWSLEGISAGLYARELMENCERFVSKYEGTKPDEILTKAAAEASSPGSSTILVGYFDGQVLHVANIGDSGFIVIRNDTVFKRSTPMVYGFNFPFQIQRGDDPSRYIEMYKIDLHEEDVVVTATDGLFDNLYEHEVADIVSKSLQASLKPREIAEILATMAQELGRSASARSPFADAALAAGYPGFTGGKLDDVTVIVSIVEGSSR

>tr|A0A4S8J166|OS=Musa balbisiana

MGRLSIPHPHRLLLPSPSRLHPSSDASTIPRRSRFSPSAHRQIRSELSLSCGSHLIPHPAKVDKGGEDAFFVTNHNGGVLAVADGVSGWAEQSIDPALFSQELMANASGLVVDEEVSYDPQALMRKAHAATSSVGSATVIIAMLEKDGTLKVANVGDCGLRLVRRGQVVFATSPQEHYFDCPYQLSSEKIGQTYQDAMVCAVELMEGDMVVMGSDGLFDNVFDHEIISIVSGSPEVAEAAKILANLASNHSKDPNFDSPYSMEARSRVRKYFQTSFLFYVGIVYKGPNDITVIVGRIISSSDSREVGYETT

>tr|A0A4U1H329|OS=Polyangium aurulentum

MSDQAARETPSEVPRAAEIPATPPESSRKPRGGDIRLRVFGRTDVGQVREHNEDNFIVADLTKASRGLMETDRNQVIGDRGVLMGVCDGMGGAAAGEVASQLAVDIIYQKMVGGDTPRDHDEIAARLVQAIEAAGLRIFSEAKLDRTRRGMGTTSTIAAMLDDHLFIGQVGDSRAYILRGDRLVQVTRDQSLVNQLIEAGQLTEEEAETFEHNNIILQALGTSDSVQVDLTFVELKKGDTLLLCSDGLSGMVRNEEIREVLRSVDEPLEACKTLTDRANQAGGHDNITVVVAKFEGDALSDPTDEEIQGLRYRKYQLPEPLPSSPVAAPDPSKRVKPDDQAKISVPLPSHGSHAGAGSSSASEGSPRPPESARRDREDSHPDGAEPPTSLARRSLGSEDPIHIPTDGAPQWLIVMMIASALACITIAGYYLLR

>tr|A0A4V3SA29|OS=Temnothorax longispinosus

MQSIYWTGRLLSRALWNGIASYSTACGGEQQPSAAVASTRRRETSLVSAVCGFPKDFARSRIRRGQFGDDAWFTARFRTAEVIGGRLPATPAGSTPRDRLARAGALLFPSRPISRFLSLSCSGIPVQKPEMKQSVADGVGGWRHYGIDPGEFSNFLMRTCERLVSMGRFTPTEPAGLLARSYYELLENKQPILGSSTACVIVLNKETSCIYAANIGDSGFVVVRRGEVVHRSSEQQHYFNTPFQLSLPPPGHSDLVLRDSPESADTSSFGVEDGDVILLATDGVFDNVPDQLLVTEMRKIEGERDPTKIQCVANTIAWMARRLAFDGAFMSPFAQNARENGIDAIGGKPDDITVLLATVAI

>tr|A0A4Y7J3R7|OS=Papaver somniferum

MLLHGTTSPEGLAINVGRKLANLRSFCEFRGNLYWHGQIRATSVITGFKIRLGSFNPGQLGGKSLSGVSRLISSVGDVQSFLRSSSTSSITKSGSVIVHEWRDSFVSRSFSTSGSSYQLGKYNIDRLLSNSPFQEVPMAAAASGSKALLSDCCLDGFTLKNGHVSNSAKQVCFSFNKKSLYSGSRASMSLKNRKPSNDNLHCGYFMFDAMRKSCDYNPLIGGCRRLHTSSAAFVSVGASPDSSVHGSGCDQQLVTPTVSSDQKSLGDKTLKLVSGSCYLPHPDKVDTGGEDAHFICIDEQATGVADGVGGWAEVGINAGLYAQELMSNSVIAIVNEPKGSIDPARVLEKAHSSTKAQGSSTACIIALTNEQGIHAINLGDSGFIVVRDGSTVYRSPVQQHGFNFPYQLECGRNGDLPSSGQVLTVPVAPGDVIVAGTDGLFDNLFNSEITDIIVQAVRAGLEPQGAAEKIAALARQRALDEKRTSPFSKAAREAGFQYYGGKLDDVTVVVSYITNSTTTNDFVFEPTD

>tr|A0A507C6C5|OS=Synchytrium microbalum

MTSTTVAAKRVAKRLAGSQHSLLLRSQPSSKNGLAITTGSLVNASASTQSSVATASSSSTVSHSSLKQSTSSFRRSGAAATTGSTTSNNANNASTVATGPTNAPSRSTSPLPSSASTSASRKNNIHAASKPVLDRLQTTGTTSTSTNPSSPTQPTNNSFSQHRKWSATSASTYHTFAHPVTPASSATSSSSSASFGSTSSSSSSSAAPCSASTSLKSSYHTRAIALPVSAIGNNNDGVPWFDKQSASSIFELFEKVKAIPQTRRSFRFTHAASAMPKASNQTVINDGRFLSVGAGEDAFFSRHDSMGVADGVGGWSHVKGANPALYSRKLMHYASLELEKYDDIANNDFDMEEYYNVDPKVILQKSYDAVQSDAPKEGLVGSTTALIVVLRDDELRITNLGDCGVMIIRDNESIFRTEEQQHSFNFPFQLGTGSHDTPDSAQSFQIKVQEGDIVIIGSDGMFDNVFDEEVVDIVRSVTDRVGVAKDGKLRIRDVDPQRITDALISRAREVAEDTRFAASPFQSRAIQEGLYYQGGKLDDISVLAGVIRLSEDSPDRR

>tr|A0A507DFJ9|OS=Synchytrium endobioticum

MASTTTAAAKRVARRWADRELSTLLLSSSTKAPLATTPQVTAASTTAAAAAATATTSTISTQLPSSAAIPFSYRWATAASASASSTSSATSSSLYTFVAPNTVSTSLPTAQASSSSSSASFGSSTSSYAAACSTTFKASYHTGSISLPVTSFGNNTDRVPWLDKQPASSIFELFEKVKSIPQTRRTFRFTHAASAMPKSTHQAVTNDNRYQSVGAGEDAFFSRHDSMGVADGVGGWSHVKGANPALYSRKLMHYASQALEKYDDIANNEFDMEEYYNVDPRIILQKSYDAVSEDAGKEGLVGSSTAVIVVLRDDELRITNLGDCGVMVIRDNESIFRTEEQQHSFNFPFQLGTGSHDTPDDAQSFKVKVQEGDIVIVGSDGMFDNVFDEDVVDIVRSVTDKISVKHHGNSVQGANSRLRLRDIDPQRITDALISRAREVAEDTRFAASPFQSRAIQEGLYYQGGKLDDISVLAGVVRLSEDSPDRR

>tr|A0A512AWT8|OS=Adhaeribacter aerolatus

MIIEIFATTDNGQRYGQNEDNFAVCKDLANKNWGFKPQEKITLSEQGAVMLVADGMGGANAGEVASDIAQKVVQEQFNQLTEVPGTDKEKVSFLKKVIQAAHEAIVAHQVDNLETAGMGTTIVLLWTIPDKAYIAWCGDSRLYIHKKGIPLKPLTDDHSYVWELVKQGKITPKEARLHPESNIITQSLGDPTTPPKTETKIIDMQEGDRFLLCSDGLNSMLDDEEIEAVLTQEEDTVEASKRLIAEANAAGGHDNITVILFDVHEATAKQVAIPARTTTQELRNFIRSKNRVIAVLSFLLVCVLGYVVAGYIPKKSSNEPIAKQPLSNAPVIVAAEAKQDSLTMPVAETATAAKPEENNDVVAPTVKPPVTSPQKNIKDKISPNKLEIENKSRIDSVAEKKSTPITPPKVDQKDSTEKSSPEKLEKDKKLTADTSGKNNNPVPSEAKKDTTKSQ

>tr|A0A516PUU8|OS=Microlunatus elymi

MTERSSGAQPLAWEYLAHSEIGLVRKNNQDSGLVSARLLMVADGMGGAAAGDLASAVAVECVAASEREALAEADSAAAGAQSEQATDPAVEAGDGPDRLGRLSAAISAANTRIADLVATDYALEGMGTTVTAAVLDGSSVGVAHIGDSRGYLFHHGELSRLTHDHSWVQSLIDDGKINEEEAATHPHRSLLLKVLNGQPANEPDLSTVPVQPGDRLLLCSDGLCGFVEDDLIAEALAGTSRDEAMDRLVAAAHRAGGLDNITIIIADVIEALGDDAEATAIHPRIELDQSDVDQSDSDQADVDQSGGTTHRVAGPIDALQIIGAAAERDIPAYESEIRERVAQRSVDAGDDGDDDDDYDDDEGYDGDDEDRYDPQPPSKRRWRRPLILTLAVILIIAAAAAAGLGWIRTQYYVGAITSNVAIYQGLNESVLGVQLSRVYEVQDLPLDDLPPYYQDMVRQTIDTGTLDSARATVQQLQDTADRCAASQTTPGSSTSPSTKSSPTSSKSPSGKETSSKPKPGGKTSSPSESATTKTSSAPKTSSAPASPSSTSSSTTQGQLPGGDKC

>tr|A0A517NW69|OS=Planctomycetes bacterium K23_9

MSKMKPLSMKEASQSPRVEVRDGGVENGKKSSAEDENADVFRVDAQHLNFQRAQVRRPGLGKMDCYAISDLGRKRRNNEDQFMVADLAKSSKLWTATRRSLGCEAISDKSRAKMMLVADGMGGHAEGQRASKLAAERMLRYVVENLAWDPLSPAQLKKPTHTFQEDLNDGLVSAVSHCQKSIIDEAAWIPSRRGMGTTLTATLLNWPMLHIAHVGDSRCYVCRDRELVQLTRDHTYAQAMVDAGEMTKEDAVRSQFANCLWNVVGGNNRTVEPDVSTHELQIGDTLLVCSDGLTNHVSDELINEIICNSTSARAACQSLVRVANEMGGKDNITAIVARFIDSETFDSDSHEFGISDEQMTDDIDLLAYETEFTLSESTIESMEDDQA

>sp|P9WHW5|OS=Mycobacterium tuberculosis (strain ATCC 25618 / H37Rv)

MARVTLVLRYAARSDRGLVRANNEDSVYAGARLLALADGMGGHAAGEVASQLVIAALAHLDDDEPGGDLLAKLDAAVRAGNSAIAAQVEMEPDLEGMGTTLTAILFAGNRLGLVHIGDSRGYLLRDGELTQITKDDTFVQTLVDEGRITPEEAHSHPQRSLIMRALTGHEVEPTLTMREARAGDRYLLCSDGLSDPVSDETILEALQIPEVAESAHRLIELALRGGGPDNVTVVVADVVDYDYGQTQPILAGAVSGDDDQLTLPNTAAGRASAISQRKEIVKRVPPQADTFSRPRWSGRRLAFVVALVTVLMTAGLLIGRAIIRSNYYVADYAGSVSIMRGIQGSLLGMSLHQPYLMGCLSPRNELSQISYGQSGGPLDCHLMKLEDLRPPERAQVRAGLPAGTLDDAIGQLRELAANSLLPPCPAPRATSPPGRPAPPTTSETTEPNVTSSPASPSPTTSAPAPTGTTPAIPTSASPAAPASPPTPWPVTSSPTMAALPPPPPQPGIDCRAAA

>tr|A0A522QQX9|OS=bacterium

MGRRCGKPEWYLRQRAADRASDGAAARRSHRDGRFGRGVRGDGTVEVAVSSELGSRRRNNDDAYVSEGITPGATLLAVADGFGRIASGGAGPAALSLALVREYLRRHMRGSYLSRSLSIPELRQTLLAALRHANGRLYAQSGSHEDFVAGGAALTLVLIVGSHAYVAHAGDARAYILRGNSLRRLTVDDSLFADLVPHGGAGEQAPCALLTRTLGTQPALEASVVHFELTPDDQLLLCTDGIHKFVSEEEIRAALQGHANAADAVHRLAVLARSRGGADNGTALLGRQLNLHTQPSAQGPQPLSFNLSRVMASLALIWLLLLTVISFRLLSSHDYFTVVHGHVALAQGYPTDIFGVHAWRVLKVYGVQADRLQAQTLTTLEAASVGSEEQADRIVERLTAK

>tr|A0A523ITT0|OS=Caldithrix sp

MYSYSYDQNGVMQKKSAMKENKKTQITAGAHTDVGNLRASNEDSFYISEDENLIIVCDGMGGQIAGGLASKIAVETVKDVYLNVEQDKLDKLFPDLEGSLTGTARRLVAAVRLANRRIFKTAVKFPKLRGMGTTVAAITFDSNLATMVHVGDSRILRLSDNKILQLTEDHSWLNELIADDEIDEEDIETFSKKNVITRALGSSPAIKIDIHCEKYKKDDMYILATDGMHNAIPQEEIKKLIYKSKNGILQKVAKFLIETAKAIDGSDNITVALAKVTKDSSNKAVVGTSKTIFEEDDKTRARENKFIVERYGEPQMAWGRFKISVKTPQRLFFASLVIFFLTLGIYFTQVRSFSNPKTPPVETKQNESPRKNKINNVAASTPPARRTKIPQLVIQRSKVNENAVMAFIFFNSKEDFENAKLRQRGTLLNTFQPYLRKDEAASEDLSIFLFDESGNVVQKSNFQLPEIQDD

>tr|A0A524H516|OS=Gemmatimonadales bacterium

MTISPVHVSVAGLTDVGRTRDHNEDTYLIADLSTGELCDATEVQVCPVGPRGSLFMVADGMGGAAAGELASALGAETIHNHLMRTWAADEDSSGERFAFRMREAVEEANRKIHEHALENPEVKGMGTTVTAAGVYGTDLYLAQIGDSRAYMVRRGVATQLTKDQSLTQRLVDAGEMTEEQAEKSERKNIILQALGPDPDVRVDLTYEELRRDDLLILCSDGLSGQVRREEFAEYAERFPDLAKLSSALIELANTRGGPDNITVVAARFEGEGLRVGTTEQEPGHHVFALPGDEPLENDIRVGFQTGGIRIDRSLQIPVPAEPPARPLRPRPPVGIIGTAVLLVAALLYYLITR

>tr|A0A524LRQ1|OS=Calditrichales bacterium

MSISRNKNIRINYYGLSDVGIVRSDNQDSFGQFPGGVLDSSESRGLLFIVADGMGGHANGKEASSMAVQITSDEYYIAPPGEDVAKSLSHAVQSANAAVFQKSSGPSDGGQMGSTLSALVLHSDIAHIAHVGDSRIYRIRDNKLLQLTTDHTKVEELKRAGILNTEDAKNHPEKSVLNRALGVKEKVLIDLISDIILRKDDIFILCTDGLAAVTKEELIKIAVSETPRDACQKLIALANERGGADNSTVQVLKIESEPQVKTAKKRTDTPILRPLLLSMTFLLFLFSMVTIYIAFDYRKAVSRTIQTVTSEDRQNDSEQQSLYNRAQRYFEQNRYEEAIQAYQKLLIINPLDEDVIKALNNIAARFKIEGEKYE

>tr|A0A524MSW1|OS=Lentisphaerales bacterium

MSFAHLHSAEATDVGKKRRNNEDAVLRLPGSGVFCVADGMGGFEHGEMASGAIIDALEEALSGRHIPGNAAAPGLDQTVHLINEAALNANHLIREYCDQQNISGSGSTIVVLAFDPNNPRRAKVLHAGDSRAYRLRGLALDRLTKDHSLAAAAGVEDTRQLNRMFSAMITRAVGINKDISLETSTVDVEQGDLFMLCSDGLTSVIDSTALQEILAQNRESAIESLAQILIDAANEAGGPDNISVVLIRVGALPVSATPPEADTAEREGAAASSSDEDLTPDEAHTDEPPAVQLPDTDEPRRFDSDQPLPTSDLECVTPSVGSTPDAEPQPAIEPRSAISGPEYPRSQGRTKLLTALAVAIALAAMVVILVWSPFRRSGGHTDNSLPVDKEVLPDAEMEALIARTLNDDLSQPDTQLADIPETGPLLSQTEIENMRKMLPTKIEATLLTGEWSHL

>tr|A0A524N3B9|OS=Lentisphaerales bacterium

MTQLRFPHLSVAALTDVGRKRKNNEDALLALPEQGVFCVSDGMGGSDAGEVASSATVEHIAAAFTGPASREWRAASQKAGVVRTALEDAGRWIWRHAIEKGMRGCGTTSVVLVFDELKSNRALSLNAGDSRAYRMRDQRLKQITDDHSFAAATGVKDETLLPARFRSIITRAVGITETIVLDETPVEVLDGDVFMLCSDGLTRMVRDERICEILETCAVQGPAQVAKALIDEANGAGGNDNISVIVIYVHGIPVVDYDAEEMTSTRTFADVAGDPAATTALESSVADDAVSVPSGRTRESTALSAGALDWAAGPSTAESAIRLPVAKRSKQSRFVLALFVIVAIVAGALYAMELVLAALDRLAAKRKADVNTEMQVPESHGEALAPAVEPDSTQWEDGLTTPEPEDDGPEWDGAAAPEEVNPDAMDTLPGNDLQEAASDAVPADGDIDAGWGEAEL

>tr|A0A524PJR7|OS=Anaerolineales bacterium

MSICIRRCASPEGDPPDASRRADRIIQGQCILIPHDTSFFSTAAATHPGNRRPNNEDRFHVGTYQLERDQTIVTLAIVADGIGGHQAGEVAAQLTIDTIIHQLLSYRGGDPLPELQSAIIEAGRVVSAAAHAQEERAGMGSTVALAMVIGSRLYTAHVGDSRIYLLRGGRLQQISTDHTWVQEAVEYDIIAPDEVRGHPHAHILRRHIGGDQLPEPDTRMRLKDGESDRQSLAHQGTALRKNDRILLCSDGLTDMVEDMEIYQGLSGYEPQQGVEFLTQQALERGGDDNITILILAAPHSRPTGARTRRQRRLMNTMVAIFVLLALITIGVFAAMWFGWWPWL

>tr|A0A525C028|OS=Desulfovibrio sp

MKIIPGNGQFIGARKEQQDSIAFSDVREERFSAHGGVIAILADGMGGLAMGREASQTAVDSALMAYGAKTPDEAIFSALNRALVQANQAVFDLAVQAGQAGDVGTTLLAAVIHQGKLYWASVGDSRLYLFRNGRLFQMTTDHAYAKELERDVALGYMTREEAEHHPDRNALTSFVGLPELMDVDRNLRPYPLREGDRVMLVSDGVYGALAEDEMAGLLAGDPQQAAEALIKAVYNKDLPYQDNSTVAVFAVEPDNTPFTGVTDRPEESLTVRMEREEKPQEQADRTVRLADSSGTADGPKRRSLWVVLVVVSVLATLAALYVILGTDIIFPPEPEPAAANATLEHEPSGPPPAEPESAIPEPDVLEPDVPESEPTEPAPMEPGPSEPGDRPSMQENATQPGVTLPGPESEPSREPHQAPEQEPGEANATQEPLRPPGAGLEYGQDEPGQEPEATPDADLDQEPEPGSGLEYGLDDAEQDDAQQDGASPDQGNGSSPQYQDPQPLPGDFGHEGDQDREGPRGEDKDLPQDSEPESGQDGSRAPEPTLPETQGSQESERGEPETVEPPPPSVYPEGGRPEPSQQETNATEPDAQGQDAEPEGSGSGSGNEEPGTEEPGKDERLLLNDSVEQDGISHVQRDRIVIRAAFSGQENTL

>tr|A0A532UPQ0|OS=candidate division LCP-89 bacterium B3_LCP

MEIRSYGFSTAGRRPENEDAYLHKVFKRSRGCLHAVVAICDGMGGQSGGSIASRLAIDEIDKAVKSPPLNNKAIRSWITHTTESIQKRLIAYCVTHPELSDMGTTLVLAAVSEESVWIANIGDSRAYQIDELSTTQLTVDHTAIQDGIDRGIYTLENVSSSAELRSLATALVRNLGEGGNSQPDIIEIPLSYSQVYLLCSDGLVGSMVNPLVFDHDMENHIFGTTDLETATDNLISIAYQRGSTDNITIVLIEIGNLPRRSILLPDETDIDVMRSRDFTTVIPAKRRLKRRSNTFLILNLIVAVILIAAIVIVIDRENIFPQREPANDMLATESPVSADTGTDGILISKTESTHDEEPDRVDGAELSSAVKPSEADNLNSVESAQDTIVFKLGDAWPGSTNVILLVTRIPIGTSNLSDAQWELQLIVRMGDKEYQSNIEKINTTE

>tr|A0A535DM32|OS=Chloroflexota bacterium

MARRTAARTDTGRIREGNEDRYLVRDDRDVTLMAVADGVGGGPGGEVAASAAVDELAARFFAAPRAQPLAERLGDAVRDANTAVLRAADTSGNASAASTLVAAAVAKGQAVIANLGDSRAYLVRDGASRQVTEDHSGIPAHGITRFVGDPRGVQADVYVEELRPGDRLVLCSDGLTRHVEPEEIALVVGTSGDDLDSAAQRLVELANSRGGEDNVTVIVHSALPFGRAARAPRRIVGLAMFIVIVLLVVSGAIAVLFAVAPGQPPSITASPSPSATASPSPSASASPSPSPSATPTETPSESPTPSASP

>tr|A0A535UF12|OS=Chloroflexota bacterium

MATKQLRLEVAQLTDVGRKREHNEDNMAYVIPKDPQVMAKKGALFIVADGMGGHAAGEVASEIAVDTVSNVYYQDDSDDVAYSLLHAIKRANALIHQRAAENMLRSGMGTTCVAAVLRGNMAYIANVGDSRAYLVRDGQVKQVSQDHSWVAEQVRAGLLTEDQARTHAQRNVITRCLGTQADVEIDVFPEQLEDKDALVLCTDGLSGLVSDDEIRRIVDQSGPQDSVYHLVQRANENGGPDNITAIVVSVQETGWEPPNVRRPVPVGGREVGEDTASLGSPGSSPTTVPSAVIGDGSAPSSSMRFSSGPLTHDDIDTAPQPVLAPQRRNRSRLFYPMLALLALLILAMVGGGIYIFLQFNSGTSVAAQVKNAQDSMSHANKVISTNPSAALTDLANAQKSLRTLQQNTTLTNDQHTTVSNLEGKLTRSVQMAIANYNIQSSITLLCPFNATTNAINDGTTGTQAKTIVQVK

>tr|A0A536A561|OS=Chloroflexota bacterium

MVVPALRQHRRASRGARGAPRDGALLRVVPEGPPVRGAGGDRRKLARGARGAKRRRHASGRRDRRAQRRRVCPGGLGCRAGGQRRGVQRRKTGSPGRDGHHPEPDGDVVLRRGEGLHEEGGLALPVRSAARSDRGRVRDTNEDRYLVRDERRTTLLAVADGVGGEAGGEMASATALEGLAKRFFDASAKIPRADALATAMREANDAVLARAGESGQRGAASTLVAAAVAGKDAVIGNLGDSRAYLARDGGVRRLTADHSGAMPSSITRFVGDPRGVQPDVFVETLRPGDRLLLCSDGLTRHVNDDEIAARVKDRDLAKSVNALVDLANERGGQDNVTVVLYAAGGGSALAAIGRAFTSAILALLAAVVIGGAIGALLFASGAVPPAP

>tr|A0A536F2J0|OS=Chloroflexota bacterium

MVVPALRQHRRASRGARGAPRDGALLRVVPEGPAVRGAGGDRRKLARGARGAKRRRHASGRRDRRAQRRRVCPGGLGCRAGGERRGVQRRKTGSPGRDGHHPEPDGDVVLRRGEGLHEEGGLALPVRSAARSDRGRVRDTNEDRYLVRDERRTTLLAVADGVGGEAGGEMASATALEGLAKRFFDASAKIPRADALATAMREANDAVLARAGESGQRGAASTLVAAAVAGKDAVIGNLGDSRAYLARDGGVRRLTADHSGAMPSSITRFVGDPRGVQPDVFVETLRPGDRLLLCSDGLTRHVNDDEIAARVKDRDLAKSVNALVDLANERGGQDNVTVVLYAAGGGSALAAIGRAFTSAILALLAAVVIGGAVGALLFASGAVPPAP

>tr|A0A536GSI8|OS=Chloroflexota bacterium

KAEEVEAHRIAEREARRIAETGSQRPYAQPAGAGTTAPLRSPKSDLRCPNCHEAIEETDTYCPNCRYLLSPTASGLHRFAAPPPAAPQVQSQPQGAYAVPPVAPPQAQPQPQGAYAAPPPPSAQSISEYPTVEMSPRNGSQESERTLPYNVQQLQGRNLSLAVGYESDPGIKRKHKPNEDSLFALQGARTHDSQPEQFGLFVVADGMGGHANGQDASRLAIQNMIDFMLPRISTVTVTDDDAYLKLLEEGVQHANQAVHQRNMEERADMGTTMTAALVIGATAYVANVGDSRTYLYREPQGLSKITHDHSVVASLVDAGIIKPDDVYTHPKRNQIYRSLGEKQVVEVDSFKVPLQVGDKLLLCSDGLWDMVRDPEIQRVISSPAPDPTQTGKDLIDAALKGGGEENVSVIVVSITEASGHTGMTGIQLLAKPESVTVPDLPPM

>tr|A0A538QWK8|OS=Deltaproteobacteria bacterium

MTAAATRCTIATDVDGANAGPVLRGVAPEGARAAARAPRDARSVARPARRPDRARTDRCGARPPGQLHCHGRFRAASWISTHLPRDARRRGPELGVGARDARRDRRHCGAGRAHPRDRDHGARGQRPDRGGARAADRPARRAGQARARGCAVSEGGSRSGDGPGRGQPRGPTEPAAPRFAGDSHVGKVRTTNEDTLILEPGLGLYAVLDGMGGASAGDVASQLARDAIRDFVLHRRPTLAPRALIEEAIAGATAAVFGEAQRHRERHGMGTTVVACLVIDLQHVVIAHVGDSRAYLWRDGRLQALTRDHTIVEELVGRGLLSPEEAERHPHKNVLSRNLGGKPETRVDSLELELRPGDRLLLCSDGLHGYASAEAIQYLLGSGDAPEHVARDLIELALRGGGGDNVTVVVIEGPPAAPSSTQVVRTSGALAWWQNRQRFLQVASDRGLTSNPIVRGLPPGEALELVALSLCQAIYHDLEKSTGVNVWTFAQNLAAGWFERGGDWRAVRGIIDVLGTSARVVVDDIRAADATLGFLLDVAVSRALIVVELVDAELIALHTAAQDTALDTAQDTIEQTTEESIELPERASERFIDRPTIPFLRPDRPITSTGESAELLEAIGRVITLARGRVAPRAVLLGQVLIALESVAGDSAGNFAAAVLAARELYGVRSADNAGVMPLHDALDQARILVAASVQQLRAQPAIRVRVLRALSTAHQRLVGATTGLVLEAVNPITDRLREVQAVTAELRDEVARAERRRADLERKFATIVDPSLRWGARGSSEWYPEPGWRSIVRL

>tr|A0A538RQQ2|OS=Planctomycetota bacterium

KERYVLAERLGARGEIIRFRGLDHGTGSLEPVPVVIVRSPFTPPAETVPAAEEVPAASAESTVAPDAPAQAEAPSVEAVPVEPPWPSSAWERALLEKVSHSSLPRVLDQFTQDGFEYLVEELPIGQVLWDAWDDPQASAEQRFGWLKQIAGALHHLHQGGAILEGLRPDIVVVVNGEHARLTDLADLLPLPLPPNPPIRATHYTAPELVLNSDLATARSDLYSFGAMLFALHLGRELTELDFEMQGVPKSIYQRFPDIHPLFGRLVSKTFCRELSQRLPTEDAAGEDATGFAELIRTLETCRRTLDQARLDIASWTTTGMVRTGNEDAFALLHATDARENGLNEAALVLLADGMGGYEAGEVAAAMAIRAMRQYLAQQRPFAPLAEDAQPAQPEAQAEPMAPAPDRELWKQHVVAALREANQKVFEASRDAGRHNMGCTAEVVYAAGRDLIVGHVGDSRTYHLQAGNLVQMTRDQTWVNRMVEIGAFTPEEAEQHPRRNELQQAIGGHPEVEPAVYEASLKPGDWVIVCSDGLTNHLPPDTIKEILQSSASAEAAARRLINLTNLAGATDNATVVAIRAT

>tr|A0A540KIR0|OS=Malus baccata

MKLNSGGGQETTTTTRTTTDETQVSNDIHAQAEENAVDAVSVTISNNATSGGGEETTTTITTTATTTTTSDETQVSPVLAENAPSTNPEDEFTEGKADTTDVSGISTSRIVEFNTVEGASDGALGQLTRSFFHREDVSAAVLQLSSSAALLPHPSKALTGGEDAYFVGCQNWLGVADGVGQWSLEGVNPGLYARELMENCERFISNCKGLPLTEPEEVLIKGSSYTKSPGSSTVLVAYFDGQALHVANIGNSGFIVIRNGAVFKKSSPMVHEFNFPVRVERGDDPSELIEKYRIDLDEGDVVVTATDGLFDNLYEREITSIVLKSLQTGLELQVHSLNVFLIYFFPVSFL

>tr|A0A540LZK6|OS=Malus baccata

MGNPFVRSGLSFSAMFPTHILPPLKPLHETVLTRIPIGFPKLRPTLPPHSSSSSSSSSSSSNFDIISTSELRDGSFLFRFGDESEKSVATETEIDARSHLEDPVQDSELSQTLLGSTGSGAGGGAGAEENVIDAVSMTSTDNSTQGGVGKEATTTTTITITTTADETQVSNITHAYPAIGGATEAENAVNVVSVTSTGSATAAGGGGEETTIRTTTTTTADQTQVSNEIHAFGASGCDGAAKAEENAVDAVSVTSTEDATGGSGGEQTTTASTTTADETQVSFVLAVNAPSTNSEEEFAEGEAQRSDVSGVSTSKIVEFHTVEGASDGEDVPAAVLQLSSSAALLPHPSKALTGGEDAYFVAGQNWLGVADGVGQWSLEGVNPGLYARELMENCERFISDCKGLPLTEPEEVLIRGSSSTKSPGSSTVLVAYFDGQALHVANIGNSGFIVIRNGAVFEKSSPMVHEFNFPVRVESGDDPSKLLEKYQIDLDVGDVVVTATDGLFNNLYEQEITSVVSKSLQTGLEIQDIAEVLATSAQELGQSKSTRSPFADAAKAHGYAGYSGGKLDDVVVILSCVEKKPIDIRLNKVE

>tr|A0A565CXP4|OS=Arabis nemorensis

MSATALSRLNPVSQFGFQKIVSGKSKSFFSIPGERRLFSDSSRFRLAGKRQAMAASGSLPVFGDACLDDLVTTCANGLDFTTKRSSGGGGFTVNCPVASMRLGKREGLMKKNRLVCHYSAVDPLEKSRALFGILSKSVHTSCPVCFSLGPAHELSSLNGGSQDSDLSGSQPTTTSLKSLKLVSGSCYLPHPEKEATGGEDAHFICDEEQAXXXXXXXXXXXXXXXXSGLFSRELMSYSVSAIREQYKGSSIDPLMVLEKAHSQTRAKGSSTACIIALTDKGLHAINLGDSGFTVVRDGTTVFQSPVQQHGFNFTYQLESGNSGDMPNSGQVFMIDVESGDVIVAGTDGVYDNLYNEDITGVVVSSVRAGLDPKATAKKIADLARQRALDRKRQSPFSAAAHEAGFRYYGGKLDDITAVVSYVTPA

>sp|Q93V88|OS=Arabidopsis thaliana

MADHLILSLQAPPFLIFPCSLHRSWRFPGGIRYSVPEFRLSSQLQLANSISPSKSSASSSSPPENSAPEKFDLVSSTQLKDGSHVFRFGDASEIEKYLEAEEKARCVEVETQNAKIAEEASEVSRKQKKLVSSIIETSTEKEETAAPSDLSNVIKIKDRKRVRSPTKKKKETVNVSRSEDKIDAKSASVSNLSSIVSVAEAIPISSTEEEAVVEKEITAKSYNVEPLSSEAMKKVSVNKIGDCETNGYQENRMEVQARPSLSTQQEITPVSTIEIDDNLDVTEKPIEAEENLVAEPTATDDLSPDELLSTSEATHRSVDEIAQKPVIDTSEENLLNTFEAEENPVVEPTATAAVSSDELISTSEATRHSVDEIAQKPIIDTSEKNPMETFVEPEAVHSSVDESTEKLVVVTSDVENDGENVASTTEDEITVRDTITDSGSISNNDDTKVEDLQLPVPETASLEPIKAASGREELVSKAFYLDSGFASLQSPFKALAGREDAYFISHHNWIGIADGVSQWSFEGINKGMYAQELMSNCEKIISNETAKISDPVQVLHRSVNETKSSGSSTALIAHLDNNELHIANIGDSGFMVIRDGTVLQNSSPMFHHFCFPLHITQGCDVLKLAEVYHVNLEEGDVVIAATDGLFDNLYEKEIVSIVCGSLKQSLEPQKIAELVAAKAQEVGRSKTERTPFADAAKEEGYNGHKGGKLDAVTVIISFVKIVST

>tr|A0A5B7E5R4|OS=Portunus trituberculatus

MLGICINEGFASPGSSTACVVVLQGDAGHLYSANIGDSGFVVVRNGSVVHRSQEQQHYFNTPFQLSLPPPGTNAQVLSDRPESAETLEFLVKEGDMLMVATDGVFDNLPDSLIVKEMVKVQGSTDLLLLQQAANSLAQQARKLAFDEDYMSPFARSARENGINAIARRNNGVGVHLYAASPSATMTGRSFSPSLEMSRTRNLGKFTINLRFTSDMHFFMSS

>tr|A0A5C5WT36|OS=Rubripirellula amarantea

MNNIENSSGGSRNLNDSKDSLAGGLTSHSCGMTHQGRVRENNQDQFLIAQLNKSMRVTATSLLLEDRLFGQVQGEVLLVADGMGGHAAGEHASRIAIDQLVTNLLGSIHWHFHGDVDREDEFMGNLESLLRNAHSRILLESAQNVDQRGMGTTLTMAYVVWPKLYVVHAGDSRCYLVRDGMAQQLTTDHTLARQMVEAGGLKPEDEAGSKWSNVLWNVLGGRSDGGEITAEVRQVDLKNNDRIVLCSDGLHRYFTAEQLAQVVGSDSNPESADLCEKLIQLANEAGGEDNITVIVSQPTGSDITKSTWIDDYDHRPPVSTQSLPKSDDLLLETIDTDEPDDSSDDFLHDTLPE

>tr|A0A5C6A270|OS=Rhodopirellula pilleata

MNQPSPEHSPVQPCANACGVTDVGRTRSENQDQFLIAELRKSMRVEASSLPLRSSGDLFGSTRGQLLIVADGMGGHAAGQRASSVAMDQLIEQLLNTVHWFLHNEHEQTHDEADFLESLKRLLHQAHKRILFEAANDLSQQGMGTTLTMAYIVWPRMYVVHAGDSRCYLIRDDVCEQITTDHTLARQLVDAGGLKPEDEASSRWSNVLWNVLGGNGQHELTAEVRRVDLREGDAVLLCSDGLSRYVSSETIAEVITSQTDDLEPVCHHLVGLANAAGGEDNITVIVARPSPEMRQASGVRAFERPADDESDLSADRQDDVVSIEEFADEDTLPG

>tr|A0A5C6FGJ9|OS=Crateriforma conspicua

MRSDESAIRLAKTHAYGMTDVGRKRRVNQDQFLIAQLNKSMLVTSTSLPLNHRSRLYGGIQGLLFLVADGMGGHAAGEKASSLAIDNLITQLLNSVHWFFQIDHDCEEDFIEDLKELLKNTHRRILSESAVNAEQQGMGTTLTMAHVVWPRLYVVHAGDSRCYLIRGGECQQLTTDHTLARKLVESGGLKPEDEATSRWSNVLWNVLGGQNEDGDLIAEVRRATLEPGDTILLCSDGLYRYLDEATLASVVSGSDDLPAICQDLINRANDAGGEDNITVVLARPCRSQLGCSESAMQSSAGSGETNTNDSDPSKLSDTDPLIQPGQFAEDASRDGQPAPGPESADSANPRGGDFLPGDVGPVIRDTRGNDDGIEDTLPG

>tr|A0A5C6UVD9|OS=Bradymonadales bacterium TMQ1

MAGAFKLDAAGFELKFSEVSLDRPPHPGGMISSTGVRVRAPPSASSETAMHTSQAAAHAASTQDRARSAGWELRFAAITDIGMRRTVNQDVYLLHPSRRLYAVADGMGGHEGGELAATLAIETLAAYFDEVGDMIESTPQDRRSLQHNLVAGIKLANAAVYQEAAERGTHRGMGTTLVALTFDETRAYWAHVGDSRLYRLRNGQLERLTRDHSLLEETLERHNITSSDASEFIANFPYKHVLTRAVGSQYCVDVSVAFSQLVPGDIFLATTDGVHNALSDEELASVLRDHADDLSAACQAIAQLTLSRGAPDNLTALLVETHTPGR

>tr|A0A5C7J8T2|OS=Rhodocyclaceae bacterium

MGAGRDCGAAARSGQRHCDRPARSACRAGRPRRRPAACRRSDRRARPGRDRVLAPAAEHRNRHGDRHPAGGHAAGRVEGVAGGGRRECRETGRAARVCTVSAPGADVAAGPARYTVLSHAAVSRRGGRSRNEDALRCFETASGVHGWALADGLGGHADGDIAANLAVDQVHAAFLDAEATMRADIVDADAFLMTAIHSAFEAANSAVVMQGSGRDDPRHMGATLVVAAVLGERVAWAHVGDSRVYRFTAGELRSVSRDHSTVALAAAPARDERHDESRNRLLAVIGGADDVRPDFADTPKALAAEDVLLLCSDGWWSLVLETEMEIDLAASRTPEDWLQRMEDRLLERAQGEFDNYSAIAVWGSAWTATARI

>tr|A0A5C8ZTE9|OS=Parahaliea aestuarii

MCGACKPGIGGLVSALEVVAEGSGATHVGQRNHNEDAYLLDGSLSLAIVADGVGGHQAGEVASAITCEVIQREVAAGRSIEEGIRTANREVMAAVAQGRGKAGMASTVVVARFSEGDYELAWVGDSRAYLWDGQLKLLTRDHSYVQALLAKGQITLAQARNHPRKNVIVQAIGLQEDDKLEVGINCGRLAPGQVLVLCSDGLSDVLDSAVLSEILSSDRSLEARCDALVETAVQAGGRDNITVIVLPGVAASSEAGLLEPEVLWTYDPSSGEYSGLPELEAVDGEEVPAPIPAVRRVVARKNSAATTPNPSQPESTQMMSASEIAAAREAMQREREQGRNRTIFWVVAAVLVAGSVVYSLGIIAAG

>tr|A0A5C9ABL3|OS=Parahaliea maris

MCGAGKPGFGGLVSALEVVAEGSGASHVGQRKHNEDAYLLDAPQSLAVVADGVGGHQAGEVASAITCEVIQREVNAGRSIEDGIRAANREVMAAVAQGRGKAGMASTVVVARFSDSDFELAWVGDSRAYLWDGQLKLLTRDHSYVQALLAKGQITLAQARNHPRKNVIVQAIGLQEDDKLEVGINCGRLSPGQVLLLCSDGLSDVLDSSVMSEILGSDRPLRDRCETLVETAVRAGGRDNITVIVLPGVAAHSEAGLLEPEVLWTFDPATGEYSGLPELEAVDENATPIPIPAVRRVVARKGGAEAPTQPESTQMMSATEIAAAREAMQREKDSRRNRTIFWIVAAVLVAGSVVYSLGIIASG

>tr|A0A5J4YE42|OS=Trebouxia sp. A1-2

MFLRPYLSVNSVFSCQEACRLEVFAALRSISAVGSVSCAPSTLERSAAVTTCLRPALVHYRTLGCWPMPWAGQVACNSNHALQNLNGLRKGLLTTQTLVRQLAPYAGGVTTLSAGSNYLRRQQTSRSKRRSALNHGVALVETNAPASMQQTGSASSSSGESDADMTGTPGNGSHSDDDGGVSNQHVRLSIGASCIPHPEKVDKGGEDAYFMTDDGKYLGIADGVGGWSQVNIDSGVYSRMLMQTAKAAAAITPPSPIAPQIVLEEAHHKTNVKGTSTACILALEDSKLHAANVGDSGFIVVRKQKVVFKSPSQQHRFNFPYQLGCPGTMSDTPENAELYTVDLSAGDIIVAATDGLFDNVYNEETAVLVSELRRRGQEPEAAASGVAQFARSRAGDQHHPSPFAQGATLAGWPNVHGGKMDDITVLVAYVLPASASSKL

>tr|A0A5J5F974|OS=Sphaerosporella brunnea

MRLHVCPRLFRPATITIYTPIRRASEQHYRPTRSSPAAFPFVPSYALFAKRPPRPFTPSRGRVGAWNTRAHGHAALWSRLVLHFWGKELLAGQWGGVDPTTIYATTNAGARGDQVWQGTTTVCGAVLTDRKLVVLNLGDSVAIVWRKGLKKWWHWFDCPTQLGTNSSDTPVDNAVVQEVEVQNGDLVLLATDGLVDNMWDAEIMTVIMGVMEVEEEEEVVEEVGEGWDQGRMKMLSQRILQAAKKTAIDPFAESPYMERSIEMGLVIIPTARPTSASLSSPPRE

>tr|A0A5J9V5N3|OS=Eragrostis curvula

MAELPLAAGLLDLRPCKLSPKPPPLPLPARRRTHSTAATAAVPSPRRAVPDLHSSTGKSLLEYPRPPLFSPFRNSYRLADWGVPRAELPDGSIVFHFGHRREALETKPEKASRGPEPAGGAGPDAADSPASFAVAGGEPQPEQSVPARAEEQLAAEALPTIPHSRSGPELEDGGGETGAASAADEEAAPEYPAGSNAGVEAEAGLTSAVARVASESEEIDGDGAVLEAGVRTVGVAVESTVTGSEERSGSDDASTVDGSGGEEATATAGLEESEEVSEGSTAQDSDTDVETESSSSSGDDKGAEFGVPLQIVEQVNNKVDLKKETSVLKSSDRMVPVAQSRLLLSSGAAILPHPSKVATGGEDAYFIACNGWFGVADGVGQWSFEGINAGLYARELMDGCKKFIMENQGAADLRPEQVLSKAADEARSPGSSTVLVAHFDGQVLQASNIGDSGFLVIRNGEVYEKSKPMVYGFNFPLQIEKGDDPLKLVQNYTIDVEEGDVIVTATDGLFDNIYEEEAAAIVSKSLQADLKPTEIAEQLATRAQGVGRSGAGRSPFSDAALAVGYLGFSGGKLDDIAVVVSIVRTSDI

>tr|A0A5M8QJW7|OS=Agrococcus sediminis

MGEQRAARARHRALPRLAAARQREARQGLARQGRPHLVRGCPRRAGSVPRQEDAHRAEDDRRRAGGGQVAAAVARDPRRPARAGLPARRPHELRRRGRRAPPAARRRRRRHRRAADGARAQPHRAGHAGGRGPPRRVPAGRAERRRAPHRTLLALARRRRGPRRHRRQRARRPPGPGREPSGRPADAPRPRAVGPDRRAHDHRAGSRRRLRRRLGRGHRRPHRAPRGGRRGRRRPRRHARAGVDPRGCRDARVRQRRPVRHHGRRAHRAQPRAGRRRGRRAPAADRRRHALDLEDPPAHHRGAARGGRPRVDERLERRARRCRAPARAGCAVRARARRRRAVRRPHRDGGGVRVSAVGEFRFSHPSIALRWAGATHPGARRTVNEDSVFASFPVFLVADGMGGHASGDVASALAVEAFRSLKGRTLADVACVEEAVERAFDDVRTRALGEPIGGTTLTGSVLVDVDGVAHWLVLNIGDSRTYLFEQGALQQVTVDHSVVQELVEAGALRRAEARSHPHRNVITRALGPGERQAADVWLRPAERGQRLLVCSDGLTGEVEDDEIAEILGDVASPAAAAALLLRRALDAGAPDNVSVVVVDVDAVALADGDLHDTHAQSVDDTIPREALR

>tr|A0A5M9KZR9|OS=Pyrenophora tritici-repentis

MPSTSFSEPLSTHNRSRDRRPTVNGQMIRGVTNGDDAVLVSESFIAANDGVGAWATREKGHAALWSRLIAHFWALEVETASYSPTSPPNLIEYLQNAYNLTKEATSEPNPWHGTTTVCGALLGADNETPDHPLLYVTQLGDSQILVIRPSTKEVVYRTQEQWHWFDCPRQLGTNSPDTPNDNAVMDRVPIQEDDVVVAMTDGVVDNLWEHEIVENICESIERWIGDKEKDTDEQTHADGMRFVAQQLMNAARVIAQDPFAESPYMEKAIDEGLSIEGGKLDDISVVVAQCKRRKG

>tr|A0A5N6NSP1|OS=Mikania micrantha

MSNYSKNKSELSKIGAEAFGDLLHGNFSTTTAPSHKPSPMLFPYQYKPQQAYVVQQAPAKTTETVITCYEALNKYGGTLVTDYPKRKHVARCYLLNSLKFVHIYLLAVAPVRAISRYCNLILDVEHLWTKIMPTGSLSKFNIPLGFGLRKGIKLQIKNQIQQRNRLFSKPAKTLVPKSDLEFGNLWENFQVTGVIKLHSCIFGYSRNPYFSKAMATSGSVAASGDLIVDNLISNCGNVSNFARPAGHFAERNRTYHAASLGVKNREPNKIRGDHGYFIFGVTQMRNNVNTFGGQLGRRYNTSSSTCYSDGGVSDEILKESSHDEAITSLAIEADGKGVLSRTIKLLSGSSYLPHPDKEETGGEDAHFICVDEQVIGVADGVGGWADVGVNAGLYSRSLMSNSMKAIQDEPKDGIDPARVLAKAHLATKARGSSTACIIALKNEGLHAINLGDSGFVVIRDGCTIFHSPVQQHDFNFTYQLANGNEGDQPSSGQVFKIPVEIGDVIVAGTDGLFDNLYNNEVTALVVQGVRSRVSPEVMARNIAELARVKALDRKRQSPFSAAAQEAGYRYHGGKLDDITVVVSFVTGPSGE

>tr|A0A5Q2RQR4|OS=Actinomarinicola tropica

MPSASTTTSRCDFDTVVASPGTTWERAGPDDEGSRPSTTRSPTATRTSPASKRSTTRGSGSAVRRASSASPAPGTEERATVRASRASSSGTGTGSSRTSAVPGAAGGSSAAVRRASAIRSTTPRSRIGGTSTLHPRGPSWIATSPASVWRTSRKSSPRPPSQIVATQATLAQRGRRVRATPMRSGASSDTTRSGALRRVATTTSATAPADHPGAGTDRYLVSSALGRARAITSHSQPVATLRHQPDRGGTSPPVGPRRARWTRRYDPPATWSAREPEMVIDVRWGSATHQGRVRQHNEDAVLAGPDVFAVADGMGGHAGGEVASGIAVAGLADLPDLTTSDDPASLVVAALGQVNAEIRRRAAGDDGVAGMGTTIAGIARVAGPRLLVFNLGDTRVYRFRAGRLDQLTEDHSVVGELTRRGEITADEARHHPHRHVVTRALGVESDIRPMVATIDVAVGDCFLVASDGLFNELHDDQMGDLLATPGTVESRARALLDAALLRGARDNVSVIVVVAAGTSQVDSLDADTSPRVATPADPAAAGSTLGLTAAAGDRSLTPTPPTG

>tr|A0A5Q2W2I0|OS=Pseudactinotalea sp. HY158

MGASDMPLGFRWAVQSDVGTVRTNNEDSAFASPGVLVLADGMGGHAGGEVASVVATNRFARIDPGGGDLPAQLTGAGRETRAALHAMSVADPSLETMGTTAIALVSDGARLLAGHIGDSRLYLLRGEELFQVTTDHTHVQYLVDSGQIHPEQVATHPYRAMLLRSLDDQPGGTDLDLIEVDASAGDRLLLCSDGLSDYVAVPVIGRLLAAGTPDQAAAALVARALADGTRDNVTVVVADVEQGPVDDVAAAGGAAADPLVLSPEAAGALGAVMPELLTGSGDDAGDGAGDGAGADAGDGAGAGVGVGAGESDAGAGIADARGDAFIDAGDSVGTDTGAPADDTDVDAGQAGRAHAGDPGIRADRGHDRDDAADATGAPADVAAESAADAAVQTPGAGQSAETAEPGAAVPSGETVPAEAAAGSGSLGATESIESTESAEPGVFGGSGRGERADAQGAPGTGDTSPYPEAATPISAQVPAPPPPRSGIPVAGVLAAVAILILGAAFVALL

>tr|A0A5Q4DAU5|OS=Gemmatimonadales bacterium

MDNGILGGPAGVLLLAVGIGAVVLGAWLWFRSRRTSRSEPPMLVFPTHRASRARGVQAFDAHVSGGPASTSNGTAATSPGPDGSNAARRDNPIPPPRPTPPATAAPAGRGASDPGSRMVISTAPAPPGLHAPGRLSTGGGAQQQRPPDGTLQLLPGRFEVTGGPGAGEEIRFVRLPGLPAEITFGRGDGPEYRHVRLESPTVSRQQARISFNGGNWSLRNESSTNPTSVNGSPLASDIEEVPLSDGDRIEMGEISFTFHHKETRDRLPFRSSWHTDLGRRPTNQDAVIVKSLPDGRELAAVCDGMGSHQAGGEASHLALEALVAALSLGAGLVDAVKQANTRVMDAARLGSTRDGIGTTLVALLRHGATYQIANVGDSRAYRIAAGGVRQLTEDHSFVAEATREGRMSHEEASRSPWRNAVTRNLGASDHVEVDLFAGFDATENHLIILCTDGVHGVLAEDEIAEVCRQTPDVRDLARALSEQALIRGGEDNVAVAAVSFGSLRAATSPTTAE

>tr|A0A5Q4DHS3|OS=Gemmatimonadales bacterium

MRRRTSGGHGFAQRRALMTGWTWALLLLAVALLAGLGAWFRLRGRGGAEVRDRGSSPMLVFPVSGGSGGGGPARPSSDRRPAEQRGTGGPPPRKAAVGIPATRNPPPPAPARPPSASTPVPPEHDGTLQLLPGWLERLAGDGAPDEIRFVRVPGSPPEITLGRQPGPEFRHVQLRSPSVSRLHARLCLGESGWTIRNESATNPTLVNGTPIDSASGDVPLDEGDRIDMGDISFRFHGQRTDHSLPSRSGWHTDQGPRAVNQDAVLVRRLPDGRELGAVCDGMGSHEAGGRASHAALEALVGTLDGGGSLEEGVAAAQAAVLHVAAGETYREGVGTTLVAVLREGSSYRVVNVGDSRAYRFHADELTQISRDHSFVEESVASGRMTVEEALRSPLRHAVTRSIGESPASEAEYFGPFPTRRGEILLLSSDGLHGVLGATDIAHILQSGLPVPDLPRRLVKAALDAGTRDNVAVAVLSFHDAGG

>tr|A0A5Q4GFI7|OS=Trueperaceae bacterium

MQRATDRSGVGELRAAPHAERVEVGGRDHHRPRRSQARDGGGLEGGTVLAQDGGAGGGTHPGHAQVVFHSDRDARERPRVTSVADRPIDRPGGLERPPVVDGDEGVGVLLGGVGAGQRGTHLVLRRARPRADVGGDGPGAAHPRHPTRRSVRRDRAVRNSSSCAPAWLAYTRPVQDDPPSRDAGAPPAPRRVKAAPDRRGASERGTRVEVGGASDAGKVRLVNQDAFWVGEIPGKGTLAVVADGMGGHQTGEVASQQAVQTFRESFDRSRATPPAAMARASQAANLEIYEYALEHPEHRGMGTTLTTVLIDDQVAIVGHVGDSRAYLVRDGAIEQLTHDHSWVADRVRQGLLTDDEARRHRWRNVITNALGATTAFKLDLHHFELREGDRLLLCSDGISMLLSELMMQQIISDNDPQEAAERLVEEANDRGSPDNVTAVVLRIEAVSTRAKRYALPERDDASIDITDTMSGIRRVEDAFPSRGPLATLRKQAWYPYRMWIVGSAYLLLLFVLFSFWRAAGA

>tr|A0A661P826|OS=Deltaproteobacteria bacterium

MVSTSPKTPTSARTRGIKLEFGQATDPGLDPAKAVNEDSCGYAETAFGHLFVVCDGMGGHAGGKQASDIAIRTIFDHVGTAPVDREPNDVLSEAIETAAGRVFDLGGPATNQQRPGSTCVALLLHDGVAHVAHVGDSRGYAIRGEQVYRLTKDHSMVQELIDSGALSEREAIGHPDSNKITRALGMAPTVDVELRAEPMELNEGDVFLLASDGLTDLVDSDDFLAVIPDHVARNDVPGACAELVALANRRGGFDNITLQLIHVLRASLPAGHTLPQEPSDGVAGSRVATTIDGAPVVSDTVVQEPQFATPAAAPITGCMAGAPDMQEAPTLIQPAKRRVATTKPEPPPLVADPVVRPPAGPQPVARHFEPPDHFDPPESEPRSTPTLVYVVVGMAVAISVLLILLIWALFYR

>tr|A0A661PI92|OS=Deltaproteobacteria bacterium

MVTTSEREPTVCLQPTAAGKTDVGRKRDHNEDQVLVVDEFGLFAVADGMGGHDAGDVASAITAASLEDYFASPPAGDTLAEAFEDLPAGAQRLASSILHANRQVYGKSGRSAHQGGMGSTVVAVHVSEEEQQIHIGHVGDSRCYRLRDGALEQLTPDHSMVNEALKLNPNLSEDILKQLPTNVVTRALGTKESVEPDIRTEPLVAGDLYLLCSDGLTGEVSDEEIHFALEDQDDISDTCELLVAMANEAGGRDNVSALLVRIDGPDGPVAARGYTDVPIVVDEDEDELPLPPPPSDEQAATVDHAFVEVEDDDDDLPPPPPPADEQTAAVEHAFVEVEDDEDDLPQPPSVSEPDGEVEAVEVEVEEEEEEEVEAVEVEVEEEEEAEVEVEAVEVEEEEEEEEEVEVEAVEVEVEVEVEVEVEEELPPPPPVAAAPRGSNLDWVDLQDDEEELPPPPPLAAEPEDGAANAAAALPLTKKSTPPRDRETTRAKAEAALDEAVDLALAEVDYDVEELDLGDDAVAEAALSESGGQGVLLVQTPAEQEETAPEISAMPIVEVGRPAEDQEATPDPAALRCPECGHQRSPEERFCGMCGSPLPEAEEPEPDVALCDECGHEILLGTTFCVECGAKHDYGEE

>tr|A0A661SEG4|OS=Deltaproteobacteria bacterium

MEMQVEQGTNVGLEREENQDDLGWFSPNKDELFIVADGMGGEAGGKTAAKMAISTIKEVFEKGGGSVHDLLKTSTEAANCRIHELGNSGDPRYHKMGSTVVVLFIRENKAYIAHDGDSRIYLYRGNQLHRMTKDHSHIQQMIDGGLISPEDAEDHPDANIITRSLGAKPTIEVDIRPEPVTIYPGDLFLLCTDGLCGLASDHEIQDVLAHGRTAKETCDDLINVALNKGGYDNVTVQVVSFEREPGEEIAPPSSPLKTTGIIGWARYKLGLPILLAAVCVCLGLFTGAAWLLFKESPPPKKSSEITQTTIVSPRPQPPMMVDGDASLSVEKILTVEVNSGANVREKPSMDAKVSFTLKKGDTVKKTGPDRWYLVMTEEKKTGWARKEFFEDSREGGSFIVRESDSVYEKPSEGKPQKLFLLNKGGRVSVIHKDDIWFLIMDKKERTGWAHRSLFEEHEEFTIVKPDPIISLVRVYQKPSDKSEELFNLKEGDEVSVIHTEDDWHLIMTKDKKTGWAFQSLFVFVQKNEESLSPQDETDKKLPHKKPNPATSNKNGKVGCPIMVYTVKERDALGYRKAIKDERK

>tr|A0A699YGW9|OS=Haematococcus lacustris

MVDLDAEDNRYLAMRFPDIQPPAAVLFRDRLMYEYSGSVVAKDAVQQVKAFYQSDYAQQSDIEKAQEGKWVKIALGVAMLAVAEIGVDAGAYARQLMGFAKELADQLTAPGCAEYDKLCSNMTKPPQDAAEVKNSTANMLQQTILEKAHAQVDVRGSSTASLLVLAGDRVLASNLGDSGFLVLRNGQVIFHSPQQQHDFNFPFQIGSSDSMSDMPASAQKFELAVQAGDVVVMGTDGLWDNCFDEEIMSVIRYCQDVNMPLDKTAQVLAHYARHRASDPKFASPFAYAAFQAGFAYMGGKMDDITVVVARVAHALEVADNPLPGQGGQDLTMPHLASKL

>tr|A0A699YJI0|OS=Haematococcus lacustris

MPLKLVNGVCCMPLCLHMAPGACEMTIMRDGATAPMDLASCNTQRNPGGNAIFQCTSTSPSQRKPRVFYQSLVLWHMIFDEHSLIRSRVPGIVHVLNPDVRSLRAPVKQLLPAMHGLMLPTGPAHIAHVRPGASRHLWAVPSSCVLCSSALHQPKTGFSSPSLGRQSQWGAPSRKVSCLVYRTLPHTVATRKLTSCFPCGGAQNCTQSPRGLTAAIAQALSTSTSEAAVAPASSNGSQQSSNGAALQRSQGTSPYVLVAGAYVLPHPDKMARGGEDWFFVSDTMHSLGVADGVGGWAEIGVDAGAYARQLMGFAKELADQLTAPGCAEYDKLCSNMTKPPQDAAEVKNSTANMLQQTILERAHSQVDVRGSSTASLLVLAGDRVLASNLGDSGFLVLRNGQVIFHSPQQQHDFNFPFQIGSSDSMSDMPASAQKFELAVQAGDVVVMGTDGLWDNCFDEEIMSVIRYCQDVNMPLDKTAQVLAHYARHRASDPKFASPFAYAAFQAGFAYMGGKMDDITVVVARVAHALEVADNPLPGQGGQDLTMPHLASKL

>tr|A0A6A3BMY5|OS=Hibiscus syriacus

MLAMIKDADEGNVGEIKRNSTLSELDEAVPFSFSIGVELHSGGTTLEREEIPAAGFFLYSGAALLPNPTKAFAGGEDAYFIACQNWLGIADGVGQWSLEGNSVGVYAKELIENCERIVSDRNGVPITDPVEVLNRAAANARSCGSSTVLVAYFDDQALHVANIGDSGFLIIRNGAVFKISSPMLYELNFPVQIEKGDHPSDFVEVYRIDLDENDVIITATDGLFDNLYEKDIAYSSSNFAREFEAPDRKKIGREDNTDFKSTNLAVKDSEEAFHPASFLLNL

>tr|A0A6A6LD30|OS=Hevea brasiliensis

MFVGVARNYKEGMRFVDGFESPTLSSGTALLDIREAADNRLTVEVLPSQGASHGKGKLIMVSGSFYIAKGKHSKPQGEDAHFICVEKQTIAVADGVGGCSRKGIDAGIYARQLMENAVKILLNEPGDNVDLCTLLYEAYLKTRAQGSSTACIIALLDNSLRAVNVGDSGFMLIRKGEVIYQSPIQQHSFNYPYQLECDEVDSSPSYAEEFVIAVESGDVVIAGTDGLFDNLFATQIEEVARAGIQQGLDPQDVAWTIAQHAYHISIDNNAFTPFAQASTKAGRIRQGGKSDDITVIVSYIIDA

>tr|A0A6B1DT06|OS=Caldilineaceae bacterium SB0662_bin_9

MDQKLVVSRLGDVMSREPSSNNPRQDKEPSTLDKLVQKLPWLGEEFTSRQSLDGLSEGDPSKPDRSTSTESLPEPSQDHSEDKALDNQGLSSGGEAAVPPDGEFAAVVPEETGVQSDRNHSGDEGEAVEPSEMAEPHVAEPGSSDEMSEEDFLTEQTVSMSDDQDIAEVDMVGPTQDTQFTSKRESDPADPESKLEDPLEGDDGAHIPTLGETVPDDVDETANGLDTSSFVDGDDLEEMILEPLSEDGSLEPGVVHMMGDVHYVVTECLPRGYYRGHNELEDETKAVLFHLSPLSHRDLWRDAGSRHRMLPEIQYEGEDGHVLEDIEGQPIGTGLSLQQALQALDRVRQLMRFMSVRCRVAVTDICIEGLVSTTDTGLRLRYLPALVPMDEPAHVSCSDGATPIEGSETEKASEQTSVFLWGAMLHALVIGEPLSAEGLDTNVLVRLRDPGLPQLLATTLEQQHPCPDLRTLRDICRDFMVSPALRYMVGAATTVGLNPDRLCNEDSYGVVHSQCEYHDSRPQLVRACVADGMGGEEAGEVASKAAVDAFCHAPAPTSFLQAEEQVRWTRSLGWTANRAVLEALAGSGGGCTLTGVVVVNDCLTLAHVGDSRAYLHSRERGLQLLSRDHSQIKALLDSGLITEDEAAASEDTNQILRALGDGRQDVLAEEYVDTLGGLKDSAGGSVHGETLKLQVGDLVLLMSDGIWGSWEYRESVISDALCQVITDADREPQAVADSLRQAALDAGADDNATVVVLKRVG

>tr|A0A6C0U579|OS=Kineobactrum salinum

MTRDPDARWRACGETDVGRREHNEDAFLIDHQLGLMVVADGVGGHQAGEVASQITCEVLARDVHESNDLEPAIRSANLEVHDAVAGGRGKQGMATTVVAAQFSGAHYQLAWVGDSRGYLWDGQLKLLTRDHSYVEALLEKGQITFEEARRHPRKNVIVQAIGLQEEDKLRVGTNQGVLLPGQILALCSDGLSDVLDCDRFASVLAQDLPLEERCRILVMAAVEQGGRDNVTLVLVEGLAGGGSDAAADAAALEPDVVWRYDPASGEYHGLPELVQPGLPGSEPPAPARRASIAPKRSRVAPRSPESTQMMSADEMAELRREALELPARRRRRRLLVASMAAAVALLAIAAWFVIGPRSGG

>tr|A0A6G0UKI9|OS=Halicephalobus sp. NKZ332

MEYIKTKGTKPAVTGLEQLGDERNERPLTMFVRVANVTNGEQLLDDVRQSVLVKEIGAGLRATGGSGKTQLCAVITASYLSSEATTQVKPGTSVVNATSCGFPKDLINGPSCVLDHGVFGEDACFIARHQSTHVAGVADGVGGWRKYGIDPSEFSSRLMKHCADIVKSGDFEPTRPDLIMAKAFTALAEAPRPVGSSTACVVVVHQNMLYTANLGDSGFLIYRKGKILHKSQEQTHYFNAPFQLTLLPESIETNGFITDTPEKSDMQKIELESGDVVLLATDGLWDNVPEKIIVDVLKNIEPQNIQMICNTIALIARRLSHDEEHKSPFALKAGEYGITATGGKPDDITLVLLYIN

>tr|A0A6G8DD02|OS=Microbulbifer sp. SH-1

MSEAKVDANVGTSGNTGGKEPASEVSRPAARATVRSAGATHPGYRREQNEDAFWGDESRGIWVVADGLGGHHAGEIASQTVVEEIQRSSATDRHYEQALRRAHALLAGEETNSTAMGTTAVVVAEDGNYFHIYWVGDSRAYLWTPPATDADNQDDTKTPHGTLKQLTIDHSYVQMLVDSGAINQEEAASHPNRHVITRCIGGSANPTLEIDRASFAWNPGQKLLLCSDGLSNDVGLEEICQILANTPDNQRASELLVAAALDAGGRDNITVQVINSPDSATANNENLNSRTLPQPLASRQLFSSPGTLVARIATLAIIFSLGIYAAWQLLNG

>tr|A0A6I2WTF0|OS=Actinomycetes bacterium

MNEDAFVVTDQVFAVADGMGGHLAGEVASSMAADSLRRLSGDVTADDAVNAVIAANLDILSLSRSDAAHRGMGTTLTGIAIVQRDGEARLAVINVGDSRTYRLRAGRLEQVSIDHSYVQELVNSGHITAAEARVHPQRNIVTRALGIEAGIGIDVWTLPIVRGDRFLSCSDGLVDEVTDEVILDALCDFSDPQAAAEELIRLANAAGGRDNTTVIVLDVQEGADPATINGGEDLLTYVTVRGHDVEDGSRDDSLDDLNIEDERTALTDDTPPFETNPPRDLTDAVLSRGLDPADRYPFPVAHADETPNTAARRMAMHARANTRFIIGGVAVALIAIILVIVAAVTGGKSNDGPQPGDTVAITDFVDDPADTAPDTLPVSVNGTIGDASDSADGSTLSS

>tr|A0A6I9RAR0|OS=Elaeis guineensis var. tenera

MPSTYFSKKKGLQASSEFLLGRGQNFLFGHTLSFPSQPLSTLVHGIHQSPRSSINVLRNFNTSDCRKGPSVIGTISRTFSVPSVTGPSCQPCLYNVDTVLSEPNQKLPCSLFKRSIMSACSSLLGRAEPGAWHLEKMSSSRQILNSVMQADICYSYKGLDYCRRISGNLKNREPWGTNMVYRCFWSNATGTSWKSKFSLEPGIRDFQSQRTVPYSAEAAPDVSLDGTPQQEQFENSAVPSDQKVLSDRTLKLLSGSCYLPHPDKEETGGEDAHFICVDDQAIGVADGVGGWADLGVDAGQYARDLMSNSVSAIREEPKGSIDPARVLEKAYSSTKAKGSSTACIIALTDQGIHAVNLGDSGFIVVRDGCTIFRSPVQQHDFNFTYQLESGNGSDLPSAAQVFNFPVAPGDVIIAGTDGLFDNLYSNEVTAVVVHAVRARLGPQVTAQKIAALARQRAQDKNRQTPFSAAAQDAGYRYYGGKLDDITVVVSYISASSS

>tr|A0A6J1QWG1|OS=Temnothorax curvispinosus

MQSIYWTGRLLSRALWNGIASYSTACGGEQQPSAAVASTRRRETSLVSAVCGFPKDFARSRIRRGQFGDDAWFTARFRTAEVIGVADGVGGWRHYGIDPGEFSNFLMRTCERLVSMGRFTPTEPAGLLARSYYELLENKQPILGSSTACVIVLNKETSCIYAANIGDSGFVVVRRGEVVHRSSEQQHYFNTPFQLSLPPPGHSDLVLRDSPESADTSSFGVEDGDVILLATDGVFDNVPDQLLVTEMRKIEGERDPTKIQCVANTIAWMARRLAFDGAFMSPFAQNARENGIDAIGGKPDDITVLLATVAI

>tr|A0A6J4JZC6|OS=uncultured Cytophagales bacterium

MTVRVAALTDVGQLREHNEDNFLVCADLAEGNWFLVETPYALSEAGTLLAVADGMGGENAGEVASALAVEAIKEFFSNLPAAPGAGGAQVTQWLRQAILFAHGRIVEHSRRNPACEGMGTTILLVWVLGPTAYVGWSGDSRCYLYRQGEGLRILSDDHSVVWELVQSGRLNEEEAESHPESHIITQSLGDSRHPPRPDVLVQPLLPHDKLLLCSDGLNGMLTSEQIGQIMGRNEPPGELCKTFIDAANRQGGEDNITVVMLEVLTGRNGGSTDPDAAAAQAAPPDFLVTKPNGTITPKTIRTLRRKPLPGAPTARQSRWLAVAGVGLVVLLLVALWTNGRGQRGSSPAVPTNRYPAEANAGNRQEVRPAAAPFILSEAKLHDVTPPLLALLDRYRNAMGRIQALREKVEREGDATTAVPEATALSGKAESLPGAAALLAVRDSAAAFDVLRRVINSRARLDRLEGGMQALDEELVLVEGRLRKIDGEEKQ

>tr|A0A6J4TDG7|OS=uncultured Rubrobacteraceae bacterium

MPLLELQPFGATDPGKVRQNNEDALLVGDGEDETLFVVADGVGGFEAGEVASSIAIDVLRGLDPDDSFEGAIEEANRRILAAGRGDDRLSGMSTTVVAARFGGTRREPVVEVAHVGDSRAYLFRDGELRPVTEDHSLVAELVRSGDLTREQAFEHPQKNLITRALGTEDGVTVDTKVLPVEIGDRFVLCSDGLTDMVREDGVGEILTRHPESPEGAVRDLLAVALEAGGNDNITVVVVDVRERKEEPRASGRGTGSADRSGGTAEMPSLGVPPGTPRERKGGAKSPPAGATQQRAPGRPKRRPGGIARFLGALVRGLAVVLVVLIALVPAYLWGSSRYFFAFEEGEVVAYRGLPYAPLGVELNQEWRKPGLTESEIKEPYEQPIETHKLYTKGDAEKVLGDLGR

>tr|A0A6J4UQW1|OS=uncultured Thermomicrobiales bacterium

MGAGAVAIAGDELRIAVGAGTDVGGRTENEDAVLVETLPPIGDPLVGAESGHLLAVADGMGGYQRGEVASRIAIETIRRTFAEDPGADHAPLLKQAYRQANRAIYENGRGSGGNDGMMGTTLVVATTRGKYLTIANIGDSRAYLVRANRLQQVTRDHSLVAEQVSQGSLSAAEARESPHRNIITQALGHRERLDAKMPDIFEITLLAEDRLLLCSDGFYDVVPDDDLIGVLLDHEPDAAARRLIEMAVERGTTDNVSAVVLSVQPVRVRERELVGAGATPGGRGSFLFPALALLAAIVFIALVLLALTTL

>tr|A0A6J7G2D5|OS=freshwater metagenome

MGIKTVSYAASDVGKVRSSNQDSGYAGVNLFFVADGMGGHAGGDIASAITAQHVATADEPVENSQQAEQKLIDYIWQANQKLSASVAEHSDLAGMGTTFSGMLVHGTSVSIGHIGDSRIYLARDGVVKQITTDHTFVQRLVDTGRISEEEALVHPRRSVLMRVLGDVEQFPEVDLETFETKPGDRWMVCSDGLSGVVPERLMHRIMLSKSTVQEATDLLVGEALEFGAPDNVTVVLVDVVDAKEEVEVSASRNFVGSAASEVVIDERKGRRILRILNPMTLIEMLQKPEDPTSFAPESEELLEKILKDTKGRIRARRLRQLATYVLLVAVAIYGLFLGYEYTQTRFFVGTNDGVVVIYKGIKEDLGPFRFSKVYEVSSITLESLTDFQREALERSIATESLEEARRVLDQLGGN

>tr|A0A6L2NMF6|OS=Tanacetum cinerariifolium

MPTGSLSKINIPLGFGLRRSVFRPDISNRLSLKRGKSLIPKSVSEFGNLRKDCQITGVTRLQSCILGYTRNSYFRTMTTSGSVAASTDLIVDNIISSCGNVSSFARPTGRYVERNSRGFRAASVGMKSREVSKGHGVNGYYIFGITHMASSSLSGRRYNTSSSTCYSDGVSDRVLKESSLDDTITSLAIEADGKSVCGKTLKLLSGSSYLPHPDKEATGGEDAHFICVDEQVIGVADGVGGWADVGINAGLYSRSLMSNSVRAIRGGPKDAIDPANVLAKAHSVTTAKGSSTACIIALKEEDLHAINLGDSGFVVIRDGCTIFQSPVQQHDFNFTYQLANGNEGDQPSSGQVFKIPVAVGDIIVAGTDGLFDNLYMNEVTALVVQGVRSKASPDAMAKNIADLARVKALDRKRQSPFSTAAQEAGFRYHGGKLDDITVVVSFVTGSIDE

>tr|A0A6L4Z180|OS=bacterium

SDGLGGAAAGEVASAMAVQTVCTELMKTIKSVLSPNDRLKRATEQANDQIWACAQSDNSLRGMGATLTAAFIYDTSVYIAQVGDSRAYIVRGGRVKQVTEDQSWANAVKKAGLEIADVPSNVILQALGTQPKVHVEVTSVDLLNGDVLLMCSDGLSNKIKDLEMREIANSSQDLAENCRQLVDLANKRGGEDNITVILARFEGKSLSIGFEERDLSITSTFKIVTPLDFGEALDESETMSFRVEEPQTVDPMSVTQSALFDGIPTISGLPQLPTILNKASAKEIKSHAEMPVLPTTPLTSDSTSKNKEEKTSATVPLLPTDSSNSASKKNLTTSERAAFRQAETKTDLPVIAPVFDLSKEPIAEPKMEAE

>tr|A0A6L9MSQ1|OS=Alteromonas hispanica

MSSGEPALTYKAIGKTSVGMVRDHNEDSFVCDSEAGIWLVADGMGGHESGEIASEIASDEIPRLIKAGTLPSNAIQAAHEAIKSAPEKGVGVKGMGTTAVLATADRAQLTISWVGDSRAYLYSPTELIQVTKDHSFVQHLLDSGAITQEEAELHPEKNIITQCLGTESLESVTVDEIKLQLYKDEKLLLCSDGLTGEVSDAEIFEILKATDSLETAVSTLISKANENGGSDNITVILIDADESAIARPKAAKTRKMKAVGAATQHKISKAKNIAAGVLGILIALLIAYLLFTFILKPKEENTPTSVSSSQTHTLPDEINSREASSELESLLLPANLNNEHDELDLSANNIQIGQQNTPEQLPTLKNDGNSTPVLGDQITTEPLQQPKPIEKNAEDQKLKEGKKDANTPK

>tr|A0A6M0L1K8|OS=Sulfurovum sp. bin170

MRKIKYLDKRDQGARKDQQDSVDIVHNGDDVLIALGDGMGGHSGGKRASEIFISTARNHFESYDYYHHQEFFNNIIYETEDEIALYANQSGEDPRTTATLALILDNAVHFANVGDSRVYIFDREGLIIRSRDHSVPEMLLQMGEIEEHEMATHPDQNKLTKSLGPDSHVEATHYSYQLNYNSDYIVLVCSDGFWEYVDEQEMMYFLFNFELSTALTSMIDIARERGGSGGDNISVGVATLIADKPKKEAIEEDNWEESETIDEEIETPKRSFLQRYFILILSLLVVLVGIIGTILFTDRGRALLLTDDNQTTQKQDSNQSKSLTQESNKTEENNGSKENNQTDVNRTKKGVNHADGKV

>tr|A0A6M5J678|OS=Herbiconiux sp. SALV-R1

MGLASVGSSGTAGAGAAGGAGGGAGGGAAGARRASAGAADVRAGSGGGAGGGVRRNELRLGVGSSTHTGLRRRGNEDSLLASDPVFLVADGMGGHEAGEVASALAVEAFSTLAGAGALEPADIRDAFDRARAAIGRLAHSGSRRAGTTVSGVAVAENDGRAYWLVFNLGDSRTYRFSDGTLEQISVDHSVVQELMDDGELDRAAAANHPGRNVITRALGGGGLSEADYWLVPMEAGDRMLVCSDGVSTELDDEVIARVLREEPVAQEAAVRLVHEGLLHGGRDNLTAVVVDAWSSSDTERPRDGAHEGSAGASHDEDTIPRGRAGAFGRN

>tr|A0A6N2CZF9|OS=Deltaproteobacteria bacterium

MNDDAEQDLRPSPRRTPGWTPRETPPRGDEPLKERTVIDIESQNTVNLHVVCDTDVGRERTENQDAWGFVDLDGVFFLIVCDGMGGHNGGSTASRLAVQTIEQTLEAEQGDIPARMTAAISKANLRIFQWARRDPALYGMGTTVVLLAMDRQSGFAHLAHVGDSRAYLLRDNVFERLTRDHTMVQRLVDDGLLTPEAAENHPHSNIISRSLGGGETVEVEHHPDPISMRNGDIFLLCSDGLTGMVPESFVPRYLARHGIDDLPKVLIDESNAQGGLDNITVGLALVGDPPEPRTNYQLVTPKILRGRKPHETSQTIRALDETARTRPNTLTGPAGQTLMIPTPAEGIPVPTHPQDSSAGPAVPPPGADVSAIAPPGPQPARRSRDLMLLLVIAGLGALVIGVAALLFLR

>tr|A0A6N2DQ56|OS=Ilumatobacter sp

MAELRWGAATDTGRVRTENEDNLFAGSTLFVVADGMGGHQAGEIASQITVDRLESGLTAETPTLSDLVAAIGHANRDIFDAAIENPEQQGMGTTVTSLAVMDDPDDGEVFALANVGDSRTYVLRNGRLRQLTIDHSYVQELVAEGHISRDEARHHPRRNIVTRALGIERSIRVDSWTLPIVRGDRFVLCSDGLVDEVHDDEITAIVEANDDPQAAADALVAAANEHGGRDNVTVVVVDVLEGDDPPDPTEEIDVIPAWAPKDDQDDTGDIDAGIDTGIDTGDVPVTPPPVDVDADPPELFVSPDAAGDDPPGPGGSEPPPVDPDADPTWLVADPKAVAPPDGADMSDAITPTIADANGSDPEPPRPRRRRVARFALVVGVAAIAVTGFAIFSAWARAGYFVAFDDDDRVVIYRGRSDAVLWFDATVEAPTPLARDLLDERSIELVEDERRFSSLRSAEIFVTDRLTPTTTTTTTTTTTTTTVPPTTTTEDEAGDETGVDDDAGSETGTTTGS

>tr|A0A6N2LA21|OS=Salix viminalis

MPSGVLLKLSFGLSRAMLSRNSVSLVSNASQFSSRRSLARTSMAASCSKAVFGDLHIDDLIATCGNGLEFPKSSGVFFSDRSRSSCVKASVKMRNGELPKSLLVCGNAIRKNGNANNLVLGPILKNLYSSSSVCVSGRAQDVSFDGNFSEEQLVDSTAVSGLSNFIIINYKIVVQTLLVFLYFAMDLAVVKQLTLFKFLQMNWDLKGSCLIVTYLHGIDFKNIVDDRNLKLLSGSCYLPHPDKEETGGEDAHFICEDEQAIGIADGVGGWADVGVNAGLFSRELMSHSVNAIQEECNGSIDPSRVLEKAHANMKARGSSTACIIALKSEGLHAINLGDSGFMVVRDGCTIFESPVQQHGFNFTYQLESGNGGDLPSSGQVFTIPVAPGDVIIAGTDGLFDNLYNNEVTAVVVHAIRTGLGPEATAQKIAALARQRALDTNRQTPFSTAAQDAGYRYYGGKLDDITVVVSYVTNSANI

>tr|A0A6P0LHB1|OS=Moorena sp. SIO3H5

MLVCPQCQFENPNANKFCQRCGTSLTDKSCHKCATQVPLNAETCHNCGAFTGTVWRAIISKQQNSLTSPQNSPQTSVEVSDSSANQATPVAVSQPQSDQTESELSQPESSLETTQDWLTKSDTILTESGDDATLSQASTLLADEASINNSSGSDQVELESGEEPLLEITPIQEKPQSTEASTWSIPNLENPTSQTATIVYLDQGKRYKVLEPEKLRYKIEESKTTDNSIFVTVLDCQPLQKSPLEALINQTILSPEKFDLSTNLEQPQPSSKENLDVWKVLDIPQSAKPYLALKDLCYPGVPEIHDVWQQNGKVIILLENRSEWQLLSNLWGSEEFSKLQILYWLDEMAKLWQALEPWHCRQSLLELTNIRVDEDQALGLEGLYLEKEENPLTLEDLGQLWQRLFNQSQRTQFTSLSTVVRQLCTKEIETIEELRSHLQAIAHEQESDLYDGMELTEAVLSEIARADTEPPDSEELLSDLSAKEQQGDEMPTVLLPMSLQSLDDLGITDIGHQRDHNEDCFGIQTQLKKQENPIGRSIQARGLYILCDGMGGHAAGEVASSMAVEVLQNYFKENWQDQFPTEDSLRKSVYVTNQAIFDINQQNARSGSGRMGTTLVMMLIENTKVGIVHVGDSRVYRLTRKRGLEQITIDHEVGQREIQQGVDPELAYSRPDAYQLTQALGPRSEQFLNPDVQFIDIAEDSIFLLCSDGLSDNDLLENHWQTHLAPLLSSRSSLDQGLANLIELANEHNGHDNITLILVRVKVRPNLAQPTLNIGH

>tr|A0A6P0SV45|OS=Moorena sp. SIO2I5

SSETANDPETESEYLEEEYYPENEISPDSSGQKLMLLTSLPESQSTLETWLQEEHSYQECLSVIIPICQCFYYLYQRQWCLVDVIPKLIEIGKPIKFFDLTSAYPIEQKLDYGLLGNYCAVELSSGNPFQESMSTYTVGALLYQAIHQQLPPQDQTLDIKIQPIPQIYQLLKICLSSVPEERFSLSHLLSLLIETRKSFQTTQVQWQVASKSTVGLSTSRLHNEDSYGVQQQQLSNGDTMILAAVADGMGGMSQGDLASQLAIKTVLEQPIPSEFKTVNQYTEWLVSLFQNANQSVTKAVRDGGTTLSVILGISHNLLVAHVGDSRIYLIRKEQICQLTEDHSLVAMLLASGEITYQESLDHPDSNILTKSLGSKPRLSDGYVQDLSRFSQDLSLALEDRDILVLCSDGVWSLVLDTELAETFIKTQPLQAAVDEIIQKVLDNGASDNATILALECCLKKAN

>tr|A0A6P0TGJ3|OS=Cyanothece sp. SIO2G6

MIQCPNHTCQAANAETERFCYRCRAPLPRRLLWAVGHQVDTFSPGEILSDRYLCKASRIFLDQQPGIPPGTLADVPKAVLPYLHLISHQVHLPQVYGWVDAELQSGGVTRLLLLENAALLSTVTTPTHAYPIDLTRTTDPDIPPKHLPCLLPSLQRAWPHAAAAQQLNWLWQLARLWAPLAAEQVAATLLKEHLIRVEGPLIRLLELRFGLNTTMMSLDQVEARPSAISAPTLVQLGTFWSGLMAQAAPEIQDFGQSICQQMIEGYLTDSAMLQDILTDAIHQSGRSQTLRVSIATQTDKGPSRSDNEDACFPPEGSVMALQDRLEAQVQEDTGLVIVCDGIGGHQGGAVASDLAIKAIAQQVRHSNHQSAEISAIQSDLKLAVAQANDVISQNNDDKQRRDRQRMGTTVVMGLLHGHDCYIAHVGDSRAYWVTQWGCHQLTLDDDVVSRETRLGYGTYRSILYQPNSGALVQALGMGPSKNLYPTVQRLMLAGEGLLLLCSDGLSDQDLIEACWEEVLRPVLLGNVADLGSVSQRLVDLANTHNGHDNVTVGIMHWSITQTAPIQLDAVTDLSEFITQELEGDRPNPSRRPITGLAPLPDSLEIRGIAHPNARSSYDQGTVPPSDEGTASNSGSHGSQADLLDNPDAETANISPLAIGLQGLKVDAPIPTLGNESDPTSDNPADASSSASLLLQMMIVAVAIASLGMVSLLAYVWIPDVSDRVNAILGIESPPHDIEQDRQEQDEYNNADEQDLILDSSAPAVADPSFGIGSVVQIKPFPPVPSPDNNAPLPEQSSVQPLLLLTLEPMPSEQGSTPSSPVQVPSAQILGAIPGGSVLTIRGSFEVPEQGATETRPEGQAGGDRPIDASSPGASSPGASAIDNTSPNASATNRSSPTVVDSRAAPDDGIVLLAGRWLEVQLCTVPPTSNSALPTSTLAPGAIGWVSESDLKTLTHRQLKPPTDPNNPCTLAPDQARD

>tr|A0A6P0XMR7|OS=Okeania sp. SIO2D1

IFWSNLIPTAHPHIQQPLQEICQMIGDIGESLEAIAPKLNQLLLEQAAKLPLNAEIVGATDTGPVRLHNEDCCYPTEQDLVSNQLVPHLAMICDGVGGHDGGEVASQLAVQSIKRLVQNLLTEVAQQQDLTSPDLVMKQLEEIIRVVNNMIATENDEQGRESRQRMGTTLVMALQLPQQVKPSLESNPNNAHELYIANLGDSRAYWLTKNTCQLLTVDDDVASRETRLGHCLYWQALERRDAGALTQALGTRDGEFIRPTIQRFLIEEEGLLLLCSDGLSDNDWVEKSWTEYTSRIFRGEFSLKQAVEYLIELANDKNGHDNTSVVLAHYRISPEQLVLLNNPTVTPLEKEVMPELSEASKALLYSSDDNDDVSELKPEVDLDPDPDSEVEAMPNSPRLKPWMFMVGLLLILFVGAVVAVWRNWENFRSNPNDAPASWESPMIPQPSEVPVSPDS

>tr|A0A6P1NU21|OS=Pseudarthrobacter psychrotolerans

MNSQPASDPADADHGTGLSLSYGYGTDRGLRRELNEDSFIASDPVFAVADGMGGHEAGEIASGMCVRALAAMPQLATGERSVTAAVLQQYLLRADSSIREVTGARAGTTLTGAVVVEQMGMPYWLVMNIGDSRTYRLSQGHFEQVSVDHSEVQELVDAGEITPEQATVHPRRHVVTRALGTGDETEADYWLLPVEEGDRIMVCSDGLNGELTDEHISRILSTVGHPQDAVDALIQAALRNGGRDNVTVIVVDARNVMNDGGLATTAPRRAADAAEEDTLPRARIVDASRPAGPDDERGER

>tr|A0A6P5GBV3|OS=Ananas comosus

MANASDLVMDEEVSYNPQILLKKAHAATSSIGSATVIIAMLEKSGTLKIANVGDCGLRILRKGQVVFSTAPQEHFFDCPYQLSSELIGQTYRDATLCSVELTEGDTIVMGSDGLFDNVFDHEIVSVISKIQNAVEAAKALAELASNHSVDTTFDSPYALEARNMGFDVPWWQRILGKKLTGGKLDDITVIVGQVIRASID

>tr|A0A6P5TAI1|OS=Prunus avium

MRMICGSYYWPKEDKLKPEGDDAHFICAQEQTIGVADGVGGWAKHGVDAGQYARELMCNSIMSVQKQTIVDPRRVLNEAYADTKCEGSSTACIVTLRETGVLHFVNVGDSGLMVFRNYKLLYMSPREQRSFNCPYQLGNSRGSDNPHSATEIEIGVFPGDIVVLGTDGLWDNMYPNEIEQVVLGNMRESRVMKPQELACLLADLAWLRSLDKDSFSPYSRAAQEAGKNHPGGKKDDITVVVGHIMLTSPVSSCVEDFVERPLEIIWDNTKPMERASSTPW

>tr|A0A1H0G108|OS=Prevotella communis

MKFQLHQPQAINELGNRSNQEDAIYPVMGEASADQRVFVVCDGMGGLDKGEVASAAVSKALGEMAKAICDVVPKFSDDDFRQCLTKAYNALDAADTKKEATMGTTMTFLCFHDGGCLVAHIGDSRVYHLRPSLGTENGVLFRTRDDSLVQQMYENGEITYEEMRTSPKKNIILKGMQPYQMERTTATLTHITDVKPGDYFYLCTDGMLEKMDDKELVSILASNESDEQKRQRLIDATVNNSDNHSAYLIQVKDVSEGTAEIQQKRKPVTTEPVDSNQISSSVILGFSALSLLAGIAVGLAVFM

>tr|A0A1H1A513|OS=Pseudomonas sp. UC 17F4

MSPLSIPELSVAIGTSIGSVRQRNEDRVAIAKVHGANGTEYFVTLVCDGVGGSDMGDVAASIALAVFLDELVYTSIRQPLEVLVPRLVRRVDDRVRDILQGRGATTLSVLVAAGDGDIIATNVGDSRIYAWSPQKDKLMQVSRDDTLENELSEFALKDPSALRLRGLGGSLSQAIGETGRTSDDLRIKLLGRELFPDGAVLATDGAWKGAEDGFNAVVKKAPTAANVVTRALSVANWSGGIDNASLIAVERISATLSVAYDFSITSPKVSVWIGNTKLVLVDSSFSSPGRYSIPEVKDKPVMKADIRRALRKKYVKAKGEPKTPEQLDFKIVEDAPSPRKKDPRADLEISIEPTPKKESDS

>tr|A0A1H1GW05|OS=Halopelagius longus

MDLLPLLDELQIIARNGLTYDGDPHDSHDKERYKRILDLVSEGYGQSLDLPTQEIRDRLSADEFGHITPKVGAAAALFDENGQILLVKQPSLKTAGNGKWVMPGGYVDPGEQPVEAAVRETREETGLKVRPVSLVDGYWIDAPTKDGPHGAVTLLYYCHVVGGDLKACNESQAVRYWSIEEVPSWFTYAYEGAKDAYEIWLNSNFGKAIKANKTLSQPRTRVTSDPGMGVNEDVGGVTWKGAWVLDGATGISDENFTPGRSDAQWYVKEFDNILRNTINDSSRDLRTIVSDGIQMVSKKFEKMTDGYEIDRATEPSAACSIVRWVDGILEVYILGDCSVLVTYGDGEIQHLTDERELLRRLEREAVNGMHGLMTERGLERTEAKNIIWPKLEHNRQKLREDREHWSLSLDPKAPESGFYTRIDAATINQIQLLSDGVGTLVEDLSAFATWNEFAEWITSHGHEKTINRLRSIEQEDRACEIYPRIKPHDDATLVSVDFK

>tr|A0A1H2D1V6|OS=Actinoplanes derwentensis

MVGGWLRREPKKAPEPEQRAPDDAESDTTTWGGWDPSNRPEPWDEQTRPVRLDGGAVTEPVQLSPGNSGVYMSRRHQDDLPGDDDEPTTVFRLEQMTGSASGPRTEPHGNTDDQYEDQTVLHLRPVGQHEDQTVRYVREDQTVQFVREVGSHEDRTVRDTAEVDQNQDHTVQLRLGADPQQDLTVQYRHDAAPPQDQDQDQDQTTRYGLGAELPEEQTLQFVRAPVRPESGRPENGQQSGSAPDQTDREPETTVLRLAGDGSGGSTSPSEVNRTDQADADVTAVQEAAPDSEVTTYLAIPVEPEIPVEPEVTVHPDVSAYQEVTAYPGVTADPEVATLPEVTADPEVAVRAEWERHPNERWVVGEVGDQAAVRPRVPHRFRNPPPDTTIDGAEVGEVAYRAASVRGISHQERGEPRQDAYTVQFTQDHEWLVGCISDGVSSAARSHEAAAAICDRISRALVEHLLDSPPSDDPERWEEEAGAVPWDRMVRAANDAVCELAWPYVSSAAQQQGTRLDPAEPVSHAQAREIMSGTALAFVVATRASPSGVHRAILADIAGDSAAFTLQDEQWIPLTRSKAEDEAIFSSPVKSLPGAAEVDARSFYLRPGAPLIMMTDGLGDPLETAQGPVSRFLADRWRTPPDLLAFAQHLGFYRETFTDDRTAIAVWPGSEPGSEPGPGPGPADDDTADGGQRQ

>tr|A0A1H2ID13|OS=Desulfobacula phenolica

MKEKNKIKREHFYTYLKNWLGQSVSCGENSITIHNKSISLKTTIGNNRPKNQDRAAFVIITNHFTNKKNLAISILADGMGGMVSGEEAASSAIASFIAYLSLNVHNSGLKDICRRAIEHANVTVNKLLNDKGGSTLSAIVYSENGCVGVNVGDSRIYYFDQNNGLTQVSKDDTILGQLDNNSSENWEDPTKGDNRLAQFIGTSDELIPHIIDLTEYSKKITDSFFILTTDGTHYIGNNMMERIIKVSDKYNTVANKIINIAEWLSGHDNSTLVICPNKITVNRLKTDQNSEELEQITIYDFDRETNYLVPIEFENFYKENAIIRSDDEKKMQYKSEALFLNHQSKIEKEKFSKTQKKVKKKPKKDDKNDLIVDLLPPEKKG

>tr|A0A1H3ASA6|OS=Marinobacter mobilis

MAVTAPDAPSPNQSLPLPCASAQVTGQRQRQEDTLRLTRFDGGPAGPGLLAVICDGMGGHVHGDMASALVAERFVASFLAMDGSAPDRLRGALSASHDALIEATTRDPQLDGMGTTLVAACIVGDQLYRISVGDSQLWRLRDHQMERLNDDHSMAPVFDSMVEMGELTPVAAREDGKRHALRSAITRKPIHKIDGPATPDRLLPGDCLLVASDGLDSLDRDSLLGILDDDTESPQHTLDRLFTHLGELNAPQQDNTSVILISMPATTSAADSVRVDTPANAPAVNAPGTGFPTVNSVLAGALALVLILVFLWILL

>tr|A0A1H4AYZ8|OS=Pedobacter hartonius

MVSYLFGITDTGMVRDNNEDVFIAQEVMDARFLIAGVIDGVGGYEGGEIAAALTKDAVLTELTVIGPDVLTQLEISFNLANEEIYARKLEDKQLANMACVATVAVLDRQNNLLHYIHVGDTRLYLFRDNSLIKLSSDQSPVGFLEDSGRISEEAAMQHPKRNVINQALGLGTQDEMAESYFETGSSPFLPGDLILVCSDGLTDMVNKEQIMAVLSTSAPLKAKAEKLVAAANAAGGNDNITIVLAKNDKVPVVHPVLRPVADLFSQGTHDVPAPKAITKENDPVVKENMAAEDKRSEISSADKGAEDINAMPMDNSPRIVPVENDNVEIRPANRQNNPFLKQEHAAASGSLAESAHGLPKSNKGLLILLSVLCVLFLASTSWLFFTNPPVKLKGSGSLNAVQAAGPNRQEKMISDTLSKLKGDTLTLSAALFKGPVRLSRSLTINRDTLLIKTSGDVIFQRDSVYKGPALVLSPVCKYIVIDGLVLENFETGIISYKNVLDLKNVRFNNCKYPLQVLFEFPDHSYVNGRVSKRAFQADSLVKK

>tr|A0A1H4G5M5|OS=Thiothrix caldifontis

MLGRNGGFDIGYATDVGSRGNQEDNLGFRQYEDGSVLAVLADGMGGHTGGEVASEMAIQWFGEYFPQTHGSIRARLEQTLRQTQQRLCLYAQEHPELQDMGSTLVAVFVQGQDLYWLSVGDSLLYRADGSGVVQLNATHTVAERFRRLWESGKISRAELDNVQEPHALTSALGPDKLQEIDCQQATLPTDAVLVLASDGLLSLNPVDINTLLDKQASAQILAERLLMATLAKRQQGQDNISLIVLKHPVLPVTQHRRRWPLILGATLLLGAATGMFYQWHQAEQRIAQERQERQEAEQRADKQRQALAEEKRQRMQAEQEAAASRTLAEKAARRADEAEQQRKAAKKRAEKAEHDKKRAEAAANAHAVPVAPAVPAAPVKNPPPPSAPAPVPTPKPRPPPAEPVQELDKRDIFPDAS

>tr|A0A1H6ADJ7|OS=Bryocella elongata

MPAKIEVAALSDMGCVRTNNEDNFGYDPANQLYVVCDGMGGMAAGEVASAIACRTLIEVFAAQPEPTAVDVRLFTAIRAANDAVWAAGQLPEHKGMGTTTVVLVVRDNRLLVGNVGDSRAYRIKAGKILQITQDHSYINELIRMGTVSEADRHTVDLKGMETVITRAIGAAATVEPDFFGGDLTPGDMILLASDGLTRYVDKEHLTELIGNDSLEASCQRLIDTARSMGGADNITCILVRYLGLEEEPAFAPQGNVVEPTVTHDFEPAIEHALELAPEPAPPVPAFVPEPDVAEPAVAEPYVEEPGYEPVLHTGMDEPAPGPALESAAAVAETSLDMPAFSLVFEPESAAPTEPTDPSLKQGSTESEDEHEAITPKPVNGEPL

>tr|A0A1H6F450|OS=Candidatus Venteria ishoeyi

MTINWEIQQAISQGGRNSQQDSIAVLHSEDGERHLLILADGMGGHKGGDLASQEVVKVAKAEWLACQQGAEIEKPHTLLKRIFVRAHEQINALGKAHQLNPPPSSTGVLLYIDQKQAWWSHIGDSRLYYFRGKKLNHRTKDHSVVQMLVDLGRIEESQMATHPDQGRLLKGLGGGSKEAIEPDFASSDSSSGDLFVICSDGFWEQIRPEVMTMALDYKNKDLSSSLQVLLNKAKKKGGSDGDNLSVLTARAPGNSFNLKYLLSGLSLVLLLSLLGLWSIFPATEKPQEQSKGRHADVTEPHLQVPAQAQAQLKPITTEIKSSSEIPLVTEKMESVEAKPLVSNSLISDPSAAEIIMDSAIDVPAPEYLTGNSIDNKAMQASTAKEIENPQKAANPVYQVMPEANKNTEILQNTGQESEPITKPAKDNSNKPVQTIPAPVQVQQNQPMLSNMAIEINTQHQKALHNKTRNTPTTAQDLVQAEVNTHPDTLPSASACNFTYEQNRNRILCYKSVLSRAPGNQVVIQELNKLFAYFQGKIDFALKENKLDEAKTLLKYQDEILSILPAMAIDDSLRFDVADKKNQLNTQLQQKIILEKQEKARQIQRKYQQLRQEKIHQAQDEAKWQADKRQALEAKRQADKRQALEAKRQADKRQALEAKRQADKRQAQEAKRQADKRQAQEAKRQADKRQAREARRQADKRQALEAKRQADKRQALEAEKKQQEQDMEELVFPGF

>tr|A0A1H6ZCG9|OS=Deinococcus reticulitermitis

MTSDDQTPRADAAPNPPSGAELQPAPGTAELTPPIVIQAAPVSAEAAPLPGAVEGGSVEHRAAEAALPADSSGAQLALDPTPAAQGTADDPSFPVIATQVTEPGQSGPVEVQAVPAPFTEPLRALFGEGPSTETDTGSTPSLLSSGAEIPASAPVEAAAPAPVAPVEAPLPLPVEAPSTPVTARLEGISDDLEEEIYAPSVTAQGPQVGDELAGWRLTADLGRGWFRAVQVATAEERDVYARPAPLWAELRPHRLLPRVSAAGELTTLEPLPEEVAPIAPPLQPAEALGHLTELARLLFALDKQGFSVTDLDPQAARLTRDGLKLRFPPRVVRSGETEQAAPRDGFTPPELLAGEAAQPASGVYVLGALLYTWLTGQPLPAEGPSPSLLGAVATPGMPQLLTRMLAPLPERLSPEGLLAALRAQGAGPLPSYRVIARTTVGLNPDRPVNEDSYGYTLREVDAEGGRVLRLRACVSDGMGGMAAGEVASRAAVEGFLDSAQPELADQIWDANAAVLAAMAGRDGGCTISGIQIDGDRMRLGHVGDTRAYLRAEGEVRQLTQDHSFVAAMVASGQMTPEEAQVSPERNKVLRSLGSLRQPQPEYVQTLPEPLTVRPGDRVLLLSDGVWGEIEPGRLTAMLRDEADGEHLIDRLIEMSLEAGAPDNATAMLIERGR

>tr|A0A1H7C883|OS=Variovorax sp. OK202

MPDTMDRAIRSFAVPISTSQFSCVGERSGNQDAIGYHLEERNACFVVSDGVGGNAGGELASRIAVDTALNTFVDDPSLGADSIQRCVKSANDAILAKQRELADQSRMSATFVSLFVDRDTNQACWAHVGDSRLYWFRRGALMQRTEDHSLSERSGDAAASQHGGERLVKTNLLYRALGARATAEATITAPQRLADGDAFLLCTDGLWQLISQQVMERSLQLADSAEEWLALLRRAAEAKADESQDNYSALAVWVGFPQQVTLAGAAAPQRAAV

>tr|A0A1H7GM49|OS=Roseateles sp. YR242

MRFSVYQVSRRGGREKNEDRMGYCYTRESGLFALADGMGGHPDGEVAAHMALQNVAALYQRDAQPVLPHPTQFLETAVLLAHQQLLAYAAERGMSDTPRTTIVVAVMQQGQIWWAHCGDSRMYLARDTELIARTRDHSYSELQEALGRHATGAERFNRHVLFTCLGSPGKPMIDVNGPVLLQNGDRMLLCSDGLWSSVSDADILRLLSDARAVGDAVPELVEQALRQAGARSDNVTALAVEWEGEGDAPDSISTQDLDDQGFASTIQGAGQEQLDEAEIDSAVREIQDAIRRAGKKPPR

>tr|A0A1H7MK91|OS=Nitrosovibrio tenuis

MSRVEAHFGDVYIVSDGMGGHRGGAVAAELATEILGRTLSGIRSISSAAEVVKAAFQEANRVVYGRGHSGDDDTREMGSTAVVLLIGQSQAVVAHIGDSRAYLFARNELRRLTKDHSRVQRMVDAGILTDAEAASHPAANLLERAIGVAPDVEVDISSPFKLNAGDLFLLCSDGLHGYVSDSEIAAILNRGTPVQALVDELVDRALEKGGEDNITVQLIGYVYSAQGRLTPIMG

>tr|A0A1H8RGH4|OS=Actinacidiphila rubida

MSQQGDDHPGQEDDWWRQLYDEDAASDALRAARGTGRSIDEHFDSAVDAMSPPPPPRSETMRLRRPTAPPPPASWPRPTRNVPPAPRDPEAPPPAASGEAGGPEAGVPAQAAEREGAVPPEPGRRHDVSLPGARADEPEAPGGADGPAFGPDGSPDADGPRGPAVPEAWFRPEVRDEGGAAGGSFDPGGRDGLGEHDAHLPWGGGVDPGDPEGLNGIGAPDGLAAVPEPGRERPLPREERAPWDPWAAPPTFGPPPEVPQAAEPEPEPEPRPWDVWSAPPTFAEPAPTPPEAASEAPPAAESDEVPAPRATHEPPPAQPWAAWVTPRPGTTPPPPSAPTTPSAPAAAEDWSAPHEPPSGTDCTPSAAAAPPAVSPAAHEARAEVPTAPEAEPRAGADPGFGALQEQHAEPEAPAEGGFGPGPVQERRAGPETPAEAGRVSEPYAEDVFFAGPEPYDGPQPYGESQDRPYAEPELTAEAAAEAYAGGEGFGGPEPYGSSQHRAYTEPEVAAEAGSAPGTSEDPEVPAGIGSAGEVSQEPFGASELSGEDAGPSAGPAADSPPEVAPDAYAEPLAEVWSPAEGPHEPYLDEPEPAVEAARPVPESFADPEPGAFAARGHQAHVEAATQSEAPQAGDPEPEPGSPAGDGAGAVQPVEAGGEASDDGGRAVEARALEVEPPRPVGLFEVPPTVTAEGVEPVVPVSPHIPFTDAPPDSPRRTEFVEGEVRWAPELPPGWVAADEGRRTERSDDVGEPEVVGGGPPTYGAEPTAWPEADPDELDGLVPDTVLDGARYGRLTLRAVALKGDSARYRGQPRRDALLTARFGSGDDGLLLVAVASGARGADEAHQAAQDAVRWIAGAVGRSRSRLAGDLRAARRGALKSGLHRLTDRCYGRLRARGEELGLPEGDYTASLRCLLLPVDPECRIRLFFGLGEGGLFRIRGGSWQDLDLAPDEVAAPFRFRATVAQPGDALLMCSAGLSEPLRGEPELAVHLAERWGSGTVPGLAAYLSDAQTRVKGYADDRTAVTVWDA

>tr|A0A1I0ENX5|OS=Thorsellia anophelis DSM 18579

MAIYGKPASEEKKELENSSVLTFNTKRITPDFRLLNGKQNEPYCQELIFDTENYLIEIIDINVTSCAGLAFDADSLKVMGSPLHNGEFDIVVTYYDKNDGFYENGPLENSVIDNQNNASIEQESAHSESILSDTDLYKTSVIKLYINPDPRNLWQNLPSNPADDYYKPDTVTKLFKVLDSDKSKNFVKTLLAARIRGRSHAHNGTCCDDDFFINRLQNNWHLTVVADGAGSALFSRKGSALATYEAGCSLTDKLEKSSYSNRLEMVVTNFIKQLSLNAKTEYGEENTNTAYWHSKEFENEFSSTSNDEAKLSGKLTLEHNLLLDEVIELVGGAAKAAIASHERFITTPEAKINNITLQDLSTTLMIVISKYIEGRWFIASFSVGDGVIGVFSEGQGLQILSSQDGGEFAGQTHFLTEEHILPESLRLRCQYMFIEEYTAILAMTDGVSDPIFETDNMIYDLSKWESFWSEIQANVLNQAELEFALAKYLNFWSKGHHDDRTLALQF

>tr|A0A1I0JW44|OS=Enterocloster lavalensis

MKFDRSEGLQEESTAEVKEDFGTPPSPEKHYEALADISVYGMSLQGSSHVERCQPCQDYSDFRFIPEAGLLISAISDGVGSCALSHWGSYTVVTTVLNVLETELKAELQTKKFTEIEGKTIGAILRKAFLESKDAVEDLADHLTQPLPNFYSTLTAAIYDGSILYYGHVGDDGIVVQLENGTYQLVTTRHKGEEANSVNPLQAGSPDQLWQFGRVSEPVVGFLMSTDGVLDQYVANKQRNNRIFYPFLENALYSMAGQEGKSGEKIVQEAFEGMRFTLDQPEYRSKVTDDITVLAAVNTKRLQNAVHPAFSVEDWKAEEEEYRREIQKKLYTGEPVGQSRPAKPQTGKKADSWQDQRAGRQTDQRQNQRTGRHADQRQDPGTDRQTDQRQDQRAGRQTDQRQDQRAGRQTDQRQDQRTGRHTDPWQDPRAGRHTDQGRDQRTGRHADQGRDQRTGRGERRNDKDQGGVIDVFYVSAKKTLDSLAEFLWEPMEEPKMCYCPKCGRRYEMTRGNSYRFCPHCGSKTRPLDGDQFW

>tr|A0A1I1B2P9|OS=Nocardioides alpinus

MSTTRPDIALTECEFDGRVVIGVSCRGLLHIGHDVVRQDSFGILALSDGRLAVIVCDGVGSRPDSHHIADAAVEAASAALSEGVSPDAAARRVNNSLLALRYRWDGATTFTLVVLGSGESSGDVEVVWVGDSPLMTLYAGEWFNRTDDAFTTRDQDDPDVSKTKALPSQDPSIQSSAFRLRPEESVFVMSDGVSKPLLGAADVRRQLALWWSNETDIYTFASQVEFGRKTHVDDRTVVAIWPSASLETTIPRADQELTELDLRVEYGEGESAGDSSERLACATPDTDAEGDLARASAQS

>tr|A0A1I1ECX8|OS=Streptomyces aidingensis

MPREEFRDNAIHALVVAGALLALVGLAPVGAWAALFLLLAALVSAFRLGLARRPAAGLAASAAAPVHQVTAEPAGGHGSGAAGGPGFDWPDKPRRPGAASHPPTMADPGPRAGSRPGHRAAAARRSPSATVALSAAAGDAAGNGCPPPLVFGGGSRAHGAPWLLPRRPAQPGVTADDATLGGLGVLAASVVGPDHRCAEPAKARQDAYRIARDDSGAHLVVAVADGISACRYSDLGAQIAVTTAVNLLVEELREGGDPRLVDEFRLYREVAERMRATAAGRGLAEREIGVVMIVAVIAAEPEADGDRTLWLSWLGDVSAWQLTGGERWEWLAGEGKSGTGSMLQEQEIHAALPWAPDAVRREVFRLPAGVRLAFLTDGVGDPLAGSPEFNAHLARSWARKPSLMSFLHDMDFDARTYLDDRTAVVVWTGP

>tr|A0A1I1F683|OS=Fructobacillus durionis

MAIAYQSDKGPVRPDNQDAVGAFYNRAGDPLVMVADGVGSQKGSKKAAQMVVMALGRAWEQVAINDEQKVKEWLIHQADLANQSILLVGQHDQGISQMATTIVLAVCLHGKVLVANAGDSRAYLLRKGQARLLTFDHTLKNELERQSGELVDTDLPDADSLTRYFGVNKEVDLEWTTVTPEKGDWLYLTSDGFGKVLSIKEQMDFIQPGERRPGNNPILGSVLDISERLSVLTTAAIARHVPDNVTALLVTDMAKSEQPSGQRLAMKSAEVPAKAEEIILKDGLKGRLD

>tr|A0A1I1FY62|OS=Cupriavidus sp. OV038

MRFSVYQESRKGGRRINQDRMGYCFTRDALLMVLADGLGGHALGEVAAQQALQTLARQFQTQARPAIRNPADFLQDTVMLAHREIHRYAEANRLADVPRTTVVCCLIQNGQIHWAHAGDSRLYLIRKGTLLTRTRDHSKIENLLQQERVLPMDVANHPERNKLYNCLGSPNLPLIDIGGPVRLEPGDVAMLCSDGLWGALEEKVIVDRVTSLSVVHAIPDMIEQALQNAGEGADNTTAIAMMWEADATVPNDDAVLTDTLPLNAFTTSILERTGNESDLLSEEEIERSIAEIRAAIDKTTNFMR

>tr|A0A1I1HXG2|OS=Ruminococcus albus

MELEYSFFTNEGDRPVNEDSIGFTEKDGRYCFVLCDGLGGHGKGELASRYVVEYAKCFFDESTDNDSYMETVLDNAQEGLLAEQIELDATFQMKTTAVVLTAVGERCRYMHIGDSRLYRFRKNKVMKRTMDHSVPQMLAMSGDIKEKQIRSHPDRNRLLRVMGSKWTSGGAKYELSETEDLKAGDAFLLCSDGFWEPITEKEMCKLLKKSSSAEDWLRKMAETVRENGKDTNMDNFSAIAVCVKG

>tr|A0A1I1KCL4|OS=Tropicimonas isoalkanivorans

MKIPDMVPGMPQRFKGFYEAASAVSVGKRACQEDAVLYDCPLGGDTGLVVVSDGMGGHAAGDVASKIVLTEVFSELKLQSGASAPEEGFRALLTRAAEGADDCIRAYIQDRPESIGMGATLVAVVLHGQQLHWISVGDSVLYLYRNGALHRLNEEHSFGRKMDRLVASGLMDSEAAAEHPDRNCLTSVVGGGGIAQIDCPADPFQLADGDLLVVASDGLEYLSPEEIRTILGDWHAVSSAQIAAQLMRGIEMIDDPEQDNISIVVVKVRLTDTAALRGPKLKSKPPIVPTRPATMVAMKSRAPAGLAGQRRLQCDQG

>tr|A0A1I1MPP7|OS=Massilia yuzhufengensis

MQFSVYQQTHIGGRKVNQDRMGYSFTRDALLLVLADGMGGHLHGEIAATIALQTLSMMFRMQAKPYVKKPERFLEEALTQAHWDILAYAEAHKLPETPRTTVVACLVQHNCAVWAHCGDSRLYWVRRNQVLARTRDHSHLEHLIERGKATEADRNTHPDRNKLYNCIGAGQAPKIDISRQASLEPGDTLLLCSDGLWSMLPETEIVHRLSSSTIVQAVPDMVSMAAAIGGARADNTTALAITWQGAEGAGAAAPGMISTQMLPDDAVSSTITAGTPLAPGSDAFDEDEIEKAIAEIREAIEKSSQLLKQGTP

>tr|A0A1I1STK5|OS=Nannocystis exedens

MSAPMHSSSPVLEGLRLRAALEPRRADCDLDLGQNYTAGSLVDAGERHLSRRQGGQDHAVALRHRGAVALAVADGVSRVDGHPSQTEVGAALVAELAARAAFDAALRGQSFVEARTHVATVLVRQLLPLWAVLSPNAGTFLHCTLVLAVTTPAWTAIWLVGDGAWGASGSLGRGRSPSSPVSPVAIACYGRRWSAHGREHKPDSPMTVANLCRGGDVEAVVSGLEVVLEAEGPALSLYVATDGLQQEPQADELLRRDDWRRDQLTAALVRPQDCDDLAIAWAHGRLNSSEGTA

>tr|A0A1I1Y7H2|OS=Nannocystis exedens

MLYLLRPVRTRHHGDTDVGRRRSQNEDAFVASDELGLFVVCDGVGGRACGEVASQTTVQLIHEWVDREAELIQYAHEEARLARKRVVHGPPVVEPQIRLSPDTVARLGGLVRSALQNACYLVHGMAEVDARYTGMSTTASVVLVAGELAIVGQVGDSRVYLARGDDVRQLTEDHTLLNMQVKQGLASADKARGRKSQITRAIGLREFVEVDIMAFPLQLGDRLLLCSDGLHEYLDQAADTLIDLFRLHPRVAAPAAIRYANLCGGKDNITALFVELIEGGRDR

>tr|A0A1I2I6I9|OS=Nannocystis exedens

MSRRSRRLTLTPARAGCAELLAADDAALPALVPLDRVSGRPQRWAPGPAPYFRDERGLRTRCAAATDVGRQRVHNEDALAIEPKLGLFVVCDGVGGRVSGEVASALAASTIREWVQREATRLAAVARSPGDAEAVASVGVLMHDAIQGASRAIRGLARSDPQHEGMCTTATVLLIVNDFAVVGQVGDSRAYLGRGVGVYQLTEDHTLHNLQIQQGLLRPDSARGCKSPITRALGREDAVEVDIGALPLMAGDRLLLCSDGLHEYLADDELRELLRLDIRDAAPAAIQHANARGGRDNITALFVELVAG

>tr|A0A1I2KTY7|OS=Actinoplanes philippinensis

MEFTDATWFVGPRRLTVTAGTVVGNRYPANFDVLHLDAARPLAVVADGMGDGPGSTAAGRTAVEVFLRETTRETGRAGMEEPATASGPATASGPTTASGPTTASGPTTASGPTTASGPTTASGPTALRAAVAEVQRAVREAGRAIAGLTGCTLTAFVGDTDGSAWLVQLGDSRAYRLRDGVFELLTSDHTAAWLGLLNAWYAADSREAYRDRHRLTRFAGHPGMPEPDLINVSLRPGDRFLLCTDGVSDQIHDRRLAEALTERITVTELLAETLDHGGDDNATAVMIVVS

>tr|A0A1I2T2T5|OS=Halobacillus alkaliphilus

MKERKTNIEHVNYTSPDKKESEDAWIINHEAAIYGVLDGATPLDEFQDEKGHNGAYLASRIFKEHFESLVPGADLTEEVRAANSKIYQQMLSYGVDVSQGYMRWSTCVAIVQLEDTHFNYVQLGDSIIMAGFKSKPAELLTKDTVKGISSRAKSKRATDRTNGVEVLDERYFDNKLNQLRYNRSLANQPDGYTVANGMKEANLFLQRGKVSCEDLNDVLLVTDGLFHPDYDLLDSYKQIKDMGLTPYINELTKELEERSMPIDDGTAVHLTFSSSNSE

>tr|A0A1I3CPH8|OS=Nocardioides psychrotolerans

MTQTPAPTPGPTPGPGAQTCPACASAMPAGTLFCENCGASLGTPAPVDPSARPVSLQGAMPADGPSPIDEAAPISAATRPVPASVPGSVAATVPGPVPCRSCGASVGPDGYCEQCGTKAPSERDHFREAPADWVAGVCDRGIRHTRNEDAMALLASPVAGERALLLVLDGVSNTDDSHIASLAGARAARDVLRTPMPRGLGTPVARLAAVTKVFSDAVVAANQAVVASTPEGSVNPPSATFVCVVVEGGTLSFANVGDSRAYWLPDGAPGVQLSVDDSVAQQMIASGIPRAQAETSPQAHAITKWLGRDAGDITPRVGELEVVGPGWVLVCSDGLWNYASEPEALQEQIRLSGARDPATLALALTDFANAQGGIDNITTTLARVVAPQEETHG

>tr|A0A1I3D3C8|OS=Selenomonas ruminantium

MAAMGMYSLLGIGALIVVLLIVRFWPEKQTVAEEAVPEKNEPPRNPIGTAATIGQRQLQQDLTGSALGDAGGLLLLADGRGQAGKIAAKLAIDTCLDLYQECQAGEKPQYYFRKAFQAANHKILSVLEDGRGSTCLAAAIIERSRLYYALVGNSRIALFRDGDLVPVTEGQTIDILAQHRYQQGRISKEQALKLLHERRLYNFVGQDGFHDIEFFSEPIELQPGDVVVILSDGVTETAPWRKIEDCLSLPITPQEKAQRIIALVEASKREDKDNASVILYDLHDPAAKPAGKKQGNKKKGSMNLEKIKQRLSDRLCV

>tr|A0A1I3V0D8|OS=Jannaschia pohangensis

MWRSPEPRYDAASAIAQGGRSYQEDAIVTDFPFGMDSGVAVLADGMGGHAAGDVASKIVVTEVYSELKFQSANFAEFESEIPSYMKAAAEGANQVMREHVEDHPETHGMGATLVSLVLVEHRMYWMSIGDSPLYLLRDGKLQQLNEDHSLAPQIDFMVEQGLISREDAQNHPDRNCLTSVILGGRVARQDCPSRPFELKMGDIVVVSSDGLQYLDNAKIQKLLYKYRRKKAAEIAGYLLEALDDLGDPEQDNISFSVIKLNHMKPVERKIVAKPTGLVEADRMLTTRVAVLPDGPKGPDPKEETPAEPVVAASDGQVPGPGEHLETYSYRGQSYTRVVPDKTAETTEDTSEAEADAIDPGLTATETTIPPGAVSLDTVEDDAKADGAAASDEDDDDDEGDRKIAVGA

>tr|A0A1I4J3L9|OS=Porphyromonadaceae bacterium KH3CP3RA

MAPGGRTFYRIRQNDPGHAHGKIYRANTGLTDGYIFLVSFRDLDCDNSSKKSNFAFGEKIITMIDENRNITFVHNSLQGSEREKNQDDVLILSDSDFCLFVLFDGVSSLSGSWDFVQACKRFIKENHIQYVSGNKVNLKELIYQMHVASFDAGENGKSTCSALLMKSGDNKAYVVSIGDSRIYSFTNSYMEVLTTDDNLSGNSNFLTRYIGLEGLRPDDIDLIQVNADQNFLICSDGFYSLMEKDLKTYFKIFHYKRKGSIVNAISRLQKNKNKDDSTFIIIRNGRI

>tr|A0A1I4SFX7|OS=Marinobacter zhejiangensis

MIAVWSAAQIQGTRDYQEDRYGVVENNAIFYRGKRYPFEPGLFPAHYSLYVLADGMGGMGHGEQAASTVVEAFIETFINLGNDALPPMERLRAALDEANQAIARIVAREPDKQGMGATLIALLWDRQDSTLNWLSVGDSLLQRYRDQALTTLNQKHTYQQLAQRHADQGDANRAAELAALGNTLSSAVDGQPIPEVDQPDQPLPVNGGDLIILASDGLETLSDQQVSTTLAEAARAWQSAPGAESSTDALATCRDALFDQLRLAQSPYQDNCTLVLIGWQANLPDQTSDHTQPPPTESRS

>tr|A0A1I4YUI7|OS=Pantoea sp. OV426

MKIVFASRCQQGLRDENQDRTGAELNEHKACFVVCDGVAGLPGGEMAAQLVRDTLLSQIKAHDDFTPEMTRQAIEQCRLALQAAQGKNPKYSRMSTTLAALFIDRQKQRAWWAHAGDSRVYHFRRGALHEVTRDHSLAQQLKDAGYENTGINSNLLYNALGAEPSRLVSFSQEIGLEDGDAFLVCTDGFWLNLTSDEMEQALRMVNACEEWLALMEQAVSRNVKKDNLSALAVWIGEPEEATLLYSLADSARFLPPRF

>tr|A0A1I5F700|OS=Roseovarius lutimaris

MTSCKTPAFSFDTASAISQGARDYQEDALISDFSNGAELGFVVLADGMGGHAAGDVASKIVVTEMFSELTFMRAEMVASKTCIGEMLRKAALVANETLKAHVTQHPETKGMGATLVATVILDERLYWISIGDSPLFLFRDNTLRQLNEDHSLSRTIDTMVETGVLSAQDGENHPDRNVLTSVLFGEPIAEIDCPSEPTELRAGDTLIVASDGLQFLTDAEIKTLLQDRPFSRSSEIANALMSGVQNLNDPDLDNVSLTVIQVRHSKESVSGMSHMRQRGPQKPEKQSLFQKVFRSLPAEAETAGLARD

>tr|A0A1I5M8G9|OS=Pseudarcicella hirudinis

MIKYNYAPQTAIGGRKENQDTEGSFLTKFGLLAVVCDGMGGAAGGKTASSMAVNTIIEEVINSSGSMPAQALATAIKKANQEIFNRSRQQPQLRGMGTTVTAILINEEKAIIAHAGDSRVYQLRDGKKVFRTNDHSKVFELVKRGILSEEQARLSEESNIIQRALGIAPDIDVEINDNIPFLKGDLFMLCTDGVCGALPEEDLLLLLQKEKNVKRLSEQLIQQIDFLGNQQGGGHDNLTVALLQPLINSKLDVIIDMKTKITIYALASMVAILSAALIYLLLFSDSRAKITQGENSDLKKEKAKIDSLQGVVIRQANILSGKNSDLKKEKTDSSENKGISVKKQADAHSGKNSDPGNVNKSAKVKKEKDSTPKTGQPKSIEENPVTPKEKADSSEKKSPGSSNIW

>tr|A0A1I5Z7P1|OS=Hymenobacter arizonensis

MLLETLSFISFMTNSGLSFLAGSAFTVAMFLMRNEKQKRLAQARQPIAEEAKQQVLVEPQKESTLVSDVPAAPLSSGLLPKVELLISAGPRKSTRETELGEDVAGSVLTPNRVIFWVLDGTSDTVVIPGEQGRDLLSSRLFCLAISAALHQLAHKFTDAHKLAEAALDEAAAQLKSLVAEHQESFQKFGKSEPEQTSHWDVSTTMLVGILSDTGEVDLFRIGDSKAICFGLDKQVVATVVDQKPASIGLGRIHARLVRGELFTLQLFTPDREDQLHRGRASGVRRIIAFSDGVGPSTEQYLRRMLAQRTYSQFHDELGRTPNKTFDDKTLVLASFEPVS

>tr|A0A1I6LH58|OS=Halomicrobium zhouii

MDYATNYDVGDRKRGGGINEDSVALSVFEQGHRSGVTADGTDGNGIGRDGAGEPDESEGPPANRSAAVFALADGAGGHDAGDVASYLATTVVCEELAPVVAEAARRDPGDFAVDIDESLLPDRPSATDLQTAVADAIVAAHRAVVAYAAESGTQAYTTVVAGVVVDGEVHYGWVGDSRAYLVNGARQSIELLTKDHGVVQELHDRGEIDDVAAHVHPRGNEITRALGGAGDGDPETASVPVETNSVPIFAEDVLLVTSDGLIDAQTDAPALYERYVEAGRSDEAAEVVRDAVVTDEEIRDLVLGSDSLVDAASGLIDLSNDRGGKDNLSVVLARDSALPSTPGEDEGMPVRAVDPDDPVEDRETVILPGE

>tr|A0A1I6MI86|OS=Yoonia litorea

MMSKTLAFRRRPEPVASFTGRPIKDRFDVASAIDIGCRLYQQDALISNFVNGDDVGIAVLADGMGGHVGGEIASNIASSTAFAVAKTSLIASRLEQEAIPSSLGAAVDRANAALGEKIARDPQLKGMGTTLVVVATIGPNLYWASVGDSPLYRYRDGQLKQLNHNHSMAAQIDAMVASGIMDAEQGRNHPQRHELTAAVCGNEIARRHCPEKPVTLTAGDILVLASDGIQTLQDESIRSTIYRNRKSSADDITKALLQSVHAVQDPEQDNVSILVIKVASDAPAQVVRPPDNEPVLVQTRAPKDEDRDVETASDLLNQALEL

>tr|A0A1I7JE38|OS=Paenacidovorax caeni

MAFETPYSFPGRSATPAAYDPFRADLQRIQHQIFPGLDKPPASNNGAPALTYGEHACGGWEIAWAQIVGPSHRISEDSLGYAMHCRYDAPGPDKHAPHGVSLVLADGVGGGARGDIASHALVRHCLAMGSLHANANATHPGLLLEGLSTADSAVNRSLAARTPLPGAATLAAAWLDSRAQGWISRVGDARLSIWNIHTGQLLPLLADQSYANLGELPPHPSHWSAPARMVGAGLMGAPEVHPLSLAAQEILMLSSDGLHQWLSSADTLPRQGVSGTRLADFAQALAMHARETGSDDDISVLLLRAPNPIS

>tr|A0A1I7TF76|OS=Caenorhabditis tropicalis

MPSSFIRKHVRGLLRNKFGPSTSLDNENQEEPTTPCGDNNSDLQHFLGGIDLRRHEFPEVYFGKTGLDLPAIQLETLDADVFASFTGPEGGLTEVGEVRQGHQMARMANIDDEMSLSIDGTTDEEAEDEEDLDAPISMNLKKSSRHSLLLGSCSSILGPNGEHHDFSAGRPSADDYDWSAFDDEGAFGMSVSLYEKNPLSGVNAGEPIADVWGVVGRNNNGVLALADGVNWGEGARLAARCAIRGAIDHLNARVIKDSLADTTEVFHEMLAAFHSAHSLILQEGGMLTTLCVSLVAPVKRGNGWALCVCNVGDSLCFVYNRHYGVREVTLGSHDIDQMRDMRDAGGALGPVDGRNPQLHNLTCSMTFVEEGDIVFITSDGVSDNFDPVVGKFCVIKKSEHENKENTHLRPREDRTLDVSTRGGDKDKNTEYARNRPCAASLPCVDAAKRHELMLVRMRDVIGNGFGNETPRGPSSEYQYPPVSASTLCHRLIHFATQLSTAKRRVLENPELYKKEKMSKSEQRARRKMVREKIADMPGKLDHASVVAYHVSRRGVDKEPATPPCGLQEQTEKSQLISTTSVSSSEAMDTESTVALKQENSTFTRVASAQCVEHLIGEPRGTDQVEMAIQEELPPPSPMRSKPEKLILHDLVDKPGKEKNNNTTVVSNGIENRPVSPYEQLPISSREAGKRRKKHGRGSIGRHTLGVDVQWLKKLVTKKEEQKTADDIRGDISLSSENADRTTLRQRIRSMLGSNRTLANTDQPTIKVSMRATNV

>tr|A0A1I8AZ45|OS=Meloidogyne hapla

MSSFFRRHVRGLLRTTFSAPQSTETISSIATNNVDDDFENHDESEEERLLLSLDHRLGPAGTSSETNNLCRNGKKRQNAPEVYSGKTGLDLPNIHVGPLSLPVMACHTGPDGGLTCVQPMRQRTIAKMAKLDDEEFSLSSMDEEVDEENEVEEEVMREERRENGEEKVEEEKHEENNGIEVNNQVMEEMNGFRPVDEEPEDIEHQTNNPPKSIGELKLLDVPKKETIKKNKKKMKRSRGGGKALIKSADSFEALGLVGVDDFPTFRNGSALEQLDWSSWDENRALGISTSLYERHPVSGLPAGHPIADVFGIIARENNAILALADGVNWGDGARLAARCAIRGAIDHLNTAIERQQLNTTNDIFHSMLGAFHAAHALILQEGGSLTTLCVALVAPVKASDSSVLCVCNVGDSLCFVYNQTHGVNEITLGSHNIAQMRDMRDAGGALGPVDGRNPQLHNLTCSMTFVEKGDLVFITSDGISDNFDPVVGKFCSIRRPREEAEEMEVTETVTNEKENNLTTNNQKNNASSILLPPPFHKTKSAPATNIENIRKPINGTHQHLNKPPLQNNINNYIPLPLSEMKRRQPAATLPFVDAPQRHELMLLRMNDIISNGLCLPYNDQQQNSPLEKRQISAVELCNNLVQFAYKLSSAKRHTLEDPELYRVVIFT

>tr|A0A1I8C9L7|OS=Rhabditophanes sp. KR3021

MTSFFRKRVNSLLRTSRWSISKDSPADAYTDDQDHFKKGKSCRYEEDKTSFVTECILNGKNGKKISTPEIYSGKTGLDLPVIHLDVLSLQVKASFTGPDGGLTRVREQREQPIVNMANLDDEFCLSVDEISLNEWNDEPMLEKIKCSHYREAHLESLGFVPGNVSPINTSNEYDWNRYDDKKAYGGSVSLYEKHPITGVNAGNPIADVFGIIARENNCVLALADGVNWGEGAQLAARCGVRGALDHINKHIESGKFQTSTDVFHSLLGAFHAGHALILQEGGALTTLCVALVVPVKNSSTSVLCVCNVGDSLCFVQNPLSGVREITLASHDISLMRDMRDAGGALGPVDGRNPQLHNLTCSMTFVEEGDIVYITSDGVSDNFDPVVGKFCTIKKTEEESEKLTPSPNFATTITNQIKNTIQNSVPPMLKKIPPRCNATLPCVDANQRHELMLLRMADIAVNGSDKSSNDDSDMIESDINQVTAKSLCKNFLEFVYNLTTVKRKTLEDPELYRVRNTHTRSEERIRRRLIKDMISQMPGKLDHSSIVAYKVGDWDGYSRSKIVFETGTTTSSGISSGSSQKSSVSPVSSTSSREEIDYDSVITSDVLGDCHELAISLTHSIDTNVSSMKTNRYSKESIDVTLIGDFTAFNKLADITPTICSPYEQQSLFTNCKPPSGATTSRNILNKENPVEPKERKKHRRNSLSRHTLGIDVSWFKKLIISNGSDKTNELNMAGKSTNISNSKVFLILAILGVYCFYDFYWKRKSLPPGPVPWLIAGNMPQLLLHIGDVDAIFQCWKNQYGGIFTIWIGPIPMVMVADVEIMKNYFVKNADVFSDRWVNYITDTFMEGFNGIVQIHGDKWREQRRFSLHVLRDFGVGRAEIEKRVMLEVERMIECLEKDAGNEAIDLHSYFAACVGNIIITILFGKRYEHDDPVFIELRDLLERQTQVVIRPVMGLYCVAPFTTKIPLINSSWKELMGMKKFLNTFLRKEVTEHLAKFDPSAEPTDFTFAYLKEMHERKASNGDMGYFSEKQLEMLLLDLFFAGMETTVTTLKWGFLNLILNPDEQKVVQTAMCDLPNIVKMEDKNHLLYLQAMMNEIQRVANILPFNLLRTTSTTVSIDGYTFKKGTMVLPMISIVMNDPFYFENPKKFDPSRFIDNNNGIKKYDGFLPFSVGRRSCLGESLAKAELFLVISNILKNFDLSIDPAKPMPSTKRILGLTCSPQKYKVLLTRRNL

>tr|A0A1I8GKP0|OS=Macrostomum lignano

MCSIPQSVSEQIFDHPVVICGSYRGQERAAETVWFGSSPEYVPALPLAALDSSVFAACTGPDDGLADASLAVYDRKPSLAGNGLQSTKVPGSIATDAEFWRQTSRKAFGSAISLYDTSPVTKRVSGDPIADCFGLVARGNSAVLAVADGVNWGEKSRLAARAAVRAGLMHANSYAALPDALPDTRKSLSVLLESFTAAHSAILRAQGGLTTLCIAAALPLARPPQFAVCCVNLGDTFAYVWSKSRGLRELTVGSHDV

>tr|A0A1I9Z5W5|OS=Nocardia seriolae

MLVICDGLGQHAHTAQIGAELFTRLCVAEIAAIPSAEVRDRGFEPLTEAANIAVGKLLRCKDILLGGIGAKQLSCTALICWLPLSHTDETEALFLRAGDCEAIWFSAAGGFEAVYPDADNGPLNLVRHTLPSPQVRDVLESARSGVNRDGLLILATDGLATDIFDSPHVRDWLVTQWARPCGAHRMLDSMRYRRQGSQDDRTAVVVWMDPPAYP

>tr|A0A1J0ACD8|OS=Gloeomargarita lithophora Alchichica-D10

MEIAPPVSDALPTQAPLRLANLEVAGLTDVGCQREYNQDYFYAHTVMHRRLSPQGELVQGKGLYILCDGMGGHAGGDEASQLATHKLASYLLDHWTEGELPGPEVIQAGVGVANQAIYLRNEEEYRRGKGRMGTTLVVALVQDNQVAITHVGDSRIYRITQSEGLKQITLDHEVGQWEIQRGTDPVVAYSRPDAYQLTQALGPRHEQTLELDVHYFTAAEDTVLLLCSDGLSDNGLVEGNWQQYLRPLLLPQTGLVHLQAASRQLIDLGNELNGHDNITAVLVKILIHQPRL

>tr|A0A1J1EU45|OS=Thermus thermophilus

MDRLEAALKTHPGLKRPKNEDAVGGEATPWGGVYVVADGMGGHRTGEVASRLAVETVLARLSREEPPSPKDLLEALEEANGRIHREAQRPENRGMGTTCTVLVLDLPYALVAHVGDSRAYLLREGNLHQLTEDHSWVAERVRQGLLSPEEARTHRWRNVITNALGSFPRPGWTSSGSSSARGTPSSSAPTGFPGSWRKGPSRRSWPTFPPPRRRSASWPWPTSGAARTT

>tr|A0A1J1JDW2|OS=Planktothrix agardhii

MQNPVAKQYLWTVGEGIEDQPSGTMIADRYLVKTNRIVVDTRPELLPEIPSEISDQISPYLKLFAYRLHIPQVYGIISPAKGGINKPIILLEKGPIADHGETLMPTLAALWPDATAMRQLNWLWQIAQIWQPFCRQNVASSLLNSHLLMVQGRLVRLLQLDPDIHNATLKQLGNFWQQWVPTAQPQIQKFLRQLTHQMISGELRTSTQLIQQLDQALSICGRGYHRQFEIAAGTDAGPTRSHNEDACYPNGDQPIEVPPSEPALAIVCDGIGGQDGGEVASSIAIDVLRQEVEKMPLHQANWDPLSLTPKLKRAICIANDVICQRNDDENRQGRERMGTTLVMALAHIHEVYLTHLGDSRVYWISSSGCHQVTVDDDVASRQVRLGCALYRDALQQGASGALIQALGITSSVTLHPTVQRFPLDEDCVFLLCSDGLSDRDRVEQYWEAEILPVLRQEVDLVMVRDRLIEIANTQNGHDNVTIALLLITVVPKDDPDAQKEVCIAPVVPLTEDLGDDSSDTSDAEDSSMETVIGRPRPAPSGVNKIWFLSLGIAVLLGILGALLYGLGVFNHDPQAQPVDNPTPTDSPSTPPIPITPL

>tr|A0A1J4URK8|OS=Candidatus Micrarchaeota archaeon CG1_02_55_22

MHGRQGTTTFNSNRFGLHSVIGGRENQEDAGLVAHAANNSNAVLAVFDGMGGHEGGEIASGAALDGLNAHAGALTNNAELDGLRRAIMSAHNAALRATVNISSERPPGTTVAAAAVSGNTAHIAHAGDSRAYLLRDGVLTQLTQDHRVQRILYNCIGSKAVVPRVDVSTHQLRTGDALLLVTDGVSGRLSDEELAQHIQRASSAQAAAESICNEIQSRQRSSGRKFDNATAAVYFHSPKPPSAAPVRESKRTLYRRTTLVERVLTGQKVVSVKPKHSR

>tr|A0A1J5DUE4|OS=Deltaproteobacteria bacterium CG2_30_63_29

MLPDQGLYVLADGMGGHVGGKMASTLAVTLVTEFICVHAKKPGFEFPFKTDENLTYEANILSNGIKYANERIFIESCKTRDLEGMGTTITAILVAKDNLVVAHVGDSRIYRLRNGELTQITEDHSLLNHLINIGELKKEDAKGHANKNVILRAVGLKDYVEVATQVVAKVPGDQYMMCSDGLSDLVDDFVIAEVMRTAPSLKDACDQLIRLALHAGGRDNVTVIIAEVEEYIDDRPDRAWTTGIFSAIPLQQQQQQQQKHSEAPSPPAPPAPKPLPPPPSRNAHGHPGPAANVRLHAVRVANGGRVSAPPPPGHMAGGFEKRPSSGSMPAIPQSQNVPRHDPSMVSGSIPMMPGGAYGQVVGPPGQLAQTRAPINRRSRTPTDMPVYQSPAPEAPAPREETTDIKAPPPGFFDEPPPPGNLVAPSRGTATRPPGRPVRPANVSSGQYAQPEPPEKATLQEVSKSLEEEEDRTSEHVTPYGSDSFEVDDDVTIIKPQVQAVGGEEEYKPTAMLPAFKLPAPAAEIEDDHKPTVQQMPAFVPLEHRPGPARVGENTIVERSNYRDEGSIQVDSSLFAEPESTIVVDDSLLTEVAEEDNGKRPPPPPPPPNKRWNK

>tr|A0A1J5FBQ9|OS=Candidatus Levybacteria bacterium CG2_30_37_29

MPDGSDYHELGPQKEAPMPIKMAAETIARDETKPNQDSYFGIPKRGIAGVFDGIGGNAGGAQASRIGMETVKQTLLFRNPTSIAEAKKNIAEILMHAHQNIRLNAQRPGFEGMGTTGTVAQIIDNKDRTYTAVIGNVGDSRAYVFRDGKLDCTTIDDSSFTQGKLHEEAKEIQSRLSNLLTRNSQEAAEYFSKRNIISAALGSEDGVVPKIYTITLRTGDRVILTTDGVHDNLTDREIESIMRFNAEPENAAKRLTGNAHTISQMGKDNYARSKRDDITAIVVKIGADSSRDEKTIPIGSTVNVMRSDGKIDSDWTVGIYNQNGSVTVFSPNGKRKTVSIASLNENNPPPKKLP

>tr|A0A1J5HK09|OS=Candidatus Roizmanbacteria bacterium CG2_30_33_16

MKLNYKDYPDAHNYGLPETPTVAQTKLSDLVNSNHPYFWFLKNFATGNNPFFNFFFGSTPLDSLQDFQAGRSLRLAHSEIAPTLELFYGTQGGQVEQKLNEDAICIYPIGSNRLLVAVYDGASSQRPITGLEPFGISGAFYVSHLAALGFPTTAEYAKLEELKELTAKEVMVTLNDWLRKELGKVEGVDYNDTLSIPGMAATIALIDYNNQSVSIAHVADTIAIATYDQGYNVLTDNRNEHYDQKTLNLVRKIAAEKEISIKEAAKDPRVKQQLAISFRQKINTPDGCGILNGMPELVSNSLIHTANIRITPELRKIYLTSDGLYIPWTGLLNAEPDTSIRQVLGVIDRGVINSPFISATEALTWDDLFQKIQRLKLQDDKAFISVSFPHNGVTKKNDESVMNQALIPNRDY

>tr|A0A1K1M263|OS=Prevotellaceae bacterium HUN156

MKILLYPPLSIHELGHRDNQEDSIAQWDNRLFALCDGMGGHEKGEVASQTVCQAIGEWYQNRAIHPLGKPQVEDALAYAYAQLDKKDDGALKKMGTTLTLLYIGIEGAVAAHMGDSRIYHIRPAEGLLYQSRDHSLAFDLYQAGEISYEEMLNYGQKNVITRAMTPGEDNRMRPDIIHITDIQSDDYFYMCSDGMLERMSNDELVALLSSDASDEEKRQQLISATANNQDNHSAWLIHIEDVVNEEGDEQLVNEEPNARCNAVNIIPQSVSEPDDDVVIVKEAPKKQSLFQRIKKLIPFTKNK

>tr|A0A1K1P157|OS=Ruminococcus flavefaciens

MDKILSFAQTTVGASHLSRDIVCQDSSLAADHEKYSFAAAADGHGSPCYLRTERGSKFAVECAAECVGEFLDGIENAAEVLADERQREELFNQLWRSIIARWHDKTEQDFRNEPFTEEEYSRIPEKFASYITRYESGEYIGAYGTTLLFAVVTEDYAFGAQIGDGKCVVIDGDSNVFAPIPEDPRCYENVTTSMCQDDAALSARFFYLPKGLMPAAVFLCTDGVENSYWNEEQLFSFFRGLALTIVENGMEDGVAQLADFLPAMTKKGSGDDVTCSGIINMPKLGACSDGIREDLEAAAAAISEETLPEDIEYSDEESQQVISDVVTEPAESEEAAEEAPEEQTEISE

>tr|A0A1L7CW67|OS=Corynebacterium sphenisci DSM 44792

MRRSRNHPRAGGPAPSSAERAPRFFDRLRLTHRRTIIIAAADLPAGPASPLPPARAADPVEAPAQPPMRAHAAPPAAPAPDPAQVPEAPEPWSPAAEPEPPAAGPRPRAVPDTTWLEPLAAGEPGAEILPVAVPTEFGSGRRVPDTLLDGWSTDSLTVRAASIRGRMHRYNGAPRQDAVALLAADDGATLYLAVADGVSAARFSHRGAEAAARYAVGWLAKCWTPEAEVDWSSLAKGAAWQVQSLAEPDKGAQPTDFATTLVCAAVRRRGDRLVGSVFSVGDSGSWVLSAGPLRLLEGGKAAVAEGEVATSAVRPLPYPPASLDVADFEVAADEVLLIGTDGIGDPIGGGGGPLTDLLRERLVGRLPTVLEFGHVTDFSKAGFDDDRTLVAVWPCGAKER

>tr|A0A1L7GEB2|OS=Streptomyces sp. TN58

MAAAAPTLVGDPADWATAPPQPRPERGPEQQPEPEHRPSPAGASYPGAGHENPYGSGEGSADGPETRHDRPGGDPAGGHPGAPGEAAPPWSTGEAAHEAPRENPYDSGYDGPSPSGAAPSGAAPAGGKTCVACRAGHVDTDGYCEHCGHAQPRERDHVEEELGSVAAVSDRGLRHHRNEDSFAVSATALPDGSAATVAIVCDGVSSASRPDDAAAAAAAAANEALLEALPRGAHPQEAMHEAILAAAAAVDALAPEVPGAQNAPACTLVGAVVSGGLLTIGWVGDSRAYWVPDDRAALPRRLTEDDSWAAQMVAAGLMGEAEAYADVRAHAITGWLGADAYELEPHTAIFKPDHPGVVVVCTDGLWNYAESAREMAQVLPADAAVRPLHSAQVLLGHALDGGGHDNITVAVVPFATQTGGGPGAAPEPGPAAAPEPEVEARPQV

>tr|A0A1M3EHX0|OS=Cellulomonas sp. 73-145

MAVEEFARLAGRPSVGTADVLACLDSAWARLREELTGDRQGGTTVAGVATVEDEGRPSWLVFNVGDSRVYRWADGVLHQVTADHSVVQELLVEGRVTPDEVARHPERNVLTRALGTGRRPDPDLWILPAGPSDRFVICTDGLTRDVPANRIAELLSQEPDARRAASALVAEALLNGGHDNVSVVVVDGGTRADGADGTQPAAAEPRDTLPRQRRDAPPHEGPTAAPATPPARSGRHS

>tr|A0A1M4E697|OS=Nonomuraea gerenzanensis

MNADAMALVRVGEWTVGVVCDGVSMSSRPERAAQVAADTGAATTVALLRAGALPETALTEGTIRAARAVTALAAPRMSPAPPGPPPDRRSPDQPALGPSDKPGENPPACTYVAGIASPDGVWTAWIGDSRAYWLPREGTPMTLTEDDTGTHDALSAWLGADAAGPDPHLRSYRPHTPGVLLLCTDGLWRHLPTPAALRTRTTRGTDHLHTARALVTHALTAGGEDNITALLLPAAPRSHPG

>tr|A0A1M4VQ25|OS=Seinonella peptonophila

MLNYRLEGLSIQNSNPQKPIEDAYVVTSVGKDQEVIAVLDGATGRYGLSGKIASETFQQELETMQEKDTLLEVLERANQKLASKAIAKFEELIAQKALPADFIEQYRSIQETDPQEIITKIDPAYSTSTAGVIIKIDRRKDEMELVQTGDCMAIAKMEDGQIQPLTKDHVAKSDLFSYHAQQIARDQLMKEQNIKNLSNLSTEEYQAFQSEVQKRMELTLKTGRRNANHTGSSPISGGAVLLSNEHDQGSSYAYPIFNGVPIDLTLHHQTIPLKNINELLLLSDGLVVPQPRIGADGWQLAGELVFNEEGKGLNSLLEIVQLIETIDPRMEYFKDRVKPDDDKTAIHLCFERSRSQHPLQTKLQQRAEADRPTLEVQLISDRDHADVTFSYQTR

>tr|A0A1M5M6J2|OS=Chryseobacterium oranimense

MEKAVIYREKEEFKDFHLVLKNASSNQFYEFSFELDDFPNIRIRNIANLHDTGLTFENNKISGTPYVNNVHDLDIEFYHIHDESSTEIKKIHLFINADPKDLWKNIPSDTDDIYHKPDENSYKGNFLDKKITVVSKRGRSHAHEGKFRDDDFAVKALPGGWFIVSVADGAGSAVLAREGSRLAVSSVNRFFDSEDILNNIERNIKNIYSFQSSKEMKSEAGEKVIRLLYEGVYHVYHVLDKTAAENSFSIKDFHTTLVFTLTKKFDFGYVILSFGVGDCPVSLINPDFSEVKLLNHMDVGEFGGGTRFVTMKEIFNDDNVASRFTITCVNDFSYLVLMTDGIYDPKFMTESRLEDIESWKTFFNDLGGNNDDRAKVDFINDTDIDEQLLLWTDFWSRGNHDDRTLAIIY

>tr|A0A1M5VPQ9|OS=Ferrimonas marina

MPIWPASCAAAQWRGDRQEQQDAVLHLPLAHHSCALLLADGLAGHPAGAIASGLAIRAAARTLEAHYRHPDALALALHSANRAIAWHIRDYRAHRGMGTTLLMVLLKGQEAQWLNVGDANLWHLHHGQFDNLVSEQPSESSNRQEAKAGSVTAALCGLPIARSCQGQRLLQQGDRLMLATDGVSALGLSTLRACARQPSCKSVVSAVITALSQQAPAGDNASLIVAAPRRGRLGSVRRKSPLALPWPVAAKENAG

>tr|A0A1M5W0Z2|OS=Streptomyces sp. 3214.6

MSQQGGRSTGHEDDWWGQLYDDFAEDTGPTPAADSLDDRFASASGAVRSAGTAAHSEGATAGTGTAAGPRAPASGADDETHLADHADDSDHAEHSDDSDDLEHVDGVDGADPSGPVGSVPAPRAERPGPRGRTDWWASQVGTPTGPPPSPEATVPPPRRGHMADSDAARYPDSGLADERDARDDAGRGTGRDVGRHAGRGVGWAADPNASSAAGQGADVGARGVEAAPGTRPPERYRAPWEPPSATPPGPANFLPGPLPPGFDERAPHGPASSKPDGPTPAETPSAPFAPPAPPSPPAPSTAPAAPTVPPPPPALPPIPAHAPPPPTAPTVPTAPSAPTVSASGTAAPLAPLPAHAPNSPASPASPAPSPAPEGAPDPQKDYVGSGPPTYDAEPTALPLADPDELDDLVADTVLDGARYGAWALRAVSVRGDSARYRGEPRRDSLLTARFGAGEHALILVAMATGARATPGAHRAAAEACRWIGRAVGRSHVRLAEDIRAARRGDLKSGLHRLTDRSLGRLRASAAEQGLAPEEYAATLRCLLLPADPDCRTRVFFGVGSGGLFRLRGGVWQDIEPQVGEVAGEPVVGFGSLPAETPEGDRLTMDLGITTPPNPYDPAPEPPRDPFRFRASVARPGDTLLMCTGGLAEPLRGEPELSAYLTGRWSGRTPPGLAAFLADTRVRVKGYSDDRTAAAVWEE

>tr|A0A1M6NUY8|OS=Nocardiopsis flavescens

MTMVRTCPGCADKVAAGDAFCERCGHSLTEEVRRDPARDTAGAGADPAAPAAPDLVPADGGPTAPQVSLYGADRYEGREEAAEDAVSGPTPSGSTAREEPHPQGVYRTVNPADVTVAGVIPSEATARSSGAAPSDPARRPGAPSGSTAPSGAPKGPARPPGAPSGSTAPSGAVPPSGAPVPGAPPAGAPRGPGRSLNDSLSVDDDWDAIDSLATQPVRRDPRPARPAPERVAPDWLPPAASGPVRPADPGLCVWCPGRVVDGYCEQCGFLQPTGRDHVEVRTDSVVGVSDRGLRHRRNEDAMAVRVVPDDAPHAAGVVCAVVCDGVSSSPRSDEASRVTAETGATVLAEQMRRGVDAREATGVAMTRAAEAVAAIADSPSSAPACTFVSAVVDPGAGTVTVGWVGDSRAYWLSGGPASSGSALLTRDDSWSEAMVQMGALSREEAMRSANAHALIAWMGADSGEIDAHISTVTPTGPGAVLLCSDGLWNYYPEARALTEAVPAAGTRPVEAARTYVRLALEGGGRDNITVVVIPVPAGGAARAEQG

>tr|A0A1M6QIA7|OS=Pseudonocardia thermophila

MTILDHPSRVTSGSATRRGVRAVAADAVAVQGRVAAVADGVGDTEQAALAARGAVDVAVHVAAAGRGPLAAVLAAAAAVRQSTEAGDAVLVVAAGRADGGWDVAWVGDAVALLHDGRVVRPVTHEHTVAAQLRAQGVRVAPQWDNVVTTSLRTVRPDEVGTASCPPGTLVLLTDGVHRTLEPGEIADAITGSPDPQRAAERLVEAALAAGAQDNVTAAVLAG

>tr|A0A1M7MNQ9|OS=Ruminococcus flavefaciens

MYYCCGVTDKGIMPHNEDALLIKDSVIDSGSSEQTVSGPFIAAVSDGVSGERSGELASKMCLELVRDMSYSGETKLDEELLGIHHKLAEYSRSDPEMHNMQATLCGIAVDESNNILTFNVGDSRLYRYRSGRIKQISRDQSLVQLLIDEGAITHEERKTHVHRNIIFPVLGNQKSEPQIDTVVLEDGMEYGDLLLLCTDGLSDYVSILDIEEIIELPKSLKSRLHLLVEKALENGSKDNISIIAVAYYEK

>tr|A0A1N6QW44|OS=Pseudacidovorax sp. RU35E

MGGRSGGRKASEQVIMTAQQLFARYSPGNDEPGRLLRQLVDEAHTVIRLTALSSEQEPHSTVAAFLLDPDGRGAWIHAGDSRIYLFRRGRMVRRTRDHSYVEVLVQRGELDEEEALRHPKANILVGCLGMQSTLPPMDLQPIDGVQPGDALMACSDGLWHYFTAEEFGAVIARYPARAAAEMLINEARRRSGGAGDNLSLILVKVDELPAKAPTPPATPLDAFLNSTPQR

>tr|A0A1N7D8R3|OS=Janthinobacterium sp. TND4EL3

MINIGPPGPQQTMHLIKDELDFGPWLDAAAGSSVGAGPMPRRENQDNFLLIDASGHAVCLSQQAPFHCQVPGWPAGHVRAAVLDGMGGHGHGREAAEAAVQGLLAIPACHDTASLSAQLDDLHARLQAAFTADRPARPPGTTLTLLEVPPGKAPLLYHVGDSRLYEISDGTATALTVDHVPATAYAMRGALDEAQWRARVHGEHHPQISQAFILGNAFGESLHLDKPLLALDADNLPPFLARLGDRRVLQLRSGAHYLLLSDGFWACEDPYAATARWPALCAGRSAAQTVAALLGDFLANPPKGLSSDNLTVLALRFLP

>tr|A0A1N7EGU8|OS=Williamsia sterculiae

MTVTGVGVVRDDTLSGADVVGDVHVSWAAVSDVGRSRETNEDAALAIPGLYVVADGMGGHDRGEVASEAALESLRAAERADIAATRLSIVDRLDAAQQDIAGIDSASGRSAGTTVTGIALVDDPTGPQWLVFNIGDSRTYRYAAGQLDQVTTDHSQVQELVAAGYLTVEQARVDPRRNVITRALGAGMPPDADFVYLPAVAGELILICSDGLPGELPDAEIEAILTDHPDVVDAADALVEAAVATGGRDNVTVVVVAVTDAPAAPDPAATETAATETTMDHPLVADADAADQVDGPDEGTGPRT

>tr|A0A1N7GE33|OS=Microbispora rosea

MEQVQEHEAPSMTETAHGFPQPLVIGREPRVISQPGPLPAVRRPDTEIDGAALPGLEVRAASIRGDAHRYYGTVRQDAMGLWHDGDRTLLACVADGLGSKEGSHVGAATACEVAQAYLSTLPIAPDHVTDARDFVDGIAGEIRNRADERGMPPEELSTTFLAARVQEMPEASVHRAVLVRVGDCMAWHLHRGVWTPCFGEDDADTGVSTSATHALPQDIEHAEVGYADLYPGDMLLLCTDGLAKPMRGPQVSSQLASWWSRPPSLPEFFWQMSFRAKTHDDDRTAVCIWRV

>tr|A0A1N7S7K1|OS=Paraburkholderia ribeironis

MHETSGIHGADAGADASADAGHDAGVQARERPRVTSTHARRWSVGQRSETGYVRSENQDRMSWIRTSAADVFVVSDGMGGHAGGATAATLTVQVLQDELQALDSLAQADQALVRALQTANAAVHARGQTPNPALAGMGATAVVLLAAGERIMLAHVGDSRAYRLDRHGVLHRLTKDHSVVQRMVDAGTLSSRDAEHHPDASLLERALGQAPHVTVDVSGWITVRRGELCMLCSDGLCGYVDDHAIASAMREGGAPQTIADRLVLLALHAGGEDNVTVQVLRCGEEHRANWRRWIGRGALAALCAAAAAMVWSGWLGFSAGRDDTAGGPVQAAGTAAARAGRFVTAPAASAAASRDASIAALERARQAIAERFLHEDALLMQKLDALLKERDAARGDAAAAAAASGALVPAKVASEAHEVSAAGARNAAVAANAVQGVNPTLGASASRASNDTSTTRAQSNTTRHPAAAIAHKPPAKRAQRPTPRAVAQEQPASAANASNEPQSADSDGQTVEIKQ

>tr|A0A1P8WCR9|OS=Fuerstiella marisgermanici

MIEKITMSTSLLTFAITDAGLARRRNEDCFLVASAESDATIVASRTAAATASIEGGDQQLLAVADGMGWHAAGNLASAIAINTLRASLNRLHVAASEKGRSARDRMLLDGLRQTVRQANRRIFHESSEHPELSGMGTTLTAALVHDRNVWIAHVGDSRCYVVRNSELIQLTTDHNLATLLKQIDPDSVDSGSGANVLWNCLGGTDIRDLEIEVKQCVLQPSDYLLLCTDGLVKHVDDEEISSVISSAATPEQACRRLVEMAKQRGGRDNITIVLGQHQDCGQLEPVGSASASDHTNTDLADTWIEHTIGPLGTA

>tr|A0A1Q2LHZ6|OS=Helicobacter bilis

MQEFISICYETQGRSHVGSKTPCQDRAYSLIDSDIGIITLCDGAGSARLSHYGAERTSRVVANLMRDNFGRYYTEAEPSVVAKEILESINNELIKESETRTNELKDGAYKEIQRLLEKGRNDLNSVCCNIDFNYKAELECKVDSLNKDIRELHNRKDKSKREHDSYRKDLQTDIEEYWQEYKERINNLRIFYIKILKKQHKKSLKKFFKKFLNFFSKEDELSGIKDIESLCKYMQDSISQENGSLKGLLSKIESSMNDIKADKQEKIANIIKDLEKIGQDILKTLKQVYKDINKHAKELQEKLKKQKDELEIQQGELHNHILKTKKEFKLIIKNFMQCYEAAAQREQDLLEKAIKDKESKKSKLQSHIKDHDDLKQKLSKAKNAFNSKKNEIKEQIEKIKKYSKERFYKESDIDSIKINELQNVLDSLENTRRTCLDFIANKINMQNRDMEKKIEINLESLLENIKKEITNSVCTPKDLASTLLFAAIKGEQCLIGHLGDGAIGGLYGNELKCISNPDNGEHANETYFVTTKHAERALKIIKGNIKEKDIHAFVLMSDGSTEGLYSKRENKFIESLQKHMLAIREGQDKVKKTTRYRKSYRKSKRAKKL

>tr|A0A1Q3L626|OS=Bacteroidales bacterium 45-6

MVITFEGKTDLGKIRTNNEDAFVAQYIWDRKHLLAVAIDGVGGYEGGEVAAAIAQKTVTEYLEKYSNGERLSLLKQAVTEANNKIAEQRIIQTRFASMSCVLTACLIELEKRRINMVHVGDSRLYEYRHGKLLKLSHDHSLVGYREEVGDLTEEQAMNHPQRNIIGRDVGSGFHEVDDNDFLEAKTFPLHPNSILLLCSDGLSDMLRSDQIALVLEKELPLAQKAGSLIDLANEKGGKDNITVVLVEYQDEESSEENSGNKTIDIPVHIDTAPPSEIKTGSDKKSTWKKAAKATLIPLALLLGGLGGWFGHNYLPSNVPATTPVQDSVKLKRHDTISVINKDSLQIRITGIGLKTDTLHQ

>tr|A0A1Q3M6F2|OS=Acinetobacter sp. 39-4

DAETELSLINDRFVVKPCSTTPLLLSTTANVDSSNTDHKQNNPSGKQNHEVKMVKKMPHFQIPNARVGQDYQARIQMQYPVKEDVLICSESIKIPEDLGIVFDDEAQQLQGIPLQAGEFKLAFQYKNSEAAQAWSSGEVTFIVTADPRSLWQVNEPDINAPYQKAHSDHQLIQTEYFNLAACSQRGRSHEHAGTFRDDDFLIARVAETDWSLLIVADGAGSASYSRQGSLLAVQSAARTLTDYLEQHHLHLDLLLQQWQVGSDDEMTKSVAQQIYKDFHDTFYKTAQVAIEAIEQEAELKQVAAKAYATTLLVAVVKQCEQKTFISTFWMGDGAIAVYAKEKVRLMGKPDGGEFAGQTRFLDRSFAQQFGSRVNIGYYDDCEAVILMTDGISDPRFETDAGLANHEKWQAFWQEIEPQLQQPHPDQALLAWSKFFSAGHHDDRTLAILWNKPVIADMEGLAHD

>tr|A0A1Q3RP94|OS=Clostridiales bacterium 38-18

MVGYQVAKATVKGDKPLTANKPNQDACDSLTVGEFKILALSDGHGSEKYTFSHLGANYAVTVALEVFREYLQHISKETTLDQIQFDIEERLSMSLQAKWINEILKDPNFQSIEQFGCTLLVAILSPEWIATLQIGDGKIAVVYESGDVYFPTARDDRFSFSETASMIQENAWIEMKVTVSELNEKVRLLGLSSDGVENAYPSGFYDDVSFYRELASSMALEETLNSLVQTASQYSHDDTTAIVCYNDELLKSPFAESIHTWFEQMPMECQPFLTSLIGGLNKRIEAAINLEHYFQKCCDLLPRFITLKRLFLDSEEAINTIGGGDRLPMSSERILQLIQQLVGISFMATSRKDIKERLIELQRSLRYDYKQCKYIFENGYDKVAPTVSFKGANGTYELFHNSYIYLHQILPMISSVDFPVGKVVQHPKNPRIWGIQNLSNISWETEREQIFSGKTLTIKPRTNFYAFGIPIQLEIKP

>tr|A0A1Q3T183|OS=Chloroflexi bacterium 54-19

MSFQYKEWKVLSKSVRGANHKLSSSPNQDYIDWRRYGQSLVLAVADGHGSSKCFRSDKGSEFAVKAFLTLFEELIANNSEETIQGLLKRDAKEHLSRKLVQGWKSLVDSHLENNPFAEDLENLLTREGYQTFMKVKLNPYLAYGSTILGVMVNPKFIFWFQLGDGDIVVITEKGGIVRPIGDNTQFLGNETASLSDKQAYNSVRVSFVSLENYPPQLIMLSTDGYSNSYPEDADFLKVAGDYLDLLKKWGDVKLEAALDTFLNTTSEKGSGDDITLGLIYRFSILPFTSQTEFNELPVVQTETSLTSVPSAKDNLSAYLPLDSGTPVIPEESQDFIRDISPVYSNPEESQPVTGANLRPFENENNYREGTASRLDPFYDNDLKDIAAASGINLSDLKPFNEVNPGHIKNEPEPFEK

>tr|A0A1Q5IYI8|OS=Streptomyces sp. CB02009

MTQQGDDNWWDKLYDESAPDTAPTASGDTLDDHFTTATRATTGPPGAPPGPAPDPWATAAVPVPPPKAAAPPSPPAAPPTPPAPPAPPVAASPPVAPPPPPPPGPAVPLVPGRAPWEARQEGPQTFPAPPPPPPPAPPVETPPVRAQTPGSPDDPEGTGTETGFRTGSDPESGSASGSGSAGEAPTDGPDSRSDSPSDSGSGLTVEVSVPRPRTGHVGSRPPTYEPEPTALPVARPGELGELVSDTVLDGARYGTCTLRAASVRGDSARYRGEPRRDALLTARFGHDETALVLVAVAAGSRAAEDAHLAAADACRWIAEAVCRSHARLSEDIRSGRRGDLKSGLHRLTDRTYGKLRTRAAERGLAPDEYTATLRCLLVPADPDCRTRVFFGIGGGGLFRLRDGAWQDIEPLLPEPAAVTGAPVVGFGSPPPQGVAAETEEGDRLTMDLGITTAPGPLVEEPVPPPAEPFRFRASVARPGDTLLLASPGLAEPMRGEPALARELAARWADAEAPGLAAFLADTQLRVTGYADDRTGVGVWEA

>tr|A0A1Q5PPJ3|OS=Boudabousia liubingyangii

MPVEIRYAARSDVGLMRKNNQDSGYAGQHLLVLADGMGGPAGGDIASSIAVAHLAPLDSDAVPGDQLLPVLTEALESAHAELISRSEADPDLAGLGTTCIAMLRSGNKIAMLHIGDSRAYLLRENKLIQVTKDHSFVQYLVDTGQLDPEAAASHPQKNVVLRVLGDQDEVLYPDESLREAVPGDRWLLCSDGLSGVVSEDTIAQVLMEEKDLNECADLLIQLALKGGGPDNVTVVLADVQESSSEDQQTTPQVVGAAATDRLAQSRAASSSAAKAAALGGKGLKDRVDPAADEEELSEARSERRSAIRKRLAWTLGSLLFVLAIAGGLFAGYRWTQTQYYAIAEGNQIVVYQGIPQKLGFIELSRPLEVKPYKLSDLSPAVRNRLEEPVTRGSRQELDHYLDSLVNEDGWRNPSQGSFDTKVILPGQKTPSPTPAPTPSKQPVPKPTPKPAPSPAPNMESAPTPSAAPKYLPAPELSEVANQAFIAKQMNASTQLLSTWSAPIKSSGGDQLWQQ

>tr|A0A1Q6RDU9|OS=Firmicutes bacterium CAG:110_56_8

MAVALILASIGTGYLGFVYDPGEDVPGGSLLPTGVNPVSATATGATEETTVGTETTAPAAVETTEATSVPMEADGAEAAPAKKDGSDKATAPTDEAPATTEAPPATTENIPAATEVPPATTEKPAANIEEPPATAEAPINPASENFDPGDAFPPSSTATEETPHSGIYINPHTFTQPASEETAPTNSEDTTPTVTSEPVALPRKSTPGFRLVMQIACFVFAAGACVDILLLAMLRKNVRDHERSGEQPTEPPTPAEVHPVPITKETVEETVCPIPGISLGKIHDIGRRDYQQDSFGQTAVLRNTGILAVLADGMGGLSGGERVSQKIVMEALTFGSTLQANQVPTALPGMVAGINRAVNQMLGPKGLSTSGSTVVSALITGNALRWISVGDSRVYLYRDGQLSQLSRDHDLLQDWMPDILGGKRSMAEALRDPNGRKLTSFIGMGELRHVDYNRTPIPLLPGDRVLLMSDGVYGTVSDAEMAAILRDCGSVQLAASHIGQRIMGAALPYQDNYTLIVLGYDPPDQPRNNR

>tr|A0A1Q7AV44|OS=Ktedonobacter sp. 13_2_20CM_2_54_8

MTQTPSDELDEAQSSQDAQATASADFPVLAVGTTVHERYEITQVISESTDQHVYSVTDHQGYQHCWNCGSEQNSEGDEFCIDCGAELQNAVYTMHEYPAAKSSDSDADTFHGTIVNTFVEQGRTYAVEQAHAAVNAFPNGVHLLAASDSDAGDVRRSEPNEDSTLALVLQRVHESQAFPVGVFIVADGLGGHDNGQEASRMAINIIAERMVRDLLAAPLASEKAGETVEEASEDSLATLLQSTIEDANSAICQVNQRDKTDMGSTITGFMVVGDFAYILNVGDSRTYMVRAGQIYQLTTDHSLVGQLVAGGLIQPDDVYTHPQRSQIFRSLGDKPNVQVDIFKQQLHPGDILLSCSDGLWEMVRNPQIESILNNAPDPQTACTQLLETANTNGGEDNVSAVVVFVH

>tr|A0A1Q7N859|OS=Acidobacteria bacterium 13_1_40CM_3_65_5

LFLVADGMGGARAGEQASALAVVAIEQFTLNTFKWFFHSGGAEAQRVLAQFQTALREADARILEESTEHPELRGMGTTVTMAYHLDAQLCIVHVGDSRAYMYEEGELHQITQDHTVTADMVRRGDLQPEEVARHMLRHLITNVVGGNEALRFVEAHALQLQAGDRLLLCSDGLTEMVTNEAIAATLRDEPDPETACKKLVAQANDAGGRDNITVLIVRFDAAPPSV

>tr|A0A1Q7PMQ7|OS=Nitrospirae bacterium 13_1_40CM_2_62_10

MSVNEQDWLVTKAAVRGKSHMESGSPCQDACEVATSTDGSWLVAVVSDGAGTASRSEIGARLAVKHVASALLTQTQRLEAEGPGIWVKDRLHSALLEVREQLREAGGSLADFKCTLVGTLIGGTGGFLFHVGDGLGLASRVVLTTNGPNGSGIELWHDLVLSEPQNGEYINETFFITDDDWHRHLHSVVLPADTDIVALMSDGTMPLVLGRRGPNSPFIDPLVSLVLDASGQRDRDAIVERTLASPETYPVTGDDKTLVLALRRRLLTLRHYSTIPYNLLPLPAPRKDSADSDRAPHDEEKASSNGLGSPRLPQEVERAKSGASAQRVNRLALGLAAAALPLAILSLLFSLQLIRIHPTRGTVYPRQPDGSAPANVSGTSSGPNASCQRDSAGRADSSRVRGKGGACGGAAGTAGKPLGRGKSVGHADSLRERQSP

>tr|A0A1Q7V1H7|OS=Cyanobacteria bacterium 13_1_40CM_2_61_4

MASTAAHSPFAGDAFVEVCSLAELAGMPSSYRLLTSAREYAVFQNSEQLAVTLAGRVGNPRVSPVPPLWPLLALLLLGWRHTLSAEQVIERAVALGLENEVQRGLAIVAYLFPELQAWMADIPLRIPLWERTLALPLAARKLALFGKMESPGDGSLAGGSSLYEEAVRPMQWRVTGASRRGRSHVRSNAPNQDAIEYWVSAGGETAVMAVADGHGSALCFRSEVGSRFAVATAVQVIRMFANTIRAHDTASAIADRARVLLAEELTQTWRIAVQHDVEAQPFTQAERAGLAALEGWTGQQVVNRHPELAYGSTILAVLATKTYVLCMQLGDGDILFVDSQGQTRRAVPKDGQVAPKQTASLWRQNAAAEVRVHVLEGLGDLPALILVATDGCAESCKSDDGFLDIGRKYLSIVRNEGFDSVEHRLKTFLEEASWGGNGDDTTIGLISRLEREPMPDLDSLESETDLSGSEKLNAAR

>tr|A0A1Q7VQF3|OS=Catenulispora sp. 13_1_20CM_3_70_7

MELVPETDANPGAAEASGTKVDVRREPFPEEPGDLRFVGPKPPSYPVQPTSAPDGAGIISGAVDTALDGFTAGAMTVRAVSVKGDRHRHYGEPRQDSFVIARAAGIGTTPHDGIVVAVVADGVGSATLSHVGAAIACERVAASVLREHSEFAKVLVAGDTDDAKQHLTRAIDDVCAALNAAAAEAGLAATEVSTTLRGVVASTDPDLLGRLSFQIGDGGTYRLSGTEWKAVDAEPIDGPIHSTATHALPDHPEQSRRRTR

>tr|A0A1Q8HSV9|OS=Actinomyces oris

MSWTTFCTAIKGRNKTQCQDATYAAKTGRHIILAVADGVGSHRHSALGSHRAIQLVRSLSPRLLESSLPPQDICRRIFGQVVHQWSHWAHSLPASDAHAPQFNNVQTTFAIAVIENDNVSVLSIGDSLLFVQGHSGPFLNLLSVQRSDSGSVNTVGDIDTGARPRELEINDPTISKVVLTTDGVERLLMRRPTSDTDEKPFHYVHGSLLATFDGTDFRDGESKQKLIDSISQGLIHRHGAKGDDVGIAAAFQDGDLPVRP

>tr|A0A1Q9D3E8|OS=Symbiodinium microadriaticum

MDLHFNFCLSDPQHPILNETSDRQAMEAAGKKTMDGKDGKEDAALVRAALSPTASGADTSATSATSATSATTATSYPRMTAPPQILGQATEHLMKKQPHTTTAAFGDAPRRSTSLSLLGVDRPLLAEQWARRKDSGPPPKEQCLNFKMKNLAENDAKKMSVEVVFQEKSQRPAMALELASYDKEEQQAAWSLAVPSADLTPGIHFFHFRVNGVFVVSCEHMRLGRWNAMHVSDPIRRYLLARDSKGQLSEDAPIVEKTRSKKIGDMAMDSCVGSGSTADVVGLQELGGQNNIARPYSVCGNLVGNADSDDEAAGQDSGNSFSPFAKEVYEGLFDRELMLRLDGVVLPEAPSPPAGKEPLDEGSELRLWAGAHLIKKAHGACEDAYFVDPHGMGVADGVGCMVQFASYGINAAQYAAELMEFSSAALKPEGLASESKIGDDVAERAATALAHGESHAAAYGASTVAVLCQQGNQIGVANLGDSGFMLLRKGPHGMNIIMKSEEQQHSWNCPYQLTRLPKALLNRFPKLQLDKASDCERYTVEIKEGDLVLMFSDGLRDNLHDREVLSIVDRALPPAQADMLGLLDRCTPPETIAKALALAAQERSMDPTAKVPFVEYSKRHGFECLGGKQDDITVVASWVVADESTLSPDTADVEAIVQDMHTAAAAAAAAPQKKMVGFRDQDFGILGQLGEDHKVATLQRSWKAEGAKKGGFEARTRAGDSVLVAALALFPVTVVCGLNIEAGGNSKKELATAPWAQEELLKSRVKELGGYGSLQRYPDGKAMEVTGAGAAMRTVFQYHDGPHAPEEADYSGSLDEARNKCVEDYRCEVVCYHQKTGKTQLYQNFNLALKGLGGSQKAWKDVLQKAPAWYDKDWTCLQASPSCRRKAAKTARKKEEAGLKDTLEGLGELRLLQQSLHQPSPPAFQTQGALSHDAANFLGLLKFPMGKQAQELREKCTDIASEVARAREVPQLEPFCQRKVWVHRPIKDSMGHWQECCQDHEGFKCKDRFVIPLDHCSSCQGVCLEKGQEPGPVQPAGDEGECLELGAAGSWSSRSGEVVELMSARAPTPTAQLLLECLGRPAIRPSRRRRRAFL

>tr|A0A1Q9JN35|OS=Roseburia sp. 499

MKRYVFGKSVKGATHEKNGLPLQDNCKIEEISDKITIIAVADGHGSSKCPRSDRGSLIAVNTFCTVMKNYLLNYGKEKDGLTNLVTFLNREGDMRFAQDICEEWQARVKQSFYKNKDDSVLDKEGNTDWKKVYSLYGTTLLGMLITDTFVFSFQIGDGDINYITGDEISPLVEPEKFLGTETHSLSKLDAWRKAVAAVRRKNAADDVPYLYMLSTDGFSNSFVNEEEFHKTCREYYKMIGEHGYEAVRDNLEKWLKETSELGCGDDVTVVMAYVDK

>tr|A0A1Q9LHF3|OS=Actinokineospora bangkokensis

MMGGTGTESAAQEDEPQAHRQSSFSPEVTWPSGKGGGIPQEGLPGLEFTPPQGTHVPGRTASTAWEPPTDNGHLWGDLGIQEQVRQPHQPPDGGSESPDRTAAFGLPAAGQDFGPAGGDEHAPRAGAKQQETPDEQQQAVEESPRGRQAHAPHAMELAIVGGFEPYSVGDPGRAASMVVPLPDTENWHRRDSVFDGFTLHLPQERPAAVVRAASIRGLAHRASGKPRQDEYAYQLSQDGRFLVLCVADGISSGARSHQAAEVAARTGVALVAKELTSTPPDSLDWDHLVREVAGHIVKFARTRLPGGEELTPEQVVSVMGTTATYAVLDLLSLEVDAVMVGDTSIWVLTRDQWVPLTAVKNADSDIATSAVEALPVVTARGRTPLRSRLRPGEALVLMSDGVGDPLGRGTGAVGRFLAGAWQNPPHEIDFAAQVAFSKRSFDDDRTVVAVWPVAPS

>tr|A0A1Q9VBK9|OS=Gordonia sp. CNJ-863

MDAATIVRDASVSGTHIVGELRLEWSAACNVGRVRETNEDAALALPGMYLIADGMGGHDSGELASEAALLTLSEATSAGELNATKVQLDDLLVAAQRRIGEIDTETDRRAGTTATGAVLVTHEQSPHWLVLNIGDSRTYRYQNGSLQQLTTDHSQVQEFIDAGFLTPEQARTDPRRNVITRALGAGMVDPVPDFFNTPAFPGDVLLLCTDGLTGELPDEEIGDILANASTSDEAAERLVDAALALGAHDNVTVIVVTVHESVPTLTMPALDGAAEGKSTDEATVSDPN

>tr|A0A1Q9YMJ1|OS=Faecalibaculum rodentium

MMEIMQVSLQGTSHLMFQVPNQDAMAVYSDSSTAVSAVCDGVSLNSVGTWSCSEIAAGYCAESFVRCTAGQSPCTDVVVRGFQRTATGLLQELKDRHIPWMDCQCTMLGVLVTPDMLYAGMAGDGGIICEDANGELQVLVTRHKTDSMVDPVILASAWRFARVPEPRKVLVMSDGLFDDLVSLEEGELRADLDQVRLWLGADQEQLEKLAASAPGHDDKTAVLIRIVDKATELPDSFEPSAD

>tr|A0A1R0LUN4|OS=Streptomyces sp. IMTB 2501

MSRDNSRRGGLMDGRGRAMLLVAVVIGAFIEVSTPHRQYGRLVDVFALAAVGVAGTAAALGSEAAATLARQRDRLEQVPKPATAVPICRYVTGPAAPQAQPARPQPFLRERPTDLSVPRFGDGARAQGYPWLLPERTVQNGIAADEATVGAFTIRAASVIGPGHRCGRPAEPRQDSYRIGRSRDSRYAIVAVADGLSSAAWSDAGATTAASQAVTLLREQIEAVGFDRLDVKELYARIAESVAAHAAGRGVGTSHVATVLITAVLAEPDANGVAQAWVAWLGDSSAWTLDPRGPLWLFSCGDAKDRTAAVVSNEVAGRLPDTPQLARGHYVSLAPGAALALVTDGIGDAWADRAGNVNEYFANAWRSPVPATRFIADVGFDAPQCLDDRTAVVAWNGGRA

>tr|A0A1R0LW44|OS=Chromobacterium violaceum

MKLSIFQESRIGGRSYNQDRLAIAHSREAVMLVMADGMGGHMRGEVAAQITVDLLSELFYQQATPTISHPNRFLVNAISSVHQVILEYAADHRLPDVPSTTVVVAILQHGQLYWCHVGDSRLYLLDGGGLRLRSRDHSQVQRLIDQGLLTEEGAKTHPERNKIYNCLGAAGEPDIDIGERQVIGPGCSILLCTDGLWSQVQDAELEKVFAGRSVNQVMPALINVAERRAGNGGDNLSAVAVTLLEDSLELGERDDVLDTEKTPPQKAGRLILESNLATMHQEILASQVDKDS

>tr|A0A1R0VEA6|OS=Mycobacterium sp. IS-2888

MTLVLRYAARSDRGLVRANNEDSVYAGARLLALADGMGGHAAGEVASQLVIAALAHLDDDEPGGDLLAKLDAAVRAGNSAIAAQVEMEPELEGMGTTLTAILFAGNRIGLVHIGDSRGYLLRDGELTQITKDDTFVQTLVDEGRITREEAHSHPQRSLIMRALTGHEVEPTLTMREARAGDRYLLCSDGLSDPVSDETILEALQIPDVAESAYRLIELALRGGGPDNVTVVVADVVDYDYGQTQPILAGAVSGEEDQLTTLPNTSAGRASAISPRKEAAKRVAPQEETPSRPRWPRRRMFIFVTLIVLLVLAGLAIVRTVIRSNYYVAEYNGIVSIVRGIQGSMLGVSLHDPYLVGCLNARNELSLISYSQSGGHLDCRVMRLQDLRPAERAQVQAG

>tr|A0A1R1L8Q7|OS=Tersicoccus phoenicis

MTETTTAEGADDVVRIVSGFTTDRGLRRELNEDSLIVTDTLFAVADGMGGHEAGEVASGVCVRTLGESELLARTDATAAQVQELLEAADARIREETGSRAGTTLTGVVLTREAGRPYWLFFNVGDSRSYRLYQHRLRQISVDHSEVQELVDLGHITPEEALVHPRRHVVTRALGTGVDIAADFWLLPLEPGERILLCSDGLTVEVPDEAIGRVLDAVADPQAAADTLVQAALANGGRDNITVVVIDVVGGSGAADDAGDAEGRGDATGSATAG

>tr|A0A1R3UQR5|OS=Nocardiopsis sp. JB363

MTMVRTCPACADTVSMSDAFCESCGRNLPVEAAAPDVVPTAPQESMAGIALDDADAQVTQPIRRDAVIPEAGPISVSPNVAPEWPPPATGSNPVAVANPGLCVWCPGRVTDGYCEKCGFLQPTGRDHVEVRAGKAFGVSDRGLRHKRNEDAMAIRVIEEHSPHAAGAVVAVVCDGVSSSPRSDEASRVTAETGATVLAERLRQGVDPREATGVAMTQAARAVAAIAESPDSAPACTFVSAVTCPRTGAVTVGWVGDSRAYWLSGGPTSSTSTLLTRDDSWSEAMVQMGALSREEAMRSANAHALIAWMGADSGEIDAHISTVTPIGPGAVLLCSDGLWNYYSEAEALTDAVPDLGTAPLEAARTYVGLALRAGGRDNITVVVIPVPTGGHRAEHV

>tr|A0A1R3VGQ0|OS=Mesorhizobium prunaredense

MNNVALPFESFGVSHRGCVRELNEDSYLVEPETGLWVVADGMGGHDAGEVASASIVDHLATIGIASSAPDLRARFEDRLSRANAEIRRISQSRGVTIGSTVAALLAMDGRFACLWAGDSRVYLIRNGSISQISKDHTEVQELLDKGMISAAEALAWPRRNVITHAVGVSDEIVIDFQQGEIMPGDIFVLSTDGLTAHVTDAEIEAAAVSATPQAACENLLQMVLARGGTDNVTIVLVKIGDGRNGAYPGPSRAEG

>tr|A0A1R3WZD4|OS=Yoonia rosea

MVNVVDEAIRFDVATAATKGERDYQEDSLIASFPQGQETGFAVLADGMGGHMCGDVASALVMSEVFSQIKMNEMLLSKRITDISSILHMTATAANDRISQYVDERPDSYGMGATLLATVIRGNELYWVSVGDSPLYLFRDGVLTQLNQDHSMAPQIDMMVKVGAMSEEMGRDHPDRNTLTSAIAGHAIAKIDCPDAPKTVRTGDIIIAATDGLQFLSNDAIAAILQETHTKPSMNISHALMAALDDLADPDQDNTAFTVIKLGASQQVDQSETDDLESAAFYRRPDLAMTKTPILETVATNLAPRTHAEIDPIDIPQKRVIRLVTSQTLDFKQTAASSETGAAVAPSKSHGNTSWYRRRTSND

>tr|A0A1R4KPS9|OS=Microbacterium esteraromaticum

MTAPIMTRSASATDVGARRRLNEDAFLAAAPLFIVADGMGGHDAGEVASAKVVEHFSTLSGQESLSIGQVRDVLASARTAVGALGQQGTAGAGTTLSGVVIASVDGLGYWLVLNIGDSRTYQFSNGMLEQITVDHSYVQELIDEGEITPEQAKSDRRRNIITRAIGAGSVGDADYWLFPAESGDRMLVCSDGLTTELPDERIAEVLATEPDPQRAADMLTAEAVRAGGRDNVTVVIVDALTVASARGVHVHADEADELDDTRPRAAVDGGMR

>tr|A0A1S1QPQ4|OS=Pseudofrankia sp. EUN1h

MSSHPAYGGHDDGEYRSAAVHVPGGKGAGGPGRHSDARRTERSEPSRPGGDGFRIADVPPWLLVGWIVAGLSLAAGAAFTAIGFLSLQVGTMRTGILTVLGSVVMSLVLGFFQRKHNRALPAGTPGLEGGRKAREKKGAMKDPPPDPAIGTPAEQPARAPDPEDQGLEEHHGFEEHGHGQWFAADPPPIDRGGSPDHVGAASQVLAPAPADFRVLGARSDLAREPWRLPVLPTQPAVAADQARIGTLEIRAASIIGPGHRTQDPATPRQDAYRLGRDTTGRHLIVAVADGMSDSPRSDHGATVAVSTAVAKLRSDLDSGATVQHLSADRLFTELARAITGSAQQRQIDPRDVRTGLIVGVVEVELNRQGRRSAWFGHLADVSAWRQAYPAGHGWEQVAGDHKGAGTDANTLRAFLPFHPNEAQDTWLTLPPGAVLAFLTDGVSDPMTQIPGASASFADWWATPPTLGAFVLDVGFEARTHQDDRTAVVVWCDTPPPGQPDPASPAVGARGRGQPR

>tr|A0A8J7DMH2|OS=Romeria aff. gracilis LEGE 07310

MPVCEHCQFENPSGNKFCQRCGEPLQQWQAWLIPHAADAKLPDLCPGDCLDSVERYQLATPLVFHPTAASAIAIDSQPEQPTLSIAPDSRSDAAAFAAAAPAEIPAAAHPYLFLADRPAVPTLHDAWQQADYTVLLVEDRSHLPRLEAAWPAAPTALKIHWCYTMVELWPALAEWNAQASLTALDNLRVDEDQLCCLQQLMVQAPDGVLPLSALGQLWRSLLNGEGDQPAALALLADKIGAGQLASPEPIRQELVSLAEQLESSTELTETLLTTPMLMALETEDDPFELGEFSEFVREDDGNRAEPDLSTMVLPMKLTHLEDTGQTHVGRQREHNEDTFFIQTEVKKQSNLQGQRLQARCLYVLCDGMGGHAGGEVASQLAVDTLRDYFTAYWQTERGQAGLPDQDCLCEAISQANQAIFDINQAEERTGSDRMGTTLVLLLLQGSQAVVAHVGDSRLYSYSRRLGLQQLTVDHEVGQREIEKGVEPEIAYGRPDAYQLTQALGPRDQKRIIPTVKFLSFTEDMLLLLCSDGLSDHELLENHTRSHLDPLLRSPKDLEAGISELIALANESSGHDNITAIGIRLKLKPDLDQLPPLQ

>tr|A0A8J7Q0L3|OS=Acanthopleuribacter pedis

MFPDKASCGSAVMAVADGMGGHEGGEVAAKQCIKIFGETLKDGYDGAWRWPDAWGKPPKTGERDLESFILEHALMTAHLAISKLAREDSTLHDMGTTFTGALIEGETLYSIHAGDSRLFLYRNGFLKQLTRDHRFDPAVSDTLMIPAELAERYTLPNVLTQSVGNGPLKPDLDSHKLQWGDVLLFCSDGLTDMISDEHIRRILGAEPSAQQRAQALIDAALLQGGRDNVTAVVATCQ

>tr|A0A8J7QFI7|OS=Acanthopleuribacter pedis

MFQLLLPKRKNSRLTKLTIETASSLCTAEKKTKSSDVPFLSQVPPFFMLADGEGQEDHGELAAALATRYMQKCLEDAWSDDYQWRWPLNWGTPPENVNADAPEILLNHARNLTHEYLLQFKSKHKKWSDISAALTTAFIVGNTLYASHTGSNRIYLSRKKDFQCISTEASGAMLGDAQSHIDSLENADKRKPPFLLKKRLVHGDRILLCTKGISALTNQEIHAVIAEKGLSADETADSLIKAVRAKKPPEDTAVVVVLVARGGGR

>tr|A0A8J7QLB2|OS=Acanthopleuribacter pedis

MRTLILDVGGYATQGGDGQEHAGNFCCSGAPPFFVVAAGRGEKAAQKLAGYLAVQRVAVLLQLARDRGHWFWHSSWGSPPVKQYANPLEAILQHAVSRAHDQLRRMSGQTRGISAMHTALTAGMIQGRELVFIHLGDTRLYRLRGGRLRCVTKDHTAAMKLLREGSISVDAYHAHPGRHVLTKYLGGASPHDAMFGAVDLAPGDAFLFCSPGVYNALSEHTMQNVLAGKPQDATRKAKALVLPAKNGSCSGSAAAVVVGVT

>tr|A0A8J7TL44|OS=Candidatus Obscuribacter phosphatis

MLKWRAAARTDAGCQRQRNEDNYYVSPDSRVFAVADGMGGAVGGARASKLAMEAVEKQWQECPPPGTDKESIKTWLTKAVNEANAAVWNSAEEDQSVRGMGTTIVVAVQSEDNYIQIAHVGDSRAYLYRDGKIKLLTNDHSVVQEMVRAGRLTEEQARINPYKNLITRCLGHEERVEIDHTPVEMQPLDWICLCSDGLPTVLRDEQIGDVLGAGVEPEAVCEELVKQTIDGNAPDNVTVVVVKYSEDNG

>tr|A0A8J7W9T7|OS=Thetidibacter halocola

MQLNDCFTFETGQATDVGCRREINEDSFLSRPDWGLWAVADGMGGHAAGDFASQTIVEELHSIGMSGSPDDQLARFHHRLARANQLILDHAATLGRGTIGATLVALLIHGDEYACLWSGDSRIYLLRDGRLRQVTRDHTELRALLDAGTITEQEARNWPRRNVITRAIGVSPHPHCDTVTGTLELDDRFVLCSDGLTEHLEDHEIAEFVGSLAPRAACHAMIDETLRRGARDNVTVVVVRMTPPPPPEDEDFPPEDTLTIEATLPDMTVESAQADPAGTDGAGDDGVADGPAEDEKA

>tr|A0A8J7WC35|OS=Thetidibacter halocola

MAELIPEATALADVGRRDRQEDSVIADFSQGARLGLAVLSDGMGGHEDGDLASRVIAAEMFSELFFSGAQPDALIREAPQLFPSALEIANRRLRTHAREGVVSDGSGGTVIAVAVVNGALHWISVGDSPLYLWRDGLLLRLNEAHSMAAQIDLMVRAGALDPEEGRTHPQRHWLTSAVTGAAIQAVDCPKSALALQPGDVVILASDGLNTLPDERIGEVIRRNRDVGSQRTAQALIEAVREASAPDQDNVSVVAITLRAAEDPQPRPAPAAARPCRQGFGAVLGRLVDALRAPARSGARQARP

>tr|A0A8J8BEC0|OS=Actinocrinis puniceicyclus

MREPERDPRRQGQPARQQPGRRHARDPQSEIGRPGYGALDARYDAPWYDDAPRNDPRHDVYGPVSDDPYAAPDTYTPGHTYYPDRGRPDQDRSGHDRPGQVHPGQPSAGPGHARPQRAAQQSTDQADGQAQGGQAQGGQQGPEHEHAAQVWPPGWAPIVVDRPTTEFEPKPPGARHLPDTEFDGWSTAHATVRLASVRGYSHRYYGKPRQDHVEACVHAATGTVLFAVADGVSSAEEAEVGARLACGTALAVMTEHLDAGHAPQWREVLRTAADQLLMAAARRYGRAIDPAEAEKLFATTLVAGFATPAPEGLTASMARIGDSGAWLLAEGQYRPALGAKDDPAAAVVSSAVHPLPRVPDPPDQTQLTLVPGQVLLVGTDGFGDALGDGRGQVGRLFAQWLAQPPPARGLAHLLDFSRETFDDDRTLLALWPRTVQGSGAGSGPR

>tr|A0A8J8BEV6|OS=Actinocrinis puniceicyclus

MTKPHQGSQRTDVMSYGPPHERAASHAHDERQPPLRYWSNGRRLIAAALCVAAAALLSASYYQPHLRPASVIVVILTGGILFRLWPRGKAFPPDSKSRRHPGGFPAMAQPPHDNGQSTGMRSAAGTQIRLNQDAPTPPQSPVGARPPGPRQDVYRNPQQGPPRQSPPNPNQALHPNQALHPGQAPNPNQPLYPPRGPNPLRPLVFVRPSAYSTAQWRLPQLLAQPGVTADQACVGDLDVRAASVVGRGHRHAAGRPLPRQDAFQLGTARCGGRGYLLVAVADGTSSAKYADLGASIAAVGAVGFLQNRLQADAAPDEKSCAEMFQWLADTIASVAQQEGHRPGDYTTTLLVAVVPDAPTDLGAGREVLLCWIGDSPAWQFNGDGWHQVAGHAKQGYDQNTLDASLPSGPRLFQHTTIRLAPATALALMTDGLGDVLTNVSSSQAELARRWAAPPPLPEFLRDLDFDAPGQDDDRTAVLVWCPAGRQAAP

>tr|A0A8J8D9L9|OS=Thermococcus sp. M39

MKNAVFLFIFLFLTGLVAGGVVPINGTLVITSEPVNAPIYINGTLKGRTSLNITLNPGVYIINVTNGKAWNATTVTLTPGEKKEVVLSLPKSEESEMSLLPILGLFLLAGVGAGVYIYRSRQSKESSTEVRKTDSFIEVKAERAFGISHIGNRENNEDNLLILKLPDAYLLAVADGLGGHNAGEVASQISVDTLKEIFEREDTKEMSEEEVKALLGKAHELAHKKIKKNAIGEREGMGTTLVTAFIRNGKAIIANTGDSRAYLIRGGKIVSRTKDHSFVQQLLDKGEITEEEAKRHPMRNIITKALGIDFGVDFYEWELERGDVLLLSTDGLHDYVDDSRIAEIVSQGKSAEEIVRKLIEDALPVTKDNVTVVVWKG

>tr|A0A8S0TMY0|OS=Olea europaea subsp. europaea

MADFFCSLLDSRSLITNFQIYFPPKTFVPTIPHFLNPRTTRRKRKPQRLTLLSESKSPTSSSSPDYDIISIHEHSDGSLLFRFGDPSEIAENVTPAEPRIFKEIEEKSQEGFSMVKVSDGDIERKVIIKKLDKEVRSAGLTEVVNKGKESTSVVETESKSSEKLSEDPKKVELETDIANSIAISNESITVVERENKFSEKLSEESKQVELAMSEANPIVISNESINVVKREDESSGMFSEDSKKVELAMNESNPISIGNESVSVVERENESREKLSKDSNDVELTVQISSIDTEDSSGRSADLESVGYVEECSSEKHKDEISPLVSVTVLDESSTVDNGSSIMGSPKKKIGGDMVLQSIHLKPDPVNDLPDEIVSGESAGETIAATNISANNRSVQAGTRIETVEDGKGNYRSSVTPVSPQLELSTSLSRDTENQNLEIGMPAEINKDGNTSDTNAVAMFPFTEAAVDFSSDSKDQNSEATVDSSEDSKDKKEGTENGMPSNAVEVGSQSDMIEVMPISQLESEPILDEEADNDSMEEAAVAEINESVTMLNKSQKSLLETRTDDDDSVRNNDSIEAGCEVTELKSIESVASRIKADFVLSSGAALLPHPSKVLTGGEDAYFISGQTWLGVADGVSQWSLEGVNPGVYAQELIKNCENIVSDCTGDSGNFPVKVLKHSVDRTQSPGSTTVLIAHFDGQALHVANIGDSGFIILRHGTVYKKSSPMFHEFHFPVQIARGDDPSCLTEEYKVELEEGDVIIAATDGLFDNLYDLEISSMVLASLDNDQPLEEIAELLAIQAQEVGRSESVRSPFVDAARAAGYIGYTGGKLDDVAVIVSVVRRQ

>tr|A0A8S1DMI3|OS=Cloeon dipterum

MPSIRKRVVSFIRQLSHPHSSTPKPLPPPPDCFIQRYLYGDEHRREQPTIEHSCGSACQELPVRHLGHLSPVVFAACTGPDGGLTTVNCAAAQKHVSTSDPDVEFIDDSRRSSSSSLRTNQLSSLSRSVDNSMMNIRAMGMTYCINEADQGKKSVHTSISDLNEYIHDMENSYPIIPPPEEYVDTPEEAPPYLRYTCSAPASPFHQSPEPMSFQTMALRSVDVAGVKDWFRPHPRAYGAATSLYERHPVTRQHAGDPIADSFGVVARENSAILVLADGVNWGEKAKLASRSAVHGCIEYLNKVLYIDSFRSPARARPKNTSDVFLALLRSFHMAHSMILQEGGMLTTLTAAVVLPLQEDDQYVVCVCNVGDSLAYVYSPRHGVREITQGSHDINSMRDMRDALGALGPVDGLNPELSNLTCSMTEVQSGDIVFITSDGVSDNFDPVVGKFAIPKRTSMSSPPLSRRNPKEHPGLPGSASAGNILDGIRRPPPFVEAHQRHELTLMRMEDLLIHGLTGDEPPCRNAQQLCVQLIDFASKLTVAKRRILEDPDLYVNESSEHDKLELKRNEQRCRRRKVFERLAMVPGKLDHASVVAYTVGDYQLC

>tr|A0A8S1HN70|OS=Caenorhabditis auriculariae

MPSSFIRKHVRGLLRSKFGPSTSLEVAAEEVQPEVSATAENDGNDFLLQCYLASFESQREEFPEVYFGKTGLDIPCIQLQRLEPQVFASYTGPDGGLTQVGEVRGHQMVRMANLDDEMSLSVAGSSGEDEENEDEQEAPQGLLCVKKTDPKRNSPLLGSCSSLIGALDHEPSPGRGNAESFDWTQHDPKSAFGISVSLYEKNPLTGVNAGEPIADVWGVVGRTNNGVLALADGVNWGEGARLAARCAIRGAIDHLNQRICGDTLGDTTEVFHQMLAAFHSAHSLILQEGGMLTTLCVALVAPTKCGSSWVLCVCNVGDSLCFVYNRSYGVREVTLGSHDIDQMRDMRDAGGALGPVDGRNPQLHNLTCSMTFVEPGDIVFITSDGVSDNFDPVVGKFCVIKKSDGDNKENSHLPPREDRKISVSTKDNSNREFVRNRTCAASLPCVDAARRHELMLVRMRDVISNGINNQNRTPTSPRRTIYPAVSASLLCNRLIQFSTQLSQAKRRTLENPELYKKEKMSKSEQRARRKAVREQIADMPGKLDHASVVAYHVCHLNDREMTSPLPCAETPTTPVPHDDEASAEVSFSFENFSRVSEASCTETLLTRRSDASADLNISLWNPPPLEPLMLSEIVDKTDRNTRPVSPYEQLPVVSAREDDGGRKRKKHARGTVGRHTLGVDVQWLKRLVVSKKEPPETGVGPSVVEEIRGDVSLSSTNNERVSLRQRLRGILGSNRTLAEPSGQMPRYPQPPAPLASRERSATEQPLHANNARATRWPDGATTTALRPYDGYTSRGPTFDGRRRCLLLLQFDDVDPQNLPAVCSAAPPLQMRKTDIVASSRGRILLLIFMPLQCAHCYSRLSKLNELGQAYEDVRIVVVAPEYESNHIISRTQQQFPSLIVDRCENSWRIYAANNFDAFLVDRCGRIGAVIAHPRSDVSTTSDVADAVRDARHHARCGFCQYDVQYSQQQQRYEPPRVVQAYIKKGAYESNVAPTNAYRNQPFYYQNQRPVEPPRQPSNPVFRTHTTMRTPLPTTTTSPPTTTSSSDYDYYQDDVVATTVAPTKTQIPRPPTRKSSWPTANPTTLQDPSEAEVYERSELPLRLSDDSIPCAAYTDDICFQQQERLGKTGISKCCSKGVYLTDVCIPGKCSNSTLQLCCFQKFIQARYSCCEEKSQSEETGATNKFNKCCHDRFVTEDPCCPQTAASYHWQSVYDVCYPSTHVDYSAVRFEVHFSEGVRVLDLDANRQWEFECRHGKNRPQTAYFP

>tr|A0A8S2APY7|OS=Arabidopsis arenosa

MADHIVLSLQAPRFLIFPCSLHRSWRFPGYSVPEFRFSSQLQLAISISPSKSPASSSSPCPPENSAPEKFDLVSSTQLKDGSHVFRFGDASEIEKYLEAQEKARCVELEKQNAKIAEEASELSRKQGVEDLDLVSELKENSYGKKKLVSSNIEISSEKEETAPSNLSNVIKIKDRKRVRSPTKKKKETVNVSQSEDKVDAKIASVSNLSSIVSVAEDIPTSSTEEEEEEEVVSDKSEPINEKEITAKSYNVEPLSSQVMEKVSVDKIGDRETNGYQRITENPMEVQARPSSSIAQQEVAPVSTIEIDENLGVREKPVKTIEAEENLVVEPTATAAVSPDELVSTSEATDRSVDEIAQKPVIDTSEENLMKTFEAEKNLVVEPTATAAVSSDELISTSEATHHSVDEIAQQPVIDKSEENPMKTPVEPKAAHLSIDEITEKPVVDTPDVENDGENVASTIEDEITVRDTITDNGSISKTADDTKDEDLQLPAPETASLEPIEVASGREELVSKAFYLDSGFASLQSPFKALAGREDAYFVSHHNWIGIADGVSEWSFEGLNKGIYAQELMSNCEKIISDETAKISDPVQVLHRSVNETKSSGSSTALIAHLDNNELHIANIGDSGFMVIRDGTVLQNSSPMFHHFCFPLHITQGCDVLKLAELYHVNLEEGDVVIAATDGLFDNLYEKEIVSIVCRSLEQSLEPQKIAELVAAKAQEVGQSETERTPFADAAKEEGYDGHKGGKLDAVTVISKLATTILPFHLILVKPFSQSTTIPRKQDRVRDHGYDNYMEVEKKIRKVIKFHSLILSQPNNTIAISLLDTLARRLGLGFKQHEPGAFLLKFPHVFEIYEHPVQRILYCRLTRKALDQIRHEHEAVLAQIPDVVTRLRKLVMMSNTGRIRLEHVRIARTEFGLPEDFEYSVILKHPQFFRLIDAEETRDKYIEIVEKDPNLSVCAIERVREIEYRTKGIDAEDVRFSFIVNFPPGFKIGKYFRIAVWKWQRLPYWSPYEDISGYDLRSMEAQNRLEKRAVACIHELLSLTVEKKITLERIAHFRNVMNLPKKLKEFLLQHQGIFYISTRGNYGKLHTVFLREAYKRGELVEPNDVYLARRRLAELVLMSPRKAKVDAELVSYRNGLDGEDDVE

>tr|A0A8S2H1M9|OS=Didymodactylos carnosus

MFKTERAISVELINNLSAIGSISRPKSDSTLYLLAMKPSTQSVMHAKFGQYLVIVCDGVGGLRGGEIASSNALKVFGHAFLSQDFARLSFIQVNKWFKEALNSAQLAIAEQAELSPEIAEMATTVALSIYVPNRSIYTFSIGDSRCYFVVNGQLVQITEDHNVINYLNASNADRKTRERYRDKLQALTAVVSREVKKPPNFHSYEHPANVAEVILSCSDGMHNFLKEKDYVFDPKLGAEMYVKKLVQTAVARHKLPKGAMSVAVYRCIDTRISASESYDDVKRREVVIKILDRVNKDRPIERMEAELITQSRINNKHLVSAYDIFRDDQYIYIVMEYVTGRELTSKIADRGSIHYRDAIFLFKQIMEGIKTLHGYSDQILHLDLKPDNIFLSADGSNAKVADFGISMILDKTGGIISSDDVPMGTAGYICPDYRRKIKPNPQFDIYALGIILFEMLTGRLPFEEDPNSAKLPINQIFEKAKKYYPPYLNQFDETIPNSLHNIIFRYEERRSEPLIVTSLKPEFEKGKLMGSFSLELENYRCALMTGIVLKVIAGFYHVHVNGTTILAKPLGQLRFRDQETMLAAGDRVELDVENDQPVATIKRMLPRTNFLRRPPAANIDNSVIIHSLANPEVS

>tr|A0A8S3HBT7|OS=Rotaria magnacalcarata

LSNNTVACESGPDVGLVDIARHQKKGILKVNNTIKIAGQKNWQMPDQQFACGLSTSLYDHNPFTGEVNGEPIADCFAVCAQENNAILSIADGVNWGYKPRLAARCAIHGAMSCLNEKLFEKKLNTTKDIFERINLAIETAQQTIVKNEGSLTTLTISIVCQSNRRWFVCSAIVGDSNAYVYSPKRKSVFELTQGSRNLNNERDMRSPGGALGMTMTEKADLSNLTYSLMEIEQGDFIFLTSDGVSDNYDPCVSGCAASIKTPSFIRKSSHTISEMPPTPMTAQERHLYSLRRMYLSIAVNDEQLSALDICNRLLQ

>tr|A0A8T0DQZ7|OS=Paragonimus westermani

MMSVTNKFALNKTNGKSELGLTKSWSLKEGLRALRRSLTFSSSQENNENIPQCNSEHSDLRFRHRIFSQCDVWRALSPDELPDCAIFPLVAISALSQEVFASCTGPNTGILAVQKWLKVKPTNCTVLESWKPMRNRKADGISVSLYEKNPLTDRTSGEPNADAFVVRARCDSAFLAVADGVNWGRDAMQAARCAIYAVYEYLESHLYGTDVPKTKVTNTREAAQLLFETFSAAQERIVACTSGLTTLCVALVLPVIDVTILHDKQSTQSDHTYSSTNSLDGSSSTVTNQFALIVVSVGDSQAFLLSKFHGIREVTGWVPVPLSNIHKTAEDRLSTPVPVKDHSDPDSPSSCSTSPPVRDFRDAGGALGAVYKNGNPELNNLMCAVTLCDPGDLVILGSDGLTDNFDPVITQIAVPESPHLEFVDDQDEPDLDSVSSVETTDRPCHPAKSWFNPLSLSPPPSVSIWPRPPPPPPSPSANAITCLSSLPNLLNPNEYARTHLQSNTLQSHPVDPISARNLNFTNQLELSWSERRRYAGKELERIWHETDALTSSPSGIGGTTSSRDLCEALIAHALRITAAKRELLQDPELARLRMNPVKLKQLSLAMPEEVLSAESVRAQRKWLATTLKLTPGKLDHATVVIYEIGHYRGDENEVYEETISEGPPVVFNRPSTFSHSEP

>tr|A0A8T0EN88|OS=Argiope bruennichi

MGKPDGKKLGDCNKLLKKKHPVTNANAGEPIADAFAVCLRENSSVLVLADGVNWGEKSCLAARCAVHGCMDYLNNALYREGGRIETTMDIFVCLLRSFHAAHNLILQEEGNLTTLCAAVVCQMRNSDKFIICTCNVGDSLAYIYSQKHGVREITQGSHDIHSMRDMRDALGALGPVDGVNPELNNLTVSMTIAEQGDYVFLCSDGISDNFDPVVGKFAIPKRPEKPHRKSSEAGKSESSKQSSSSIKRQNSNSQDVTSKRQSRFKRQSSNNNNEESKKSLEVSSTDPPLPIVEAHQRHQLTLLRMEDLLNNGQNDNEKGFTSSQKLCEILIDFAMKLTAAKRKILEDPDLYNADEPDTTADQRSRRRKVGDKLCVVPGKLDHASVVACRIGNYKPHSPVHRSRDSSKNHSKSPVASSNKTNKSSGHHHHHGHHHHHHHSHQSNAVNNNNNNISKVSRGMKTDENFCRFESNI

>tr|A0A8T3RJ07|OS=Gammaproteobacteria bacterium

MAQSLSHKPLLGRYQLQRELGRGTMGVVYLGLDRAANRYVAVKTICLAEILEEAQLDEIKSRLFQEAETIGRLHHPNIVAVYEAGDEHDLAYIGMEYLEGTTLAPYIKKNDLLPVRKTLFLVATLARALDYAHRQQVAHRDIKPGNVMYAASKNTVKITDFGIASVIDAHGGTAGLAGGTPFYMSPEQVLQAQLDGRSDLFSLGVMLYQLTTGSLPFSAGTMDALMHKIVHDAPISLFDVCPDLARTAPYLGRIIDKALKKSPSERYQRGAEMARDLLISINQIRYKRGVMFQAGALEIINLSDRGRRRPNNEDSTASDAHLGLAVVADGMGGYRSGEVASAIAVSSVMSHVRKALDSARLSGRRRGATVQSSIVDQAITHANSMIYRTGQTEAQCQGMGTTVIACLFQGNNVTVGHVGDSRLYRLRDGSFEQITTDHSVVQELIDQAGMTPQQAAAAAPKNLVTRALGIDEAVQVDIYEESLYPGDIYVLCADGLHDMVDNGEIHLTLREYNANLVRAGDRLIALANERGGHDNISLVIARLRAYRRASFCWPRKLQRCVS

>tr|A0A8T3YJC3|OS=Candidatus Aenigmarchaeota archaeon

MLTAAAATEKGIHLRNQDAYLCLPGRGLFAVADGVTTGGRSERASKAAIDAVRNSKELAAAFAKAHARIRQLKDAGSTTLTACTIRGTRLEFVHVGDSALLLVRDRVLQLTREHAQPGSNLLTQVVGKGGIRPQQGAATLRKGDILLLLTDGVTKHLDEEELLALARTLRVQLLPKALLARARTKPKLYEDDKTAVAVRIG

>tr|A0A8T4CFR5|OS=Thermoproteota archaeon

MKTEHFESAELSDVGKKRKNNEDACLRIPEKGVFLVADGMGGVAGGELASEAIVKSVQKVYAKAASEQCSTLRGLIGLFEQGVNRASKWIKELADEKLVRGMGSTVVGLVMDPRQPTRAMALHAGDSRLYRFRNNELKQLTADHTTAAELAKALNRSEQSFRKEFQNELSRAVGLKERAELEQTPVEVQSGDVFMLCSDGLPRMVSDKQISELLAQHVQDSAEVAAKVLIEAANKAGGNDNITVILIKLGDVSVVDKEAGSVEDGEASPTIVPTIAPSDTLLSAAAPVESHDTPQTPRTSETPDTPSDKTPDTDRDSVTIEPAITPPPSVASSSATEKVAEPVGAAEPREEEHEQPVKEETPPVGLPQERGVDGGRRRLVAQAYRRKAGAIAVAAIAVAFGAYWMSRTPGTKPVTPTPPSLPEVSLSTNDVSKVIPESIVKIDSEPSKAHVFVEGKRIGQTPCIITNVTEGEVTYTLGTPAHTGSVTLAMAFNKTIETKVPLKPRQSDLQVSSKPSEAEIWSDGQKLGITPTNFLLTVNRYELVLKKEGLDDVALSLQVMADQPINTNVTFAHGAVILRSEPSGRIFATDGKHVGDTPYTNLMMKPGSFVWQVIADNYERTNVIVHVSNKGEQTVMDIKLQRGKGQLQLGANVAGVKAHWGDTHFNSLPTNVAVVAGKKYQVVAEYMGQLKTNSVEVAMDDDKVLTFTFEEIKKPEPRPIPTIKPTVKLPERQEQTLVWTNNMGMVFLKPAKWPFWVGTTRVTAEQYAQVAGGLGNCVESKDDGPPCVTNVSYADANSFAKSLTERLKQKNDLPAGCGSWRFTLPTASQWIKFANFAYTSEGLHVSLRELTKYGELCIPETAQDKYVRGEYSLMG

>tr|A0A8T4FLJ1|OS=Methanomicrobium sp

MIGFYNQTIPGKSHIEEGTQCRDANGVTRLENGCIIAAVAKGLSGTMNSAAAASVAVVSSIRLLKEHSELIDFSDKEALRALIYLSFFAAQKKIDSYAEINGISPQDCDTTLTIAVYDGKSVAYGHSGYGGGIITLDEFGTYRRLTPVQIGDTHDEKYSLRTGRGKWSFGFSDEERICSVLMATDGLFDVFCPQILAMMSNPVDIHEVRGYMDTGLWKFNTVAEHKSQKKLFDEMLRKNFPTVTDDKTAVLIVNTEVLPEVKDDGYYAETDYEAMSRCESDVLYYGSDGKEYLLKDKILDIDADNSSVYSVYNNAEILAVIYDDVLGKVVGQLSKIAAVIDAAAGNSAEKFLDVPAVLYDRNEMPKGYVKLRGHGDTDYEKYCRNLTIDLKQRLKVAINLTVALDRLHNAGLIAGDLAPAKIGIDGNGMVHLSNCESYIVRNAKKSDGFKFDPSRVDIVHMAPEIRSRIGNKFLYYRFSVETDLYSLAFLIYRLLAFGNYPSDSGDEDNTERESESIREVMGYLPAKIVSLFERAFIAGKTNPKARPSVSEWYFAMVNEYDSIIQCEADKKHYYWEEFTQCPWCRTEGRTSASMSNSTSKSASKSVSKSTSKSTSASKSGKSSKAKESGKSVKSSKAKEPDKSEASEKSVKSAESVKSDKAGKSVNAGKSDKAAKAGKSDKTAKTDKSNKSDKSDKSEKSGKSGSAKK

>tr|A0A8T4FLV3|OS=Candidatus Methanomethylophilaceae archaeon

MGGEAVLLAVIDGVGHYRGGDVAAALARDTILRHLDGYGQASAEAGLEKALTEAGNRIRKAAEKSGEHSQMGCVATACVIDEEGRLTYAHQGDCRLYVYQDGLLRKITHDDAPVGELEEQGLLTEEEAMCHPDRNIIYDMLGAGLHEASDGFIEHGTLHLGGNCQVLLCSDGLTDQVGSGEIKAVLDEDVSCTAKADLLVGAANGKGGKDNVTVVVATVDAPKRREDPPKQTPIQSETDNKNNVKRMKKNKTVIPVMTIVTLGLLAGFLVLFNNQKPMLDAAREAYAGRTAVNLDEDTPEGILTLILRDGGFYPDGRDAECVSHHLATARHKNGRPFRRLGELTGWQNQMPVSTARNRGGEALAALADSSCRRLGQTPEIAAMYGKDSVPPFPRTGDAVIEVRVARAGDGAPVPGVIVRVKEHFYEERTDTSGALAAVSPRDSVVGYMRTDDLGRVRIPATRGRSYSVLPVRPGFEYGSEKGTSGGSLERRKTRIVFQEREHRISPFPGTTFSDLKSSRSLTVRTPEEYLRRLAESVLLFLLSWWAALAAVLLRDRKVGHPSNAEMLPVLAFI

>tr|A0A8T4KQG6|OS=Candidatus Aenigmarchaeota archaeon

MNTNYETENLFNVNCQSDVGRRRPNNEDRCGYRINDTDKYLIAWVADGAGGHANGDKASGIIDNEFQDSDGHLAEMIKSANQRVMDEYPHSHSTATAVRINSDGHYEIAHVGDSRAYRINKDESGQYRIEQLTRDHNLGGKMSNVLTNSVGSDDMYVDEYPGTLNSGDYMLLCSDGLTGMVDDNKILDLVIGGERTAVDALVERANNNGGHDNISVVLIRYGQQNMDDMVTRELPKYTSQARGGNMMVFPLRRKRSSCGCLIATGLSLYLMGKLVTGMIDQKNHYSEEARQRNQQMTATLELGEKDTIYGHVVDELDLSQEYLGRRRTQEEELRVLKMTDEIMRKTGLDKASVKKIQPGEIINVPGKYDLNDDGKIGG

>tr|A0A8T4LKW9|OS=Micrarchaeota archaeon

MNLSILASSVKSEEIKCSDTSSAYIKKSKEHSCDDSALLIIKDEYTLIGVFDGVSGQPNASTASETALITIVNYAEKNFGKKKVEELLENAISEANYAVQAGGTTASIALILKDGSYYYANIGDSHIYKISKNKITRITKDDRDSSSFASYIKGRYYVAECIGSLITSIDTGNGKLNRGEIIFAVSDGIVDNLFVKVEGGIVTDTSGKEDLERIFESKNIKTMCEALAEEIKKRMQLKEEVIDNGILVPKEDDAAIVIFKY

>tr|A0A8T4R305|OS=Candidatus Woesearchaeota archaeon

MALESAHETRKHPRKEANEDFCSVNYFSESDVSLFLVCDGVSGKEGEIGSHLAANFLRDGIRKSIRKTLSEDKTKPKSIKQIIEEEFKRTSDRMLNETKAATTLELALFDHETQILHTAHSGDSEMYLIKGNAEDSPFLGARIEPFTTPHVDRQTGGPETRLGPGGYNRFDYQELDIGTMEDDHVFLFMTTDWIRERNLRDFTLKRG

>tr|A0A8T4T3Y9|OS=Candidatus Woesearchaeota archaeon

MHYEVVRGSIVGDQNTSHDRRRSHEHKKGNEDFYFVDAHNGIYILADGLGGHKAGKTAAEQATLYAARLLQEFKLDIDLGRYTSIQKTLEDVTRMCNKRLKDLGAARLEYAGMATTLDVMLIHNEVAHISHIGDASVYHWNGRKLKLLTEAHEIGETRQSEALTKAARILHGAGITHAIGLEDQLSPQYVEHIMLPGDCVFMATDGVAEFLTAREIAASLQSFPDVRTPLWNLVRKPQAVARAYARIHGCDVQEAQRDIGGYDNATFICVRRIPCP

>tr|A0A8T4U2L1|OS=Candidatus Woesearchaeota archaeon

MRLEVIAQSRGKFKCSNRDSHIARAFGDDYFLAAVADGMDGDKGYGDRMKVARASRAAIKFLEQSVQHDKDRPLKEIVEETEQRMLGSRLSKETTLTTVSIREGYLEIAQLGDSVAYLFRDGEAIRLVNQQTDRQLLEIVFGKHPSKITLEELLQVFNRNSQFYSGIINFDVLTIGIDLYNCSNSSLAEWYPVDEELKKQVRKIFGDKPIMYESVDDLPTDRKMAAKKIFAKIAYSWWNKLYVMVHHEWDAMYSALSYDKKVIVTIDTFSPKAAELVEDGAIKLKKDDVLVIASDGAFYHIGNKYVHDGSLKYYPGANWDYEAERLDRIAAKLMELLQQNPKKAFRDAIKQKYLDDDATLIVVRYRG

>tr|A0A8T5DGE1|OS=archaeon

MVYVYELVTAACTDNGKVRETNQDNYFLNQDNPSLITLFDGAGGHSGGEIASGYGSESSREIYAELDSLLGENLANGGEQVLTTIALKTKKYISECVNKERVKNPETKLDPLTTYVGLFLNPIGIANYAHAGDSRLYLLREGTLARLTDDHNFAFQTLKTKAEEEGKPFDEEAFKNHHMNNYITRSISNGDHDNHGNEVLVDCSYVGIQEGDVYLLCSDGLTDMLNDKTRRELDSPSGKITIKSIEEILNQTIKPEEKVSEKKLKQCANSLIYEANRAGGQDNITVILARVNGMNESFDLAEDAFFRNETVQASSETGQLTYDVLAENYRKVHAAFVEERKLRMDLERTTLELSDEHVKLTNDVHNLGLNLESYQYENANLEADVTERTGTIKILELEKEGLSRKVKNYENNPHKQGKNPRILGLLAGVALTLGVYGGSQYWAEHDLGSNISDVHASITQLHVEDYLLGASTIKLQYTLGKTSCGDLRFEVDGLNWEFQEGNIYGNLEHFSTAKDNLNQFVDEKCPKVE

>tr|A0A8T5HBD3|OS=Candidatus Woesearchaeota archaeon

MEFKTLTIPKPGETQSGDSCTAMQFEEFRKALFVVCDGLSGSRPNFASNYATRTISERLISRLSGQTTNIDAIIKEEMDKINDELKAESAGTTMELAYLDKESQILHTAHIGDSRIYLINGTQNLNPFTSAVPEQITPDQSHADENRGPSNCLGIGGVTKIHPNRFDLSKMADTVFMLMCTDGLTSYKVTQRELTNIFQNYTLNQELHAIMDTFEHLIEHPYGMITEGLEEGGITKGYWRNIAQHFAERGYQLDTDDYHRRRDMILPLIEPRNPDHLMYRALFADAYATAVRVHDDVGIIIADLKDGRKEFMQKTILTKRAVEVIKEPVMTTVIPPQTPPPLIGNEASIQNEVAVPATYDVEKTDTVLTGRFDEEDLTGDDLPETTTSKGPEVLPGQIPTTEGETIGYIPLDEVPSEEGELIPEPQTLGELELEDTQNPDYQNPALEEEKEEPELDDLRIRNSELEARVGELEERLELMRDYVPRNEHDEIITAHEEAKDELVQGFSSLEDELREIIANYEKSIIEYENINSQLKLDLEAKTSEAEEYLRNATGFKKNLEAALEREEGEIQNVQLYSQFYAEKIQELLRANSIIKDLTEKNSELETNIYEIRTELEGKIAEIEADKKTLVEGYEGLLKGYDERTNKLVQEKDELSSDYEARIQTLTKEKDAAIKEKEELDKNYGGRVSELETELEQANTEKDRLVGEKEELSIGYEARIQTLTEEKERLDRDYGGKVSE

>tr|A0A8T5Q424|OS=Candidatus Woesearchaeota archaeon

MAYEIIQASVIGKQKRGRGKENEDTVLVDRKRNIYLLADGVGGHSYPKKASYLACQAAYFELKSLYSHLKKKKFDLKRIPEFMKDIFYKANELISIKDNPDETCMGTTLDACIIHEDVAYWAHAGNGRIYKFNREGKLVKLTKEHVTYEEDTKKCSPAEQAVIEARLGLDSYIGCGADIEVDAGIEPIKPGEILLIEDDGLSHTVSENERVHALKMFHIARNTLLAFSENPNEIAEAHARLELSEKDENAEGYDEKFRKQKEKSRKELGGMDNTSFIAIRRGI

>tr|A0A8T5R7I2|OS=Euryarchaeota archaeon

MAGMVPIPESSSSPHQETHQVGPWRVVVRSDVGLARSRLEDAWKVVKGLTMGGRPVDAFAVFDGLGGLPYGQEAAWAAADALGRVVAASTGPDDVLPALNKVVRETGGATTAVVALMYDDEPGHGVLLCVGDSAAYMRDAEGRLQILNPKDSVGPHVLSDFLGNLNMKGHRRPIEVPVDGSLMLCTDGVDGVADPARLSTVLGANGTAPVKAIDALFSEIHDRGAPDNATAVVAYRA

>tr|A0A8T6CRY9|OS=Gammaproteobacteria bacterium

MIEGRDYAIAATLGARGRQEDCSGLNDRPPPSREGSPGLLAVVADGMGGAPAGDQASEIVVNAFLSGYGRTNLPSAAERLVFALEHANETLGETIRANSAELTGERGESPGCTLIASLFFADRYFWLSIGDSLILRYRGGKLERINPLHVYAFELDELVRRNEISREEADTHPDRLSLTSAVLGDGVEKLAFVEDHIMPQDI

>tr|A0A8T6CXE7|OS=Gammaproteobacteria bacterium

MSIHIDVAGAQIAGSRKRQEDFFAIHKDVALHGEVKGILALLCDGMGGHVKGDIASKLACEAFYGCFIDYIGLDLKDRLGASLVAANEAIRAAAEENPEHEGMGTTLIAVVVHKNLLHWISVGDSPLWLKRKKELRRLNADHSMAPVLDKLVEIGEITMDEAKADKGRSQLRSALTGNEFNLVDITDEPLTLALGDDIVLASDGIRSLETDEINRLLRKGRSRTTQQRALALLEKVSRKNQLPQDNATVVIIKCGSEPPRIATKFKQVASKFRLIFS

>tr|A0A8T6IYY2|OS=Chloroflexota bacterium

MAQQLANSPLKKLAGRACGAPINRRVGNCMTIFAAAIGKRIGDRHRAKAKQSQDYGCYRLLPGGTGIVAAVSDGAGSAPEAGTGSLVAAHYATMGAWRRATEKSAEPSACVRAGIRAAREKLEERAKRDRRSMENYHTTLIVAAATKNKAAVAHIGDGASIAKTGGQYKMLTIPARGEYANETFFITMDGYKNLTAYNEVHEPEELILFTDGVQNELIDFKGKRADQEALLRLKFLDIPAERNQPTTEAGQLGIRSTTHHLLNQWLEDGQTTHGDDATILIIRPPSEGK

>tr|A0A8T6NV55|OS=Chloroflexota bacterium

MTTIRVSQEEETRLSTEFTPFGTVMYLYARSTDSVSAGTIGQDYVTFRYDEANVAFAVCDGVGQSFMGDLAARILGDGLIDWLLDLRSKPADAGAFRTMVEGALADLTAVGRETVANYQLPDHLPPILKQALEMQRSYGSESMFVAGRLALRGDPPFLALAWLGDSPMAAIDFNGELVDLGPRGSTAARWNADTGVKGTVNTWVGAADAVARVAGYTDGMTIDSVPTDAVLSRLTTEWQSNPPADDASLVDIRLKPSPQTTGKDDPEHLVRIAEFVPDETRPIPLPGTLKQRTPTPEKPAPPEPDEQPEPASPAAPGQPVGEWRSLDESGEKVHKPPATADRAAARLEQIRAWQSAARERLIGARLALDQIERLLADNEDDQTST

>tr|A0A8T6NY34|OS=Chloroflexota bacterium

MSFFNPSKRTPIRTQTVPQNIETPVTFVSTNGAYVRYTYSRSSDSINNQVEGQDYLCFRYNDQRLTFVVADGVGSSFCGNLAARIVGDQLLDWLWSLDIEYYGSPSALSEAAVSFLNRIQKQAQHEVEEYEIPSEVTGLIRQALESQRAYGSEAIFAAARIDYPSELIPNGLVSVFWMGDTQIHVRDEMGKELDIGGRWENANRWSTVQGVRGQMTAWMHELKGVGRVTSFSDGLMAHAHDLLGYSDSKLNREIYLGARLPTSDDVSFIDVVLRTPRYEGYPNPDEPDLDAERPHLEPIWNPTGQSSYEIRWTWQRGDSGDALQEPMPRFLLQEGTNPALPDAKVIDVPADQLSWRSSNGQSPGHYYYRVRAILRSGIVTPWSDLRQTRVAYPPPVAPQLTVINPEQAPILQWEAEGEGLEYTLQQAREEDFSDAVVVYDGRGTSWSVPMHQSKPGAYYYRVRAKSDGGEGPWSDPQSVEIVLPPPPKSQLGNPSYGYTPGNYDLRWQPVPGATYYELEQVEQASGEEELIRVEDTTISFTSQAVGDYSYRVRACHDFGCGEWSNVQRVQIAPVAPTDAPELSLEGPDDDDVYTLSWTGVVGATSYIIETSEDASFDNARVYSQNETSLTVARREPGTVYVRVCGTNRGGDSPWSNAVHIRVEAPSPGWIEAKLDAPNQQINLAWGAIGGRTTYRVEMASVPANDDQFELAYEGDEVQTAVDVPDGALELIFRVRAEAGGIESDWLASQPVRLSPPLGAPRLEQPEYDEKGVVKLSWTGIEGATSYLIEVARDAAFTQITWSEPMEETSCLFHPPSSGEYWFRVRASKGARNSDYSDSAHIVLRRPPPPRMWQRDPVAANSRYEVTWKGVLGSQFYEIQSSSTSTFDPDKTKTVRVFHPEQKLEVDGGAPGVVYYRVKAVDENSQASLWSVPIRVDIVDQS

>tr|A0A8T6NY75|OS=Chloroflexota bacterium

MTDLLVGRAFSVGGRRVYEDRLITETITLASDRMLTLAIVADGEGGEGRGDRAAQIAVDAVLSHLRENTKRDGVTALLSAAMEHASEQVHSFAWEQRPDEVRTTLTVAAVSEENVLFIGNIGNGGVYLHRLDEPDSGLRKLTQDHTLFNSQMFEGASRERLQRKHEDQSPSLMRVLGRQLDVEPDMGIYLDIDNYLRANQRGVGGLELRPGDSILVCSDGLFSQSENGDRQLVTEGEVAEILSLEEGDSAARMLVNLANSREPGDNISAAVLQLPDPRRKEVKQRRRIVNVGVVGTVAAFMVAALLFTVFRQGQQIDAISQLATQQAEFAVFEQ

>tr|A0A8T6P7I6|OS=Chloroflexota bacterium

MSSTLVYGQGFSIGGGQIYEDRVVVKAVTLSDGTNIVVAIVADGYGGEGLGDRAAQVAVDSVVAYLEQAVYEDNLPLILYAAIEYAGDVVNRLAWDHRPAEIGTTLTIAAVTESNTLHVANIGNGRVYLHSLDHPELGLRQLTQDHTFAHTVLRDTKPSQRRLNQTFDETSGLIRVLGPRAEVHPDIGIYLDTDNSQVAEQTGILGVELLPGDSVLVTTDGLTRWSDSSRSTAVTDDEIKEVLATEEGDTAANMLVTMANARASDDNVSVAVLQVPDPRRVARKRVNRAIVFGAIAALVTVLVGILLFALQQQGRQVDAIAALATQQAEFSIFERTAIAETLAAVPTATASPSPTPRPPLLPNEVAALFVSGERSVVIEGQSVQAAFEDTYLAVNHTGELEDGHIFMLPPSGVQITNISNETASFFLTLGSRVFIETGRYLGGMIAVLDPLREVRFRSASCMSIDYLSEAFISAGCYSGTCEYSVDIGRTFQQIPVGSRVVINPETLDGTIEPIPIADTLFFNGILFQTGAGMAIQNECLVSYLPTPTPSPTPTQPVIATFTPVVTLSIDDSYGAFDSRLVPPFSDFRDPDSPSGSDVRMATLVVITLAGLGFLAVTLWLVISRMDLRSGKPAQVEGK

>tr|A0A8T6QXV4|OS=Lyngbya confervoides BDU141951

MPIAPPRETAPSEAVSTHSKTAVSPSATAVATVATLFTPQNRLKDQERYQLRQPTDADKPLNADVTILSILDCEPAADSPLVEFLENAPDDLDQAAIDELIPPLAFPYWELQESLYPVVPELQAAWQIENYAITVIEDRTSWRTLTDLASAGVVEPLELVHWLYEIIALWMTLSPFAAETSVLAPDNLRVDDDQVVCLQRLIFNPDETPPSLETLGQLWQTWLTQSSLPQLEPLEDLVTDLVAGLLDDPTTVQTRLIEIADRLSEPTSAAEPAVETAPSTTTTLPLAATDTPGETVAALSIEEIAAEAKASAADSPLLLVEDLLLGAELDDAANSSPEAESASEEGGLGDLPTMALPMKLHRLDDAGRTHVGRQRAHNEDSFFAHTQVQRLNSPAGAQVTARGLYILCDGMGGHSGGEVASQLAVDSLKEYFAEHWQADLPDEEVIRTGILNANQAIFDQNESEERAGNARMGTTLVMVLVNDHQVAVAHVGDSRLYGLTRQELTQITVDHEVGQREINRGVEPAIAYARPDAYQLTQALGPRSNQEIAPTITYLTISQDTLFVLCSDGLSDNELLENHVESHLKPLMRTKVDLDDGVADLIDLANEHNGHDNITAIVVRLKLRPNLNAIADSE

>tr|A0A8T7A9M6|OS=Gammaproteobacteria bacterium

MQIETAKASLLGDREENQDRVGVLRRGDSLLAVVLDGMGGHSRGDLAAQVALDCMLKKFAEAKLPLMQEQRFLSELIFDAHEAVIKAGAQMSMDMMPRATAAVCLIQRDRMLWAHVGDSRLYHTRKNKLLTRTRDHTHVEMLLQQGLIKESQINEHPMRNYVESCLGGEIEMSILTESGPMTVEAGDRIMLCSDGLWSAFSDTEIAEHLSRHDMKTQDMVENMCELAVNRCFPMSDNTSVVVLKINET

>tr|A0A8T7ANZ2|OS=Gammaproteobacteria bacterium

MTDDNATTVRDQGPLTEVQLFGGSFDDELKLLLNSYSLPTGDVAIGLAIDPGKTSPIEDSAGLVPLSDGALVMMVADGVGGLPTGWKASGVVLEQVRDQLAKADIDDARRRTAILDGIEQANSVLRELGTGTSTTLVVAIVRDQQVRTFHIGDSSAWICGQRGVVKLQTTPHSPIGMALQAGFVNEKEALQHEDLNLVSNVIGSGDMRIEIGTYQALAPKDTLLIASDGLFDNVTEKEIVEIIRTGGVADSLRELFELTAKRMAGAHKNKPSKPDDLSVILFRPNSPSSG

>tr|A0A8T7BE57|OS=Gammaproteobacteria bacterium

MGLEFTLAGSQIDGDRDYQEDAFLITHLTDADDKPAALIIIADGMGGHAAGNVASNMAVQAFNKHVSANYPTDKPDEILHECVIKANNSIAETVKETPALAGMGCTMVAAILESGKVWWASVGDSHLYLIRDKKRQKINADHSYGGFLDRMEAAGTPIEPEPGLARNMLMSAVMGEELGEVDVSKEPLELNDGDRIFVCSDGMDTLSEGKFNQFSDWSENPKECTEALLTAVEDEAKPKQDNTTIVVVDVAEKAATETPVAPAPEPVAESAPAAT

>tr|A0A8T7BLN3|OS=Gammaproteobacteria bacterium

MSVEFEIASDQIDGARDYQEDAYMVNQLGESENGDICSLVIMADGMGGHAAGNVASNMVVATFNKTFQSRFPTKDVADALTESLTRANDQIGASVKETPALRGMGCTMVSAYLQDNKLYWVSVGDSHLYLLRDRELIKQNADHSYGAYLDMMKEQGMEMDEQAGMSRNMLMSAMTGEEISSIDVSETPVKVRPGDRVIIASDGLDTLGAGAIIQYSSWSATAKECVYALLKAVEDANKINQDNTTLIVIDIKEKVVQPGSVQIDQHQQQKAEPVQRVADVSLPEPPREPRSFKWLVWPLIILILGAGGYFAWKQGLVQQGIELVENMIEVNKPEILPVIEPVADQQSDDSGFVEDEIPADPVVEPVDEAEPVYVDKPDVFRDRLRSGGRGPTMIKVPAGTFRMGSPSGILSADEVPRHEVTVESFM

>tr|A0A8T7BR68|OS=Gammaproteobacteria bacterium

ADSDTLVLVVADGVGGLPSGSQASKILVEKISKACINYRKDDLELRDALLSGIESANHEIIGSSSGSATTLAAVEIQNSYIRTYHVGDSAILVVGQKGKLILETVLHSPTGYAVESGFMSEDEALLHEDRHIVSNVIGASDMHISMSVPVKLKKFDTLMIATDGIIDNIDKPTIIQTIRKGNLNDCLNKLLDITNERMNSGDNSYKPDDASCILFRRSPQDE

>tr|A0A8T7DNC9|OS=Gammaproteobacteria bacterium

MIDIGCCAPDERGAKSMSYRWWSSCKTNTGRVRQVNEDAFLDLGDCGLWIVADGMGGHARGDVASRLIIEAFSGLERPSSIMEFSADVRDRLKLAHHRVKQESDRTDTQQIIGSTVVVLLVFKRQWLCLWAGDSRAYLLRNGALQRITKDHSVAQELVDMGKLGIEEVEGHPYANHITRAVGANHELVLDERSSELRDGDAILLCSDGLNKELSE

>tr|A0A8T7DRX6|OS=Gammaproteobacteria bacterium

MSINNNIEIIGLTDVGLVRDHNEDSIGENRDLGLAVLADGMGGHRGGEVASAIAVSTILESLDNELKNIKSGDIDEVTGYRLESIAVNDAVTKANENVFTSSNENSQYRGMGTTVVVLLFHDNHFTVAHVGDSRLYRYRDNELVQITCDHSLMQELVDKGFYTKEQARESLNKNLVTRAIGAEESVQVDIHEDAALPEDIYLLCSD

>tr|A0A8T7IQ34|OS=Gammaproteobacteria bacterium

MRFQVQRQGDREYQEDTLWNGVIGGAQVLMVADGLGGHDAGDQASALMVESLRVCLEAEGDLELTEANAKQWFQDALVRFQEHIVQIEGAKDAHTTLACALIKGQTLLTITVGDSRVYHRSNQGLWRTKDHSVVQMLVDDGEITEAEMATHPEQGKLYRSIGPTKPAKPRVKSREYDASDQVLVCSDGFWEAFSELDLNELLRNSTQQSLDAAADSAALKFSPKSDNISAVIAIGSNSDEESNVSDANDNMIEPSLDISNRSRDRFLIGLSIVIAVILVALVMRFSGEDKSDESLPVGIEDPSQREACLLDGTFLAETNSSRVTFRSIETGEEFEVILPLSEGKYEVVIDDSKGDCSAELDEAEEARLKSKIGNSI

>tr|A0A8T7K0R7|OS=Chloroflexota bacterium

MANTHISFGQAMDTGLDEKAQRADASTCIVSALNSHNSTTDCGIFAVAAGVGSKEGSNPQKAAAIAIQNVAAAYSGVYTTILADENSNTAKFSDSMLTMAQKANKAIQAFASKEKSSDQASLTAVMIFKNTAYLAHVGNTRAYMVNGKTVKQITHDQVKEDNKTLASALGQAADPEVECSSHPITPDTKFLLCSANLWNFISEEHILKYLTEEPYAQQACEKLVTFAKTNGAAEHVAAVVVYMPEA

>tr|A0A8T7L0U0|OS=Chloroflexota bacterium

MSHPEHAIRLRSSARTSTGQVRENNEDNIHLWARGPNVLAVVADGMGGAAAGEEASRLAVEAIKSGLIVRDGRSDEALEHLGDEQLFLELREAIQDANQRIVEKAASAPEFRGMGTTITIAFVRGTAAFFAHVGDSRAYLIEGGDGHITQITSDHSFVEALLAAGHITREQAEEHPMRNVLYRALGQGDDIDVDLYQENLHVGDRLVLCSDGLTRHVRPNEIARLTLESSNPDTVSQKLIDLANERGGEDNVSVIVISVERNTPSETARKEQAAMIADDEDETLVLKSRVEARYAAYPPPERLASRPDAPPPVAHRIAAEPVGDQGLEAEMPSDTRDQAIANRPHYLAAPAPYERHSPARPQEKQGEGHDTLMPDQ

>tr|A0A8T7M897|OS=Chloroflexota bacterium

MFNKDIPGSGEKKKSKSSRSKTRKEKAPVVTENLPVVAPTEELTRPDPDMATQPLSSMDFSQLEVKPIDVTVNGTSSLIPQKVKCQQIEAAFASHVGMIRDNNEDSLTVFMGTIPRSENVPEQLFGFFAVADGMGGHENGEVASNIAIRTTSKNVVNEFYLRALSGLKPGSTGETPGEILVRLIENTNQLIIEQGQEARHNMGTTLTCIIILGSMAYVGHIGDSRLYGVNKETRQLQQITKDHSLVARLVEAGALTAQEALDSPQRSVLYRSLGQRLEMSADTDFFRISEYTHLVLCSDGLWDMLPDMSISYIINKYDEPATICQELINSANSAGGEDNVSVIVIKL

>tr|A0A8T7MEQ0|OS=Chloroflexota bacterium

MAQAQNGGLSIRFVEKANIGRQKKEQQDYHGHLILPQDESKGQSAIYLFVVADGVSMGAAGALASRTAVEVLLHRFGQLVSRGATDLAEALEDAFNTASDEVARLAESQPGMATTCAAALLAGDRLITAHVGDSRIYYVQKLPPEALAKPEDATLKLKIEAKPETKLKTKAEVKPEAKLKAEAEIKPDAILETDATLKFKIEPKPDAKLKAEAEAKPDAILETDATLKLKIEPKPDAKLKAEAEIKPDAILETDATL

>tr|A0A8T7MK43|OS=Chloroflexota bacterium

MSFSQSEQEEGKKRGRPRISGGKASGGNRSRNREKVEPTTDTSTPTNGNSGGSTAVLEASDVIAVAPIVAPPAEEIQDPVATKPSSITVTPPPPLRTEPKYNPNDGTLELPELDFELNVQFPNTDFGAGEGPGTVDKPRILNCGTIECAYASDVGRQRTNNEDSAVAFLGMVERSNTTFGFFALADGMGGHENGEVASNIAVRKMMDGVMRDFYLPTKEGRPLGIAGETPTEIMVSLINDANQAIVTEGMSRYRVSMGTTLSCVILYGPVGIIGHVGDSRIYVVERETKVMRQLTRDHSLVQRMVELGQLTQEETLDHPQRSFLYMSLGQRGLVNPDTETMPLADASHLLLCSDGLWDMLDDATIAQILTSNAEPDKACQQLIEAANEAGGGDNVTVIIIKL

>tr|A0A8W8N5T3|OS=Crassostrea gigas

MAELTQLMDQEIPDGRQHLQDSYKNLEKVAQYCRDNYVQATDKRAALEETKNYTTHSLASVAYQINSLATNFLKLLDLQQSQLAEMESGVNYLAQTVNIHKEKVARREIGVLTTNRSSTRPTGVKNGIIFPEQAEKPTKYQRKPIDYASLDELGHGVRVQEPGLMLKGSTKPARSPSISSSSSSQAPTSHPPTPPMNRAGGTMGRNAGSHYRTPAPPVAPPSVPSVPSQYGPGPNSYTVQTTGLPGQSRSSTRGSNYGQMMAPSMAPPPAPAPPPPVGMATPMSHPGSGRMSQSAVDNLPPPPSPEVQQGVEVPNGILEASPPLPPPPQDNQQYPPYLEHPLPPPEFIDDGDDIGYRQEEDPYAATGPNFALPDWVPEHYLEKVIAIYDYNAEKEDELSFFENSVIYVLKKNDDGWYEGVLNGLTGLFPGNYVEPCMDKTMTNFGSETWFGKSSEDVPLLSLDEFSDEVFASYTGPEDGLGFVFRDTSSNRVPSNTYIEAAGVKNWNIKDQRAYGICMSLYEQHPVNGKMSGDPIADAFALCSRKNNCILLIADGVNWGEKSRLAARCALYGAMKYINRKLFQDRQQPQNTQEALVLLRQAFDAAHATILEREGGLTTMCACMVCPVKNSQQFAICCVNVGDSYCYIFSHRFGIRELTIGSHDITSERDIRDAGGAIGPVCGNDPELQNLTCSLSFCEPGDIVFLTTDGISDNFDPVVTKSALPKKINDANSNELSPSSENSGKPEMEPSERHLYAMKQMERVVYEFELVTEEQCSAQELCGALVQHVLSLTDAKRKILENPQLYVRRKLTMKEKEKRDSDIVNQMAKAPGKLDHASIVAFEVGVMKRDEEEMENIPLIETVQELDSVSESDECPSTSVTLSPTPKKKSSRPRKLFARIKNLSVNTTNGHTSSSLVTSPKSASVSPKKFSFKRPRSRTAEAMSPGSPEPVSPPEIPSPSLVSPSNPFPVPAPRRKHRQSSHESSV

>tr|A0A8X8I9D9|OS=Hydrobacter penzbergensis

MNIRAFTLNEIGGRKNLEDAILPKRYLPQDIPLFIVCDGVGGSSFGEVASDIATRCYYNILATAVVDSEQAFKNKLDEALASFQQQVHDYILTHPAAATTSTTLALLLFSQHNAYIAWCGDSKIYQLRNGISIYKSKDHSLVATLVAQGVITEKEALMHPQRNVITRSLSTHTHPSDITYTVLSDIREDDWFLLCTDGLMEQFTEDHFATILFPYNPFSNYSEVIDALCRNNTKDNYSMYLLHINSLKKKVAAKSRLFPVLLLLLLIAGAWYAYQRSSGNRHTQISSPMKPDTSKSIMMTHPQKKEAVQQDSVAKQPGKPH

>tr|A0A914AUP1|OS=Patiria miniata

MAGLFRVRKRSDTVHVPGTRACQPRPPVPDSRALRRQEGDCDDKGRRRSLSDPAKRKAMSKIGVGGLLGKLGNLLSLTPTAPRVYYSGKSYNFLPKKKLVPFSREMVASYSGPHGGLTNSGPILPDSAGENTALSPANGNAVHGTNGVGVKTQQQQEFERNEKKPPLDRDVSKWRQRNEKAYGKSVSLYEYKNSDKKECTGDPIADVFAIHATRNSCIMAVADGVGWGQAARRAAQCAVKGSMEYLLKTLYSKSKDFQNEPFTTTDVFKCMTESLDEAQELIISREGSLTTLCLAVVADVTSDDTTSKVLCVINVGDSLAFVNSQRHGVKEVTVGCREYNLHRNIRFCDGALGPCFGENPDLENLTFSYTTLEEGDIVFLTSDGVSDNFDPVVAKRAVANTDFKSNKSRAEMIFSDDQWEAGNREDGRLPAATPRERQEEMTRMMDEVIHKTEMPSEDPYSASSLCSKLLQYTVNLTERKRAAHEATNNIISICEKEHLETQTMMTGQEALSRHARSLPGKLDHATVVAYVVGGTTDLGEPKPRVIKEVPARDSSSYFDVSVGTQQCKDFISLID

>tr|A0A914F8G2|OS=Panagrolaimus sp. ES5

MSSFIRKRVRGLLKNTFSNASNTHNNDENHRGRNNHHSNGQQQNDIKASDFAEDNVIYERILTGRHGKKVEIPETYAGKTGLDLPVIHLAPLKKHIRSCFTGPDGGLTRVGQMRQQPIVRMANLDDELSLSSYEDDGDDDDAEGDGRTARLNMSGDGGMNPSHSMEALGFHPTDLAPCCSTDTFDWTTCNPLRSYGASTSLYEKHPVTGMNAGTPIADVFGIISRENNAIIALADGVNWGEGARLAARCAIRGALDHLNAAVESNSFETTTDVFHCMLGAFHAGHSLILQEGGALTTLCVAFVAPIKNSDNYALCVANVGDSLCFVYNHASGVREVTLASHDIAQMRDMRDAGGALGPVDGRNPQLHNLTCSMTFVQEGDIVFITSDGVSDNLDPVVGKFCVIKKDESEKENCDIIEEIAPKTSKAQ

>tr|A0A914LYS8|OS=Meloidogyne incognita

MSSFFRRHVRGLLRTTFSAPQSTETISSIATNNADDDFDNHDESEEERLLLSLDHRLGPAGTSSENNFQTRNGKKRQSAPEVYSGKTGLDLPNIHIGPLSLPVKACHTGPEGGLTCVQPMRQRTIAKMAKLDDEEFSLSSMDEEVDEREEEEEVEVREEEEDEAREEEKEEVKERDKEREEEKEEEDEEENKGKEVGEEVKEGRGENEGERFEEENEEENKGTEVKNEVIEEMNRFRPVDEDQEDLSGRYQQQTQLIKPTKETKFLEVPKKENKKIKKTTRKSRGGGKALIQSADSIEALGLVGVDDFSPFRNGSALEQLDWSSWDEHRALGLSTSLYERHPVSGLPAGHPIADVFGIIARENNAILALADGVNWGDGARLAARCAIRGAIDHLNNAIERQQLNTTNEIFHSMLGAFHAAHALILQEGGALTTLCVAFVAPVKDSDSSVLCVCNVGDSLCFVYNQTHGVNEITLGSHNIAQMRDMRDAGGALGPVDGRNPQLHNLTCSMTFVEKGDLVFITSDGISDNFDPVVGKFCSIKKPRDEAEEEVTETITSEKENNLTTNQQQQRNK

>tr|A0A914Y8B7|OS=Panagrolaimus superbus

MRQQPIVRMANLDDELSLSSYEEDGDGEGEDDDEGDGRTARLNISADAGMNPSHSMEALGFHPTDLAPCCSTDTFDWTTCNPLRSFGASTSLYEKHPVTGMNAGTPIADVFGIISRENNAIIALADGVNWGEGARLAARCAIRGALDHVNAAVEKDSFETTTDVFHCMLGAFHAGHSLILQEGGALTTLCVAFVAPIKNSDNYALCVANVGDSLCFVYNHVSGVREVTLASHDIAQMRDMRDAGGALGPVDGRNPQLHNLTCSMTFVQEGDIVNCTKNKVRLSHTDCI

>tr|A0A915D4V1|OS=Ditylenchus dipsaci

MTNSCSLEALGFTQTDLLPGTSTNNFNWKSWDEEKAYGLSTSLYEKHPVTGTNAGTPIADVFGIIARDNNCILALADGVNWGEGARLAARCAIRGAIDHLNSAVEKNNFENTTDVFHSLLGAFHAAHALILQEGGALTTLCVALVAPVRNSESSVLCVCNVGDSLCFVFNPLYGVREVTLASHDIGLNRDMRDAGGALGPVDGRNPQLHNLTCSMTFVEEGDLVFITSDGVSDNFDPVVGKFCVIKRLDTEKENFRVPLDHKTSSQPRMGTNAFHKTKSVPATSSCAISLSSTLLPNVALLHCRVSML

>tr|A0A916D7U5|OS=Anaerolineales bacterium

MNEMIPNKLTARLYSWLSRKTTPSAIRRVGELPLAIGSDIGVVRTENQDRVAVLRMELGRGRSFTAAVLCDGMGGMAEGSACASQAVASFFAACILYRELPLSARLSRATQEANSSVYAVYHGRGGATLSAIIHDSADGVLGVNIGDSRIYIYQDGTLEQLSVDDTMLGLLPNNKDAYQHRNEILQFIGMGEGMEPHIVDIPALHESIFLTCDGVHFVERQTMQLIVDSAKDSALAVRRLIDVAKWCGGHDNASVIAFKPFSARLPFSNDPGTVQVWDPYGELQIVTVDMYNNERIMNKQSWEKKPAVQKTAEEVAHPTHLLKKKRQAKKKVTEKIVPKIDDTDIASERPQLNIYFNADVDKDGNRD

>tr|A0A916FPI7|OS=Gallionellaceae bacterium

MKFAIYQSSRIGGRKYNQDRVAYAYSDRALLLVLADGMGGHMHGEVAAQLAVKAFVDAFEQAALPRVPEPDAFLREIMQRAHMDIIRHAREQMLPGNPGTTCVVALVQDGQVHWAHAGDSRLYLLRGREVVARTRDHSVVQQWADWGVIKPEEMRTHPDRNRITNCLGGVEDMFYVESGPAAPLQSGDVLLLCSDGLWGPLNDAEIAAELLDTPLPETLEQLISKAIGREGPRADNSTAVVARWGDAENEHSAPAPVSEVLDCN

>tr|A0A916NPV0|OS=Leucobacter soli

MNARREQPLLEVSARTDAGLRRRQNEDWLLAADPCFLVADGMGGHEAGDEASRAAIAAFSEEFTAPGPATLERIDAALARARSDVARLAARRERGAGCTLTGVIRIEHDGAPFWYVLNIGDSRAYLHRDGELIQLTRDHSLLAERLDAGRADAASTPRNLITRALGSDDSRHDAWLLPIESATRLLICTDGLTSEVEDQRLETALRGADRPARLVERLLDEALRSGGRDNITLIVVDVLSSGSGTLGSAPSPTQPGTAAAGTDTGAVELEAAGIDDGGDDREVDEITIERTRPARRPVTAGAR

>tr|A0A916Q4K9|OS=Anaerostipes butyraticus

MAFRLKKHKRLKECDEKVVIKEKEIFADTEDKGKENLKEHEEEEEIKLAPAPKESTPFFGMNQDIVYWGFSQRGESHIKNDTPCQDRCKVLVANHNRPIIIAAIADGVGSCALSHYGSGIATELSVAYLKEKIENYEGEEFEDKVIGDMLRDTMQYANEAVRKAAEEMEQLEYSFQSTLTITIYDGSTLYISHAGDDGVVVLTEDGKLELVTSRIKGEEASSVYPLQAGSQYWQVLKVDREVNGFVMATDGVLDAFVRGEKEENRVYYPFIQPAFETKQTKRKQIQDILDFYYDYMAGKEYRKAVTDDLTMVVVTNQKKLKKQNFPVFDEEEWNQKTKEYQEKVNAALYPDMAKFKKIEDDSEDEEDGTDLICPYCHEYVNEGDKFCRNCGKKLKKKSKKTKDEQEPDQISESQKESDLNKSDGDLQKTNRKSRKKKRRRVKRKYKWKKIIVLCVILILFIVLLVAAGFMIIGAL

>tr|A0A916Q7W1|OS=Anaerostipes butyraticus

MHENELNEEDKIMEDFSETGNSVNEQISKKNDIKENKKSTESVLPPIILPDPSYAFLHNISILCFSQQGESHIKNNVPCQDRSGFKLINDKIIVAAIADGVGSCALSDYGAEIAVNSSLMFLEEYFNKEMKQKGFKFDDSPRMGQILREMMLYATDCVEKRSMELQQFSYSFQSTLTVAVYDGNTLYFAHAGDDGIVAQNQKGIYAMVTSRHKGEEVSSVYPLQSKNTWQFGKVNDVVAFMMATDGVLDAFVRPAAENNRIYYPFVEAVFYDIQKNEEDVKKNCRDWYEYMASESYRKSVTDDISFVSVVNHEVIQKSVKPNFKRQEWDKQTKKYEKKRKAALYPSDSKTKSQQRISEKLSEKGKSINTEEAAKQAKIAAHKMNDGMKELASASADILLKGVYRATSYLGETMGQISKDLKKRNDQRNSAEKGKNDESND

>tr|A0A917P301|OS=Streptomyces brasiliensis

MTLGRRLALLIATYEYQDDGLRALTAPAHDAEALAAVLKDPDIAGFEVTTLINEPNHRVGEAIADLYRDRRRDDLTLLYFTGHGLKDDDGRLYLAMTNTRRSSLLFTSLSAEQIDQAMADCMSRQKVLILDCCYSGAFPAGRLAKADPDVHTLERFQGRGRTVLTASDATQYSFEGSRQPHGTAAPSVFTRHLVAGLRDGSADLDGDGDITLDELYSYVYDRVVDEMPQQRPKKQDNVEGRMVIARNINWSLPTHLCHALSSPIPTDRLGALDGLAHLHRIGNNLVRQRALDEIQRLLDDDSRTVSATAAAHLQSLLPPTPEPTPPAASVTKQAVEGQAAPRGSRPVTSEAQPPAAAALTSKKQSGISGADGRSALVVPVAAPEPVPWQRVTVGVPGPEFEARPPGQYSFDFPDTECDGWSTPALALRYASVRGNAHRYFRQPRQDAARAAVHEPTGSIVFAVADGESGAKEPALGAVEACRASVEKMLHQLSQDQEQLDLLGVVGHAAERLWELTKWRLGVKEPERSDVAQFYATALVAGVVRPHPAGPVVEVCRIGDSEAWVLDSSSGQYQRLFGSKTPSETARLPDVPDPLKHTSTRLTSHHALLIGTGGFADVLGDDGQGRVGVLFAKHLAAPPPRLWLGHLLDFSRETFDDDRTLLAIWPRQQSHDDRPGA

>tr|A0A917TLJ6|OS=Dactylosporangium sucinum

MAEGPAGDSGLLAVVTVGVLAILVMTVLYFLQRRRSHNVQRVPAGAEVSTGSAGKAQPRRTTGAPQPPPRTGAAAPARSSRPARTSKFQGLRQVMPVRLTRGGRPATEPEFVDPVVVHGSWCDSVFHATHEAGGTRFAIRAATVRGKEHAYGGEPGQDAVGVVWSASRGALLLVAADGLGSLRDSGAVALTAAELALDHGARLGPGDPLSEVFRRVSAGLYREIERRGLDGATTLVLAELRPTRDGVLVTTCGVGDSEAWAMQPGKWKALHHERVRDSENITRHLPKHQPAAGRDPIAVRRGTVVVVASDGFAGALGGEGSPLTRELDKHWQNPPPAVDFLAQVGFQDDYFNDDRSAVAVWIQ

>tr|A0A917Z698|OS=Nonomuraea cavernae

MLRVVDITQSPGSTSEPTADRVGFSGNIAWVIDGATDFTDERTLPDISNVQWLVNLLDKTLLEIGASNKSTDLNLIFERLGEDTRAALAAIGSQGLRNHPCCSIGLAVFSDRVVRLGRIGDATLIAYQGEQVIGDVSTDFFEHRETQAVRQSQEGQQTEAEIIEAMFARRLEYIRGLHRESVFSGHPEAVFRIHTETFPLDSVDTVLICTDGFARCIDDYKIIKSWADLKVDVHRHGLKEIASRIRKFEAQGQSNPSPTKFKQSDDLAAIILAR

>tr|A0A918E0A1|OS=Wenjunlia tyrosinilytica

MSQQGDPHHGEDEWWQQLYGAAQRDTVPEPGQESVDKHFDAVRTVMDGGRTTDGDDHRGDDLDDLPDDGLHDGGVPDDRSPDDGLQDDGLQDDDFQDGDLREDGFPDTPPGPVPEQRTDPYRAPATLRLRTVSAPQGQDPGRGRDAGQAHDPGRGQASGPAAGKRPPPAYVGDRPPTYAPEPTAWPAADPEDMESLTPDTVVDGAQYGPLTLRAASVRGDSARYRGQPRRDAMLTVRFGEGDSALLLLAVACGERTGEDSHLAAREACRWIASAVGRHSDRLAEDIRAARRGSLKSGLQRLTDRCLGQLRLRARELGFEPEEYTAGLRCLLLPADRQCRTRVFFGVGDGGLFRLRDGEWQDLDPVRDPRAEETGGEVHRSFRFRASVGRPGDALLLCTQGLASVLRAEPDLVGLLAERWSGGTAPGLVDFLRDTQIRAKGYADDRTSAAAWEA

>tr|A0A918FKZ1|OS=Streptomyces aurantiogriseus

MVAGASVSGSVTGMPAPVTVAGTPTPGSVTGTSVPGQSIGGAPMGGSQMSGAPLPGSGFGAAPVPGVDLTGTPVPGSPASSAPLPGQGVRSAPLPGADMSGAPAPGSPFSGTPTPSADASGAHAPGPGFTGTPVPRADMSGAPAPGSPFSGTPTPSADASGAHTPGPGFTGTPVPGADGSGAHTPGPGFTNAPTPGADASGTPATGPGFGGAPVPGPGVGGGHLPPAPAAAHLPPGSPEPPGGGPVPPGAPVPPPPPGAPASGVRFDRPAEPEEYPLQAPDPRVAAEAAAVAETAKVCVACRAGRVDDDGYCENCGHAQPRERDHMEQESGPVAAVSDRGLRHHRNEDAFGIGHTALPDGSPALVAIVCDGVSSATRPDEASLAASRAASQSLLTALPQGTHPQQAMHEAIVAASHAVNALAGEPATAREHAPHQNAPACTLVGAVVTAGLLVVGWVGDSRVYWVPADRGSAPARLTEDDSWAAQMVAAGLMNEAEAYADDRAHAITGWLGADAYELEPHTASFKPDRPGVVVVCTDGLWNYAEAAEEMAEALPLDAAVRPLHGARVLVGHALDGGGHDNVTVAVLPFPAPAQGAGSA

>tr|A0A918HHK5|OS=Streptomyces purpureus

MMSQMDQLSARSTCPNCAEPVESGDLFCGACGCDLSAVAPAAPDRPTLAIGTPVPPPAMPGWPAAEERASGEPSAQSAGDLPGTDSGGRDLQAPVRHDTPAGAADGDYPLAAPDPRTAEHSPAVPPGAKLCVACRAGRVDGDGYCENCGHAQPRERDHIEEELGQVAAVSDRGLRHHRNEDAFAVSSTALPDGSPAVVAVVCDGVSSATRPDEASAAAASAANESLLAALPLGTHPQQAMHDAILAAGEAVNALAEESGAREHDPHQNSPACTIVASVVAGGLLIVGWIGDSRAYWVPDDRSAPPARLTEDDSWAAQMVATGLMSEAEAYADERAHAITGWLGADAYELDPHTAAFKPDRPGVVVVCTDGLWNYAEAPEEMARVLAPDAAVRPLGAAQVLVGHALDGGGHDNVTVALLPFAVPPQGAGSPYRTS

>tr|A0A918J5J4|OS=Streptomyces lucensis JCM 4490

MLKLANLLAMALTVPVLAVVILVLRELRLTFVRRHQHQHQHQAPADRGAPEKKSQEAEPGKRSAFETPGHGVPPRLREPSRTLPALGPTAAGTGEPPEEKRTPEEEQRPSEPLVTPRKSLPALPPETRTEARPAGTSRELVPLPGTVPQRQREDGAGVWLHTGAATTAGRHRRRNEDSHFADTDLLVVADGVGGAPAGDVASKLAVDTVVQAWRRGARRTHHHLQGGFRDANAAVLGHVKGNPQLRGMATTLDACAVIGDRIVGAHVGDGYVWAVPPERTSVRQITRPHAAPQGPLLRMIGSSVPVHPDLWAVDARPDTRLVLSSDGLPADLDERELHRLILSTADMAPAETASVLMKAALKAGGTDNITVIVADVVLSGSIEQPLLLSE

>tr|A0A918K767|OS=Streptomyces minutiscleroticus

MHRSTALSKCPSCEEPLETGDRFCGACGYDLSAVPPPPADPPTNAVNGWAPQPSPQTARSDGPAAVPGTQGGAPSAPVPAGAPGAGPDGFGLPQDGVRFDRPAGSAESVEPVGPTGSAEPTGSAGSAEPDDYPLAAPQPPAAPPVVVPPRPEQPPADPRTAEPPTPPAGTKLCVACRAGRVDTDGYCENCGHAQPRERDHMEQELGAVAAVSDRGLRHHRNEDAFAVSSTALPDGSPAVVAVVCDGVSSATRPDEASLAAAATANEALLAALPLGTHPQQAMHDAIVAAAHAVTALADGPGQDDEHAPHQNAPACTIVGAVVTPALLVVGWIGDSRAYWVPVDRSAPPARLTEDDSWAAQMVAAGLMNEAEAYADDRAHAITGWLGADAYELEPHTAAFKPDRPGAVVVCTDGLWNYAEAAEEMAAAVPLDAAERPLHAARVLVGHALDGGGHDNVTVAVLPFPAPPQGAGSA

>tr|A0A918QM77|OS=Streptomyces inusitatus

MSQQAEDDWWDRLYEASATDGRGGPDGGRGIRKDEPAGPGGEPGDTLDSRFATASEAVAKAPGASPPARREPDPADLPAISGTPVAPPPGAGAERRAAGRAGLPDQGGPTAPDGVRPRPRAPWEPPLDPTPPAPAPEPAPAAEPVELPSAVSYPLFSGLGQPVPASPPPPPASPHPAAAAGHPGHVGDGPPTYRPEPDALPAATAENLGHLVPDTVLDGATYGSSTLRAASVRGDSARYRGETRREALLTARFGSGPAALVLVAVAAGERSAAGGHLAAADLCRRIGGAVARGHARLSDDIRAGRHGDLESGLRRLTDHGYGRLRAAAAERGMAPGAYTAGLRCLLLPADPGCRTRVFFGAGPGGFFRLRDGVWQDIEPAPPEPADEPGAPGRATGPEPFRFRAPVARPGDTLLLCGPGLADPLRSEPALAAELATRWAGPGEPPGLAGFLADAQLRVKGYADDRTAAAVWET

>tr|A0A918VLB5|OS=Arenicella chitinivorans

MPWNCVVDTEIGDRDEQQDRFLMAQSPTGERCLLVVADGAGGHKTGGAAAEAAIEYIHAQLTNLLNSQNPQTALHELIQDCNERVLNVGGDELACTTLVLVLTHEDQLFWGHVGDSRAYLIRDGETVLRTTDHSMVELQRSDPTAVPETEDAVTPNQLYMCLGALDDVLPDTDSSVAREGDTLLLCSDGLWSQVDMQSVTRALSSAPMTTELLRKWAGLAKAGGAGQSDNITLIAARYYTKPSLLERLFSAIRKPVDKLRN

>tr|A0A919C8S6|OS=Streptomyces finlayi

MSQQGERRTGAEDDWWDRLYDEAAPDTGPTPSGHTVDDHFDSTPGAGPARDAGVREPPRPVPKEPRTRAPWEPPEPPEPPSVPPSANSPADSPGDVFPRDASPDVPPDVPPVVRPDASPDASPDALPDPRSARPAERGWDAWLPTPRRSRRVGGTEEGPRDAVPRPSPTQPEPQPEPAPDTGPEVSGPAPAPLPADWRPGTPDTGPRTPAPGPTADSGPTADPTRGQAAAPGPTSEQAPAAGPPPAPGPSAPSSPPGAAPDSPRRDADGPWSGSETSTWGPPPPLPGPPGSPGPRHDLRRTRRLWDEPSPDAYGPRPEPPAPDAARDDTPAADPRRAASRTEGRLRDVPPLPTSYPPRQDAPQQPPTVPPQPRRAPADPGVFGPPPTPADDKPDTVTDTDTSTTPHPETHPNTLRTETRPDVPTPTPAPTAPWDASVSAGPHRARTVVTHLGDGPPTYDAEPTALAAADPQDLGALVADTVLDGARYGSYTLRAASVRGDSARFRGEPRRDALLTARFGSGDGGLVLVAVASGARAAEGAHLAAAEACQWIAGAVGRSHARLAEDIRAGRRGDLKSGLHRLTDRSYGKLRARAAELGLEPGEYTADLRCLLLSADPLCRTRIYFGVGEGGLFRLRDGVWQDIEPPEPVPEAVTGEAVVGFGSAPSADSEPEEGPDGDRLTMDLGITTPPSPYVETAPPSSRFRFRASVARTEDVLLLCSAGLAEPLRGEAALAKELAGRWGDPRPPGLAAYLADVQLRVKGYADDRTAVAVWEA

>tr|A0A919DPV1|OS=Streptomyces longispororuber

MHQPAALSTCPSCEWPLESGDLYCGACGYDLSAAPEPPRDHPTLAVNGRPGDAAAPPAAPADWPTAPGAAAEPDNGLGTVETQDAGDVRVRDSADERNGQHVHHQNQSHSQNQRPGQGQGQDGRLGHAGPSSPSVPSSPSGQTGPTGPTGQGLDGRQGLDGVRGVGDGRDVPGVRDGLDSRDGRDGRGGRDGWDGPAGAGPDDYPLPAPDPRATAPAAAPAPAKVCVACRAGSVAADGYCENCGHAQPRERDHMEQELDAVAAVSDRGLRHHRNEDAFAISSAALADGTPAAVAIVCDGVSSATRPDEASLAASRAASASLEAALVRGTHPQQAMHDAILAAGEAVNALADEPVPAREHHPHQNAPACTLVGAVVTGGLLVVGWVGDSRVYWVPVDRTGPAVRLTEDDSWAAQMVAAGLMSEAEAYADERAHAITGWLGADAYELEPHTASFKPDRAGVVVVCTDGLWNYAEAAEEMAAAVPADAAERPLHSAQVLVGHALDGGGHDNVTVAVLPFPAAPRGAGSA

>tr|A0A919SWD8|OS=Actinoplanes consettensis

MVENPFMAVVVATVLGVTALALVLFGTGHLTLPPVEKIPRLWHRPDRHRDGIEPPPDMPVKEEKPTEAQPQVARLVIDPPRDGQEREPRLPARVVTGSAWHGSFFHGWCGTTGGARPAVLLNVRAATLRGATHAGMGTEGQDAIGAAWDETHSALYVAVADGLGSLPASGRVAAEAITAALHLCTTRPDSLSFAAGATRMFQAIGAGLVRSLHEGESGACTLVVAEVVPRFDGAEVTVVGVGDSEAWALFDSRWTVLHHERGGSENATRDMPTHAADPRVQSFMLSPGSVLLLGTDGFTGALDVSVSPLARALARLWRQRPGWLDFVNHVGFVDEYWSDDRSAVAVWIGEGAVDG

>tr|A0A921ITD8|OS=Collinsella ihumii

MDVCRGTRLEVVASACSERGSRALNEDSFMIAGDLMCVSDGIGGAPHGDIVSRVCCGALADEWRREPADEDGMVRAFCAADDLVSRVSGYLGKGSGATLVAAARCGEKMVFGCVGDSAAWLLPPAGGLVRVFEASGRLREAGSALDAAMGYRMLQGGGRACVRVATAPMLPGMKVLLCTDGVWSQLPHRHIGFILSNHDDPYAAAYRIVREAVSAGGEAGDNATALVACAREVRADRDQQTPFSFEDALTERSAG

>tr|A0A921SYV5|OS=Romboutsia timonensis

MKFNIFEKSVIGYKNLVKGYQSQDYIDYKEDDKYIICSVADGHSTDFFKYSLYGAKFACKASIDILSENFDMDIEVLREKLINYEIQKQIDSRWRLLVEDHYKKNHPNVFKIEYIKYSTTLLSVLITDKFILYLKLGDGDIVLKSKDEYKYVINTRNNLTVDSLGRNDEYKNIMYSLQKIDEKENINIVLFTDGYSNAFKNKFD

>tr|A0A923CT09|OS=Desulfobacterium sp

MFNEKVSLKNISYHQNKILWAVETNSGLIRSHNEDYYIARPAFGLWILCDGMGGHAGGAMASRVCAETIAHYIIQGKDLEQAIQLSHEAVHNLGKQASDQSGQPCSTVAVLLISKRQWTVAWAGDTRVWLFEKKKLQQITRDHTVVRKLLDWGVITEAEAKIHPDRHKVTRAIGIGKEHPEISIKTGVWNSDQVFLLGTDGMAYRDEPEILLNILINTVNPDQVVDELAYASLQISGSDNFTIAVVGRSLENRFIRNAVEKSREIWNRHSKMGGLLQPVHF

>tr|A0A923LPZ3|OS=Roseburia zhanii

MKAKSFNLSAQGASHIKKNKECQDASVSYFDEEIAIAVVCDGHGGDDYMRSAVGSKLAAAVAEKNIRNFLKAMTKDQFFNDTEKVLKNLEASIINGWNEAIYMHFTANPFSQEELVGVSEKAKKRYVQDGRIESAYGTTMIAVAMTHEFWFGIHIGDGKCVAVNPEGKFVQPIPWDPKCFLNATTSICDSDALERFRHFYSDKLPIAVFAGSDGIDDCFSNNDQLYNLYKTIIYSFGTTDFEEAVNGLREYLPRLSAKGSGDDVSIAAILDLDLLPELDIVKEFDREKEKARIEENARKEVEKNEAEKRRVEEEHAKFQRANQQKTAQPSKYRHTCKFCTECGARLAYGVKFCSECGTRVAPKIDKPEQENDGIKLIDICQQHMGKKEDETEECEKYSTNGIEDSNDQIEKNAECEIILTEENSTVQIENEYIAFEE

>tr|A0A923TKT7|OS=Burkholderiaceae bacterium

MAKGYRLTASTGIHKGDRDYQQDQVALLNHARQPGCVLGVVADGMGGRSGGRKASDQVMMTVKQLFERYSPESDDAPSMLKQMVQEAHIVIKLTAISAEQEPHSTFAAFLINPAGDCHWVHAGDSRIYHFHGNKLVKRSMDHSYVQTLVDRGEITEEEANIHPQSNILMGCLGTEDDPPIDVHFIPQLRPGDVLMACSDGVWHYFSPNELGSVLSSLSPREATEFLIEKARSRGRGGGDNLSLVIVKLEPLGPDQPVPIALSSLPPLGR

>tr|A0A923W7E9|OS=Polaromonas sp

MKFSVFQLSRIGGRAMNEDRMGYCYTRESGLFVLADGMGGHPQGEVAAQIALDTICACYQQAAQPVLADPAEFLANAMLAAHRQIVRYASEKGMLDTPRTTLVAAVVQDARATWLHCGDSRLYLVRDGQLLLRTRDHSFLEQDRPGAAQGELLNRNILFTCLGSPIRPTFDTAGPFALQQGDKLMLCSDGLWGSLDEAEIVRQLSAEGVCESVPALVERALLAGGRHCDNVTVLALEWQTPEVFATTRSLAAGSAADGAFASTLQGDLLGSQADDLDDAMIERSIAEINAAIRRSAAKKA

>tr|A0A923XVA9|OS=Candidatus Sericytochromatia bacterium

MKKCQNCSNDNSLETNFCIYCGNSFIMQCSPCDFSNGFCVECGKKEISFSLTQTVDEKLACISDIGRRHKTNQDYAGVKTFSDGTTLLVVADGVSTAFNSEEAAKLAVSTVMQYVEGKPTTTETLAQAIYAAHDKIIKMTYQEIPELVEPMCTIVVTAVKDNKVFVSWVGDSRAYVFSDKKAQLLTTDDSWVSDAVKHGLSEEEALKSIHSHEITQCLGTRDEEPLANVLEHVVKPGDTIMLCSDGLWNYFKNPQDMQKYVSHDMQETCKKFVLYANSSGGHDNISVACFLVNSQ

>tr|A0A924C995|OS=Herminiimonas sp

MISSSLHPPIALTVAQKSGIGARSTNQDYLAWTRRNELACFVVADGAGGHEGGEIASRVVAQAIVDCFKKASSCTSALLDSCIELATASLGRQQAADRRFKDMTTTVALLFIDLQKRRAIWTHLGDTRIYLFRRNRIISITRDHSLVQQFVDAGYCKPDQLRTHPQRSRLYAAIGADNDTVCVAPHGSTDIEDGDTFLVCTDGFWEWVTEHNMEVTLSVATTAHDWLAAMMRLVETPGEQSKNPQDNFSAFAIWIGEPNEVTVRR

>tr|A0A924DVI3|OS=Candidatus Sericytochromatia bacterium

MQNLFAINSTSECGLRPVNEDAVWSDAVAGWVAVIDGIGGPGKGDLAGGFVCASLARAVAQEGPIADRVEDALREANQAMLARPLSNPTLKGLGASVTVVVLDGSKLLVWHAGDTRLYAVRQGKSQLLTEDHNLAAFLVKQRQITPEQATTHPGRHTLLRSLGKDANLKLDTTEFQVQTGDRFVLASDGCYVDCPEADWDEQMRARMRLCDAEALVRGALDNGSQDNATAIVVDMVEPLADDRRRAANYFPKIERLEVFLEFSNEAIKCVTVAHLYELLLQYTLRISNTERALVLTQADNGQLTAITGFAFTAGAQTLNGTGFQLLGPTGNFAQQRARVTVPAGLSGAAHVQ

>tr|A0A924DWZ3|OS=Candidatus Sericytochromatia bacterium

MTVAAVGPKYHVHSGFATAAGLRAANEDSLVALDARGMFAVCDGIGGPDRGDVGSGLVTECLTEMVADGSAEAAAVEAALAKANRKIIATFLADPTKKGMGSTATVALLAADRLRFWHIGDARLYRQRGAQLELLTEDHNLAWQLLKLKQITMAQYATHPGRNTLMRCLGKEGTPKLDFHEVTLEAGDRLALMSDGVYQPLGDERITALMGDGNDPTAIATALTQAALTAGSTDNVTAVVILVAGGPHDGDLPAGAPAAPSTELPALWHSPDLTTAVATLRQRFRETVHELAVVIRRHGEASQAGSDDATVAVLAEQVAAAPAIAGWTEAGRWMALPLDPAEPAAGILAVVPLAAPGPDVATWQAELRAWGSQLQHVAEVAHLRQIADNAVHLSSLAREMATADSNQAAMRQLLMRCLLATGAERGAILVSGDMRPIVSLDAAGTAATVEVDRPFVKGVMSQRRGSLQVGGTEDTGASVIGFSLITAICAPIVAAGDLMGVVYLSANAVIAQLTLAKLRLVEDLVLQGAPVVQMAEMLAMASDRHRHLADSVHRLAHLSGDADWIDHLQARTLDASLSHALSLGASGILTLDQGPSLSGSIHLVQGRIVSVQASLRGLTGLDALGFLLSWDLPTLTWTAGAAATTPPPAGGGVTMLIQALEQAGPWRQAVRTAPWDAVPVRLDPSKTDSDYRVELAEVIACVDNQRTVRDLLDQTSIPPQEFLVALAHLSDSADIYFERQGGQ

>tr|A0A924F2F4|OS=Gemmatimonadaceae bacterium

MDEPGAVLAPDDAVVPAAWVAREPGSAWCPDAIAALLGDEFVRAAAADPCVIAQGDEVWQVRGASVRGKSHAHRGEHRDDALAVHAADGLLLLCVADGAGSSALSRVGAAVTARLVVERTAARHAAGSPDDAASSRLGTAMAHAVHDAAVRLHELAAAAGVSPTALRTTVILVAICGDLILVSQVGDGAVLMQRHDGSVLRVGAMRETAWAGEVTCFVPDACAMTQAAELRQASAGDMSLIALMTDGIDDPFHPLEQSGAALVEQWRHGTMEPIGTATQAIGG

>tr|A0A924IMP2|OS=Candidatus Sericytochromatia bacterium

MSVALPLGWGADQWSLRSTAGESGANQDGIWTDAQTGWAVVIDGGGPEGSGAKATQVLLRFFATRLGQIDRSDDRVVRTLIGQANQALLAAVPGQGVSATLLVARFLPDRAQFWHVGDVRAYLWRAGKLDQITADHCLATLLIARGQLSREQARQHQASKSLLRYLGRPQDPEPDYTEVMLEAADRVVIVSDGVHTSLGDDLLAEAVGTAHLSTALDRLWQTTVTDDRSALIVEVPAQAAKPEHRDAMFDRLFQIITQTTDPENLLEQILVLAVEISHGDRGYIFLVDEDGGLDCRVRWGSPMPVDGESVSRSIIQKAILEGKALWIADAQHDEAFKQQASIVALNLHSALCVPLKVPKGDTEEVAGVLYVDRTSPMEILDDLRLLELLANYAGVIIQNGALYASARVQNERLRILNSLSRSIGNVSDIEAIMKDILANALRVSGADEALLLLGPHLTFGSGLTRSGEPATASHLSKSVLKHVTTELKSLCVLDAAIDEQWAHQASVRGMDLRTVMCVPLIENAALSGAIYVSGRAAVNGFSKGDLEFLEALAAHASVAMANARMLKQQQQQIDQMEYVLRLYQQAQEQAITDELTRVHNRAYLDDQFRKHFEAARRYVEPMALVMLDLDHFKRVNDTLGHQAGDEVLRRVGAILLDVCRSSDTVGRYGGEEFLVVMPHSGLEAAMGIAESLWQRVRAEPPDGGLPVTVSIGVAEIGTSTSLEGFLKRVDDALYQAKAAGRDQIVVAGDAPAGRAATP

>tr|A0A924MCX5|OS=Chitinophagaceae bacterium

MTVNTFSLNKIGGRKNVEDAIMPKQFNPQDPKLFIVCDGVGGSSFGEVASDLAVISFYETFKKQPIAKEADFEKILHQSLAFFRQKVNEFVATNPEAANTSTTLTLLTLQNDKAYVAWCGDSRIFQLRNGKPIYKSKDHSLVAELTAQGVITEAEAEKHPQRNIITRSLNSQTKPTDIEYAVLTDIQTNDWFLLCTDGLMEQFTENLFSVVLNKYDSKTKYDDIIENICFGKTKDNYSMYLVHITAAAATSNFKKIILPLIVVILAVGGWLAYSKFLKPTTQPVNFKVADTIKLSIPKKDYVDKDTVKSIMIKTHDSDST

>tr|A0A924PSG1|OS=Aquabacterium sp

MELEIAIISKQGGRSYNEDACGHWNSDRHLCCVMADGAGGHGGGDIASKLAVQNVLNGFALLPATTSPQLRELVVGTNHVILDHRIDGTVQANMHTTVALLAIDFIDQTALWAHVGDSRVYWFRQGRIQTRTRDHSLVQALADGGLIKPEEMRTHPKRSELQSALGTPAEYLEVGVPEQAVAIEDGDVFLLCTDGLWEYVDDEVLAQSLIDAPSPQAWLDVLEATVLTNAAHKPSHDNFSALTVWTSATPA

>tr|A0A924Q7K7|OS=Candidatus Saccharibacteria bacterium

MENQPTTPDIDPVAIAQRLSIDHATKAQEDAVRGRVDVTNPVAIEEYSLLHDKLGLMEGAGYEPLSFLNPSYPNEADSQKLPSPSEILTAKQQAAENKDRTKQLDLRDRPRNIDVLADSLKAHPQTRRNLVELRNKAVELSDLDSLFAIDNALIDKLALSKSTPDVQQAAIEKLNRLFGAKKQQEARTEVKVALAEVEPGSVKISSKSESNPSESVDSPLVASENSVPTVVVNNVAVTAETSATDIHGVVPIAAVEEQPTTEIAETSVNEAPSETSEDSSSEALEIIPADSEQLKGFKFDVGVSEKALKGEDFIVCDDSLGLFAVIDGMGGHGGGDVAAQTIGKALSEYFEKEAEQSTIDENNAVKVMKEAFLRAQADVASAAFNGLGNYDMGAVATAVKAFKGKGGKNLAVYGHVGDTRLYLLDTDNVITQVTKDEGHLHKVFNSISADREVKTDQFGIIEIPVGSRLLLISDGISGDRGSDIMSLDEIRGGLIQGDPQEAAEALLEASRKEDDKSVLVIDIERTDSPDKNDEDNISEESAKDTSKTAKRIGKLLSNLRRGRSAQMSAQTELSGIDPKEVFEIPSKEDDSENGEDAISMLKKYAALHGASEAIKIMKPEDTKESIEPRTADTPEAKAPSITSTRIRRAGQMGVAHTVIFRSNQTAPPIEELEAKKAA

>tr|A0A924U839|OS=Pseudorhodobacter sp

MPGDRSAPNSKPDQDFGRTEGDLLCVAASLRGRSHAREGGFRDDDFCLSADRQGGWHIATVADGAGSAKFSRHGAHLATKTVLNQLPGLLADIVTPGLDQLLDPYLQGHPDAGPQIKAQLQYRSLVTVAFNAFKTIETEAAQRGVPTSAYSTTLLIAVARKIRDDLWFIASFGIGDGGIAVFDAQDGNLRPLSRPDSGDYAGQTRFCTNPNLPAMIRSNAACPLTCAKTLPPSP

>tr|A0A925B2T6|OS=Pyrinomonadaceae bacterium

NLDYGDDAAFIHRKTFILLINPDPKSLWKNIEPDADAPYQKSNTATEQISSDDFRIFAASRRGRSHAHDGKFREDDFALKILENGWHLIIVADGAGSAKFSREGSRIAATTGIEFLTEAINNLIEPKFADLVKSLDAKNANADKEIRNLLYQTLCGAAHRAYKNIEREANTSQSVIKDFATTFLLTIVKKFNEKTFVASFGIGDGAICVYDERGGKSDLMNTPDGGEFSGQTRFLTMRELVSDGEEMLRRIKYNVYDDFTTLFMMTDGVSDPKFATDKNLLDAEKWREFGDELAKTIDFTESEKPVSQALLEWTDFWSQGEHDDRTIAFLKPKSKVQSRSS

>tr|A0A925BIQ8|OS=Anaerolineae bacterium

MSASGHEIRLRASARTSKGQVRENNEDNIHLWMVDNLLLAVVADGMGGAAAGEEASRIAVESIEEGMAIREGGHREEYEKMADTLVSKKLKEAIRRANHSIVKRASDNPKFKGMGTTVTLAFVRDTHVIVAHVGDSRAYLVNSQNAAISQITADHSFVEALVAAGHITAEQAEEHPMRNVLYRALGQTDDIEVDVYQERLRVGDRVVLCSDGLTRHVKPKEIMQFVLADDNPESASQRLIDLANARGGEDNVSVIIISAEKYVVTGTLDSKPSKFYDGPTQPVPDDAPSYPIDQDGYSEDSAPTPTDSTEASEDTILLREKVITPPPAPTDTNPNTETLVSRPKSGISRSGRDTLSEANREANRKGEREDAPENVRDTGSMEYVMVDPATSTAADVPTLETRIAPTELTGDDTVELRPRRGLPQAQEAMSEPNLPDNAEAAETGDTLPAATRTDRLPLLDADEADETETAAPRKEDDGEGRDTSRPEQ

>tr|A0A925FAR1|OS=Cytophagaceae bacterium

MLSYSFQLAERIGGRAEQQDAAGALVTRYGLLVLVCDGMGGARGGSTASRMAVDLILRGVQQSVEPNGATALLNAIREANRELFLRSQHDASLRGMGTTVTALLLQETHATLAHAGDSRIYQLRKDKVVFRTTDHSKVFELVKRGILNEEQARLSEDANVILRALGIKSDVEVELHDPQPYLPGDRFLLCTDGVSGAVPEELFLGWLASDNPVDSLAAQLVDRVDEYGFRQGGDHDNLTAALVECGTPAGSTIRKSVASRWPELIRIVPGLLLALALAYIAYERLVAAPRQATERRTLVAETKKLKQLRDALTTERDSLRRLLNDPAVRQAVRRKKAETPTGKPELSPPPKGLKTPSTLAPADSGTKSRRGTRPGTLLSPAPIPTDPEL

>tr|A0A925G6E0|OS=Pyrinomonadaceae bacterium

MLPEAKESHRSKDWAIITGQIGDDPAQMEKLIAALADPLIDVIRWGIKRPPELKANHSLQARWTHLQEYIQKQGELPKMMAHKAATFYEGINNAEPHKAAPSPVNQPAARPLAETPQPPAKAEAEPPPPPSPKPPEPLAITRQRETTSAVPDDSSEVTVVRAQNELFDETTIIAPVTHTETSINGETMKEESEIETSATPSIEASTTPSLAPQWKYLPVPDDPDKHTEYDSHAGVSPEGLKLIGARARGKKHKHEGTNCDDWFKFTASGPWTIIAVSDGAGSKAFSRVGAKVACNFAVKRLVEDLREHQLKSREVWTAETLKRDETNGSFVEEDLEAVQIALHNAMVMAYGALVAAAEDRAESSEHEKVLGRKVEVDDLSATLLLAVHTTVKYKEGDRNFVLACQIGDGMLGAIDQKGRLQLLGEPDSGEFAGQTDFLTSRNKVDKDNLTRKTRGFFSPLQSLMVMTDGVADDYYPNDPDLLRLYGDLALNQIIEIKGPSDKDVAARLELTKLPTLDQVMQADFYSDVETNTAEGIRKTRLKSVAAYAEKLDLPLAEVVASPALLLAGARGEPLCDGEAQDRLRVWLDSYHVRGSFDDRTLVVLHRETVS

>tr|A0A925GBQ8|OS=Rhodoferax sp

MKFSVFQISRKGGREKNEDRMGYCYTKGSGLFLLADGMGGHPEGEVAAQIALQTVSALYQKEARPEIEDVTAFFKMAVLAAHRQILKYAAEKGLPDTPRTTLVVAVVQGGAATWVHCGDSRLYMVRQGELLARTRDHSYSEQQQAARPHAPLPEGFNRNVLYTCLGSPTKPVFDVTGPVPLQQGDRIMLCSDGLWGSLDDADIVYHLAQKPVASAAPDLVERALLKAGSHSDNVTMIAMEWETPDTFVSTRGSITTDSIDEGVFASTVQAGWLDSTVDDLDDAAIERSIAEINAAIRRSAAKKF

>tr|A0A925H0R8|OS=Pyrinomonadaceae bacterium

MKLPSLLRGLRRERGGDRQQATTPPPKQEPATTVEAERAPRLEKADAESNGTNENRGTMKKAVKATGALNAKAAYETIASVQTDVGCVREINEDSGTFVSPSDPAVLKLKGILLIVADGMGGHSAGEVASGMAVELIPRLYYEAKGDPQVALKGAVEEANRQINAAAAADTAKHGMGTTCTALAILDGQAFAAHVGDSRLYMQRDGKIYRLTEDHSAVMEMVKLGLITLEESRTHEDKNVILRALGTAPAVEVATLVPFSVRVGDHYLLCSDGLYDLVPDDEIERELTEAEDIHAAGERLITLAKARGGHDNITIGILAIVPVGTEVAEAEGMRATREWRVQG

>tr|A0A1S6EUF4|OS=Brevundimonas sp. LM2

MADAKTIRIDPKDYRLDGRLAKRIWRLSKPYWTDPRHWKSWVLMVFTIAVGPAWAYVGYWTAQKNADQVNALVATDQSVFLSLFWLLFWLGVGQWVYQQLLSLLTQLMTMQWFRWMTEWMVNRYLEHKTYYDITLNEDIDNPDERIESNVKPFIDSMLSVPGRILGSVLGVATNAVLLTQVSSAMTGFVVIYSMITMGIQVLIYIPLIRMNFEQVAANADLRYGLLRVRDHAETIAFFRGERTESRQVVSRLWRLVNIQMKIFYYTLWTGGLSQALSYVWALAPLFLVYPLYFSGQIAYGTITLATAAASNLMGALTQLNQYLPFIANLAPVTVRLAQLVERWDMLAERNKGNTPGLITLREGREGLRLEGVTFTTPGGQSPLVKDLTFDMGAGRSLIVIGQTGVGKSSLLRVMAGLWRQGSGTIILPPEADTMFVPQRPYMMLGDLRAQLLYPHGDPKLSDAELQAVLEQVRLPDLIQKCGGLDAERDWIKVLSLGEQQRISFARVLISKPSFVVLDEATSALDIPTEAAVYQALGDSGCAYVSVGHRETILRFHERALRLMPQGAWELVDPQSLPLTEIAPLASARPARPDAVEAPVALTAGRAPATDLALAERQPLVTDLDTTAPLTVSRFAADLAGRRLDVAAAQIQGERDDQEDSVLIQPLPGDALLIAVSDGMGGHPDGEVASALGLATLRQAVLALSDQVSTAAGWSAALHAAVIQATTAVRLLGDASGLGMKAAKEDPGPKTPGATLAAVILVPSQDVWVYAAAGDSWIYKLNGQRGGKRINTLHTGARKHTITSFLGLKTPQVEGTDLVLPLKGVTGLVLATDGVDVLTLRDVEAAVRAASDAPDAAADILARVTAKAHPKQDNASVIVVRLSASA

>tr|A0A1S6RTJ7|OS=Streptomyces hygroscopicus

MLEGARYGTLTLRATSLRGDSARYRGEPRRDALLAVRFGIGDSALVLVAMASGQPAAPGAHRVARELCEWIAAAVGRNQARLTEDIHTANRGALSSGLHRLTGRAYGRLRAGATVRGLAPADHTASVRCLLLPAHPACRTRVFFGVGDGGLFRLRDGVWQDLEPAGGERDTVGGPVLGYGSGRPTAQQPPEQPQPPLPPQPPYTRADPSSAGPAAPSAPGPNPDPAHEPFRFRASVARPGDTLLVCSAGLAEPLRGEAALADRLAERWDTAEAPGLAAFLADAQTGVKGYADDRTAAAVWEA

>tr|A0A1S8C573|OS=Modestobacter sp. VKM Ac-2676

METVLVLDSAAVSTRGPRPDNQDSAVGGPLLVAIADGVGGNVGGAVASSLVATWLAPLAVGSTGEGSDDPVRVVASANERIRAAYTERPRLRTMATTLTAVHVDAEGLVLLHIGDSRGYLLSGGELNQVSTDHTLVQALIDAGSLTPAEARVHPQRSAVYAALHGADDDVAALDVIRLEAARRPGDGLLRRALRRRPPGTSSSGSSPPAPLPRPPRRCGTPRWPARRRTTSPWRRRRARDRRVPR

>tr|A0A1S8TC55|OS=Clostridium puniceum

MLKVNNYSNLNLILLLGVSLLILFLIKSFLLEKINKKDLEIAKEISIGYEEIQEDYGEVLTSPNGTLAVLADGLGKNEAGRISSITSVKTIIKMFKEEGSKERLLYFFKKAFNKANREIIKRVEKDKGGASVLSVIITNNLLNYALVGDVMLAIFRNKELVKLSEGHSISEIAKREYYNGKLEKAKALYAIKEKKLLYYVGQESFKDIEISEIPIELYKDDIVVLMTKGIYEGLKWVEFEKILNNKKVHVNEICEEIMISIANNNKSNCNGSIILIKYCGKKRKAAATNQNNY

>tr|A0A1S9CQY9|OS=Epulopiscium sp. Nele67-Bin004

MFVFNHTVVGNKREDKICQDYSDIYVDNSFYIGAIADGHGSKTCIRSDIGAKLVVECAIECFRQFATTFLAENITLNIPTTRHCRNIIQNLTNCIVASWYDAINRHYATDPIKGLPAECVPHLYGTTFMGALVVGDYLILLQQGDGRCVVFFENGDISQPIPWDRRCEYNSTTSMCDADVTTSIRHAIIKREDVVACFLGCDGVEDAYIDTYHDINNDTIKNHCIMGGVNVFYKHLLVKIFEEQTNFNNYFRHMLADFAISGLFSKTGSGDDVSVVGFVEKGKVFNNIQSYKKDVELYELEELFYWKNDDIRSKKRKLEVLEKRLETAKSDLNIVKLDVLKKRKSGLLKYRENFYIDVLQASKNKQQAQQTFDNYKSQYFDLEQQCEKLQQKIRNYE

>tr|A0A1T0CQV0|OS=Moraxella pluranimalium

MIYQIQAVTWRGGMAHQQDAVLIAPKVYQHKGLISHHYETKRFCVAVADGVSSSPYSALASRTLLKLAAAMYADSGAVDFAELQHQLNQALTDDRHEGASSTLVCAYVMDDEIVLKHLGDSRAYYYNGASWACLTTDHSFMNELKADGVVSHDEYASCYGALMGYFAVDRMADVSTVKMASYQTLGLRAGECLLLCSDGFSEVLDGRFLPMGNQVSLKDWLSDSINQIRQNKPKWQLDNISAVLVRCVNEIRGEDVQ

>tr|A0A1T2KSA6|OS=Solemya velesiana gill symbiont

MNLMSIEAVSRSDKGLVREINEDNLATLPEFGLVVLADGMGGHNSGEVASQVAVETMASYLMPVLASVGEAPLEEAVVAANDTIFATIEEESQFNGMATTVVLGVFGEQTLRYAHVGDSRLYCYSDGQLGLLTRDHSMIQELVNQGMFENTEEAREAGVQNNVLTRGLGVDQQVEVDIAEIEIKTGDLYLFCSDGLSNMVSDGVMEELLAATDEDIESTADKLLALALENGGLDNVSLVLVRPQFD

>tr|A0A1T3NZB1|OS=Embleya scabrispora

MPDGTSKRHTPGDGSPGRPVPRAQGPRRSGPGDRERPEPDWDRRPDPDYDRRADPDYDRRADPDYDRRPDPLYDREIEAPSGPPGFPDPAPPDQGGRRSGRARRVANWFGFGAPAEEDEPDQYDRRPGFGRRDPYEADAGVYDYGRDQGQDYDADYDPAPPASGRNRRRTGSSHWEDVMLGSSPPPGTPMSDSIPGDTMPPDRLHRDAQGVLPPPRGGARPGRREDPQQWRPEPDDDIEPADPMDPVDPLDPGPSPSAERRPNYPPIPKHRPAPTVESDGYSSIDASPALGDEPAERRRDVGGEPAPDDAGDAGTSFLKVPPAQRTPHRSAPADNPADEVPRRMLRMLPPPALADGGGMVVDGGRLGRLNVRAAGVRGELHQQQGLPRQNCFAVGPDPSARWTIAIICDGVDNAHASELAAQIAVRSAYRTVARMVARERHENWDWAGVQAEVEEDLRSRLTGTADRAHELGRSEPATRLAILVTAADPLEDDRVDMAVLGDSTVLRLAQGGWRAPLGIRDTSMEGAPAAIPDQAGDDQLRICRTTWRPTDVLVMMTGGFAEALGRPTAGLAPRLAEDWRTPPSLFDYLRDVDEGLRQREEDGCVVALWPSP

>tr|A0A1T4Q1D7|OS=Selenihalanaerobacter shriftii

MRKDNSELLTKFISKPGNFKKNRDYFAYAEHDDMACWVLADGLDSAPDVLSAEIVVGSILNDFTENPSMKKRHIKKYIRNANQALLHESRMMNLQSAVLVVITDYNSIIWGNVGNTRLYHLRKGRINLKSKDHSLAQMMVDAGELEDRQINHHQERNNLTDYLGQRDKIKPYVSKKHTLKDDDFLLMCTSGFWENLEDKEIGAAAKEATEAQGFIEELEGIMLNKDNDNLDNYSMVSVFAKKVFKEEKNSRKNLYKKAAVILIPIFMLTGGFIVYRKIRAASNLKRQKQLILRKIKQATTRSRSADKLARKGNSRQALAEYKKSKETYRKHGKKEKVAEIDKKMKRTETIILGEKIEKEADKQFDEAKYERALTKYEAAKLKYLKVDDYDLNNIEEKIAKTENILKAISYEEEGNMFFKSNQFSVSKEKYTAALDIYSKYELANKKERINQKIEKSQQLMNSNQRVVRARKVESSGDELFKMRDFKSATLKYTEAKVIYSELGMSRKVSKLEEKINSISSSKAYIEAKEYENQADVQLEKSKYTEALFGFKQANKIYSRIGKDRDCTRIEKKIEDSKKIIKAQKAEAEGDELLKMQNFENASLRYMEAKVIYAEMDMEDDMEMIQQKITNVSTAKLYKEAKEYEGLADMQFDNEKYKEALFNYKQANKMYGKVSKGKDYARVREKIQKTEEEKEKFLFFF

>tr|A0A1T4ZDC4|OS=Planktothrix sp. PCC 11201

MQWQAVGWKEQGIGHIKNGTPCQDDFEYQIISNKQVIIGAVSDGMGSAAHSELGSQIAVQTAIRLLNQNNWLNNPLNDNQARAFFEKLLKSVVTEIQAEANDKGYAVRDLACTLLAFVATPDWLAAMQVGDGLIVIRPQEEATYKLLFRPNKGEFTNETVPITSSIASQEMQVTVIPKAVDFICSATDGIERISLLKSQNWIAYHKFFEPLERQIMRPNITQTEKQNILRDFLNSERVNQKTDDDKTVLLCIQQEENRSNSVKTQSSNISKPVQKSAPSSSPSPHHKSPNQSQEATDDRDQALDQVEQRIYKLIRCLDPNLKIISEIKEQTLGIYLISQRPLNKGYSLILLILLNINDIKSIRVKKKVKVFNQNVSASQTYWSGEVSLVLLPFWIKKVLAIAVGAIVMTLISAFIKNIPNQFFVILFYFIYSLILIFIFDLFVNQKK

>tr|A0A1U7I6F9|OS=Phormidium ambiguum IAM M-71

MLICPQCQFENPVTNKFCQQCGTSLTHKACPECGTQVTYSTKECPQCGAFTGTVWLAIISPRTLTQIQENNETADAELRSASVFEKLNPEQPDIINETEPVKTEIQVSWEEELADHNPEKNSAPAISELSSDIYLDKQQRYQLLEPLPPKITEEVKVQVLDSQPLQASPLQVLINQQNQEWETETIPEIQSNIDIWQTMPDIVKAYLALKAEFDPILPNIHDAWSEEDCEIILLEDRSHWPSLVDLWKDHQIPQIHLLQIFHEMSSLWVAAESQKCRQSLLELHNLRVDEDQSLGIVRLYPDLPNSDLKLSELGQVWQSLFYETGRTLFGPVGTLINDLQLEIISTVQDLRSRLQEIADELQSESIPAVEDAIGIDTTYANPTNNNQSISPPPTTTPIIKEHMNPYSLINPEGSDDMPTVVLPMQLLSLEDAGRTDVGRQRRHNEDYFGVQTKLTKIENPLGRNMEARGLYILCDGMGGHAGGEIASALAVKTLIEYFHENWRDELPDEDSIRQGVLKANQAIYDINQQDARSGSGRMGTTLVMVLVQETQVAVAHVGDSRLYRLSRRQGLEQITVDHEVGQREIKRGVEPEIAYTRPDAYQLTQALGPRNEHFVDPDIEFFELNEDTLILLCSDGLSDNDLIETHWQTHLQPYLSSRSNLEESVNNLIELGNQYNGHDNITAILIRAKVRPDLDQARVNNS

>tr|A0A1U7J5W9|OS=Phormidium tenue NIES-30

MNGSFLHDKLQEYLAQWLNPEEGHRTFQVDGAEVAIGSAIGCVREANEDRVLVARLLRDKEPNRNFFLGILCDGMGGMSRGGVCASLAISSFIKSLISGKNSDFSKLVETAAFEANNAVYEKFNGAGGTTLSAVVFSANGEALSVNVGDSRIYRLLAKNKLDKLTVDDTLKGQLEEKNKTAQLSLPEFRSLLQYIGMGEGLEPHIFNISPINDIKPIILTSDGIHSMPPETLKQLITNSQSVGDIVFRLITHSEWIGGHDNATSLSIKGDLSSTMSSKGKEFLNVLEVWTSTKNLQIWITSPSRVLANSFTESSSDTKYRTRNERISTDESVKDSFVEDKIRKDEGEQKITKPRRSKKRLKAGESPAEADNISIVDIYIPEINVDVPDPSEGSGSE

>tr|A0A1U8A814|OS=Nelumbo nucifera

MADFVGRFLDHRSLKNLSCFRAPCISCNISPKPFGIFRISPITPFPKRRLAHQISAKPSSSTTQLSSSATPSPDSTSSGFIVISTTERSDGSIIFRFGKVEEAKTDVVSGESSSVAVGGVQIEGSTPVEKEDESVFSTLVEEEVELSEEKSESGTCVSEEEETQMTTDTLETLGTLNGDPVTETITEEETQMTTDTLETLGTLNGDPVRETITEEFTGTSHEQNVVALTEDRPEVAGSSTLPNSSLDVETSNSSKEDIEEKNAVEVFLPSKASMPDSVFDVETNAEAEEASKDETVILSTTTNTTQDPNNVLDAGRTDTTEDSEEKGNGEGKGPKEMHEDENPSPTAALDGGKVLRIKQSISSKEKTEDQTVLQLTASEDVGDVLYVEHNISSEEDSKEKNEDQTVLQLTASQPDSILPVGNSYVSKEDSEEKSEDENLPQLVATEPDSVLEAENTNAFKEEFKEKGNDEGIGSPSSAQFHQITDAETSHASEEDPKEMNMAYVTPFSVGLESMSNVSEDDSREKVIIEDVRSPLDSEPEPAYEAVGNASEENGAENIKVEVMSLSTAAELDRILDAKTSHPSEEDDVVGIMPVMPPLASNPDHVLDVETGSKTMEEFEEKDTVESSAMVEVIEGALQNEHGVEVAGTELVELQDVETTPDPHGGEEISAPELFLSSGAAMLPHPSKALTGGEDAFFVTGQNWLGVADGVGQWSFEGINAGLYAQELMENCAKIVSEYQGVQLTKPDKILIQSAAEAESPGSSTALVACFDGQAFHVANIGDSGFIIIRNGTIFKRSSAMVHEFNFPFQIERGDDPSELIEAYTIDLDEGDVIVTATDGLFDNLYEQEIASIVSKSLKASLKPKEMAEFLAMRAQEVGRSESARSPFADAAQAAGYTGYIGGKLDDVTVIVSLVHRKSESQV

>tr|A0A1U8AGV2|OS=Nelumbo nucifera

MADFVGRFLDHRSLKNLSCFRAPCISCNISPKPFGIFRISPITPFPKRRLAHQISAKPSSSTTQLSSSATPSPDSTSSGFIVISTTGHPSDRAFLLTSICFCVACCLFTPNLVLSAERSDGSIIFRFGKVEEAKTDVVSGESSSVAVGGVQIEGSTPVEKEDESVFSTLVEEEVELSEEKSESGTCVSEEEETQMTTDTLETLGTLNGDPVTETITEEETQMTTDTLETLGTLNGDPVRETITEEFTGTSHEQNVVALTEDRPEVAGSSTLPNSSLDVETSNSSKEDIEEKNAVEVFLPSKASMPDSVFDVETNAEAEEASKDETVILSTTTNTTQDPNNVLDAGRTDTTEDSEEKGNGEGKGPKEMHEDENPSPTAALDGGKVLRIKQSISSKEKTEDQTVLQLTASEDVGDVLYVEHNISSEEDSKEKNEDQTVLQLTASQPDSILPVGNSYVSKEDSEEKSEDENLPQLVATEPDSVLEAENTNAFKEEFKEKGNDEGIGSPSSAQFHQITDAETSHASEEDPKEMNMAYVTPFSVGLESMSNVSEDDSREKVIIEDVRSPLDSEPEPAYEAVGNASEENGAENIKVEVMSLSTAAELDRILDAKTSHPSEEDDVVGIMPVMPPLASNPDHVLDVETGSKTMEEFEEKDTVESSAMVEVIEGALQNEHGVEVAGTELVELQDVETTPDPHGGEEISAPELFLSSGAAMLPHPSKALTGGEDAFFVTGQNWLGVADGVGQWSFEGINAGLYAQELMENCAKIVSEYQGVQLTKPDKILIQSAAEAESPGSSTALVACFDGQAFHVANIGDSGFIIIRNGTIFKRSSAMVHEFNFPFQIERGDDPSELIEAYTIDLDEGDVIVTATDGLFDNLYEQEIASIVSKSLKASLKPKEMAEFLAMRAQEVGRSESARSPFADAAQAAGYTGYIGGKLDDVTVIVSLVHRKSESQV

>tr|A0A1U9VF65|OS=blood disease bacterium A2-HR MARDI

MRFSVYQESKKGARRVNQDRMGYCFTRDAMMMVLCDGLGGHSLGEVAAQQALQTMARQFQRHARPMIRNPHDFLHESIMLAHRDIHRYAETNGLPDAPRTTIVCALAQRGSLHWAHAGDSRMYLMRKGELVTRTRDHSKIENLLQQERVLPMQVANHPERNKIYNCLGSPNLPLIDIGGPVSLEPGDVALLCSDGLWGSVAEDELVDTCTSFSVTQALPELIARALRNAGDTADNTTAIAMMWELDAVTTTQDSVQTDTLPLNAFTTSILDAPQSESGLMSEEEIERSIAEIRAAIDKTTNLTR

>tr|A0A1V0AKZ4|OS=Nonomuraea sp. ATCC 55076

MDAEGYCERCGARQPTGRDHAEIVLDRSGAVAVTDRGLRRSRNEDAVALLETPNGAVVTVVCDGVGSSPRADEASAVAVETASAALARGVPHEEAFELAGRAVAKLATSLDDAPACTYIAAMAGPDGGVTIGWAGDTRAYLLPSGTPLTEDDAVETGEITAWLGADADDADPHVRTLPPGTTGLLLICSDGLWRYLDGYDFPTSGTPLELAREMLRHALSSGGQDNVTIALIPLGEAHG

>tr|A0A1V0DAE8|OS=Rhodothermaceae bacterium RA

MTPPSPRLRASGASDPGCVRSDNEDRVFADAAHGLFLVIDGVGGHAAGDLAADLTLAVLRDRLASAEGPAEARLRAAIAAANNAVFKRSQAAPSLAGMGCVLTIALVGERSVTVGHVGDTRLYEISADGLRKVTRDHSVVGRLEDDGLLDETAAMAHPRRHEILRDLGSAWRHGDDADFIDVYHLPFAEERALLLCSDGLTDLVPGAVLHATVLAHAGRPEAAVRALIAQARAAGGYDNITALVVEGPRFAEAVARASSTNRPGAMPHSFRHDPDAWEGLQRRMQERRWLLAASLILLMGLLLTYRAKTRTFDETEALLAAGDVVNLSTLTRSAPLVPVFAALYPDPRDQALAAEHTVEVLSRLRAQHRLPGWHLPNVGRLNTAAFAVPVEQAARGGHVFRSRLADAGSATTVPLLGSMTAFQRLKPRLVVRSPGRYRWTFWILAGLFFLGFYAVHADGFARRFRGTPSCSPCSIC

>tr|A0A1V0QX77|OS=Streptomyces sp. Sge12

MSQQGADDWWQKLYEGPDAGAEPDPGDTLDNRFRSAAGVTTEPAPNRGRPLGRAPDQDPAPTPGAPPTPGPDPAPSQNQIPAQSQAPDQARGPATGRGQVPSQGIDPGPPPSQAPDQGRAPGQGRGPASGQSEVPAEGQGRSTAQVPGHGFDPGPPPSQAPDQGLGQGRAPDQALRQGLGPASGQSQAADQVPGQGLGQGWAPGQGFGPASGQSEVPAEGQGRSTAQVPGHGFDPGPPPSQAPDQGLGQGRAPGEGRGPATGQSQVPAQGLGWGPAQGIDPSPPPSQAPDQGQAPSQAPDQGQGWALPQGPAGVGPAGGRGAASEPPLLPGPRQGDAAAPAKTPTLPTLPPPRDPRSGDATGYALGGVAVPPMPESELPVRRPESPPTEARFEPPAAAPPVPAAPPVPAAAPAPAGPAAARPEVSHLGDRAPTYAPEPGALPPADPAALDALTPDTVLEGARYGTYTLRAASLRGDSARYRGEARRDFLLTARFGSGDEALVLVVLAGGDRAAPGAAEAAAELCRTVAGAVGRSQERLAEDIRAGRRDALRSGLQRLTDRGYGRLRARAAELGLAETAYTAGLRGLLLPVDPQCRTRVCFGAGAGGLFRLRSGAWQDLEPDAQAEDTEGGFRFRASVARSGDTLLLCSGGLAEPMREEAALPAELAARWAEQEPPGLAAFLADTQLRLKGYADDRTAAAVWEA

>tr|A0A1V1P2F3|OS=Candidatus Magnetoglobus multicellularis str. Araruama

MKIDVQQLTDPGPRDENQDEMGWFTLQNGGQLFIVADGVGGAIGGKEAAKIAVATTNEIFQKYVGKKQISNILQTAINTANIRIYKKGHSGNSNFQGMTTIVILYIENKKAYLAHIGDSRIYHYRARNLILLTKDHSRVQQMVDDKLISEEIAFDHPEGNVITRALGREQTIEPDICKLNPFHILPGDRFMLCSDGLCGVLRKEILSKLFHTYENDPKHLCKEFIKRSLNEGGSDNITVQIISFEGTPPTPVNTISDSHLLQPAQSKPTESKTWFSKKNMFMLVACFLLIGLIVVVSLKIPDDKQTETGTTARQTVTNADKQENQDKKDNQDQQNAETKMPDPKKKMKKDDDPKKVVRNEGTKQTLNKINTRVHENKRVIEISINKNKQLTLEDLKKEISDLIAKYKIKKSFTDILQSNNLNQYITSKDKNLLNPSITIRIIVE

>tr|A0A1V3NW97|OS=Rhodanobacter sp. C06

MDQAIDFHSQPRVAAGRARGGRETQQDALACLHDAATDVHLLVLADGMGGDGAGELAAEGVVHVAHQLWAQGLWREQPGALFLETLCQEAHAELVRRREGLTRGEPHSTVVALLIRGNQAYWAHVGDSRLYRFQGRHCLNRTEDHSLAQLKLQRGEIAPEQLASDPDQHKLLRGLGGPQPPEVEHGGAILRVGQTFVLCSDGVWEQLSTPELRRLSRRRDQAEALREALQLATTRGGEQGDNAALILLRIAGKGWMRRYGERLWSMVHPTGATAHGSGAQAAQGDGKA

>tr|A0A1V5P8Q9|OS=bacterium ADurb.Bin363

MSNEIKCPRCGFISEGNFCVSCGTDLKSIIKKEEIDINEIEEKIIKIFNWLCHQSPQIQISRLCNKVASEFSDIDTATWEHIRIFLKGFKEGSRITKRYYRKVTIRKDETFKSLEDFFFKGPVEDEETKEFRVFRIASFITELTGLSETREEGSKEISEDFASVSDEALDSNLSTQDIILPEEKLEELKKEIPLPEGENIITVDKEKSFYEEDKPLPITSNWGYEPVPEGPDMHEEFDCRWQDITKKFKLIGARGRGKKHKHEATNCDDWFEFSKSNGWTIIAVSDGAGSHVYSRVGAKVSCQTAVDYLSKELKDHLIKEREDWSRETFKRDEQTGSFAEEDIEFVQKKLHEGIKKAYETVVAAALERENNPDYFNSLGRKLNLRDLSATLLVAVHTTVKYKDKDYSFGLTCQVGDGMTASVDTTGNLRLLGIPDSGEFSGETDFLTTKEKLEPENLMRKTFPYFWPIRALMIMTDGVADDYFPNDPGLLRLYGDLVLNGIIDIKGIDKEEIEKALSVTKLTTLEEVKKADYHNDGEQITKHGAFRIPVQSADKFAEKLGLSLQDLIKSPSLLLAGRKKLVGIKEEAGPEEKLRYWLDSYYVRGSFDDRTLVVLYREEDIL

>tr|A0A1V5UZR2|OS=Lentisphaerae bacterium ADurb.Bin242

MNMEKNSQEETLPPDSANPVPSEKTETEQSTAESPKAPFVPDKFVPHKTKLLPNCKITGIDYQTSLAVALTEFAQHEIVGVDIDLEKKTAMPFELQWNPENCEIFCHPEQAGEFEIDLYVDVIRRERHIFPFKLTVNPDPKTLWKDIPSDPNGVYAKPDSDKMFLACGENLSIVGASRRGRSHAQEGKPRDDDFIADWDAETNCAILIAADGAGSAKFSRKGSQIATRIALEKVKAAVTPDFWMTLEPALVKWNESKDAQTEKNIQAALYKVLASAAWEAKIQIKKESQEHEARYLADYKKTEKFTPRDYATTLIITVAKKRDAGNWFAATFWIGDGGQGIYRPEAGEVLVQGIPDGGEFGGQTRFLTEDSTEVWPQDANKLIERRLRFTMVDSFKAIVLMTDGVTDPKFETDNNLVSVAKWNEFWEALVKEVPFEKRDSSVADALLKWMDFWSPGNHDDRTMMLLY

>tr|A0A1V5W7C4|OS=Chloroflexi bacterium ADurb.Bin222

MKCPACGTENRDGARFCQKCGHDMGPENGITVEVADEIRSGTQPMTDAPEAPPKVAAPAAVREGPVTAPLPGLTSTFAPLPEGALVGQERYAVLEVRSTGPHINEYLVLDRQPVWVCAQCHHVSEAADERYCESCGAERGDAPAINLHYRLHESADPQAFAQEAQLLALRLQHPGLRLSTAIFTEAPYGPPRQYRVVPEFPLPSAATLSIPQELNTVLAWGVALGQALTYLHRHQVTLQTISPAHIVFDGTTPLWTNLNRAYVIPPAQRTTGEALNELLAHDIQGLASVLLYLMTGQTQLSTDMPFSPRLKTLLYQALNTPQMFMAETFTAELEACLDELRHPTSAMLVAGRRSDVGRVRSLNEDSLLTVEFTSVFKSVCEPVGLYVVADGMGGHEAGDVASELTSRVLAQRVTAELLPLLAAGKPLPDGRAWLTAAVQAANQAVYEQRIAAGNDMGNTLTMAFVRGGRALIANIGDSRCYYLDAEGIRQVTVDHSLVERLVATGQITRAEAAQHPQRNVIYRVIGDHPRTEVDLFEQLLDPGSALLLCSDGLSGMVSNEDIWKIWHTSASPQEACDRLVRAANEAGGEDNVTVIVVQMLA

>tr|A0A1V5XI91|OS=Deltaproteobacteria bacterium ADurb.Bin207

MNKSTSLPPPRFQVFVGFHTDVGRQRQKNEDALVEVGVPLGYLLAVADGMGGHDYGDRAAIMALNTIQESLRESTDQPQESLAKSLRKANAVVFKEAEEVGRTMGATCVAGLISGGKLYVAHVGDARAYLLRALTLFPLTRDHSFVQEIADAKGPTMAGNLPQNFTHIVSRSLGTQPSVDVCDREPISLSAGDVILLCSDGLTNMVDDSRIRLTLAGATPREAAKRLVDMANEAGGQDNITVVVARIDTEASLLDTQYIGLNDLRSIFVKTSDGQLHPIVSGLLDPSTWSISAVIIDLRNVKKGLLCTLSISELGPVMYGKQTVSIPQSTEALVEMAETGVWKTDAKRSQPPSAPPAKK

>tr|A0A1V5XM60|OS=Deltaproteobacteria bacterium ADurb.Bin207

MSPTVVVLIVLFALGTALAIFVFTRKRSTAADTRHDEPPKSLQPQAVLSVIPHEQLEQEAEEIAPTDYELRIFGVGRTDVGLRRKLNEDAFVCLPEHELYALADGMGAHAAGEVASKLTLEAISDAFEHNRYLSAESSEQPSHRKMRLVRILERANQIVWTMSNEVQAYRGMGTTVVMAHFSVHKRYVFIANVGDSRCYRIRDGQLTQLTTDHTLGSVGVLGKTSSLLSRAVGIEPNVEVDIMTYRTKPDDILLLCSDGLSRMVADDQICATVMDHPDLNVAADALIAKAKDAGGKDNITAVLIRMEKLPPSNHEGDSGLRDRTTLPV

>tr|A0A1V5XPE0|OS=Deltaproteobacteria bacterium ADurb.Bin207

MNNPLRVSSAGASDVGRRRKHNEDVILVREDLSLWSVTDGAGGHNAGEVAAALAARSISNYMGATVRKAWDQPIYDPFGIPNGGRRLAAAIRKANKDVLEVARHQNKYQNMVCTAVAAALCHRSMLMHVAHVGDSRCYRLRSCQLEQLTLDHSLLIEVLEREPDLDNTIVARMPRNVVTRALGIEDNVRVTFRSFPMLPGDRYLLCSDGLSGMVDHPTIEAALTEPSSPKEAVDRLIALANENGGKDNISVIVIDVDADVDLVGWQAPLATTKPETLDSDIPEIQVLGTDESDTVGQRLSLIESFPPDLIEPIGELFKKNKDDQDD

>tr|A0A1V6C0T5|OS=bacterium ADurb.Bin132

MVNIVWAGLSNTGKVKENNEDHYAIVPPQYPELPYIFAISDGVGGRKGGDLASVRAIQVISDYFVRHLPKSYNSPEVTQKAFEIANRDLRLTGSSNANLSGMGTTVVFAALFDDTLQVTWIGDSRAYVVRKNNLEQVTVDHTLPNELFKRGSITEKQLKNHPCKHHLVRALGIDETVKPEMVQIPAQNLEWLVLCTDGLYEHVSDMEIQGAIHKLNDPTLVTKKLIQLALSRGGTDNVTVVVVKIAGTQTKEDKASKNRFGIF

>tr|A0A1V6FI38|OS=Lentisphaerae bacterium ADurb.Bin082

MFRFQTSVLEPFLVNILDVEAENKAAWDKFCRIMYRTLAQVKLDLAAERGLEEKDFDFTVAYAIAGTHHIGCFQVGDGALVLRQHRVCVTAFAPQKGEFDNMTAFLRSGGEDSNHFLANLFPAQGNAGIAAVSDGPQHLMFNLADMTPGKVFTQMFDDLENGQLIRQNIMDYLTRSEWNKDLRGVDDRSLAILAPTTQTAAKAAEAAPASKSQKPKQQSAKANTAKQKPKAAAASAAAQVLKAATASAEAAQNVQEPKAAAASAEADTPSETVVDSDHSTEQPSKRLTVFVVISFVIAALLLQAVVLGAHRRAQSKSQAVLHQTLNELLMTVSKLQEQLDELVKLRQDVNTPEPDADAQATNQQQVDAQLPPDVKAVDEPIRETKAIEPDSLALPAPAPVGQSETLFNPSNVQTNSQETP

>tr|A0A1V6HT49|OS=Deltaproteobacteria bacterium ADurb.Bin058

MRVEPTQEQVGYLASIGASRSSTPQPRPREFYTSNNQATSSSDASIGGQVERQPANAPTTSFKTSKTQALPWLAAILLLAALSLVLAHKASAQQLESKPGVSMVENDFFQYVPRDGYTVFTSDLVYDNPTNVQLRSQDQYMEIGALRNELAALRQSIARLLVGFSAITILGISLVIIGRIQLRRGIRQMKQNDILKPSETDPNLQDDQNPTELKEDSQQIIQIEKNSPDPQTEENCETLPQTEVGEAKQIQTQDENQQPALAADEEGDKSPRKVSTDEVTDTMPPPSQTAVEEFGAIIAEAELAAKIRVKPTLPSARWELGLATAKGNVRSENQDYGLCFKINGYDVLIVADGCGGLPHGRQASHLAVVSAAVSVIQAYGAGYEWYRPHPKDVALRAIIDAERRLSVEGEKLQIKRSYDGLRTTLIVVIGTQHEFGVAYIGDGGGCIISTNGLVNNFLKPQRPNGVPNVISASLGPVIEGEPMSDLIPRQKGDLLIVGTDGVFDHCIKNFPKNVLRHCLLNWGNLQEATEQVLDSFASAEDQNGYIFDDNMTLGLMGDGLAPKLCQGFWEPTEATHQEAV

>tr|A0A1V6IGI8|OS=Euryarchaeota archaeon ADurb.Bin023

MNRSDKRTILTIFVIFFLSILTLSNVYCSYAPYESYEEWIISSNIKITNAENNIRDGRLNDAAENLSDIPQRILEVNEVWYKDYYTTQIKNISNEYKDSNSSIVTELDTAKYKNLGEMVAILDTILLRKDDMKQLSGFQEKVSTLHTNISTSLLEYDIICKIQEDYKKSSDLINRLKIIEAKITPENIENPEIALTIERDYTLIQEYPSYVSIFQEDGSNWVRKRASFFSDFHDILITMETVGSSKEINFDGIILELQRLEQKVPDLKLEPNTEVFVQESIKNKKAYVIELKGNQGKSKYTYILILLVSSLVAIFALVYIFFFASKKKENRGNSDVSQFLEGRTLLIFDSRRFGSQSHPRGVEIYAESDVGLKRELNEDSIGITFSKDGSKGLFVLADGMGGHNAGEVASKIAVQTAIECGKRDLLNYQALSDIDIKDILRNIVYNIHEEILHMSKSSPSMCNMGTTLEIIFLNKNHVYYAHVGDSRVYMTYTDGNSDEIISRVTTDHSELGAYMERCGVTEAEARKKVPSNVITQAVGITSAPLNPDIGDFHIGKNNWILICSDGLSDMVQDDSYIGEVLVHKQLDVASKVKELIQAAKDVGGKDNISLILFRIR

>tr|A0A1V9K9Y7|OS=Streptomyces sp. M41(2017)

MMSQMPQPTALSRCPSCEEPLEAGDRFCGACGYDLSTAPAPPQDHPTIAINGAAAGAPAAAPAAPPPPPAPSVDWPVAPEADTSDVPAPVHHAADIRGTDSGGSELPHPEHPHGDHPSGVRFDRPPEPDEYPLAPPAAGSVPAAPGAPAVPADPRTADLPAPAGGGRTCVACRSGQVDTDGYCENCGHAQPRERDHMEQELGAVAAVSDRGLRHHRNEDAFAISSTALPDGSPAVVAIVCDGVSSATRPDEASLAASRAASESLMAALPLGTHPQQALHEAIVAAAEAVNALADPSTAQGDHAPHVNAPACTIVGAVVTPALLIVGWVGDSRAYWVPVDRTSLPARLTEDDSWAAQMVAAGLMNEAEAYADERAHAITGWLGADAYELEPHTASFKPDRPGVVVVCTDGLWNYAEAAEDMAAVVPPDAAERPLHSAQVLVGHALDGGGHDNVTVAVLPFPAPVQGAGSA

>tr|A0A1W1CK47|OS=hydrothermal vent metagenome

MNSSIEEIKKFLKDWIDKDTPPDSFLSIQFNNSTKESIVYNLGLYLYKELTNEEYSGVLKRKEVQQPRVYQFLNIVLKKDEKEREVDPQILLLTIGSQNRDIIEESIDNGNYRVEKFSTVGLTRLQNQDYLGTLELDNALVLIVADGVGGAESGEIASKIAVNFMRDSFKNSFSSHMESRAVQDFLEDIVFQANQEVVEYSKKHNIDMMGTTLSVAVIVDRINLYIAHVGDTRIYELEHHSKVRQRTPDHSVREILFRSNKITQEEREEYKKNILAYCLGKSNLKRENIFVEYSILYEDSQLFLCSDGFWEKIVVKKNTFELSLEELKERIYATIPTDNVTIIRYLPKVYKSQAISVPYEEDYSEEEEKISSSNKKQFSNRKINPDKIRAKKINRIKRVVMLVTVIVIIAVVIIKFSL

>tr|A0A1W1HA99|OS=Desulfamplus magnetovallimortis

MGQILKLQGHGDKKMKKNETSADNIKSDSSTHNNLERYHKYQSLCHELLNLHDLEETDISEIKSFIQEPSATAFFKNMAQHFIELWESWQKTFDNYRKSESALEFKDALDTKNRKKSYESQNNKKRDKSKAVREIESIKENPNVKDTSKGVIQPGFSAPCNKKTDKETSLDTPKADHNTTSFPATENDKSSSSSIEDDKDSSTATDDDKSSSAAIEDYKNSSPATDYDKSSSSVIEDYKISSSATEDDKTSSNRLLMPALKFPNATCGKPYSEKVEVSRQSDHEKAFGKSSDSSEDHIKILSIKGLEAAGLSYNAKEQLISGTPSASGEIKLEITFELSSSESEKTCTTESIFIVNPDPRSLWQNKPSDKSVLFWKEDEYKEEIVTAKGWSITGASKRGRSHAHEGKTRDDHFFISTDSSTEWDILAVADGAGSASLSREGAKTAVMESSRILKEKLKEYDTEIINLLLGLGDNDFQKAQGSEEAQNSGQTKNSEKDKGFKKEDDSAKNQEKKLKNILYNVFSRAVYEPVKVIHDTVENLKKQKSLHEKTTYEKTTYEKTANGKTTNGKTTNGKTAYGKTAYEKTANGKTAYGKTANGKTTYGNPSHEIEPENIKFRDFHTTLLLAAHKEIEGKHFIASYWIGDGAVAVYCEGKSITILGEGDSGEFAGQTRFLDNSAVTSEDIYKRIQFDFKESITALILMTDGITDPFFETDHNLNQIDFWDDLWKNQLKPQISGIKGETAQNILNWLDFWSQGNHDDRTVAMLFKKSE

>tr|A0A1W1X261|OS=Andreprevotia lacus DSM 23236

MVNLTQALDITGLTDSGQVREHNEDAIGFDAADGFVVLADGMGGYNAGEVASGIAVEVIAHLLREMLLKNAPERALQAHGQPAAYDMLEHAVRQANASIYGTAQSQPQCAGMGTTVVVGLYFDNRVLVAHVGDSRLYRLRGGALEQITRDHSLLQEQLDSGLITAEEARHSANRNLVTRAVGIDPDVAVDLSEFETQPGDLYLFCSDGLSDMLEDGDIADTLNTLRANLPLAAEQLVQMANDAGGRDNISVVLVGIKRDYPAQSGLWSRLTSWLG

>tr|A0A1W1YF31|OS=Oscillospiraceae bacterium

MVDLSVAAVTVIGQKHRQRGVPCEDASVAVTSNGVSAVVVADGAGSKQYTHARFGSAAAVNTIAKLLTEHFDALYNENREAAVRSLIIAALHVKFADLIVEHKIDSIERLSCTLLFCAVKDRRMIAGHIGDGLIVKVTPSGLSPLTMPQNGVSSSSTYFVTANHAADYLRLIKTTVDDVHAIALMTDGVQDSVYDENSGLIKPVVARMADTLASGRQKAEGELKGIIEKFVVGSSNNSDDASFGVIYFNGTKGPDPKSLPTGADQFPRSTETFKDLQTSLVPDVKRAKKIIVEAAANAPVILKEEEKPSKKTGETENDAAKENSSKPINVRPVIKKSSILMWIIVIMELAAIIWLILKLYILKV

>tr|A0A1W9GXM9|OS=Proteobacteria bacterium SG_bin4

MTNFQFTAFSQRGRGKSRNEDAVLLDNQVYQGGVREGGMVDTAQPRYFAIADGVAIGTLPRLASRRLLEILRDHLASASATESLPPLLHRVQQDYVALSANPRFHGMASTLVGVRLLGNTAAIFNVGDSRAYLLADGRADLLSRDHSLLNDLMDDGEITAEQTKDAASILQGLTCQFLADAECADFRVNTATHELQRGERILLCSDGLNEALDDAQIASLFSNQNETDLAEVFKAARRAGGSDDFSVIVLAGEN

>tr|A0A1W9Q7V4|OS=Sorangiineae bacterium NIC37A_2

MSTDVTFSALTDVGRKREHNEDNYLVDKKLGLYVVCDGMGGHAAGEVASALAVRSFHEEVKREAEMLSDFALGKTGADRVTTRDILNMLEFAANRASARVYAEAAADESRRGMGTTLVAALICGKQLFVTWVGDSRLYLLRDGVLEQVTEDHNVLNELVKRRKMPREKVEQLAQKNAVTRAIGVYEHAEPESIAIDWLPGDRLLLCSDGLYQYFEDDLDRLAEKMSLDDIELAARLLIDAANEAGGSDNITTVLVALGSGEARDDERARKLQLKRELLARMRLFRPLSDRELLRVLQVTDVLSYEPDQVVMEQGAPGEELYIVLEGSVKVFRGDTAIATLSPGDHVGEMALVRNQPRSATVKSVGKSELMVIRRRDFFELLRTEHALAVKLLWQFLGVVADRLAETSRELGQVREELLAEDLTDSLFDDDPDDFDDRPTLHLGRTSTNHDESPPSQSGVVMSEPSRPMTPPPPSVRGVPPVKGSTPSSLPPQNPSLVRNARPLPGSPPPKNEEETVPPPSVRGPGPERPSVPRPPRRSSATPLTRTPSLRPVPPAPSPESASSDEDKSDEPDPEDKS

>tr|A0A1W9QA39|OS=Sorangiineae bacterium NIC37A_2

MGQKNARIAWGIAPRAPRRPKTLGGPIGAPQQRLEPLAFLNPGEPMHGVFRTDGLECAYSVAPCPIGSGPNEDSALIVPSKNGCFLVVADGAGGHPEGEKASALAVLELRERLASLDESRSPQSIIIDGFDAASDRIVTGTRGALTTLLVVQVELRPQGLHFRSFHAGDSAALVFSARGQVRFSTVMHSPVGYAVESGLLDPDLALHHEDLNLVTSLVGGPEMRLEIGPWMLLKPRETLLVATDGLLDNVYRRELAQAARGKIELAEERLRALVRRRMVEPGSPLHKPDDCSFMLLRARST

>tr|A0A1W9TR25|OS=Desulfobacteraceae bacterium 4572_187

MEKLLQVSDEIARELCGGSKDAVKPLSNLLRMEIEKLLTLEVLPEDLAQGFSEDPFEKGTVLKKWCMENRHNGNFPENVFLLLSTVAKYMDIRGASANPLLAFVPDWFSVDGAGKIIIAEPLLKLATPDKGLGRLKDFQRSLLKPYLHPNLLKSLRNCEFRTALVFSFSLFLLDLFLDVQGRSIAELKDGLKKCGAKNHAIPPGLAEAFTTIIAVAPNQEHHTSCEDMVEKASCFFRENPFNCGVMVDPQFKHDVFGYSVPGLNKKMNSNEDRFLSRTHNNITFFMVADGVSTADIGTGEIAAEEVVRLFKTDFFKRFETLAVKLEQNIADDPAFVWYDPADDFLVRFFREASERVTTELNKFYKTENFKPPFQAPMCTTLSAAVVVYDQAAVRYAGDSPVMLFSPERNIFRKLTIDHHHGIEQEFTMADTPDSDALTRVIGARDFSQTEHKFVPSAEEGDPLRVQLKKGDMLLLASDGLIDCIDALTPEQKIDRIEKHIRKFSDDDMRLNELVRQLVALGENELSHDNITLNLLKSSNVGVTSQP

>tr|A0A1X1Q7N6|OS=Anaerovibrio sp. JC8

MHIGDGVIGYTKDNKIKIASYPDNGEFSNVTVFTTSSDAIFSMKLLKGELNGIDSFILMSDGTEAGLYHKKNKSLTSALVRVVDFVRFFPELTVRGMLVDSLKNVIQQVTVDDCSIAIIADDYGKDVRHLPISEQRDLLAVGSKKYPAHKSKRMNRINYILSMLEQPIAIDEIARRLHIKKKYVRKYTDFLESKGIIEQCGNKFIYLR

>tr|A0A1X7UGS7|OS=Amphimedon queenslandica

MFKRRPQVEKQFISGKSTRDVPEAIQLASFKKEPPLAAYTGPNTGLRVLDTDTEISTSDQCVAGVNDWNNPSDYAYGLSVSLYETDKRCNRQVGGPVADVFGVVVRENNAIMAIADGSGWGKKARLAARCAVNGAIAHITDNLHKLSVKKPTSATLNKLLHESMDIAHKSILSHGGTLTTLSIAVACELSAPSNQWGLFVASVGDSPVFVYCPHTYQLHEATVDCHSKDGNRVVQLSGGALGPAIGTLPDLDNFSMSYSPVYPGDIVLLMTDGVYDNFAPGVVQAVHDERALDCDSGVSFTPPNASPVPGPNEGSSSPVLNRQTCCPSPSNPHTTERPWPPCDVDLHRSISSAHPPSSVYAEEDTQLIELKKCCESLPEMMKFLHQHQANLCGNMTAQTVSSAIINFVYEATEKKRIFKSDCLEKDINVSRRRRADPEFASELQSHKSKLDHATIVSYCIGTH

>tr|A0A1Y0D8R9|OS=Oceanisphaera profunda

MNNDTQQAEQKGIIMRAKESASAGVTHLEPEANEFVVSHVDPLANNAKIPDSATADSAPNEVKRIPHTPFGTFPLGVLPFGFGMTFTPSHIAKLIAGGEGAVDSKTEQEASIAMEVVAETAFEKSIASDQVLEGVAAPSVIIPEAKSLKHKAQESTAQETMAHELSASVLEQQSKVEPQEAEPAKTTHSLKKTKAAKKASLSKPLACVINHQWQSMHEQVAGRSHRNALQPMPCQDVAFSAIQPRPILLLADGAGSAAVSEQGAQALVSGISRLLNTLEQQVASLLDQPTSTAAEARQFCLLLIKHGIGLLTDKASEQRRAVKDFNATFLVAILGKENWLWLRVGDGGLVMETMTVEQNAEATAGAKMQLSPTLTVLGSSGKGEFANQTTFVNAHLQPEQVQSGFMPIAGLTGLALMSDGAAERLVAYDGSRASSQLSQWFHSLRAGKLKRHTLVQRLVAEEFVTGMSGDDCSLALLATEFVME

>tr|A0A1Y0ENW6|OS=Comamonas serinivorans

MKFSVFQLTRRGGRDKNEDRMGYCYTREAALFVLADGMGGHPEGEVAAQLALQAMSAMFQRDAKPTVANPEEFLGAALLAAHHQIIRYAAERGFLDSPRTTLVACLLQDNQVTWVHCGDSRLYVVRDGSLLTRTRDHSYAEQQASGVFKLGNVNRNVLFTCLGSPSRPVFDVGGPIKLQQGDRFLLCSDGLWGTVEDDEIAGQLASKPVSEAVPDLVELALVRGGSTGDNVTVISAEWQMPTVVDSHLDSITTDLISEDVFASTIQASLVDSSYDDLDDDAIERSIAEINAAIARSSARRQT

>tr|A0A1Y1KAH4|OS=Photinus pyralis

MPSLRKRVSSYFRQLSFYNEPREKKYNVNENSFVTKYLEGQITVKDGSPILYGKNPTDLPNYDLGLYDVGPTTIVGCYSGPNGGLTTVKRTEKHLSVPDTDIDFIDTQDEVEVPVKKTSRKHVDNTTMLIKVAGQEYLVTNRKKKENKSHSLGSLSDLEVEKTKICTDNNNSVTVKCKNQEIAAGRGRQEQNERRKGVLVVDATPARPSYGLSKSKSEGNPFQQKKKGKIVRLATRPSDGATGGGEDDPILPESWNRKTDYAFGISDSLYDRNQVTKNRNGDPIADCFGIIARGDSAILAVADGVNWGEKASIAAKSAIHGCLHYLDKTIFNDTKPFDEISASKADAIISQNRVSNTREVFVCLLRAFHCAHDLILENQGMLTTLTVAVVLPLKVKDEVESVCCVCNVGDTLAYVYSHKYGVRELTKGSHDVNCNRDMRDALGALGPVDGINPELSNLTLSITTVHKGDIIMVASDGLTDNFDPNVCKFTVNTKDALKPKRSVAKDAHATSSKEAAPAKPPRKTKPENVKTTDNPLKVQFLRENSLEAEGRAHSTFNPRPRQP

>tr|A0A1Y1KFH7|OS=Photinus pyralis

MPSLRKRVSSYFRQLSFYNEPREKKYNVNENSFVTKYLEGQITVKDGSPILYGKNPTDLPNYDLGLYDVGPTTIVGCYSGPNGGLTTVKRTEKHLSVPDTDIDFIDTQDEVEGATGGGEDDPILPESWNRKTDYAFGISDSLYDRNQVTKNRNGDPIADCFGIIARGDSAILAVADGVNWGEKASIAAKSAIHGCLHYLDKTIFNDTKPFDEISASKADAIISQNRVSNTREVFVCLLRAFHCAHDLILENQGMLTTLTVAVVLPLKVKDEVESVCCVCNVGDTLAYVYSHKYGVRELTKGSHDVNCNRDMRDALGALGPVDGINPELSNLTLSITTVHKGDIIMVASDGLTDNFDPNVCKFTVNTKDALKPKRSVAKDAHATSSKEAAPAKPPRKTKPENVKTTDNPLKVQFLRENSLEAEGRAHSTFNPRPRQP

>tr|A0A1Y1RN94|OS=Rothia nasimurium

MTTAPQLTLYAGKSTDVGQFRDNNEDSMVVAGTLCVVADGMGGHEKGEVASRLCTRTLAYAQVFTRPGGMNEQEQQAFEADLATEALGDDPGGRRLRKRRKETGALNRQILRTLDKIKEIIGEADEAIKSALDLRAGTTVTGAWLTHIGEQQMWFIFNVGDSRTYRLVRTEDAETEGALSVSHGPVDGLELEQITVDHSEVQYLVDTGQITALEALTHPRRNVITRALGTGNYWEPDFWVIPARAGDRLMLCSDGLSGELSHNYMARVLATVEHPQDAADVLQMAALRAGGRDNITVIVADAITVEEAAARAEELKALTATDAHRIVANIDGYDYQHTTTEEEERFHSEKELTRDTKKA

>tr|A0A1Y1SAX1|OS=Oceanococcus atlanticus

MGGHADGALAAQCVMDVAARIFADEAQQPAAQLIQRIGLESHLAIQSVAPDLSERQQPRTTVVCAVVDGPNVLFAHAGDSRAYHLRRGEIVHVTRDHSAVAMMVRRGDIRPQDARHHPMRNQVTRCLGGGGPPPALELTPCPPLQPGDTLLLCSDGFWDPLEPDELGSNIELTELAQRAVARNPGSADNCTALRIRFESV

>tr|A0A1Y2ET10|OS=Neocallimastix californiae

MTAIDTYTNTPDLFNIQKLNDREEEQEIKYRNPKQYILKDFNDKKNAFIVKSESTATLYNKDNEIVQPIGIVWSVTSEQGYRKKNGERTKLHETIEDFHWPKRNDHAFERSHPINGKSIKMYILADGHGGNEAPQIFLARACASCFKVMEDKNWDLSKEDHQKELKKILTDTYIKLDKQFCEEKVLEYEEYNRNGKEGKKPVDDGCTLIVNIIYDGYLINVNTGDSRTVMGSKKEKTHFLQKATYEYSPVFSSLDQSPDHPEKAYNVSHNGGIFLSSGGIPQRRPNIEPFEKRGMVTYPELVGTRIYRPSNKRIEELGINHLMTLNMGGSMGDLLFKIEPAVISCRPDVSIVKIDSSVDNMLLIATDGIWDHLNWQEPEKQNEKIMSYISKQLGDGAEVSEELLNEVVSKLVKRENNLDLYEPRQRYDDCTAFLVFIPAGSEKENVEASS

>tr|A0A1Y2JZK2|OS=Magnetofaba australis IT-1

MARFEAYGHTATGPVREHNEDHLLLDRYVKNRGRMTLWLDADDEALQSVGVLLAVADGIGGASAGEVASQLALRTLERHFHGVTKAAPEAFVETLRDAIQRANDTVLQAAASRPEWARMGCTLSGACLTPHGYWVFNAGDSRVYRLRNGLLRLLTLDDSATRMAMDAGQLSQDQAEQSGQRHVLTNHVGLASFQCRIEPGPELRGGDVLLVCSDGVHDLLGESRMEALCAADDAPLERMGQALLDAATAAGGHDNQSVILLRLHEDSAG

>tr|A0A1Y2KAT6|OS=Magnetofaba australis IT-1

MVVSDGMGGHDHGEVASREVIAALREYLMSPGTFGRKQTIRSDDTYPDEAPTVVNTIATAETDDEEEDGPTQDDVPNPAVTLVRHAVEYANSKVFEENQKRKYPEGTGMGATVAGLWLSNYEDRLVVFHVGDSRVYRFREGDFSQVTRDHSMIQQWRDFGERGAQPSQNILTQAMGPAANVVPDVALVDDQAGDCYLICSDGLSNMVDDAVLAEKLGKATEENLEEIGDELIELAKHNGGKDNITLILGLVVA

>tr|A0A1Y3WKL3|OS=Faecalibacterium sp. An58

MISFLKHLKNKQEKAVSSSKRAKSGMALDDITQTLILPLDSEYENCKDQNLLTSQENQTLEVAVCQSIGTRNKQQDTIRFQVNDQGAVCVVCDGMGGLSGGEMASTISAEGMLQALLSREDNVPLTMARASVSLNEQVKGLRDDQNHPIEAGTTLTAIVAEDHKLFWCSLGDSRIYLWRNGTMEQLNEDNTLGVQLDKMVKDGQITEIEAMQHPKRAALTGYLGMPSLSQIGGSRKAVQLYPRDIILQCTDGLYRALPQEEICSILGEDNENLQEIADKLVRYALWTDGPHDNTSVALTRIKE

>tr|A0A1Y3YP31|OS=Pseudoflavonifractor sp. An44

MKTSKKLFSEGMKILNGPNFWTSSQNYMNNDTIAAACLTHIGKRLNQEDNFLFNGVYLTSELQTQLTYRHCYFSSVAPASKVQLFAVSDGMGGHNAGEVASRICVEKLALAHKELQQYSSIKAAVSYLQQLIADINNTVCEMSRRQPEWKGMGATLVLLVVCGAECAVLNVGDSRAYQFSNHQLDRITKDHTEGQRMLDLGLLTRKELSGFPARKHLNRYIGYGQSGYVLQADEYYPALENGVILLCSDGISDFIPEARILEILDSEHEPVIAGKQLISEAVTATNADNATAILIPFRR

>tr|A0A1Y4H0G9|OS=Anaerofilum sp. An201

MDRNKPAAPETAVSPLPPAEDFPLPPTQTGKRVVDGVFSAIALSQQGASHLAKTPPVPCQDYSDLRLLEEPGLLLACISDGVGSCSFSHWGAYLAVNGALDSAEAALRQAAQEDAAFFSAPPTVAALLRGAMHAALAGIEAKARADGIDPMQMQCTLTLCVYDGRTVYFAHAGDGGIVAQFSDAQGGVEMITNRIKGDEAGSVFPLQVGEQGWMVGMSNRPIAGFVMATDGVLDGFVGDRRLSDMYIYYPFMKHALYSLLPDGQEDAGDCADRALSQQQELLLCEEYRSQRQDDLSLIAVVNRSALADAPQPAFDLDRWEELRRERERRLMEVLYGAAKTAPAPDEEPNEEKGEEPAPSPAGEPGEEPAPAPAGEQGEEPATAPTEKPGEEPAPAPDEAPILFPERPPFVHRFRGFRAQVRKRRLHQKKKRFGV

>tr|A0A1Y4H122|OS=Anaerofilum sp. An201

MPSVQFSAAPDPHTDWHTRPVCRSVFCDGVFAFNLSAQGESHIQQHVPCQDHSRVELVHTADGLFAVAAAADGVGSCRLSHYGSCIAVNVAVTYLAVFFQQHPHPGETGCPAEVLPEAFALSQQAVERYAQDHQLLAYSFFSTLTVAVYHNGTLWYGHIGDDGIVALSRDGAWRLLTTRHKGDEASSVRPLQSGSEAWEFGCAQDISAFALLTDGLLDRVVGDAYTDCMVYAPFFTALLPSGRADAGEVEALFARWTAALTGEEGRQGITDDLTVAVAGDTAALCRTAVQPFEESQWNERIERRSRVIHEKLYGQPVQDAPTESPVGEAPSGCDDDPIAQLEAITARHQQAVRAQQQSPPSPRSPHHERRPSTAKRRARRQ

>tr|A0A1Y4HD84|OS=Anaerofilum sp. An201

MNDHFEQARPASEAPSHPDAQPDPPAPPADPAQTASDPPAQTGRADSYGNGMFSVVALSLQGASHEKKNIPCQDYCNYLYIEELQILVAAVADGVGSCALSHWGAYTAVERALSFLKEALLAPGEDGRPRGMASSQEGGRMMREAFSAALEAVEDKAAEMQRLSYSFQSTLTVAIYDGKTLLYGHAGDDGIVAQQADGSYGMTTSRMKGEEASSVCPLQIGPDKWQVGVTINEDISGFLMATDGVLDSFVDSAAHHSRVYYPFMESVLYPLCADGETPGENARQVLDKLRETLLSDGYRRQVTDDITLLAVTNNLLMNNGVRPVFDQKKWDEDTAAYARQVHEKLYGTQPAAPPQEPAA

>tr|A0A1Y4IHV3|OS=Lachnoclostridium sp. An196

MRYFISCCTDEGRRKKGNQDSILVQRAVSGREQAVLAVLCDGMGGLDRGDMASASVVKAFSGWMEKELSRLRWTRKKKIFLALDRVLKKADRRLRAYSGRYGLRMGTTATVLLLFRGMYYIAHVGDSRVYELGKKARQVTSDQTLVAEEVKRGLLTEEQAGNDPRRNILLQCVGGNGCIPEHICGTVKEGAVYLVCSDGFCHEISAEEMKDTFCPEQMQDEETLEERCRILVERNLERGERDNISVIAIRTEKERRNAGNRFGH

>tr|A0A1Y4K113|OS=Olsenella sp. An188

MLHTYGASVVGPWHAEAGVPCQDSNASWVSEDGTLAAAAVSDGLGSELHSDVGSEVASRVAVDYCAEHARPGADAQETIEVLRAAYRAAYLAVLDCAAEMGEPAGEFDATLSLVLLDGDRLFWGQSGDSGIVAGMTDGTYVLVTDKQRDGEGRVYPLCFDTCWDFGVLEGAATALLCTDGVLEGMIAPPILAANSDCPLDRSKARMFLHPLADDAKHLDEVQRQVAAYLESYPRDLIDDDKTVVVVFDDERFPESQPEDYYAEPDYEAILERAYQNLYRGDRQKSSGAARGDGAAEPASLEGGAPVAPEGAGTPDPRADPDEGAPGESPAVRTICRMGGTVAKSLRDSTPALKRAAGAALEAGAVIGRGCIRAIDEALDAVEGTAPPPPEDAGSSRPRA

>tr|A0A1Y4RY85|OS=Lachnoclostridium sp. An14

MVDYKAFHISTKGTGNQTPLKEYSAAQTAGNAQIAVIASGLEENLCFRSHLGAKFALEVALQAGKTFAEEMRADRLFRDAQATEQALRQLEGSIIVGWQDRVQKHWSGSPFKEEEIESIGNTPELERPKAVYRSGRNVEYAYSAHIIMAIAAKDYVLVIRNGNSGCVFLSEDGTGKEPLPWNEKNTDLYCTTFCNREALREFRHYCGKKTPDMVWLSSAGVERSFSEKSKFLEFCNYTAKGIKNPAEDSEEYLKEELVKLSRRGSHEDMSLAFLWMANIVKKTVLENQRKKEQQSQAVQAEKTDKKETKETPKKEEPKKTEPEKAPEPKPKKVSEPKTEPKPVKKETAPKTAPAGGQQAAKKKSPLPLILLALVVAAAAWMLFGGSKEPEPKEAETRTETVAETAEVITVPETEEIPETSPQETEEETQEETEAETEEETVPETSPQEETTEAPTTAAPTTAAPAPTPAPTPAPTTAAPAPTPAPTPAPTQPPETPAPTPAPTQPPETPAPTTAPAAPTLPGPNIIW

>tr|A0A1Y4TNC2|OS=Gemmiger sp. An120

MKLAAKTDIGYGRLENQDNYRAARLPDDTVWGLICDGMGGANSGKLASQLAAQALEEYFDQGLADLVPGQEIEFLRKAVQQANQAIYEEASRHPEHTGMGTTVAGALVRGGHAWLFHAGDSRVYLFRAGQIRQLTRDHSMVQELVENGTITAQQAATHPRKNIITRALGVNPTVETETGECTVRPGDVLLLCSDGLSNPVSDRTMARILTEVPFYEAADVLVAKALEHGGQDNITVLLIGVEPVAAPAP

>tr|A0A1Y4W4F6|OS=Flavonifractor sp. An100
[truncated: 1,271,910 more chars]
