# Supplementary figures and images for: Evolution and classification of Ser/Thr phosphatase PP2C family in bacteria: Sequence conservation, structures, domain distribution
Source: PLoS One. 2025 May 19;20(5):e0322880. doi: 10.1371/journal.pone.0322880 (PMC12088040; doi:10.1371/journal.pone.0322880)

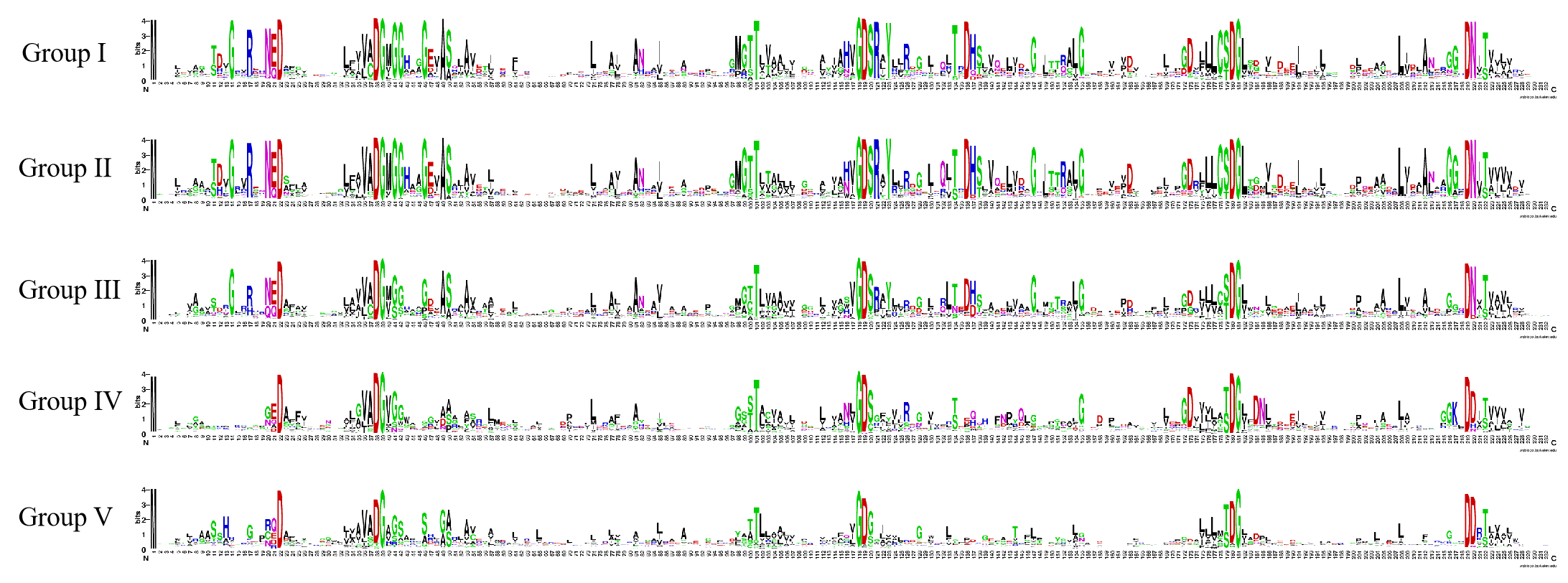

Supplement: S2 Fig — The height of different amino acids represents repeatability. The scale bar at the bottom indicates the length of the motif protein sequence. (PNG) [file pone.0322880.s002.png]

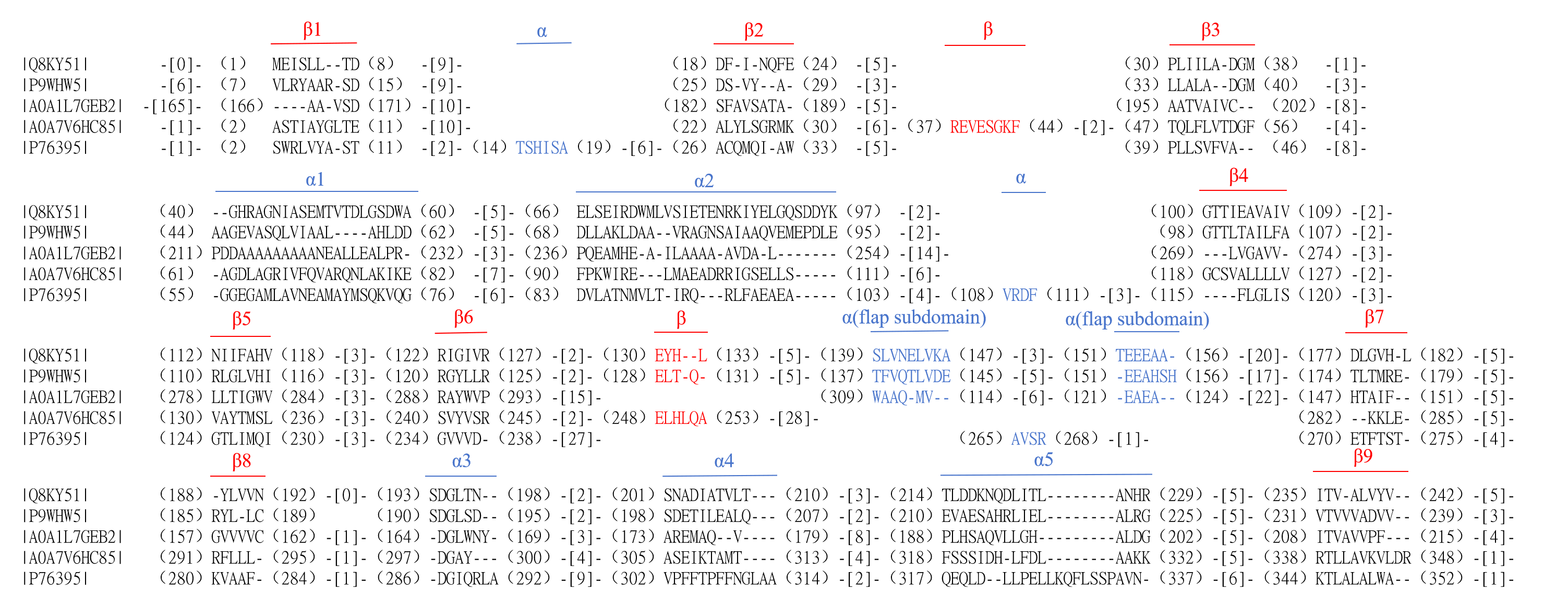

Supplement: S3 Fig — Structural sequence alignment of five representative proteins. The sequences covered by the red short lines form β-strands, while the sequences covered by the blue short lines form α-helices. Additionally, the α-helices corresponding to the flap subdomain are also marked in blue. (PNG) [file pone.0322880.s003.png]
